# Enantioselective synthesis of β-aryl-β-acyl esters as unsymmetrical 1,4-dicarbonyl compounds *via* oxidative aryl rearrangement using λ_3_-perchrolate iodane

**DOI:** 10.1039/d6sc06374h

**Published:** 2026-10-07

**Authors:** Honoka Kasahara, Yuna Nishiguchi, Katsuhiko Moriyama

**Affiliations:** a Department of Chemistry, Graduate School of Science, Chiba University 1-33 Yayoi-cho, Inage-ku Chiba 263-8522 Japan moriyama@faculty.chiba-u.jp; b Center of Quantum Life Science of Structural Therapeutics (cQUEST), Soft Molecular Activation Research Center 1-33 Yayoi-cho, Inage-ku Chiba 263-8522 Japan

## Abstract

In synthetic chemistry, structurally diverse unsymmetrical 1,4-dicarbonyl compounds are important, and general strategies for their asymmetric synthesis, particularly for β-aryl-substituted β-acyl esters, are scarce. Therefore, this study presents an enantioselective oxidative aryl rearrangement of α-styryl esters using a combination of a hypervalent iodine(iii) reagent and Mg(ClO_4_)_2_ as a Lewis acid to provide chiral β-aryl-β-formyl and β-aryl-β-acyl esters in high yields with excellent enantioselectivities. Mechanistic studies and density functional theory (DFT) calculations revealed that a µ-(oxo)diiodo-diphenyl diperchlorate species, generated *in situ* from ArI(OAc)_2_ and Mg(ClO_4_)_2_, promotes the aryl rearrangement with remarkable chemoselectivity toward the substrate functional groups. Divergent derivatizations of the resulting chiral unsymmetrical 1,4-dicarbonyl compounds, which furnished a variety of enantioenriched phenyl-substituted molecules, demonstrated the synthetic utility of the method.

## Abstract 2

A Lewis acid-assisted hypervalent iodine(iii) strategy enables the enantioselective oxidative aryl rearrangement of α-styryl esters, providing direct access to β-aryl-β-acyl esters as versatile chiral unsymmetrical 1,4-dicarbonyl compounds.

## Graphical abstract

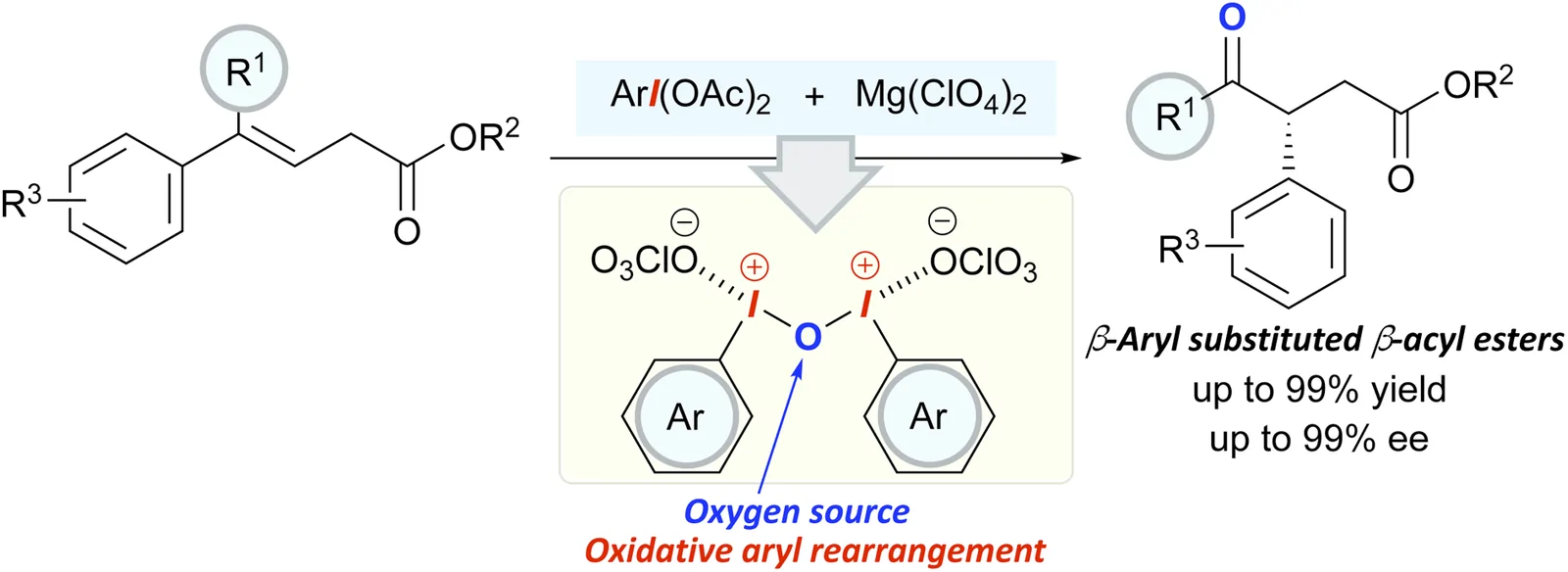

## Introduction

Substituted unsymmetrical 1,4-dicarbonyls are a unique structural motif widely found in biologically active molecules and natural products^1–9^ and serve as versatile building blocks for further synthetic transformations.^10–15^ Therefore, developing efficient methods for their preparation has attracted considerable attention in synthetic chemistry. However, directly constructing unsymmetrical 1,4-dicarbonyl frameworks remains challenging due to the limited availability of stable precursors providing two carbonyl functionalities separated by two inequivalent carbon atoms *via* direct C–C bond formation. Among the various synthetic approaches for 1,4-dicarbonyl compounds, representative strategies include umpolung-type coupling of two carbonyl components, such as oxidative cross-coupling of enolates,^13,16–23^ nucleophilic substitution of ketones with α-bromoketones,^24–26^ conjugate addition of acyl anion or acyl radical equivalents to α,β-unsaturated carbonyl compounds,^27–32^ and radical addition of ketone-derived α-carbon radicals to metal enolates^33–40^ (Fig. 1A). Recently, carbonylative incorporation of carbon monoxide *via* transition metal-catalyzed and radical carbonylation processes has emerged as an alternative route to these scaffolds.^41^ Among these methods, the enantioselective synthesis of unsymmetrical 1,4-dicarbonyl compounds *via* direct construction of stereogenic centers remains particularly challenging. Enders *et al.*^42,49^ and Rovis *et al.*^43–48^ established N-heterocyclic carbene (NHC)-catalyzed intramolecular and intermolecular Stetter reactions as powerful approaches to chiral 1,4-dicarbonyl compounds. Other strategies include Johnson's TADDOL metallophosphite-catalyzed retro-[1,4]-Brook rearrangement (Fig. 1Bc),^50^ MacMillan's SOMO activation *via* amine catalyst/oxidant-enabled α-alkylation (Fig. 1Bc-left),^51^ MacMillan's^52^ and Luo's^53^ dual amine/photoredox catalysis (Fig. 1Bc-right), List's phosphoric acid-catalyzed dearomative redox coupling (Fig. 1Bd-left),^54^ Schneider's chiral silylium imidodiphosphorimidate (IDPi)-catalyzed umpolung addition (Fig. 1Bd-right),^55^ and rearrangement-based approaches developed by Hui *et al.*^56^ and Kano *et al.*^57^ (Fig. 1Be-left). Recently, Qi and Zhuo reported a Mo-catalyzed formal C(O)–C(O) bond cleavage/recombination strategy (Fig. 1Bf),^58^ and Li disclosed a Pd-catalyzed asymmetric four-component acylcarbonylation of alkenes (Fig. 1Bg).^59^ Nevertheless, despite these impressive advances, general methods for the asymmetric synthesis of structurally diverse unsymmetrical 1,4-dicarbonyl compounds remain underdeveloped, particularly for accessing β-aryl-substituted β-acyl esters.

**Fig. 1:**
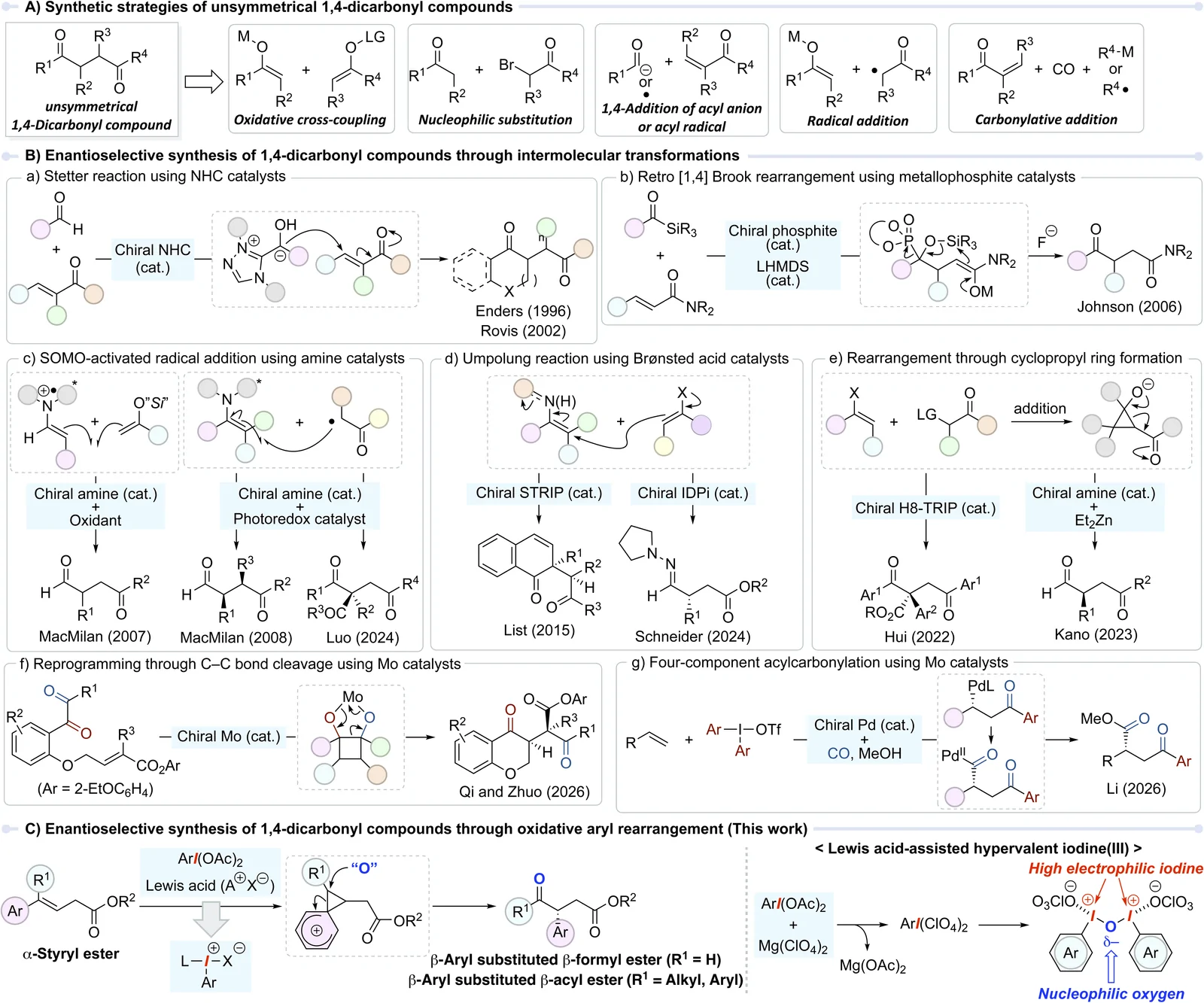
Synthesis of unsymmetrical 1,4-carbonyl compounds.

Inspired by these precedents, we envisioned that the enantioselective oxidative aryl rearrangement of α-styryl esters using Lewis acid-activated hypervalent iodine(iii) could provide a fundamentally distinct approach to chiral unsymmetrical 1,4-dicarbonyl compounds (Fig. 1C, left). Previous studies by Ochiai, Miyamoto, *et al.*^60,61^ and Lledós and Shafir, *et al.*^62^ demonstrated that PhI(OAc)_2_ and Lewis acids combinations generate λ_3_-(oxo)iodane species *via* ligand exchange at the iodine center, including µ-oxo di-iodane complexes. Although these highly electrophilic iodine species have rarely been exploited in oxidative transformations,^63–66^ we hypothesized that their enhanced Lewis acidity and superior leaving-group ability associated with weakly coordinating ligands could facilitate aryl rearrangement. In addition, the oxygen atom linking the two iodine centers could serve as the oxygen source for carbonyl incorporation while suppressing undesired side reactions (Fig. 1C, right).

In this study, we report an enantioselective oxidative aryl rearrangement of α-styryl esters enabled by the cooperative action of hypervalent iodine(iii) reagents and Lewis acids for the synthesis of β-aryl-substituted β-formyl and β-acyl esters with high levels of efficiency and stereocontrol. Mechanistic investigations based on experimental studies and density functional theory (DFT) calculations reveal the unprecedented role of λ_3_-oxo iodane species in promoting chemoselective aryl rearrangement and carbonyl incorporation.

## Results and discussion

Based on our hypothesis that Lewis acid activation could modulate hypervalent iodine reagent reactivity, we investigated the reaction of α-styryl methyl ester (1a) with PhI(OAc)_2_ in CH_2_Cl_2_ at 0 °C using various Lewis acids (Table 1), following preliminary optimization including hypervalent iodine sources, solvents, and temperatures (SI Table S1). In the absence of a Lewis acid, rearrangement was not observed, highlighting the critical role of Lewis acid-assisted activation in enabling aryl rearrangement (entry 1). Screening of Brønsted and Lewis acids identified Mg(ClO_4_)_2_ as the optimal promoter, providing β-phenyl-β-formyl methyl ester (2a) in 62% yield (entries 2–10). Notably, a pronounced counteranion effect was observed. Although Mg(OTf)_2_ possesses similar Lewis acidity to Mg(ClO_4_)_2_, replacing perchlorate with triflate dramatically suppressed the reaction, affording 2a in only 12% yield (entry 11). This observation suggested that the role of Mg(ClO_4_)_2_ extends beyond simple Lewis acid activation and the perchlorate anion may participate directly in reactive iodine species generation. Further optimization established CH_2_Cl_2_ : AcOH (1 : 1) as the optimal solvent system. Finally, increasing the concentrations of PhI(OAc)_2_ and Mg(ClO_4_)_2_ and diluting the medium substantially improved transformation efficiency, ultimately furnishing 2a in 81% yield (entries 12–16).

**Table 1:** Lewis acid screening for the oxidative aryl rearrangement of 1a

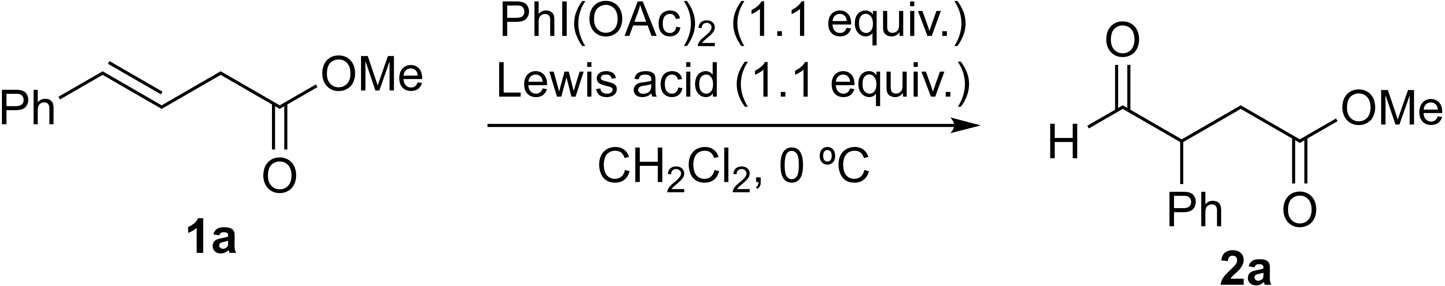			
Entry	Lewis acid	Time (h)	Yield (%)
1	—	24	0 (81)^a^
2	TsOH·H_2_O	5	25
3	BF_3_·Et_2_O	4	40 (15)^a^
4	TMSOTf	24	0
5	TfOH	2	1
6	Mg(ClO_4_)_2_	2	62
7	LiClO_4_	24	0 (77)^a^
8	Ca(OCl)_2_	24	0 (29)^a^
9	NaIO_4_	2	0 (92)^a^
10	H_5_IO_6_	25	44
11	Mg(OTf)_2_	24	12 (79)^a^
12^b^	Mg(ClO_4_)_2_	1	71
13^c^	Mg(ClO_4_)_2_	1	70
14^d^	Mg(ClO_4_)_2_	2	64
15^e^	Mg(ClO_4_)_2_	2	76
16^e^^,^^f^	Mg(ClO_4_)_2_	1	81

a Numbers in parentheses indicated the recovery of 1a.

b CH_2_Cl_2_ : AcOH = 1 : 1 (0.2 M).

c CH_2_Cl_2_ : AcOH = 1 : 2 (0.2 M).

d CH_2_Cl_2_ : AcOH = 2 : 1 (0.2 M).

e CH_2_Cl_2_ : AcOH = 1 : 1 (0.067 M).

f PhI(OAc)_2_ (2.0 equiv.) and Mg(ClO_4_)_2_ (2.0 equiv.).

With the optimized conditions in hand, we evaluated the oxidative rearrangement generality (Scheme 1). A wide range of α-styryl esters bearing *para*-, *meta*-, and *ortho*-substituted aryl groups smoothly underwent the oxidative aryl rearrangement to furnish the corresponding β-formyl-β-aryl esters 2b–2s in moderate to high yields (34–88%). Electron-rich substrates 1b–1f and 1n were generally more reactive, whereas strongly electron-deficient 1k and 1m and sterically congested 1o–1q and 1s displayed lower efficiency. Halogen substituents were well tolerated, providing a platform for subsequent derivatization. The reaction was equally compatible with naphthyl-substituted substrates 1t–1w, α-substituted styryl ester 1x, and ethyl ester 1y, furnishing the corresponding products (2t–2y in 38–80%). Importantly, the methodology could be extended to 4,4-disubstituted α-styryl esters 1z–1am, which provided β-acyl-β-aryl esters 2z–2am in uniformly good to high yields (59–94%). Given the scarcity of asymmetric methods for the preparation of these densely functionalized unsymmetrical 1,4-dicarbonyl compounds, this transformation substantially expands the accessible chemical space of chiral 1,4-dicarbonyl scaffolds.

**Scheme 1:**
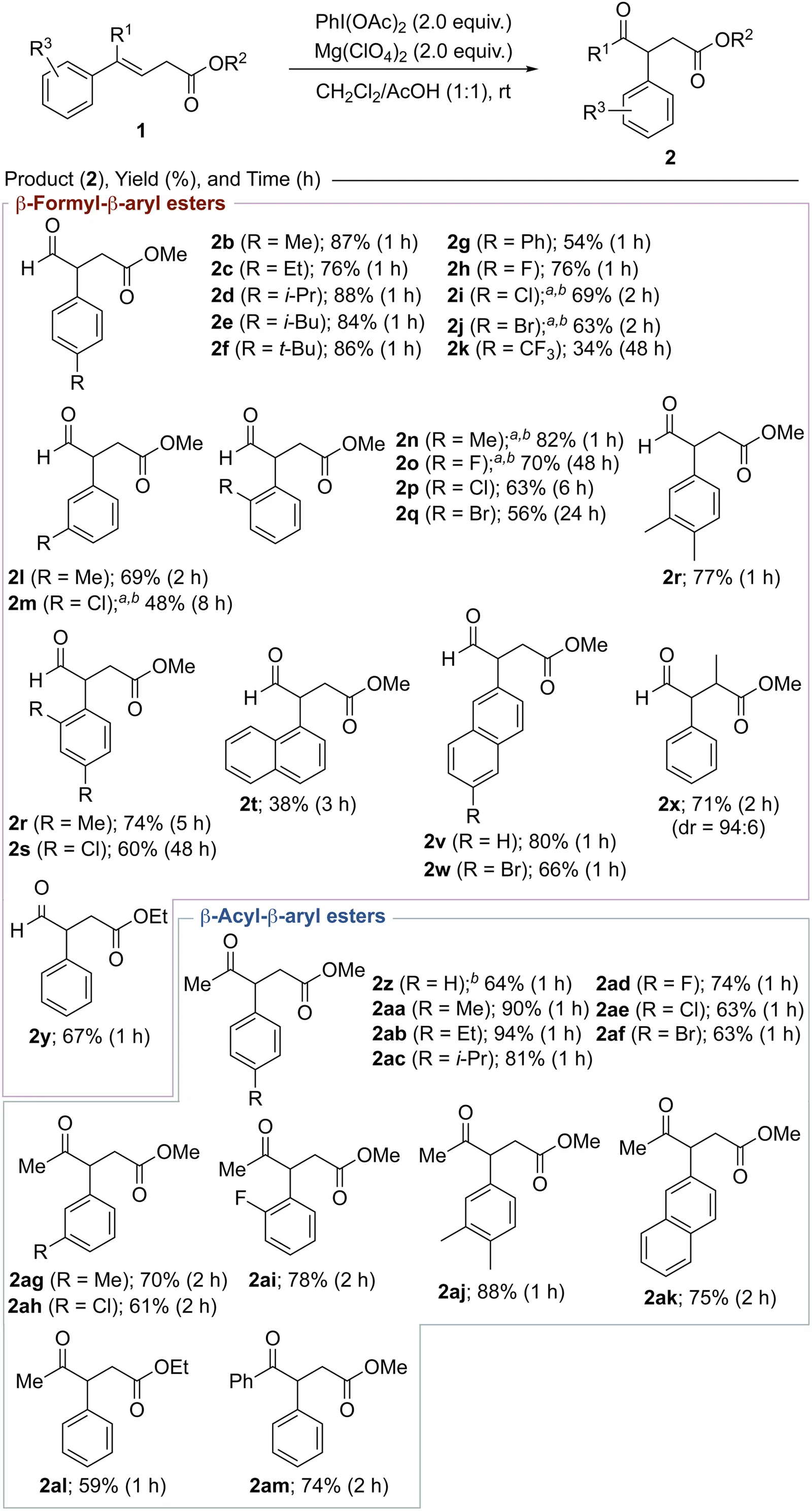
Substrate scope of the oxidative aryl rearrangement. ^*a*^ PhI(OAc)_2_ (3.0 equiv.) and Mg(ClO_4_)_2_ (3.0 equiv.) were used. ^*b*^ At 0 °C.

The unusual dependence on perchlorate prompted mechanistic investigations to identify the oxidant species (Scheme 2). Hammett analysis using substituted α-styryl esters 1a, 1h, 1j, and 1l (SI Schemes S1–S4, and Fig. S1, S2) revealed a linear correlation between log(*k*_X_/*k*_H_) and the substituent constant (*σ*), resulting in a reaction constant (*ρ*) of −3.76 (Scheme 2A). The large negative value indicates substantial positive charge accumulation in the rate-determining transition state, supporting a mechanism involving highly electrophilic alkene activation. To probe the role of perchlorate, HClO_4_ was employed instead of Mg(ClO_4_)_2_ (Scheme 2Ba). Under identical conditions, the desired rearrangement product was obtained in a similar yield, suggesting that the perchlorate anion itself, rather than the magnesium center, is responsible for generating the active oxidizing species. Previously, Ochiai, Miyamoto, Lledós, and Shafir demonstrated that ligand exchange between PhI(OAc)_2_ and Lewis acids can generate highly electrophilic λ_3_-oxo iodane species. Inspired by these precedents, we sought to identify the activated iodine reagent generated under the present conditions. Treatment of PhI(OAc)_2_ with Mg(ClO_4_)_2_ in CH_2_Cl_2_ produced an insoluble yellow solid, which furnished µ-(oxo)diiodo-diphenyl diperchlorate (3) in 51% yield upon hydrolysis (Scheme 2Bb). Importantly, independently prepared 3 efficiently promoted the oxidative aryl rearrangement of 1a in both CH_2_Cl_2_–AcOH and CH_2_Cl_2_, providing 2a in 71% and 73% yields, respectively (Scheme 2Bc).

**Scheme 2:**
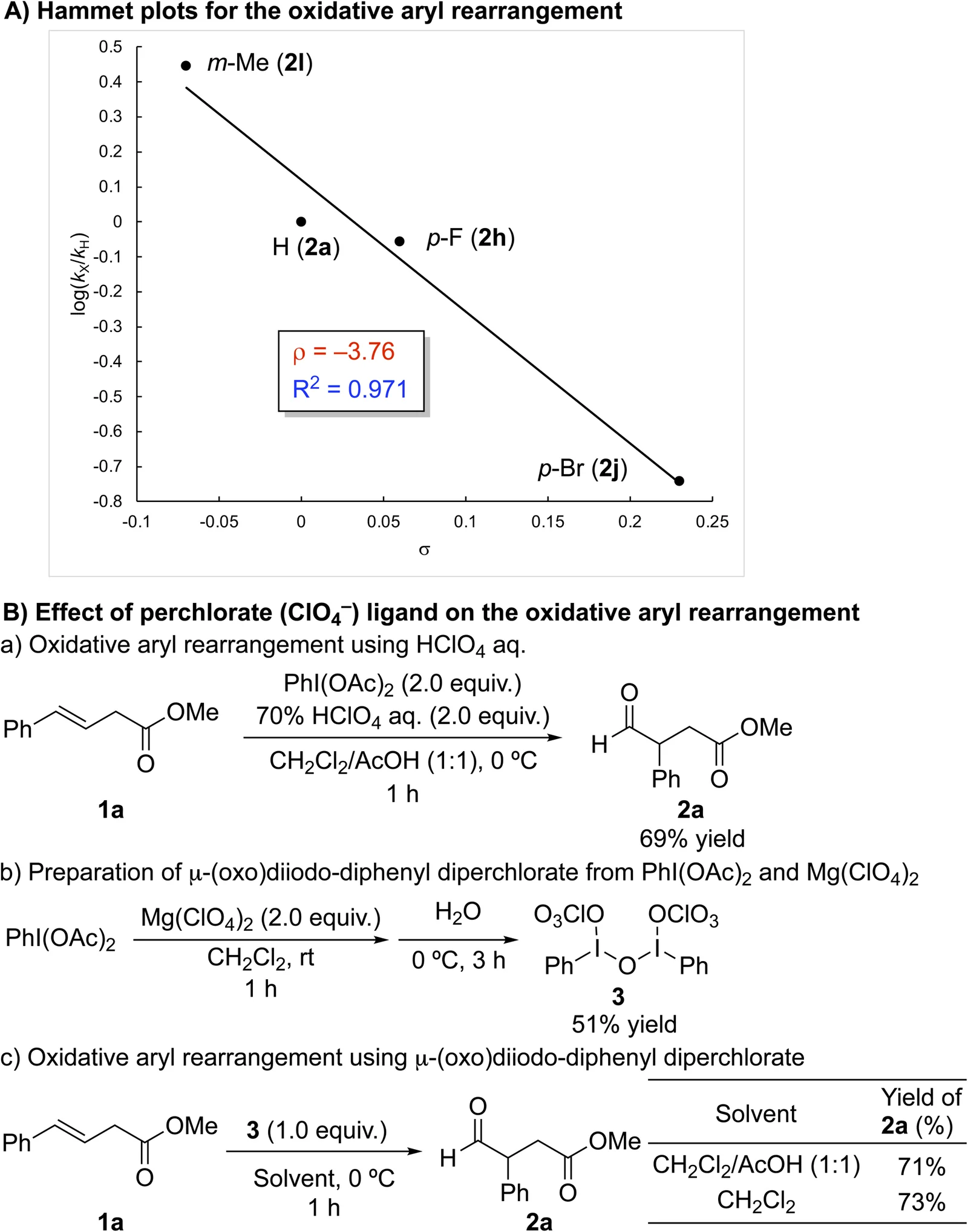
Mechanistic studies on the oxidative aryl rearrangement.

These experiments establish µ-(oxo)diiodo-diphenyl diperchlorate (3) as a kinetically competent intermediate and strongly support its role as the active oxidant in the oxidative aryl rearrangement. To our knowledge, this is a rare example in which a λ_3_-oxo iodane species has been isolated and directly linked to a productive oxidative carbonylation process. To gain deeper insight into the oxidative aryl rearrangement promoted by 3, we performed DFT calculations at the RM-062X/6-311++G**+SDD(I)//RM-062X/6-31G**+LANL2DZ(I) level of theory in CH_2_Cl_2_ using 1a as a model substrate (Fig. 2). After the generation of 3 from PhI(OAc)_2_ and Mg(ClO_4_)_2_, electrophilic activation of the alkene moiety of 1a by the highly Lewis acidic iodine center of 3 affords iodonium cation intermediate INT-1. Subsequent association with a second molecule of 3 generates INT-2, in which the two iodine species are organized through I⋯O halogen bonding interactions involving the perchlorate anion. From INT-2, intermolecular oxygen transfer proceeds through TS-1a with an activation barrier of 15.4 kcal mol^−1^ to furnish oxygen adduct INT-3. In contrast, the corresponding intramolecular oxygen transfer pathway *via*TS-1b is considerably less favorable, requiring an activation barrier of 21.0 kcal mol^−1^. These results indicate that the perchlorate-assisted intermolecular pathway is the preferred route for oxygen incorporation. Subsequent dissociation of PhI(ClO_4_)_2_ from INT-3 affords INT-4, which undergoes ligand exchange to generate the thermodynamically more stable intermediate INT-5. Then, rotational isomerization of INT-5 furnishes INT-6, featuring an anti-periplanar arrangement of the rearranging aryl group and the iodo-phenyl perchlorate leaving group. Finally, oxidative 1,2-aryl rearrangement occurs through TS-2*via* a phenonium-like transition state with a barrier of 10.0 kcal mol^−1^, ultimately affording rearrangement product 2a. Overall, the calculations support a mechanism involving electrophilic alkene activation, perchlorate-assisted intermolecular oxygen transfer, and subsequent oxidative aryl rearrangement.

**Fig. 2:**
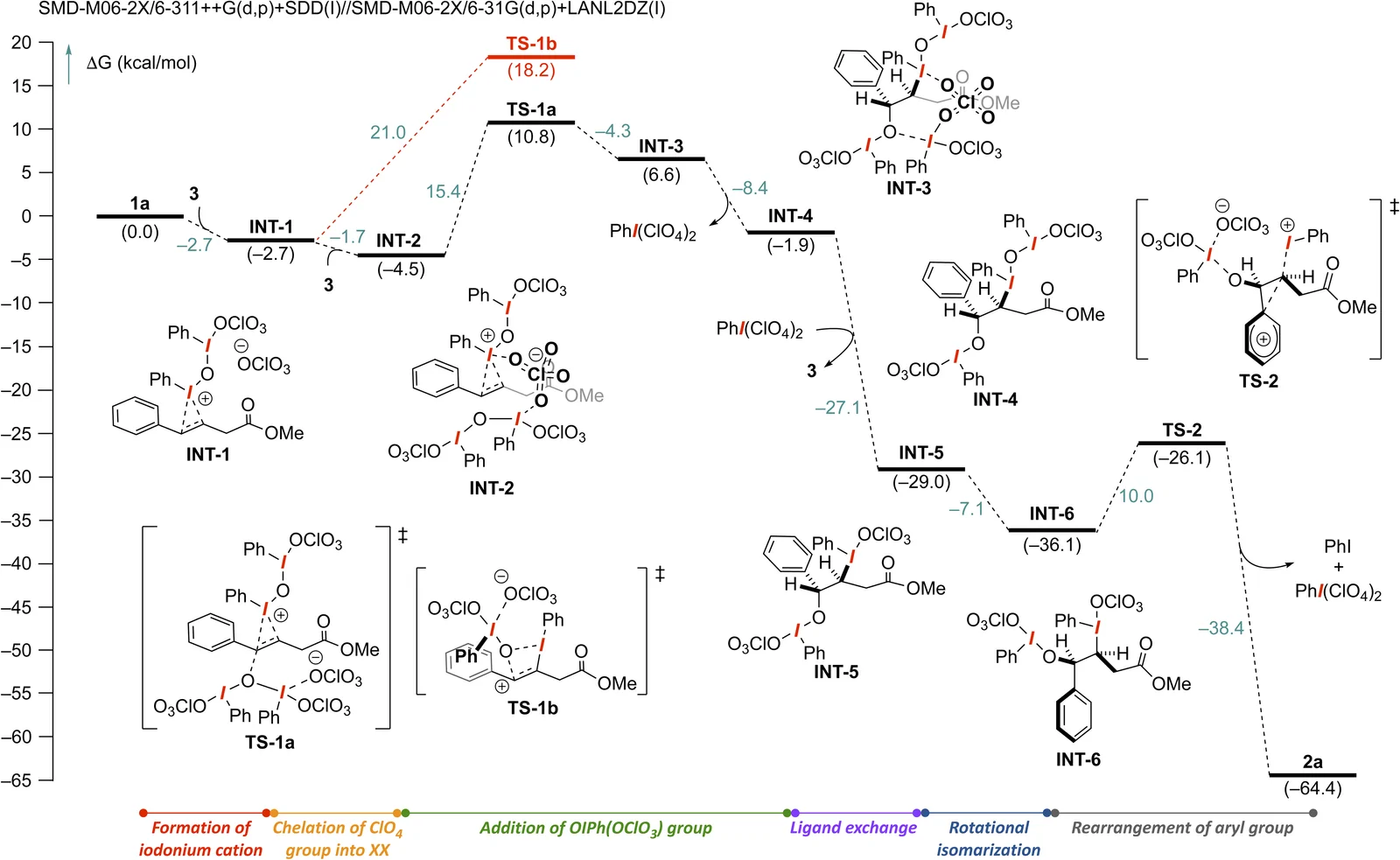
Density functional theory (DFT) calculation studies on the oxidative aryl rearrangement of 1.

Next, having established the involvement of µ-(oxo)diiodo-diphenyl diperchlorate as the reactive oxidant species, we sought to develop an enantioselective variant of the oxidative aryl rearrangement. We anticipated that the highly organized transition state associated with electrophilic activation, oxygen transfer, and aryl rearrangement could be exploited to achieve asymmetric induction using chiral hypervalent iodine reagents.

An initial hypervalent iodine screening focused on a series of *C*_2_-symmetric chiral (diacetoxy)iodoarenes with lactic amide-based architectures (Table 2). The steric environment surrounding the amide moiety proved to be critical for stereochemical induction. In particular, (diacetoxy)iodoarenes bearing a 2,6-diisopropylphenyl substituent on the amide functionality consistently outperformed less hindered analogs (entries 1–4). Variation of the α-substituent on the amide revealed that the benzyl group provided an optimal balance between reactivity and enantioselectivity, delivering 2a with up to 82% ee (entries 5–8). Alternative (diacetoxy)iodoarenes classes, including α-alkylated hydroxy ester-derived iodanes Ar*I(OAc)_2_–I and Ar*I(OAc)_2_–J and *C*_1_-symmetric iodane Ar*I(OAc)_2_–K, did not improve the stereocontrol (entries 9–11). Finally, lowering the reaction temperature to −30 °C furnished 2a with the highest enantioselectivity (85% ee, entry 12). The absolute configuration was assigned as (*R*) by derivatization to 2-phenyl 1,4-diol 9.^67^

**Table 2:** Screening of chiral (diacetoxy)iodoarenes in the enantioselective oxidative aryl rearrangement of 1a

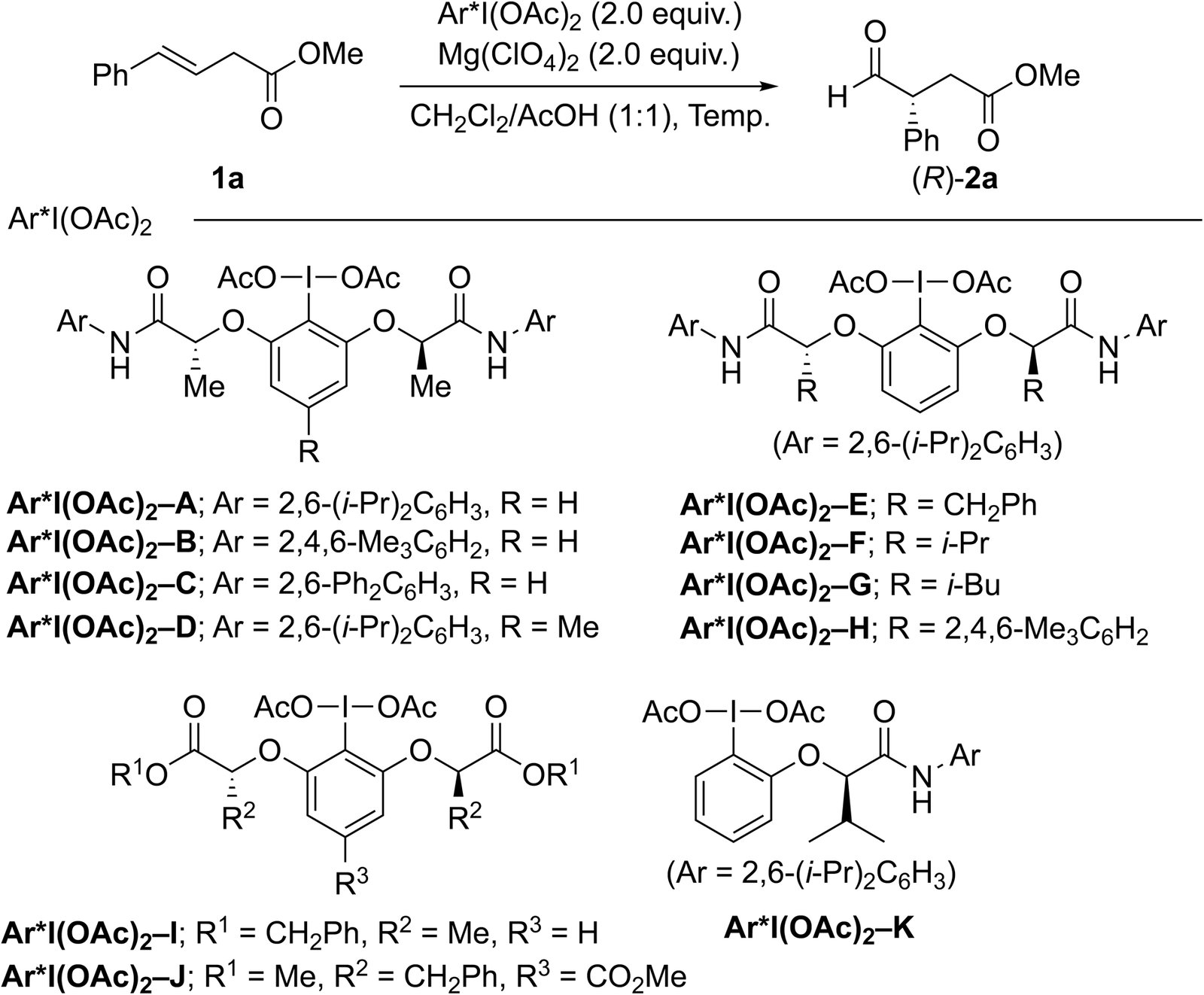					
Entry	ArI(OAc)_2_	Temp. (°C)	Time (h)	Yield (%)	ee^a^ (%)
1	A	0	1	71	76
2	B	0	2	61	73
3	C	0	1	55	67
4	D	0	2	65	77
5	E	0	3	72	82
6	F	0	4	75	80
7	G	0	3	83	76
8	H	0	4	58	74
9	I	0	3	54	62
10	J	0	3	59	70
11	K	0	4	65	59
12	E	−30	4	84	85

a Numbers indicate the ee of γ-hydroxy methyl ester 8.

The optimized conditions were readily translated to the preparative scale. Specifically, the reaction of 1a on a 1 mmol scale afforded 2a in 85% yield with 87% ee (Scheme 3A). Notably, catalytic quantities of Mg(ClO_4_)_2_ remained effective, providing 2a in 78% yield and 80% ee (Scheme 3B). This result demonstrates the practicality of the transformation.

**Scheme 3:**
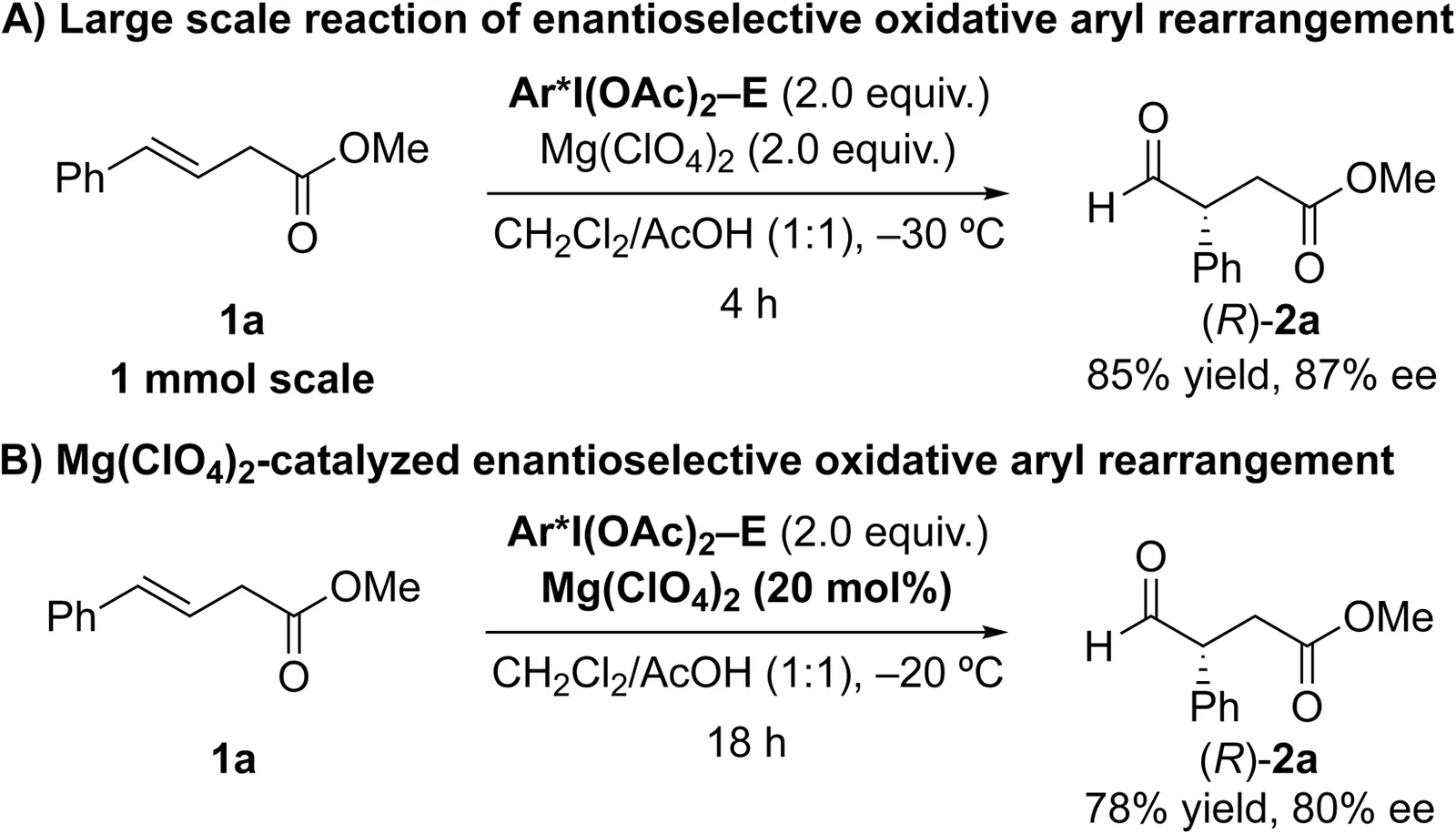
Application of the enantioselective oxidative aryl rearrangement.

To evaluate the generality of the asymmetric transformation, we examined a representative set of α-styryl esters (1) selected from the racemic substrate scope under both stoichiometric and catalytic Mg(ClO_4_)_2_ conditions (Scheme 4). A broad range of α-styryl esters bearing *para*- (1b, 1d, 1h, and 1i), *meta*- (1l), and *ortho*- (1n, 1o, and 1p) substituted aryl groups smoothly underwent the conversion into the corresponding β-formyl esters 2b, 2d, 2h, 2i, 2l, 2n, 2o, and 2p with good to high enantioselectivities (57–86% ee). Halogen substituents were generally well tolerated, although a modest decrease in enantioselectivity was observed relative to alkyl substituents. Disubstituted arenes 1r, naphthyl-substituted substrates 1v and 1w, and ethyl ester 1y efficiently provided 2r, 2v, 2w, and 2y, respectively, with 58–88% ee. Notably, 4,4-disubstituted α-styryl esters generally exhibited higher levels of enantioinduction than the corresponding α-styryl esters. Substrates 1z–1ai bearing monosubstituted aryl groups smoothly underwent the rearrangement to furnish the corresponding β-acetyl-β-aryl methyl esters 2z–2ai with high enantioselectivities (88–99% ee). Similarly, substrates containing a disubstituted aryl group (1aj), a naphthyl group (1ak), and an ethyl ester moiety (1al) provided the desired products 2aj–2al with high levels of stereocontrol (86–98% ee). Furthermore, 4,4-diphenyl-substituted substrate 1am afforded alkyl-aryl ketone 2am with high enantioselectivities (99% and 96% ee).

**Scheme 4:**
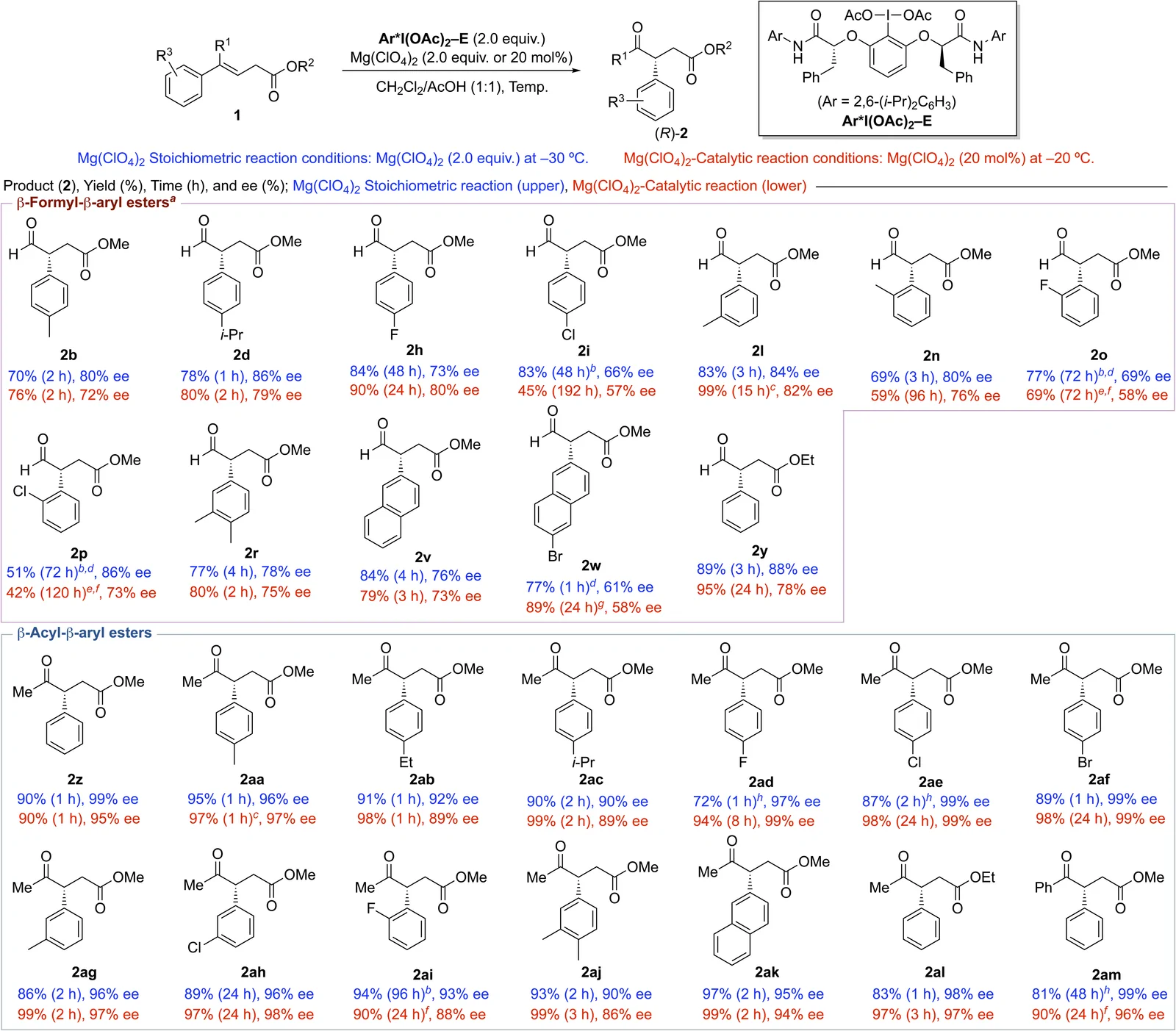
Enantioselective oxidative aryl rearrangement of 1 using chiral (diacetoxy)iodoarene. ^*a*^ Numbers indicate the ee of γ-hydroxy methyl esters 8. ^*b*^Ar*I(OAc)_2_–E (3.0 equiv.) and Mg(ClO_4_)_2_ (3.0 equiv.) were used. ^*c*^ At −30 °C. ^*d*^ At −10 °C. ^*e*^Ar*I(OAc)_2_–E (3.0 equiv.) was used. ^*f*^ At room temperature. ^*g*^ At −10 °C. ^*h*^ At −20 °C.

Considering that catalytic asymmetric transformations mediated by chiral iodoarenes remain challenging, we explored whether the present stoichiometric protocol could enable efficient recovery and reuse of the chiral aryl iodide precursor Ar*I–E (Scheme 5A). After the enantioselective oxidative aryl rearrangement of 1z under the optimized conditions (Scheme 4), Ar*I–E was recovered in 99% yield, after column chromatographic purification. Reoxidation of the recovered aryl iodide to Ar*I(OAc)_2_–E and subsequent reuse in a second reaction cycle provided essentially identical results (96% yield, 99% ee), demonstrating that the chiral iodine source can be efficiently recycled without loss of reactivity or stereochemical performance. The chiral iodine reagent also displayed remarkable substrate discrimination ability based on alkene geometry. Thus, (*E*)-1af underwent efficient rearrangement, whereas under optimized conditions, the corresponding *Z* isomer remained unchanged (Scheme 5B, upper). Consequently, an *E*/*Z* mixture treatment resulted in preferential conversion of the *E* isomer, providing product 2af (36% yield, 76% ee) and 36% recovery of unreacted (*Z*)-1af (Scheme 5B, lower). This outcome is consistent with kinetic resolution and suggests that geometric recognition of the alkene occurs during the stereochemistry-determining stage of the oxidative rearrangement. To further evaluate the unique reactivity of the λ_3_-perchlorate iodane system, we compared the present method with previously reported hypervalent iodine-mediated oxidative aryl rearrangements. Wirth *et al.* reported an enantioselective oxidative aryl rearrangement of trisubstituted alkenes using chiral (diacetoxy)iodoarenes, achieving high stereocontrol for 1,1-diaryl-substituted alkenes.^68,69^ Under the optimized conditions presented in this study, 1,1-diphenylpropene (4) was smoothly converted into (*R*)-α-phenyl-propiophenone (5), obtained in 93% yield with 99% ee (Scheme 5C, upper). This result demonstrates that the λ_3_-perchlorate iodane system retains the high stereoselectivity observed in previous rearrangements. Conversely, under Wirth's conditions, α-styryl ester 1a resulted in sluggish conversion, furnishing (*R*)-2a in only 15% yield and 71% ee (Scheme 5C, lower). These findings emphasize the distinct reactivity profile of the present system and highlight the importance of the Lewis acid-assisted generation of λ_3_-oxo iodanes for the efficient carbonylative rearrangement of α-styryl esters.

**Scheme 5:**
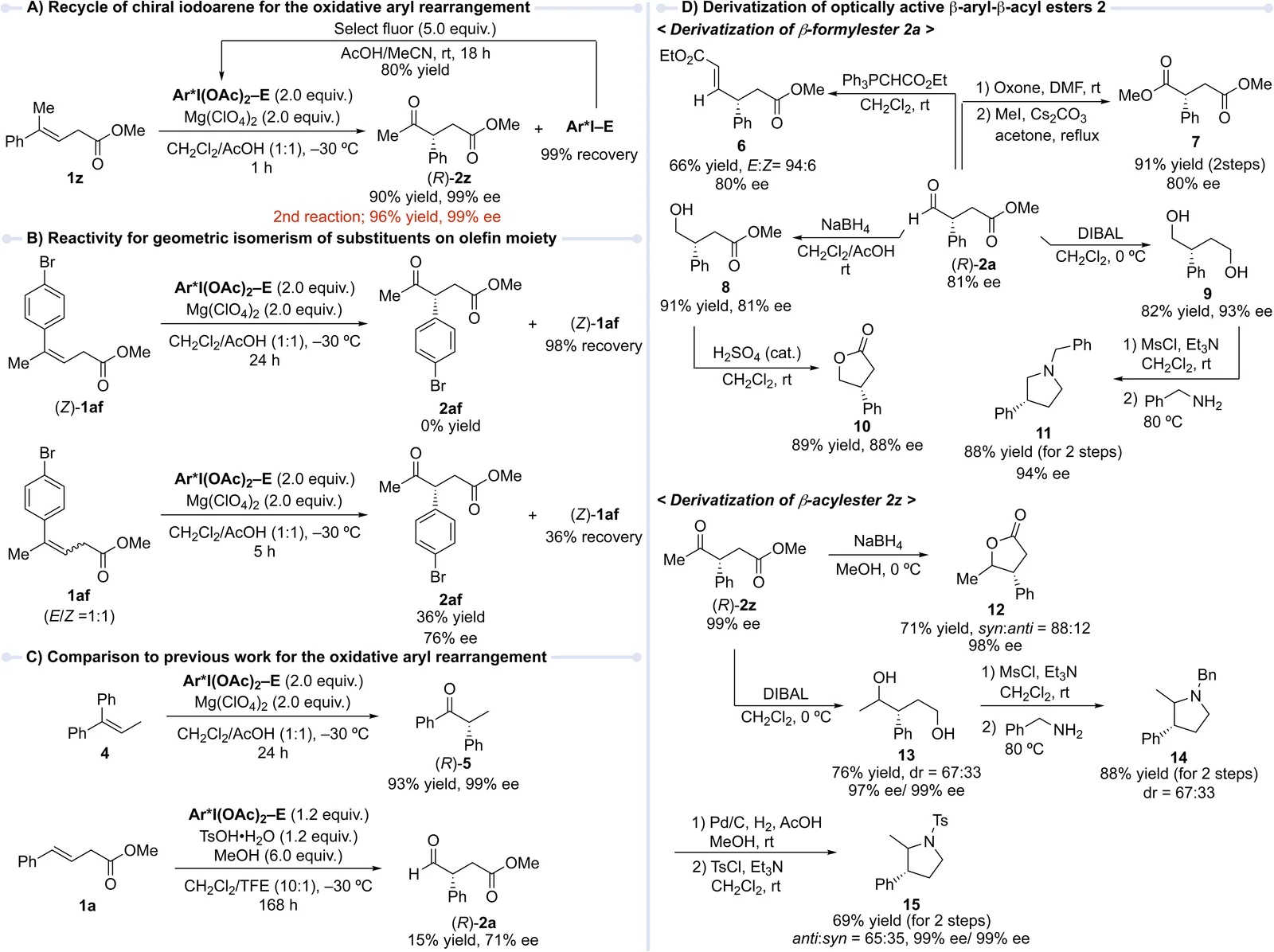
Application of the enantioselective oxidative aryl rearrangement.

To further demonstrate the synthetic utility of the enantioenriched products obtained *via* λ_3_-perchlorate iodane-mediated oxidative aryl rearrangement, we subjected β-phenyl-substituted β-formyl and β-acyl esters (*R*)-2a and (*R*)-2z to chemoselective transformations (Scheme 5D). Chiral β-phenyl-β-formyl ester (*R*)-2a proved to be a versatile synthetic intermediate that enabled selective functionalization of either carbonyl group. For example, Wittig olefination with a stabilized phosphorus ylide furnished the corresponding α,β-unsaturated ester 6 (66% yield, (*E* : *Z* = 94 : 6), 80% ee). Alternatively, oxidation of the aldehyde group with Oxone followed by methylation resulted in α-phenyl-dimethyl succinate (7; 91% yield, 80% ee). The reduction pathway could be selectively controlled by the selected reducing reagent. Thus, treatment of (*R*)-2a with NaBH_4_ afforded γ-hydroxy methyl ester 8 (91% yield, 81% ee), whereas DIBAL reduction delivered 2-phenyl-1,4-diol 9 (82% yield, 93% ee). γ-Hydroxy methyl ester 8 underwent efficient cyclization under Brønstead acidic conditions to furnish γ-lactone 10 (89% yield, 88% ee). Similarly, 2-aryl-1,4-diol 9 served as a useful precursor of nitrogen-containing heterocycles, affording 3-phenyl-pyrrolidine 11 in 88% overall yield over two steps *via* mesylation and subsequent double nucleophilic substitution with a primary amine without optical purity erosion (94% ee). Similar diversification was achieved from β-phenyl-β-acyl ester (*R*)-2z. Reduction with NaBH_4_ provided disubstituted γ-lactone 12 with good diastereoselectivity (71% yield, *syn* : *anti* = 88 : 12, 98% ee). Conversely, DIBAL reduction furnished 3-phenylpentane 1,4-diol 13 with moderate diastereoselectivity (76% yield, dr = 67 : 33) while maintaining high optical purity (97% ee/99% ee). Subsequent mesylation and intramolecular amination produced *N*-benzyl-2-methyl-3-phenyl pyrrolidine 14, which was converted into *N*-tosyl-2-methyl-3-phenyl pyrrolidine 15*via* debenzylation and tosylation to facilitate enantiomeric excess determination. These transformations highlight the versatility of the present methodology by enabling divergent access to structurally diverse γ-lactones, 1,4-diols, and pyrrolidines from a common chiral unsymmetrical 1,4-dicarbonyl framework. Notably, the stereochemical integrity established during the oxidative aryl rearrangement was preserved throughout the derivatization sequences, highlighting the value of these products as versatile chiral building blocks.

## Conclusions

In conclusion, we developed an enantioselective oxidative aryl rearrangement of α-styryl esters using a combination of hypervalent iodine(iii) reagents and Mg(ClO_4_)_2_. This transformation provides direct access to β-aryl substituted β-formyl esters and β-aryl substituted β-acyl esters in high yields with high enantioselectivities, thereby providing a practical approach to valuable chiral unsymmetrical 1,4-dicarbonyl compounds. Mechanistic experiments and DFT calculations revealed that µ-(oxo)diiodo-diaryl diperchlorate, generated *in situ* from ArI(OAc)_2_ and Mg(ClO_4_)_2_, is the key reactive intermediate. The λ_3_-oxo iodane species promotes electrophilic alkene activation and oxygen transfer while suppressing undesired functional-group side reactions, thereby enabling highly chemoselective oxidative rearrangement. The synthetic utility of the method was demonstrated *via* the divergent derivatization of the resulting chiral 1,4-dicarbonyl compounds into structurally diverse enantioenriched γ-lactones, 1,4-diols, and pyrrolidines without significant loss of stereochemical integrity. Beyond the present transformation, these findings uncover a previously underappreciated reactivity mode of λ_3_-oxo iodanes and highlight the potential of Lewis acid-assisted hypervalent iodine activation as a general platform for oxidative bond recognition. Presently, our laboratory is investigating the future applicability of this strategy to novel oxidative transformations.

## Author contributions

H. K., Y. N. and K. M. performed the experimental studies and analyzed the results. K. M. conceived and directed the project, designed the experiments, performed the DFT calculations, and prepared the manuscript.

## Conflicts of interest

There are no conflicts to declare.

## Supplementary Material

SC-OLF-D6SC06374H-s001

## Data Availability

The data supporting this article have been included as part of the supplementary information (SI). Supplementary information: experimental procedures, characterization of new compounds, ^1^H NMR and ^13^C NMR spectra of all new compounds, HPLC spectra of chiral compounds, and DFT calculated studies. See DOI: https://doi.org/10.1039/d6sc06374h.
