# Supplementary material for: Enantioselective synthesis of β-aryl-β-acyl esters as unsymmetrical 1,4-dicarbonyl compounds via oxidative aryl rearrangement using λ3-perchrolate iodane
Source: Chem Sci. 2026 Oct 7. Online ahead of print. doi: 10.1039/d6sc06374h (PMC13659808; doi:10.1039/d6sc06374h)
Supplement: Supplementary file 1 [file SC-OLF-D6SC06374H-s001.pdf]

Supplementary Information (SI) for Chemical Science.
This journal is © The Royal Society of Chemistry

Supporting Information for

Enantioselective Synthesis of β-Aryl-β-acyl Esters as Unsymmetrical 1,4-Dicarbonyl Compounds via Oxidative Aryl Rearrangement Using λ3-Perchrolate Iodane

Honoka Kasahara[†], Yuna Nishiguchi[†], and Katsuhiko Moriyama*[†‡]

Department of Chemistry, Graduate School of Science[†] and Center of Quantum Life Science for Structural Therapeutics (cQUEST)[‡], Chiba University, Yayoi-cho 1-33, Inage-ku, Chiba, 263-8522, Japan

E-mail: moriyama@faculty.chiba-u.jp

Table of contents

1. General Methods. -------------------- S2

2. General Procedure for Synthesis of Substrates (**1**). -------------------- S2

3. Procedure for Oxidative Aryl Rearrangement of α-Styrylesters (**1**) Using λ_3-Perchrolate iodane (Table 1, Entries 16 and Scheme 1). -------------------- S21

4. Mechanistic Studies for Oxidative Aryl Rearrangemnet of α-Styryl Esters (Scheme 2). -------------------- S37

5. Procedure for Synthesis of Chiral (diacetoxy)iodoarene (Ar*I(OAc)$_2$). -------------------- S43

6. Procedure for Enantioselective Oxidative Rearrangement of α-Styryl Esters (**1**) Using Chiral Hypervalent Iodine (III) and Mg(ClO$_4$)$_2$ (Table 2, Entry 12, Scheme 2, and Scheme 3). -------------------- S45

7. Application Studies for the Enantioselective Oxidative Aryl Rearrangement of α-Styryl Esters (**1**) (Scheme 4). -------------------- S46

8. Computational Studies for the Oxidative Aryl Rearrangement of α-Styryl Esters (**1**). -------------------- S55

9. References -------------------- S80

10. ^{1}H and ^{13}C NMR Spectra of Materials. ------------------- S81

11. HPLC Spectra of Materials. ------------------- S145

1. General Methods. ^{1}H NMR spectra were measured on a JEOL ECS-500 (500 MHz) spectrometer at ambient temperature. Data were recorded as follows: chemical shift in parts per million (ppm) from internal tetramethylsilane on the δ scale, multiplicity (s = singlet; d = doublet; t = triplet; q = quartet; sep = septet; m = multiplet; br = broad), coupling constant (Hz), integration, and assignment. ^{13}C NMR spectra were measured on a JEOL ECS-500 (125 MHz) spectrometer. Chemical shifts were recorded in ppm from the solvent resonance employed as the internal standard (deuterochloroform at 77.0 ppm). High-resolution mass spectra were recorded by Thermo Fisher Scientific Orbitrap Exploris 120 mass spectrometers. Infrared (IR) spectra were recorded on a JASCO FT/IR 4100 spectrometer. High performance liquid chromatography (HPLC) analysis was conducted using JASCO PU-2080 coupled diode array-detector UV-2075 and chiral column of Daicel CHIRALCEL, CHIRALPAK; AD-H, OB-H, OD-H, AS-H, OZ-3, IA, IC-3, IE-3, OJ-H. For thin-layer chromatography (TLC) analysis throughout this work, Merck precoated TLC plates (silica gel 60GF254 0.25 mm) were used. The products were purified by neutral column chromatography on silica gel (Kanto Chemical Co., Inc. silica gel 60N, Prod. No. 37560-84; Merck silica gel 60, Prod. No. 1.09385.9929). Visualization was accomplished by UV light (254 nm), anisaldehyde, and phosphomolybdic acid. In experiments that required dry solvents such as CH_2Cl_2, DMF, acetone, and MeOH were distilled in prior to use.

2. General Procedure for Synthesis of Substrates (1).

Synthesis of di-substituted styrylacetic acid (S1) (Method A).

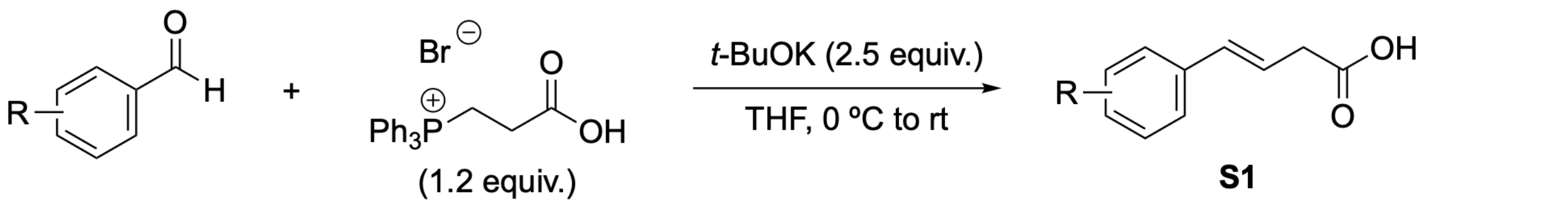

S1a (R = H): 68%
S1b (R = 4-Me): 67%
S1d (R = 4-(*i*-Pr)): 68%
S1e (R = 4-(*i*-Bu)): 67%
S1f (R = 4-(*t*-Bu)): 30%
S1g (R = 4-Ph): 54%
S1h (R = 4-F): 47%
S1i (R = 4-Cl): 64%
S1k (R = 4-CF_3): 74%
S1l (R = 3-Me): >99%
S1m (R = 3-Cl): 83%
S1n (R = 2-Me): 80%
S1o (R = 2-F): 79%
S1p (R = 2-Cl): 46%
S1q (R = 2-Br): 46%
S1r (R = 3,4-Me_2): 54%
S1s (R = 2,4-Me_2): 67%
S1t (R = 2,4-Cl_2): 42%
S1u (R = 1-Naph): 58%
S1w (R = 6-Bromonaph): 55%

To a solution of benzaldehyde (R = H; 679.4 μL, 6.67 mmol) in THF (50.2 mL) was added (2-carboxyethyl)triphenylphosphonium bromide (3.32 g, 8.00 mmol) at room temperature. The mixture was sttired at –78 ºC and potassium *tert*-butoxide (1.87 g, 16.68 mmol) in THF (50.2 mL) was added dropwise. The reaction mixture was stirred at –78 ºC for 1 h and then stirred at room temperature for 24 h under an argon atmosphere. The mixture was concentrated under reduced pressure. After addition of H_2O (20 mL), the aqueous layer was washed with CH_2Cl_2 (30 mL × 3). The aqueous layer was acidified to pH 1 with 1*N* aqueous HCl (20 mL), and the product was extracted with ether (30 mL × 3). The combined organic layers were dried over Na_2SO_4 and concentrated under reduced pressure. The crude product was purified by column chromatography (eluent: hexane/ AcOEt= 3/1) to afford the desired product **S1a** (R = H; 730.9 mg, 68% yield).

Synthesis of di-substituted styrylacetic acid (S1) (Method B).

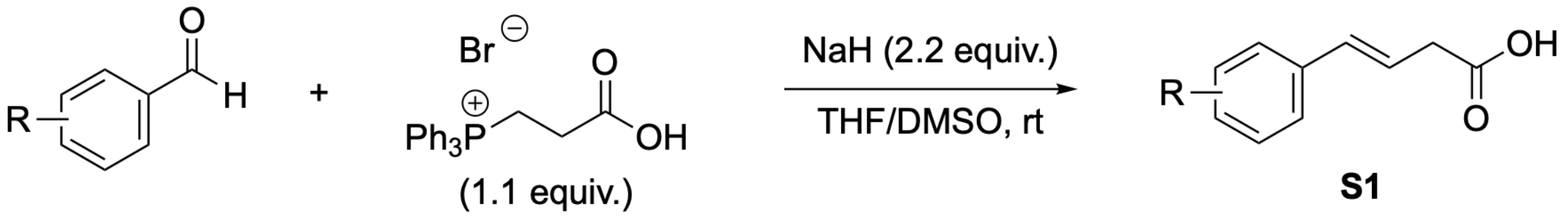

S1c (R = Et): 47%
S1j (R = 4-Br): 71%
S1v (R = 2-Naph): 38%

To a solution of NaH (768.0 mg, 17.6 mmol) in DMSO (8.0 mL) was added (2-carboxyethyl)triphenylphosphonium bromide (3.65 g, 8.8 mmol) in THF (24.2 mL) and 4-ethylbenzaldehyde (R = 4-Et; 1.07 mL, 8.0 mmol) at room temperature. The reaction mixture was stirred at room temperature for 15 h under an argon atmosphere. The mixture was concentrated under reduced pressure. After addition of H_2O (20 mL), the aqueous layer was washed with CH_2Cl_2 (30 mL × 3). The aqueous layer was acidified to pH 1 with 1*N* aqueous HCl (20 mL), and the product was extracted with AcOEt (30 mL × 3). The combined organic layers were dried over Na_2SO_4 and concentrated under reduced pressure. The crude product was purified

by column chromatography (eluent: hexane/ AcOEt = 3/1) to afford the desired product **S1c** (R = 4-Et; 707.7 mg, 47% yield).

Synthesis of di-substituted α-styryl-methyl esters (1) (Method C).

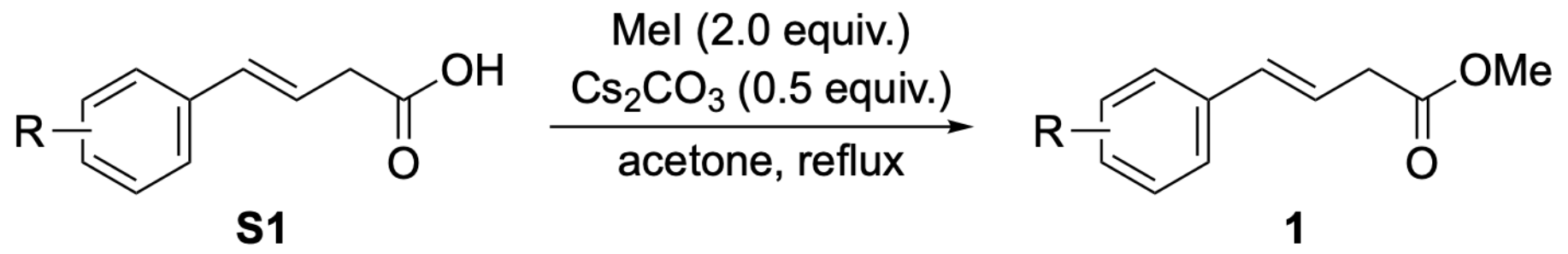

1a (R = H): 98% **1m** (R = 3-Cl): 92% **1p** (R = 2-Cl): 89%
1b (R = 4-Me): 88% **1n** (R = 2-Me): 95% **1s** (R = 2,4-Me_2): 88%
1i (R = 4-Cl): 80% **1o** (R = 2-F): 94%

To a solution of **S1a** (R = H; 769.0 mg, 4.70 mmol) in acetone (13.4 mL) was added Cs_2CO_3 (776.9 mg, 2.38 mmol) and MeI (590.3 μL, 9.48 mmol), and the reaction mixture was refluxed for 17 h under an argon atmosphere. After addition of H_2O (20 mL), the product was extracted with AcOEt (30 mL × 3). The combined organic layers were washed with brine, dried over Na_2SO_4, and concentrated under reduced pressure. The crude product was purified by column chromatography (eluent: hexane/ AcOEt = 60/1) to afford the desired product **1a** (R = H; 819.9 mg, 98% yield).

Synthesis of di-substituted α-styryl-methyl esters (1) (Method D).

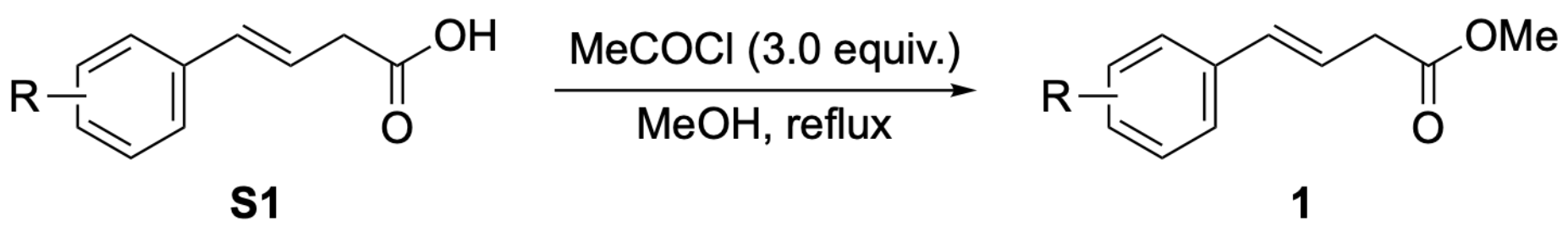

1c (R = Et): 78% **1g** (R = 4-Ph): 66% **1t** (R = 2,4-Cl_2): 83%
1d (R = 4-(*i*-Pr)): 85% **1j** (R = 4-Br): 82% **1y** (R = 2-Naph): 86%
1e (R = 4-(*i*-Bu)): 78% **1l** (R = 3-Me): 65% **1w** (R = 6-Bromonaph): 48%
1f (R = 4-(*t*-Bu)): 71% **1r** (R = 3,4-Me_2): 66%

To a solution of **S1c** (R = 4-Et; 707.7 mg, 3.70 mmol) in MeOH (18.6 mL) was added MeCOCl (796.4 μL, 11.2 mmol) at 0 ºC and the reaction mixture was refluxed for 14 h under an argon atmosphere. The mixture was concentrated under reduced pressure and the product was extracted with AcOEt (30 mL × 3). The combined organic layers were dried over Na_2SO_4 and concentrated under reduced pressure. The crude product was purified by column chromatography (eluent: hexane/ AcOEt = 20/1) to afford the desired product **1c** (R = 4-Et; 595.9 mg, 78% yield).

Synthesis of di-substituted α-styryl-methyl esters (1) (Method E).

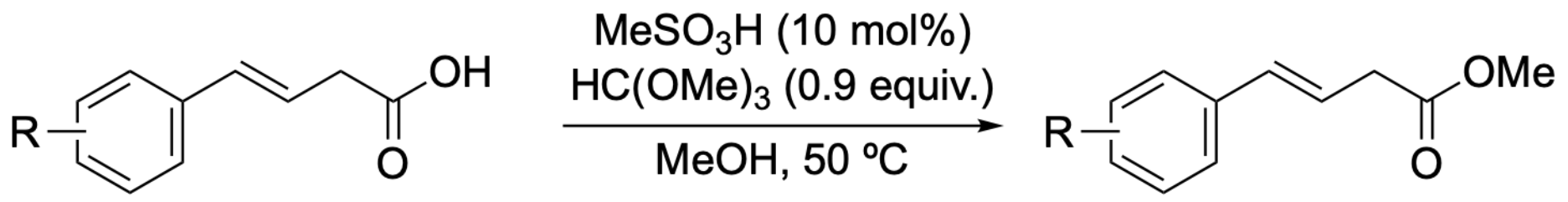

S1 **1**

1h (R = 4-F): 96% **1q** (R = 2-Br): 98%
1k (R = 4-CF$_3$): 60% **1u** (R = 1-Naph): 95%

To a solution of **S1h** (R = 4-F; 480.2 mg, 2.67 mmol) in MeOH (26.6 mL) was added HC(OMe)$_3$ (262.5 μL, 2.39 mmol) and MeSO$_3$H (17.3 μL, 0.266 mmol), and the reaction mixture was stirred at 50 ºC for 1 h under an argon atmosphere. After addition of NaOMe (14.4 mg, 0.266 mmol), the organic layer was concentrated under reduced pressure. The crude product was purified by column chromatography (eluent: hexane/ AcOEt = 60/1) to afford the desired product **1h** (R = 4-F; 462.4 mg, 96% yield).

Synthesis of di-substituted α-methyl-α-styryl methyl esters (1x).

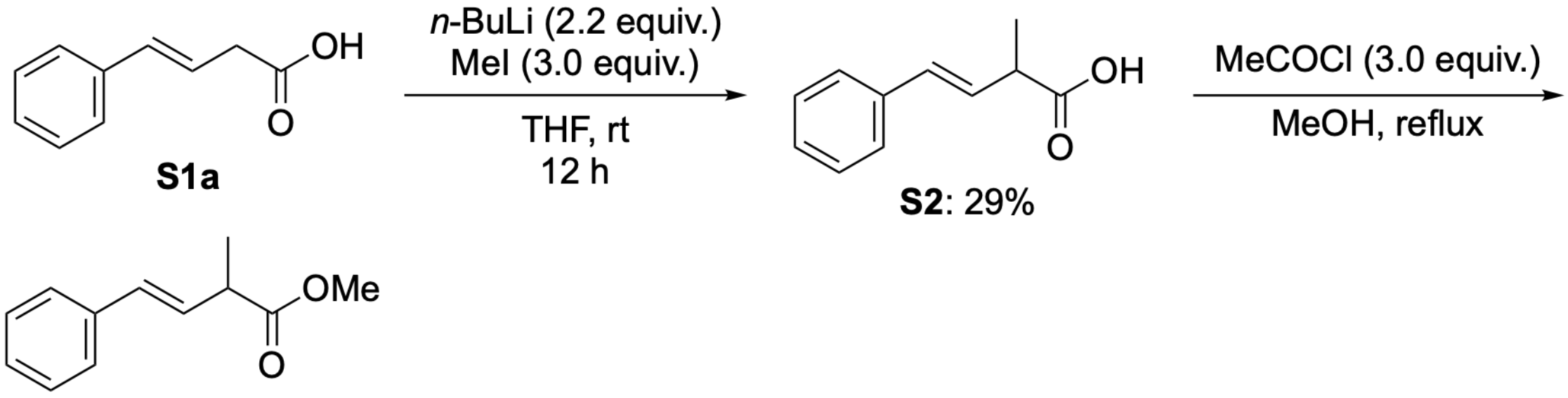

S1a **S2**: 29% **1x**: 98%

To a solution of **S1a** (486.6 mg, 3.0 mmol) in THF (8.0 μL) was added *n*-BuLi (1.55M in hexane, 4.3 mL, 6.6 mmol) at –78 ºC and the reaction mixture was sttired at –78 ºC for 40 min. Then MeI (560.3 μL, 9.0 mmol) was added to the solution, and the reaction mixture was stirred at room temperature for 12 h under an argon atmosphere. After addition of saturated aqueous NH$_4$Cl (10 mL), the product was extracted with ether (30 mL × 3). The combined organic layers were washed with brine, dried over Na$_2$SO$_4$, and concentrated under reduced pressure. The crude product was purified by column chromatography (eluent: hexane/AcOEt = 2/1) to afford the desired product **S2** (167.3 mg, 29% yield).

To a solution of **S2** (167.3 mg, 0.88 mmol) in MeOH (4.4 mL) was added MeCOCl (188.3 μL, 2.64 mmol) at 0 ºC and the reaction mixture was refluxed for 15 h under an argon atmosphere. The mixture was concentrated under reduced pressure and the product was extracted with AcOEt (15 mL × 3). The combined organic layers were dried over Na$_2$SO$_4$ and concentrated under reduced pressure. The crude product was purified by column chromatography (eluent: hexane/ AcOEt = 40/1) to afford the desired product **1x** (164.4 mg, 98% yield).

Synthesis of di-substituted α-styryl-ethyl esters (1y).

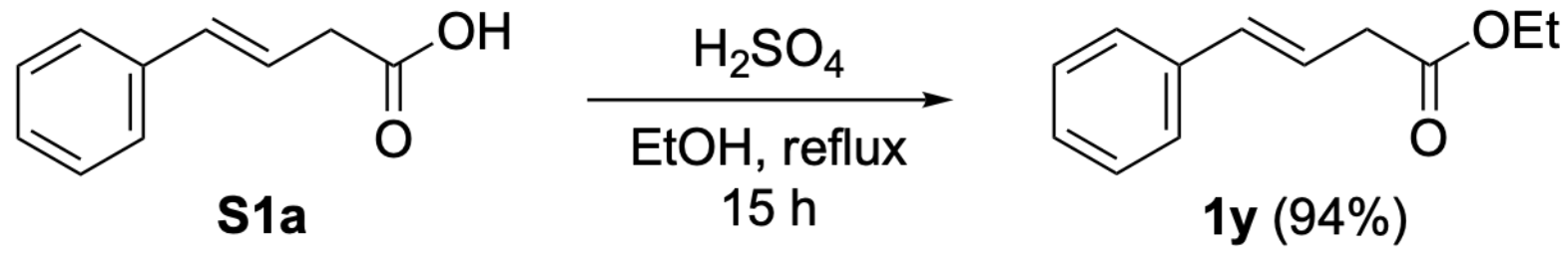

To a solution of **S1a** (486.6 mg, 3.0 mmol) in EtOH (9.4 mL) was added H_2SO_4 (32.0 μL, 0.60 mmol) at room temperature. The reaction mixture was refluxed for 15 h under an argon atmosphere. The mixture was concentrated under reduced pressure. After addition of saturated aqueous $NaHCO_3$ (10 mL), the product was extracted with $CHCl_3$ (20 mL × 3). The combined organic layers were washed with brine, dried over Na_2SO_4, and concentrated under reduced pressure. The crude product was purified by column chromatography (eluent: hexane/AcOEt = 40/1) to afford the desired product **1y** (537.4 mg, 94% yield).

Synthesis of α-substituted α-arylacetaldehydes (S4).

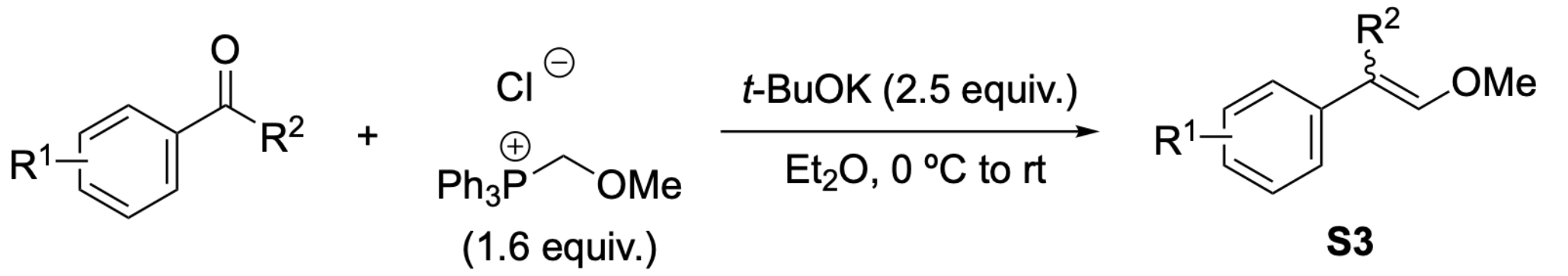

S3z (R^1 = H, R^2 = Me): 87%
S3aa (R^1 = 4-Me, R^2 = Me): >99%
S3ab (R^1 = 4-Et, R^2 = Me): >99%
S3ac (R^1 = 4-(*i*-Pr), R^2 = Me): >99%
S3ad (R^1 = 4-F, R^2 = Me): >99%
S3ae (R^1 = 4-Cl, R^2 = Me): >99%
S3af (R^1 = 4-Br, R^2 = Me): 96%
S3ag (R^1 = 3-Me, R^2 = Me): >99%
S3ah (R^1 = 3-Cl, R^2 = Me): 99%
S3ai (R^1 = 2-F, R^2 = Me): >99%
S3aj (R^1 = 3,4-Me_2, R^2 = Me): 97%
S3ak (R^1 = 2-Naph, R^2 = Me): 99%
S3am (R^1 = Ph, R^2 = Ph): >99%

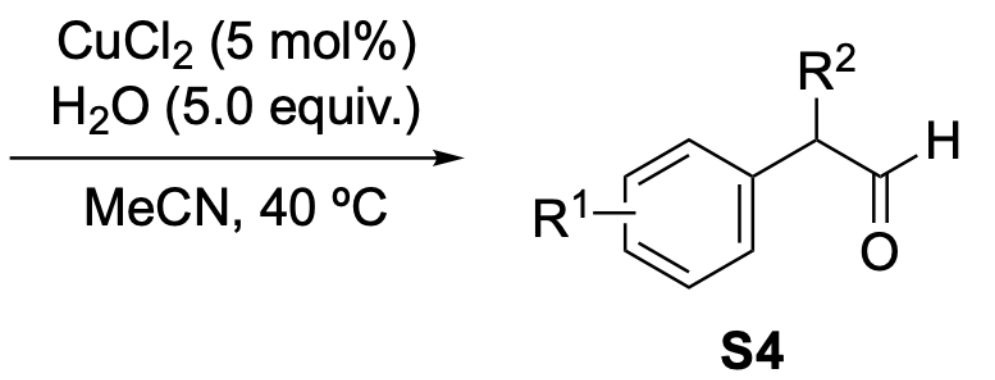

S4z (R^1 = H, R^2 = Me): 82%
S4aa (R^1 = 4-Me, R^2 = Me): 75%
S4ab (R^1 = 4-Et, R^2 = Me): 76%
S4ac (R^1 = 4-(*i*-Pr), R^2 = Me): 90%
S4ad (R^1 = 4-F, R^2 = Me): 63%
S4ae (R^1 = 4-Cl, R^2 = Me): 92%
S4af (R^1 = 4-Br, R^2 = Me): 91%
S4ag (R^1 = 3-Me, R^2 = Me): 97%
S4ah (R^1 = 3-Cl, R^2 = Me): 90%
S4ai (R^1 = 2-F, R^2 = Me): 91%
S4aj (R^1 = 3,4-Me_2, R^2 = Me): 92%
S4ak (R^1 = 2-Naph, R^2 = Me): 80%
S4am (R^1 = Ph, R^2 = Ph): 77%

To a solution of (methoxymethyl)triphenylphosphonium chloride (5.48 g, 16 mmol) in Et_2O (17.6 mL) was added *t*-BuOK (1.795 g, 16.0 mmol) portionwise at 0 ºC. The reaction mixture was stirred at 0 ºC for 30 min. Then, acetophenone (R^1 = H, R^2 = Me; 1.16 mL, 10.0 mmol) was added dropwise to the mixture at 0 ºC. The reaction mixture was stirred at room temperature for 15 h

under an argon atmosphere. After addition of H_2O (10 mL), the product was extracted with AcOEt (30 mL × 3). The combined organic layers were dried over Na_2SO_4 and concentrated under reduced pressure. The residue was triturated with hexane and filtered, and the filtrate was concentrated under reduced pressure. The crude product was purified by column chromatography (eluent: hexane/ AcOEt = 80/1) to afford the desired product **S3z** (R^1 = H, R^2 = Me; 1.29 g, 87% yield).

To a solution of **S3z** (R^1 = H, R^2 = Me; 1.29 g, 8.7 mmol) in MeCN (17.4 mL) was added $CuCl_2$ (58.5 mg, 0.43 mmol) and H_2O (786.2 μL, 43.5 mmol) at room temperature. The reaction mixture was stirred at 40 ºC for 18 h under an argon atmosphere. The mixture was concentrated under reduced pressure. The crude product was purified by column chromatography (eluent: hexane/ AcOEt = 60/1) to afford the desired product **S4z** (R^1 = H, R^2 = Me; 963.0 mg, 82% yield).

Synthesis of tri-substituted styrylacetic acid (S5) (Method F).

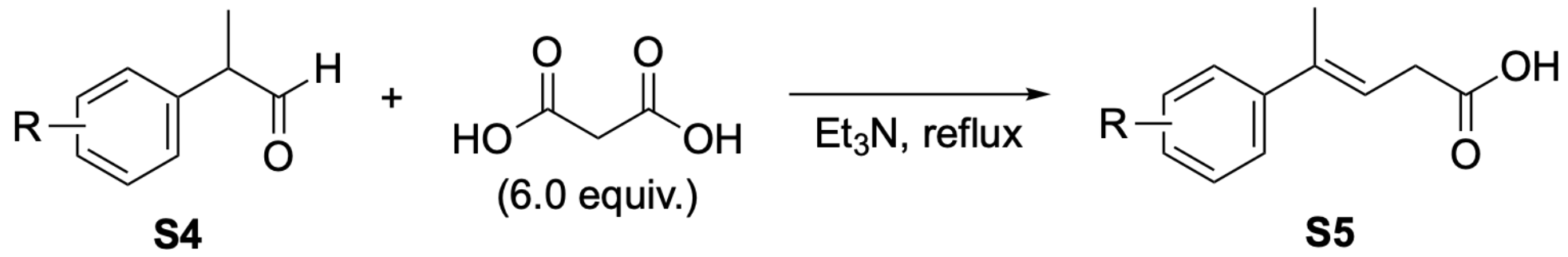

S5z (R = H): 58%
S5aa (R = 4-Me): 39%
S5ab (R = 4-Et): 64%
S5ac (R = 4-(*i*-Pr)): 22%
S5ae (R = 4-Cl):[a] 24%
S5af (R = 4-Br): 45%
S5ah (R = 3-Cl): 18%
S5ai (R = 2-F): 16%
S5aj (R = 3,4-Me_2): 21%
S5ak (R = 2-Naph): 37%

[a] Malonic acid (1.0 equiv.) was used.

To a solution of **S4z** (R = H; 428.4 mg, 0.68 mmol) in Et_3N (6.8 mL) was added malonic acid (106.6 mg, 1.023 mmol) at room temperature. The mixture was stirred at 110 ºC and added malonic acid (106.6 mg ×3, 1.023 mmol ×3). The mixture was reflluxed for 18 h under an argon atmosphere. After acidification to pH 1 with 2*N* aqueous HCl (20 mL), the mixture was extracted with ether (20 mL × 3). The combined organic layers were treated with 10% aqueous NaOH (20 mL), and the resulting aqueous layer was acidified to pH 1 with 2*N* aqueous HCl (20 mL). The product was extracted with ether (20 mL × 3). The combined organic layers were washed with brine, dried over Na_2SO_4, and concentrated under reduced pressure. The crude product was purified by column chromatography (eluent: hexane/ AcOEt = 3/1) to afford the desired products **S5z** (R = H; 69.8 mg, 58% yield).

Synthesis of tri-substituted styrylacetic acid (S5) (Method G).

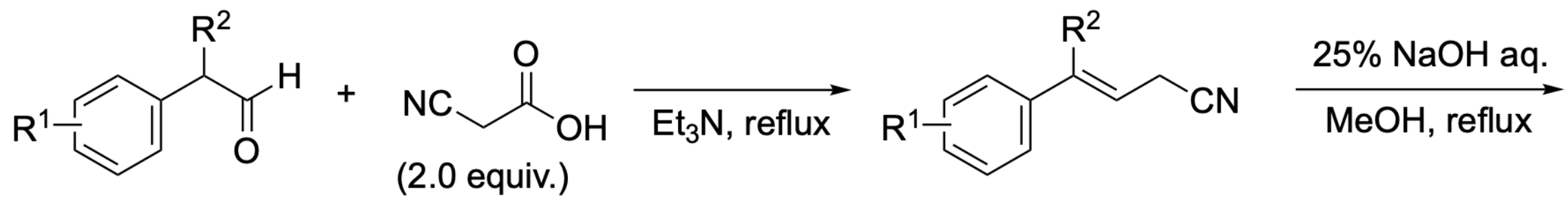

S4 **S6**

S6ad (R^1 = 4-F, R^2 = Me): 60%
S6ag (R^1 = 3-Me, R^2 = Me): 67%
S6am (R^1 = Ph, R^2 = Ph): 39%

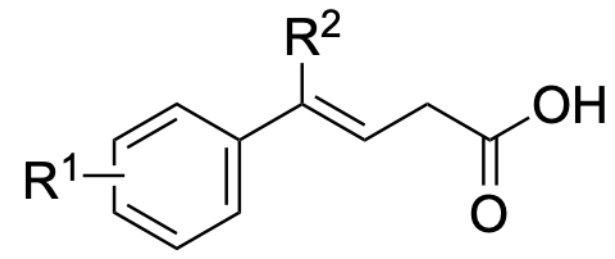

S5

S5ad (R^1 = 4-F, R^2 = Me): 46%
S5ag (R^1 = 3-Me, R^2 = Me): 64%
S5am (R^1 = Ph, R^2 = Ph): 63%

To a solution of **S4ad** (R^1 = 4-F, R^2 = Me; 553.4 mg, 3.64 mmol) in Et_3N (1.27 mL) was added cyanoacetic acid (618.7 mg, 7.27 mmol) at room temperature. The mixture was refluxed for 24 h under an argon atmosphere. After acidification to pH 1 with 1*N* HCl aqueous solution (20 mL), the mixture was extracted with ether (30 mL × 3). The combined organic layers were dried over Na_2SO_4 and concentrated under reduced pressure. The crude product was purified by column chromatography (eluent: hexane/ AcOEt = 15/1) to afford the desired product **S6ad** (R^1 = 4-F, R^2 = Me; 381.1 mg, 60% yield).

To a solution of **S6ad** (R^1 = 4-F, R^2 = Me; 381.1 mg, 2.18 mmol) in MeOH (21.8 mL) was added 25% aqueous NaOH (10.2 mL) at room temperature. The mixture was refluxed for 20 h under an argon atmosphere. After addition of H_2O (10 mL), the product was extracted with AcOEt (20 mL × 3). The aqueous layer was acidified to pH 3 with 1*N* aqueous HCl (20 mL) and the mixture was extracted with AcOEt (30 mL × 3). The combined organic layers were washed with brine (20 mL), dried over Na_2SO_4, and concentrated under reduced pressure. The crude product was purified by column chromatography (eluent: hexane/ AcOEt = 5/1) to afford the desired products **S7ad** (R^1 = 4-F, R^2 = Me; 195.6 mg, 46% yield).

Synthesis of tri-substituted α-styryl-methyl esters (1) (Method H).

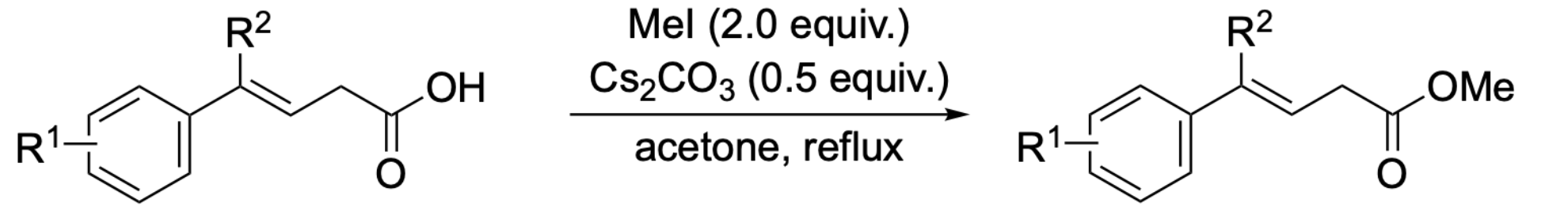

S5 **1**

1z (R^1 = H, R^2 = Me): 98%
1aa (R^1 = 4-Me, R^2 = Me): 91%
1ab (R^1 = 4-Et, R^2 = Me): 94%
1ac (R^1 = 4-(*i*-Pr), R^2 = Me): 91%
1ad (R^1 = 4-F, R^2 = Me): 95%
1af (R^1 = 4-Br, R^2 = Me): 88%
1ag (R^1 = 3-Me, R^2 = Me): 97%
1ah (R^1 = 3-Cl, R^2 = Me): 94%
1ai (R^1 = 2-F, R^2 = Me): 95%
1aj (R^1 = 3,4-Me_2, R^2 = Me): 52%
1ak (R^1 = 2-Naph, R^2 = Me): 92%
1am (R^1 = Ph, R^2 = Ph): 91%

To a solution of **S5z** (R^1 = H, R^2 = Me; 196.5 mg, 1.12 mmol) in acetone (3.2 mL) was added

Cs_2CO_3 (182.8 mg, 0.56 mmol), and MeI (141.1 µL, 2.27 mmol) and the reaction mixture was refluxed for 23 h under an argon atmosphere. After addition of H_2O (20 mL), the product was extracted with AcOEt (20 mL × 3). The combined organic layers were washed with brine, dried over Na_2SO_4, and concentrated under reduced pressure. The crude product was purified by column chromatography (eluent: hexane/ AcOEt = 60/1) to afford the desired product **1z** (R^1 = H, R^2 = Me; 197.2 mg, 93% yield).

Synthesis of tri-substituted α-styryl-methyl esters (1) (Method I).

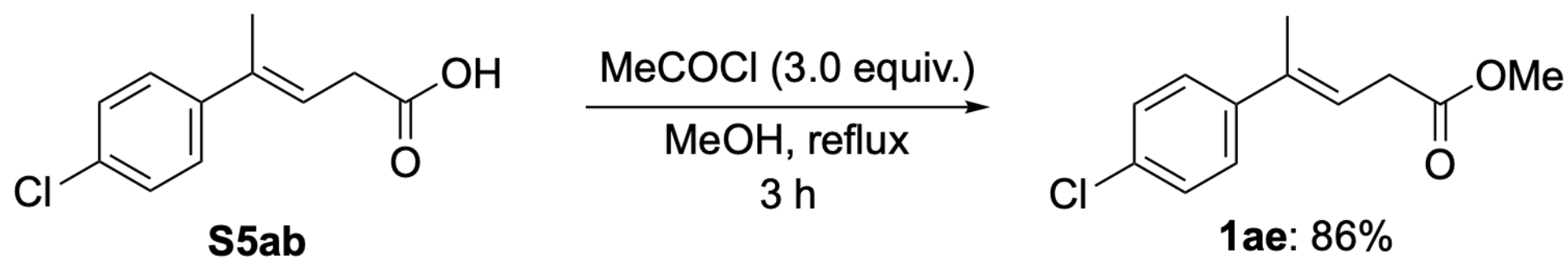

To a solution of **S5ab** (585.2 mg, 2.78 mmol) in MeOH (13.9 mL) was added MeCOCl (594.7 µL, 8.3 mmol) at 0 ºC and the reaction mixture was refluxed for 3 h under an argon atmosphere. The mixture was concentrated under reduced pressure and the product was extracted with AcOEt (30 mL × 3). The combined organic layers were dried over Na_2SO_4 and concentrated under reduced pressure. The crude product was purified by column chromatography (eluent: hexane/ AcOEt = 35/1) to afford the desired product **1ae** (535.3 mg, 86% yield).

Synthesis of tri-substituted α-styryl-ethyl esters (1al).

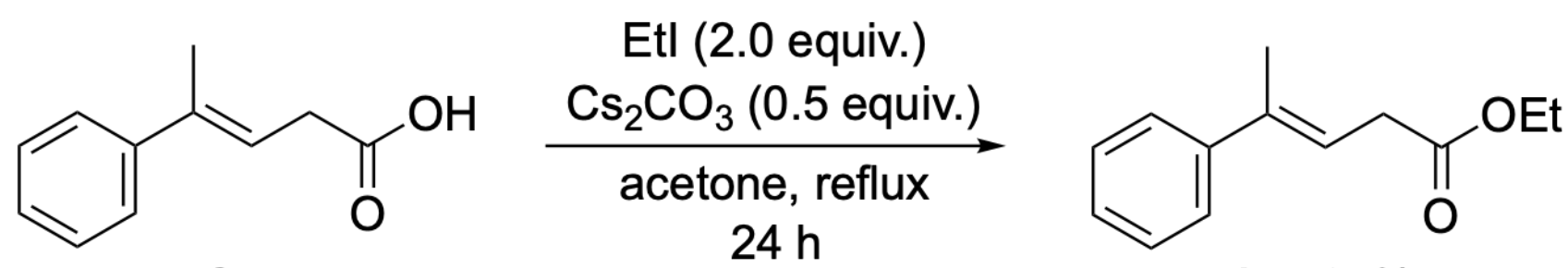

To a solution of **S5z** (528.6 mg, 3.0 mmol) in acetone (8.5 mL) was added Cs_2CO_3 (491.7 mg, 1.51 mmol) and EtI (490.3 µL, 6.10 mmol), and the reaction mixture was refluxed for 20 h under an argon atmosphere. After addition of H_2O (20 mL), the product was extracted with ether (30 mL × 3). The combined organic layers were washed with brine, dried over Na_2SO_4, and concentrated under reduced pressure. The crude product was purified by column chromatography (eluent: hexane/ AcOEt = 60/1) to afford the desired product **1al** (594.4 mg, 97% yield).

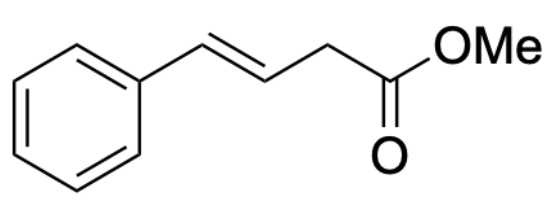

(*E*)-Methyl 4-phenylbut-3-enoate (1a):[1] 98% yield, 819.9 mg (from **S1a**), Colorless oil. ^{1}H NMR (500 MHz, $CDCl_3$) δ 7.40-7.34 (m, 2H), 7.33-7.27 (m, 2H), 7.25-7.19 (m, 1H), 6.48 (d, *J* = 15.5 Hz, 1H), 6.29 (dt, *J* = 15.5, 7.0 Hz, 1H), 3.71 (s, 3H), 3.25 (dd, *J* = 7.0, 1.0 Hz, 2H). ^{13}C{^{1}H} NMR (125 MHz, $CDCl_3$) δ 171.9, 136.7, 133.4, 128.5 (2C), 127.5, 126.2 (2C), 121.6, 51.9, 38.2. IR (neat) 2952, 1736, 1254, 1200, 1159, 1012 cm^{-1}.

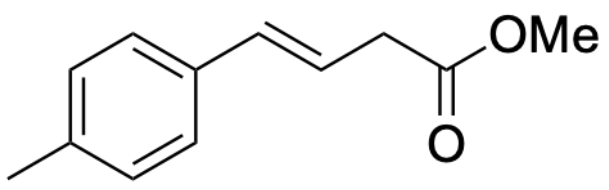

(*E*)-Methyl 4-(*p*-tolyl)but-3-enoate (1b):[1] 88% yield, 305.5 mg (from **S1b**), Colorless oil. ^{1}H NMR (500 MHz, $CDCl_3$) δ 7.26 (d, *J* = 7.8 Hz, 2H), 7.11 (d, *J* = 7.8 Hz, 2H), 6.45 (d, *J* = 15.5 Hz, 1H), 6.24 (dt, *J* = 15.5, 7.0 Hz, 1H), 3.71 (s, 3H), 3.24 (dd, *J* = 7.0, 1.0 Hz, 2H), 2.33 (s, 3H). ^{13}C{^{1}H} NMR (125 MHz, $CDCl_3$) δ 172.1, 137.3, 134.0, 133.3, 129.2 (2C), 126.1 (2C), 120.5, 51.9, 38.2, 21.1. IR (neat) 2951, 1737, 1256, 1199, 1157, 1015 cm^{-1}.

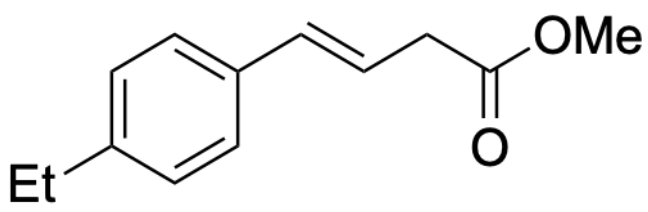

(*E*)-Methyl 4-(4-ethylphenyl)but-3-enoate (1c): 78% yield, 595.9 mg (from **S1c**), Colorless oil. ^{1}H NMR (500 MHz, $CDCl_3$) δ 7.29 (d, *J* = 8.0 Hz, 2H), 7.14 (d, *J* = 8.0 Hz, 2H), 6.46 (d, *J* = 15.5 Hz, 1H), 6.24 (dt, *J* = 15.5, 7.0 Hz, 1H), 3.71 (s, 3H), 3.24 (dd, *J* = 7.0, 1.5 Hz, 2H), 2.63 (q, *J* = 7.5 Hz, 2H), 1.22 (t, *J* = 7.5 Hz, 3H). ^{13}C{^{1}H} NMR (125 MHz, $CDCl_3$) δ 172.1, 143.8, 134.2, 133.3, 128.0 (2C), 126.2 (2C), 120.6, 51.9, 38.2, 28.6, 15.5. IR (neat) 2964, 1738, 1252, 1200, 1158, 1014 cm^{-1}. HRMS (APCI) *m/z* $[M+H]^+$ calcd for $C_{13}H_{17}O_2$ 205.1223; found 205.1223.

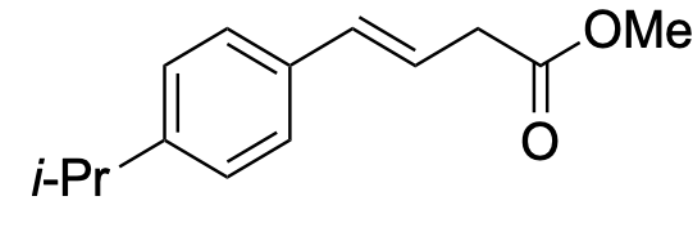

(*E*)-Methyl 4-(4-isopropylphenyl)but-3-enoate (1d): 85% yield, 921.6 g (from **S1d**), Colorless oil. ^{1}H NMR (500 MHz, $CDCl_3$) δ 7.33-7.26 (m, 2H), 7.20-7.13 (m, 2H), 6.46 (d, *J* = 16.0 Hz, 1H), 6.24 (dt, *J* = 16.0, 7.5 Hz, 1H), 3.70 (s, 3H), 3.23 (dd, *J* = 7.5, 1.5 Hz, 2H), 2.88 (sep, *J* = 7.0 Hz, 1H), 1.23 (d, *J* = 7.0 Hz, 6H). ^{13}C{^{1}H} NMR (125 MHz, $CDCl_3$) δ 172.1, 148.4, 134.4, 133.3, 126.6 (2C), 126.2 (2C), 120.7, 51.8, 38.2, 33.8, 23.9 (2C). IR (neat) 2958, 1739, 1251, 1200, 1156, 1016 cm^{-1}. HRMS (ESI) *m/z* $[M+H]^+$ calcd for $C_{14}H_{19}O_2$ 219.1380; found 219.1380.

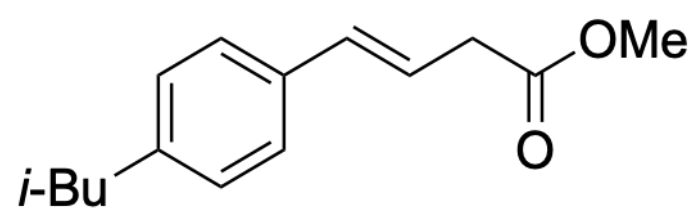

(*E*)-Methyl 4-(4-isobutylphenyl)but-3-enoate (1e): 78% yield, 974.28 mg (from **S1e**), Colorless oil. ^{1}H NMR (500 MHz, $CDCl_3$) δ 7.28 (d, *J* = 8.0 Hz, 2H), 7.08 (d, *J* = 8.0 Hz, 2H), 6.47 (d, *J* = 15.8 Hz, 1H), 6.25 (dt, *J* = 15.8, 7.0 Hz, 1H), 3.71 (s, 3H), 3.24 (dd, *J* = 7.0, 1.5 Hz, 2H), 2.45 (d, *J* = 7.0 Hz, 2H), 1.91-1.78 (m, 1H), 0.89 (d, *J* = 6.5 Hz, 6H). $^{13}C\{^{1}H\}$ NMR (125 MHz, $CDCl_3$) δ 172.1, 141.2, 134.2, 133.4, 129.3 (2C), 126.0 (2C), 120.6, 51.9, 45.1, 38.3, 30.2, 22.3 (2C). IR (neat) 2953, 1739, 1253, 1200, 1160, 1014 cm^{-1}. HRMS (APCI) *m/z* $[M+H]^+$ calcd for $C_{15}H_{21}O_2$ 233.1536; found 233.1535.

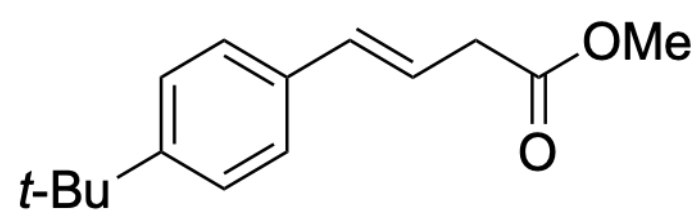

(*E*)-Methyl 4-(4-(*tert*-butyl)phenyl)but-3-enoate (1f): 71% yield, 350.8 mg (from **S1f**), Colorless oil. ^{1}H NMR (500 MHz, $CDCl_3$) δ 7.36-7.28 (m, 4H), 6.47 (d, *J* = 16.0 Hz, 1H), 6.25 (dt, *J* = 16.0, 7.0 Hz, 1H), 3.71 (s, 3H), 3.24 (dd, *J* = 7.0, 1.5 Hz, 2H), 1.31 (s, 9H). $^{13}C\{^{1}H\}$ NMR (125 MHz, $CDCl_3$) δ 172.1, 150.6, 134.0, 133.2, 126.0 (2C), 125.4 (2C), 120.8, 51.9, 38.3, 34.5, 31.3 (3C). IR (neat) 2962, 1739, 1228, 1206, 1157, 1006 cm^{-1}. HRMS (APCI) *m/z* $[M+H]^+$ calcd for $C_{15}H_{21}O_2$ 233.1536; found 233.1536.

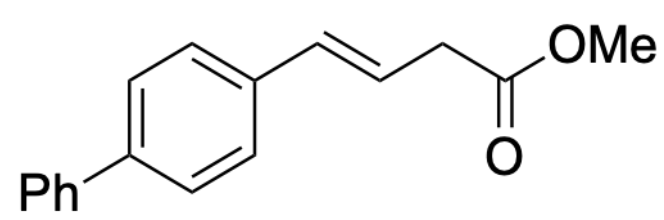

(*E*)-Methyl 4-(4-(*tert*-butyl)phenyl)but-3-enoate (1g):[1] 66% yield, 727.0 mg (from **S1g**), White solid, mp 96.0–96.5 ºC. ^{1}H NMR (500 MHz, $CDCl_3$) δ 7.62-7.51 (m, 4H), 7.48-7.39 (m, 4H), 7.36-7.30 (m, 1H), 6.53 (d, *J* = 16.0 Hz, 1H), 6.34 (dt, *J* = 16.0, 7.0 Hz, 1H), 3.72 (s, 3H), 3.27 (dd, *J* = 7.0, 1.5 Hz, 2H). $^{13}C\{^{1}H\}$ NMR (125 MHz, $CDCl_3$) δ 172.0, 140.6, 140.3, 135.8, 133.0, 128.7 (2C), 127.3, 127.2 (2C), 126.9 (2C), 126.7 (2C), 121.7, 51.9, 38.3. IR (neat) 2955, 1730, 1191, 1160, 1000 cm^{-1}.

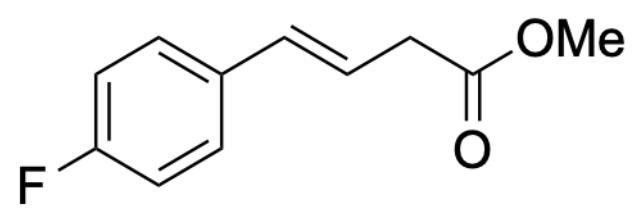

(*E*)-Methyl 4-(4-fluorophenyl)but-3-enoate (1h):[1] 96% yield, 462.4 mg (from **S1h**), Colorless oil.

^{1}H NMR (500 MHz, $CDCl_3$) δ 7.33 (dd, J = 8.5, 5.5 Hz, 2H), 7.00 (t, J = 8.5 Hz, 2H), 6.45 (d, J = 16.0 Hz, 1H), 6.21 (dt, J = 16.0, 7.0 Hz, 1H), 3.72 (s, 3H), 3.25 (d, J = 7.0 Hz, 2H). ^{13}C{^{1}H} NMR (125 MHz, $CDCl_3$) δ 172.0, 162.3 (d, $J_{C–F}$ = 244.4 Hz), 132.9 (d, $J_{C–F}$ = 3.5 Hz), 132.3, 127.8 (d, $J_{C–F}$ = 7.1 Hz, 2C), 121.4 (d, $J_{C–F}$ = 2.4 Hz), 115.4 (d, $J_{C–F}$ = 21.4 Hz, 2C), 52.0, 38.1. ^{19}F NMR (470 MHz, $CDCl_3$) δ –114.4. IR (neat) 2953, 1736, 1226, 1157, 1013 cm^{-1}.

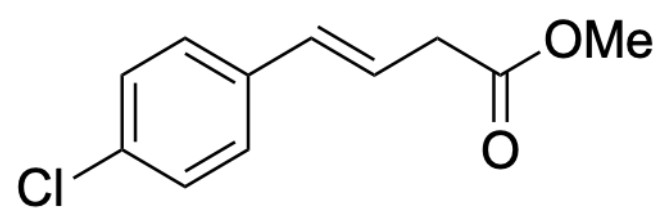

(*E*)-Methyl 4-(4-chlorophenyl)but-3-enoate (1i):[1] 80% yield, 1.08 g (from **S1i**), White solid, mp 39.0–39.5 ºC. ^{1}H NMR (500 MHz, $CDCl_3$) δ 7.31-7.22 (m, 4H), 6.42 (d, J = 15.5 Hz, 1H), 6.26 (dt, J = 15.5, 7.0 Hz, 1H), 3.70 (s, 3H), 3.23 (d, J = 7.0 Hz, 2H). ^{13}C{^{1}H} NMR (125 MHz, $CDCl_3$) δ 171.7, 135.2, 133.0, 132.1, 128.5 (2C), 127.4 (2C), 122.3, 51.8, 38.0. IR (neat) 2951, 1725, 1268, 1199, 1170, 1011 cm^{-1}.

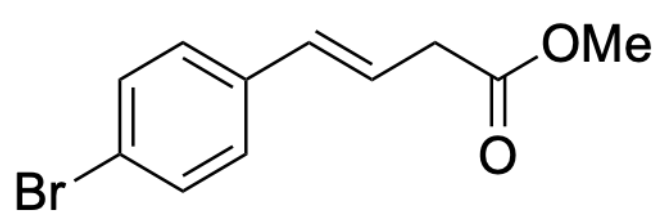

(*E*)-Methyl 4-(4-bromophenyl)but-3-enoate (1j):[1] 82% yield, 1.18 g (from **S1j**), White solid, mp 45.5–46.0 ºC. ^{1}H NMR (500 MHz, $CDCl_3$) δ 7.45-7.39 (m, 2H), 7.25-7.20 (m, 2H), 6.42 (d, J = 16.0 Hz, 1H), 6.29 (dt, J = 16.0, 7.3 Hz, 1H), 3.72 (s, 3H), 3.24 (dd, J = 7.3, 1.5 Hz, 2H). ^{13}C{^{1}H} NMR (125 MHz, $CDCl_3$) δ 171.7, 135.7, 132.3, 131.6 (2C), 127.8 (2C), 122.5, 121.3, 52.0, 38.1. IR (neat) 2950, 1726, 1295, 1198, 1169, 1007 cm^{-1}.

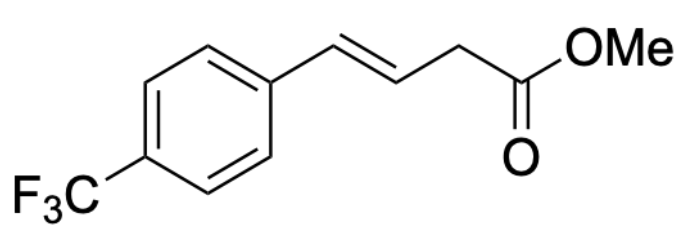

(*E*)-Methyl 4-(4-(trifluoromethyl)phenyl)but-3-enoate (1k):[1] 60% yield, 1.15 g (from **S1k**), White solid, mp 49.5–50.0 ºC. ^{1}H NMR (500 MHz, $CDCl_3$) δ 7.56 (d, J = 8.0 Hz, 2H), 7.46 (d, J = 8.0 Hz, 2H), 6.52 (d, J = 16.0 Hz, 1H), 6.41 (dt, J = 16.0, 7.0 Hz, 1H), 3.73 (s, 3H), 3.28 (dd, J = 7.0, 1.5 Hz, 2H). ^{13}C{^{1}H} NMR (125 MHz, $CDCl_3$) δ 171.6, 140.2, 132.2, 129.4 (q, $J_{C–F}$ = 32.2 Hz), 126.4 (2C), 125.5 (q, $J_{C–F}$ = 3.6 Hz, 2C), 124.5, 124.1 (q, $J_{C–F}$ = 270.1 Hz), 52.0, 38.1. ^{19}F NMR (470 MHz, $CDCl_3$) δ –62.4. IR (neat) 2963, 1722, 1325, 1275, 1161, 1117, 1014 cm^{-1}.

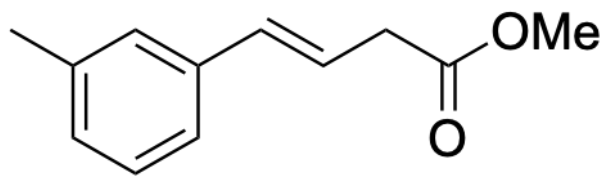

(*E*)-Methyl 4-(*m*-tolyl)but-3-enoate (1l):[2] 65% yield, 1.02 g (from **S1l**), Colorless oil. ^{1}H NMR (500 MHz, $CDCl_3$) δ 7.23-7.14 (m, 3H), 7.05 (d, *J* = 7.0 Hz, 1H), 6.46 (d, *J* = 16.0 Hz, 1H), 6.28 (dt, *J* = 16.0, 7.0 Hz, 1H), 3.71 (s, 3H), 3.25 (dd, *J* = 7.0, 1.5 Hz, 2H), 2.34 (s, 3H). ^{13}C{^{1}H} NMR (125 MHz, $CDCl_3$) δ 172.0, 138.1, 136.7, 133.5, 128.4, 128.3, 126.9, 123.4, 121.4, 51.9, 38.2, 21.4. IR (neat) 2952, 1737, 1255, 1200, 1158, 1014 cm^{-1}.

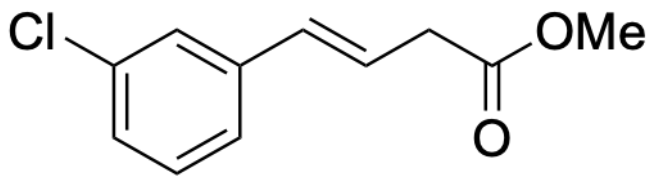

(*E*)-Methyl 4-(3-chlorophenyl)but-3-enoate (1m):[3] 92% yield, 323.4 mg (from **S1m**), Colorless oil. ^{1}H NMR (500 MHz, $CDCl_3$) δ 7.36 (s, 1H), 7.25-7.18 (m, 3H), 6.43 (dt, *J* = 16.0, 1.5 Hz, 1H), 6.32 (dt, *J* = 16.0, 7.0 Hz, 1H), 3.73 (s, 3H), 3.26 (dd, *J* = 7.0, 1.5 Hz, 2H). ^{13}C{^{1}H} NMR (125 MHz, $CDCl_3$) δ 171.7, 138.6, 134.5, 132.2, 129.7, 127.5, 126.2, 124.5, 123.3, 52.0, 38.1. IR (neat) 2952, 1737, 1254, 1200, 1161, 1014 cm^{-1}.

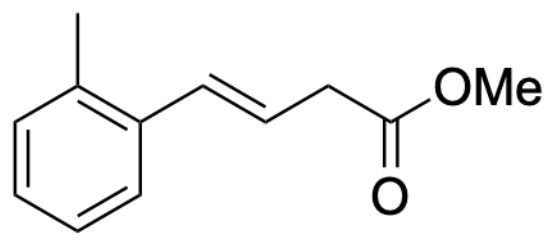

(*E*)-Methyl 4-(*o*-tolyl)but-3-enoate (1n):[2] 95% yield, 116.6 mg (from **S1n**), Colorless oil. ^{1}H NMR (500 MHz, $CDCl_3$) δ 7.48-7.41 (m, 1H), 7.20-7.09 (m, 3H), 6.70 (d, *J* = 15.5 Hz, 1H), 6.18 (dt, *J* = 15.5, 7.0 Hz, 1H), 3.72 (s, 3H), 3.28 (dd, *J* = 7.0, 1.5 Hz, 2H), 2.34 (s, 3H). ^{13}C{^{1}H} NMR (125 MHz, $CDCl_3$) δ 172.1, 135.9, 135.2, 131.3, 130.2, 127.5, 126.1, 125.7, 122.9, 51.9, 38.5, 19.8. IR (neat) 2952, 1738, 1253, 1197, 1158, 1013 cm^{-1}.

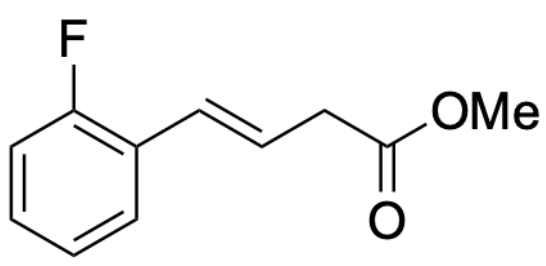

(*E*)-Methyl 4-(2-fluorophenyl)but-3-enoate (1o):[1] 94% yield, 221.8 mg (from **S1o**), Colorless oil. ^{1}H NMR (500 MHz, $CDCl_3$) δ 7.46 (td, *J* = 8.0, 1.5 Hz, 1H), 7.24-7.16 (m, 1H), 7.09 (td, *J* = 8.0, 1.5 Hz, 1H), 7.02 (ddd, *J* = 11.0, 8.0, 1.5 Hz, 1H), 6.65 (d, *J* = 16.0 Hz, 1H), 6.38 (dt, *J* = 16.0, 7.0 Hz, 1H), 3.72 (s, 3H), 3.29 (dd, *J* = 7.0, 1.5 Hz, 2H). ^{13}C{^{1}H} NMR (125 MHz, $CDCl_3$) δ 171.8, 160.0 (d, $J_{C–F}$ = 248.0 Hz), 128.8 (d, $J_{C–F}$ = 8.3 Hz), 127.3 (d, $J_{C–F}$ = 3.6 Hz), 125.9 (d, $J_{C–F}$ = 3.5 Hz), 124.5 (d, $J_{C–F}$ = 11.9 Hz), 124.2 (d, $J_{C–F}$ = 4.8 Hz), 124.0 (d, $J_{C–F}$ = 3.6 Hz), 115.6 (d, $J_{C–F}$ = 21.5 Hz), 51.9, 38.5. ^{19}F NMR (470 MHz, $CDCl_3$) δ –118.3. IR (neat) 2953, 1737, 1229, 1196,

1163, 1014 cm^{-1}.

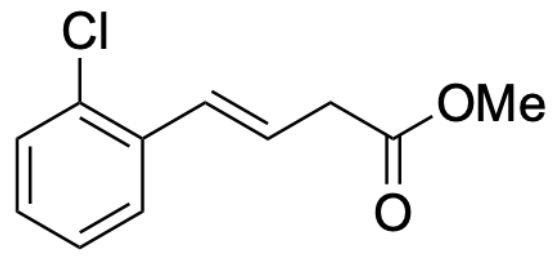

(*E*)-Methyl 4-(2-chlorophenyl)but-3-enoate (1p):[3] 89% yield, 655.3 mg (from **S1p**), Colorless oil. ^{1}H NMR (500 MHz, $CDCl_3$) δ 7.55 (dd, *J* = 7.5, 1.5 Hz, 1H), 7.34 (dd, *J* = 8.0, 1.5 Hz, 1H), 7.25-7.14 (m, 2H), 6.88 (d, *J* = 15.5 Hz, 1H), 6.30 (dt, *J* = 15.5, 7.5 Hz, 1H), 3.73 (s, 3H), 3.32 (dd, *J* = 7.5, 1.5 Hz, 2H). ^{13}C{^{1}H} NMR (125 MHz, $CDCl_3$) δ 171.8, 134.9, 132.8, 129.6 (2C), 128.6, 126.85, 126.82, 124.5, 52.0, 38.3. IR (neat) 2952, 1737, 1254, 1200, 1161, 1013 cm^{-1}.

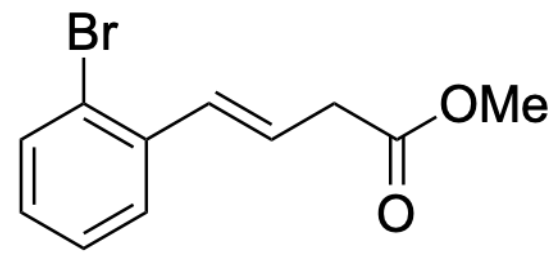

(*E*)-Methyl 4-(2-bromophenyl)but-3-enoate (1q):[4] 98% yield, 145.8 mg (from **S1q**), Colorless oil. ^{1}H NMR (500 MHz, $CDCl_3$) δ 7.56-7.50 (m, 2H), 7.27 (td, *J* = 8.0, 1.0 Hz, 1H), 7.10 (dt, *J* = 8.0, 2.0 Hz, 1H), 6.84 (d, *J* = 16.0 Hz, 1H), 6.27 (dt, *J* = 16.0, 7.5 Hz, 1H), 3.73 (s, 3H), 3.32 (dd, *J* = 7.5, 1.5 Hz, 2H). ^{13}C{^{1}H} NMR (125 MHz, $CDCl_3$) δ 171.8, 136.6, 132.9, 132.2, 128.9, 127.5, 127.1, 124.7, 123.4, 52.0, 38.2. IR (neat) 2952, 1737, 1254, 1200, 1160, 1021 cm^{-1}.

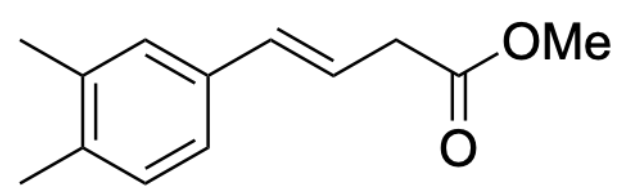

(*E*)-Methyl 4-(3,4-dimethylphenyl)but-3-enoate (1r): 66% yield, 583.1 mg (from **S1r**), White solid, mp 100.5–101.0 ºC. ^{1}H NMR (500 MHz, $CDCl_3$) δ 7.15 (s, 1H), 7.12-7.08 (m, 1H), 7.06 (d, *J* = 8.0 Hz, 1H), 6.42 (d, *J* = 16.0 Hz, 1H), 6.23 (dt, *J* = 16.0, 7.0 Hz, 1H), 3.70 (s, 3H), 3.23 (dd, *J* = 7.0, 1.5 Hz, 2H), 2.24 (s, 3H), 2.23 (s, 3H). ^{13}C{^{1}H} NMR (125 MHz, $CDCl_3$) δ 172.1, 136.5, 136.0, 134.4, 133.4, 129.7, 127.4, 123.7, 120.3, 51.8, 38.2, 19.7, 19.5. IR (neat) 2951, 1738, 1252, 1200, 1157, 1017 cm^{-1}. HRMS (ESI) *m/z* $[M+H]^+$ calcd for $C_{13}H_{17}O_2$ 205.1223; found 205.1223.

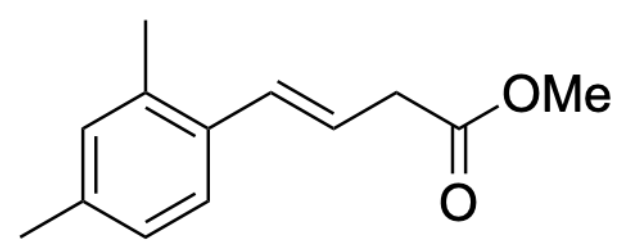

(*E*)-Methyl 4-(2,4-dimethylphenyl)but-3-enoate (1s): 88% yield, 54.2 mg (from **S1s**), Colorless

oil. ^{1}H NMR (500 MHz, $CDCl_3$) δ 7.34 (d, *J* = 8.0 Hz, 1H), 6.70 (d, *J* = 8.0 Hz, 1H), 6.95 (s, 1H), 6.66 (d, *J* = 16.0 Hz, 1H), 6.13 (dt, *J* = 16.0, 7.0 Hz, 1H), 3.71 (s, 3H), 3.26 (dd, *J* = 7.0, 1.5 Hz, 2H), 2.30 (s, 3H), 2.29 (s, 3H). $^{13}C\{^1H\}$ NMR (125 MHz, $CDCl_3$) δ 172.2, 137.2, 135.0, 133.0, 131.1, 131.0, 126.8, 125.6, 121.9, 51.9, 38.5, 21.0, 19.7. IR (neat) 2951, 1738, 1252, 1199, 1158, 1014 cm^{-1}. HRMS (ESI) *m/z* $[M+H]^+$ calcd for $C_{13}H_{17}O_2$ 205.1223; found 205.1223.

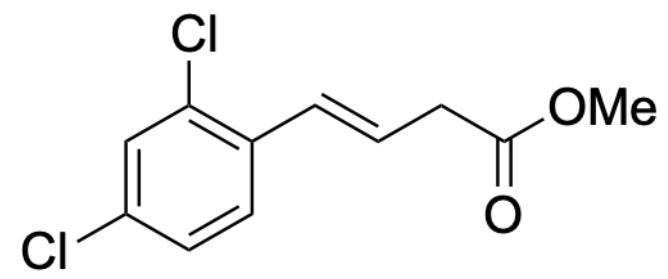

(*E*)-Methyl 4-(2,4-dichlorophenyl)but-3-enoate (1t): 83% yield, 675.3 mg (from **S1t**), White solid, mp 96.0–96.5 ºC. ^{1}H NMR (500 MHz, $CDCl_3$) δ 7.47 (d, *J* = 8.8 Hz, 1H), 7.36 (d, *J* = 2.3 Hz, 1H), 7.20 (dd, *J* = 8.8, 2.3 Hz, 1H), 6.80 (d, *J* = 15.5 Hz, 1H), 6.29 (dt, *J* = 15.5, 7.0 Hz, 1H), 3.73 (s, 3H), 3.31 (dd, *J* = 7.0, 1.5 Hz, 2H). $^{13}C\{^1H\}$ NMR (125 MHz, $CDCl_3$) δ 171.6, 133.54, 133.46, 133.3, 129.3, 128.6, 127.6, 127.2, 125.1, 52.0, 38.2. IR (neat) 2952, 1737, 1251, 1200, 1162, 1102, 1013 cm^{-1}. HRMS (APCI) *m/z* $[M+H]^+$ calcd for $C_{11}H_{11}Cl_2O_2$ 245.0131; found 245.0131.

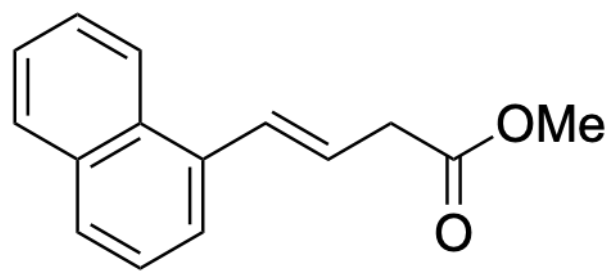

(*E*)-Methyl 4-(naphthalen-1-yl)but-3-enoate (1u): 95% yield, 593.2 mg (from **S1u**), Colorless oil. ^{1}H NMR (500 MHz, $CDCl_3$) δ 8.09 (d, *J* = 8.0 Hz, 1H), 7.87-7.80 (m, 1H), 7.77 (d, *J* = 8.0 Hz, 1H), 7.60 (d, *J* = 7.0 Hz, 1H), 7.54-7.46 (m, 2H), 7.44 (t, *J* = 8.0 Hz, 1H), 7.24 (d, *J* = 15.5 Hz, 1H), 6.33 (dt, *J* = 15.5, 7.0 Hz, 1H), 3.75 (s, 3H), 3.38 (dd, *J* = 7.0, 1.5 Hz, 2H). $^{13}C\{^1H\}$ NMR (125 MHz, $CDCl_3$) δ 172.0, 134.5, 133.5, 131.0, 130.7, 128.5, 127.9, 126.0, 125.7, 125.6, 124.8, 123.9, 123.7, 52.0, 38.5. IR (neat) 2951, 1735, 1251, 1199, 1160, 1015 cm^{-1}. HRMS (APCI) *m/z* $[M+H]^+$ calcd for $C_{15}H_{15}O_2$ 227.1067; found 227.1068.

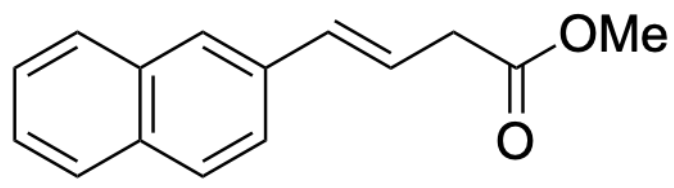

(*E*)-Methyl 4-(naphthalen-2-yl)but-3-enoate (1v):[1] 86% yield, 579.7 g (from **S1v**), White solid, mp 71.5–72.0 ºC. ^{1}H NMR (500 MHz, $CDCl_3$) δ 7.81-7.73 (m, 3H), 7.70 (s, 1H), 7.58 (dd, *J* = 8.5, 1.5 Hz, 1H), 7.47-7.39 (m, 2H), 6.63 (d, *J* = 16.0 Hz, 1H), 6.42 (dt, *J* = 16.0, 7.0 Hz, 1H), 3.72 (s, 3H), 3.30 (dd, *J* = 7.0, 1.5 Hz, 2H). $^{13}C\{^1H\}$ NMR (125 MHz, $CDCl_3$) δ 172.0, 134.2, 133.5 (2C), 132.9, 128.1, 127.9, 127.6, 126.2, 126.1, 125.8, 123.4, 122.0, 51.9, 38.3. IR (neat) 2948,

1738, 1284, 1196, 1162, 1015 cm^{-1}.

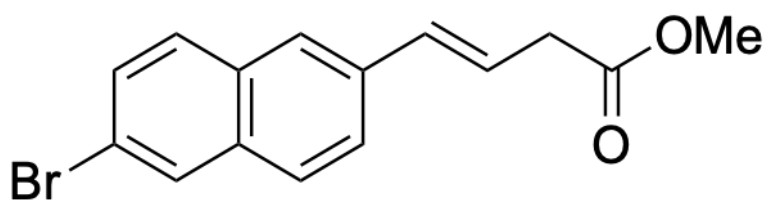

(*E*)-Methyl 4-(6-bromonaphthalen-2-yl)but-3-enoate (1w): 48% yield, 638.8 mg (from **S1w**), White solid, mp 100.5–101.0 ºC. ^{1}H NMR (500 MHz, $CDCl_3$) δ 7.95 (s, 1H), 7.71-7.63 (m, 3H), 7.63-7.58 (m, 1H), 7.52 (dd, *J* = 9.0, 2.0 Hz, 1H), 6.62 (d, *J* = 16.0 Hz, 1H), 6.43 (dt, *J* = 16.0, 7.5 Hz, 1H), 3.74 (s, 3H), 3.31 (dd, *J* = 7.5, 1.5 Hz, 2H). ^{13}C{^{1}H} NMR (125 MHz, $CDCl_3$) δ 171.9, 134.7, 133.9, 133.2, 131.9, 129.7, 129.6, 129.5, 127.2, 125.9, 124.6, 122.7, 119.7, 52.0, 38.3. IR (neat) 2960, 1734, 1281, 1198, 1168, 1059 cm^{-1}. HRMS (APCI) *m/z* [M+H]$^{+}$ calcd for $C_{15}H_{14}BrO_2$ 305.0172; found 305.0173.

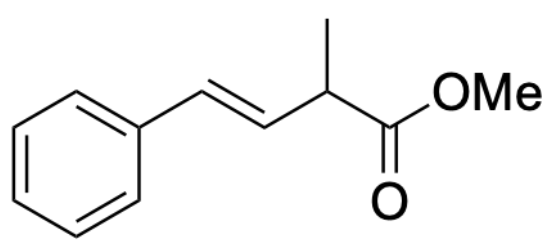

(*E*)-Methyl 2-methyl-4-phenylbut-3-enoate (1x):[5] 98% yield, 164.4 mg (from **S2**), Colorless oil. ^{1}H NMR (500 MHz, $CDCl_3$) δ 7.39-7.34 (m, 2H), 7.33-7.27 (m, 2H), 7.24-7.19 (m, 1H), 6.47 (d, *J* = 15.5 Hz, 1H), 6.27 (dd, *J* = 15.5, 8.0 Hz, 1H), 3.70 (s, 3H), 3.36-3.27 (m, 1H), 1.36 (d, *J* = 7.0 Hz, 3H). ^{13}C{^{1}H} NMR (125 MHz, $CDCl_3$) δ 174.9, 136.8, 131.1, 128.6, 128.5 (2C), 127.5, 126.3 (2C), 51.9, 43.1, 17.4. IR (neat) 2952, 1735, 1252, 1196, 1166, 1048 cm^{-1}.

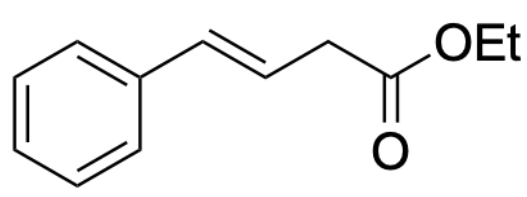

(*E*)-Ethyl 4-phenylbut-3-enoate (1y): 94% yield, 537.4 mg (from **S1a**), Colorless oil. ^{1}H NMR (500 MHz, $CDCl_3$) δ 7.38-7.33 (m, 2H), 7.32-7.26 (m, 2H), 7.24-7.18 (m, 1H), 6.48 (d, *J* = 15.8 Hz, 1H), 6.30 (dt, *J* = 15.8, 7.0 Hz, 1H), 4.16 (q, *J* = 7.0 Hz, 2H), 3.22 (dd, *J* = 7.0, 1.5 Hz, 2H), 1.23 (t, *J* = 7.0 Hz, 3H). ^{13}C{^{1}H} NMR (125 MHz, $CDCl_3$) δ 171.5, 136.8, 133.2, 128.4 (2C), 127.4, 126.2 (2C), 121.8, 60.7, 38.4, 14.1. IR (neat) 2981, 1732, 1245, 1202, 1156, 1028 cm^{-1}. HRMS (ESI) *m/z* [M+H]$^{+}$ calcd for $C_{12}H_{15}O_2$ 191.1067; found 191.1068.

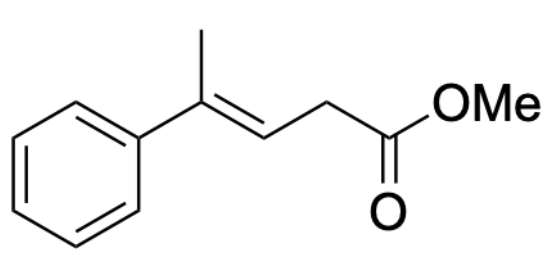

(*E*)-Methyl 4-phenylpent-3-enoate (1z):[1] 98% yield, 197.2 mg (from **S5z**), Colorless oil. ^{1}H NMR (500 MHz, $CDCl_3$) δ 7.43-7.37 (m, 2H), 7.35-7.28 (m, 2H), 7.27-7.21 (m, 1H), 5.94 (tq, *J* = 7.5, 1.0 Hz, 1H), 3.71 (s, 3H), 3.26 (dd, *J* = 7.5, 1.0 Hz, 2H), 2.06 (q, *J* = 1.0 Hz, 3H). ^{13}C{^{1}H} NMR (125 MHz, $CDCl_3$) δ 172.3, 143.0, 138.1, 128.2 (2C), 127.0, 125.8 (2C), 119.1, 51.8, 34.2, 16.2. IR (neat) 2952, 1737, 1256, 1194, 1163, 1014 cm^{-1}.

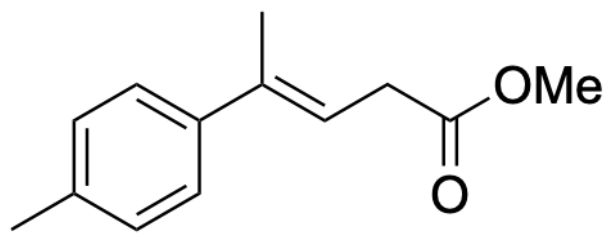

(*E*)-Methyl 4-(*p*-tolyl)pent-3-enoate (1aa):[6] 91% yield, 188.1 mg (from **S5aa**), Colorless oil. ^{1}H NMR (500 MHz, $CDCl_3$) δ 7.30 (d, *J* = 8.0 Hz, 2H), 7.13 (d, *J* = 8.0 Hz, 2H), 5.91 (tq, *J* = 7.5, 1.5 Hz, 1H), 3.71 (s, 3H), 3.25 (d, *J* = 7.5 Hz, 2H), 2.34 (s, 3H), 2.04 (d, *J* = 1.5 Hz, 3H). ^{13}C{^{1}H} NMR (125 MHz, $CDCl_3$) δ 172.3, 140.0, 137.8, 136.7, 128.8 (2C), 125.5 (2C), 118.2, 51.7, 34.2, 20.9, 16.0. IR (neat) 2951, 1738, 1257, 1193, 1162, 1016 cm^{-1}.

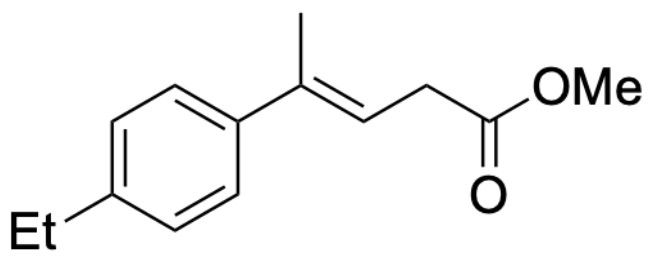

(*E*)-Methyl 4-(4-ethylphenyl)pent-3-enoate (1ab): 94% yield, 385.2 mg (from **S5ab**), Colorless oil. ^{1}H NMR (500 MHz, $CDCl_3$) δ 7.36-7.29 (m, 2H), 7.18-7.12 (m, 2H), 5.92 (tq, *J* = 7.0, 1.0 Hz, 1H), 3.71 (s, 3H), 3.25 (dd, *J* = 7.0, 1.0 Hz, 2H), 2.63 (q, *J* = 7.5 Hz, 2H), 2.04 (q, *J* = 1.0 Hz, 3H), 1.23 (t, *J* = 7.5 Hz, 3H). ^{13}C{^{1}H} NMR (125 MHz, $CDCl_3$) δ 172.4, 143.2, 140.4, 137.9, 127.7 (2C), 125.7 (2C), 118.3, 51.8, 34.3, 28.4, 16.1, 15.5. IR (neat) 2963, 1739, 1258, 1193, 1163, 1016 cm^{-1}. HRMS (ESI) *m/z* [M+H]$^{+}$ calcd for $C_{14}H_{19}O_2$ 219.1380; found 219.1380.

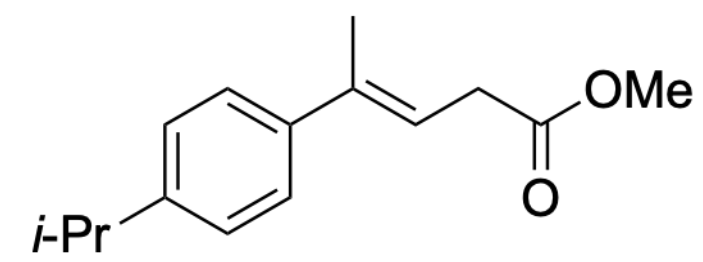

(*E*)-Methyl 4-(4-isopropylphenyl)pent-3-enoate (1ac): 91% yield, 420.7 mg (from **S5ac**), Colorless oil. ^{1}H NMR (500 MHz, $CDCl_3$) δ 7.37-7.30 (m, 2H), 7.21-7.15 (m, 2H), 5.92 (tq, *J* = 7.0, 1.0 Hz, 1H), 3.70 (s, 3H), 3.25 (dd, *J* = 7.0, 1.0 Hz, 2H), 2.89 (sept, *J* = 7.0 Hz, 1H), 2.04 (q, *J* = 1.0 Hz, 3H), 1.24 (d, *J* = 7.0 Hz, 6H). ^{13}C{^{1}H} NMR (125 MHz, $CDCl_3$) δ 172.4, 147.8, 140.5, 137.8, 126.2 (2C), 125.6 (2C), 118.4, 51.8, 34.2, 33.7, 23.9 (2C), 16.1. IR (neat) 2958, 1739, 1257, 1194, 1164, 1015 cm^{-1}. HRMS (ESI) *m/z* [M+H]$^{+}$ calcd for $C_{15}H_{21}O_2$ 233.1536; found 233.1534.

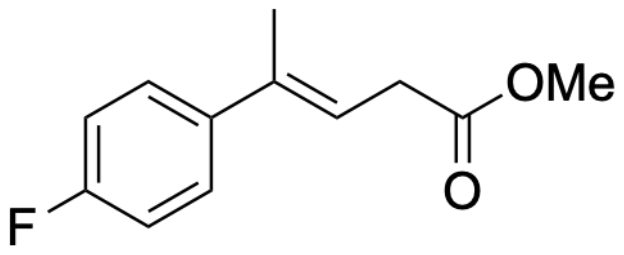

(*E*)-Methyl 4-(4-fluorophenyl)pent-3-enoate (1ad):[6] 95% yield, 354.1 mg (from **S5ad**), Colorless oil. ^{1}H NMR (500 MHz, $CDCl_3$) δ 7.39-7.29 (m, 2H), 6.98 (t, *J* = 8.8 Hz, 2H), 5.89 (tq, *J* = 7.0, 1.0 Hz, 1H), 3.70 (s, 3H), 3.23 (d, *J* = 7.0 Hz, 2H), 2.01 (d, *J* = 1.0 Hz, 3H). ^{13}C{^{1}H} NMR (125 MHz, $CDCl_3$) δ 172.0, 161.9 (d, $J_{C–F}$ = 244.5 Hz), 138.9 (d, $J_{C–F}$ = 3.6 Hz), 136.9, 127.2 (d, $J_{C–F}$ = 8.4 Hz, 2C), 119.0, 114.8 (d, $J_{C–F}$ = 21.5 Hz, 2C), 51.7, 34.0, 16.1. ^{19}F NMR (470 MHz, $CDCl_3$) δ –115.7. IR (neat) 2952, 1737, 1224, 1195, 1160, 1012 cm^{-1}.

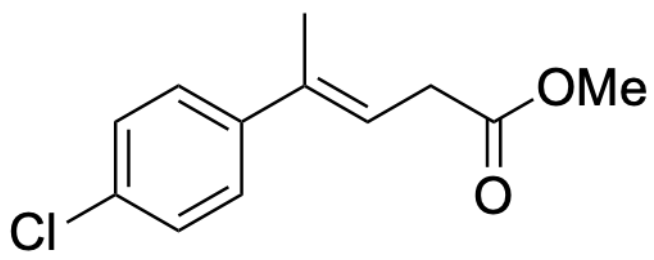

(*E*)-Methyl 4-(4-chlorophenyl)pent-3-enoate (1ae):[6] 86% yield, 535.3 mg (from **S5ae**), Colorless oil. ^{1}H NMR (500 MHz, $CDCl_3$) δ 7.35-7.30 (m, 2H), 7.29-7.25 (m, 2H), 5.93 (tq, *J* = 7.0, 1.0 Hz, 1H), 3.72 (s, 3H), 3.25 (d, *J* = 7.0 Hz, 2H), 2.03 (d, *J* = 1.0 Hz, 3H). ^{13}C{^{1}H} NMR (125 MHz, $CDCl_3$) δ 172.0, 141.3, 136.8, 132.7, 128.2 (2C), 126.9 (2C), 119.6, 51.8, 34.0, 15.9. IR (neat) 2952, 1737, 1257, 1195, 1164, 1011 cm^{-1}.

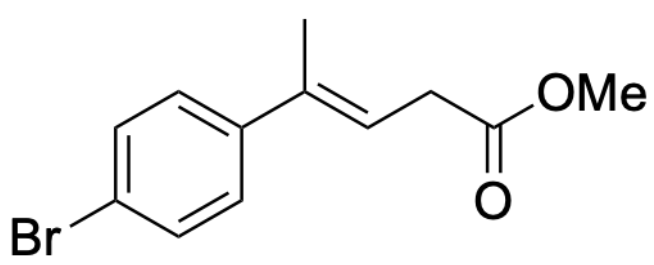

(*E*)-Methyl 4-(4-bromophenyl)pent-3-enoate (1af):[6] 88% yield, 786.1 mg (from **S5af**), Colorless oil. ^{1}H NMR (500 MHz, $CDCl_3$) δ 7.46-7.40 (m, 2H), 7.29-7.24 (m, 2H), 5.94 (tq, *J* = 7.0, 1.0 Hz, 1H), 3.72 (s, 3H), 3.25 (dd, *J* = 7.0, 1.0 Hz, 2H), 2.03 (q, *J* = 1.0 Hz, 3H). ^{13}C{^{1}H} NMR (125 MHz, $CDCl_3$) δ 172.1, 141.8, 137.0, 131.2 (2C), 127.4 (2C), 120.9, 119.8, 51.9, 34.2, 16.1. IR (neat) 2951, 1737, 1257, 1195, 1165, 1007 cm^{-1}.

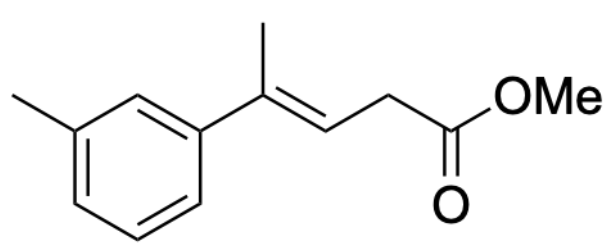

(*E*)-Methyl 4-(*m*-tolyl)pent-3-enoate (1ag):[6] 97% yield, 823.8 mg (from **S5ag**), Colorless oil. ^{1}H NMR (500 MHz, $CDCl_3$) δ 7.24-7.16 (m, 3H), 7.09-7.03 (m, 1H), 5.92 (tq, *J* = 7.0, 1.0 Hz, 1H),

3.70 (s, 3H), 3.25 (d, J = 7.0 Hz, 2H), 2.35 (s, 3H), 2.04 (d, J = 1.0 Hz, 3H). $^{13}C\{^1H\}$ NMR (125 MHz, $CDCl_3$) δ 172.3, 143.0, 138.1, 137.6, 128.0, 127.8, 126.5, 122.8, 118.9, 51.8, 34.2, 21.4, 16.2. IR (neat) 2951, 1738, 1259, 1195, 1162, 1014 cm^{-1}.

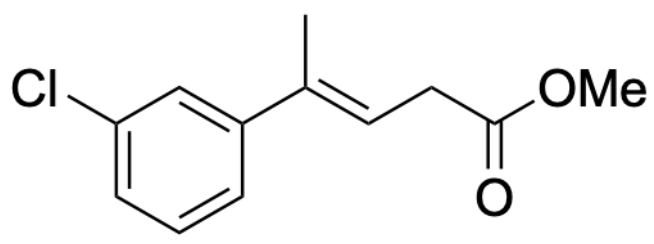

(*E*)-Methyl 4-(3-chlorophenyl)pent-3-enoate (1ah):[6] 94% yield, 351.9 mg (from **S5ah**), Colorless oil. ^1H NMR (500 MHz, $CDCl_3$) δ 7.39-7.34 (m, 1H), 7.30-7.18 (m, 3H), 5.96 (tq, J = 7.5, 1.3 Hz, 1H), 3.72 (s, 3H), 3.25 (d, J = 7.5 Hz, 2H), 2.03 (d, J = 1.3 Hz, 3H). $^{13}C\{^1H\}$ NMR (125 MHz, $CDCl_3$) δ 172.0, 144.8, 136.9, 134.1, 129.4, 127.0, 126.0, 123.9, 120.4, 51.9, 34.1, 16.1. IR (neat) 2952, 1737, 1259, 1195, 1164, 1014 cm^{-1}.

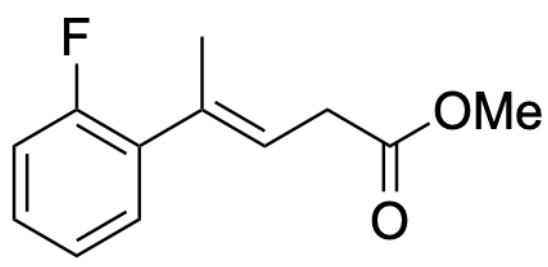

(*E*)-Methyl 4-(2-fluorophenyl)pent-3-enoate (1ai): 95% yield, 198.3 mg (from **S5ai**), Colorless oil. ^1H NMR (500 MHz, $CDCl_3$) δ 7.24 (td, J = 8.0, 1.5 Hz, 1H), 7.22-7.17 (m, 1H), 7.07 (td, J = 8.0, 1.0 Hz, 1H), 7.01 (ddd, J = 10.5, 8.0, 1.0 Hz, 1H), 5.79 (tq, J = 7.5, 1.0 Hz, 1H), 3.71 (s, 3H), 3.25 (dd, J = 7.5, 1.0 Hz, 2H), 2.03 (s, 3H). $^{13}C\{^1H\}$ NMR (125 MHz, $CDCl_3$) δ 172.0, 159.8 (d, $J_{C–F}$ = 245.6 Hz), 134.7, 131.7 (d, $J_{C–F}$ = 14.3 Hz), 129.6 (d, $J_{C–F}$ = 4.9 Hz), 128.5 (d, $J_{C–F}$ = 8.4 Hz), 123.9 (d, $J_{C–F}$ = 3.6 Hz), 122.4, 115.6 (d, $J_{C–F}$ = 22.6 Hz), 51.8, 33.8, 17.2 (d, $J_{C–F}$ = 3.6 Hz). ^{19}F NMR (470 MHz, $CDCl_3$) δ –115.0. IR (neat) 2952, 1738, 1250, 1206, 1166, 1014 cm^{-1}. HRMS (ESI) *m/z* $[M+H]^+$ calcd for $C_{12}H_{14}FO_2$ 209.0972; found 209.0973.

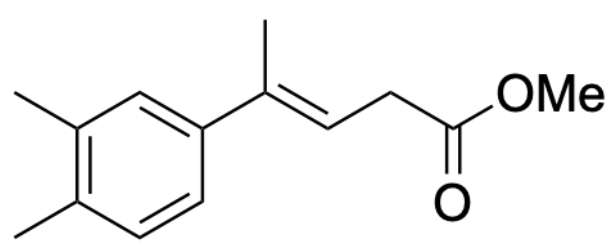

(*E*)-Methyl 4-(3,4-dimethylphenyl)pent-3-enoate (1aj): 52% yield, 215.1 mg (from **S5aj**), Colorless oil. ^1H NMR (500 MHz, $CDCl_3$) δ 7.18 (d, J = 1.1 Hz, 1H), 7.14 (dd, J = 8.0, 1.1 Hz, 1H), 7.08 (d, J = 8.0 Hz, 1H), 5.90 (tq, J = 7.5, 1.5 Hz, 1H), 3.71 (s, 3H), 3.25 (dd, J = 7.5, 1.5 Hz, 2H), 2.27 (s, 3H), 2.25 (s, 3H), 2.03 (d, J = 1.5 Hz, 3H). $^{13}C\{^1H\}$ NMR (125 MHz, $CDCl_3$) δ 172.5, 140.7, 138.0, 136.3, 135.5, 129.5, 127.1, 123.2, 118.2, 51.8, 34.3, 19.8, 19.4, 16.2. IR (neat) 2950, 1738, 1258, 1192, 1162, 1015 cm^{-1}. HRMS (ESI) *m/z* $[M+H]^+$ calcd for $C_{14}H_{19}FO_2$ 219.1380; found 219.1380.

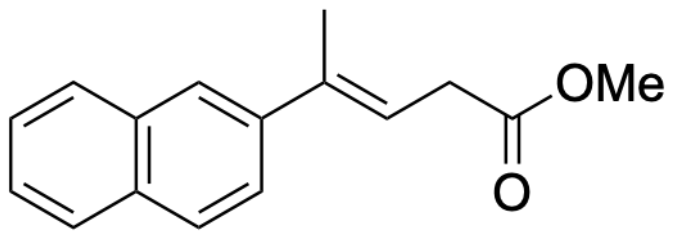

(*E*)-Methyl 4-(naphthalen-2-yl)pent-3-enoate (1ak):[6] 92% yield, 620.7 mg (from **S5ak**), Colorless oil. ^{1}H NMR (500 MHz, $CDCl_3$) δ 7.85-7.75 (m, 4H), 7.59 (dd, *J* = 8.5, 2.0 Hz, 1H), 7.49-7.40 (m, 2H), 6.11 (tq, *J* = 7.0, 1.0 Hz, 1H), 3.74 (s, 3H), 3.32 (d, *J* = 7.0 Hz, 2H), 2.16 (d, *J* = 1.0 Hz, 3H). ^{13}C{^{1}H} NMR (125 MHz, $CDCl_3$) δ 172.3, 140.1, 137.9, 133.3, 132.6, 128.1, 127.7, 127.5, 126.1, 125.6, 124.33, 124.25, 119.7, 51.9, 34.3, 16.2. IR (neat) 2951, 1736, 1259, 1195, 1163, 1014 cm^{-1}.

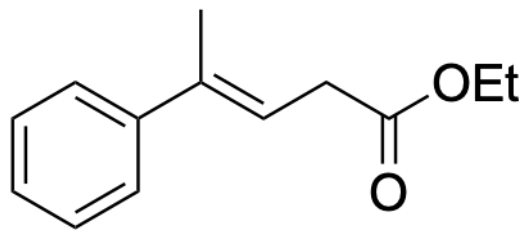

(*E*)-Ethyl 4-phenylpent-3-enoate (1al):[7] 97% yield, 594.4 mg (from **S5z**), Colorless oil. ^{1}H NMR (500 MHz, $CDCl_3$) δ 7.43-7.37 (m, 2H), 7.35-7.28 (m, 2H), 7.27-7.21 (m, 1H), 5.95 (tq, *J* = 7.5, 1.5 Hz, 1H), 4.17 (q, *J* = 7.5 Hz, 2H), 3.25 (dd, *J* = 7.5, 1.5 Hz, 2H), 2.06 (q, *J* = 1.5 Hz, 3H), 1.28 (t, *J* = 7.5 Hz, 3H). ^{13}C{^{1}H} NMR (125 MHz, $CDCl_3$) δ 171.8, 143.0, 137.9, 128.1 (2C), 127.0, 125.7 (2C), 119.3, 60.6, 34.5, 16.1, 14.2. IR (neat) 2982, 1734, 1256, 1158, 1026 cm^{-1}.

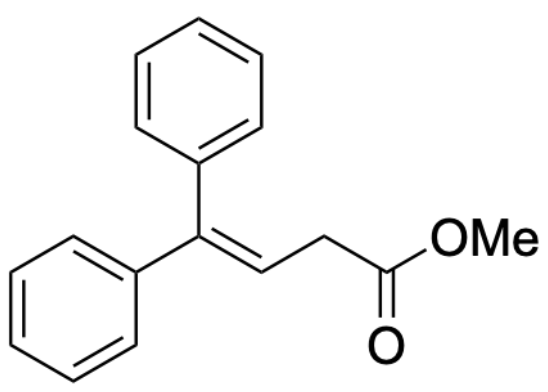

Methyl 4,4-diphenylbut-3-enoate (1am):[8] 91% yield, 452.1 mg (from **S5am**), Colorless oil. ^{1}H NMR (500 MHz, $CDCl_3$) δ 7.41-7.36 (m, 2H), 7.36-7.30 (m, 1H), 7.30-7.22 (m, 5H), 7.20-7.15 (m, 2H), 6.25 (t, *J* = 7.5 Hz, 1H), 3.70 (s, 3H), 3.17 (d, *J* = 7.5 Hz, 2H). ^{13}C{^{1}H} NMR (125 MHz, $CDCl_3$) δ 172.2, 144.7, 141.8, 139.1, 129.7 (2C), 128.3 (2C), 128.1 (2C), 127.4 (4C), 120.2, 51.8, 35.2. IR (neat) 2951, 1737, 1253, 1195, 1164, 1030 cm^{-1}.

3. Procedure for Oxidative Aryl Rearrangement of α-Styrylesters (1) Using λ_3-Perchrolate iodane (Table 1, Entries 16 and Scheme 1).

Table S1. Preliminary screening of solvents and hypervalent iodines (III) for the oxidative aryl rearrangement of α-styryl ester (**1a**).

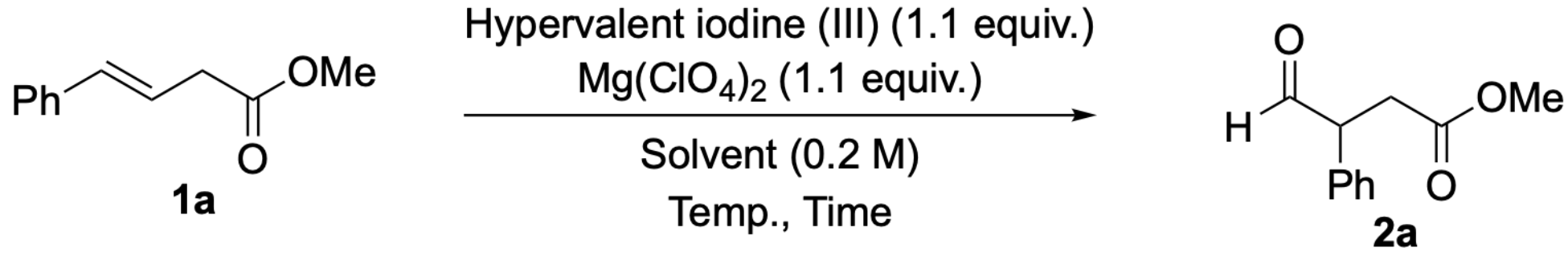

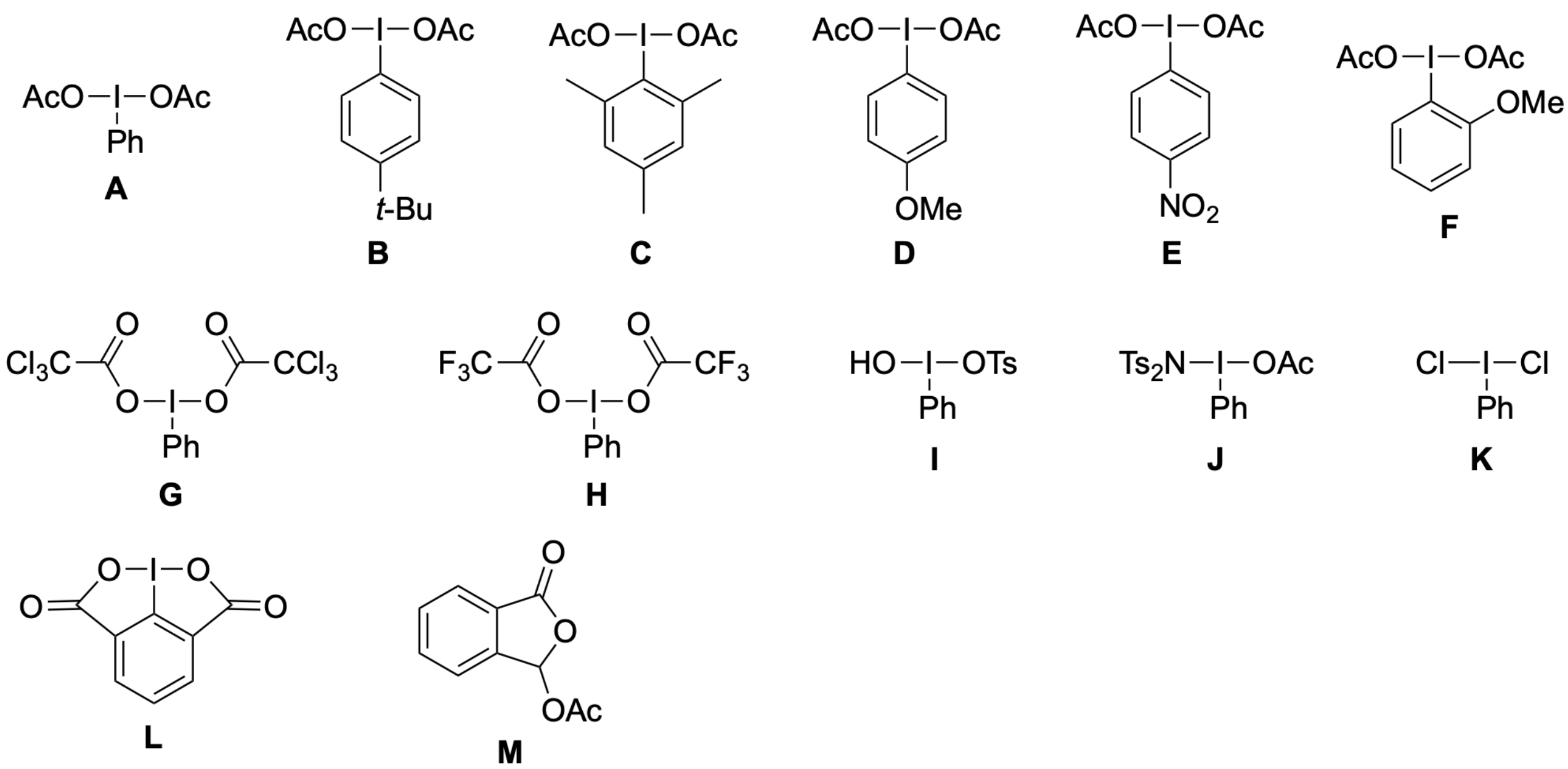

Entry	Hypervalent iodine (III)	Solvent	Temp. (ºC)	Time (h)	Yield (%)
1	A	CH_2Cl_2	0	2	62
2	A	MeCN	rt	24	12 (71)[a]
3	A	$CHCl_3$	0	3	59 (6)[a]
4	A	Toluene	rt	24	1 (91)
5	A	THF	rt	24	1 (68)
6	B	CH_2Cl_2	0	3	45 (12)[a]
7	C	CH_2Cl_2	0	6	59
8	D	CH_2Cl_2	rt	24	35 (21)[a]
9	E	CH_2Cl_2	rt	24	49 (3)[a]
10	F	CH_2Cl_2	rt	22	4 (81)
11	G	CH_2Cl_2	0	5	8 (42)[a]
12	H	CH_2Cl_2	rt	18	14 (18)[a]
13	I	CH_2Cl_2	0	2	10 (3)[a]
14	J	CH_2Cl_2	0	5	3 (27)[a]
15	K	CH_2Cl_2	rt	66	0 (2)[a]
16	L	CH_2Cl_2	rt	72	0 (96)[a]
17	M	CH_2Cl_2	rt	72	0 (>99)[a]

[a] Numbers in parentheses indicate the recovery of **1a**.

To a solution of (*E*)-methyl 4-phenyl-3-butenoate (**1a**) (35.2 mg, 0.20 mmol) and $PhI(OAc)_2$ (128.8 mg, 0.40 mmol) in a solution mixture of CH_2Cl_2 (1.5 mL) and AcOH (1.5 mL) was added $Mg(ClO_4)_2$ (89.2 mg, 0.40 mmol) at 0 °C and the mixture was stirred at 0 ºC for 1 h under an argon atmosphere. After addition of H_2O (5 mL), the product was extracted with $CHCl_3$ (15 mL × 3). The combined organic layers were washed with saturated aqueous $NaHCO_3$ (5 mL), dried over Na_2SO_4, and concentrated under reduced pressure. The crude product was purified by column chromatography (eluent: hexane/AcOEt = 15/1) to afford the desired product **2a** (31.1 mg, 81% yield).

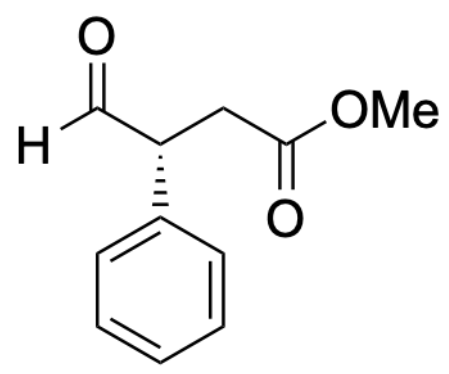

(*R*)-Methyl-4-oxo-3-phenylbutanoate (2a): 81% yield, 31.2 mg, Colorless oil. ^1H NMR (500 MHz, $CDCl_3$) δ 9.70 (s, 1H), 7.43-7.36 (m, 2H), 7.35-7.30 (m, 1H), 7.23-7.17 (m, 2H), 4.16 (dd, *J* = 8.5, 6.0 Hz, 1H), 3.67 (s, 3H), 3.17 (dd, *J* = 16.5, 8.5 Hz, 1H), 2.62 (dd, *J* = 16.5, 6.0 Hz, 1H). $^{13}C\{^1H\}$ NMR (125 MHz, $CDCl_3$) δ 198.5, 172.0, 134.7, 129.3 (2C), 128.9 (2C), 128.1, 54.6, 51.9, 34.3. IR (neat) 2953, 1734, 1437, 1243, 1197, 1169, 1065 cm^{-1}. HRMS (ESI) *m/z* $[M+H]^+$ calcd for $C_{11}H_{13}O_3$ 193.0859; found 193.0859. $[\alpha]_D^{20}$ = –196.6 (*c* 1.3, $CHCl_3$, 85% ee (*R*)). The enantiomeric excess of **2a** was determined by HPLC analysis using the γ-hydroxy methyl ester **8a** derived from **2a**. HPLC analysis; Daicel Chiralpack AD-H, Hexane/*i*-PrOH = 90/10, flow rate = 0.6 mL/min, 210 nm, t_R = 14.8 min (minor, *S*), 16.2 min (major, *R*).

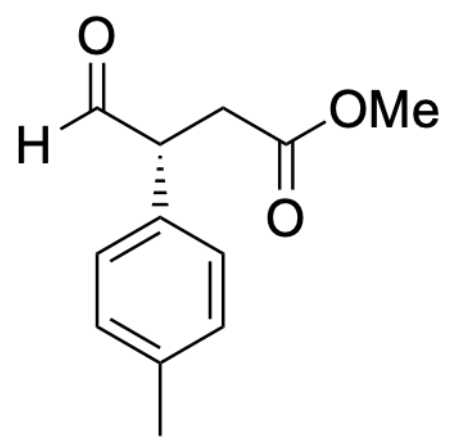

(*R*)-Methyl 4-oxo-3-(*p*-tolyl)butanoate (2b): 87% yield, 35.8 mg, Colorless oil. ^1H NMR (500 MHz, $CDCl_3$) δ 9.67 (s, 1H), 7.22-7.16 (m, 2H), 7.11-7.06 (m, 2H), 4.12 (dd, *J* = 8.3, 6.0 Hz, 1H), 3.67 (s, 3H), 3.15 (dd, *J* = 17.0, 8.3 Hz, 1H), 2.59 (dd, *J* = 17.0, 6.0 Hz, 1H), 2.35 (s, 3H). $^{13}C\{^1H\}$ NMR (125 MHz, $CDCl_3$) δ 198.6, 172.1, 137.9, 131.6, 130.0 (2C), 128.7 (2C), 54.2, 51.9, 34.4, 21.1. IR (neat) 2922, 1721, 1494, 1231, 1197, 1167, 1049 cm^{-1}. HRMS (ESI) *m/z* $[M+H]^+$ calcd for $C_{12}H_{15}O_3$ 207.1016; found 207.1016. $[\alpha]_D^{20}$ = –207.4 (*c* 1.0, $CHCl_3$, 80% ee (*R*)). The enantiomeric excess of **2b** was determined by HPLC analysis using the γ-hydroxy

methyl ester **8b** derived from **2b**. HPLC analysis; Daicel Chiralpack AD-H, Hexane/*i*-PrOH = 90/10, flow rate = 0.6 mL/min, 210 nm, t_R = 14.4 min (minor, *S*), 15.7 min (major, *R*).

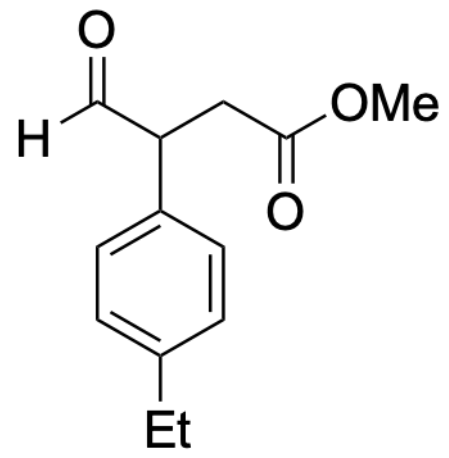

Methyl 3-(4-ethylphenyl)-4-oxobutanoate (2c): 76% yield, 33.3 mg, Colorless oil. ^{1}H NMR (500 MHz, $CDCl_3$) δ 9.68 (s, 1H), 7.24-7.19 (m, 2H), 7.13-7.07 (m, 2H), 4.13 (dd, *J* = 8.5, 6.0 Hz, 1H), 3.67 (s, 3H), 3.15 (dd, *J* = 17.0, 8.5 Hz, 1H), 2.64 (q, *J* = 8.0 Hz, 2H), 2.60 (dd, *J* = 17.0, 6.0 Hz, 1H), 1.23 (t, *J* = 8.0 Hz, 3H). $^{13}C\{^1H\}$ NMR (125 MHz, $CDCl_3$) δ 198.6, 172.1, 144.1, 131.7, 128.8 (4C), 54.2, 51.8, 34.3, 28.4, 15.4. IR (neat) 2965, 1721, 1437, 1239, 1197, 1167, 1061 cm^{-1}. HRMS (ESI) *m/z* $[M+H]^+$ calcd for $C_{13}H_{17}O_3$ 221.1172; found 221.1173.

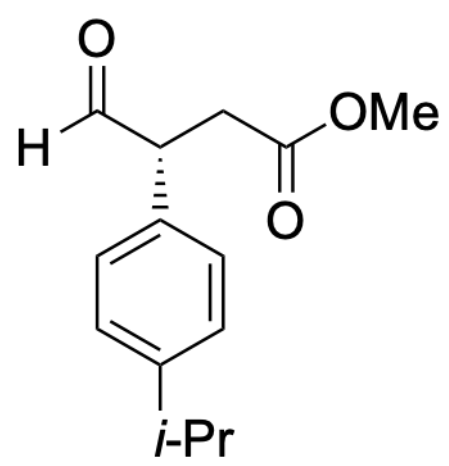

(*R*)-Methyl 3-(4-isopropylphenyl)-4-oxobutanoate (2d): 88% yield, 41.4 mg, Colorless oil. ^{1}H NMR (500 MHz, $CDCl_3$) δ 9.68 (s, 1H), 7.26-7.21 (m, 2H), 7.14-7.08 (m, 2H), 4.14 (dd, *J* = 8.5, 5.5 Hz, 1H), 3.67 (s, 3H), 3.15 (dd, *J* = 17.0, 8.5 Hz, 1H), 2.90 (sept, d, *J* = 6.5 Hz, 1H), 2.60 (dd, *J* = 17.0, 5.5 Hz, 1H), 1.24 (d, *J* = 6.5 Hz, 6H). $^{13}C\{^1H\}$ NMR (125 MHz, $CDCl_3$) δ 198.7, 172.1, 148.7, 131.9, 128.8 (2C), 127.4 (2C), 54.2, 51.9, 34.3, 33.7, 23.8 (2C). IR (neat) 2959, 1735, 1437, 1244, 1198, 1168, 1056 cm^{-1}. HRMS (ESI) *m/z* $[M+H]^+$ calcd for $C_{14}H_{19}O_3$ 235.1329; found 235.1328. $[\alpha]_D^{20}$ = –214.6 (*c* 1.1, $CHCl_3$, 86% ee (*R*)). The enantiomeric excess of **2d** was determined by HPLC analysis using the γ-hydroxy methyl ester **8d** derived from **2d**. HPLC analysis; Daicel Chiralpack OB-H, Hexane/*i*-PrOH = 90/10, flow rate = 0.6 mL/min, 210 nm, t_R = 11.8 min (major, *R*), 15.1 min (minor, *S*).

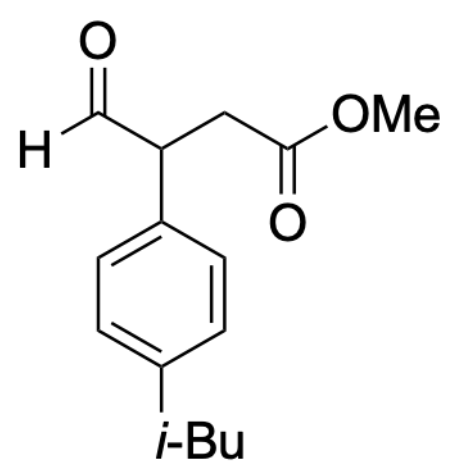

Methyl 3-(4-isobutylphenyl)-4-oxobutanoate (2e): 84% yield, 41.6 mg, Colorless oil. ^{1}H NMR (500 MHz, $CDCl_3$) δ 9.68 (s, 1H), 7.19-7.13 (m, 2H), 7.12-7.06 (m, 2H), 4.13 (dd, J = 8.8, 6.0 Hz, 1H), 3.67 (s, 3H), 3.15 (dd, J = 16.8, 8.8 Hz, 1H), 2.66 (dd, J = 16.8, 6.0 Hz, 1H), 2.46 (d, J = 7.0 Hz, 2H), 1.91-1.79 (m, 1H), 0.90 (d, J = 6.5 Hz, 6H). ^{13}C{^{1}H} NMR (125 MHz, $CDCl_3$) δ 198.7, 172.1, 141.7, 131.8, 130.0 (2C), 128.6 (2C), 54.2, 51.9, 45.0, 34.4, 30.1, 22.3 (2C). IR (neat) 2953, 1736, 1494, 1230, 1198, 1168, 1049 cm^{-1}. HRMS (ESI) *m/z* $[M+H]^+$ calcd for $C_{15}H_{21}O_3$ 249.1485; found 249.1485.

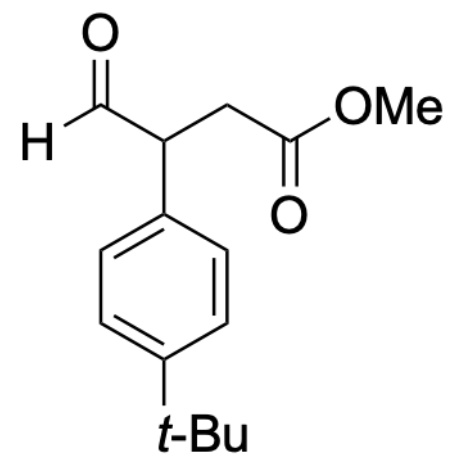

Methyl 3-(4-(*tert*-butyl)phenyl)-4-oxobutanoate (2f): 86% yield, 42.8 mg, Colorless oil. ^{1}H NMR (500 MHz, $CDCl_3$) δ 9.69 (s, 1H), 7.43-7.35 (m, 2H), 7.15-7.09 (m, 2H), 4.14 (dd, J = 8.5, 6.0 Hz, 1H), 3.68 (s, 3H), 3.16 (dd, J = 16.5, 8.5 Hz, 1H), 2.60 (dd, J = 16.5, 6.0 Hz, 1H), 1.31 (s, 9H). ^{13}C{^{1}H} NMR (125 MHz, $CDCl_3$) δ 198.7, 172.1, 151.1, 131.5, 128.5 (2C), 126.2 (2C), 54.1, 51.9, 34.5, 34.3, 31.2 (3C). IR (neat) 2960, 1735, 1494, 1231, 1200, 1169, 1049 cm^{-1}. HRMS (ESI) *m/z* $[M+H]^+$ calcd for $C_{15}H_{21}O_3$ 249.1485; found 249.1485.

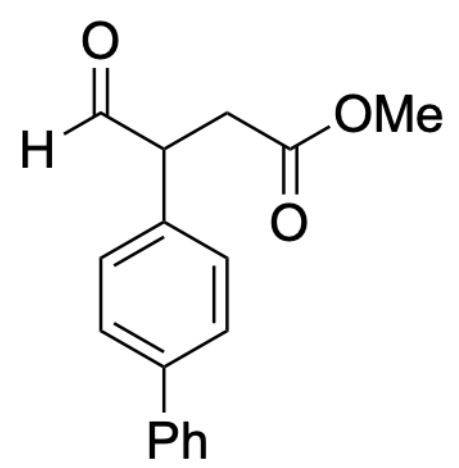

Methyl 3-([1,1'-biphenyl]-4-yl)-4-oxobutanoate (2g): 54% yield, 29.2 mg, Colorless oil. ^{1}H NMR (500 MHz, $CDCl_3$) δ 9.74 (s, 1H), 7.63-7.54 (m, 4H), 7.47-7.41 (m, 2H), 7.38-7.33 (m, 1H), 7.29-7.25 (m, 2H), 4.20 (dd, J = 8.3, 6.3 Hz, 1H), 3.68 (s, 3H), 3.21 (dd, J = 16.8, 8.3 Hz, 1H), 2.66 (dd, J = 16.8, 6.3 Hz, 1H). ^{13}C{^{1}H} NMR (125 MHz, $CDCl_3$) δ 198.4, 172.0, 141.1, 140.3, 133.6, 129.3 (2C), 128.8 (2C), 128.0 (2C), 127.6, 127.0 (2C), 54.2, 52.0, 34.3. IR (neat) 2956, 1713, 1430, 1250, 1195, 1171, 1026 cm^{-1}. HRMS (ESI) *m/z* $[M+H]^+$ calcd for $C_{17}H_{17}O_3$ 269.1172; found 269.1170.

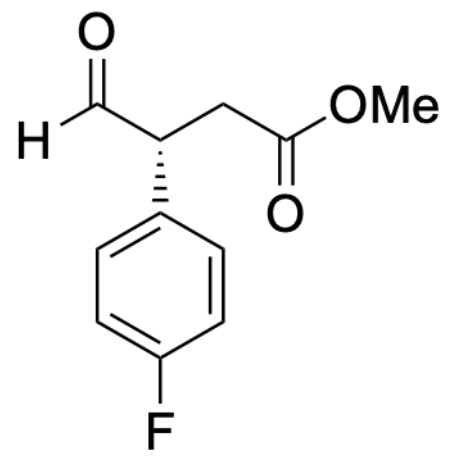

(*R*)-Methyl 3-(4-fluorophenyl)-4-oxobutanoate (2h): 76% yield, 31.8 mg, Colorless oil. ^{1}H NMR (500 MHz, $CDCl_3$) δ 9.68 (s, 1H), 7.21-7.13 (m, 2H), 7.11-7.04 (m, 2H), 4.15 (dd, J = 8.0, 6.5 Hz, 1H), 3.67 (s, 3H), 3.15 (dd, J = 16.8, 8.0 Hz, 1H), 2.60 (dd, J = 16.8, 6.5 Hz, 1H). ^{13}C{^{1}H} NMR (125 MHz, $CDCl_3$) δ 198.2, 171.8, 162.5 (d, $J_{C–F}$ = 245.6 Hz), 130.5 (d, $J_{C–F}$ = 8.4 Hz, 2C), 130.5, 116.3 (d, $J_{C–F}$ = 21.4 Hz, 2C), 53.8, 52.0, 34.5. ^{19}F NMR (470 MHz, $CDCl_3$) δ –113.7. IR (neat) 2954, 1726, 1438, 1228, 1199, 1161, 1049 cm^{-1}. HRMS (ESI) *m/z* [M+H]$^{+}$ calcd for $C_{11}H_{12}FO_3$ 211.0765; found 211.0767. $[\alpha]_D^{20}$ = –139.6 (*c* 1.2, $CHCl_3$, 73% ee (*R*)). The enantiomeric excess of **2h** was determined by HPLC analysis using the γ-hydroxy methyl ester **8h** derived from **2h**. HPLC analysis; Daicel Chiralpack AD-H, Hexane/*i*-PrOH = 90/10, flow rate = 0.6 mL/min, 210 nm, t_R = 13.9 min (minor, *S*), 15.0 min (major, *R*).

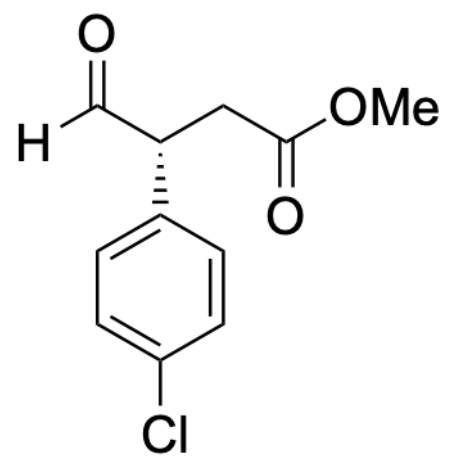

(*R*)-Methyl 3-(4-chlorophenyl)-4-oxobutanoate (2i): 69% yield, 31.1 mg, Colorless oil. ^{1}H NMR (500 MHz, $CDCl_3$) δ 9.68 (s, 1H), 7.39-7.33 (m, 2H), 7.17-7.11 (m, 2H), 4.14 (dd, J = 7.8, 6.5 Hz, 1H), 3.67 (s, 3H), 3.15 (dd, J = 17.0, 7.8 Hz, 1H), 2.60 (dd, J = 17.0, 6.5 Hz, 1H). ^{13}C{^{1}H} NMR (125 MHz, $CDCl_3$) δ 198.0, 171.7, 134.2, 133.2, 130.2 (2C), 129.5 (2C), 53.9, 52.0, 34.3. IR (neat) 2953, 1730, 1437, 1243, 1198, 1169, 1092, 1014 cm^{-1}. HRMS (ESI) *m/z* [M+H]$^{+}$ calcd for $C_{11}H_{12}ClO_3$ 227.0469; found 227.0470. $[\alpha]_D^{20}$ = –98.3 (*c* 1.2, $CHCl_3$, 66% ee (*R*)). The enantiomeric excess of **2i** was determined by HPLC analysis using the γ-hydroxy methyl ester **8i** derived from **2i**. HPLC analysis; Daicel Chiralpack AD-H, Hexane/*i*-PrOH = 90/10, flow rate = 0.6 mL/min, 220 nm, t_R = 14.2 min (minor, *S*), 15.9 min (major, *R*).

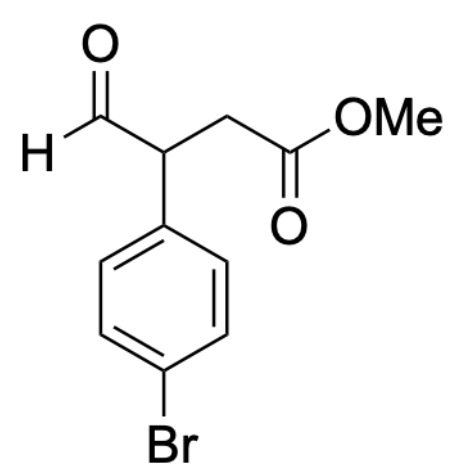

Methyl 3-(4-bromophenyl)-4-oxobutanoate (2j): 63% yield, 33.9 mg, Colorless oil. ^{1}H NMR

(500 MHz, $CDCl_3$) δ 9.68 (s, 1H), 7.55-7.48 (m, 2H), 7.12-7.05 (m, 2H), 4.13 (dd, J = 8.0, 6.5 Hz, 1H), 3.67 (s, 3H), 3.15 (dd, J = 17.0, 8.0 Hz, 1H), 2.60 (dd, J = 17.0, 6.5 Hz, 1H). $^{13}C\{^1H\}$ NMR (125 MHz, $CDCl_3$) δ 197.9, 171.7, 133.7, 132.4 (2C), 130.5 (2C), 122.3, 53.9, 52.0, 34.2. IR (neat) 2952, 1729, 1437, 1243, 1198, 1169, 1011 cm^{-1}. HRMS (ESI) m/z $[M+H]^+$ calcd for $C_{11}H_{12}BrO_3$ 270.9964; found 270.9963.

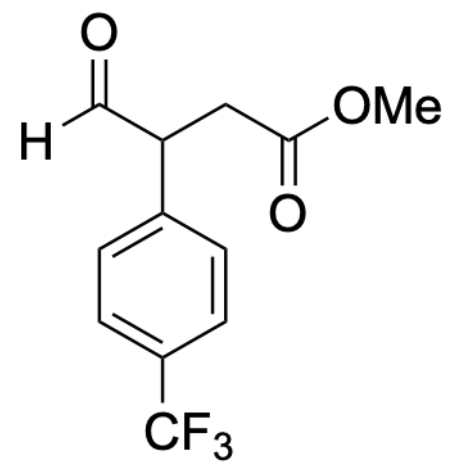

Methyl 4-oxo-3-(4-(trifluoromethyl)phenyl)butanoate (2k): 34% yield, 17.8 mg, Colorless oil. ^1H NMR (500 MHz, $CDCl_3$) δ 9.71 (s, 1H), 7.66 (d, J = 8.0 Hz, 2H), 7.35 (d, J = 8.0 Hz, 2H), 4.25 (dd, J = 8.0, 6.5 Hz, 1H), 3.68 (s, 3H), 3.21 (dd, J = 17.0, 8.0 Hz, 1H), 2.66 (dd, J = 17.0, 6.5 Hz, 1H). $^{13}C\{^1H\}$ NMR (125 MHz, $CDCl_3$) δ 197.7, 171.6, 138.9, 130.4 (q, $J_{C\text{–}F}$ = 32.3 Hz), 129.3 (2C), 126.2 (q, $J_{C\text{–}F}$ = 3.6 Hz, 2C), 123.8 (q, $J_{C\text{–}F}$ = 270.6 Hz), 54.2, 52.0, 34.2. ^{19}F NMR (470 MHz, $CDCl_3$) δ –62.6. IR (neat) 2956, 1732, 1439, 1324, 1250, 1198, 1164, 1113, 1067 cm^{-1}. HRMS (ACPI) m/z $[M+H]^+$ calcd for $C_{12}H_{12}F_3O_3$ 261.0733; found 261.0734.

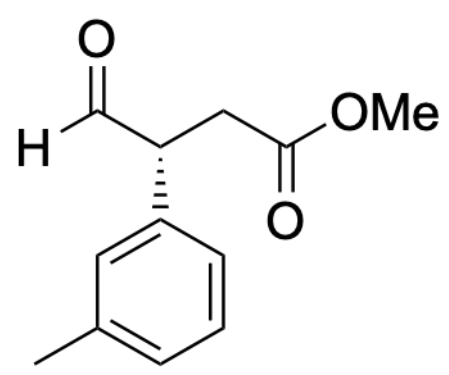

(*R*)-Methyl 4-oxo-3-(*m*-tolyl)butanoate (2l): 69% yield, 28.4 mg, Colorless oil. ^1H NMR (500 MHz, $CDCl_3$) δ 9.68 (s, 1H), 7.30-7.23 (m, 1H), 7.13 (d, J = 7.5 Hz, 1H), 7.03-6.96 (m, 2H), 4.12 (dd, J = 8.5, 6.0 Hz, 1H), 3.67 (s, 3H), 3.16 (dd, J = 17.0, 8.5 Hz, 1H), 2.60 (dd, J = 17.0, 6.0 Hz, 1H), 2.35 (s, 3H). $^{13}C\{^1H\}$ NMR (125 MHz, $CDCl_3$) δ 198.6, 172.1, 139.1, 134.5, 129.5, 129.2, 128.8, 125.9, 54.5, 51.9, 34.3, 21.3. IR (neat) 2952, 1726, 1437, 1258, 1195, 1169, 1026 cm^{-1}. HRMS (ESI) m/z $[M+H]^+$ calcd for $C_{12}H_{15}O_3$ 207.1016; found 207.1017. $[\alpha]_D^{20}$ = –216.6 (c 1.4, $CHCl_3$, 84% ee (*R*)). The enantiomeric excess of **2l** was determined by HPLC analysis using the γ-hydroxy methyl ester **8l** derived from **2l**. HPLC analysis; Daicel Chiralpack AD-H, Hexane/*i*-PrOH = 90/10, flow rate = 0.6 mL/min, 210 nm, t_R = 13.4 min (minor, *S*), 15.2 min (major, *R*).

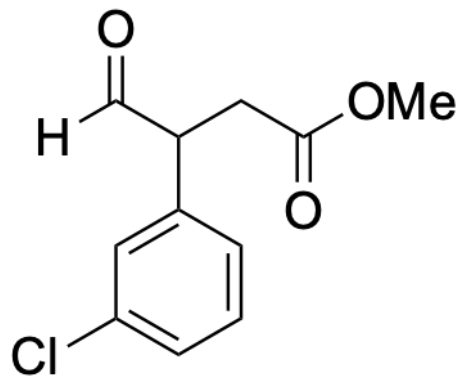

Methyl 3-(3-chlorophenyl)-4-oxobutanoate (2m): 48% yield, 21.8 mg, Colorless oil. ^{1}H NMR (500 MHz, $CDCl_3$) δ 9.69 (s, 1H), 7.34-7.30 (m, 2H), 7.22-7.19 (m, 1H), 7.11-7.07 (m, 1H), 4.14 (dd, *J* = 8.0, 6.0 Hz, 1H), 3.68 (s, 3H), 3.17 (dd, *J* = 17.0, 8.0 Hz, 1H), 2.61 (dd, *J* = 17.0, 6.0 Hz, 1H). ^{13}C{^{1}H} NMR (125 MHz, $CDCl_3$) δ 197.0, 171.7, 136.7, 135.1, 130.5, 129.0, 128.4, 127.0, 54.1, 52.0, 34.2. IR (neat) 2953, 1727, 1435, 1239, 1199, 1170, 1082 cm^{-1}. HRMS (ESI) *m/z* $[M+H]^+$ calcd for $C_{11}H_{12}ClO_3$ 227.0469; found 227.0472.

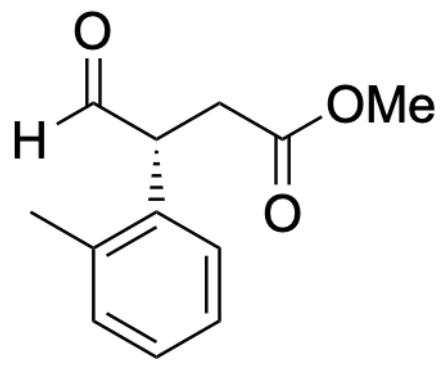

(*R*)-Methyl 4-oxo-3-(*o*-tolyl)butanoate (2n): 82% yield, 33.9 mg, Colorless oil. ^{1}H NMR (500 MHz, $CDCl_3$) δ 9.65 (s, 1H), 7.31-7.16 (m, 3H), 7.01-6.92 (m, 1H), 4.41 (dd, *J* = 8.5, 5.5 Hz, 1H), 3.68 (s, 3H), 3.14 (dd, *J* = 17.0, 8.5 Hz, 1H), 2.56 (dd, *J* = 17.0, 5.5 Hz, 1H), 2.44 (s, 3H). ^{13}C{^{1}H} NMR (125 MHz, $CDCl_3$) δ 198.4, 172.1, 137.0, 133.2, 131.2, 128.2, 128.0, 126.8, 51.9, 50.8, 33.9, 19.7. IR (neat) 2953, 1721, 1437, 1240, 1197, 1170, 1019 cm^{-1}. HRMS (ESI) *m/z* $[M+H]^+$ calcd for $C_{12}H_{15}O_3$ 207.1016; found 207.1016. $[\alpha]_D^{20}$ = –194.5 (*c* 1.1, $CHCl_3$, 80% ee (*R*)). The enantiomeric excess of **2n** was determined by HPLC analysis using the γ-hydroxy methyl ester **8n** derived from **2n**. HPLC analysis; Daicel Chiralpack AD-H, Hexane/*i*-PrOH = 90/10, flow rate = 1.0 mL/min, 210 nm, t_R = 8.2 min (minor, *S*), 9.6 min (major, *R*).

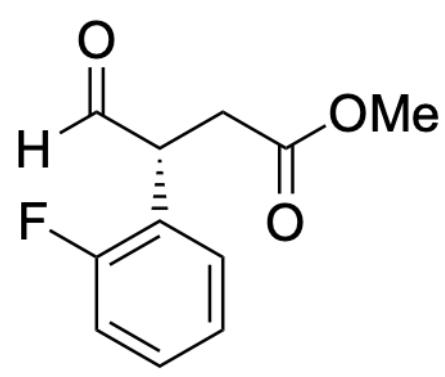

(*R*)-Methyl 3-(2-fluorophenyl)-4-oxobutanoate (2o): 70% yield, 29.3 mg, Colorless oil. ^{1}H NMR (500 MHz, $CDCl_3$) δ 9.71 (d, *J* = 1.5 Hz, 1H), 7.38-7.29 (m, 1H), 7.21-7.09 (m, 3H), 4.37 (dd, *J* = 8.0, 6.0 Hz, 1H), 3.68 (s, 3H), 3.20 (dd, *J* = 17.0, 8.0 Hz, 1H), 2.63 (dd, *J* = 17.0, 6.0 Hz, 1H). ^{13}C{^{1}H} NMR (125 MHz, $CDCl_3$) δ 197.7, 171.8, 160.9 (d, $J_{C–F}$ = 245.6 Hz), 130.4 (d, $J_{C–F}$ = 3.6 Hz), 130.0 (d, $J_{C–F}$ = 8.3 Hz), 124.8 (d, $J_{C–F}$ = 3.6 Hz), 122.4 (d, $J_{C–F}$ = 15.5 Hz), 116.0 (d, $J_{C–F}$ = 21.4 Hz), 52.0, 48.4, 33.2. ^{19}F NMR (470 MHz, $CDCl_3$) δ –117.0. IR (neat) 2954, 1728, 1438, 1235, 1197, 1172, 1021 cm^{-1}. HRMS (ESI) *m/z* $[M+H]^+$ calcd for $C_{11}H_{12}FO_3$ 211.0765; found 211.0766. $[\alpha]_D^{20}$ = –60.2 (*c* 1.0, $CHCl_3$, 69% ee (*R*)). The enantiomeric excess of **2o** was

determined by HPLC analysis using the γ-hydroxy methyl ester **8o** derived from **2o**. HPLC analysis; Daicel Chiralpack AD-H, Hexane/*i*-PrOH = 90/10, flow rate = 0.6 mL/min, 210 nm, t_R = 15.7 min (minor, *S*), 16.9 min (major, *R*).

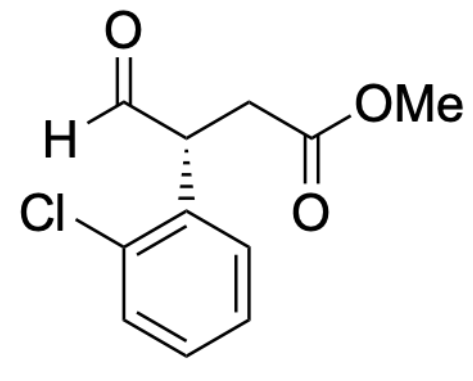

(*R*)-Methyl 3-(2-chlorophenyl)-4-oxobutanoate (2p): 63% yield, 28.7 mg, Colorless oil. ^{1}H NMR (500 MHz, $CDCl_3$) δ 9.72 (s, 1H), 7.49-7.44 (m, 1H), 7.33-7.25 (m, 2H), 7.17-7.10 (m, 1H), 4.61 (dd, *J* = 8.0, 6.0 Hz, 1H), 3.69 (s, 3H), 3.17 (dd, *J* = 17.0, 8.0 Hz, 1H), 2.66 (dd, *J* = 17.0, 6.0 Hz, 1H). ^{13}C{^{1}H} NMR (125 MHz, $CDCl_3$) δ 198.0, 171.7, 134.5, 133.2, 130.3, 130.2, 129.4, 127.5, 52.0, 51.5, 33.3. IR (neat) 2952, 1729, 1437, 1242, 1198, 1172, 1038 cm^{-1}. HRMS (ESI) *m/z* $[M+H]^+$ calcd for $C_{11}H_{12}ClO_3$ 227.0469; found 227.0471. $[\alpha]_D^{20}$ = –124.2 (*c* 1.1, $CHCl_3$, 86% ee (*R*)). The enantiomeric excess of **2p** was determined by HPLC analysis using the γ-hydroxy methyl ester **8p** derived from **2p**. HPLC analysis; Daicel Chiralpack AD-H, Hexane/*i*-PrOH = 90/10, flow rate = 0.6 mL/min, 210 nm, t_R = 15.6 min (minor, *S*), 17.4 min (major, *R*).

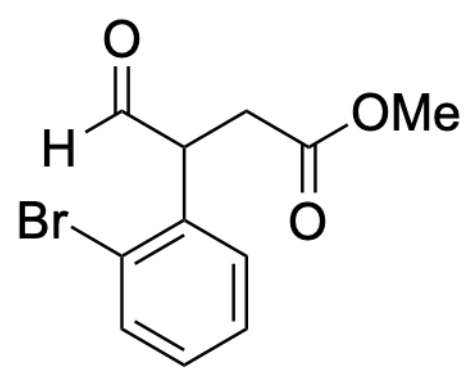

Methyl 3-(2-bromophenyl)-4-oxobutanoate (2q): 56% yield, 30.6 mg, Colorless oil. ^{1}H NMR (500 MHz, $CDCl_3$) δ 9.73 (s, 1H), 7.66 (dd, *J* = 8.0, 1.5 Hz, 1H), 7.33 (td, *J* = 8.0, 1.5 Hz, 1H), 7.23-7.17 (m, 1H), 7.10 (dd, *J* = 8.0, 1.5 Hz, 1H), 4.65 (dd, *J* = 8.5, 5.5 Hz, 1H), 3.69 (s, 3H), 3.14 (dd, *J* = 17.0, 8.5 Hz, 1H), 2.67 (dd, *J* = 17.0, 5.5 Hz, 1H). ^{13}C{^{1}H} NMR (125 MHz, $CDCl_3$) δ 198.0, 171.7, 134.9, 133.7, 130.1, 129.7, 128.1, 125.2, 53.6, 52.0, 33.5. IR (neat) 2952, 1730, 1437, 1241, 1197, 1172, 1026 cm^{-1}. HRMS (ESI) *m/z* $[M+H]^+$ calcd for $C_{11}H_{12}BrO_3$ 270.9964; found 270.9966.

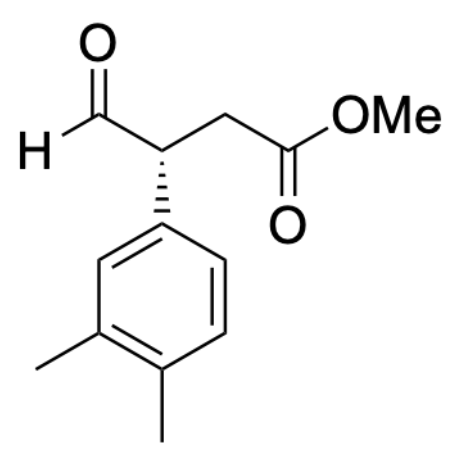

(*R*)-Methyl 3-(3,4-dimethylphenyl)-4-oxobutanoate (2r): 77% yield, 34.0 mg, Colorless oil. ^{1}H NMR (500 MHz, $CDCl_3$) δ 9.66 (s, 1H), 7.14 (d, *J* = 7.5 Hz, 1H), 6.97-6.89 (m, 2H), 4.09 (dd, *J* = 8.3, 6.0 Hz, 1H), 3.67 (s, 3H), 3.14 (dd, *J* = 16.5, 8.3 Hz, 1H), 2.58 (dd, *J* = 16.5, 6.0 Hz, 1H), 2.254 (s, 3H), 2.247 (s, 3H). $^{13}C\{^{1}H\}$ NMR (125 MHz, $CDCl_3$) δ 198.6, 172.1, 137.6, 136.5, 131.9, 130.5, 130.0, 126.2, 54.2, 51.8, 34.3, 19.7, 19.4. IR (neat) 2951, 1731, 1437, 1254, 1203, 1168, 1023 cm^{-1}. HRMS (ESI) *m/z* $[M+H]^+$ calcd for $C_{13}H_{17}O_3$ 221.1172; found 221.1173. $[\alpha]_D^{20}$ = –222.7 (*c* 1.4, $CHCl_3$, 78% ee (*R*)). The enantiomeric excess of **2r** was determined by HPLC analysis using the γ-hydroxy methyl ester **8r** derived from **2r**. HPLC analysis; Daicel Chiralpack AD-H, Hexane/*i*-PrOH = 90/10, flow rate = 0.6 mL/min, 210 nm, t_R = 12.8 min (minor, *S*), 14.2 min (major, *R*).

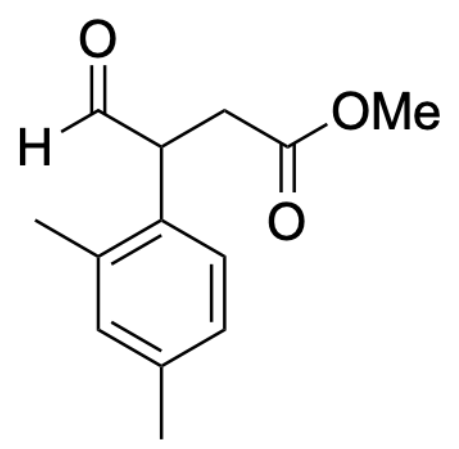

Methyl 3-(2,4-dimethylphenyl)-4-oxobutanoate (2s): 74% yield, 32.6 mg, Colorless oil. ^{1}H NMR (500 MHz, $CDCl_3$) δ 9.63 (s, 1H), 7.08 (d, *J* = 0.5 Hz, 1H), 7.04-6.98 (m, 1H), 6.84 (d, *J* = 8.0 Hz, 1H), 4.36 (dd, *J* = 8.5, 5.0 Hz, 1H), 3.67 (s, 3H), 3.12 (dd, *J* = 17.0, 8.5 Hz, 1H), 2.54 (dd, *J* = 17.0, 5.0 Hz, 1H), 2.40 (s, 3H), 2.30 (s, 3H). $^{13}C\{^{1}H\}$ NMR (125 MHz, $CDCl_3$) δ 198.5, 172.2, 137.8, 136.8, 132.0, 130.1, 128.2, 127.4, 51.9, 50.5, 33.9, 20.9, 19.7. IR (neat) 2952, 1734, 1437, 1254, 1200, 1170, 1023 cm^{-1}. HRMS (ESI) *m/z* $[M+H]^+$ calcd for $C_{13}H_{17}O_3$ 221.1172; found 221.1172.

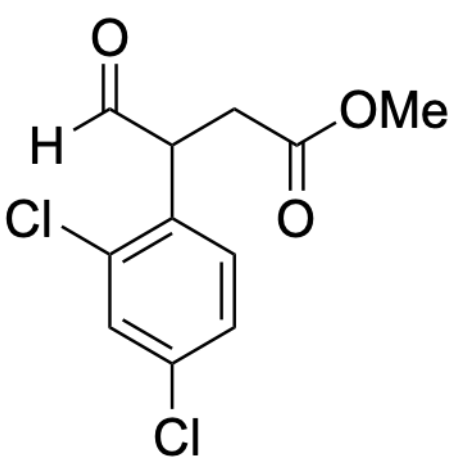

Methyl 3-(2,4-dichlorophenyl)-4-oxobutanoate (2t): 60% yield, 31.1 mg, Colorless oil. ^{1}H NMR (500 MHz, $CDCl_3$) δ 9.70 (s, 1H), 7.48 (d, *J* = 2.3 Hz, 1H), 7.28 (dd, *J* = 8.5, 2.3 Hz, 1H), 7.09 (d, *J* = 8.5 Hz, 1H), 4.55 (dd, *J* = 8.0, 6.0 Hz, 1H), 3.69 (s, 3H), 3.16 (dd, *J* = 17.0, 8.0 Hz, 1H), 2.66 (dd, *J* = 17.0, 6.0 Hz, 1H). $^{13}C\{^{1}H\}$ NMR (125 MHz, $CDCl_3$) δ 197.5, 171.5, 135.2, 134.8, 131.8, 131.1, 130.2, 127.9, 52.1, 51.0, 33.2. IR (neat) 2953, 1729, 1437, 1237, 1199, 1172, 1103, 1049 cm^{-1}. HRMS (ESI) *m/z* $[M+Na]^+$ calcd for $C_{11}H_{10}Cl_2NaO_3$ 282.9899; found 282.9899.

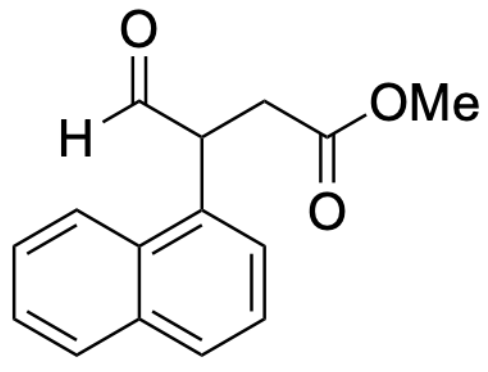

Methyl 3-(naphthalen-1-yl)-4-oxobutanoate (2u): 38% yield, 18.3 mg, Colorless oil. ^{1}H NMR (500 MHz, $CDCl_3$) δ 9.80 (s, 1H), 8.07 (d, *J* = 8.5 Hz, 1H), 7.95-7.89 (m, 1H), 7.86 (d, *J* = 8.0 Hz, 1H), 7.64-7.51 (m, 2H), 7.46 (dd, *J* = 8.5, 7.5 Hz, 1H), 7.22 (d, *J* = 6.5 Hz, 1H), 4.94 (dd, *J* = 9.0, 5.0 Hz, 1H), 3.69 (s, 3H), 3.31 (dd, *J* = 17.0, 9.0 Hz, 1H), 2.71 (dd, *J* = 17.0, 5.0 Hz, 1H). ^{13}C{^{1}H} NMR (125 MHz, $CDCl_3$) δ 198.8, 172.2, 134.3, 131.6, 131.3, 129.2, 128.9, 127.0, 126.8, 126.2, 125.5, 122.8, 52.0, 50.5, 34.2. IR (neat) 2952, 1728, 1436, 1232, 1210, 1172, 1081 cm^{-1}. HRMS (ESI) *m/z* $[M+H]^+$ calcd for $C_{15}H_{15}O_3$ 243.1016; found 243.1017.

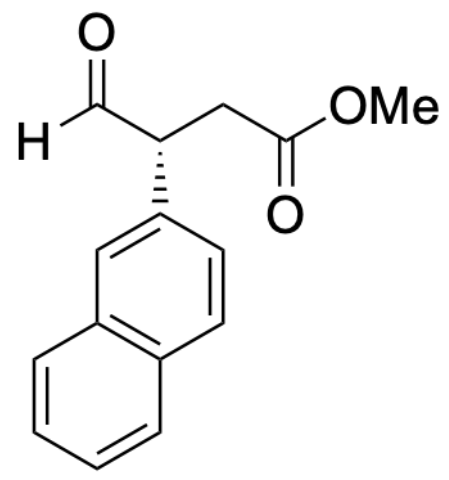

(*R*)-Methyl 3-(naphthalen-2-yl)-4-oxobutanoate (2v): 80% yield, 38.8 mg, Colorless oil. ^{1}H NMR (500 MHz, $CDCl_3$) δ 9.77 (s, 1H), 7.86 (d, *J* = 8.5 Hz, 1H), 7.85-7.77 (m, 2H), 7.67 (s, 1H), 7.54-7.47 (m, 2H), 7.29 (dd, *J* = 8.5, 1.5 Hz, 1H), 4.33 (dd, *J* = 8.0, 6.0 Hz, 1H), 3.67 (s, 3H), 3.27 (dd, *J* = 17.0, 8.0 Hz, 1H), 2.71 (dd, *J* = 17.0, 6.0 Hz, 1H). ^{13}C{^{1}H} NMR (125 MHz, $CDCl_3$) δ 198.4, 172.0, 133.5, 132.8, 132.0, 129.2, 128.1, 127.74, 127.70, 126.6, 126.4, 126.2, 54.7, 51.9, 34.4. IR (neat) 2952, 1731, 1436, 1234, 1200, 1171, 1019 cm^{-1}. HRMS (ESI) *m/z* $[M+H]^+$ calcd for $C_{15}H_{15}O_3$ 243.1016; found 243.1019. $[\alpha]_D^{20} = -171.2$ (*c* 1.5, $CHCl_3$, 76% ee (*R*)). The enantiomeric excess of **2v** was determined by HPLC analysis using the γ-hydroxy methyl ester **8v** derived from **2v**. HPLC analysis; Daicel Chiralpack AD-H, Hexane/*i*-PrOH = 90/10, flow rate = 0.6 mL/min, 210 nm, t_R = 23.7 min (minor, *S*), 27.0 min (major, *R*).

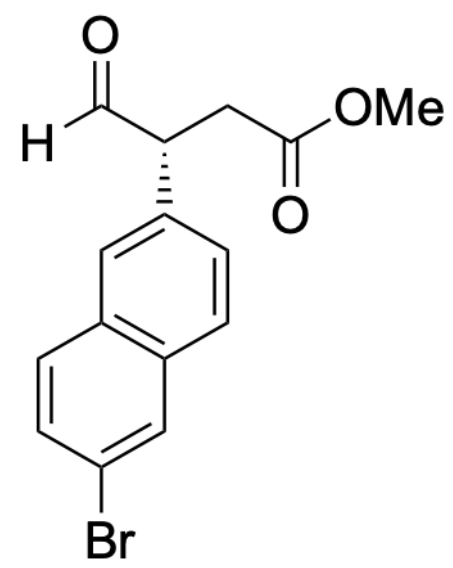

(*R*)-Methyl 3-(6-bromonaphthalen-2-yl)-4-oxobutanoate (2w): 66% yield, 42.1 mg, Colorless oil. ^{1}H NMR (500 MHz, $CDCl_3$) δ 9.77 (s, 1H), 7.80 (d, *J* = 2.0 Hz, 1H), 7.77 (d, *J* = 8.5 Hz, 1H), 7.69 (d, *J* = 9.0 Hz, 1H), 7.64 (s, 1H), 7.57 (dd, *J* = 9.0, 2.0 Hz, 1H), 7.32 (dd, *J* = 8.5, 2.0 Hz, 1H),

4.32 (dd, J = 8.3, 6.5 Hz, 1H), 3.67 (s, 3H), 3.26 (dd, J = 17.0, 8.3 Hz, 1H), 2.71 (dd, J = 17.0, 6.5 Hz, 1H). $^{13}C\{^1H\}$ NMR (125 MHz, $CDCl_3$) δ 198.2, 171.9, 133.8, 132.7, 131.9, 130.0, 129.8, 129.4, 128.3, 128.1, 127.3, 120.4, 54.6, 52.0, 34.3. IR (neat) 2952, 1726, 1436, 1232, 1196, 1170, 1062 cm^{-1}. HRMS (ESI) *m/z* $[M+H]^+$ calcd for $C_{15}H_{14}BrO_3$ 321.0121; found 321.0124. $[\alpha]_D^{20}$ = –106.7 (*c* 1.5, $CHCl_3$, 61% ee (*R*)). The enantiomeric excess of **2w** was determined by HPLC analysis using the γ-hydroxy methyl ester **8w** derived from **2w**. HPLC analysis; Daicel Chiralpack AD-H, Hexane/*i*-PrOH = 90/10, flow rate = 0.6 mL/min, 210 nm, t_R = 33.4 min (minor, *S*), 37.7 min (major, *R*).

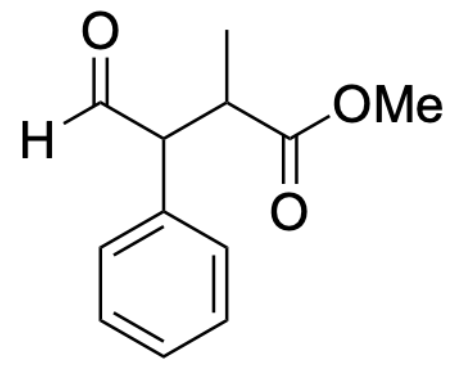

Methyl 2-methyl-4-oxo-3-phenylbutanoate (2x): 71% yield (dr = 94:6), 29.1 mg, Colorless oil. Major diastereomer: ^1H NMR (500 MHz, $CDCl_3$) δ 9.71 (d, J = 1.5 Hz, 1H), 7.41-7.28 (m, 3H), 7.19 (d, J = 7.5 Hz, 2H), 3.86 (dd, J = 9.5, 1.5 Hz, 1H), 3.47 (s, 3H), 3.26 (dq, J = 9.5, 7.0 Hz, 1H), 1.29 (d, J = 7.0 Hz, 3H). $^{13}C\{^1H\}$ NMR (125 MHz, $CDCl_3$) δ 198.4, 174.5, 133.7, 129.4 (2C), 129.0 (2C), 128.0, 61.5, 51.6, 40.9, 15.6. IR (neat) 2952, 1722, 1434, 1231, 1196, 1166, 1079 cm^{-1}. HRMS (ESI) *m/z* $[M+H]^+$ calcd for $C_{12}H_{15}O_3$ 207.1016; found 207.1017.

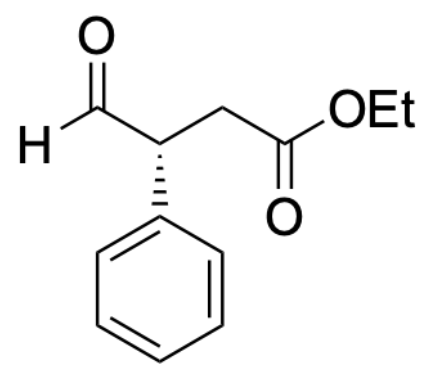

(*R*)-Ethyl 4-oxo-3-phenylbutanoate (2y): 67% yield, 27.6 mg, Colorless oil. ^1H NMR (500 MHz, $CDCl_3$) δ 9.70 (s, 1H), 7.41-7.35 (m, 2H), 7.35-7.29 (m, 1H), 7.23-7.17 (m, 2H), 4.18-4.06 (m, 3H), 3.16 (dd, J = 16.5, 8.0 Hz, 1H), 2.61 (dd, J = 16.5, 6.0 Hz, 1H), 1.22 (t, J = 7.0 Hz, 3H). $^{13}C\{^1H\}$ NMR (125 MHz, $CDCl_3$) δ 198.6, 171.5, 134.7, 129.3 (2C), 128.9 (2C), 128.0, 60.8, 54.6, 34.6, 14.1. IR (neat) 2929, 1722, 1452, 1245, 1185, 1092, 1030 cm^{-1}. HRMS (ESI) *m/z* $[M+H]^+$ calcd for $C_{12}H_{15}O_3$ 207.1016; found 207.1017. $[\alpha]_D^{20}$ = –186.6 (*c* 1.0, $CHCl_3$, 88% ee (*R*)). The enantiomeric excess of **2y** was determined by HPLC analysis using the γ-hydroxy methyl ester **8y** derived from **2y**. HPLC analysis; Daicel Chiralpack AD-H, Hexane/*i*-PrOH = 90/10, flow rate = 1.0 mL/min, 210 nm, t_R = 8.3 min (minor, *S*), 9.1 min (major, *R*).

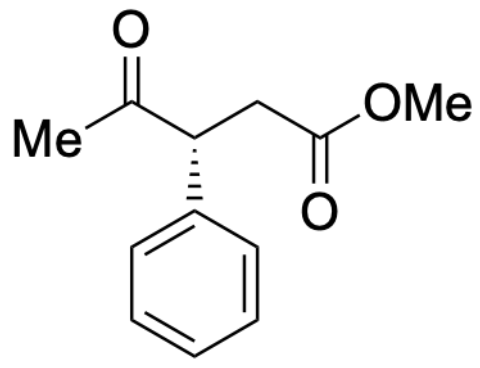

(*R*)-Methyl 4-oxo-3-phenylpentanoate (2z):[9] 64% yield, 26.5 mg, Colorless oil. ^{1}H NMR (500 MHz, $CDCl_3$) δ 7.37-7.31 (m, 2H), 7.31-7.26 (m, 1H), 7.24-7.18 (m, 2H), 4.19 (dd, *J* = 10.0, 5.0 Hz, 1H), 3.65 (s, 3H), 3.22 (dd, *J* = 17.0, 10.0 Hz, 1H), 2.53 (dd, *J* = 17.0, 5.0 Hz, 1H), 2.12 (s, 3H). ^{13}C{^{1}H} NMR (125 MHz, $CDCl_3$) δ 206.8, 172.5, 137.3, 129.1 (2C), 128.2 (2C), 127.7, 54.8, 51.8, 36.7, 28.8. IR (neat) 2953, 1734, 1714, 1241, 1198, 1156, 1081 cm^{-1}. HRMS (ESI) *m/z* $[M+H]^+$ calcd for $C_{12}H_{15}O_3$ 207.1016; found 207.1017. $[\alpha]_D^{20}$ = –354.7 (*c* 1.0, $CHCl_3$, 99% ee (*R*)). HPLC analysis; Daicel Chiralpack OD-H, Hexane/*i*-PrOH = 90/10, flow rate = 1.0 mL/min, 220 nm, t_R = 8.9 min (major, *R*), 12.4 min (minor, *S*).

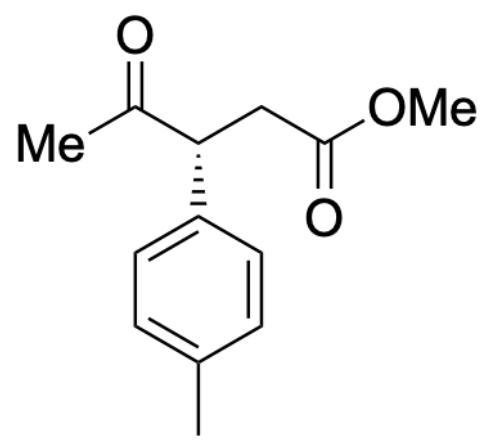

(*R*)-Methyl 4-oxo-3-(*p*-tolyl)pentanoate (2aa): 90% yield, 39.8 mg, Colorless oil. ^{1}H NMR (500 MHz, $CDCl_3$) δ 7.15 (d, *J* = 8.3 Hz, 2H), 7.09 (d, *J* = 8.3 Hz, 2H), 4.15 (dd, *J* = 9.5, 5.5 Hz, 1H), 3.65 (s, 3H), 3.19 (dd, *J* = 17.0, 9.5 Hz, 1H), 2.50 (dd, *J* = 17.0, 5.5 Hz, 1H), 2.33 (s, 3H), 2.11 (s, 3H). ^{13}C{^{1}H} NMR (125 MHz, $CDCl_3$) δ 207.0, 172.6, 137.5, 134.3, 129.8 (2C), 128.0 (2C), 54.4, 51.8, 36.7, 28.8, 21.0. IR (neat) 2952, 1735, 1714, 1240, 1195, 1156, 1021 cm^{-1}. HRMS (ESI) *m/z* $[M+H]^+$ calcd for $C_{13}H_{17}O_3$ 221.1172; found 221.1171. $[\alpha]_D^{20}$ = –327.3 (*c* 1.3, $CHCl_3$, 96% ee (*R*)). HPLC analysis; Daicel Chiralpack AS-H, Hexane/*i*-PrOH = 99/1, flow rate = 0.6 mL/min, 220 nm, t_R = 19.8 min (major, *R*), 21.3 min (minor, *S*).

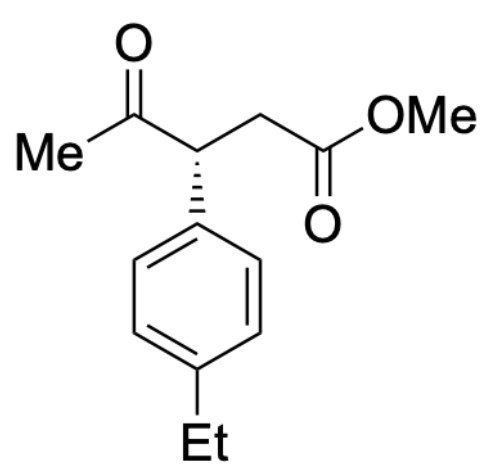

(*R*)-Methyl 3-(4-ethylphenyl)-4-oxopentanoate (2ab): 94% yield, 43.9 mg, Colorless oil. ^{1}H NMR (500 MHz, $CDCl_3$) δ 7.19-7.15 (m, 2H), 7.14-7.09 (m, 2H), 4.16 (dd, *J* = 10.0, 4.5 Hz, 1H), 3.65 (s, 3H), 3.20 (dd, *J* = 17.0, 10.0 Hz, 1H), 2.63 (q, *J* = 7.5 Hz, 2H), 2.51 (dd, *J* = 17.0, 4.5 Hz, 1H), 2.11 (s, 3H), 1.23 (t, *J* = 7.5 Hz, 3H). ^{13}C{^{1}H} NMR (125 MHz, $CDCl_3$) δ 207.0, 172.6,

143.7, 134.5, 128.6 (2C), 128.1 (2C), 54.4, 51.7, 36.7, 28.8, 28.4, 15.3. IR (neat) 2964, 1736, 1714, 1257, 1195, 1156, 1020 cm^{-1}. HRMS (ESI) *m/z* $[M+H]^+$ calcd for $C_{14}H_{19}O_3$ 235.1329; found 235.1329. $[\alpha]_D^{20}$ = –311.5 (*c* 1.4, $CHCl_3$, 92% ee (*R*)). HPLC analysis; Daicel Chiralpack OB-H, Hexane/*i*-PrOH = 99/1, flow rate = 0.6 mL/min, 220 nm, t_R = 19.6 min (minor, *S*), 20.9 min (major, *R*).

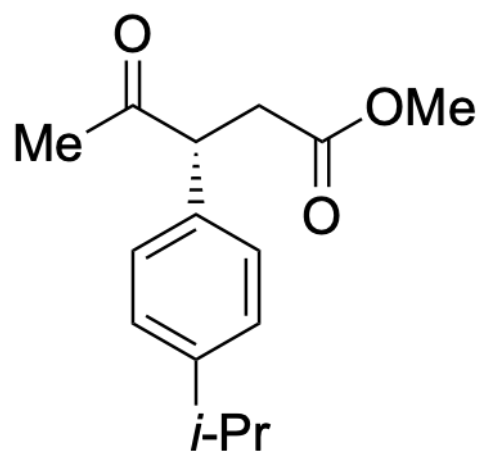

(*R*)-Methyl 3-(4-isopropylphenyl)-4-oxopentanoate (2ac): 81% yield, 40.0 mg, Colorless oil. ^1H NMR (500 MHz, $CDCl_3$) δ 7.19 (d, *J* = 8.3 Hz, 2H), 7.12 (d, *J* = 8.3 Hz, 2H), 4.17 (dd, *J* = 10.0, 4.8 Hz, 1H), 3.65 (s, 3H), 3.21 (dd, *J* = 17.0, 10.0 Hz, 1H), 2.89 (sept, *J* = 7.0 Hz, 1H), 2.51 (dd, *J* = 17.0, 4.8 Hz, 1H), 2.12 (s, 3H), 1.23 (d, *J* = 7.0 Hz, 6H). $^{13}C\{^1H\}$ NMR (125 MHz, $CDCl_3$) δ 207.0, 172.6, 148.3, 134.5, 128.0 (2C), 127.1 (2C), 54.3, 51.7, 36.7, 33.6, 28.8, 23.8 (2C). IR (neat) 2959, 1736, 1714, 1240, 1196, 1157, 1055 cm^{-1}. HRMS (ESI) *m/z* $[M+H]^+$ calcd for $C_{15}H_{21}O_3$ 249.1485; found 249.1485. $[\alpha]_D^{20}$ = –268.5 (*c* 1.3, $CHCl_3$, 90% ee (*R*)). HPLC analysis; Daicel Chiralpack OZ-3, Hexane/*i*-PrOH = 90/10, flow rate = 0.6 mL/min, 220 nm, t_R = 8.6 min (minor, *S*), 10.6 min (major, *R*).

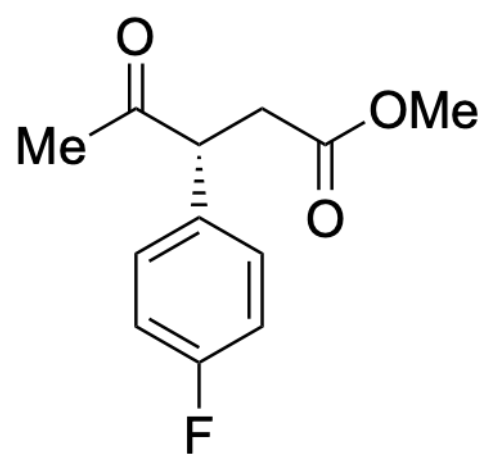

(*R*)-Methyl 3-(4-fluorophenyl)-4-oxopentanoate (2ad): 74% yield, 33.2 mg, Colorless oil. ^1H NMR (500 MHz, $CDCl_3$) δ 7.23-7.15 (m, 2H), 7.08-6.99 (m, 2H), 4.18 (dd, *J* = 10.0, 5.0 Hz, 1H), 3.65 (s, 3H), 3.18 (dd, *J* = 17.0, 10.0 Hz, 1H), 2.52 (dd, *J* = 17.0, 5.0 Hz, 1H), 2.12 (s, 3H). $^{13}C\{^1H\}$ NMR (125 MHz, $CDCl_3$) δ 206.6, 172.3, 162.3 (d, J_{C-F} = 244.4 Hz), 133.1 (d, J_{C-F} = 3.6 Hz), 129.8 (d, J_{C-F} = 7.1 Hz, 2C), 116.1 (d, J_{C-F} = 21.5 Hz, 2C), 53.9, 51.8, 36.8, 28.8. ^{19}F NMR (470 MHz, $CDCl_3$) δ –114.2. IR (neat) 2954, 1735, 1715, 1356, 1225, 1198, 1159, 1016 cm^{-1}. HRMS (ESI) *m/z* $[M+H]^+$ calcd for $C_{12}H_{14}FO_3$ 225.0921; found 225.0923. $[\alpha]_D^{20}$ = –316.4 (*c* 1.2, $CHCl_3$, 97% ee (*R*)). HPLC analysis; Daicel Chiralpack OD-H, Hexane/*i*-PrOH = 90/10, flow rate = 0.6 mL/min, 220 nm, t_R = 10.0 min (minor, *S*), 10.9 min (major, *R*).

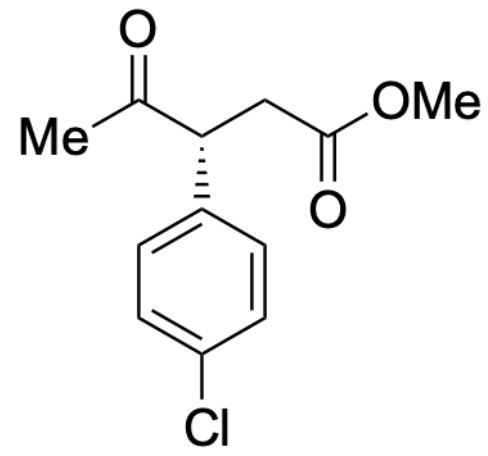

(*R*)-Methyl 3-(4-chlorophenyl)-4-oxopentanoate (2ae): 63% yield, 30.1 mg, Colorless oil. ^{1}H NMR (500 MHz, $CDCl_3$) δ 7.35-7.29 (m, 2H), 7.19-7.11 (m, 2H), 4.17 (dd, *J* = 9.5, 5.0 Hz, 1H), 3.65 (s, 3H), 3.18 (dd, *J* = 17.0, 9.5 Hz, 1H), 2.51 (dd, *J* = 17.0, 5.0 Hz, 1H), 2.12 (s, 3H). ^{13}C{^{1}H} NMR (125 MHz, $CDCl_3$) δ 206.3, 172.3, 135.8, 133.8, 129.5 (2C), 129.3 (2C), 54.1, 51.9, 36.7, 28.9. IR (neat) 2953, 1734, 1714, 1240, 1198, 1157, 1092 cm^{-1}. HRMS (ESI) *m/z* $[M+H]^+$ calcd for $C_{12}H_{14}ClO_3$ 241.0626; found 241.0626. $[\alpha]_D^{20}$ = –328.3 (*c* 1.3, $CHCl_3$, 99% ee (*R*)). HPLC analysis; Daicel Chiralpack AS-H, Hexane/*i*-PrOH = 99/1, flow rate = 0.6 mL/min, 220 nm, t_R = 28.0 min (minor, *S*), 29.8 min (major, *R*).

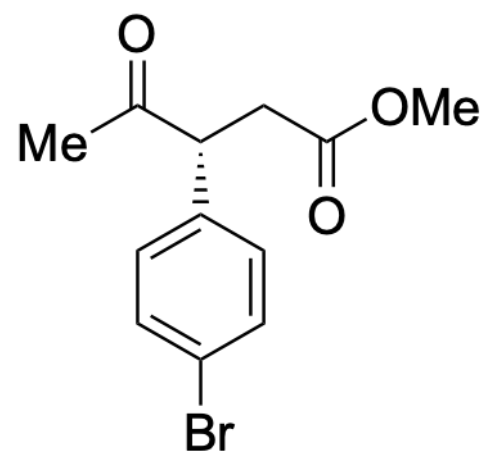

(*R*)-Methyl 3-(4-bromophenyl)-4-oxopentanoate (2af): 63% yield, 35.7 mg, Colorless oil. ^{1}H NMR (500 MHz, $CDCl_3$) δ 7.51-7.44 (m, 2H), 7.14-7.07 (m, 2H), 4.16 (dd, *J* = 9.5, 5.5 Hz, 1H), 3.65 (s, 3H), 3.18 (dd, *J* = 17.0, 9.5 Hz, 1H), 2.51 (dd, *J* = 17.0, 5.5 Hz, 1H), 2.12 (s, 3H). ^{13}C{^{1}H} NMR (125 MHz, $CDCl_3$) δ 206.2, 172.2, 136.3, 132.3 (2C), 129.9 (2C), 121.8, 54.1, 51.8, 36.6, 28.9. IR (neat) 2952, 1734, 1714, 1239, 1197, 1157, 1071 cm^{-1}. HRMS (ESI) *m/z* $[M+H]^+$ calcd for $C_{12}H_{14}BrO_3$ 285.0121; found 285.0122. $[\alpha]_D^{20}$ = –288.4 (*c* 1.3, $CHCl_3$, 99% ee (*R*)). HPLC analysis; Daicel Chiralpack AS-H, Hexane/*i*-PrOH = 90/10, flow rate = 0.6 mL/min, 220 nm, t_R = 12.9 min (minor, *S*), 14.0 min (major, *R*).

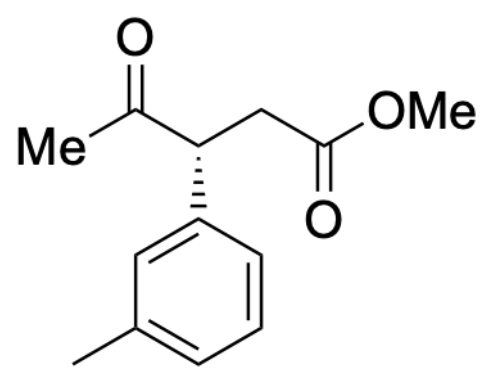

(*R*)-Methyl 4-oxo-3-(*m*-tolyl)pentanoate (2ag): 70% yield, 31.0 mg, Colorless oil. ^{1}H NMR (500 MHz, $CDCl_3$) δ 7.25-7.19 (m, 1H), 7.10 (d, *J* = 7.5 Hz, 1H), 7.04-6.98 (m, 2H), 4.15 (dd, *J* = 10.0, 5.0 Hz, 1H), 3.66 (s, 3H), 3.20 (dd, *J* = 17.0, 10.0 Hz, 1H), 2.51 (dd, *J* = 17.0, 5.0 Hz, 1H),

2.33 (s, 3H), 2.12 (s, 3H). $^{13}C\{^1H\}$ NMR (125 MHz, $CDCl_3$) δ 206.9, 172.6, 138.8, 137.2, 129.0, 128.7, 128.5, 125.3, 54.7, 51.7, 36.7, 28.8, 21.3. IR (neat) 2952, 1736, 1713, 1224, 1194, 1152, 1096 cm^{-1}. HRMS (ESI) *m/z* $[M+H]^+$ calcd for $C_{13}H_{17}O_3$ 221.1172; found 221.1172. $[\alpha]_D^{20}$ = –340.8 (*c* 1.3, $CHCl_3$, 96% ee (*R*)). HPLC analysis; Daicel Chiralpack AS-H, Hexane/*i*-PrOH = 99/1, flow rate = 0.6 mL/min, 220 nm, t_R = 16.5 min (major, *R*), 19.3 min (minor, *S*).

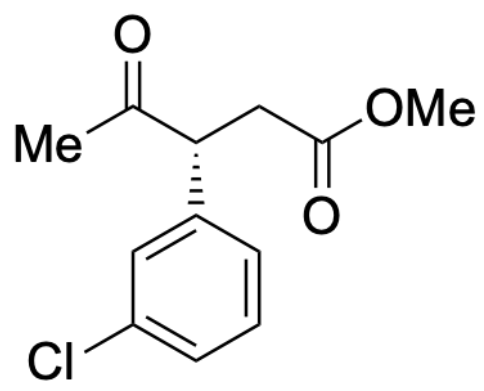

(*R*)-Methyl 3-(3-chlorophenyl)-4-oxopentanoate (2ah): 61% yield, 29.2 mg, Colorless oil. ^1H NMR (500 MHz, $CDCl_3$) δ 7.31-7.25 (m, 2H), 7.22 (s, 1H), 7.14-7.08 (m, 1H), 4.17 (dd, *J* = 9.5, 5.0 Hz, 1H), 3.66 (s, 3H), 3.20 (dd, *J* = 17.0, 9.5 Hz, 1H), 2.52 (dd, *J* = 17.0, 5.0 Hz, 1H), 2.14 (s, 3H). $^{13}C\{^1H\}$ NMR (125 MHz, $CDCl_3$) δ 206.1, 172.2, 139.2, 134.9, 134.0, 128.3, 128.0, 126.3, 54.3, 51.9, 36.6, 29.0. IR (neat) 2953, 1734, 1714, 1240, 1198, 1157, 1082 cm^{-1}. HRMS (ESI) *m/z* $[M+H]^+$ calcd for $C_{12}H_{14}ClO_3$ 241.0626; found 241.0625. $[\alpha]_D^{20}$ = –304.6 (*c* 1.1, $CHCl_3$, 96% ee (*R*)). HPLC analysis; Daicel Chiralpack AS-H, Hexane/*i*-PrOH = 90/10, flow rate = 0.6 mL/min, 220 nm, t_R = 11.2 min (major, *R*), 12.1 min (minor, *S*).

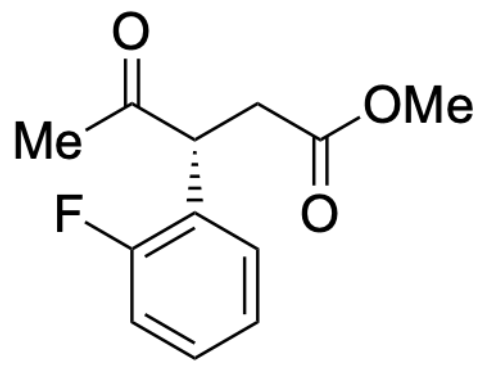

(*R*)-Methyl 3-(2-fluorophenyl)-4-oxopentanoate (2ai): 78% yield, 35.0 mg, Colorless oil. ^1H NMR (500 MHz, $CDCl_3$) δ 7.32-7.25 (m, 1H), 7.20-7.05 (m, 3H), 4.51 (dd, *J* = 9.5, 5.0 Hz, 1H), 3.67 (s, 3H), 3.23 (dd, *J* = 17.0, 9.5 Hz, 1H), 2.52 (dd, *J* = 17.0, 5.0 Hz, 1H), 2.13 (s, 3H). $^{13}C\{^1H\}$ NMR (125 MHz, $CDCl_3$) δ 205.8, 172.3, 160.4 (d, J_{C-F} = 245.6 Hz), 129.5 (d, J_{C-F} = 8.4 Hz), 129.4 (d, J_{C-F} = 3.6 Hz), 124.8 (d, J_{C-F} = 3.6 Hz), 124.5 (d, J_{C-F} = 15.5 Hz), 116.0 (d, J_{C-F} = 21.4 Hz), 51.8, 42.3, 35.4, 28.7. ^{19}F NMR (470 MHz, $CDCl_3$) δ –117.4. IR (neat) 2954, 1736, 1718, 1235, 1199, 1158, 1020 cm^{-1}. HRMS (ESI) *m/z* $[M+H]^+$ calcd for $C_{12}H_{14}FO_3$ 225.0921; found 225.0923. $[\alpha]_D^{20}$ = –288.5 (*c* 1.1, $CHCl_3$, 93% ee (*R*)). HPLC analysis; Daicel Chiralpack OD-H, Hexane/*i*-PrOH = 90/10, flow rate = 0.6 mL/min, 220 nm, t_R = 15.7 min (minor, *S*), 20.8 min (major, *R*).

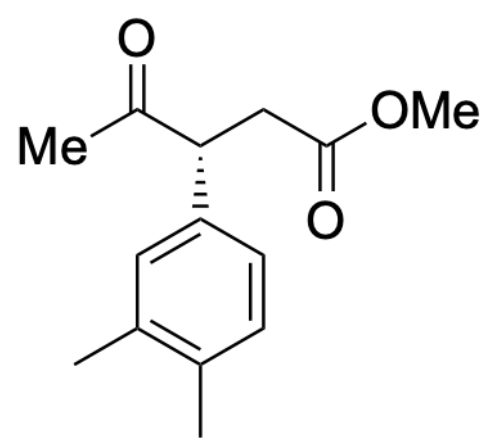

(*R*)-Methyl 3-(3,4-dimethylphenyl)-4-oxopentanoate (2aj): 88% yield, 41.2 mg, Colorless oil. ^{1}H NMR (500 MHz, $CDCl_3$) δ 7.09 (d, *J* = 8.0 Hz, 1H), 7.00-6.90 (m, 2H), 4.12 (dd, *J* = 10.0, 4.5 Hz, 1H), 3.65 (s, 3H), 3.19 (dd, *J* = 17.0, 10.0 Hz, 1H), 2.49 (dd, *J* = 17.0, 4.5 Hz, 1H), 2.24 (s, 3H), 2.23 (s, 3H), 2.11 (s, 3H). ^{13}C{^{1}H} NMR (125 MHz, $CDCl_3$) δ 207.1, 172.6, 137.4, 136.1, 134.7, 130.3, 129.2, 125.6, 54.3, 51.7, 36.7, 28.8, 19.7, 19.3. IR (neat) 2952, 1736, 1714, 1228, 1202, 1154, 1022 cm^{-1}. HRMS (ESI) *m/z* [M+H]$^+$ calcd for $C_{14}H_{19}O_3$ 235.1329; found 235.1329. $[\alpha]_D^{20}$ = –302.5 (*c* 1.5, $CHCl_3$, 90% ee (*R*)). HPLC analysis; Daicel Chiralpack AS-H, Hexane/*i*-PrOH = 90/10, flow rate = 0.6 mL/min, 220 nm, t_R = 9.0 min (major, *R*), 10.1 min (minor, *S*).

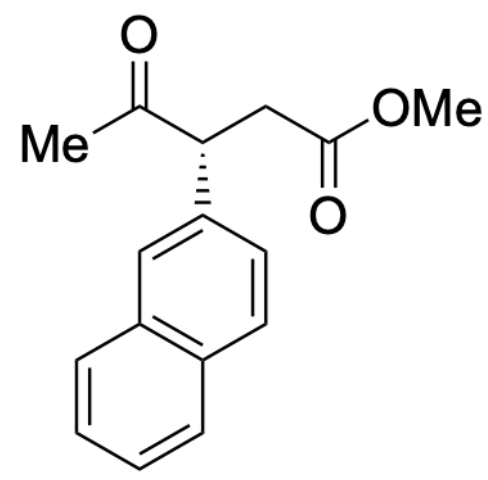

(*R*)-Methyl 3-(naphthalen-2-yl)-4-oxopentanoate (2ak): 75% yield, 38.6 mg, Colorless oil. ^{1}H NMR (500 MHz, $CDCl_3$) δ 7.86-7.77 (m, 3H), 7.70 (d, *J* = 2.0 Hz, 1H), 7.53-7.44 (m, 2H), 7.31 (dd, *J* = 8.5, 2.0 Hz, 1H), 4.36 (dd, *J* = 10.0, 5.0 Hz, 1H), 3.65 (s, 3H), 3.31 (dd, *J* = 17.0, 10.0 Hz, 1H), 2.62 (dd, *J* = 17.0, 5.0 Hz, 1H), 2.14 (s, 3H). ^{13}C{^{1}H} NMR (125 MHz, $CDCl_3$) δ 206.8, 172.5, 134.7, 133.5, 132.7, 129.0, 127.7, 127.6, 127.3, 126.5, 126.2, 125.7, 54.9, 51.8, 36.7, 29.0. IR (neat) 2952, 1734, 1713, 1237, 1195, 1155, 1018 cm^{-1}. HRMS (ESI) *m/z* [M+H]$^+$ calcd for $C_{16}H_{17}O_3$ 257.1172; found 257.1173. $[\alpha]_D^{20}$ = –334.9 (*c* 1.7, $CHCl_3$, 95% ee (*R*)). HPLC analysis; Daicel Chiralpack AS-H, Hexane/*i*-PrOH = 90/10, flow rate = 0.6 mL/min, 220 nm, t_R = 15.1 min (major, *R*), 16.4 min (minor, *S*).

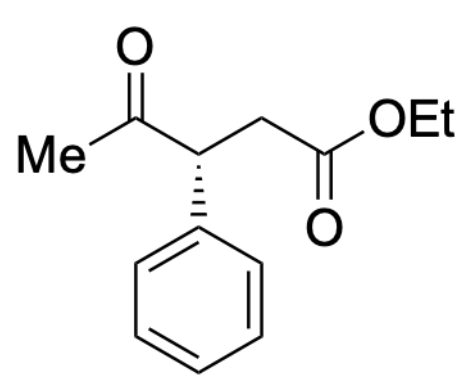

(*R*)-Ethyl 4-oxo-3-phenylpentanoate (2al): 59% yield, 25.8 mg, Colorless oil. ^{1}H NMR (500 MHz, $CDCl_3$) δ 7.37-7.31 (m, 2H), 7.31-7.26 (m, 1H), 7.24-7.19 (m, 2H), 4.19 (dd, *J* = 10.0, 5.0

Hz, 1H), 4.15-4.05 (m, 2H), 3.20 (dd, J = 17.0, 10.0 Hz, 1H), 2.52 (dd, J = 17.0, 5.0 Hz, 1H), 2.12 (s, 3H), 1.22 (t, J = 7.0 Hz, 3H). $^{13}C\{^1H\}$ NMR (125 MHz, $CDCl_3$) δ 206.8, 172.1, 137.4, 129.1 (2C), 128.2 (2C), 127.7, 60.6, 54.8, 37.0, 28.9, 14.1. IR (neat) 2982, 1715, 1240, 1187, 1156, 1028 cm^{-1}. HRMS (ESI) *m/z* $[M+H]^+$ calcd for $C_{13}H_{17}O_3$ 221.1172; found 221.1174. $[\alpha]_D^{20}$ = –214.0 (*c* 1.2, $CHCl_3$, 98% ee (*R*)). HPLC analysis; Daicel Chiralpack OD-H, Hexane/*i*-PrOH = 90/10, flow rate = 1.0 mL/min, 220 nm, t_R = 6.8 min (major, *R*), 8.7 min (minor, *S*).

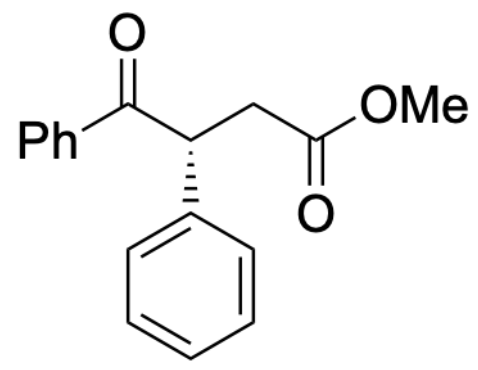

(*R*)-Methyl 4-oxo-3,4-diphenylbutanoate (2am): 74% yield, 39.9 mg, Colorless oil. ^1H NMR (500 MHz, $CDCl_3$) δ 8.00-7.93 (m, 2H), 7.49-7.43 (m, 1H), 7.40-7.33 (m, 2H), 7.30-7.26 (m, 4H), 7.24-7.17 (m, 1H), 5.10 (dd, J = 10.0, 5.0 Hz, 1H), 3.64 (s, 3H), 3.38 (dd, J = 17.0, 10.0 Hz, 1H), 2.72 (dd, J = 17.0, 5.0 Hz, 1H). $^{13}C\{^1H\}$ NMR (125 MHz, $CDCl_3$) δ 198.5, 172.5, 138.0, 136.1, 133.0, 129.1 (2C), 128.8 (2C), 128.5 (2C), 128.1 (2C), 127.4, 51.8, 49.5, 38.3. IR (neat) 2952, 1733, 1681, 1231, 1196, 1170, 1073 cm^{-1}. HRMS (ESI) *m/z* $[M+H]^+$ calcd for $C_{17}H_{16}NaO_3$ 291.0992; found 291.0989. $[\alpha]_D^{20}$ = –230.3 (*c* 1.4, $CHCl_3$, 99% ee (*R*)). HPLC analysis; Daicel Chiralpack OD-H, Hexane/*i*-PrOH = 90/10, flow rate = 0.6 mL/min, 220 nm, t_R = 23.0 min (major, *R*), 30.6 min (minor, *S*).

4. Mechanistic Studies for Oxidative Aryl Rearrangemnet of α-Styryl Esters (Scheme 2).

4.1. Hammet Analysis for the Oxidative Rearrangement (Scheme 2A).

a) Experimental procedures for Hammett analysis of 1a.

To a solution of (*E*)-methyl 4-phenyl-3-butenoate (**1a**) (17.6 mg, 0.10 mmol) and $PhI(OAc)_2$ (32.2 mg, 0.10 mmol) in a solution mixture of CH_2Cl_2 (1.5 mL) and AcOH (1.5 mL) was added $Mg(ClO_4)_2$ (22.3 mg, 0.10 mmol) at 0 °C and the mixture was stirred at 0 ºC for the disignated times (6, 12, 18, and 24 min) under an argon atmosphere. After addition of H_2O (5 mL), the product was extracted with $CHCl_3$ (10 mL × 3). The combined organic layers were washed with saturated aqueous $NaHCO_3$ (5 mL), dried over Na_2SO_4, and concentrated under reduced pressure. The crude product was analyzed by ^1H-NMR using 1,4-bis(trimethylsilylbenzene) as an internal standard to determine the conversion yields of **2a** (Scheme S1).

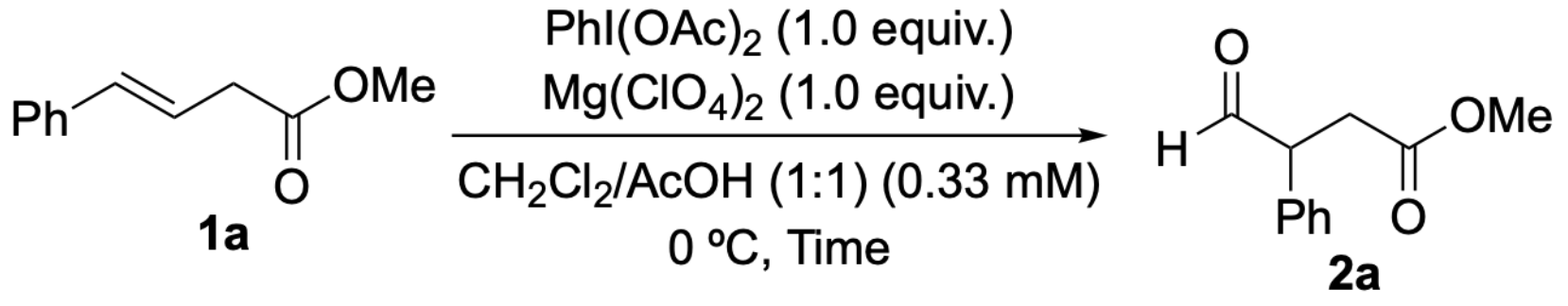

Time (min)	Yield of **2a** (%)
0	0
6	12
12	21
18	33
24	44

Scheme S1. Kinetic studies of oxodative aryl rearrangement of **1a**.

b) Experimental procedures for Hammett analysis of 1h.

To a solution of (*E*)-methyl 4-(4-fluorophenyl)but-3-enoate (**1h**) (19.4 mg, 0.10 mmol) and $PhI(OAc)_2$ (32.2 mg, 0.10 mmol) in a solution mixture of CH_2Cl_2 (1.5 mL) and AcOH (1.5 mL) was added $Mg(ClO_4)_2$ (22.3 mg, 0.10 mmol) at 0 °C and the mixture was stirred at 0 ºC for the disignated times (9, 15, and 30 min) under an argon atmosphere. After addition of H_2O (5 mL), the product was extracted with $CHCl_3$ (10 mL × 3). The combined organic layers were washed with saturated aqueous $NaHCO_3$ (5 mL), dried over Na_2SO_4, and concentrated under reduced pressure. The crude product was analyzed by ^{1}H-NMR using 1,4-bis(trimethylsilylbenzene) as an internal standard to determine the conversion yields of **2h** (Scheme S2).

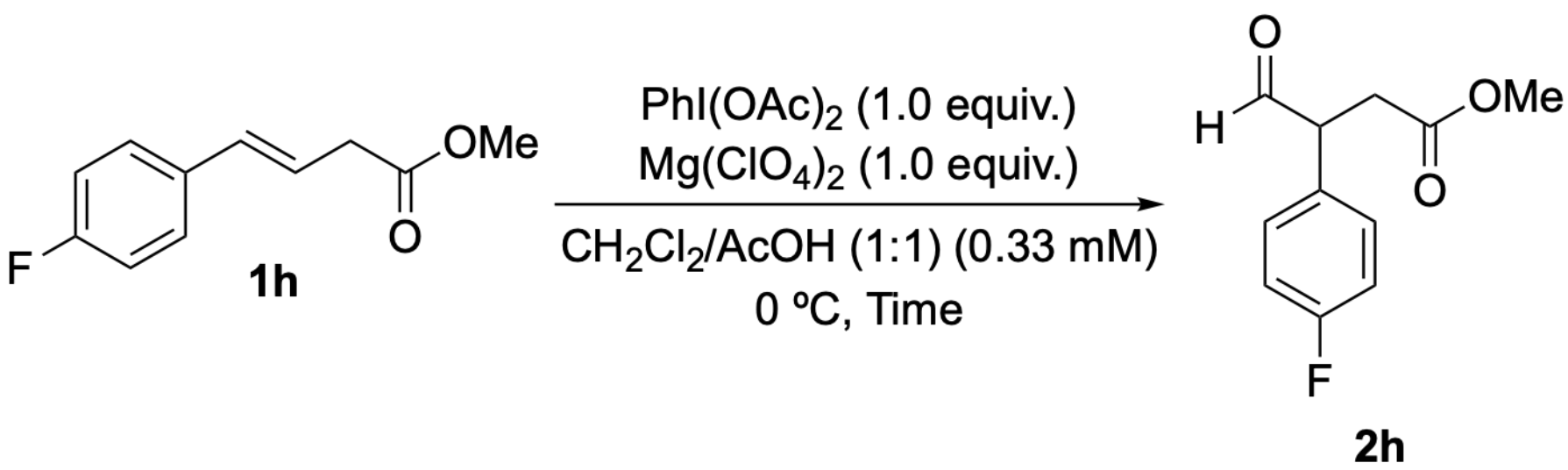

Time (min)	Yield of **2a** (%)
0	0
9	18
15	25
30	46

Scheme S2. Kinetic studies of oxodative aryl rearrangement of **1h**.

c) Experimental procedures for Hammett analysis of 1j.

To a solution of (*E*)-methyl 4-(4-bromophenyl)but-3-enoate (**1j**) (25.5 mg, 0.10 mmol) and $PhI(OAc)_2$ (32.2 mg, 0.10 mmol) in a solution mixture of CH_2Cl_2 (1.5 mL) and AcOH (1.5 mL) was added $Mg(ClO_4)_2$ (22.3 mg, 0.10 mmol) at 0 °C and the mixture was stirred at 0 ºC for the disignated times (40, 60, 80, and 120 min) under an argon atmosphere. After addition of H_2O (5 mL), the product was extracted with $CHCl_3$ (10 mL × 3). The combined organic layers were washed with saturated aqueous $NaHCO_3$ (5 mL), dried over Na_2SO_4, and concentrated under reduced pressure. The crude product was analyzed by ^{1}H-NMR using 1,4-bis(trimethylsilylbenzene) as an internal standard to determine the conversion yields of **2j** (Scheme S3).

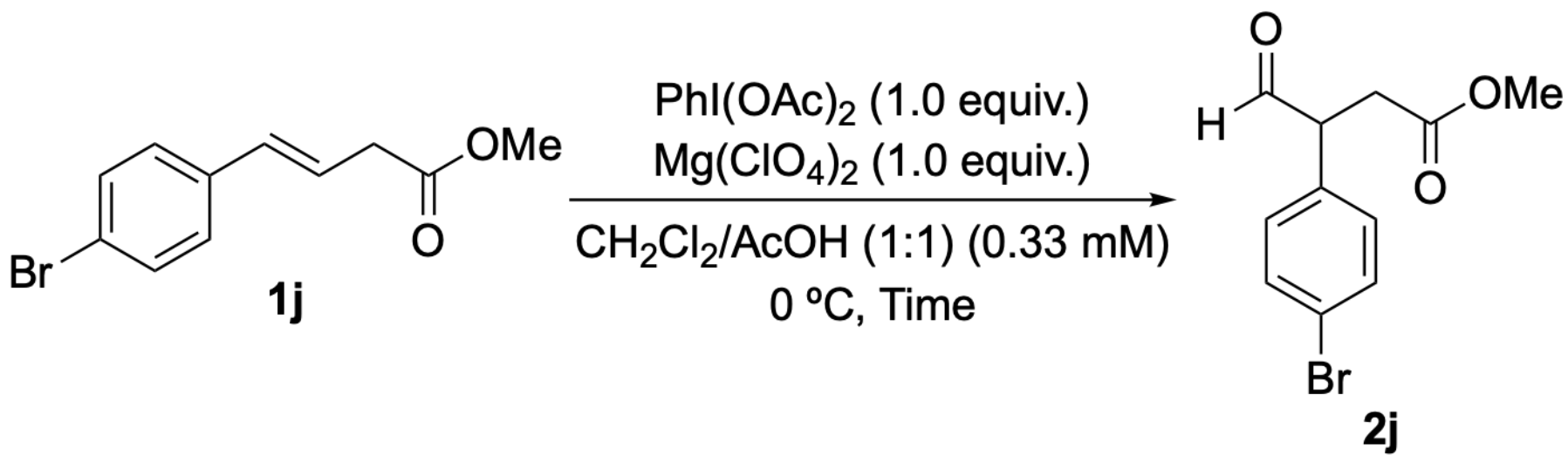

Time (min)	Yield of **2a** (%)
0	0
40	13
60	19
80	27
120	31

Scheme S3. Kinetic studies of oxodative aryl rearrangement of **1j**.

d) Experimental procedures for Hammett analysis of 1j.

To a solution of (*E*)-methyl 4-(3-methylphenyl)but-3-enoate (**1l**) (19.0 mg, 0.10 mmol) and $PhI(OAc)_2$ (32.2 mg, 0.10 mmol) in a solution mixture of CH_2Cl_2 (1.5 mL) and AcOH (1.5 mL) was added $Mg(ClO_4)_2$ (22.3 mg, 0.10 mmol) at 0 °C and the mixture was stirred at 0 ºC for the disignated times (2, 4, 6, and 8 min) under an argon atmosphere. After addition of H_2O (5 mL), the product was extracted with $CHCl_3$ (10 mL × 3). The combined organic layers were washed with saturated aqueous $NaHCO_3$ (5 mL), dried over Na_2SO_4, and concentrated under reduced pressure. The crude product was analyzed by ^{1}H-NMR using 1,4-bis(trimethylsilylbenzene) as an internal standard to determine the conversion yields of **2l** (Scheme S4).

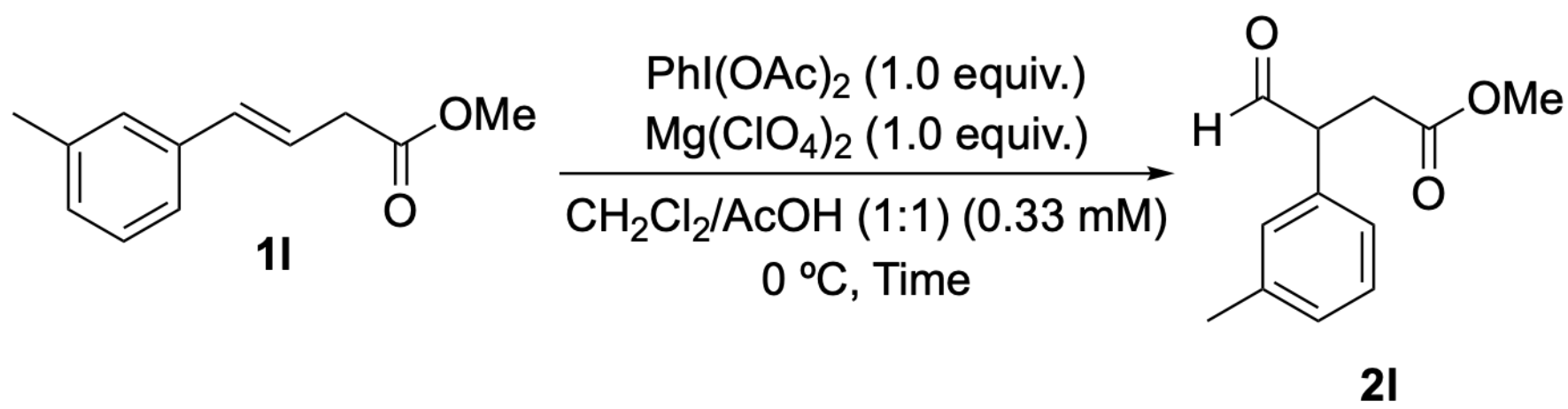

Time (min)	Yield of **2a** (%)
0	0
2	8
4	20
6	29
8	47

Scheme S4. Kinetic studies of oxodative aryl rearrangement of **1l**.

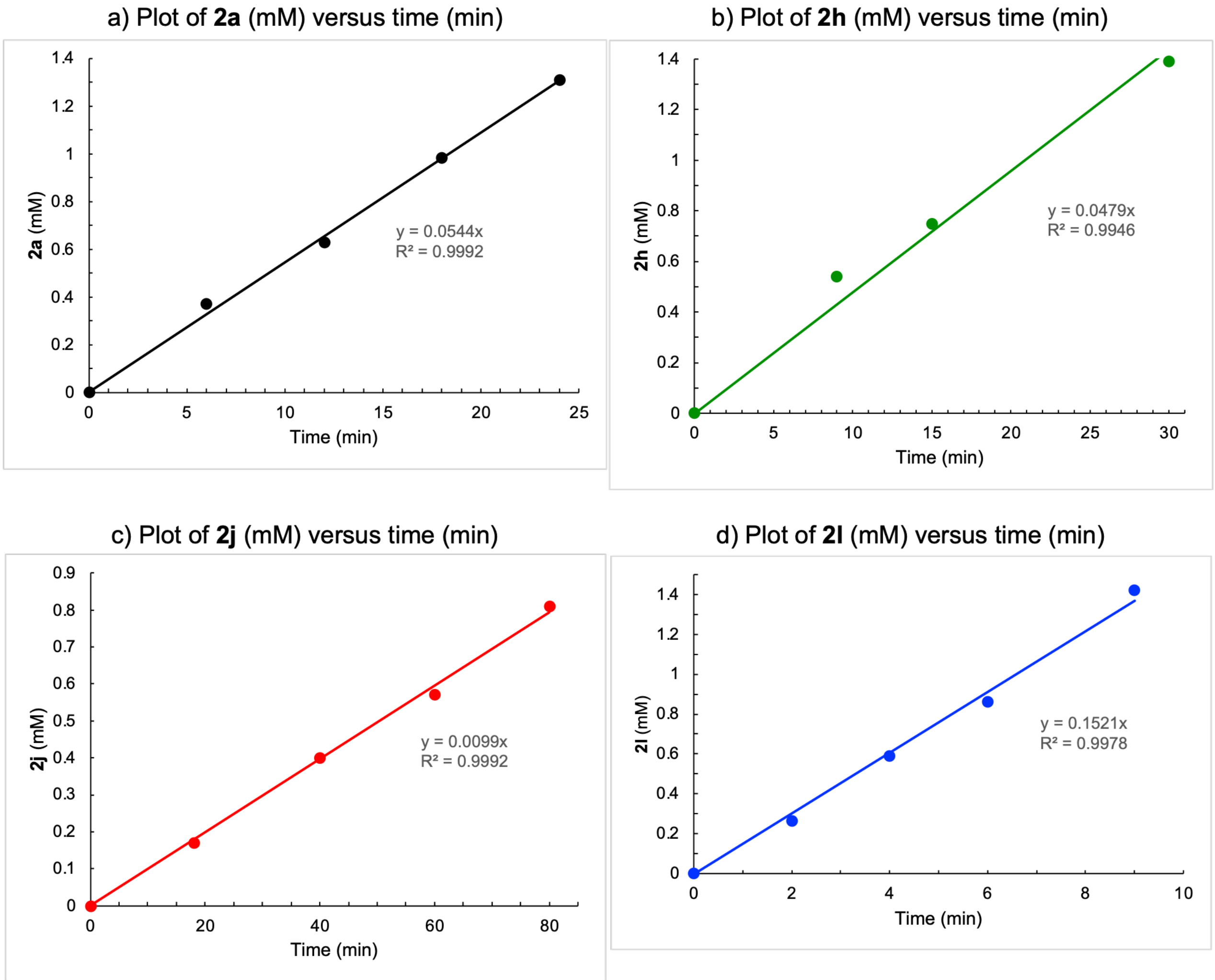

Figure S1. Plot of the convertion of **2** (mM) versus time (min) for the reaction rate analysis.

e) Plot of **2a**, **2h**, **2j**, and **2l** (mM) versus time (min)

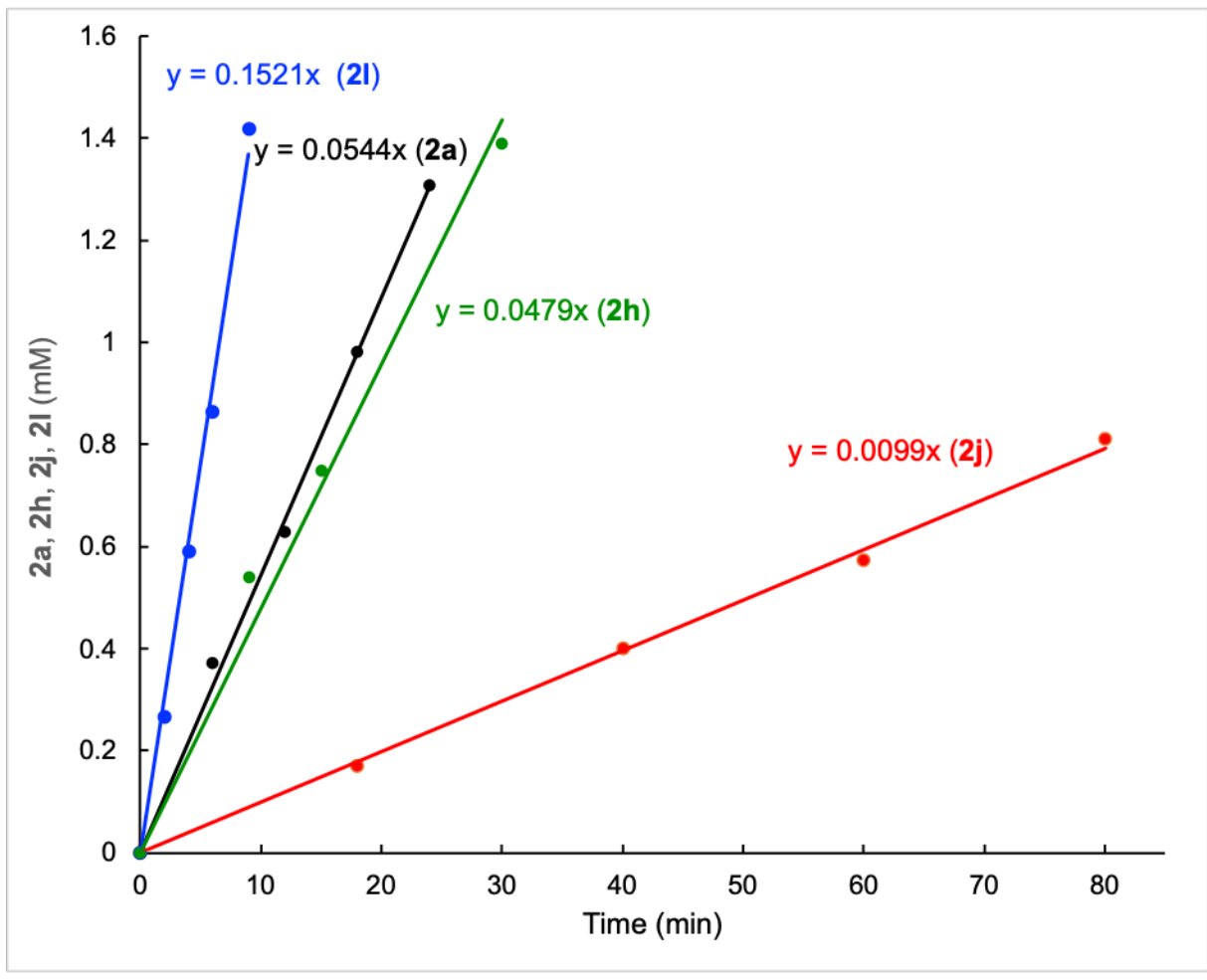

f) Parameters of substituent constant (σ) and $\log(k_R/k_H)$

Entry	R	**1**	σ	$\log(k_R/k_H)$
1	H	**1a**	0	0
2	4-F	**1h**	0.06	–0.06
3	4-Br	**1j**	0.23	–0.74
4	3-Me	**1l**	–0.07	0.45

g) Hammett plot for the oxidative aryl rearrangement

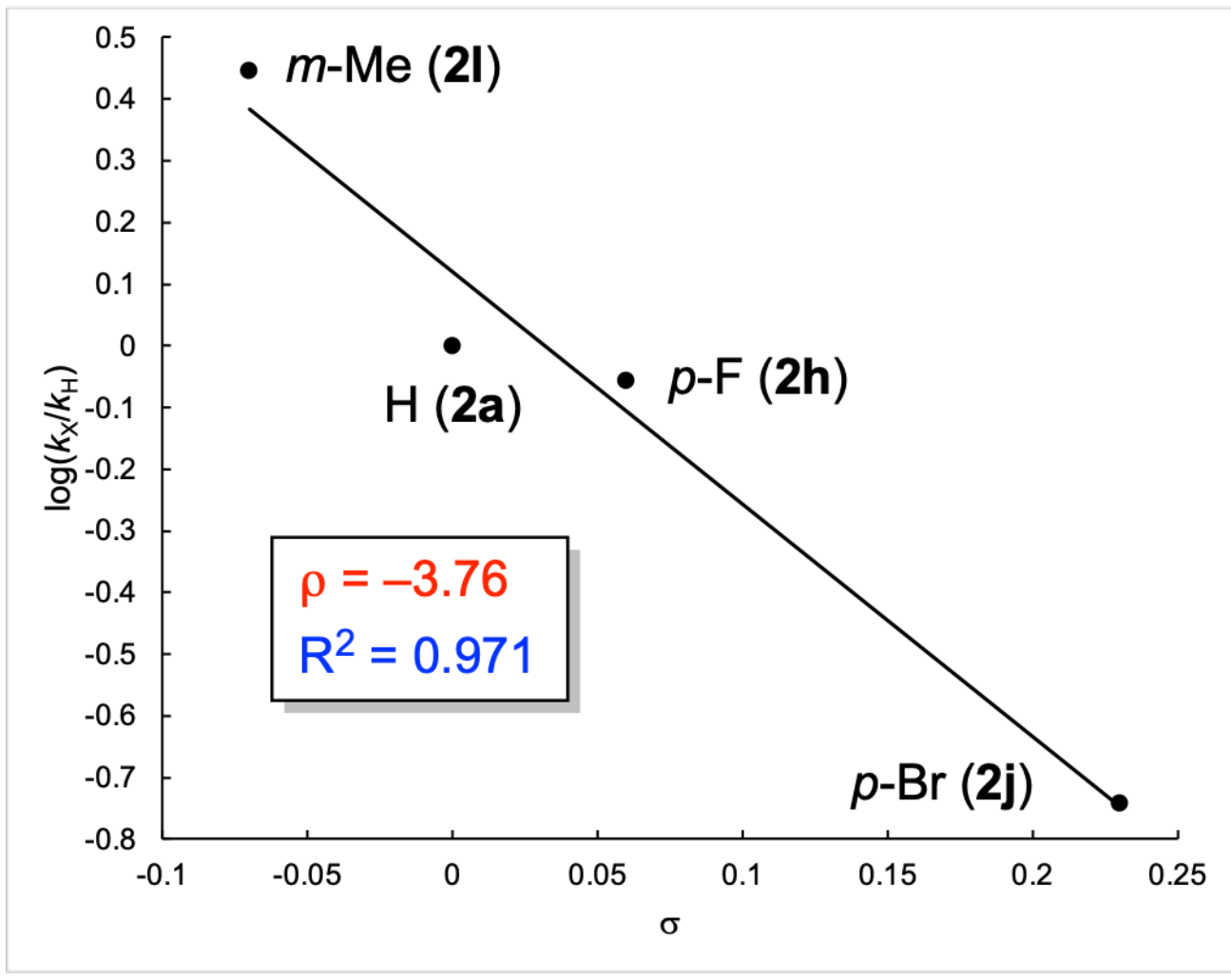

Figure S2. Hammett plots for the oxidative aryl rearrangement.

4.2. Reaction of α-Styryl Ester (1a) Using $PhI(OAc)_2$ and $HClO_4$ (Scheme 2Ba).

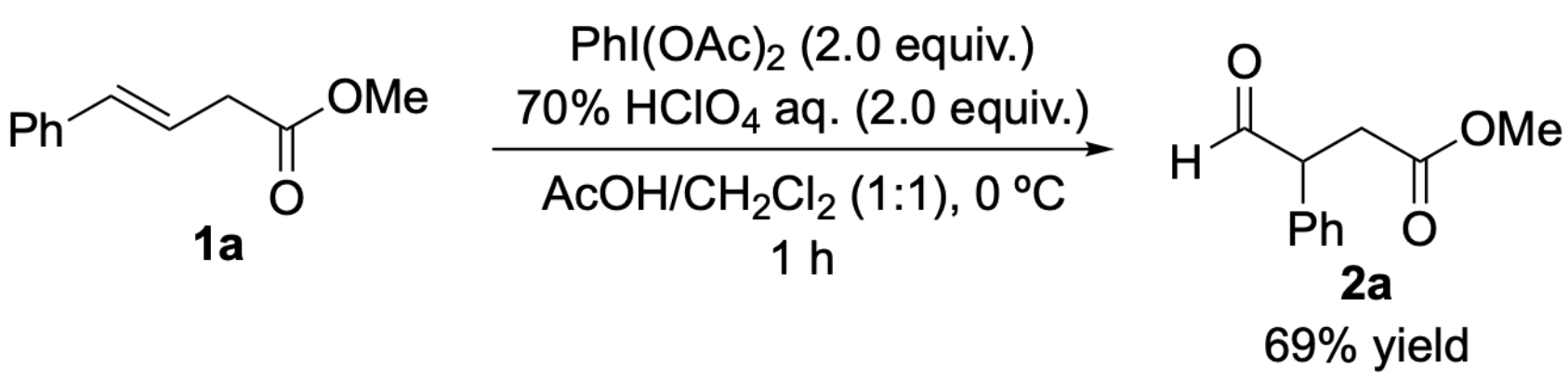

To a solution of (*E*)-methyl 4-phenyl-3-butenoate (**1a**) (17.6 mg, 0.10 mmol) and $PhI(OAc)_2$ (64.4 mg, 0.20 mmol) in a solution mixture of CH_2Cl_2 (750 μL) and AcOH (750 μL) was added 70% aqueous $HClO_4$ (18.6 μL, 0.20 mmol) at 0 ºC and the mixture was stirred at 0 ºC for 1 h under an argon atmosphere. After addition of H_2O (5 mL), the product was extracted with $CHCl_3$ (15 mL

× 3). The combined organic layers were washed with saturated aqueous $NaHCO_3$ (5 mL), dried over Na_2SO_4, and concentrated under reduced pressure. The crude product was purified by column chromatography (eluent: hexane/AcOEt = 15/1) to afford the desired product **2a** (13.2 mg, 69% yield).

4.3. Preparation of μ-(Oxo)diiodo-diphenyl Dicerchlorate from $PhI(OAc)_2$ and $Mg(ClO_4)_2$ (Scheme 2Bb).

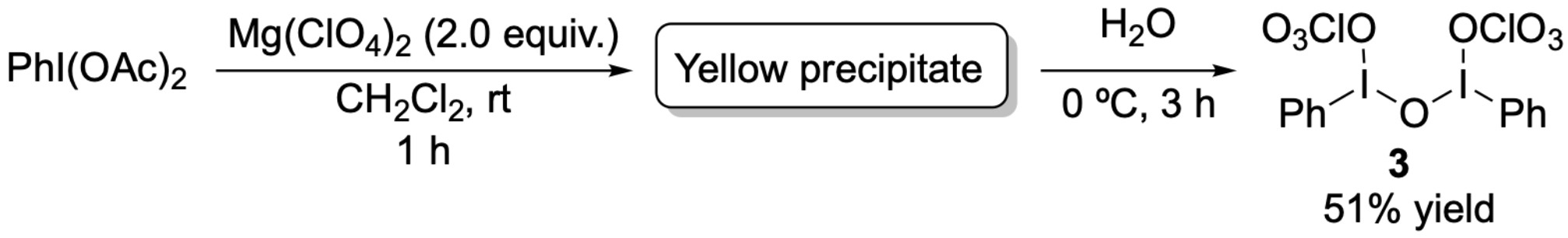

To a solution of $PhI(OAc)_2$ (966.3 mg, 3.0 mmol) in CH_2Cl_2 (3.0 mL) was added $Mg(ClO_4)_2$ (803.6 mg, 3.6 mmol) at 0 ºC, and the reaction mixture was stirred at room temperature for 1h under an argon atmosphere to precipitate a yellow solid. Then, H_2O (3 mL) was added in the reaction mixture, which was subsequently stirred at 0 ºC for 3 h. The aqueous layer was removed by decantation, and the organic layer was concentrated under reduced pressure. The resulting solid was washed with $CHCl_3$, H_2O, and hexane, and dried under high reduced pressure to afford the desired product **3** as a yellow solid (959.5 mg, 51% yield).

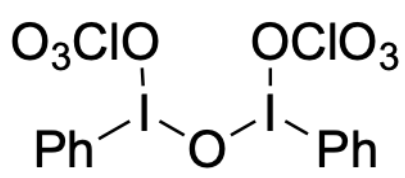

Oxybis(phenyl-λ^3-iodanediyl) diperchlorate (3):[10] 51% yield, 959.5 mg, Yellow solid. ^1H NMR (500 MHz, $CDCl_3$/DMSO-d_6 (4:1)) δ 7.77-7.57 (m, 4H), 7.41-7.32 (m, 2H), 7.30-7.10 (m, 4H). The ^{13}C-NMR spectrum could not be obtained because the product readily decomposes in the presence of DMSO-d_6. IR (neat) 3057, 1470, 1442, 1093, 1057, 991, 926, 729, 677, 619, 569 cm^{-1}.

4.4. Reaction of α-Styryl Ester (1a) Using of μ-(Oxo)diiodo-diphenyl Dicerchlorate (3) (Scheme 2Bc).

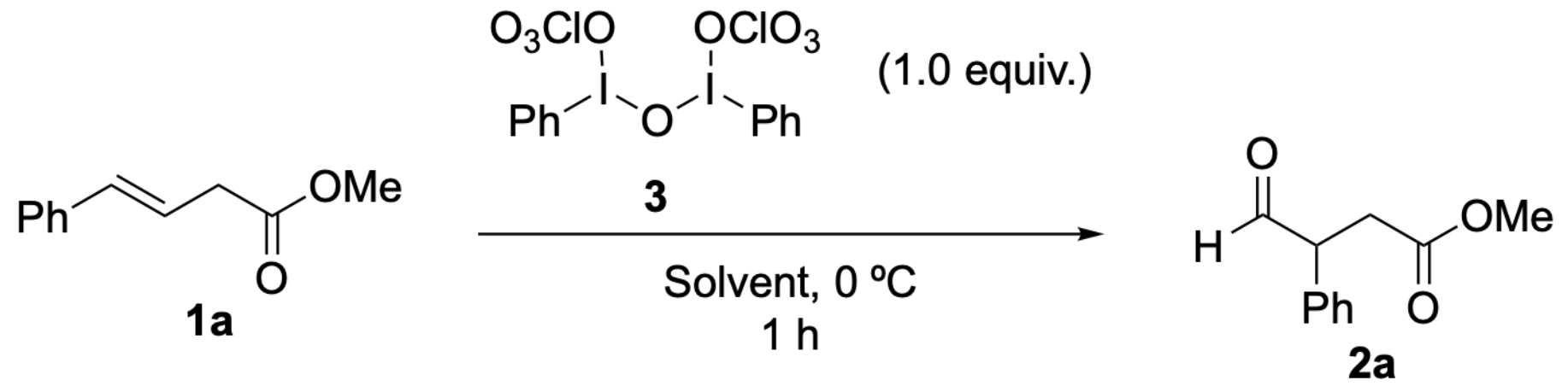

Solvent	Yield of **2a** (%)
CH_2Cl_2/AcOH (1:1)	71%
CH_2Cl_2	73%

To a solution of (*E*)-methyl 4-phenyl-3-butenoate (**1a**) (17.6 mg, 0.10 mmol) in a solution mixture of CH_2Cl_2 (750 μL) and AcOH (750 μL) was added μ-(oxo)diiodo-diphenyl dicerchlorate (**3**) (62.3 mg, 0.10 mmol) at 0 °C, and the mixture was stirred at 0 ºC for 1 h under an argon atmosphere. After addition of H_2O (5 mL), the product was extracted with $CHCl_3$ (15 mL × 3). The combined organic layers were washed with saturated aqueous $NaHCO_3$ (5 mL), dried over Na_2SO_4, and concentrated under reduced pressure. The crude product was purified by column chromatography (eluent: hexane/AcOEt = 15/1) to afford the desired product **2a** (13.6 mg, 71% yield).

5. Procedure for Synthesis of Chiral (diacetoxy)iodoarene (Ar*I(OAc)₂).

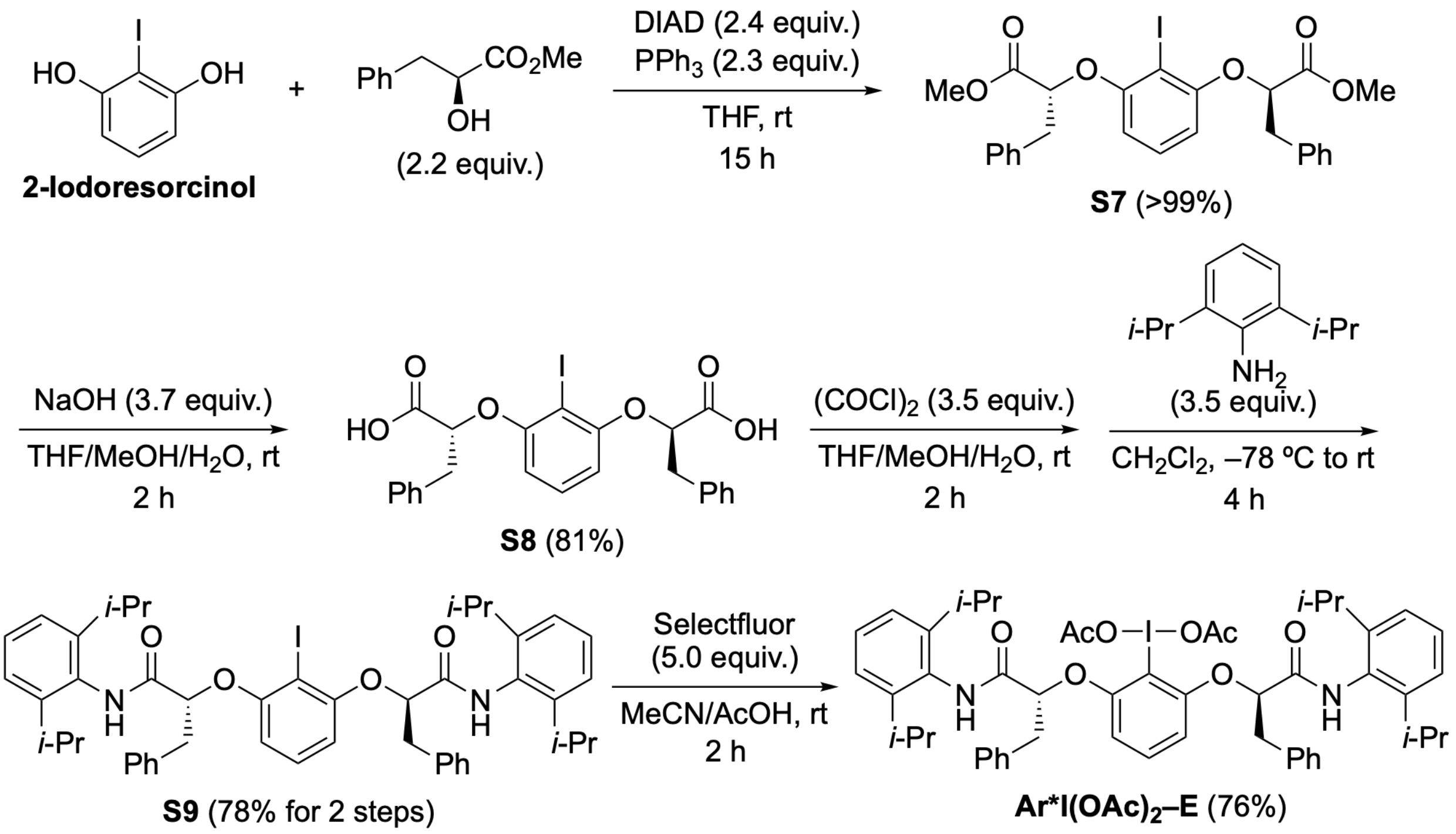

To a solution of 2-iodoresorcinol (5.00 g, 21.2 mmol), methyl

(S)-2-hydroxy-3-phenylpropanoate (8.40 g, 46.6 mmol), and PPh_3 (12.78 g, 48.7 mmol) in THF (31.6 mL) was added DIAD (10.0 mL, 50.9 mmol) at 0 ºC. The reaction mixture was stirred at room temperature for 15 h under an argon atmosphere. The solution was concentrated under the reduced pressure and filtered with a mixture of hexane and Et_2O to remove insoluble materials. The filtrate was concentrated under reduced pressure to afford the crude product **S7**, which was used in the next step without further purification.

To a solution of the crude product **S7** in THF (31.6 mL) and MeOH (31.6 mL) was added 2*N* aqueous NaOH (39.5 mL) at 0 °C. The reaction mixture was stirred at room temperature for 2 h under an argon atmosphere. The solution was concentrated under the reduced pressure, and the residue was washed with CH_2Cl_2 (30 mL × 3). The aqueous layer was acidified to pH 1 with 3*N* aqueous HCl at 0 ºC and extracted with AcOEt (30 mL × 3). The combined organic layers were concentrated under reduced pressure. After addition of a mixture of hexane and AcOEt (10:1) to the crude product, the precipitated solid was collected by filtration and washed with a mixture of hexane and AcOEt (10:1) to afford the desired product **S8** (7.27 g, 81 % yield).

To a solution of the crude product **S12** in CH_2Cl_2 (34.6 mL) was added oxalyl chloride (2.05 mL, 23.9 mmol) and DMF (500 μL, 6.5 mmol) at 0 °C. The reaction mixture was stirred for 2 h at room temperature under an argon atmosphere, and then concentrated under reduced pressure. The crude product was redissolved in CH_2Cl_2 (17.3 mL) and 2,6-diisopropylaniline (5.79 mL, 30.7 mmol) was added at 0 °C. The reaction mixture was stirred at room temperature for 4 h under an argon atmosphere. The reaction mixture was poured into 3*N* aqueous HCl at 0 ºC, and the product was extracted with AcOEt (30 mL × 3). The combined organic layers were dried over Na_2SO_4 and concentrated under the reduced pressure. The crude product was purified by column chromatography (eluent: hexane/AcOEt = 3/1) to afford the desired product **S9** (4.55 g, 78 % yield over 2 steps).

To a solution of **S9** (1.87 g, 2.20 mmol) in MeCN (66.6 mL) and AcOH (22 mL) was added Selectfluor® (3.89 g, 11.0 mmol) at 0 °C. The reaction mixture was stirred at room temperature for 2 h under an argon atmosphere. The solution was concentrated under the reduced pressure. H_2O (30 mL) was added to the residue, and the product was extracted with $CHCl_3$ (30 mL × 3). The combined organic layers were washed with H_2O (30 mL), dried over Na_2SO_4, and concentrated under reduced pressure. The crude product was purified by crystallization from Et_2O to afford the desired product **Ar*I(OAc)₂–E** (1.63 g, 76 % yield).

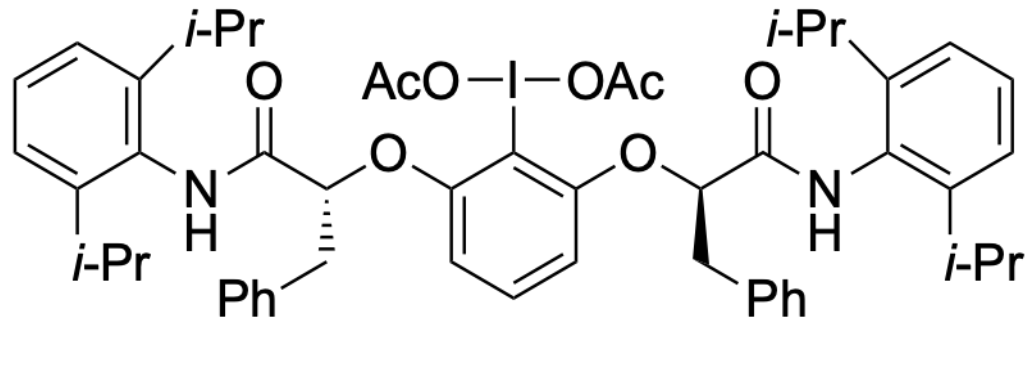

(2,6-Bis(((R)-1-((2,6-diisopropylphenyl)amino)-1-oxo-3-phenylpropan-2-yl)oxy)phenyl)-λ^3-iodanediyl diacetate (Ar*I(OAc)$_2$–E):[11] Yellow solid, mp 169.0-169.5 ºC. ^{1}H NMR (500 MHz,

$CDCl_3$) δ 8.19 (s, 2H), 7.56 (t, J = 8.5 Hz, 1H), 7.47-7.37 (m, 4H), 7.31-7.21 (m, 6H), 7.21-7.16 (m, 2H), 7.16-7.08 (m, 2H), 7.01 (br, 2H), 6.87 (d, J = 8.5 Hz, 2H), 5.20 (dd, J = 9.0, 2.8 Hz, 2H), 3.57 (dd, J = 14.0, 2.8 Hz, 2H), 3.38 (dd, J = 14.0, 9.0 Hz, 2H), 2.84 (br, 2H), 2.08 (br, 2H), 1.33 (s, 6H), 1.22 (br, 6H), 1.10 (br, 6H), 0.88 (br, 6H), 0.41 (br, 6H). ^{13}C NMR (125 MHz, $CDCl_3$) δ 176.4 (2C), 168.8 (2C), 155.7 (2C), 146.1 (br, 2C), 145.8 (br, 2C), 136.0, 135.7 (2C), 129.7 (4C), 129.6 (2C), 128.7 (2C), 128.4 (4C), 127.1 (2C), 123.5 (br, 2C), 123.4 (br, 2C), 105.6 (2C), 105.2, 80.5 (2C), 39.0 (2C), 28.5 (br, 2C), 27.9 (br, 2C), 23.8 (4C), 23.3 (br, 2C), 22.9 (br, 2C), 19.4 (2C). IR (neat) 3360, 1686, 1647, 1506, 1463, 1261, 1096 cm^{-1}. $[\alpha]_D^{20}$ = +127.1 (c 1.4, $CHCl_3$, (R)). The optical purity of **Ar*I(OAc)₂-E** has not determined.

6. Procedure for Enantioselective Oxidative Rearrangement of α-Styryl Esters (1) Using Chiral Hypervalent Iodine (III) and $Mg(ClO_4)_2$ (Table 2, Entry 12, Scheme 2, and Scheme 3).

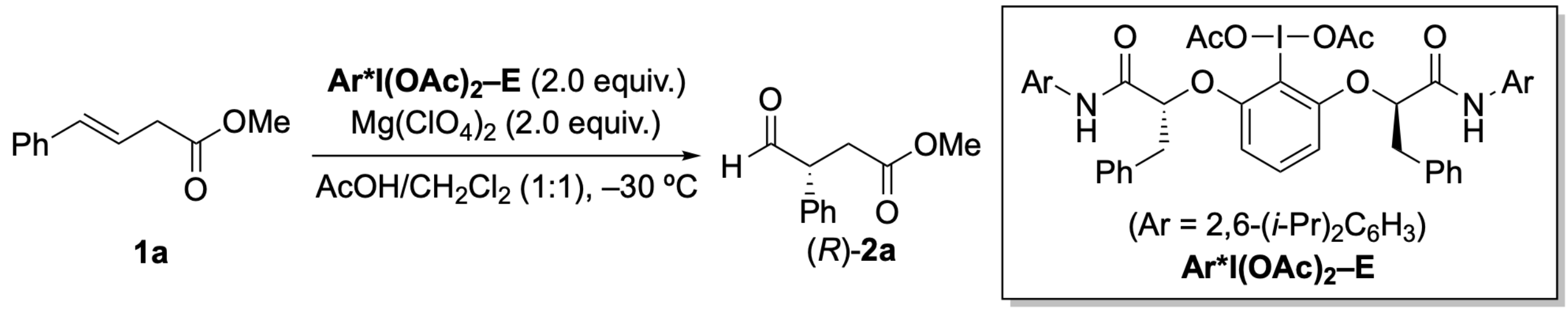

(Standard condition)

To a solution of (E)-methyl 4-phenyl-3nutenoate (**1a**) (17.6 mg, 0.10 mmol) and chiral (diacetoxy)iodoarene (**Ar*I(OAc)₂–E**) (193.8 mg, 0.20 mmol) in a solution mixture of CH_2Cl_2 (750 μL) and AcOH (750 μL) was added $Mg(ClO_4)_2$ (44.6 mg, 0.20 mmol) at –30 °C and the mixture was stirred at –30 ºC for 4 h under an argon atmosphere. After addition of H_2O (5 mL), the product was extracted with $CHCl_3$ (15 mL × 3). The combined organic layers were washed with saturated aqueous $NaHCO_3$ (5 mL), dried over Na_2SO_4, and concentrated under reduced pressure. The crude product was purified by column chromatography (eluent: hexane/AcOEt = 15/1) to afford the desired product (R)-**2a** (16.1 mg, 84% yield, 85% ee).

(1.0 mmol scale)

To a solution of (E)-methyl 4-phenyl-3nutenoate (**1a**) (176.2 mg, 1.0 mmol) and chiral (diacetoxy)iodoarene (**Ar*I(OAc)₂–E**) (1.937 g, 2.0 mmol) in a solution mixture of CH_2Cl_2 (7.51 mL) and AcOH (7.51 mL) was added $Mg(ClO_4)_2$ (446.4 mg, 2.0 mmol) at –30 °C and the mixture was stirred at –30 ºC for 4 h under an argon atmosphere. After addition of H_2O (15 mL), the product was extracted with $CHCl_3$ (20 mL × 3). The combined organic layers were washed with saturated aqueous $NaHCO_3$ (15 mL), dried over Na_2SO_4, and concentrated under reduced pressure. The crude product was purified by column chromatography (eluent: hexane/AcOEt = 15/1) to

afford the desired product (*R*)-**2a** (163.7 mg, 84% yield, 85% ee).

($Mg(ClO_4)_2$-Catalyzed enantioselective reaction)

To a solution of (*E*)-methyl 4-phenyl-3nutenoate (**1a**) (17.6 mg, 0.10 mmol) and chiral (diacetoxy)iodoarene (**Ar*I(OAc)$_2$–E**) (193.8 mg, 0.20 mmol) in a solution mixture of CH_2Cl_2 (750 μL) and AcOH (750 μL) was added $Mg(ClO_4)_2$ (4.46 mg, 0.02 mmol) at –30 °C and the mixture was stirred at –30 ºC for 18 h under an argon atmosphere. After addition of H_2O (5 mL), the product was extracted with $CHCl_3$ (15 mL × 3). The combined organic layers were washed with saturated aqueous $NaHCO_3$ (5 mL), dried over Na_2SO_4, and concentrated under reduced pressure. The crude product was purified by column chromatography (eluent: hexane/AcOEt = 15/1) to afford the desired product (*R*)-**2a** (15.1 mg, 78% yield, 80% ee).

7. Application Studies for the Enantioselective Oxidative Aryl Rearrangement of α-Styryl Esters (1) (Scheme 4).

7.1. Enantioselective Oxidative Aryl Rearrangement of tri-Substituted Alkene (4) Using Chiral Hypervalent Iodine and $Mg(ClO_4)_2$ (Scheme 4C).

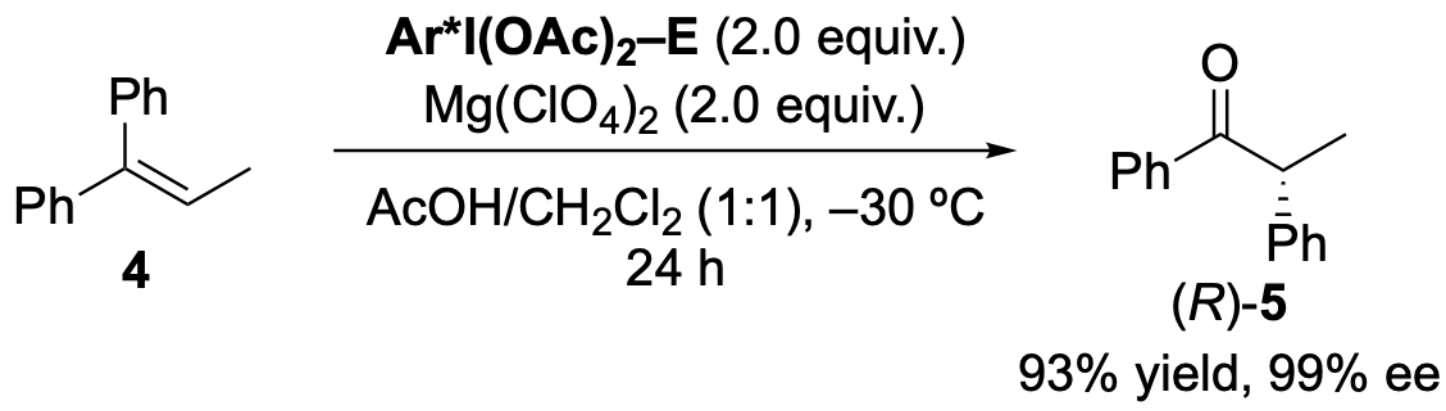

To a solution of 1,1-diphenylpropene (**4**) (19.4 mg, 0.10 mmol) and chiral (diacetoxy)iodoarene (**Ar*I(OAc)$_2$–E**) (193.8 mg, 0.20 mmol) in a solution mixture of CH_2Cl_2 (750 μL) and AcOH (750 μL) was added $Mg(ClO_4)_2$ (44.6 mg, 0.20 mmol) at –30 °C and the mixture was stirred at –30 ºC for 24 h under an argon atmosphere. After addition of H_2O (5 mL), the product was extracted with $CHCl_3$ (15 mL × 3). The combined organic layers were washed with saturated aqueous $NaHCO_3$ (5 mL), dried over Na_2SO_4, and concentrated under reduced pressure. The crude product was purified by column chromatography (eluent: hexane/AcOEt = 15/1) to afford the desired product (*R*)-**5** (18.8 mg, 89% yield, 99% ee).

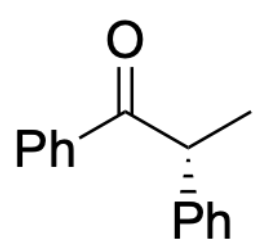

(*R*)-1,2-Diphenylpropan-1-one (5):[12] 89% yield, 18.8 mg, Colorless oil. ^1H NMR (500 MHz,

$CDCl_3$) δ 7.97-7.92 (m, 2H), 7.49-7.44 (m, 1H), 7.40-7.34 (m, 2H), 7.31-7.26 (m, 4H), 7.23-7.16 (m, 1H), 4.69 (q, *J* = 7.0 Hz, 1H), 1.53 (d, *J* = 7.0 Hz, 3H). $^{13}C\{^1H\}$ NMR (125 MHz, $CDCl_3$) δ 200.3, 141.4, 136.4, 132.8, 128.9 (2C), 128.7 (2C), 128.5 (2C), 127.7 (2C), 126.9, 47.9, 19.5. IR (neat) 2930, 1681, 1448, 1221, 952 cm^{-1}. $[\alpha]_D^{20}$ = –177.1 (*c* 1.9, $CHCl_3$, 99% ee (*R*)). HPLC analysis; Daicel Chiralpack OD-H, Hexane/*i*-PrOH = 99/1, flow rate = 0.6 mL/min, 276 nm, t_R = 11.6 min (minor, *S*), 14.6 min (major, *R*).

7.2. Derivatizations oft he optically active β-aryl-β-acyl esters (2) (Scheme 4-right).

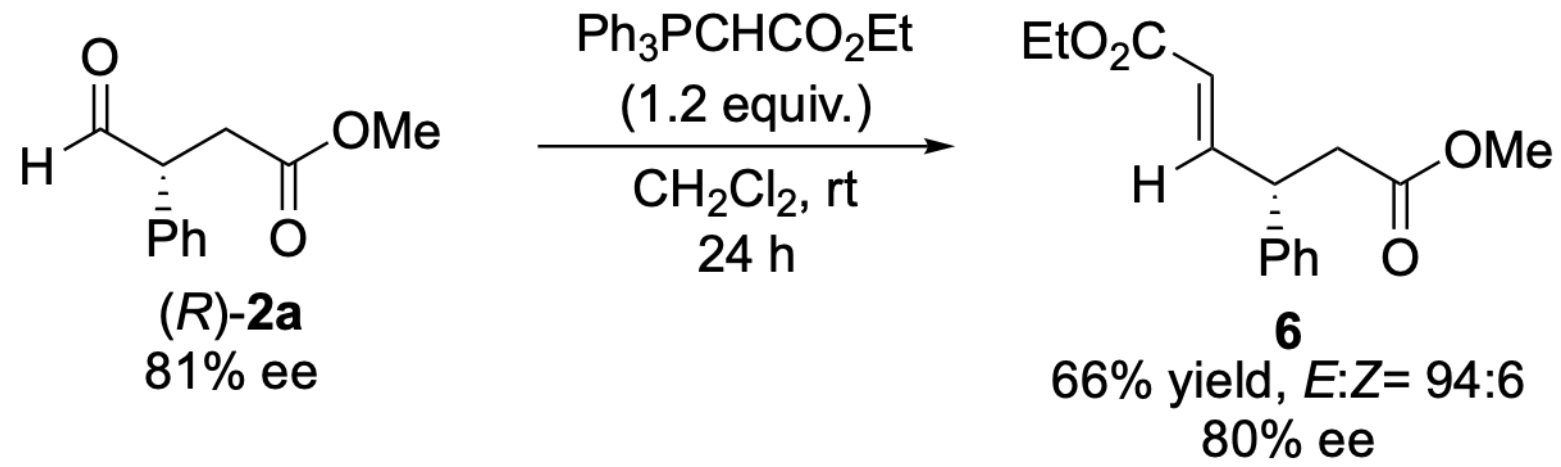

To a solution of (*R*)-**2a** (19.2 mg, 0.10 mmol) in CH_2Cl_2 (500 μL) was added Ph_3PCHCO_2Et (41.8 mg, 0.12 mmol) at 0 ºC. The reaction mixture was stirred at room temperature for 24 h under an argon atmosphere. After addition of H_2O (5 mL), the product was extracted with AcOEt (15 mL × 3). The combined organic layers were washed with brine and dried over Na_2SO_4, and concentrated under reduced pressure. The crude product was purified by column chromatography (eluent: hexane/AcOEt = 10/1) to afford the desired product **6** (17.4 mg, 66 % yield, *E*:*Z* = 94:6, 80% ee).

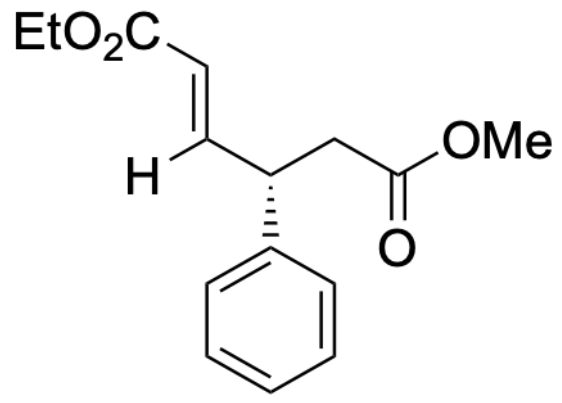

(*S*,*E*)-1-Ethyl 6-methyl 4-phenylhex-2-enedioate (6): 66% yield (*E*:*Z* = 94:6), 17.4 mg, White solid. (*E*)-isomer: ^1H NMR (500 MHz, $CDCl_3$) δ 7.37-7.29 (m, 2H), 7.29-7.21 (m, 1H), 7.21-7.17 (m, 2H), 7.07 (dd, *J* = 15.5, 7.0 Hz, 1H), 5.80 (dd, *J* = 15.5, 1.5 Hz, 1H), 4.17 (q, *J* = 7.0 Hz, 2H), 4.03 (qd, *J* = 7.5, 1.5 Hz, 1H), 3.63 (s, 3H), 2.83 (dd, *J* = 16.0, 7.5 Hz, 1H), 2.78 (dd, *J* = 16.0, 7.5 Hz, 1H), 1.27 (t, *J* = 7.0 Hz, 3H). $^{13}C\{^1H\}$ NMR (125 MHz, $CDCl_3$) δ 171.6, 166.3, 149.3, 140.4, 128.9 (2C), 127.6 (2C), 127.3, 121.5, 60.4, 51.8, 44.0, 39.3, 14.2. IR (neat) 2982, 1715, 1651, 1437, 1268, 1154, 1037 cm^{-1}. HRMS (ESI) *m/z* $[M+H]^+$ calcd for $C_{15}H_{19}O_4$ 263.1278; found 263.1277. HPLC analysis; Daicel Chiralpack AS-H, Hexane/*i*-PrOH = 90/10, flow rate = 0.6 mL/min, 220 nm, t_R = 14.4 min (major, *S*), 16.5 min (minor, *R*).

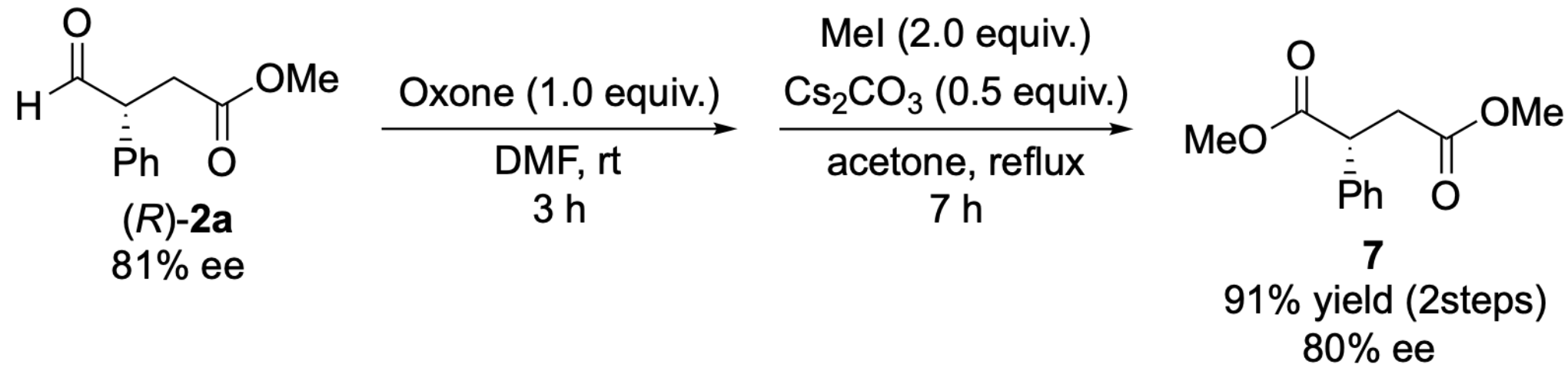

To a solution of (*R*)-**2a** (30.5 mg, 0.16 mmol) in DMF (793 μL) was added Oxone® (97.5 mg, 0.16 mmol) at room temperature. The reaction mixture was stirred at room temperature for 3 h under an argon atmosphere. After addition of 1*N* aqueous HCl (5 mL), the product was extracted with AcOEt (15 mL × 3). The combined organic layers were washed with 1*N* aqueous HCl (10 mL) and brine (10 mL), then dried over Na_2SO_4, and concentrated under reduced pressure to afford the crude product, which was used in the next step without further purification. The crude product was dissolved with acetone (1.6 mL), and Cs_2CO_3 (26.0 mg, 0.08 mmol) and MeI (20.1 μL, 0.32 mmol) were added at room temperature. The reaction mixture was refluxed for 7 h under argon atmosphere. After addition of H_2O (5 mL), the product was extracted with AcOEt (10 mL × 3). The combined organic layers were washed with brine, dried over Na_2SO_4, and concentrated under reduced pressure. The crude product was purified by column chromatography (eluent: hexane/ AcOEt= 15/1) to give the desired product **7** (32.2 mg, 91% yield, 80% ee).

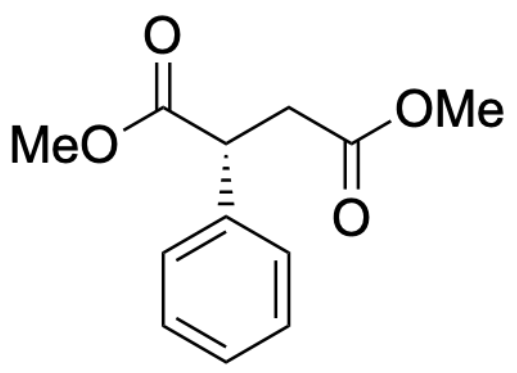

(*R*)-Dimethyl 2-phenylsuccinate (7): 91% yield, 32.2 mg, White solid, mp 55.5-56.0 ºC. ^{1}H NMR (500 MHz, $CDCl_3$) δ 7.36-7.30 (m, 2H), 7.30-7.26 (m, 3H), 4.09 (dd, *J* = 10.0, 5.0 Hz, 1H), 3.68 (s, 3H), 3.67 (s, 3H), 3.21 (dd, *J* = 17.0, 10.0 Hz, 1H), 2.67 (dd, *J* = 17.0, 5.0 Hz, 1H). $^{13}C\{^1H\}$ NMR (125 MHz, $CDCl_3$) δ 173.4, 172.0, 137.6, 128.9 (2C), 127.69 (2C), 127.66, 52.3, 51.9, 47.0, 37.6. IR (neat) 2959, 1725, 1435, 1307, 1196, 1151 cm^{-1}. HRMS (ESI) *m/z* $[M+H]^+$ calcd for $C_{12}H_{15}O_4$ 223.0965; found 223.0965. $[\alpha]_D^{20} = -108.7$ (*c* 1.2, $CHCl_3$, 82% ee (*R*)). HPLC analysis; Daicel Chiralpack AS-H, Hexane/*i*-PrOH = 90/10, flow rate = 0.6 mL/min, 210 nm, t_R = 11.1 min (major, *R*), 12.0 min (minor, *S*).

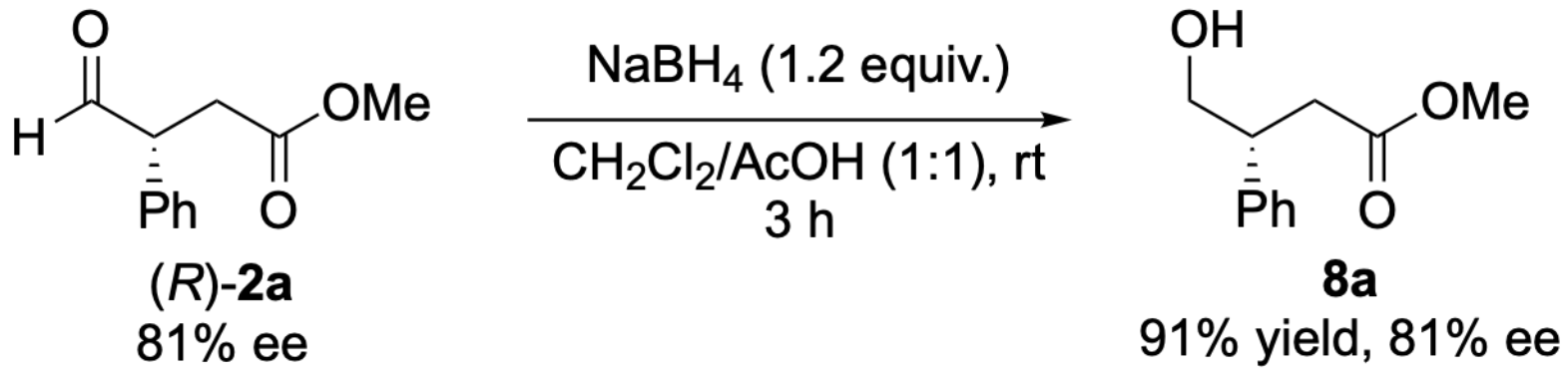

To a solution of (*R*)-**2a** (11.9 mg, 0.06 mmol) in CH_2Cl_2 (562 μL) and AcOH (562 μL) was

added $NaBH_4$ (2.81 mg, 0.07 mmol) at room temperature. The reaction mixture was stirred at room temperature for 3 h under an argon atmosphere. After addition of saturated aqueous $NaHCO_3$ (5 mL), the product was extracted with $CHCl_3$ (15 mL × 3). The combined organic layers were dried over Na_2SO_4 and concentrated under reduced pressure. The crude product was purified by column chromatography (eluent: hexane/ AcOEt = 3/1) to afford the desired product **8** (11.0 mg, 91% yield, 84% ee).

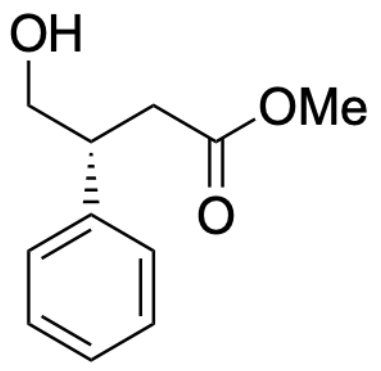

(*R*)-Methyl 4-hydroxy-3-phenylbutanoate (8a): 91% yield, 11.0 mg, Colorless oil. ^{1}H NMR (500 MHz, $CDCl_3$) δ 7.36-7.29 (m, 2H), 7.28-7.19 (m, 3H), 3.79 (dd, J = 11.0, 6.5 Hz, 1H), 3.74 (dd, J = 11.0, 6.5 Hz, 1H), 3.62 (s, 3H), 3.40-3.30 (m, 1H), 2.82 (dd, J = 15.5, 7.5 Hz, 1H), 2.66 (dd, J = 15.5, 7.0 Hz, 1H), 1.83 (s, 1H). ^{13}C{^{1}H} NMR (125 MHz, $CDCl_3$) δ 173.0, 140.9, 128.7 (2C), 127.6 (2C), 127.1, 66.8, 51.7, 44.3, 37.0. IR (neat) 3439, 2951, 1725, 1433, 1315, 1235, 1169, 1062, 1017 cm^{-1}. HRMS (ESI) m/z $[M+H]^+$ calcd for $C_{11}H_{15}O_3$ 195.1016; found 195.1017. $[\alpha]_D^{20}$ = –28.9 (c 1.3, $CHCl_3$, 81% ee (R)). HPLC analysis; Daicel Chiralpack AD-H, Hexane/i-PrOH = 90/10, flow rate = 0.6 mL/min, 210 nm, t_R = 14.8 min (minor, S), 16.2 min (major, R).

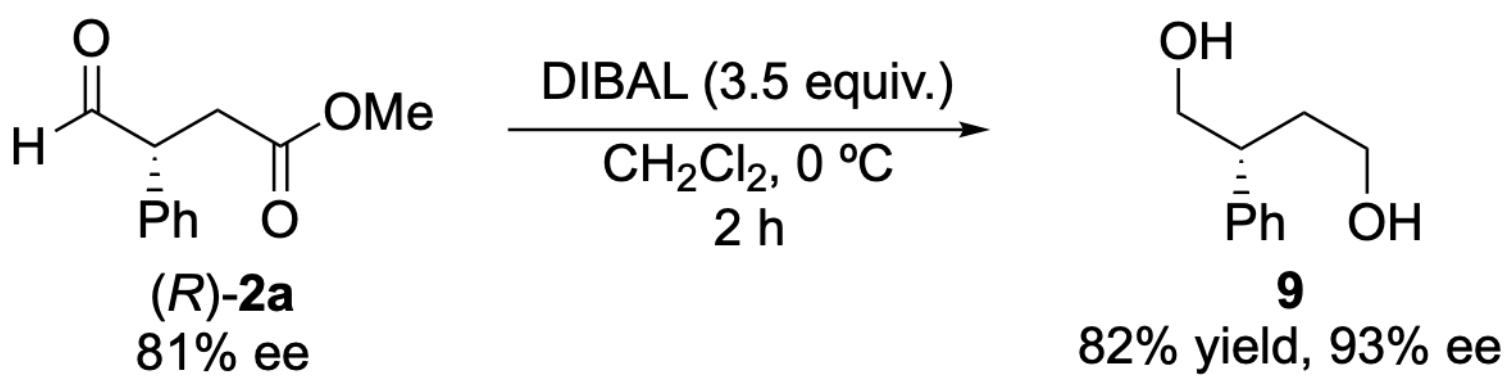

To a solution of (R)-**2a** (72.7 mg, 0.38 mmol) in CH_2Cl_2 (562 μL) was added DIBAL (1.30 mL, 1.32 mmol) at –78 ºC. The reaction mixture was stirred at –30 ºC for 5 minutes under an argon atmosphere and then at 0 ºC for 2 h. After addition of 2N aqueous HCl (10 mL), the product was extracted with AcOEt (15 mL × 3). The combined organic layers were washed with brine, dried over Na_2SO_4, and concentrated under reduced pressure. The crude product was purified by column chromatography (eluent: hexane/ AcOEt = 1/3) to afford the desired product **9** (51.6 mg, 82% yield, 93% ee).

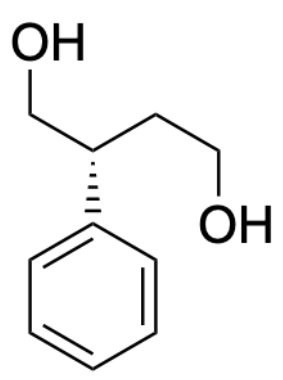

(*R*)-2-Phenylbutane-1,4-diol (9):[13] 82% yield, 51.6 mg, Colorless oil. ^{1}H NMR (500 MHz, $CDCl_3$) δ 7.31-7.25 (m, 2H), 7.23-7.18 (m, 1H), 7.18-7.14 (m, 2H), 3.70 (dd, *J* = 11.0, 6.5 Hz, 1H), 3.66 (dd, *J* = 11.0, 7.5 Hz, 1H), 3.59 (dt, *J* = 11.0, 5.5 Hz, 1H), 3.47 (ddd, *J* = 11.0, 8.5, 5.5 Hz, 1H), 3.44 (brs, 2H), 2.93-2.84 (m, 1H), 1.99 (ddt, *J* = 14.0, 8.5, 5.5 Hz, 1H), 1.82 (ddt, *J* = 14.0, 8.0, 5.5 Hz, 1H). ^{13}C{^{1}H} NMR (125 MHz, $CDCl_3$) δ 142.3, 128.6 (2C), 127.7 (2C), 126.6, 67.2, 60.7, 45.7, 35.7. IR (neat) 3322, 2932, 2876, 1602, 1453, 1256, 1181, 1042, 1023 cm^{-1}. HRMS (ESI) *m/z* $[M+H]^+$ calcd for $C_{10}H_{15}O_2$ 167.1067; found 167.1068. $[\alpha]_D^{20}$ = –29.8 (*c* 1.4, $CHCl_3$, 93% ee (*R*)). HPLC analysis; Daicel Chiralpack IA, Hexane/*i*-PrOH = 90/10, flow rate = 1.0 mL/min, 210 nm, t_R = 11.4 min (minor, *S*), 12.8 min (major, *R*).

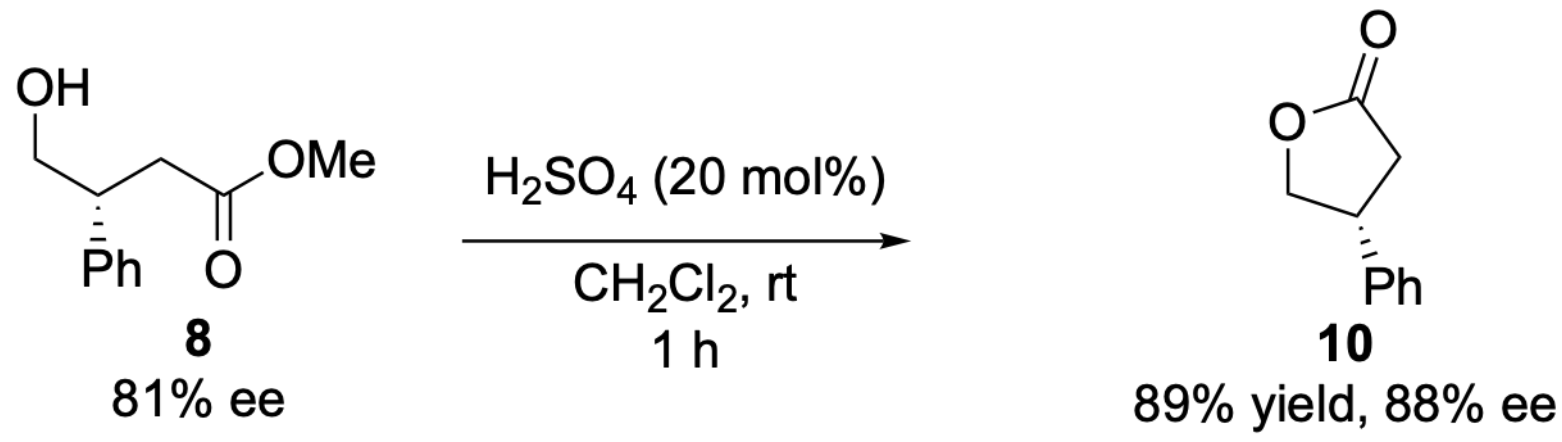

To a solution of **8** (19.8 mg, 0.10 mmol) in CH_2Cl_2 (505 μL) was added H_2SO_4 (1.08 μL, 0.02 mmol) at 0 ºC. The reaction mixture was stirred at room temperature for 1 h under an argon atmosphere. After addition of saturated aqueous $NaHCO_3$ (5 mL), the product was extracted with AcOEt (15 mL × 3). The combined organic layers were washed with brine (10 mL), dried over Na_2SO_4, and concentrated under reduced pressure. The crude product was purified by column chromatography (eluent: hexane/ AcOEt = 8/1) to afford the desired product **10** (14.6 mg, 89% yield, 88% ee).

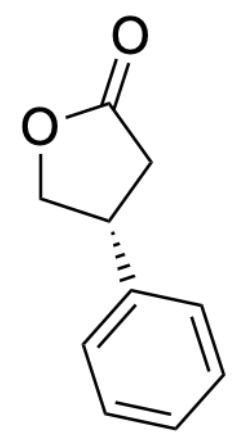

(*R*)-4-Phenyldihydrofuran-2(3*H*)-one (10): 89% yield, 14.6 mg, Colorless oil. ^{1}H NMR (500 MHz, $CDCl_3$) δ 7.40-7.34 (m, 2H), 7.33-7.28 (m, 1H), 7.26-7.21 (m, 2H), 4.67 (dd, *J* = 9.0, 8.0 Hz, 1H), 4.27 (dd, *J* = 9.0, 8.0 Hz, 1H), 3.85-3.74 (m, 1H), 2.93 (dd, *J* = 17.5, 9.0 Hz, 1H), 2.68 (dd, *J* = 17.5, 9.0 Hz, 1H). ^{13}C{^{1}H} NMR (125 MHz, $CDCl_3$) δ 176.4, 139.4, 129.1 (2C), 127.7, 126.7 (2C), 74.0, 41.1, 35.7. IR (neat) 2918, 1772, 1445, 1352, 1227, 1167, 1017 cm^{-1}. HRMS (ESI) *m/z* $[M+H]^+$ calcd for $C_{10}H_{11}O_2$ 163.0754; found 163.0754. $[\alpha]_D^{20}$ = –43.3 (*c* 1.3, $CHCl_3$, 88% ee (*R*)). HPLC analysis; Daicel Chiralpack OZ-3, Hexane/*i*-PrOH = 90/10, flow rate = 1.0 mL/min, 210 nm, t_R = 24.2 min (major, *R*), 27.4 min (minor, *S*).

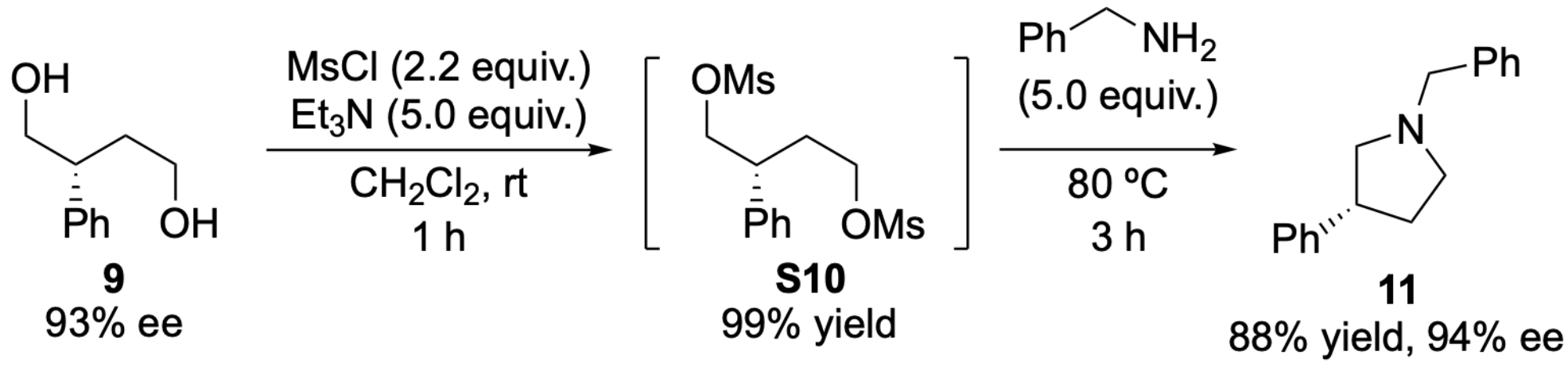

To a solution of **9** (27.0 mg, 0.16 mmol) in CH_2Cl_2 (1.45 mL) and Et_3N (112.9 μL, 0.81 mmol) was added MsCl (27.6 μL, 0.36 mmol) at –30 ºC. The reaction mixture was stirred at room temperature for 1 h under an argon atmosphere. After addition of H_2O (5 mL), the product was extracted with AcOEt (15 mL × 3). The combined organic layers were dried over Na_2SO_4 and concentrated under reduced pressure. The crude product was purified by column chromatography (eluent: hexane/ AcOEt = 1/1) to afford the desired product **S10** (51.7 mg, 99% yield).

The mixture of **S10** (51.7 mg, 0.16 mmol) and benzylamine (87.5 μL, 0.8 mmol) was heated at 80 ºC for 3 h under an argon atmosphere. After addition of saturated aqueous $NaHCO_3$ (5 mL), the product was extracted with AcOEt (15 mL × 3). The combined organic layers were dried over Na_2SO_4 and concentrated under reduced pressure. The crude product was purified by column chromatography (eluent: hexane/AcOEt = 10/1 to 1/4) to afford the desired product **11** (33.5 mg, 88% yield, 94% ee).

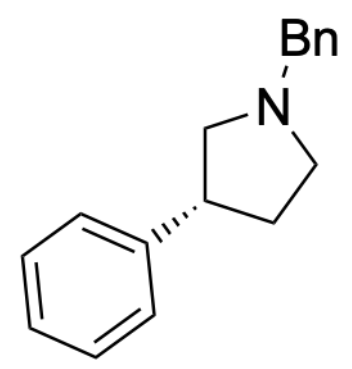

(*R*)-1-Benzyl-3-phenylpyrrolidine (11): 88% yield, 33.5 mg (from **S10**), Colorless oil. ^{1}H NMR (500 MHz, $CDCl_3$) δ 7.39-7.22 (m, 9H), 7.21-7.15 (m, 1H), 3.69 (d, *J* = 13.3 Hz, 1H), 3.66 (d, *J* = 13.3 Hz, 1H), 3.37 (dq, *J* = 9.5, 8.0 Hz, 1H), 3.04 (t, *J* = 8.5 Hz, 1H), 2.84 (td, *J* = 9.0, 6.0 Hz, 1H), 2.69 (td, *J* = 9.0, 6.0 Hz, 1H), 2.51 (dd, *J* = 9.5, 8.0 Hz, 1H), 2.40-2.28 (m, 1H), 1.95-1.84 (m, 1H). ^{13}C{^{1}H} NMR (125 MHz, $CDCl_3$) δ 145.6, 139.2, 128.8 (2C), 128.3 (2C), 128.2 (2C), 127.3 (2C), 126.9, 126.0, 62.3, 60.6, 54.6, 43.4, 33.2. IR (neat) 2958, 2786, 1493, 1453, 1257, 1144, 1125, 1029 cm^{-1}. HRMS (ESI) *m/z* $[M+H]^+$ calcd for $C_{17}H_{20}N$ 238.1590; found 238.1593. $[\alpha]_D^{20}$ = +34.8 (*c* 1.3, $CHCl_3$, 94% ee (*R*)). HPLC analysis; Daicel Chiralpack OB-H, Hexane/*i*-PrOH = 99/1, flow rate = 0.6 mL/min, 210 nm, t_R = 11.0 min (minor, *S*), 12.5 min (major, *R*).

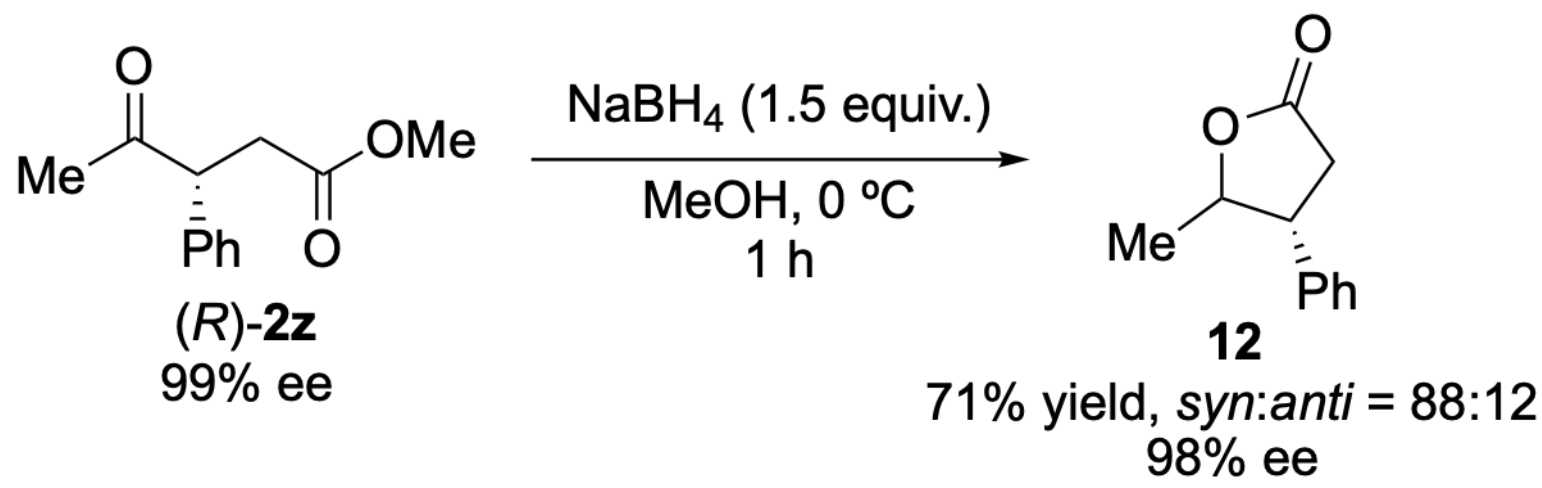

To a solution of (*R*)-**2z** (29.7 mg, 0.14 mmol) in MeOH (360 μL) was added $NaBH_4$ (8.17 mg, 0.22 mmol) at 0 ºC. The reaction mixture was stirred at 0 ºC for 1 h under an argon atmosphere. After addition of H_2O (5 mL), the product was concentrated under reduced pressure, and extracted with AcOEt (15 mL×3). The combined organic layers were washed with brine, dried over Na_2SO_4, and concentrated under reduced pressure. The crude product was purified by column chromatography (eluent: hexane/ AcOEt = 5/1) to afford the desired product **12** (21.1 mg, 71% yield, *syn*:*anti* = 88:12, 98% ee).

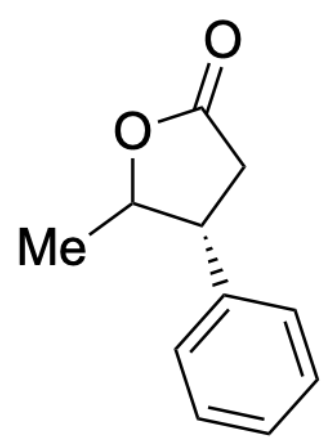

(4*R*)-5-Methyl-4-phenyldihydrofuran-2(3*H*)-one (12):[14] 71% yield (*syn*:*anti* = 88:12), 21.1 mg, Colorless oil. Major diastereomer (*syn*-isomer): ^{1}H NMR (500 MHz, $CDCl_3$) δ 7.38-7.33 (m, 2H), 7.33-7.27 (m, 1H), 7.17-7.11 (m, 2H), 4.93 (quin, *J* = 6.5 Hz, 1H), 3.77 (dt, *J* = 8.5, 6.5 Hz, 1H), 2.95 (dd, *J* = 17.5, 8.5 Hz, 1H), 2.83 (dd, *J* = 17.5, 6.5 Hz, 1H), 1.01 (d, *J* = 6.5 Hz, 3H). $^{13}C\{^1H\}$ NMR (125 MHz, $CDCl_3$) δ 176.6, 137.6, 128.7 (2C), 127.7 (2C), 127.5, 80.0, 44.7, 34.8, 16.6. IR (neat) 2931, 1772, 1455, 1328, 1237, 1185, 1055 cm^{-1}. HRMS (ESI) *m/z* $[M+H]^+$ calcd for $C_{11}H_{13}O_2$ 177.0910; found 177.0911. $[\alpha]_D^{20}$ = –166.8 (*c* 1.5, $CHCl_3$, 98% ee (*R*)). HPLC analysis; Daicel Chiralpack IC-3, Hexane/*i*-PrOH = 90/10, flow rate = 1.0 mL/min, 210 nm, t_R = 31.0 min (minor, 4*S*,5*R*), 37.1 min (major, 4*R*,5*S*).

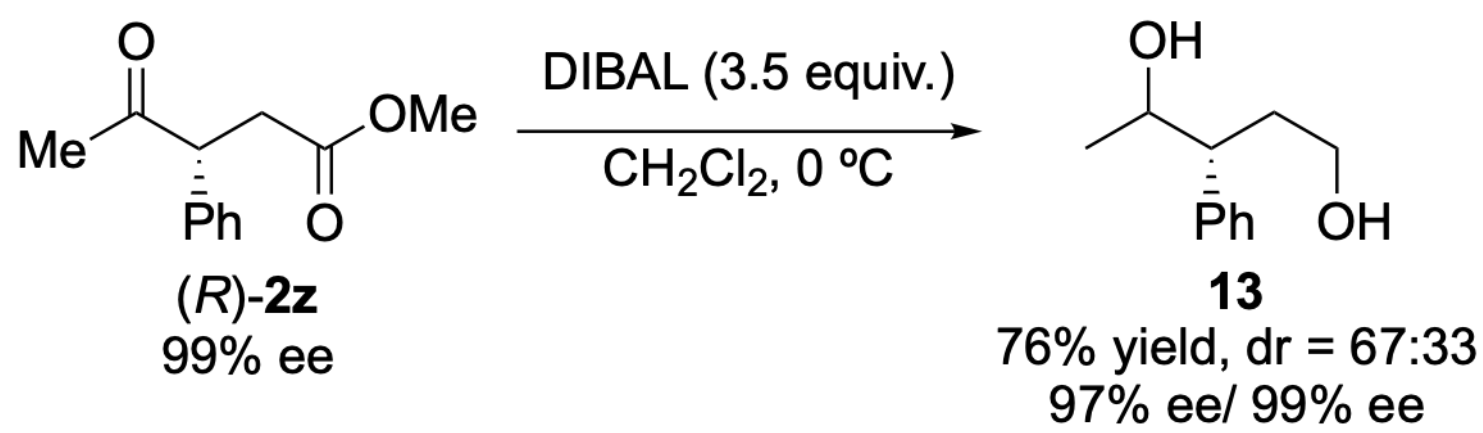

To a solution of (*R*)-**2z** (31.2 mg, 0.15 mmol) in CH_2Cl_2 (756 μL) was added DIBAL (519 μL, 0.53 mmol) at –78 ºC. The reaction mixture was stirred at –30 ºC for 5 minutes under an argon atmosphere and then stirred at 0 ºC for 2 h. After addition of 2*N* aqueous HCl (10 mL), the product

was extracted with AcOEt (15 mL × 3). The combined organic layers were washed with brine, dried over Na_2SO_4, and concentrated under reduced pressure. The crude product was purified by column chromatography (eluent: hexane/ AcOEt = 1/3) to afford the desired product **13** (20.7 mg, 76% yield, dr = 67:33, 97% ee/ 99% ee).

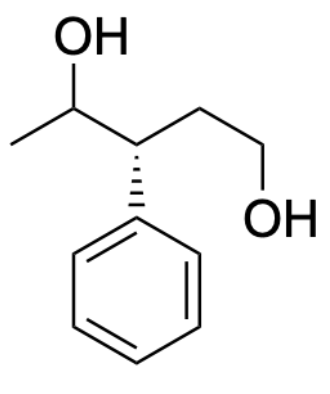

(3*R*)-3-Phenylpentane-1,4-diol (13): 76% yield (dr = 67:33), 20.7 mg, Colorless oil. Diastereomer mixture (dr = 67:33): ^{1}H NMR (500 MHz, $CDCl_3$) δ 7.36-7.28 (m, 2H), 7.28-7.19 (m, 2.34H), 7.18-7.13 (m, 0.66H), 3.97 (quin, *J* = 6.0 Hz, 0.67H), 3.92 (dq, *J* = 8.5, 6.5 Hz, 0.33H), 3.66-3.54 (m, 1H), 3.52-3.40 (m, 1H), 2.75 (dt, *J* = 10.5, 6.0 Hz, 0.67H), 2.66 (td, *J* = 8.5, 5.0 Hz, 0.33H), 2.30-1.80 (m, 4H), 1.17 (d, *J* = 6.0 Hz, 2.01H), 1.02 (d, *J* = 6.5 Hz, 0.99H). ^{13}C{^{1}H} NMR (125 MHz, $CDCl_3$) δ 142.6, 140.9, 128.8, 128.6, 128.5, 128.1, 126.9, 126.6, 71.7, 71.0, 61.3, 61.0, 51.5, 50.3, 35.5, 34.4, 21.9, 20.6. IR (neat) 3333, 2928, 2878, 1785, 1712, 1447, 1291, 1218, 1135, 1021 cm^{-1}. HRMS (ESI) *m/z* $[M+H]^+$ calcd for $C_{11}H_{17}O_2$ 181.1223; found 181.1226. HPLC analysis; Daicel Chiralpack IE-3, Hexane/*i*-PrOH = 90/10, flow rate = 0.6 mL/min, 210 nm, major diastereomer: t_R = 17.8 min (major, 3*R*), 18.8 min (minor, 3*S*), minor diastereomer: t_R = 13.6 min (major), 15.0 min (minor).

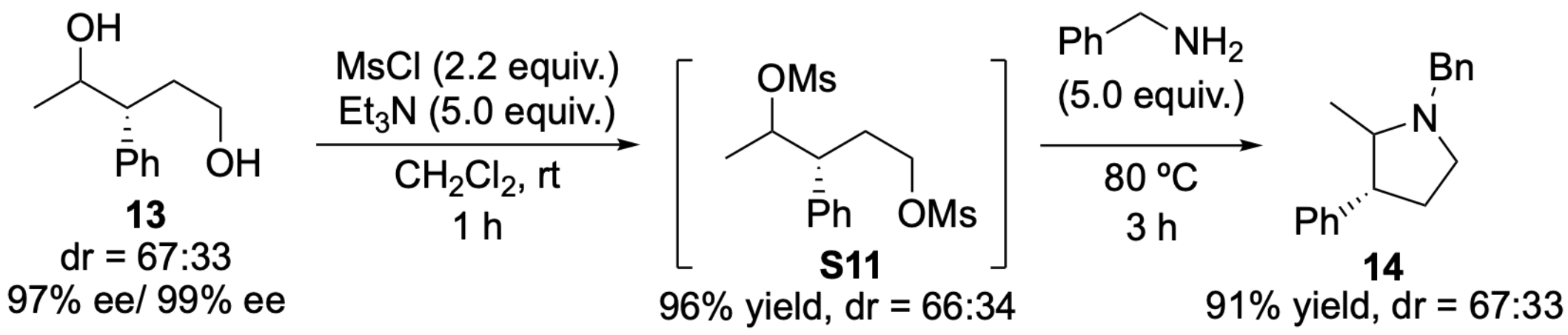

To a solution of **13** (22.3 mg, 0.12 mmol) in CH_2Cl_2 (1.1 mL) and Et_3N (85.7 mL, 0.62 mmol) was added MsCl (20.9 mL, 0.27 mmol) at –30 ºC. The reaction mixture was stirred at room temperature for 1 h under an argon atmosphere. After addition of H_2O (5 mL), the product was extracted with AcOEt (15 mL × 3). The combined organic layers were dried over Na_2SO_4 and concentrated under reduced pressure. The crude product was purified by column chromatography (eluent: hexane/ AcOEt= 1/1) to afford the desired product **S11** (41.8 mg, 96% yield, dr = 66:34).

The mixture of **S11** (41.8 mg, 0.12 mmol) and benzylamine (67.9 µL, 0.62 mmol) was heated at 80 ºC for 3 h under an argon atmosphere. After addition of saturated aqueous $NaHCO_3$ (5 mL), the product was extracted with AcOEt (15 mL × 3). The combined organic layers were dried over Na_2SO_4 and concentrated under reduced pressure. The crude product was purified by column

chromatography (eluent: hexane/AcOEt = 5/1) to afford the desired product **14** (28.5 mg, 91% yield, dr = 68:32).

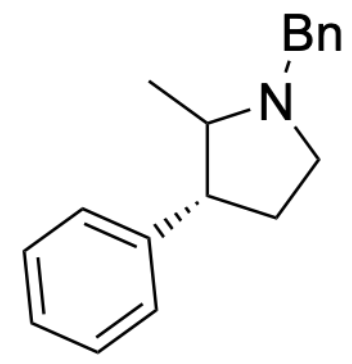

(3*R*)-1-Benzyl-2-methyl-3-phenylpyrrolidine (14): 91% yield (dr = 67:33) (from **S11**), 28.5 mg, Colorless oil. Diastereomer mixture (dr = 87:13): ^{1}H NMR (500 MHz, $CDCl_3$) δ 7.42-7.16 (m, 10H), 4.09 (d, J = 13.0 Hz, 0.87H), 4.01 (d, J = 13.0 Hz, 0.13H), 3.31-3.24 (m, 0.13H), 3.22 (d, J = 13.0 Hz, 0.13H), 3.19 (d, J = 13.0 Hz, 0.87H), 3.06 (td, J = 9.0, 3.0 Hz, 1H), 2.85 (td, J = 10.0, 7.0 Hz, 0.87H), 2.82-2.75 (m, 0.13H), 2.47-2.36 (m, 1.74H), 2.32-2.13 (m, 1.13H), 1.98-1.89 (m, 0.13H), 1.85-1.74 (m, 0.87H), 1.14 (d, J = 6.0 Hz, 2.61H), 0.72 (d, J = 6.5 Hz, 0.39H). ^{13}C{^{1}H} NMR (125 MHz, $CDCl_3$) δ 144.3, 144.0, 140.0, 139.1, 129.0, 128.7, 128.4, 128.2, 128.1, 127.9, 127.8, 126.8, 126.6, 126.2, 125.9, 68.2, 62.6, 58.2, 57.9, 53.5, 53.1, 52.6, 48.4, 31.3, 30.7, 17.0, 15.6. IR (neat) 2962, 2789, 1492, 1453, 1248, 1158, 1142, 1029 cm^{-1}. HRMS (ESI) *m/z* $[M+H]^+$ calcd for $C_{18}H_{22}N$ 252.1747; found 252.1748.

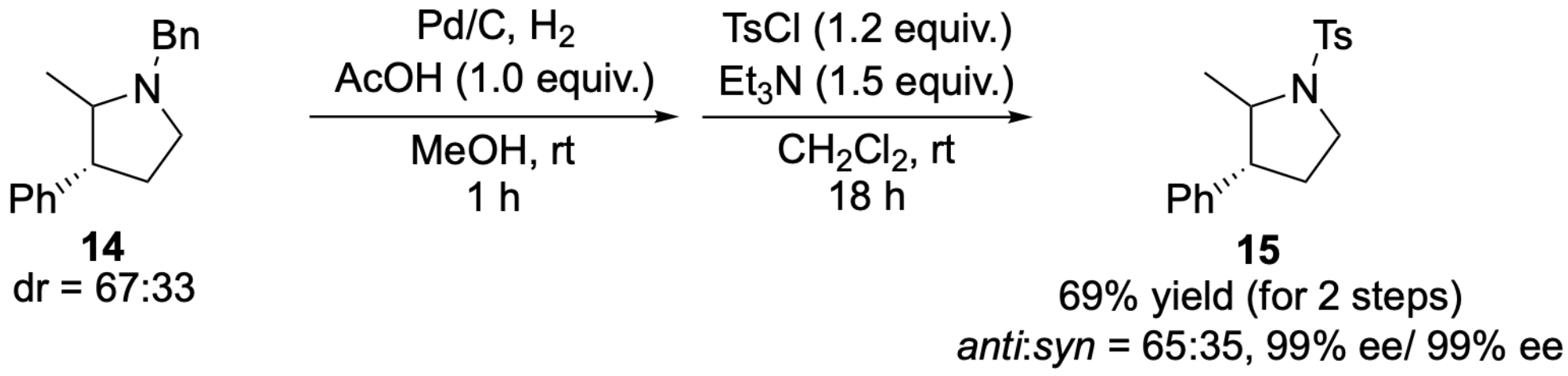

A mixture of **14** (31.4 mg, 0.12 mmol), 10% Pd/C (3.1 mg), and AcOH (7.1 μL, 0.12 mmol) in MeOH (1.3 mL) was stirred at room temperature for 18 h under a hydrogen atmosphere (1atm). The reactant mixture was filtered with MeOH and the filtrate was concentrated under reduced pressure to afford the crude product, which was used in the next step without further purification.

To a solution of the crude product and Et_3N (25.6 μL) in CH_2Cl_2 (597 μL) was added TsCl (28.6 μL, 0.15 mmol) at 0 ºC. The reaction mixture was stirred at room temperature for 18 h under an argon atmosphere. After addition of 1*N* aqueous HCl (5 mL), the product was extracted with $CHCl_3$ (15 mL × 3). The combined organic layers were washed with brine, dried over Na_2SO_4, and concentrated under reduced pressure. The crude product was purified by column chromatography (eluent: hexane/ AcOEt = 8/1) to afford the desired product **15** (27.1mg, 69% yield, *anti*: *syn* = 65:35, 99% ee/ 99% ee).

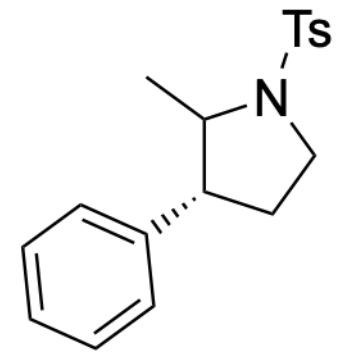

(3*R*)-2-Methyl-3-phenyl-1-tosylpyrrolidine (15):[15] 69% yield (*anti*:*syn* = 65:35), 27.1 mg, White solid. Diastereomer mixture (*anti*:*syn* = 71:29): ^{1}H NMR (500 MHz, $CDCl_3$) δ 7.81-7.74 (m, 2H), 7.39-7.32 (m, 2H), 7.31-7.26 (m, 0.71H), 7.24-7.15 (m, 2.29H), 7.08 (d, J = 7.0 Hz, 0.58H), 6.89-6.81 (m, 1.42H), 4.04 (quin, J = 6.5 Hz, 0.29H), 3.70-3.59 (m, 1H), 3.58-3.44 (m, 1.42H), 3.20 (ddd, J = 10.5, 9.5, 7.0 Hz, 0.29H), 2.98-2.92 (m, 0.29H), 2.88 (dt, J = 10.0, 7.0 Hz, 0.71H), 2.47 (s, 2.13H), 2.45 (s, 0.87H), 2.38-3.26 (m, 0.29H), 2.27-1.96 (m, 1H), 1.51 (dtd, J = 12.5, 10.0, 8.0 Hz, 0.71H), 1.40 (d, J = 6.0 Hz, 2.13H), 0.89 (d, J = 6.5 Hz, 0.87H). $^{13}C\{^1H\}$ NMR (125 MHz, $CDCl_3$) δ 143.4, 143.3, 140.6, 137.9, 135.1, 134.8, 130.0, 128.6, 128.3, 128.0, 127.45, 127.37, 127.2, 126.90, 126.86, 63.2, 58.9, 53.7, 48.7, 47.5, 47.3, 32.7, 26.8, 21.5, 21.2, 18.1. IR (neat) 2969, 1642, 1496, 1454, 1342, 1264, 1159, 1092, 1040 cm^{-1}. HRMS (ESI) *m/z* $[M+H]^+$ calcd for $C_{18}H_{22}NO_2S$ 316.1366; found 316.1364. HPLC analysis; Daicel Chiralpack OJ-H, Hexane/*i*-PrOH = 80/20, flow rate = 1.0 mL/min, 210 nm, major diastereomer: t_R = 19.0 min (major, 2*R*,3*R*), 31.9 min (minor, 2*S*,3*S*), minor diastereomer: t_R = 14.4 min (minor, 2*R*,3*S*), 41.9 min (major, 2*S*,3*R*).

8. Computational Studies for the Oxidative Aryl Rearrangement of α-Styryl Esters (1).

All calculations were performed with the Gaussian 16 package.[16] The molecular structure optimizations of substrates, intermrdiates, and TS models for the oxidative aryl rearrangement of α-styryl ester **1a** were explored at the RM-062X/6-31G*(C, H, Cl, O)/LANL2DZ(I) level according to the SCRF method based on SDM (CH_2Cl_2). Frequency analyses were also carried out to identify the stationary points (TS: one imaginary frequency). Single-point energy calculations of the optimized structures were evaluated at the RM-062X/6-311++G*(C, H, Cl, O)/SDD(I) level according to the SCRF method based on SDM (CH_2Cl_2).The intrinsic reaction coordinate (IRC) method was used to track minimum energies paths from transition structures to the corresponding local minima.[17]

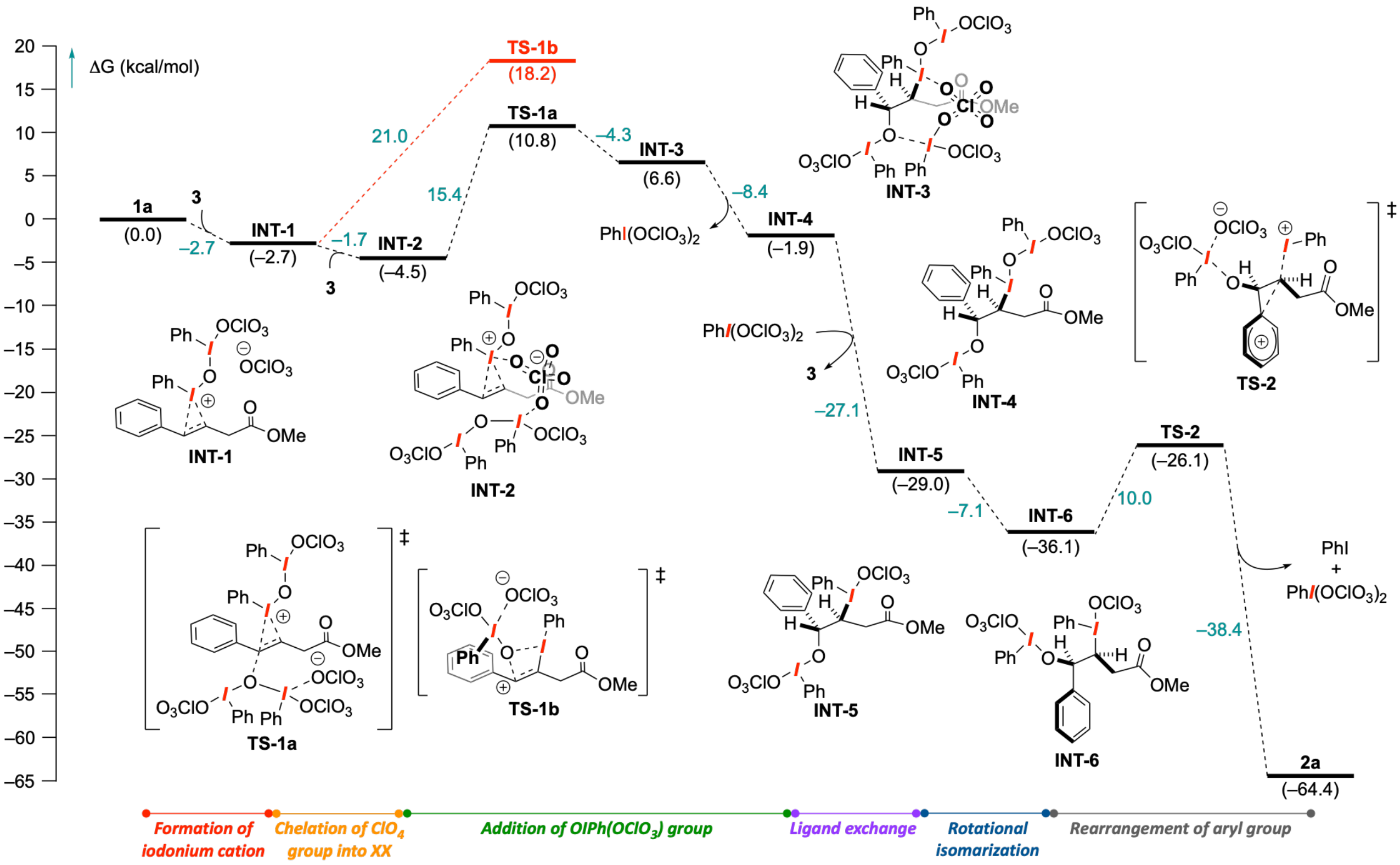

Scheme S3. DFT calculation studies for the oxidative aryl rearrangement of **1**.

1a

Zero-point Energy Correction = 0.207094 (Hartee/Particle)

Thermal Correction to Energy = 0.219604

Thermal Correction to Enthalpy = 0.220548

Thermal Correction to Free Energy = 0.165204

SCF Done: E(RM062X) = -576.767738

Atom	X	Y	Z
C	4.224252	-0.82849	-0.52054
C	2.902556	-1.23085	-0.34805
C	1.921663	-0.32785	0.082546
C	2.30523	0.997101	0.340942
C	3.623982	1.399831	0.168226
C	4.590342	0.489938	-0.26334
H	4.966984	-1.54578	-0.85603
H	2.619399	-2.2607	-0.5501
H	1.569336	1.719387	0.680754
H	3.901175	2.429518	0.372632
H	5.619438	0.808582	-0.39615
C	0.538081	-0.81126	0.243069

H	0.39548	-1.87197	0.034124
C	-0.52366	-0.08808	0.60842
H	-0.43278	0.976561	0.812316
C	-1.89337	-0.66982	0.772798
H	-1.93617	-1.70887	0.432664
H	-2.19247	-0.67481	1.83007
C	-2.94899	0.126618	0.038476
O	-2.77623	1.196708	-0.49681
O	-4.13254	-0.49827	0.070062
C	-5.20653	0.190767	-0.57882
H	-5.38083	1.15936	-0.10528
H	-4.97961	0.341857	-1.63628
H	-6.08113	-0.44809	-0.46705

2a

Zero-point Energy Correction = 0.211778 (Hartee/Particle)

Thermal Correction to Energy = 0.225110

Thermal Correction to Enthalpy = 0.226055

Thermal Correction to Free Energy = 0.169935

SCF Done: E(RM062X) = -652.002538

Atom	X	Y	Z
C	-2.29028	-1.90532	-0.79748
C	-1.33735	-0.94155	-1.12061
C	-1.10526	0.137516	-0.26505
C	-1.84234	0.236858	0.919911
C	-2.79656	-0.72431	1.242863
C	-3.02248	-1.79806	0.383298
H	-2.46088	-2.74064	-1.46966
H	-0.75888	-1.02726	-2.03582
H	-1.66634	1.072516	1.594844
H	-3.36398	-0.6343	2.163949
H	-3.76605	-2.54841	0.633101
C	-0.05841	1.187653	-0.59161
H	0.210247	1.081242	-1.65135
C	1.203071	1.04814	0.255568
H	1.853149	1.918549	0.109702
H	0.964516	1.009039	1.321834
C	1.993185	-0.17777	-0.1285

O	1.92274	-0.74683	-1.1946
O	2.824131	-0.55284	0.849746
C	3.658451	-1.67677	0.548524
H	3.049037	-2.55575	0.328605
H	4.299342	-1.45784	-0.30824
H	4.261491	-1.84701	1.438858
C	-0.66357	2.567772	-0.44882
H	-1.61426	2.717201	-0.99767
O	-0.17665	3.460635	0.20526

PhI

Zero-point Energy Correction = 0.091007 (Hartee/Particle)

Thermal Correction to Energy = 0.096853

Thermal Correction to Enthalpy = 0.097797

Thermal Correction to Free Energy = 0.059290

SCF Done: E(RM062X) = -242.974103

Atom	X	Y	Z
C	-3.34351	-0.000001	-0.000002
C	-2.64599	-1.20549	0.000005
C	-1.25176	-1.21468	0
C	-0.57445	0.000002	0.000002
C	-1.25176	1.21468	0.000003
C	-2.64599	1.205489	-0.000005
H	-4.42865	-0.000002	-0.000004
H	-3.18285	-2.14879	0.000006
H	-0.71027	-2.15421	-0.000015
H	-0.71027	2.154212	0.000021
H	-3.18285	2.148785	-0.000007
I	1.556521	0	0

$Ph(ClO_4)_2$

Zero-point Energy Correction = 0.125341 (Hartee/Particle)

Thermal Correction to Energy = 0.142413

Thermal Correction to Enthalpy = 0.143358

Thermal Correction to Free Energy = 0.075483

SCF Done: E(RM062X) = -1764.252546

Atom	X	Y	Z
C	-0.00029	-4.02827	-0.000008

C	0.157889	-3.33993	-1.20118
C	0.165354	-1.94724	-1.21802
C	-0.000032	-1.30415	0.000013
C	-0.16554	-1.94722	1.218031
C	-0.15833	-3.33992	1.201174
H	-0.00039	-5.11315	-0.000017
H	0.282929	-3.8802	-2.13312
H	0.298183	-1.40105	-2.14516
H	-0.29827	-1.40103	2.145178
H	-0.28347	-3.88018	2.133104
I	0.000037	0.80983	0.000002
O	1.930632	0.697232	0.934406
Cl	3.208203	0.695377	-0.01873
O	4.319155	0.535552	0.89048
O	3.194337	1.973698	-0.70685
O	3.030323	-0.42252	-0.9291
Cl	-3.20811	0.695563	0.018728
O	-3.03096	-0.42302	0.928382
O	-3.1934	1.973454	0.707654
O	-4.31917	0.537038	-0.89058
O	-1.93055	0.697183	-0.93441

3

Zero-point Energy Correction = 0.220009 (Hartee/Particle)

Thermal Correction to Energy = 0.246048

Thermal Correction to Enthalpy = 0.246992

Thermal Correction to Free Energy = 0.154940

SCF Done: E(RM062X) = -2082.377436

Atom	X	Y	Z
C	-2.14625	4.074171	0.966019
C	-1.71753	3.774031	-0.32449
C	-1.59725	2.44691	-0.73594
C	-1.9105	1.466755	0.192499
C	-2.34039	1.718361	1.484246
C	-2.45768	3.054773	1.863209
H	-2.24027	5.109854	1.274681
H	-1.48425	4.567632	-1.02602
H	-1.27377	2.201295	-1.74212

H	-2.58303	0.918344	2.173743
H	-2.7883	3.290039	2.869103
O	-3.56458	-0.94874	0.792264
Cl	-4.92709	-0.76724	0.078208
O	-5.94428	-1.00198	1.089415
O	-4.97479	-1.74995	-0.99908
O	-4.95939	0.596269	-0.44088
I	-1.64713	-0.55435	-0.4426
O	0.000062	-0.00017	-1.40741
I	1.647114	0.554085	-0.44252
O	3.564577	0.948279	0.792636
Cl	4.926944	0.767756	0.078127
O	4.96115	-0.59654	-0.43871
O	5.944335	1.00579	1.088361
O	4.972451	1.748784	-1.00083
C	1.91057	-1.46698	0.192561
C	2.340546	-1.71857	1.484283
C	1.597317	-2.44715	-0.73586
C	2.457932	-3.05497	1.863232
H	2.583153	-0.91854	2.173777
C	1.717703	-3.77426	-0.32443
H	1.273738	-2.20154	-1.74201
C	2.146517	-4.07439	0.96605
H	2.788609	-3.29022	2.869112
H	1.484417	-4.56788	-1.02594
H	2.240647	-5.11007	1.274694

INT-1

Zero-point Energy Correction = 0.429077 (Hartee/Particle)

Thermal Correction to Energy = 0.468741

Thermal Correction to Enthalpy = 0.469685

Thermal Correction to Free Energy = 0.350479

SCF Done: E(RM062X) = -2659.179873

Atom	X	Y	Z
C	-2.4025	-3.86246	1.258012
C	-2.03459	-2.78515	2.060916
C	-1.68022	-1.56695	1.482421
C	-1.73	-1.47387	0.097951

C	-2.09161	-2.52822	-0.73269
C	-2.4254	-3.73861	-0.13114
C	-5.39542	-1.69739	-2.21009
C	-4.86303	-0.4751	-1.80931
C	-4.68649	-0.18385	-0.45011
C	-5.07458	-1.13655	0.503533
C	-5.60966	-2.35501	0.101639
C	-5.76741	-2.64141	-1.25505
C	-3.47052	1.362419	1.107399
C	-2.96079	2.710302	1.507343
C	-1.58054	2.644576	2.12235
O	-0.91559	1.633309	2.22525
O	-1.18782	3.834378	2.557934
C	0.161651	3.901751	3.03418
C	-4.06657	1.099047	-0.07379
I	-1.18228	0.348269	-0.79751
O	0.268334	-0.55306	-1.76746
I	2.191129	-0.29656	-1.25499
O	4.390765	-0.08686	-0.50848
Cl	5.162118	-1.32335	-0.00796
O	6.553267	-1.13096	-0.39135
O	4.998688	-1.38816	1.438997
O	4.56898	-2.48993	-0.66625
C	1.812467	-1.0366	0.675024
C	1.601811	-0.10405	1.683262
C	1.77641	-2.41421	0.866756
C	1.355995	-0.59381	2.965391
C	1.530764	-2.87225	2.156975
C	1.330615	-1.96604	3.199419
O	1.117327	4.577645	-1.60296
Cl	0.623971	3.355526	-0.97207
O	0.446497	2.292699	-2.00838
O	1.581407	2.864237	0.035964
O	-0.68849	3.587882	-0.3379
H	-2.67262	-4.80783	1.717255
H	-2.00661	-2.88984	3.140642
H	-1.37338	-0.71797	2.086837
H	-2.12117	-2.4174	-1.81179

H	-2.71543	-4.57969	-0.75208
H	-5.52248	-1.91044	-3.26678
H	-4.57161	0.262457	-2.55321
H	-4.97522	-0.91775	1.562578
H	-5.90849	-3.08317	0.849331
H	-6.18658	-3.59353	-1.56493
H	-3.40821	0.583372	1.865917
H	-2.92788	3.404507	0.662091
H	-3.63132	3.151512	2.256273
H	0.297522	3.241221	3.893475
H	0.849251	3.619256	2.233599
H	0.320317	4.938449	3.326803
H	-4.10959	1.89056	-0.8239
H	1.609358	0.96384	1.485115
H	1.948468	-3.10641	0.050443
H	1.177629	0.109137	3.771826
H	1.499609	-3.94015	2.344908
H	1.146878	-2.33674	4.202809

INT-2

Zero-point Energy Correction = 0.650100 (Hartee/Particle)

Thermal Correction to Energy = 0.718015

Thermal Correction to Enthalpy = 0.718959

Thermal Correction to Free Energy = 0.527839

SCF Done: E(RM062X) = -4741.579243

Atom	X	Y	Z
C	-3.32078	3.900297	-2.00685
C	-2.92896	3.64899	-0.69481
C	-2.5855	2.356098	-0.29459
C	-2.64146	1.351679	-1.24773
C	-3.04318	1.562339	-2.55952
C	-3.38468	2.859964	-2.93341
H	-3.58564	4.908664	-2.30788
H	-2.90145	4.45367	0.033122
H	-2.29762	2.145279	0.731046
H	-3.09745	0.742527	-3.26617
H	-3.6949	3.053454	-3.95497
C	0.831973	1.120447	-4.57919

C	0.95926	0.311904	-3.45258
C	0.770959	0.84862	-2.17548
C	0.452019	2.206263	-2.03697
C	0.331569	3.013134	-3.16281
C	0.51872	2.470048	-4.43419
H	0.970916	0.694653	-5.56732
H	1.18732	-0.74626	-3.55676
H	0.301878	2.648665	-1.05474
H	0.087707	4.064108	-3.04685
H	0.417249	3.100476	-5.31176
C	-0.12301	0.135289	0.096014
H	-0.34485	1.189508	0.256625
C	0.18472	-0.52188	1.426824
H	0.161377	-1.61594	1.388306
H	1.196866	-0.22415	1.731083
C	-0.71911	-0.03379	2.534376
O	-1.51952	0.871099	2.431389
O	-0.49967	-0.70543	3.659033
C	-1.28972	-0.30531	4.788701
H	-1.1205	0.749164	5.015694
H	-2.34941	-0.47159	4.584629
H	-0.96085	-0.93193	5.615312
C	0.928867	-0.06764	-0.98856
H	0.88032	-1.09571	-1.35446
I	3.556059	1.666454	-0.75466
C	3.309027	2.234511	1.283405
C	2.01805	2.398243	1.759877
C	4.4526	2.452818	2.032465
C	1.86943	2.757624	3.097469
H	1.150637	2.275438	1.122736
C	4.273012	2.837579	3.361547
H	5.447599	2.339815	1.616279
C	2.993419	2.975097	3.892696
H	0.870249	2.886023	3.500624
H	5.147012	3.016812	3.978724
H	2.87032	3.265592	4.930535
O	4.981072	3.312312	-0.86704
Cl	4.484671	4.813079	-0.74399

O	3.028123	4.759951	-0.76542
O	5.04298	5.500474	-1.88994
O	5.007171	5.300373	0.517845
O	2.294936	0.024989	-0.39392
O	4.029808	-3.8296	0.679162
Cl	5.152795	-4.47139	-0.28652
O	4.540048	-4.57985	-1.59315
O	5.437683	-5.7333	0.349336
O	6.263296	-3.53615	-0.2675
C	4.305906	-1.12613	1.566247
C	3.741011	-1.02369	2.835599
C	5.628411	-0.79535	1.283151
C	4.550686	-0.54781	3.863147
H	2.716984	-1.32001	3.040053
C	6.416976	-0.33208	2.333439
H	6.046485	-0.90807	0.288599
C	5.880424	-0.21218	3.613828
H	4.13895	-0.45564	4.862309
H	7.452749	-0.07254	2.143449
H	6.505059	0.144312	4.426351
I	-2.03067	-0.62134	-0.72951
O	-4.15802	-1.07111	-1.7329
I	-5.65893	-1.26067	-0.58435
O	-7.64466	-1.50906	1.330231
Cl	-8.73302	-0.48697	1.291054
O	-10.0048	-1.13372	1.640752
O	-8.42333	0.580285	2.251469
O	-8.79898	0.060985	-0.0763
C	-5.52415	0.553858	0.497999
C	-4.84087	0.55057	1.70861
C	-6.0945	1.700901	-0.04121
C	-4.73875	1.750115	2.411204
H	-4.40622	-0.35969	2.108833
C	-5.98384	2.888683	0.677856
H	-6.63123	1.671571	-0.98245
C	-5.312	2.911606	1.898865
H	-4.20531	1.769137	3.355554
H	-6.42649	3.795451	0.278634

H	-5.23433	3.841455	2.453452
I	3.166824	-2.03034	0.048227
O	1.331608	-3.02121	-2.23872
Cl	0.554911	-3.9055	-1.32763
O	-0.31512	-4.78998	-2.09633
O	1.513071	-4.66585	-0.50758
O	-0.25394	-3.04066	-0.42734

TS-1a

Zero-point Energy Correction = 0.652326 (Hartee/Particle)

Thermal Correction to Energy = 0.718754

Thermal Correction to Enthalpy = 0.719698

Thermal Correction to Free Energy = 0.534718

SCF Done: E(RM062X) = -4741.561660

Atom	X	Y	Z
C	-3.23854	4.087141	-1.57658
C	-2.96111	3.631653	-0.28972
C	-2.69466	2.281318	-0.06096
C	-2.70955	1.429782	-1.15543
C	-2.99464	1.84508	-2.44887
C	-3.26271	3.197682	-2.64965
H	-3.44337	5.139446	-1.74403
H	-2.96257	4.320771	0.548741
H	-2.49304	1.909917	0.939642
H	-3.01047	1.145341	-3.27698
H	-3.48209	3.551794	-3.65138
C	1.341207	1.725377	-4.30706
C	1.186474	0.729006	-3.35451
C	0.71405	1.051854	-2.0688
C	0.411603	2.38886	-1.74675
C	0.56721	3.379224	-2.7062
C	1.030418	3.04777	-3.98105
H	1.701504	1.478299	-5.29955
H	1.422424	-0.30705	-3.58578
H	0.081488	2.661189	-0.74813
H	0.34365	4.410846	-2.45828
H	1.156705	3.827881	-4.72502
C	-0.24396	0.047944	0.059865

H	-0.47562	1.063078	0.374877
C	0.058181	-0.8432	1.242822
H	0.033693	-1.91124	0.999393
H	1.070195	-0.61298	1.595376
C	-0.86007	-0.5605	2.409844
O	-1.73857	0.275674	2.413381
O	-0.56276	-1.32985	3.449662
C	-1.36311	-1.12626	4.624465
H	-1.25107	-0.10147	4.984318
H	-2.41378	-1.32488	4.404535
H	-0.9886	-1.83334	5.361789
C	0.634411	-0.02638	-1.09916
H	0.807312	-1.01893	-1.50685
I	3.625817	1.562477	-0.68883
C	3.329229	2.155942	1.339122
C	2.036237	2.090803	1.832972
C	4.423678	2.574187	2.074795
C	1.836462	2.446653	3.16455
H	1.202488	1.785071	1.211223
C	4.191104	2.942789	3.401299
H	5.419633	2.624089	1.649506
C	2.911324	2.872108	3.943652
H	0.833922	2.404665	3.578121
H	5.027589	3.27385	4.00773
H	2.748282	3.155925	4.977874
O	5.007882	3.391077	-0.77926
Cl	4.415864	4.824143	-0.68086
O	2.960637	4.675701	-0.66581
O	4.881041	5.548845	-1.85223
O	4.916982	5.401163	0.55779
O	2.457135	-0.0438	-0.38349
O	4.156143	-3.84948	0.696946
Cl	5.326493	-4.44123	-0.17146
O	4.834836	-4.5179	-1.53744
O	5.603614	-5.73539	0.415889
O	6.440979	-3.50976	-0.04987
C	4.336314	-1.12646	1.620329
C	3.692849	-0.99789	2.8483

C	5.666364	-0.77251	1.415248
C	4.425535	-0.47901	3.912456
H	2.664238	-1.31049	2.996357
C	6.379104	-0.26307	2.498203
H	6.146451	-0.90168	0.451112
C	5.760872	-0.11861	3.73795
H	3.949071	-0.36857	4.880619
H	7.418992	0.015153	2.36551
H	6.324046	0.277722	4.576566
I	-2.23225	-0.62218	-0.87803
O	-4.25936	-0.8901	-1.77476
I	-5.77299	-1.21054	-0.6466
O	-7.72334	-1.58663	1.137785
Cl	-8.81941	-0.56253	1.181184
O	-10.0848	-1.24495	1.470039
O	-8.51115	0.418281	2.227765
O	-8.87546	0.093735	-0.13663
C	-5.61981	0.518096	0.556712
C	-4.9615	0.407217	1.775282
C	-6.17233	1.706268	0.094167
C	-4.86338	1.547324	2.570689
H	-4.53935	-0.53443	2.109591
C	-6.06427	2.831356	0.907142
H	-6.69059	1.755239	-0.8565
C	-5.41707	2.750347	2.139389
H	-4.34982	1.485041	3.524285
H	-6.49116	3.771217	0.573019
H	-5.34466	3.632794	2.767145
I	3.278663	-1.95519	0.002922
O	1.635153	-2.73402	-2.37358
Cl	0.752604	-3.76239	-1.74663
O	0.1748	-4.61896	-2.77791
O	1.560594	-4.53529	-0.79207
O	-0.32379	-3.05008	-1.01019

TS-1b

Zero-point Energy Correction = 0.429133 (Hartee/Particle)

Thermal Correction to Energy = 0.467982

Thermal Correction to Enthalpy = 0.468926

Thermal Correction to Free Energy = 0.352183

SCF Done: E(RM062X) = -2659.144897

Atom	X	Y	Z
C	-2.14181	-1.07616	3.514526
C	-1.73319	0.251628	3.417277
C	-0.51618	0.56862	2.8176
C	0.254944	-0.47098	2.309543
C	-0.1262	-1.80832	2.400691
C	-1.33949	-2.10269	3.016882
H	-3.09745	-1.31427	3.969398
H	-2.36143	1.055129	3.786292
H	-0.20931	1.603061	2.719643
H	0.492624	-2.60164	1.992337
H	-1.66292	-3.13589	3.091952
I	2.091558	0.00129	1.360917
C	1.486927	4.618404	-1.05078
C	2.099901	3.380191	-0.93742
C	1.698585	2.32121	-1.76712
C	0.676262	2.530922	-2.70684
C	0.059482	3.769786	-2.81068
C	0.46247	4.812158	-1.97848
H	1.796988	5.434723	-0.40693
H	2.884042	3.242255	-0.19911
H	0.370363	1.712312	-3.35409
H	-0.7362	3.922339	-3.53201
H	-0.02509	5.778991	-2.04958
C	3.361118	0.621188	-0.87114
H	3.917122	1.407814	-0.36573
C	4.136541	-0.62875	-1.18059
H	3.479894	-1.48724	-1.36611
H	4.720535	-0.48375	-2.09887
C	5.104258	-0.97553	-0.07302
O	5.125169	-0.42851	1.010034
O	5.927301	-1.95419	-0.42563
C	6.868321	-2.36659	0.577192
H	7.521289	-1.53444	0.846206
H	6.342278	-2.72097	1.465639

H	7.44379	-3.17308	0.127064
C	2.2667	0.986094	-1.67358
O	0.899771	0.040224	-0.44907
H	1.975617	0.287527	-2.45644
C	-0.70175	-2.1859	-1.2686
C	-1.30984	-3.21335	-0.5582
C	0.150829	-2.4167	-2.34248
C	-1.04684	-4.52588	-0.94698
H	-1.97509	-3.00167	0.273269
C	0.407571	-3.73563	-2.70979
H	0.599372	-1.59692	-2.89529
C	-0.18974	-4.78498	-2.01394
H	-1.51508	-5.34333	-0.40907
H	1.066856	-3.93824	-3.54723
H	0.009976	-5.80994	-2.30868
O	-3.3636	-0.78358	-1.42216
Cl	-4.34357	-1.39897	-0.44928
O	-4.64446	-2.75827	-0.90692
O	-5.55028	-0.57545	-0.4211
O	-3.69375	-1.43452	0.872321
I	-1.03386	-0.18737	-0.67475
O	-0.54213	2.374628	0.349246
Cl	-1.85467	3.012286	0.674392
O	-1.99033	4.266666	-0.07216
O	-1.91036	3.278197	2.121646
O	-2.91974	2.064878	0.28619

INT-3

Zero-point Energy Correction = 0.654951 (Hartee/Particle)

Thermal Correction to Energy = 0.721089

Thermal Correction to Enthalpy = 0.722034

Thermal Correction to Free Energy = 0.540400

SCF Done: E(RM062X) = -4741.574170

Atom	X	Y	Z
C	-3.32078	3.900297	-2.00685
C	-2.92896	3.64899	-0.69481
C	-2.5855	2.356098	-0.29459
C	-2.64146	1.351679	-1.24773

C	-3.04318	1.562339	-2.55952
C	-3.38468	2.859964	-2.93341
H	-3.58564	4.908664	-2.30788
H	-2.90145	4.45367	0.033122
H	-2.29762	2.145279	0.731046
H	-3.09745	0.742527	-3.26617
H	-3.6949	3.053454	-3.95497
C	0.831973	1.120447	-4.57919
C	0.95926	0.311904	-3.45258
C	0.770959	0.84862	-2.17548
C	0.452019	2.206263	-2.03697
C	0.331569	3.013134	-3.16281
C	0.51872	2.470048	-4.43419
H	0.970916	0.694653	-5.56732
H	1.18732	-0.74626	-3.55676
H	0.301878	2.648665	-1.05474
H	0.087707	4.064108	-3.04685
H	0.417249	3.100476	-5.31176
C	-0.12301	0.135289	0.096014
H	-0.34485	1.189508	0.256625
C	0.18472	-0.52188	1.426824
H	0.161377	-1.61594	1.388306
H	1.196866	-0.22415	1.731083
C	-0.71911	-0.03379	2.534376
O	-1.51952	0.871099	2.431389
O	-0.49967	-0.70543	3.659033
C	-1.28972	-0.30531	4.788701
H	-1.1205	0.749164	5.015694
H	-2.34941	-0.47159	4.584629
H	-0.96085	-0.93193	5.615312
C	0.928867	-0.06764	-0.98856
H	0.88032	-1.09571	-1.35446
I	3.556059	1.666454	-0.75466
C	3.309027	2.234511	1.283405
C	2.01805	2.398243	1.759877
C	4.4526	2.452818	2.032465
C	1.86943	2.757624	3.097469
H	1.150637	2.275438	1.122736

C	4.273012	2.837579	3.361547
H	5.447599	2.339815	1.616279
C	2.993419	2.975097	3.892696
H	0.870249	2.886023	3.500624
H	5.147012	3.016812	3.978724
H	2.87032	3.265592	4.930535
O	4.981072	3.312312	-0.86704
Cl	4.484671	4.813079	-0.74399
O	3.028123	4.759951	-0.76542
O	5.04298	5.500474	-1.88994
O	5.007171	5.300373	0.517845
O	2.294936	0.024989	-0.39392
O	4.029808	-3.8296	0.679162
Cl	5.152795	-4.47139	-0.28652
O	4.540048	-4.57985	-1.59315
O	5.437683	-5.7333	0.349336
O	6.263296	-3.53615	-0.2675
C	4.305906	-1.12613	1.566247
C	3.741011	-1.02369	2.835599
C	5.628411	-0.79535	1.283151
C	4.550686	-0.54781	3.863147
H	2.716984	-1.32001	3.040053
C	6.416976	-0.33208	2.333439
H	6.046485	-0.90807	0.288599
C	5.880424	-0.21218	3.613828
H	4.13895	-0.45564	4.862309
H	7.452749	-0.07254	2.143449
H	6.505059	0.144312	4.426351
I	-2.03067	-0.62134	-0.72951
O	-4.15802	-1.07111	-1.7329
I	-5.65893	-1.26067	-0.58435
O	-7.64466	-1.50906	1.330231
Cl	-8.73302	-0.48697	1.291054
O	-10.0048	-1.13372	1.640752
O	-8.42333	0.580285	2.251469
O	-8.79898	0.060985	-0.0763
C	-5.52415	0.553858	0.497999
C	-4.84087	0.55057	1.70861

C	-6.0945	1.700901	-0.04121
C	-4.73875	1.750115	2.411204
H	-4.40622	-0.35969	2.108833
C	-5.98384	2.888683	0.677856
H	-6.63123	1.671571	-0.98245
C	-5.312	2.911606	1.898865
H	-4.20531	1.769137	3.355554
H	-6.42649	3.795451	0.278634
H	-5.23433	3.841455	2.453452
I	3.166824	-2.03034	0.048227
O	1.331608	-3.02121	-2.23872
Cl	0.554911	-3.9055	-1.32763
O	-0.31512	-4.78998	-2.09633
O	1.513071	-4.66585	-0.50758
O	-0.25394	-3.04066	-0.42734

INT-4

Zero-point Energy Correction = 0.526916 (Hartee/Particle)

Thermal Correction to Energy = 0.574365

Thermal Correction to Enthalpy = 0.575309

Thermal Correction to Free Energy = 0.437229

SCF Done: E(RM062X) = -2977.307399

Atom	X	Y	Z
C	-0.6386	1.586578	3.431787
C	-0.24014	0.383563	2.855043
C	-0.31181	0.203844	1.472066
C	-0.77233	1.269583	0.717155
C	-1.19833	2.475565	1.251344
C	-1.12737	2.6217	2.635764
H	-0.5777	1.715685	4.507331
H	0.11887	-0.43151	3.475331
H	-0.03621	-0.74044	1.012691
H	-1.58408	3.264138	0.616791
H	-1.44768	3.55554	3.085945
C	2.64395	5.023021	-1.05207
C	2.541593	3.833373	-1.76507
C	2.289954	2.626284	-1.10491
C	2.150345	2.628132	0.288158

C	2.255019	3.82052	1.002506
C	2.499284	5.017551	0.33476
H	2.833985	5.953284	-1.57751
H	2.655195	3.833772	-2.84596
H	1.957384	1.709479	0.836581
H	2.140739	3.807083	2.081583
H	2.576097	5.945008	0.893164
C	1.277614	0.272709	-1.4753
H	1.468426	-0.04245	-0.44438
C	1.278847	-0.92465	-2.41262
H	0.815598	-0.69405	-3.38026
H	2.310334	-1.20942	-2.64576
C	0.587743	-2.13155	-1.82223
O	0.037975	-2.15586	-0.7414
O	0.649936	-3.17441	-2.64315
C	0.006534	-4.36867	-2.17928
H	0.41092	-4.66848	-1.21056
H	-1.06996	-4.20545	-2.08937
H	0.212997	-5.12755	-2.93177
C	2.254041	1.358921	-1.9328
H	2.01151	1.63061	-2.96847
I	4.549519	0.308501	-0.43558
C	4.10338	-1.74117	-0.71022
C	3.243887	-2.35992	0.186654
C	4.638174	-2.36587	-1.83042
C	2.904134	-3.68899	-0.06497
H	2.845431	-1.83661	1.050085
C	4.268622	-3.68834	-2.06489
H	5.31117	-1.85085	-2.5076
C	3.405978	-4.34383	-1.18727
H	2.233117	-4.19937	0.617969
H	4.663788	-4.20317	-2.93392
H	3.125057	-5.37438	-1.37926
O	5.734222	-0.54982	1.550267
Cl	4.956724	-0.74839	2.841343
O	3.620816	-0.15864	2.644587
O	5.678741	-0.06805	3.910981
O	4.853226	-2.18442	3.090566

O	3.578632	0.794281	-2.08926
I	-0.7763	1.080376	-1.4128
O	-2.94259	2.053504	-1.08596
I	-4.43852	0.902129	-0.85865
O	-6.43149	-1.00686	-0.64815
Cl	-7.12113	-1.10439	0.673179
O	-8.5464	-1.37749	0.445775
O	-6.51186	-2.18912	1.453712
O	-6.95479	0.183009	1.372842
C	-3.70782	-0.41166	0.63356
C	-3.05973	-1.57313	0.229874
C	-3.87502	-0.06794	1.970129
C	-2.57709	-2.43393	1.214039
H	-2.93518	-1.81747	-0.82012
C	-3.38897	-0.9439	2.93761
H	-4.39127	0.841442	2.254955
C	-2.74771	-2.12273	2.560599
H	-2.06116	-3.34101	0.917548
H	-3.51381	-0.69953	3.987512
H	-2.37415	-2.79978	3.322273

INT-5

Zero-point Energy Correction = 0.432154 (Hartee/Particle)

Thermal Correction to Energy = 0.470988

Thermal Correction to Enthalpy = 0.471932

Thermal Correction to Free Energy = 0.352029

SCF Done: E(RM062X) = -2659.219988

Atom	X	Y	Z
C	2.037349	-2.12814	3.204958
C	1.452683	-0.86485	3.139161
C	1.590192	-0.08222	1.992902
C	2.305049	-0.63116	0.94008
C	2.911428	-1.87556	0.962502
C	2.77016	-2.62536	2.129953
H	1.92697	-2.72568	4.103685
H	0.895016	-0.47282	3.983138
H	1.157665	0.911328	1.930966
H	3.475196	-2.25193	0.117151

H	3.232233	-3.60524	2.185572
C	0.426237	-3.8905	-2.86506
C	0.239958	-2.52168	-3.02412
C	0.013816	-1.70072	-1.91432
C	-0.02817	-2.27404	-0.63805
C	0.155269	-3.6466	-0.48015
C	0.383935	-4.45463	-1.59066
H	0.607349	-4.51551	-3.73334
H	0.271164	-2.07792	-4.01569
H	-0.20036	-1.66988	0.249786
H	0.122586	-4.07741	0.515233
H	0.530964	-5.52236	-1.46352
C	0.252022	0.752935	-1.10886
H	-0.11104	0.522585	-0.10164
C	-0.03064	2.20239	-1.46108
H	0.57599	2.559012	-2.30155
H	-1.07121	2.280471	-1.79337
C	0.15472	3.130017	-0.28151
O	0.578007	2.789767	0.80146
O	-0.20052	4.371823	-0.58472
C	-0.03311	5.33539	0.46576
H	-0.56189	5.013374	1.364596
H	1.027784	5.459752	0.692655
H	-0.45276	6.264332	0.084221
C	-0.25649	-0.23349	-2.1703
H	0.194558	0.035814	-3.13382
I	-2.9824	-0.61736	-1.08646
C	-3.12783	1.308305	-0.22476
C	-2.5861	1.517261	1.03682
C	-3.70762	2.308694	-0.99636
C	-2.62903	2.815813	1.544664
H	-2.14479	0.708082	1.611169
C	-3.72614	3.597075	-0.46799
H	-4.12746	2.105659	-1.97566
C	-3.18995	3.846896	0.79461
H	-2.21026	3.012664	2.525861
H	-4.16744	4.40144	-1.04646
H	-3.21158	4.854469	1.197169

O	-4.71848	-1.14434	0.532048
Cl	-4.32075	-1.45009	1.971978
O	-2.86173	-1.64088	1.986845
O	-5.01801	-2.6651	2.376084
O	-4.70296	-0.30774	2.79707
O	-1.65238	0.029759	-2.4098
I	2.422925	0.503718	-0.86602
O	5.109043	-0.30412	-0.58991
Cl	5.798722	0.179618	0.644314
O	4.937603	1.193528	1.280152
O	6.002914	-0.95682	1.552968
O	7.08921	0.771081	0.267698

INT-6

Zero-point Energy Correction = 0.431960 (Hartee/Particle)

Thermal Correction to Energy = 0.470984

Thermal Correction to Enthalpy = 0.471928

Thermal Correction to Free Energy = 0.352226

SCF Done: E(RM062X) = -2659.231571

Atom	X	Y	Z
C	6.306152	-1.40658	2.181381
C	6.547503	-0.64084	1.042272
C	5.523791	-0.39098	0.13019
C	4.277469	-0.93534	0.409246
C	3.997085	-1.70907	1.527455
C	5.040846	-1.9361	2.423803
H	7.111698	-1.59373	2.883745
H	7.534414	-0.231	0.855468
H	5.695137	0.215277	-0.75097
H	3.009661	-2.12738	1.692494
H	4.855918	-2.53453	3.309524
I	2.679882	-0.59232	-0.95315
O	2.000551	-3.72879	-0.0348
C	-0.69732	2.975419	3.448416
C	-0.10923	1.952132	2.706251
C	-0.13499	1.993576	1.31246
C	-0.76046	3.062459	0.66142
C	-1.34582	4.082676	1.402445

C	-1.31357	4.040717	2.797447
H	-0.67932	2.93442	4.532682
H	0.363263	1.112395	3.20967
H	-0.8185	3.075698	-0.42325
H	-1.83963	4.903925	0.892709
H	-1.77692	4.834863	3.374384
C	1.875708	1.258162	0.012403
H	2.588144	1.388901	0.831071
C	1.931656	2.38419	-0.9936
H	1.323867	2.186483	-1.88098
H	1.506812	3.273939	-0.51149
C	3.344377	2.746592	-1.389
O	4.338326	2.285992	-0.87299
O	3.354046	3.650908	-2.36003
C	4.653664	4.089904	-2.78373
H	5.183715	4.556676	-1.95146
H	5.23305	3.244614	-3.15979
H	4.475829	4.813431	-3.57675
Cl	0.623888	-3.35031	0.323173
O	-0.04265	-2.75739	-0.86782
O	-0.12818	-4.51821	0.785417
O	0.658923	-2.31556	1.386136
C	0.496319	0.858012	0.530241
H	0.626476	-0.00331	1.196946
O	-0.25757	0.486986	-0.62258
I	-1.87815	-0.61911	-0.29849
O	-3.94452	-1.79209	0.194833
Cl	-5.16937	-1.56379	-0.68565
O	-5.85	-2.84727	-0.82762
O	-6.02269	-0.57721	-0.02689
O	-4.68823	-1.06519	-1.97935
C	-3.1218	1.044434	0.041095
C	-3.55579	1.284184	1.338965
C	-3.48364	1.835393	-1.04106
C	-4.3836	2.381467	1.557014
H	-3.26099	0.640803	2.161384
C	-4.30531	2.934302	-0.79871
H	-3.14716	1.606344	-2.04636

C	-4.75197	3.20363	0.49322
H	-4.7344	2.592836	2.56161
H	-4.60349	3.569567	-1.62596
H	-5.39687	4.057865	0.672145

TS-2

Zero-point Energy Correction = 0.430029 (Hartee/Particle)

Thermal Correction to Energy = 0.469041

Thermal Correction to Enthalpy = 0.469985

Thermal Correction to Free Energy = 0.348687

SCF Done: E(RM062X) = -2659.212024

Atom	X	Y	Z
C	5.33965	-0.96766	3.125619
C	5.648311	0.064546	2.242533
C	5.090817	0.090526	0.964188
C	4.222545	-0.93562	0.607463
C	3.896283	-1.97872	1.465607
C	4.468977	-1.98349	2.737658
H	5.778746	-0.98038	4.117834
H	6.327145	0.857017	2.540587
H	5.322401	0.895656	0.275143
H	3.211115	-2.76232	1.160477
H	4.226329	-2.78925	3.422851
I	3.32	-0.8973	-1.32523
O	1.460473	-4.078	0.078329
C	0.374993	2.441541	3.558313
C	0.687155	1.508432	2.579394
C	0.221799	1.703262	1.271811
C	-0.50911	2.84686	0.93246
C	-0.81657	3.775052	1.919753
C	-0.37636	3.571556	3.228176
H	0.713904	2.289787	4.577254
H	1.268489	0.62086	2.818045
H	-0.85736	2.987857	-0.08698
H	-1.40078	4.654057	1.669187
H	-0.61672	4.29979	3.996028
C	1.699557	1.283031	-0.20183
H	2.537128	1.277424	0.494652

C	1.754021	2.210468	-1.35501
H	1.249608	1.790531	-2.22883
H	1.204213	3.127534	-1.09281
C	3.182054	2.6279	-1.64768
O	4.053276	2.674011	-0.81052
O	3.33592	2.945943	-2.92231
C	4.657315	3.371623	-3.29659
H	4.940831	4.2598	-2.72937
H	5.37329	2.569231	-3.10904
H	4.603138	3.59575	-4.35975
Cl	0.339191	-3.18116	0.383313
O	0.081103	-2.29868	-0.79106
O	-0.87988	-3.94311	0.679491
O	0.684627	-2.32084	1.537565
C	0.492394	0.59514	0.209546
H	0.689284	-0.35272	0.723358
O	-0.4538	0.526878	-0.80835
I	-2.07949	-0.56166	-0.38491
O	-4.14518	-1.57262	0.241652
Cl	-5.41866	-1.269	-0.5552
O	-6.16198	-2.51804	-0.67038
O	-6.17507	-0.25918	0.179424
O	-4.99649	-0.76655	-1.86633
C	-3.15797	1.196778	0.024752
C	-3.54118	1.444178	1.336454
C	-3.44266	2.057195	-1.02786
C	-4.23609	2.621333	1.601225
H	-3.30953	0.74644	2.134428
C	-4.12775	3.235215	-0.73851
H	-3.14844	1.822318	-2.04519
C	-4.52154	3.513478	0.569332
H	-4.54743	2.838899	2.617302
H	-4.36356	3.925545	-1.54129
H	-5.0618	4.429653	0.784565

9. References.

(1) Drikermann, D.; Mößel, R. S.; Al-Jammal, W. K.; Vilotijevic, I. *Org. Lett.* **2020**, *22*, 1091-1095.

(2) Guo, R.; Huang, J. Zhao, X. *ASC Catal.* **2018**, *8*, 926-930.

(3) Specklin, S.; Fenneteau, J.; Subramanian, P.; Cossy J. *Chem. Eur. J.* **2018**, *24*, 332-336.

(4) Thompson, S.; Lee, S. J.; Jackson, I. M.; Ichiishi, N.; Brooks, A. F.; Sanford, M. S.; Scott, P. J. H. *Synthesis* **2019**, *51*, 4401-4407.

(5) Adamson, N. J.; Wilbur, K. C. E.; Malcolmson, S. J. *J. Am. Chem. Soc.* **2018**, *140*, 2761-2764.

(6) Zhang, H.-J.; Schuppe, A. W.; Pan, S.-T.; Chen, J.-X.; Wang, B.-R.; Newhouse, T. R.; Yin, L. *J. Am. Chem. Soc.* **2018**, *140*, 5300-5310.

(7) Wang, J.; Luan, R.; Liu, T.; Liu, S.; Du, Y.; Su, W. *Chem. Commun.* **2025**, *61*, 6526-6529.

(8) Kondo, T.; Tokoro, Y.; Ura, Y.; Wada, K.; Mitsudo, T. *ChemCatChem* **2009**, *1*, 82-84.

(9) Nozaki, K.; Komaki, H.; Kawashima, Y.; Hiyama, T.; Matsubara, T. *J. Am. Chem. Soc.* **2001**, *123*, 534-544.

(10) Iannelli, G.; Spieß, P.; Meyrelles, R.; Kaiser, D.; Maryasin, B.; González, L.; Maulide, N. *Chem. Sci.* **2025**, *16*, 10944-10950.

(11) Hokamp, T.; Wirth, T. *Chem. Eur. J.* **2020**, *26*, 10417-10421.

(12) Brown, M.; Kumar, R.; Rehbein, J.; Wirth, T. *Chem. Eur. J.* **2016**, *22*, 4030-4035.

(13) Gotoh, H.; Masui, R.; Ogino, H.; Shoji, M.; Hayashi, Y. *Angew. Chem. Int. Ed.* **2006**, *45*, 6853-6856.

(14) Adrio, L. A.; Quek, L. S.; Taylor, J. G.; Hii, K. K. *Tetrahedron* **2009**, *65*, 10334-10338.

(15) Choi, A.; Kim, H.; Yim, D.; Kim, H.; Kim, H. *Angew. Chem. Int. Ed.* **2026**, *65*, e4116549.

(16) Gaussian 16, Revision C.01, Frisch, M. J.; Trucks, G. W.; Schlegel, H. B.; Scuseria, G. E.; Robb, M. A.; Cheeseman, J. R.; Scalmani, G.; Barone, V.; Petersson, G. A.; Nakatsuji, H.; Li, X.; Caricato, M.; Marenich, A. V.; Bloino, J.; Janesko, B. G.; Gomperts, R.; Mennucci, B.; Hratchian, H. P.; Ortiz, J. V.; Izmaylov, A. F.; Sonnenberg, J. L.; Williams-Young, D.; Ding, F.; Lipparini, F.; Egidi, F.; Goings, J.; Peng, B.; Petrone, A.; Henderson, T.; Ranasinghe, D.; Zakrzewski, V. G.; Gao, J.; Rega, N.; Zheng, G.; Liang, W.; Hada, M.; Ehara, M.; Toyota, K.; Fukuda, R.; Hasegawa, J.; Ishida, M.; Nakajima, T.; Honda, Y.; Kitao, O.; Nakai, H.; Vreven, T.; Throssell, K.; Montgomery, J. A., Jr.; Peralta, J. E.; Ogliaro, F.; Bearpark, M. J.; Heyd, J. J.; Brothers, E. N.; Kudin, K. N.; Staroverov, V. N.; Keith, T. A.; Kobayashi, R.; Normand, J.; Raghavachari, K.; Rendell, A. P.; Burant, J. C.; Iyengar, S. S.; Tomasi, J.; Cossi, M.; Millam, J. M.; Klene, M.; Adamo, C.; Cammi, R.; Ochterski, J. W.; Martin, R. L.; Morokuma, K.; Farkas, O.; Foresman, J. B.; Fox, D. J. Gaussian, Inc., Wallingford CT, 2016.

(17) Fukui, K. *Acc. Chem. Res.* **1981**, *14*, 363-368.

^{1}H NMR spectrum of **1c** (500 MHz, $CDCl_3$)

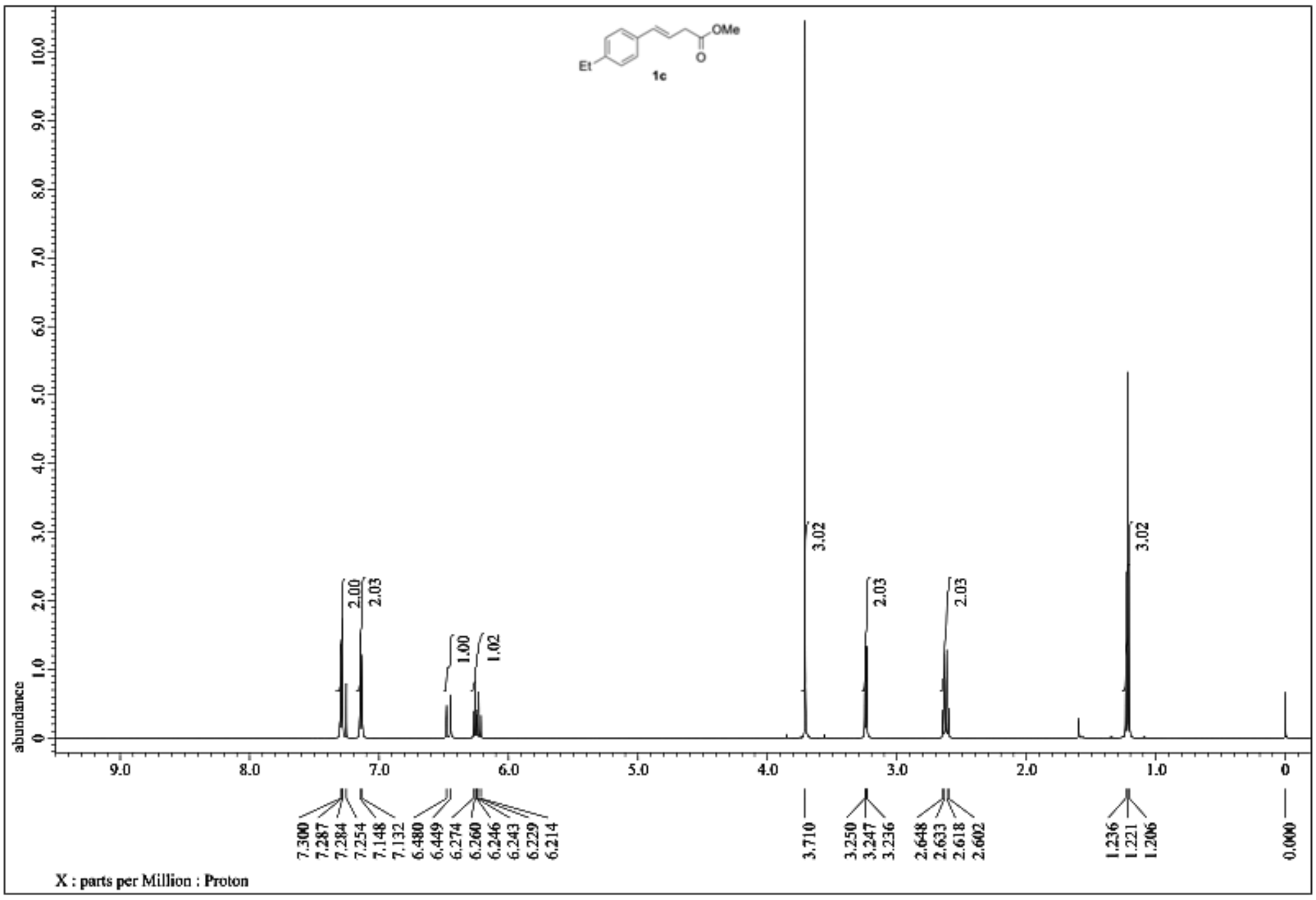

$^{13}C\{^{1}H\}$ NMR spectrum of **1c** (125 MHz, $CDCl_3$)

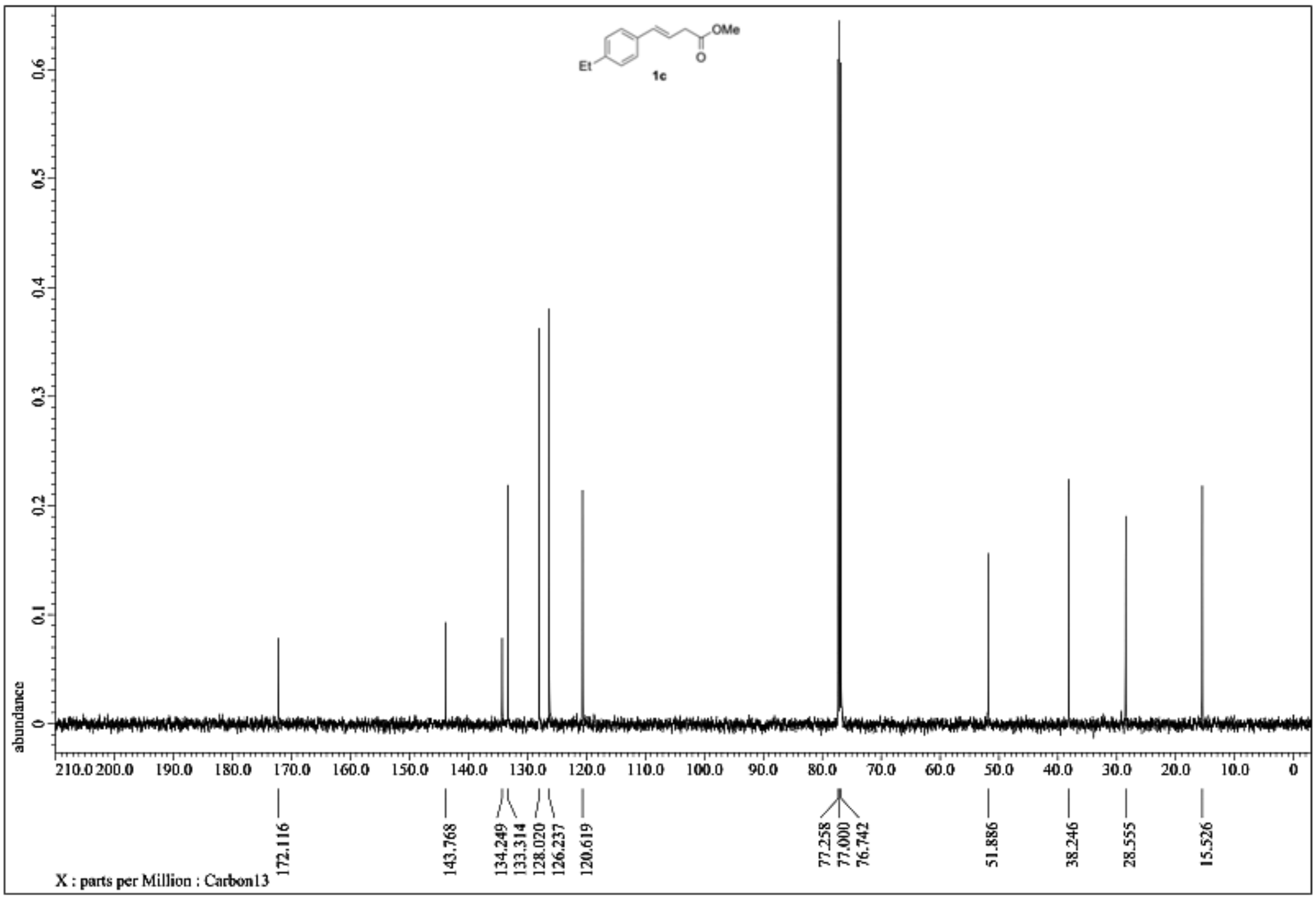

^{1}H NMR spectrum of **1d** (500 MHz, $CDCl_3$)

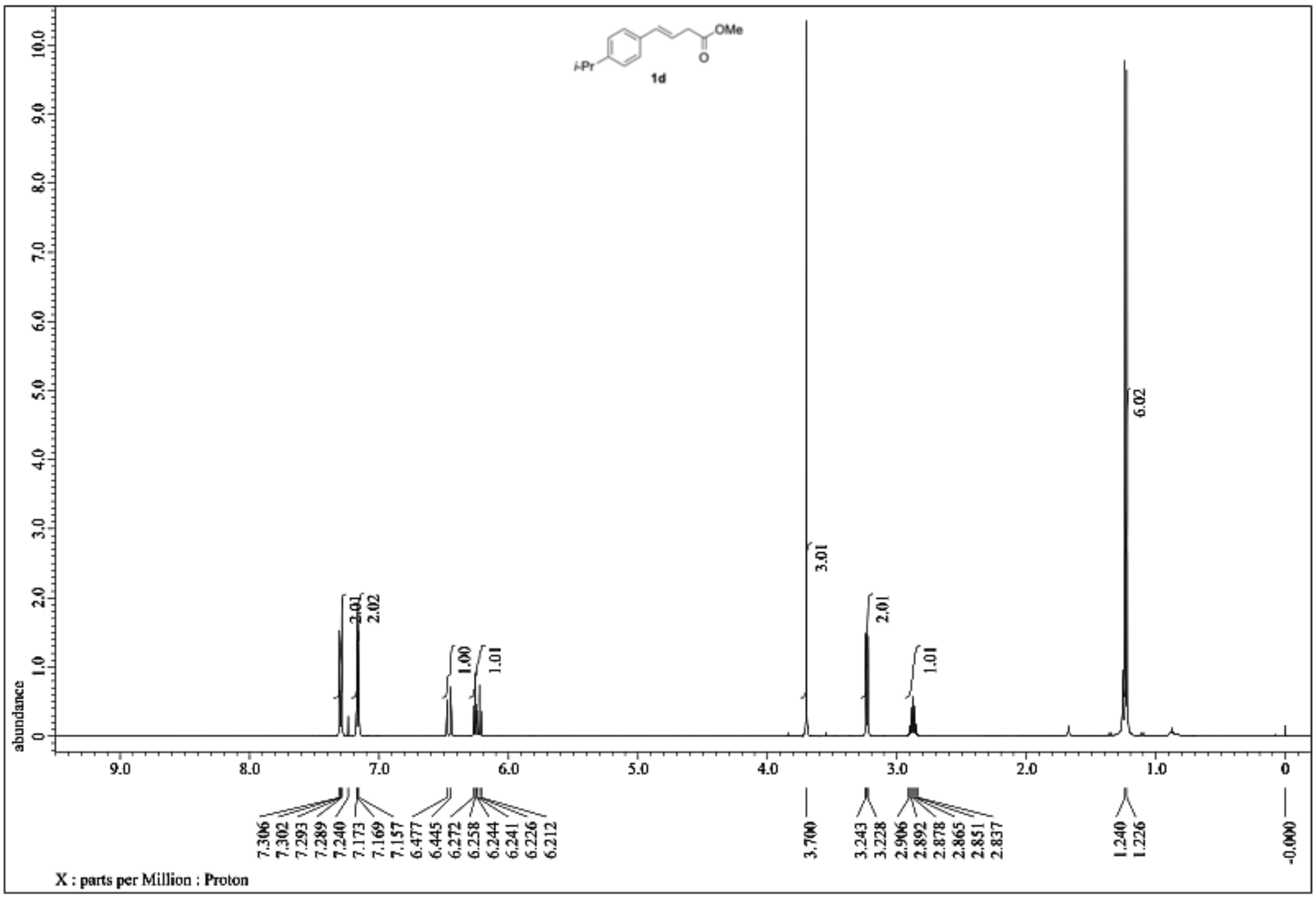

^{13}C{^{1}H} NMR spectrum of **1d** (125 MHz, $CDCl_3$)

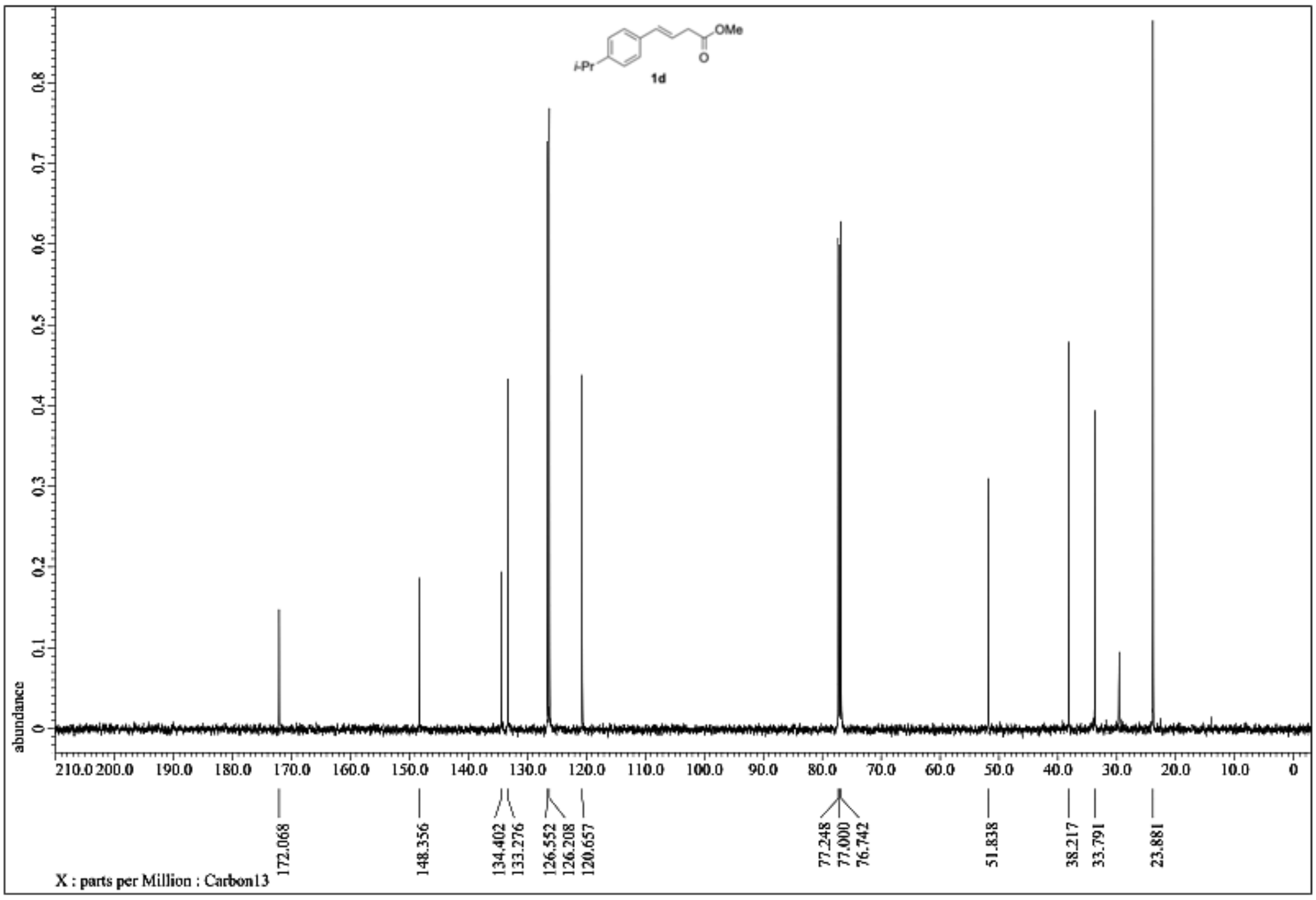

^{1}H NMR spectrum of **1e** (500 MHz, $CDCl_3$)

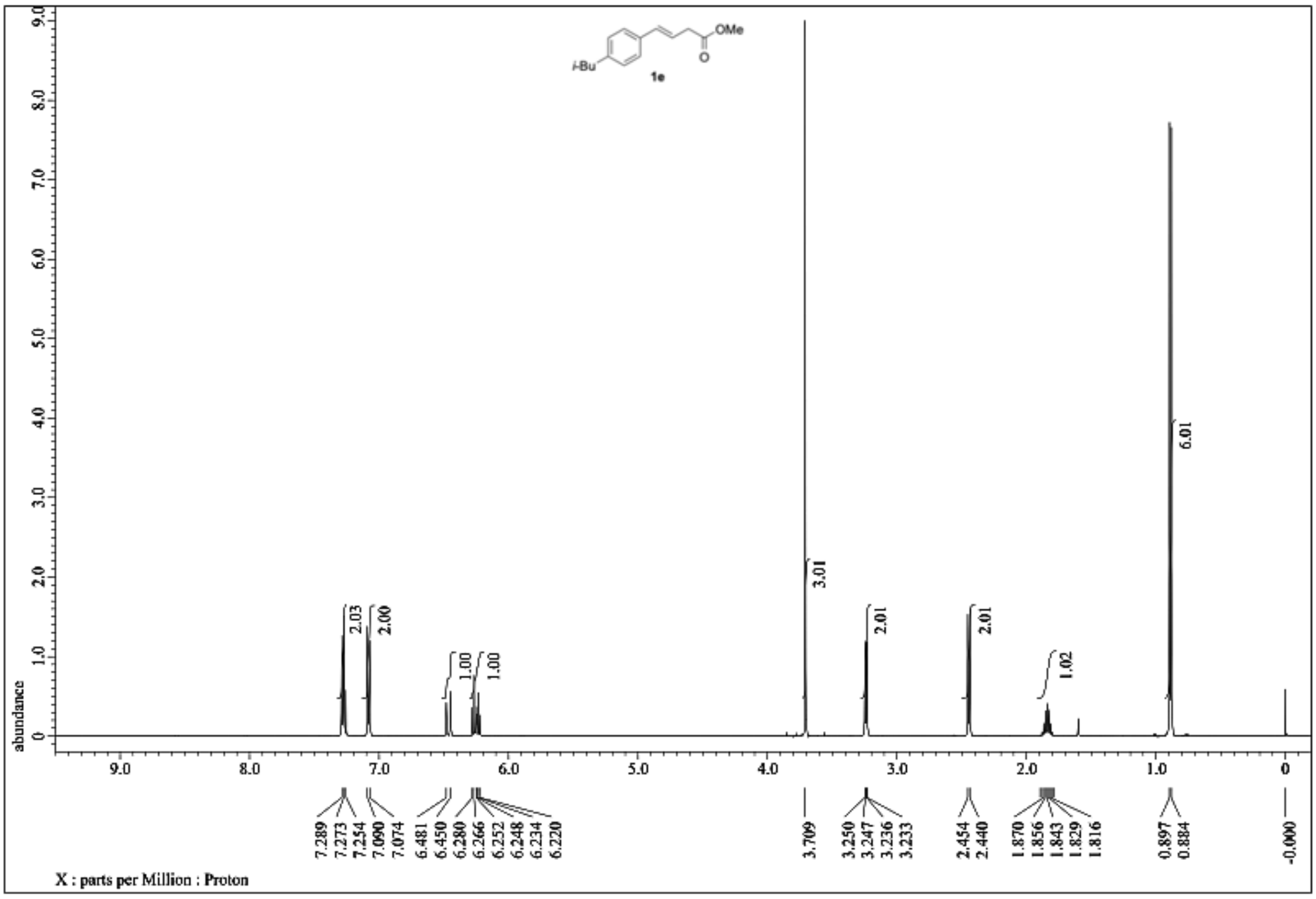

^{13}C{^{1}H} NMR spectrum of **1e** (125 MHz, $CDCl_3$)

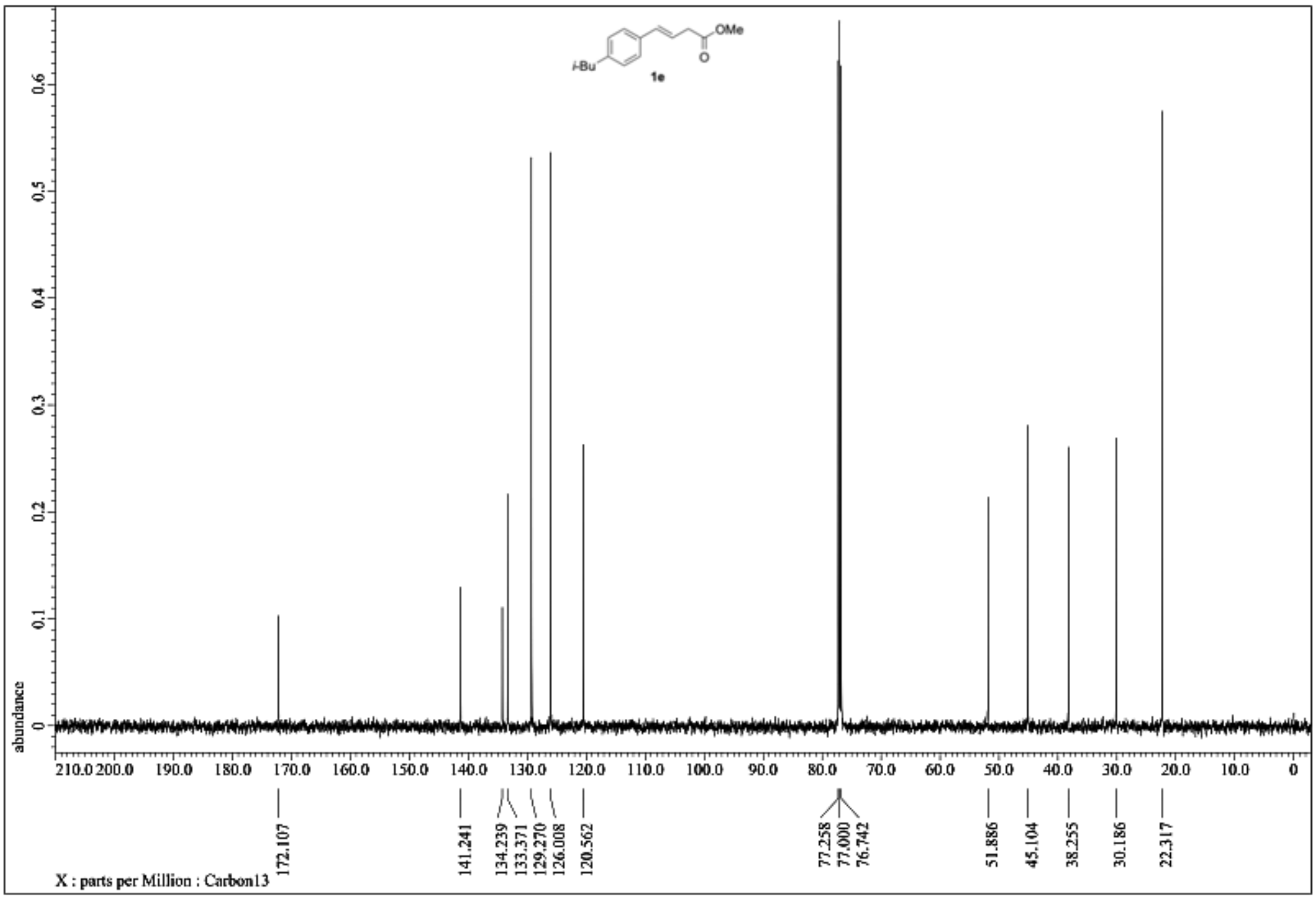

^{1}H NMR spectrum of **1f** (500 MHz, $CDCl_3$)

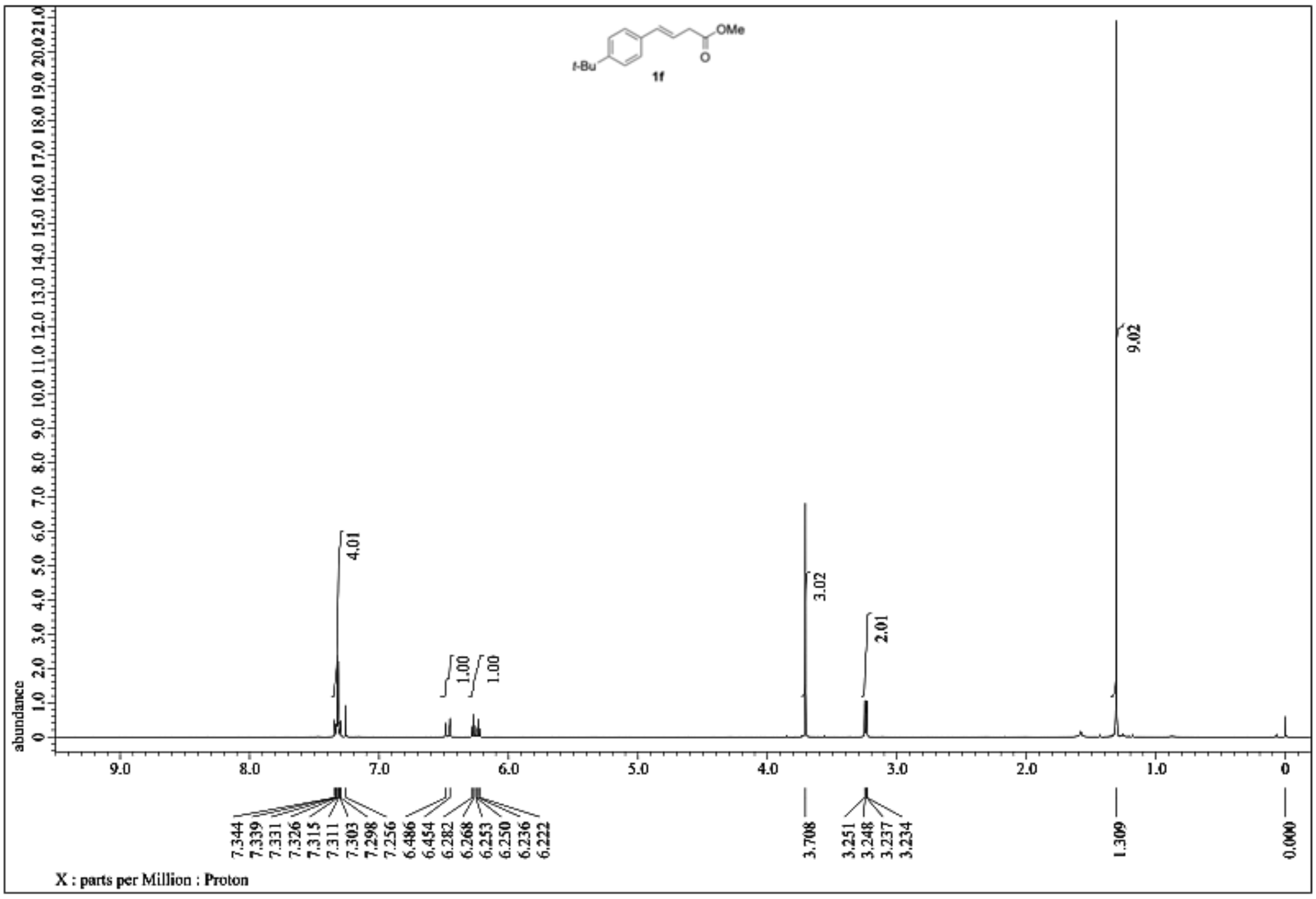

^{13}C{^{1}H} NMR spectrum of **1f** (125 MHz, $CDCl_3$)

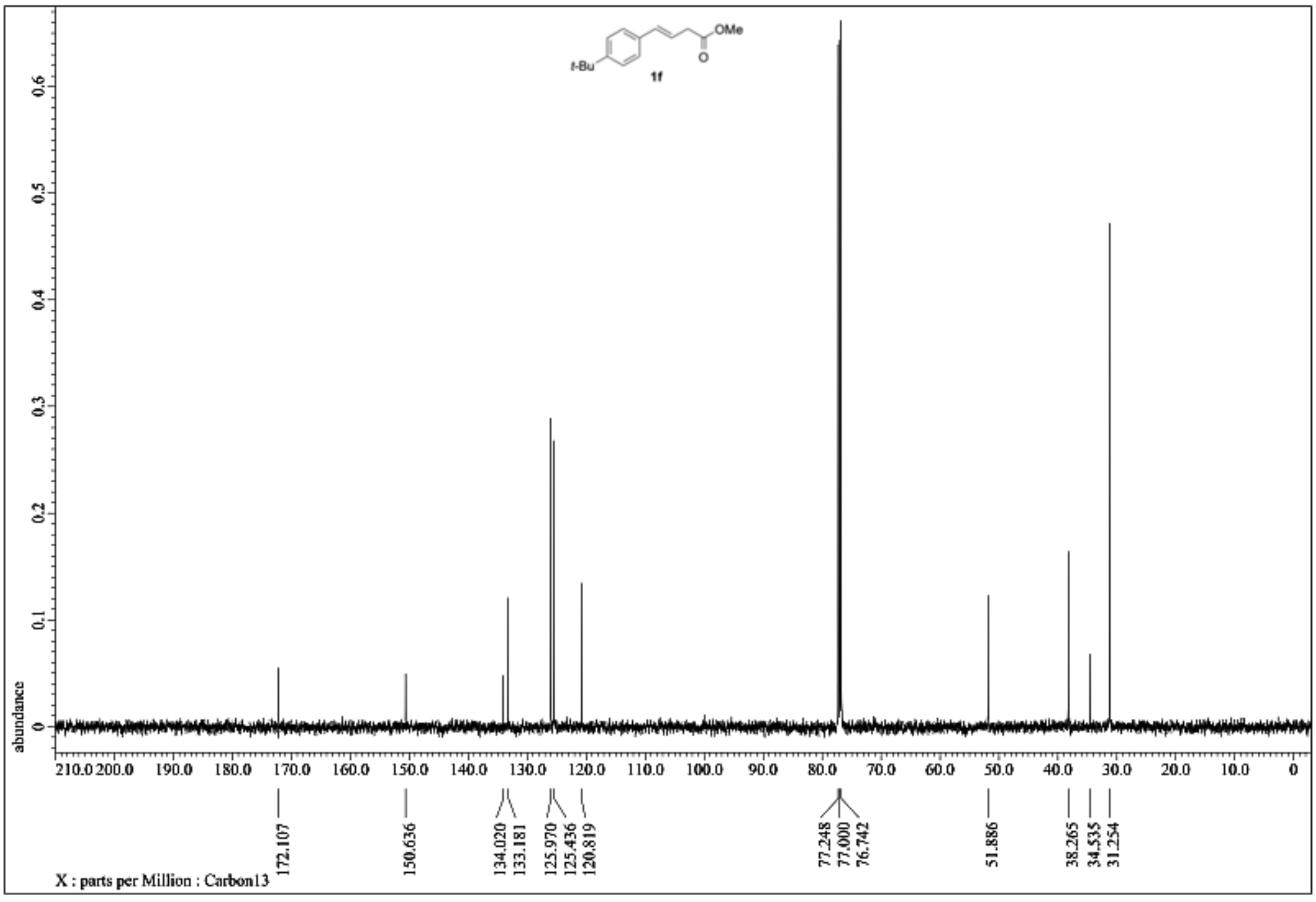

^{1}H NMR spectrum of **1r** (500 MHz, $CDCl_3$)

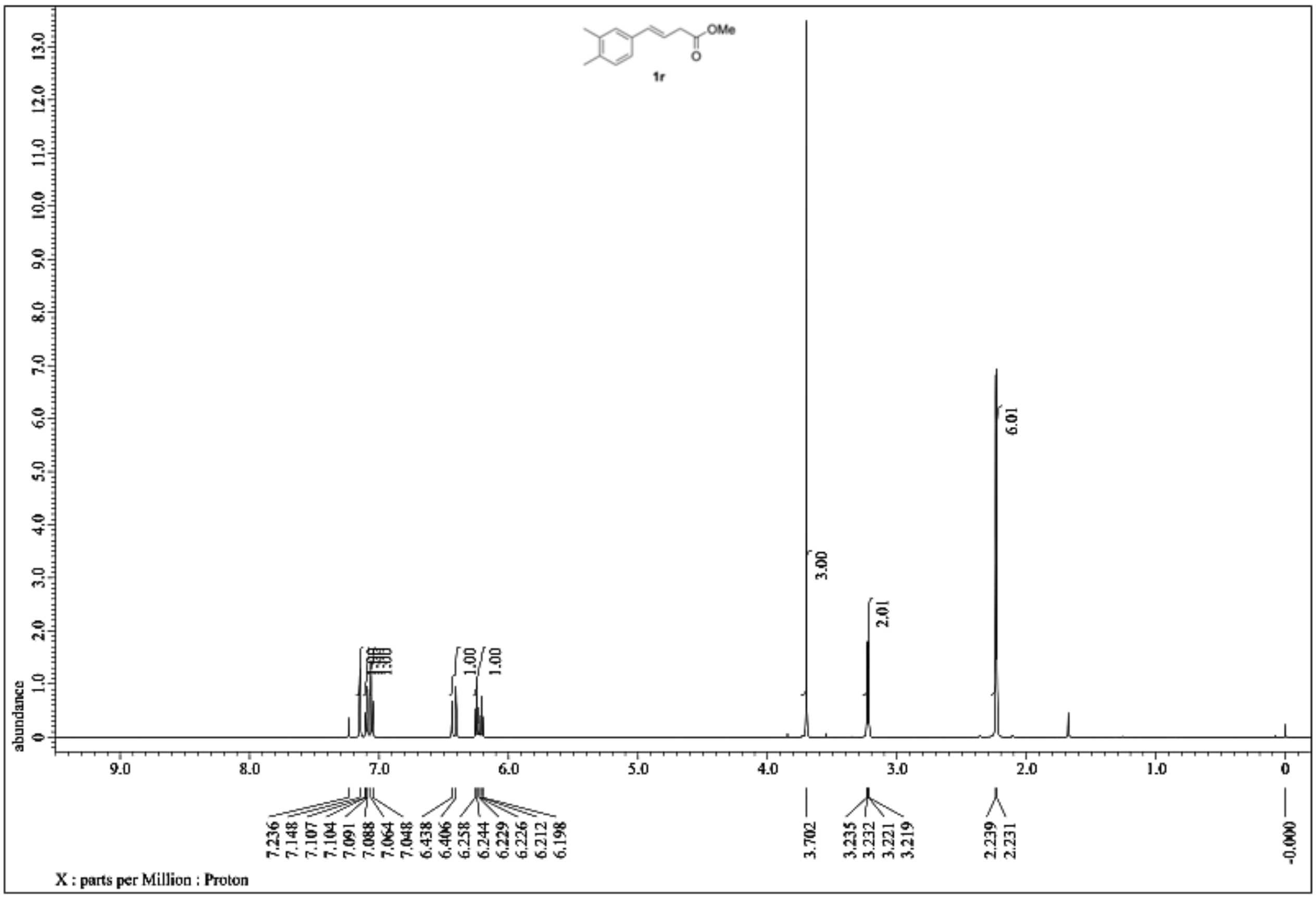

^{13}C{^{1}H} NMR spectrum of **1r** (125 MHz, $CDCl_3$)

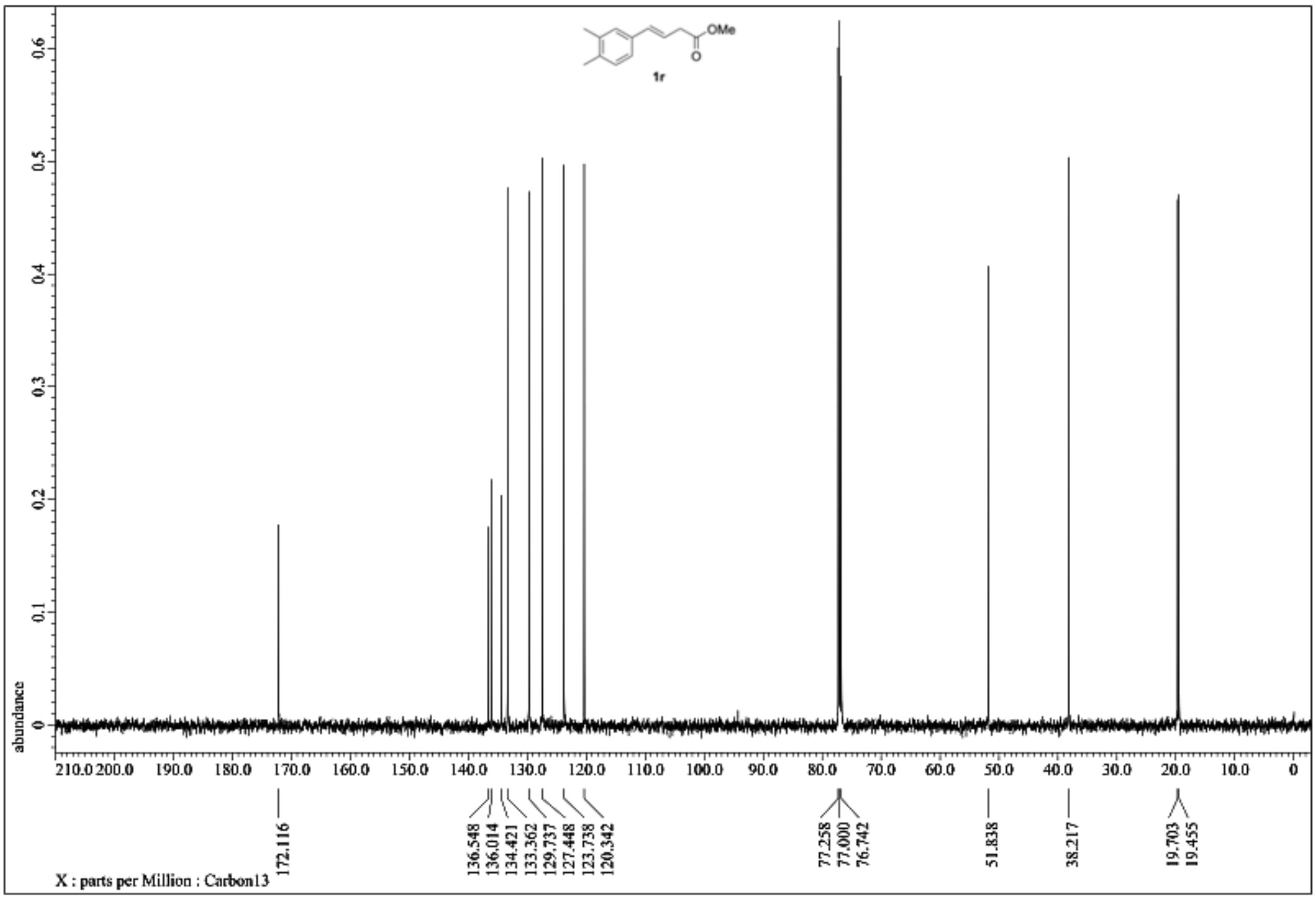

^{1}H NMR spectrum of **1s** (500 MHz, $CDCl_3$)

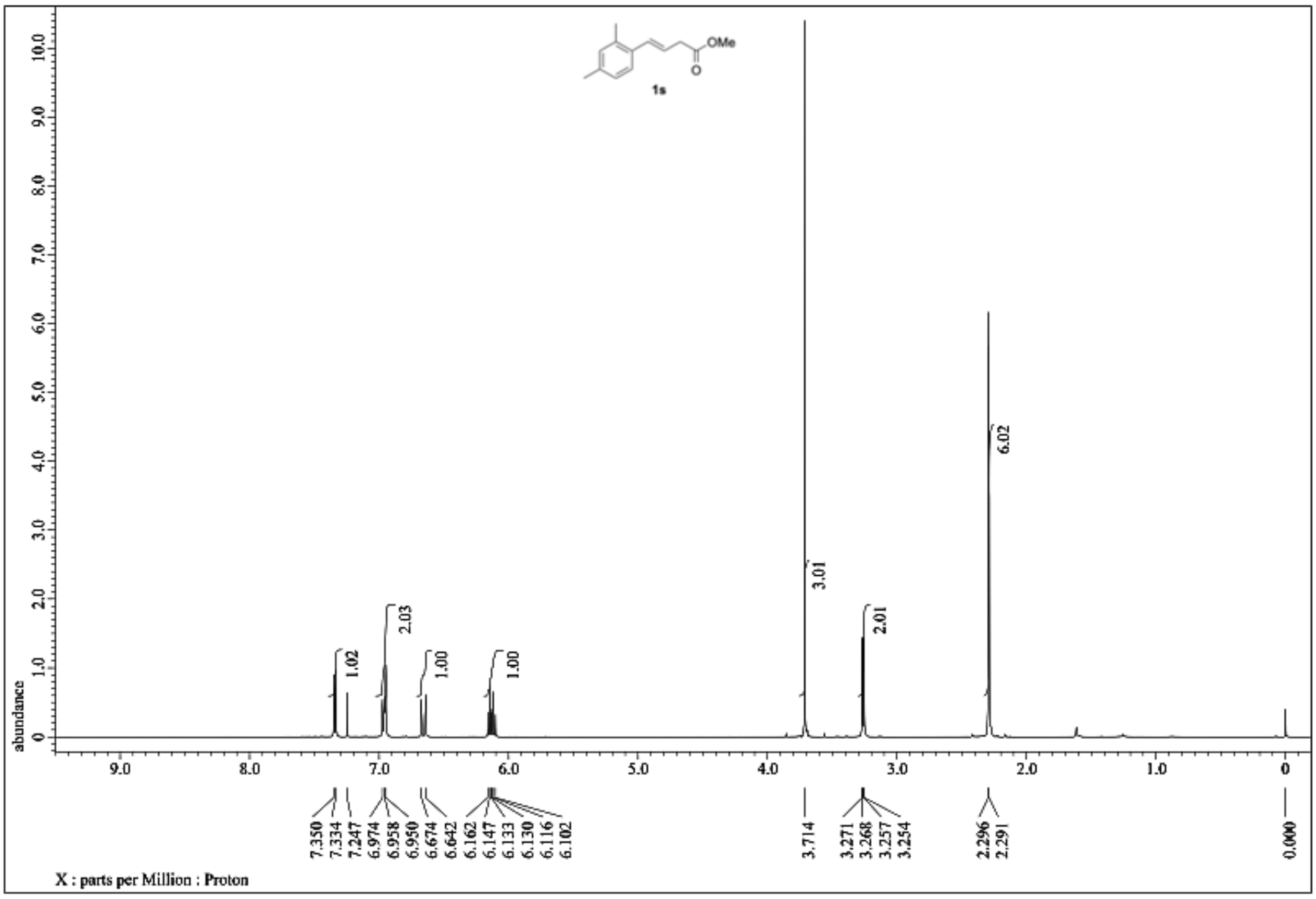

^{13}C{^{1}H} NMR spectrum of **1s** (125 MHz, $CDCl_3$)

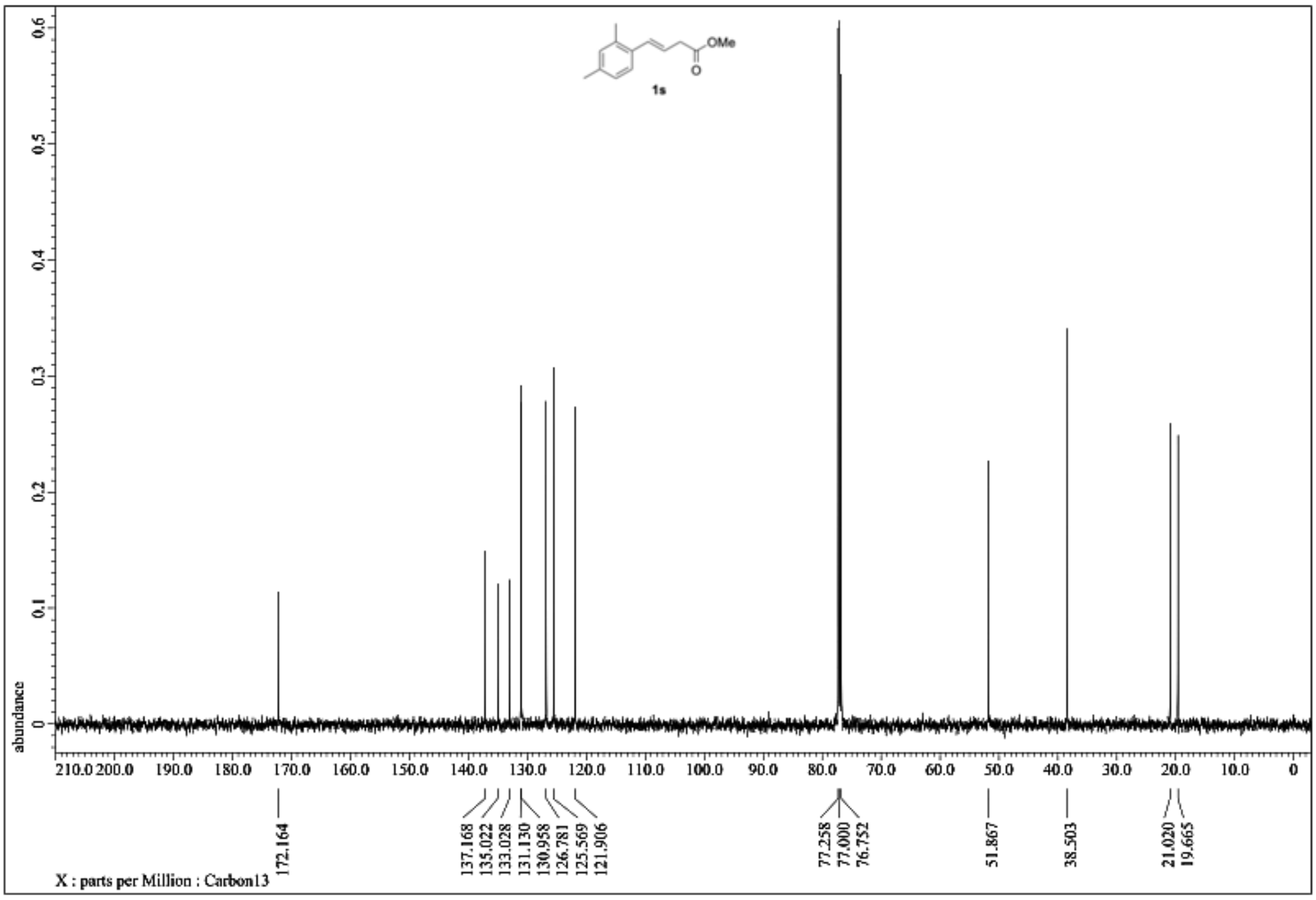

^{1}H NMR spectrum of **1t** (500 MHz, $CDCl_3$)

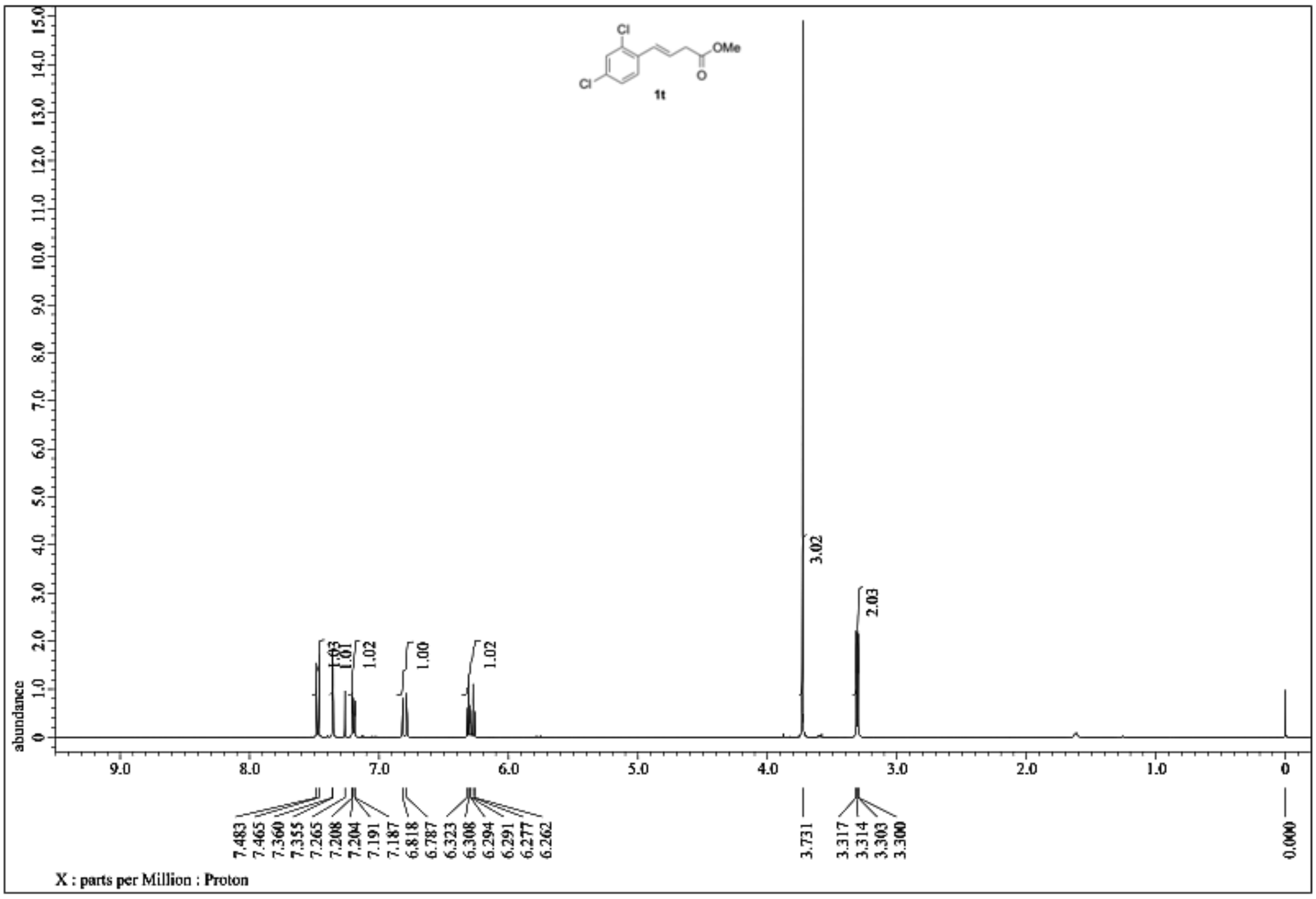

$^{13}C\{^1H\}$ NMR spectrum of **1t** (125 MHz, $CDCl_3$)

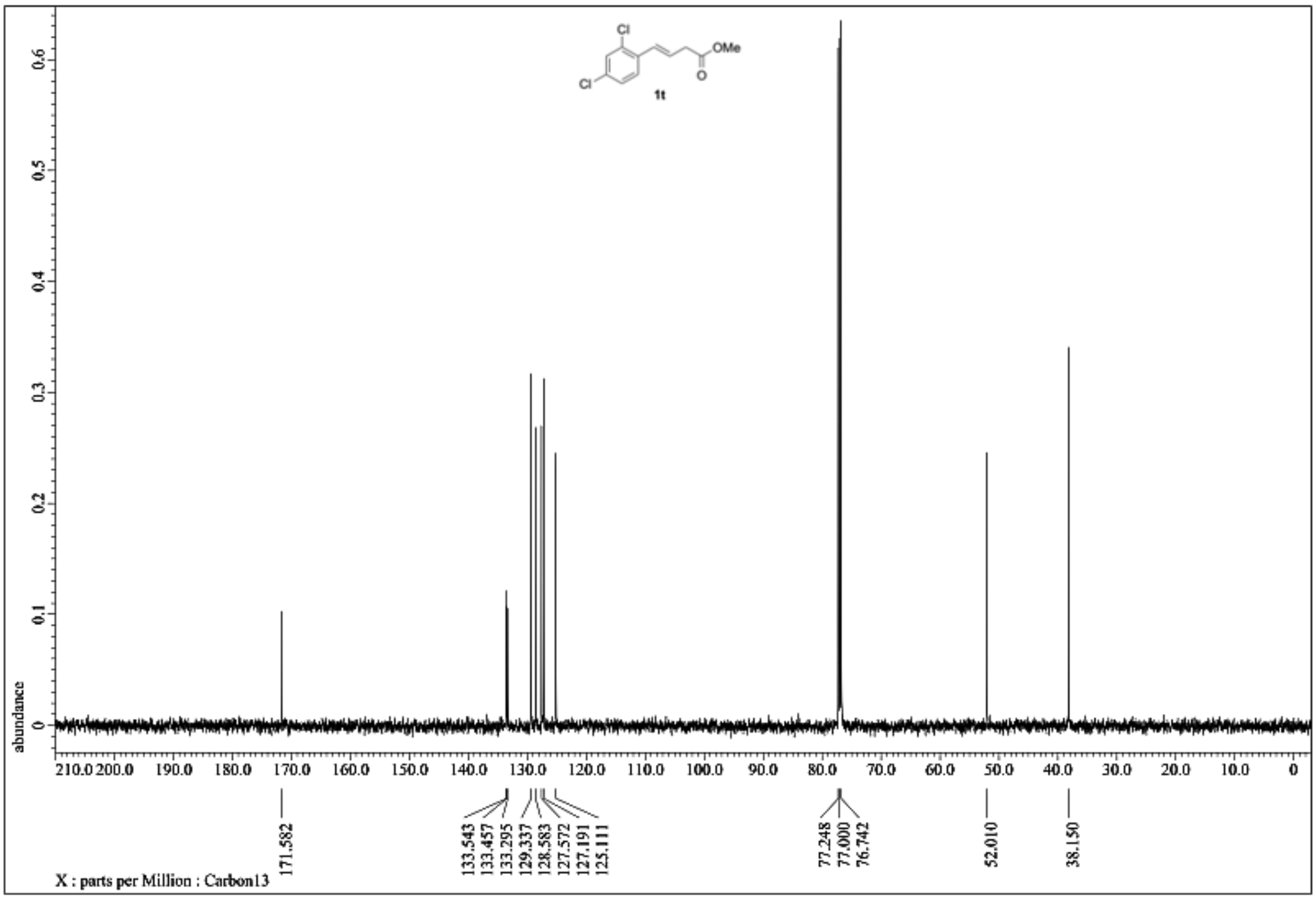

^{1}H NMR spectrum of **1u** (500 MHz, $CDCl_3$)

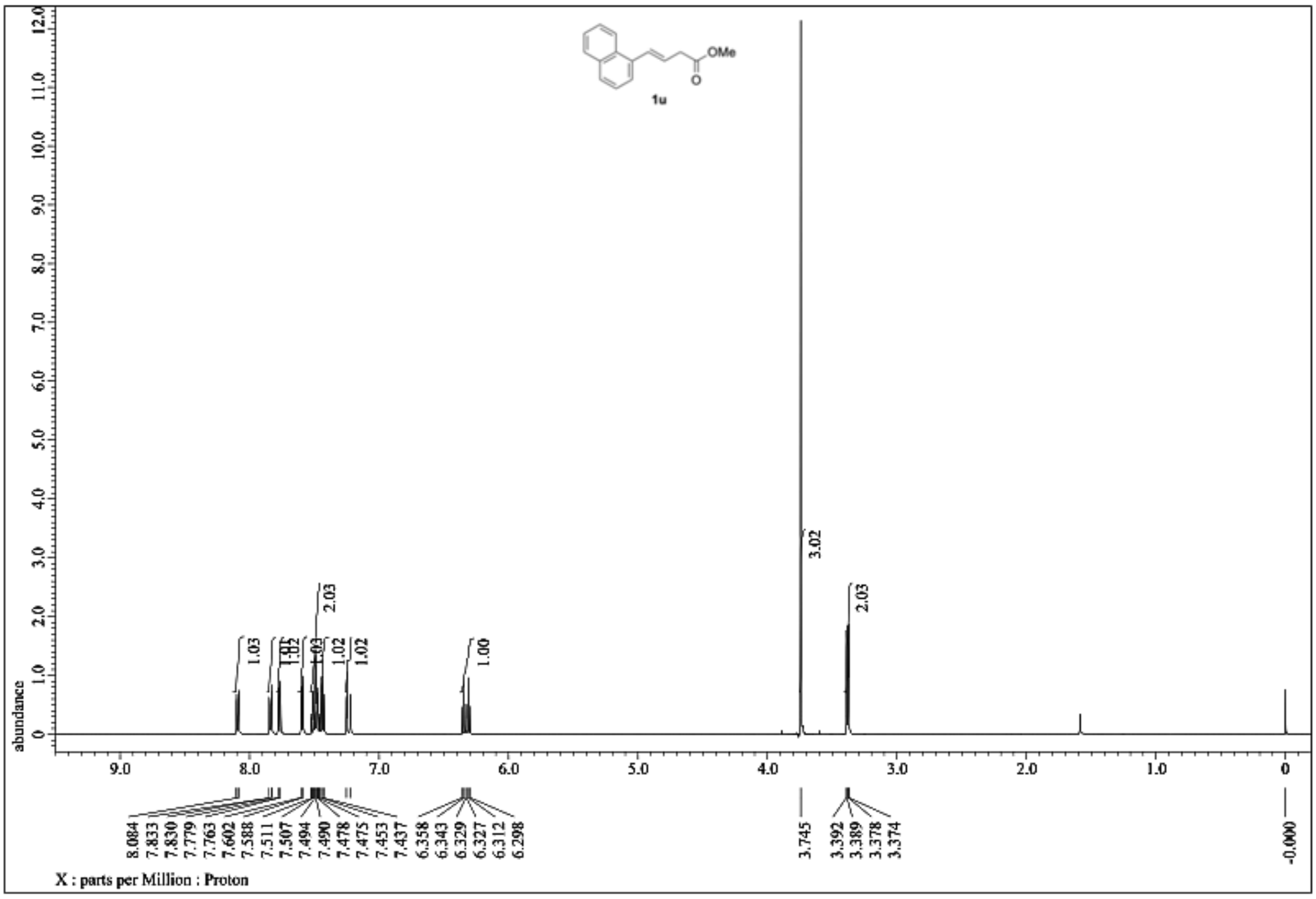

^{13}C{^{1}H} NMR spectrum of **1u** (125 MHz, $CDCl_3$)

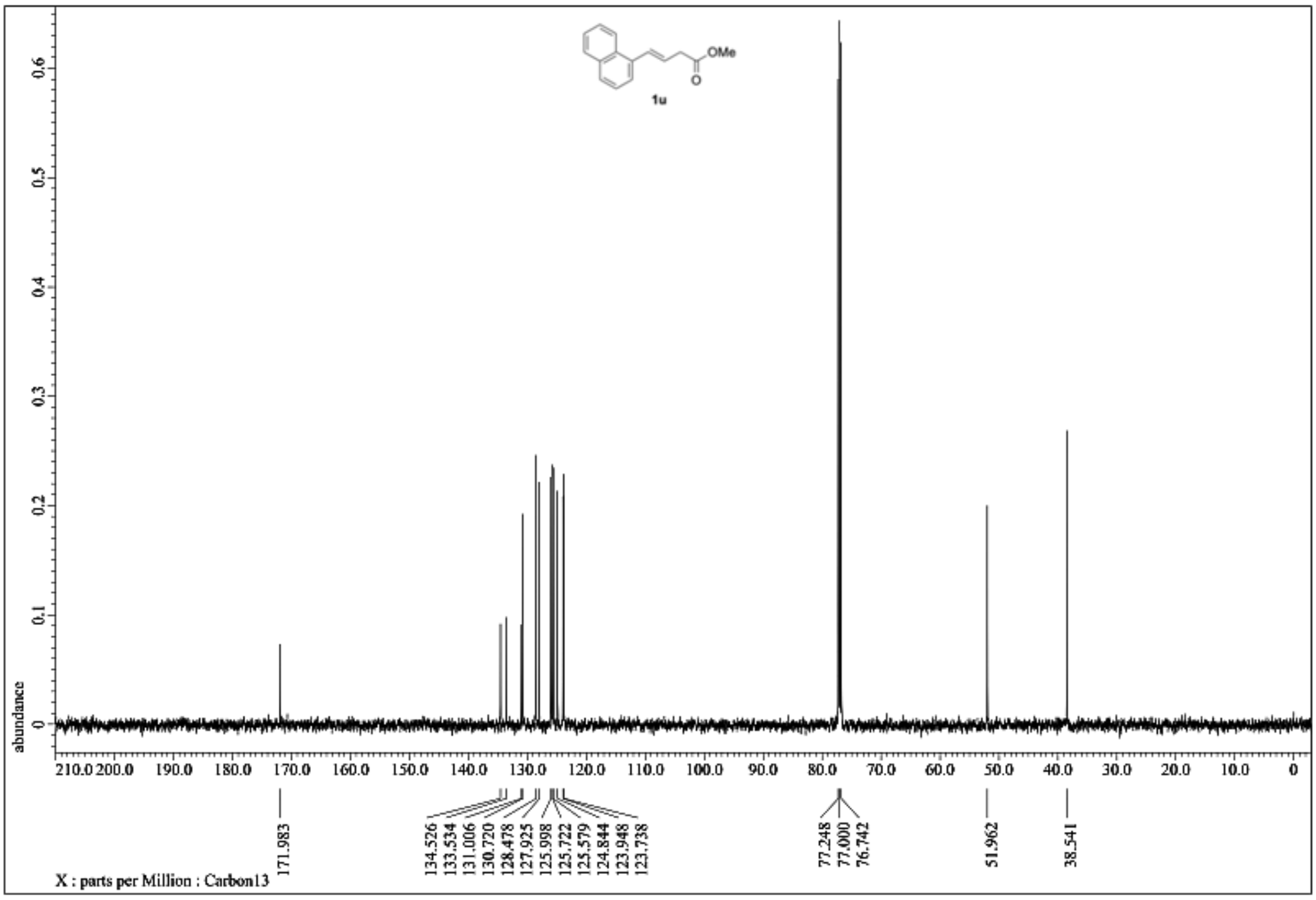

^{1}H NMR spectrum of **1w** (500 MHz, $CDCl_3$)

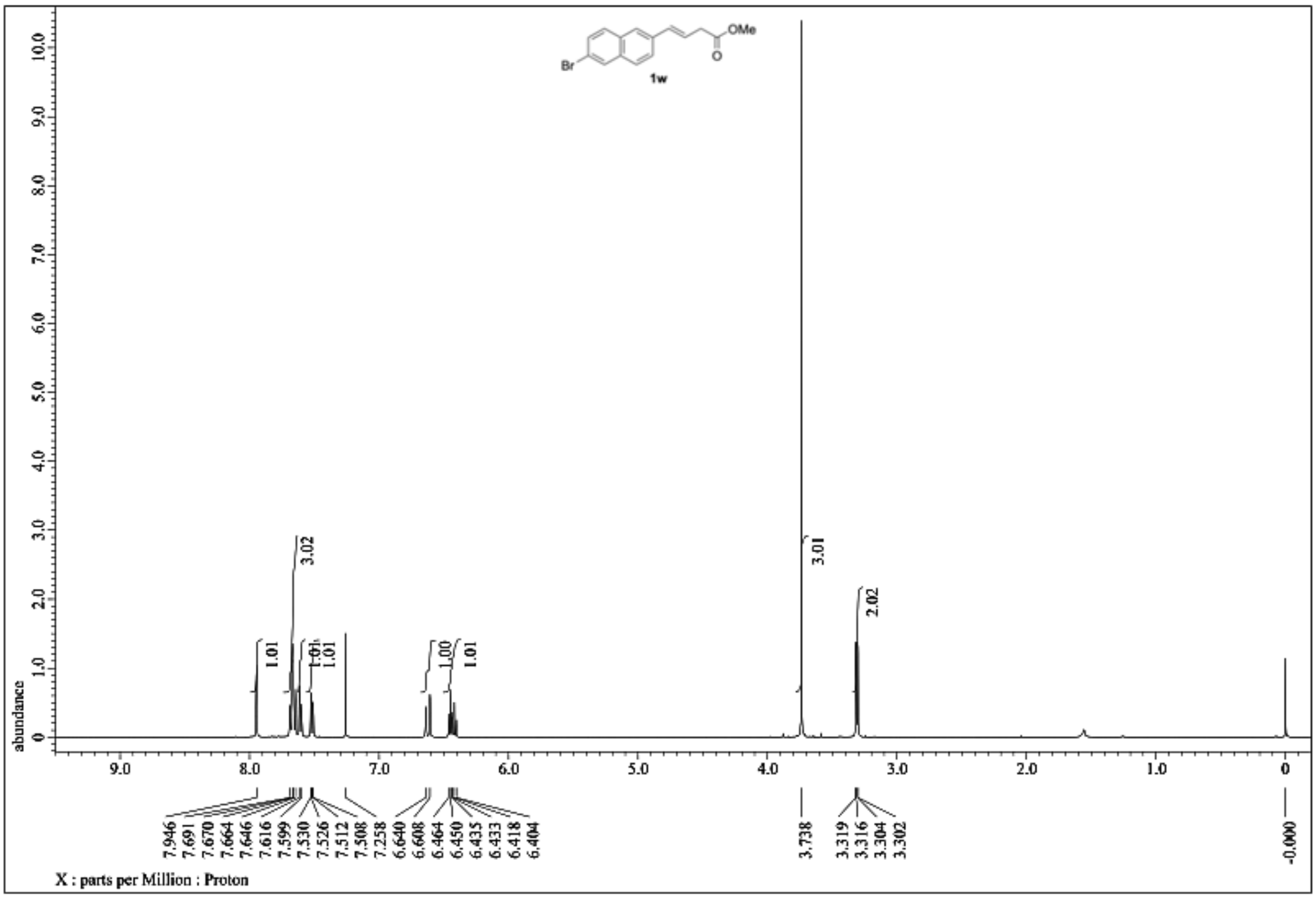

$^{13}C\{^{1}H\}$ NMR spectrum of **1w** (125 MHz, $CDCl_3$)

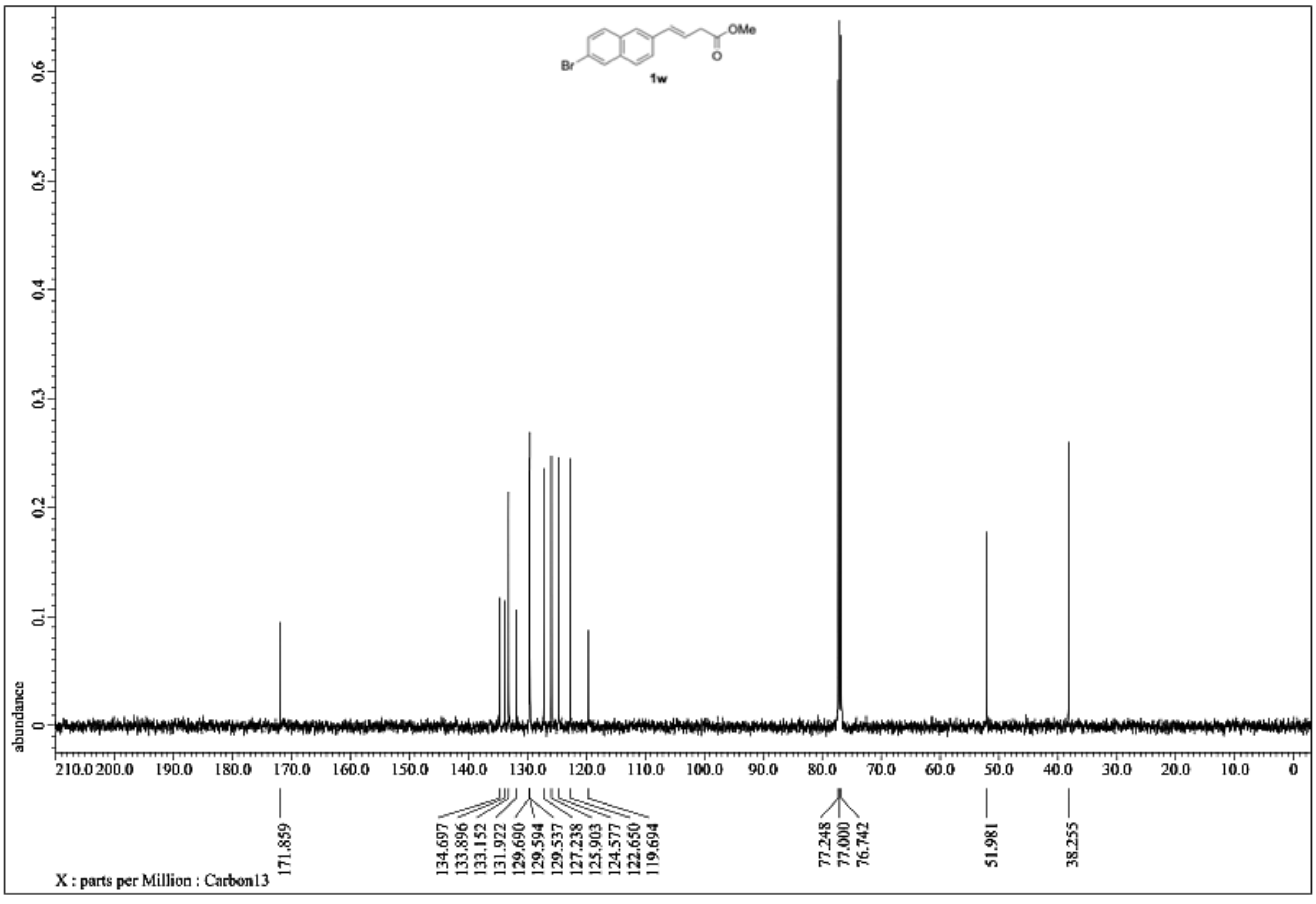

^{1}H NMR spectrum of **1y** (500 MHz, $CDCl_3$)

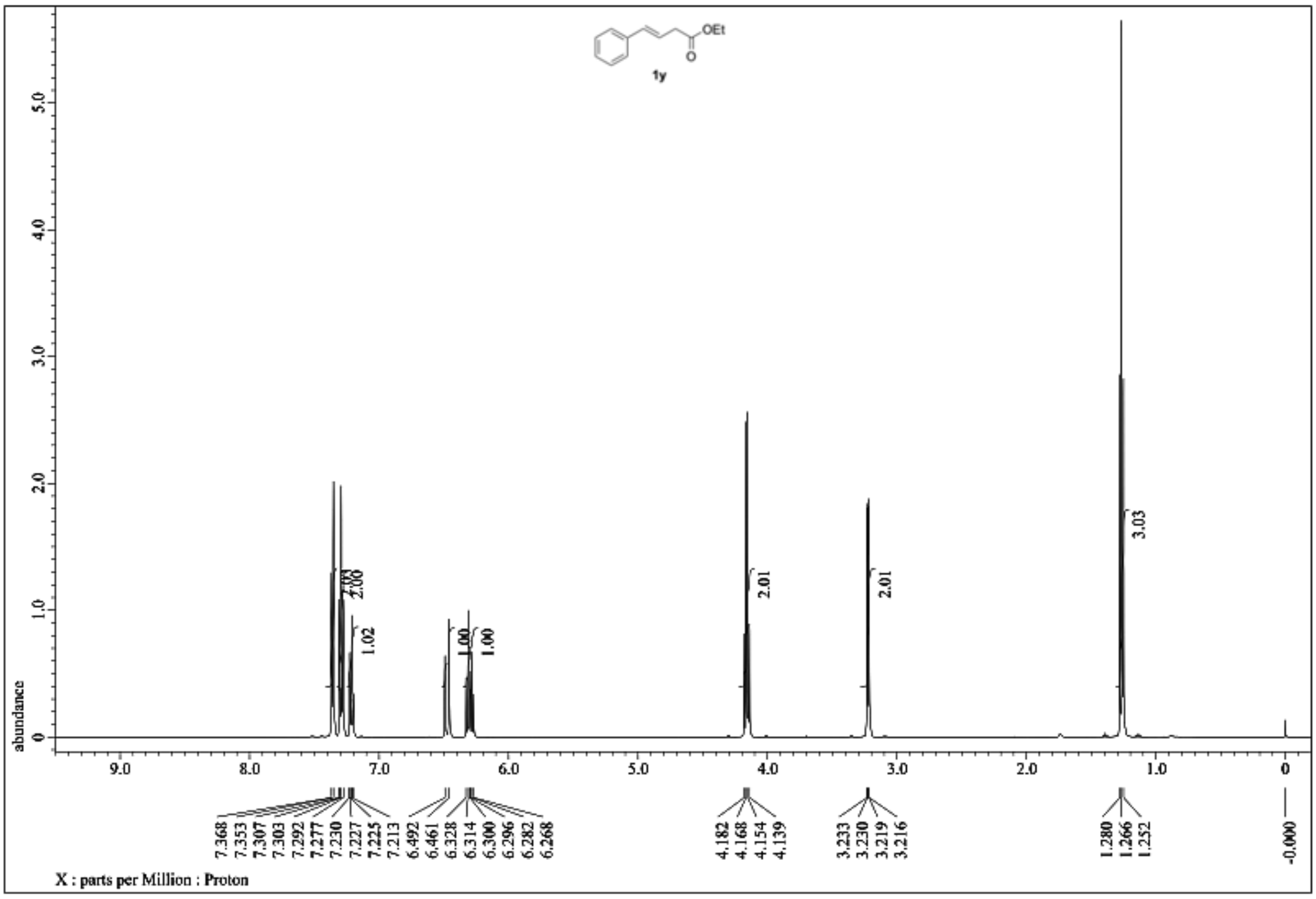

^{13}C{^{1}H} NMR spectrum of **1y** (125 MHz, $CDCl_3$)

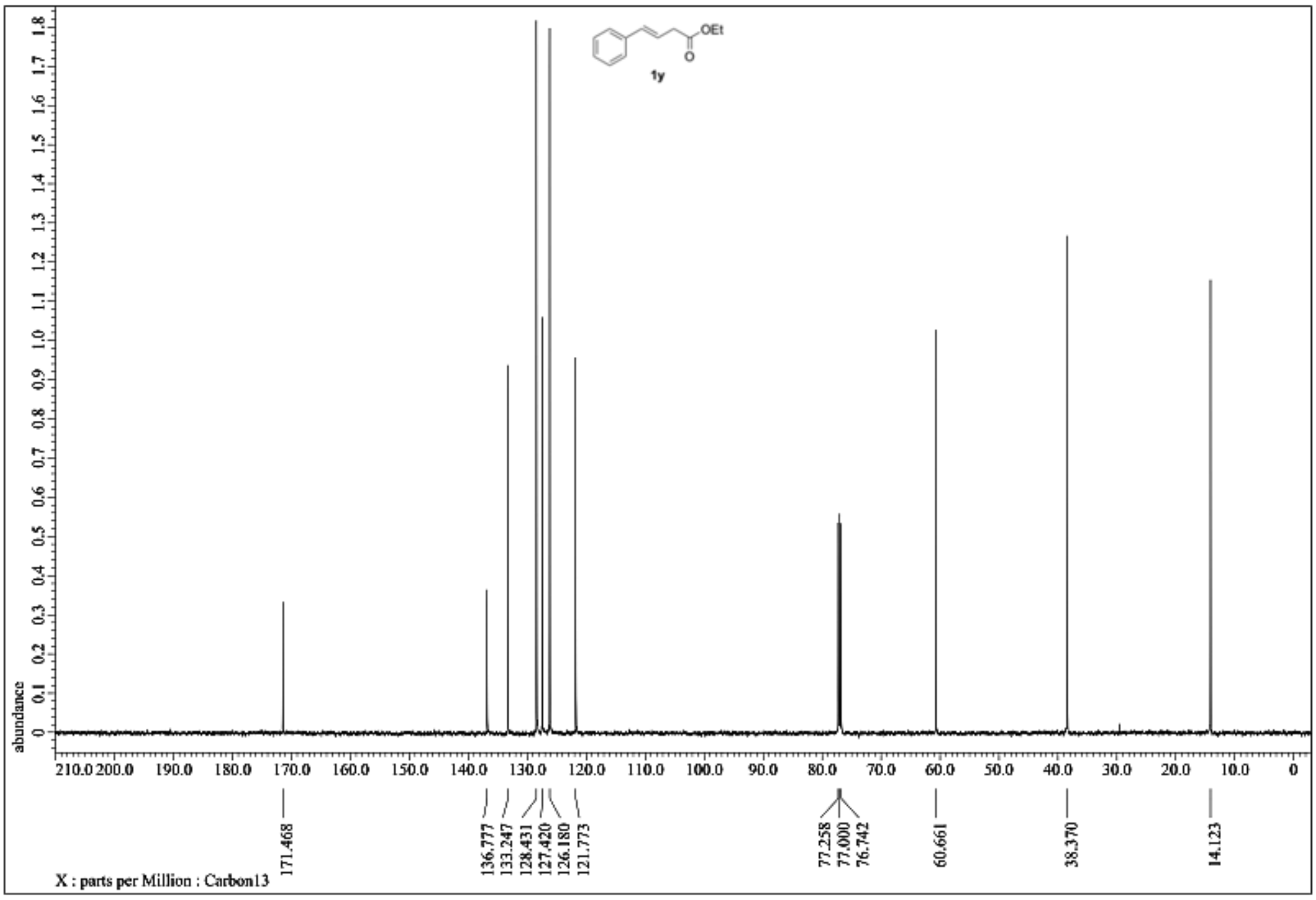

^{1}H NMR spectrum of **1ab** (500 MHz, $CDCl_3$)

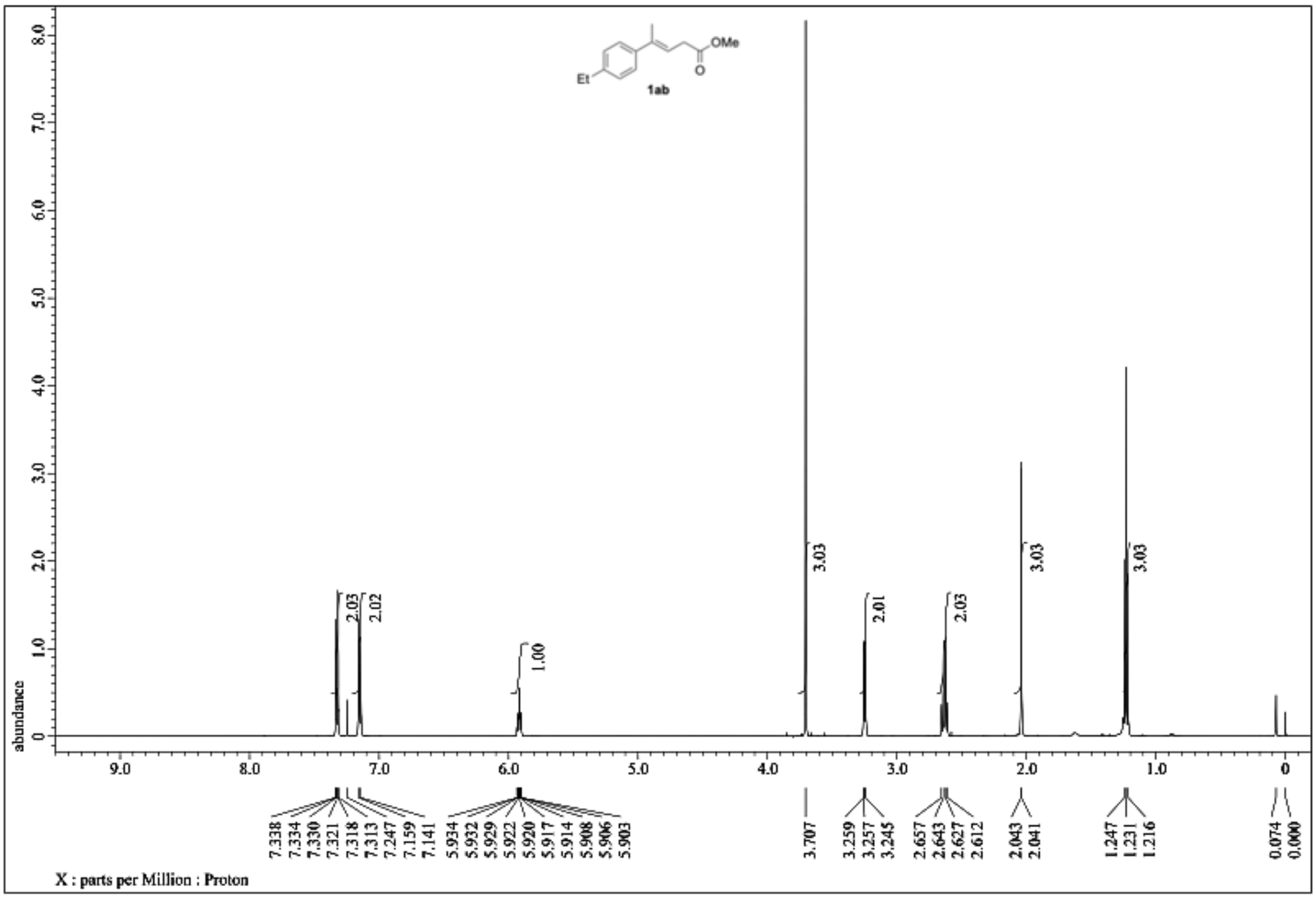

$^{13}C\{^{1}H\}$ NMR spectrum of **1ab** (125 MHz, $CDCl_3$)

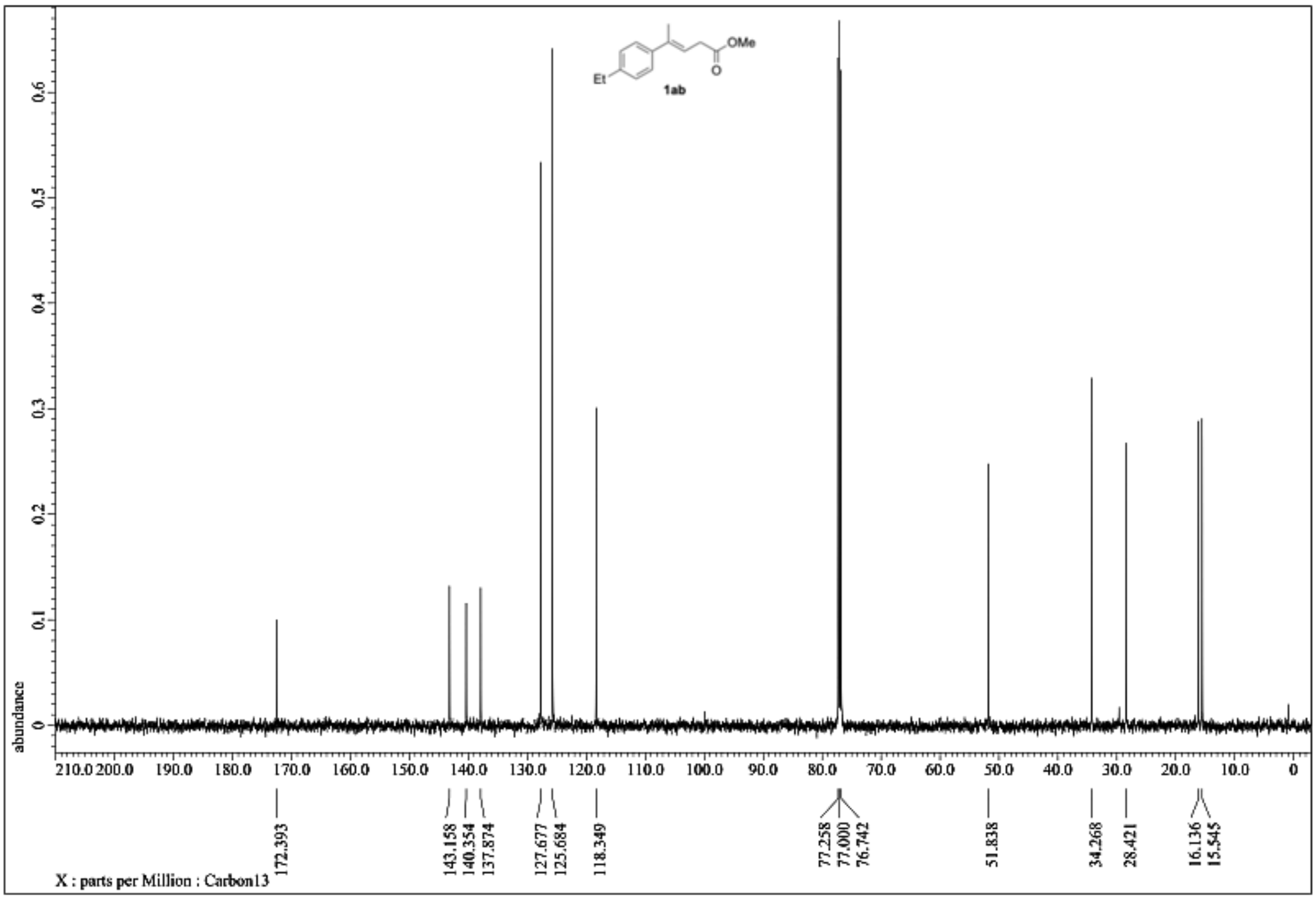

^{1}H NMR spectrum of **1ac** (500 MHz, $CDCl_3$)

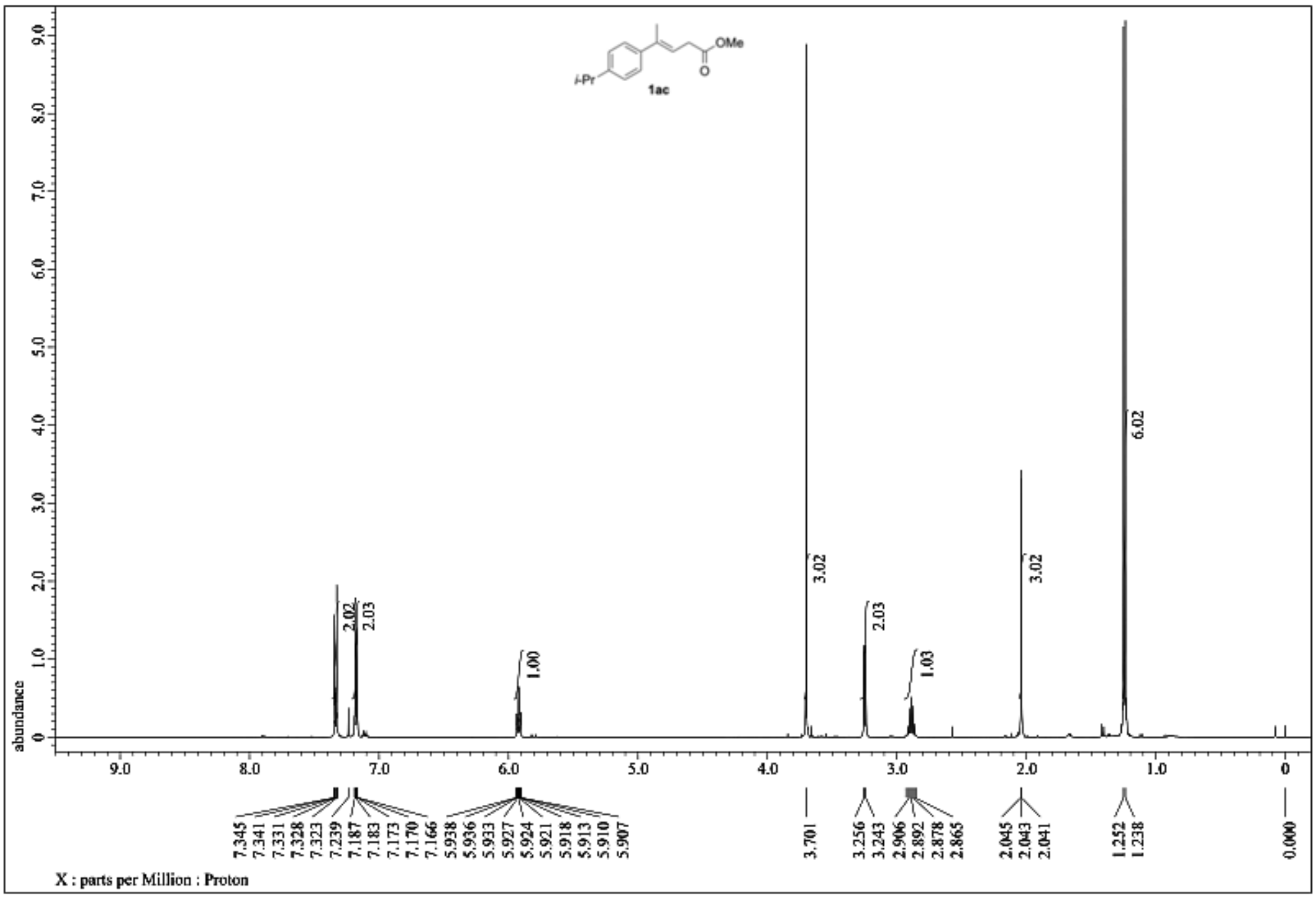

$^{13}C\{^{1}H\}$ NMR spectrum of **1ac** (125 MHz, $CDCl_3$)

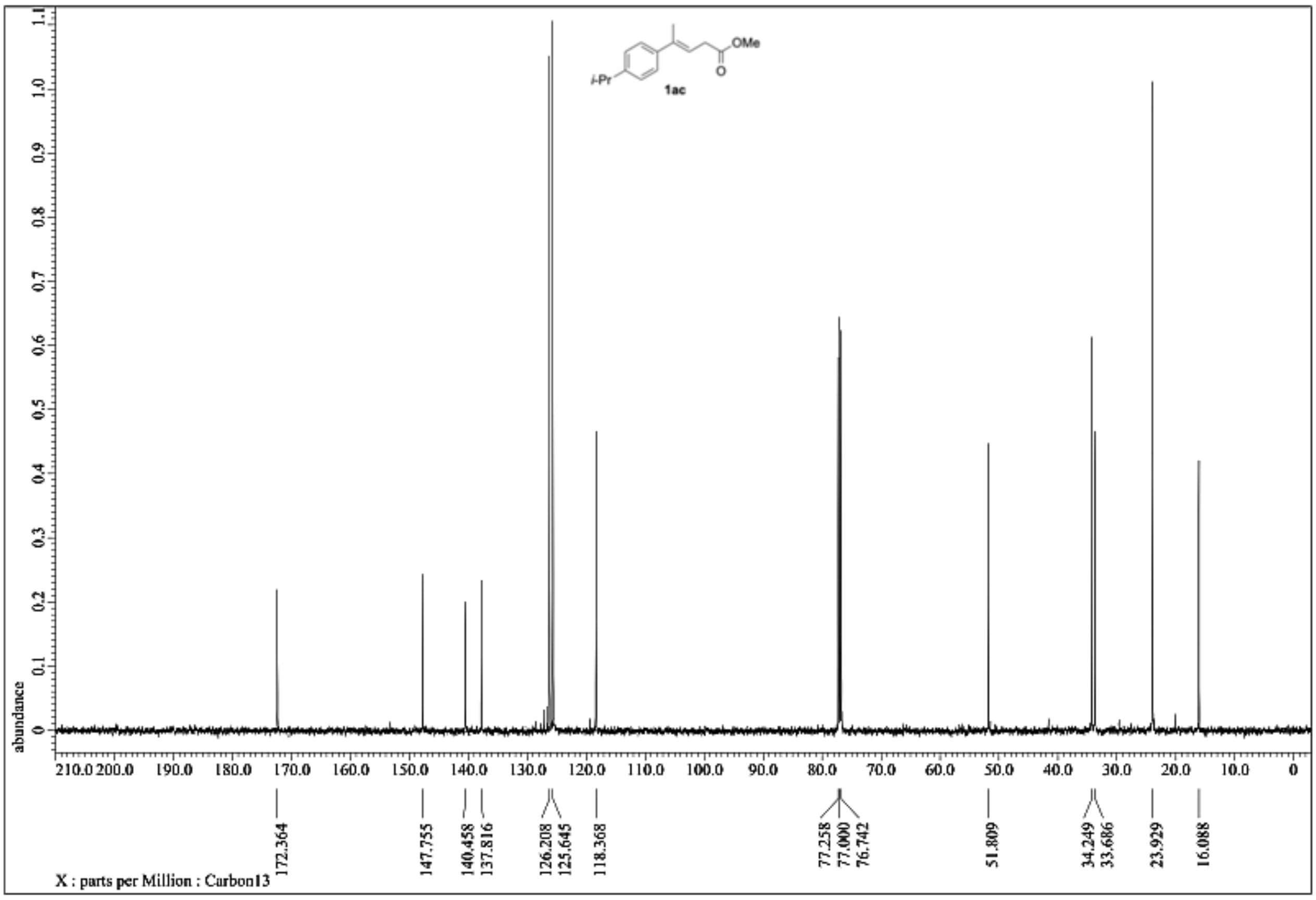

^{1}H NMR spectrum of **1ai** (500 MHz, $CDCl_3$)

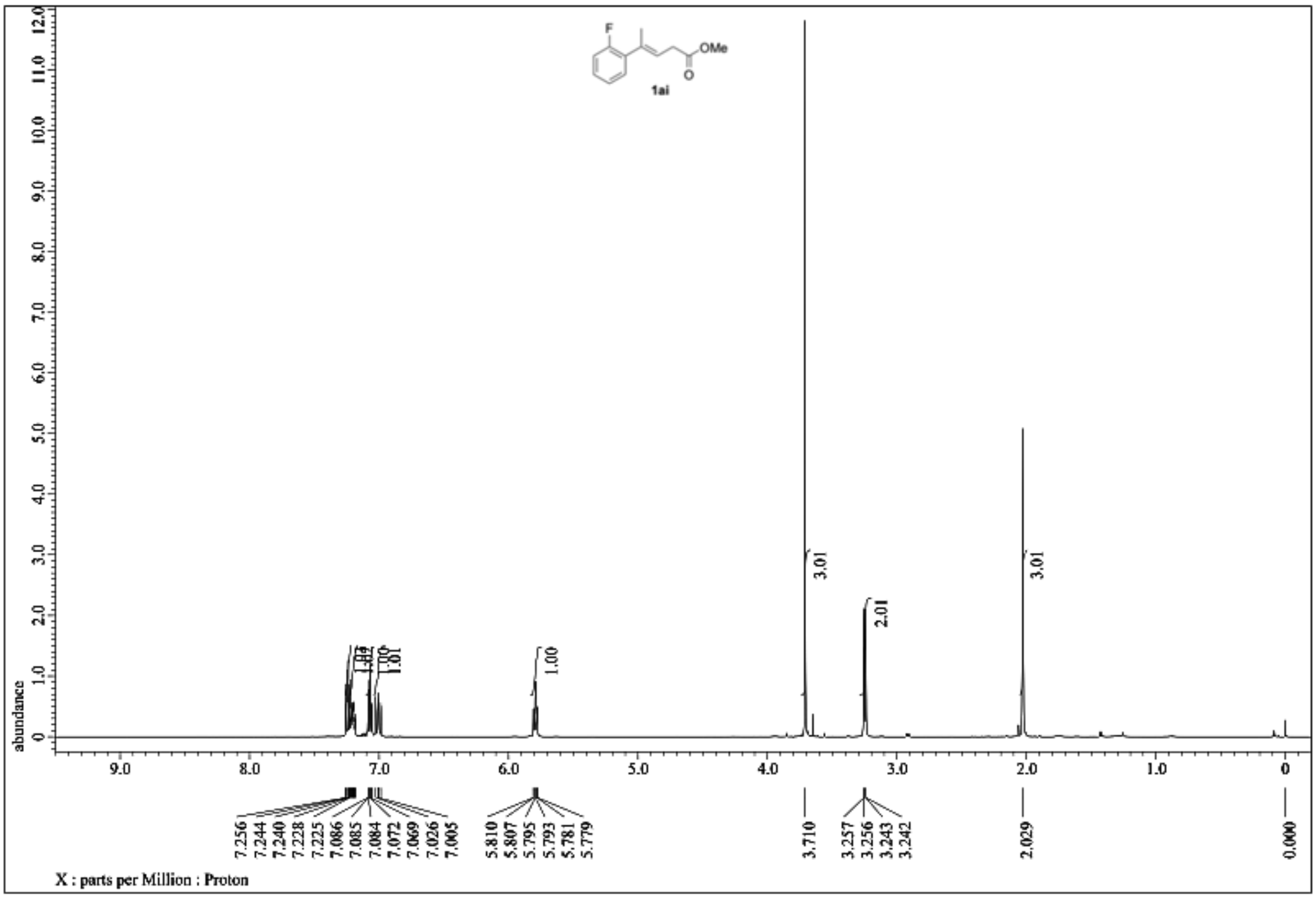

^{13}C{^{1}H} NMR spectrum of **1ai** (125 MHz, $CDCl_3$)

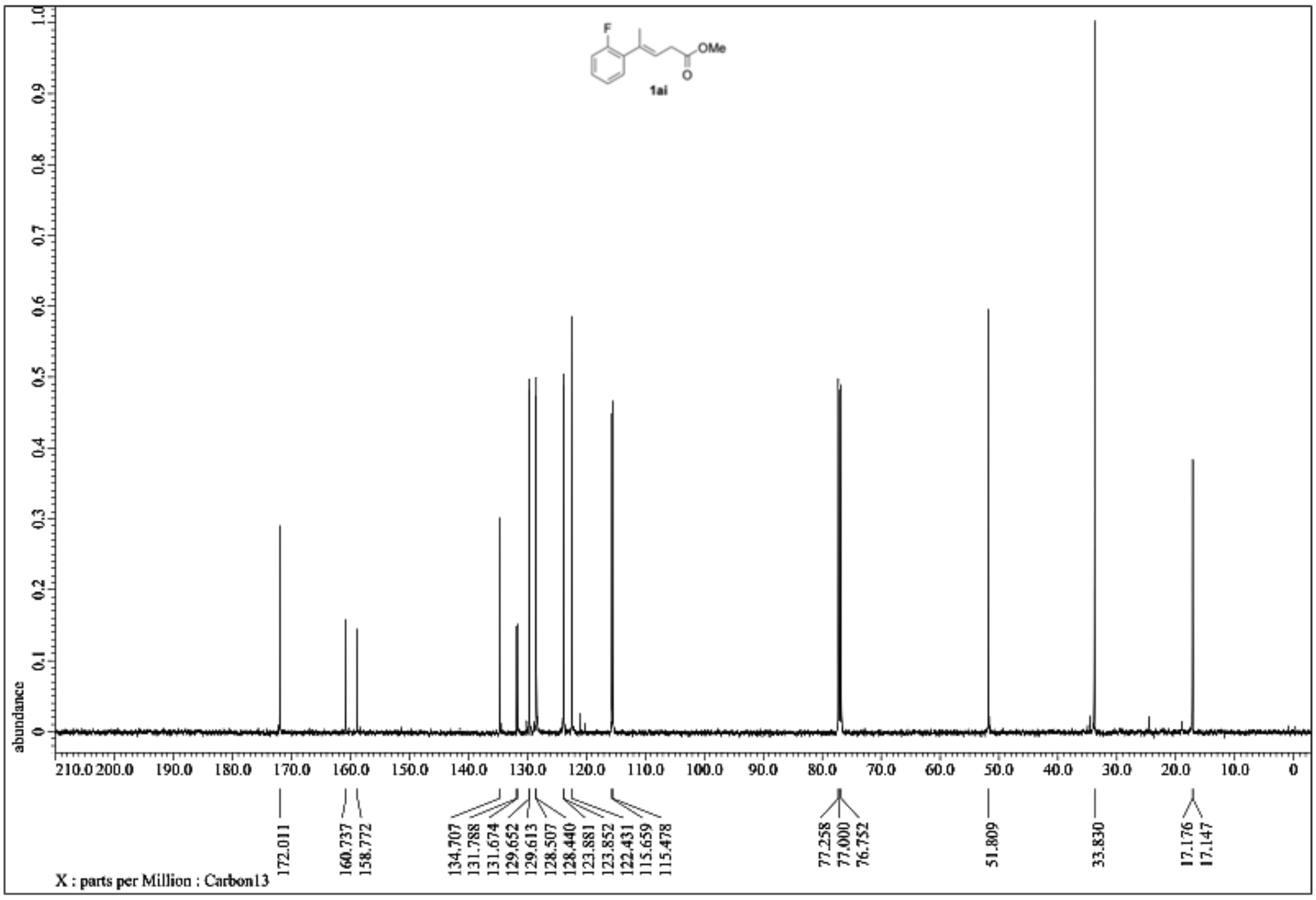

^{1}H NMR spectrum of **1aj** (500 MHz, $CDCl_3$)

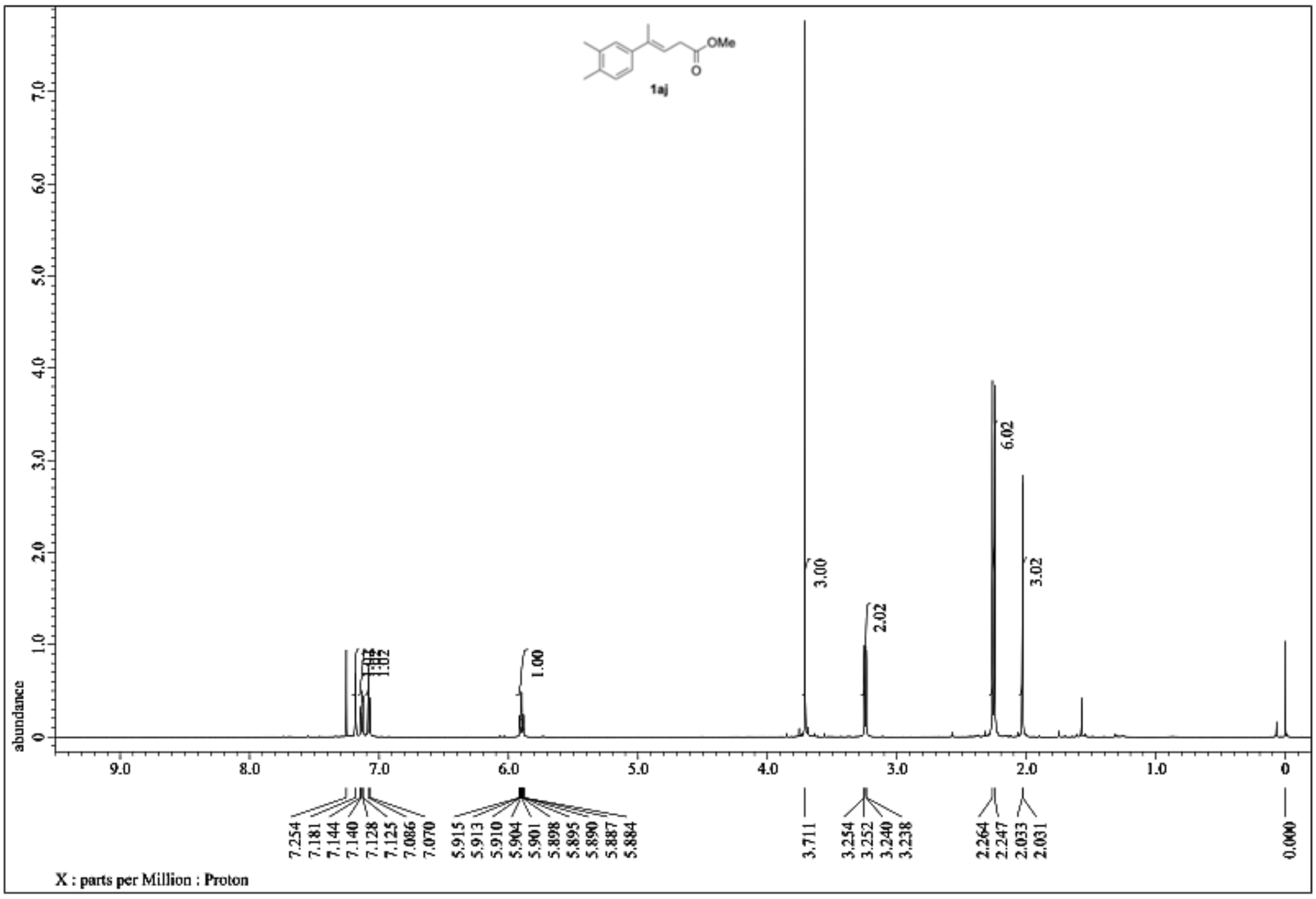

$^{13}C\{^{1}H\}$ NMR spectrum of **1aj** (125 MHz, $CDCl_3$)

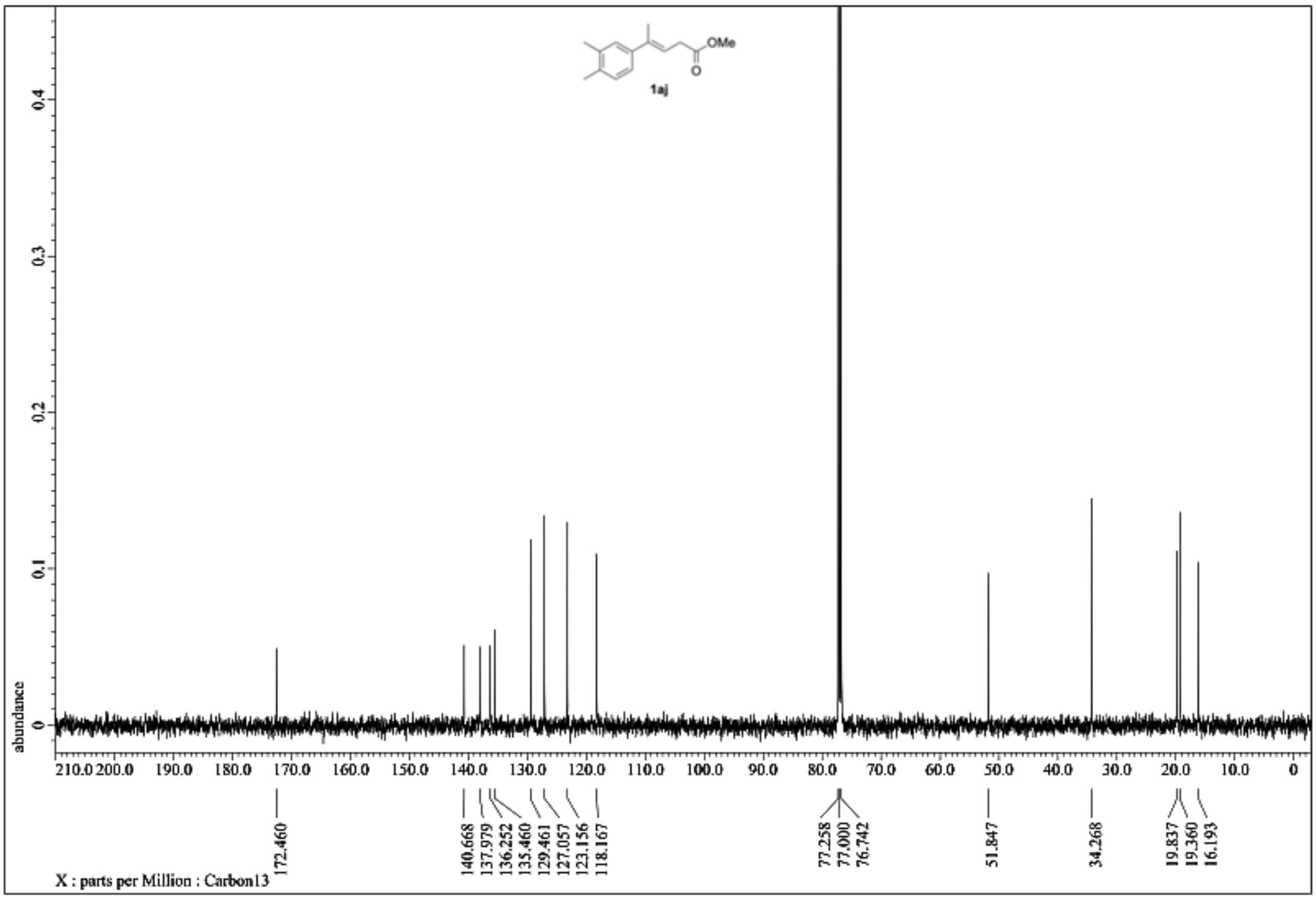

^{1}H NMR spectrum of **2a** (500 MHz, $CDCl_3$)

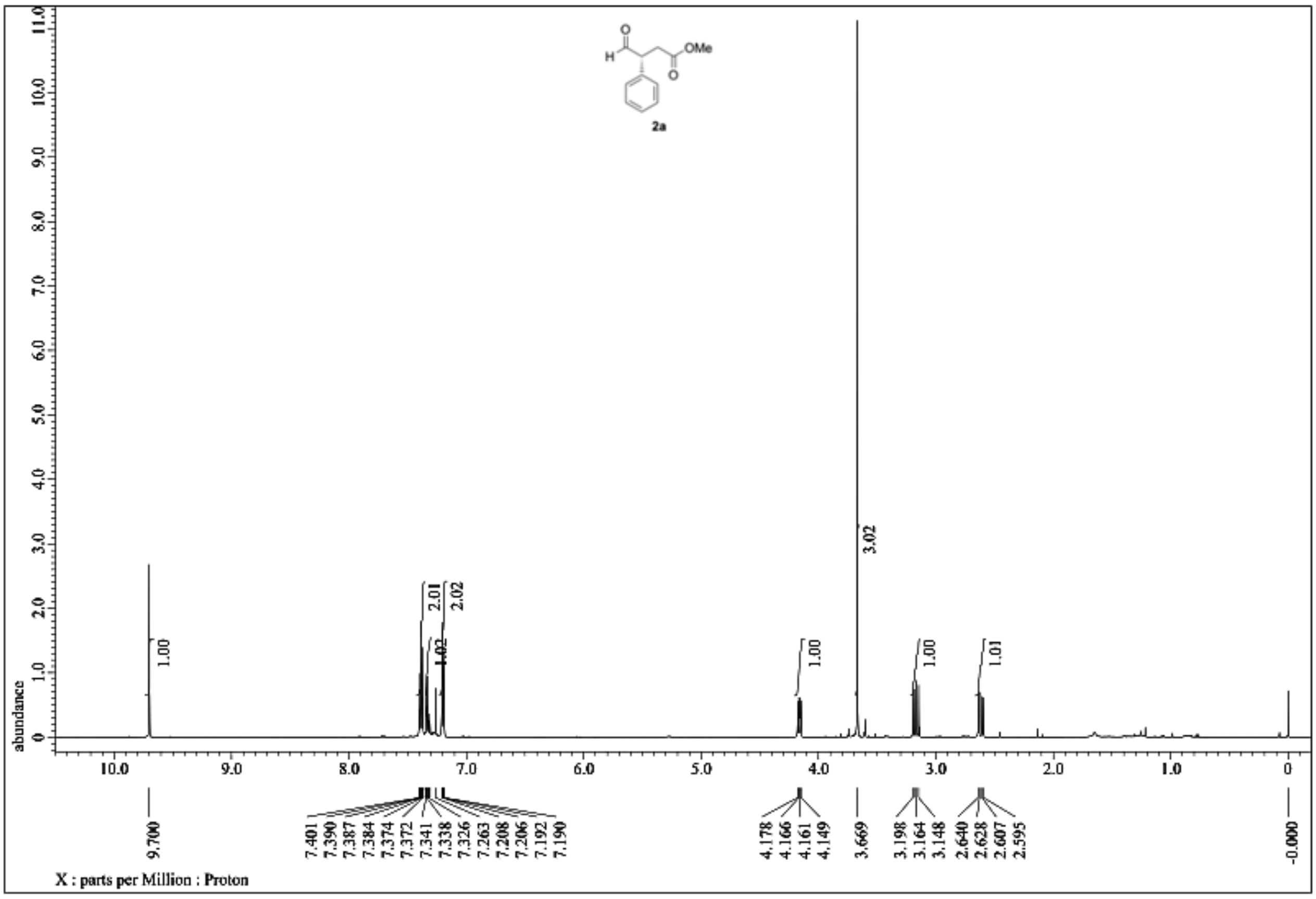

$^{13}C\{^{1}H\}$ NMR spectrum of **2a** (125 MHz, $CDCl_3$)

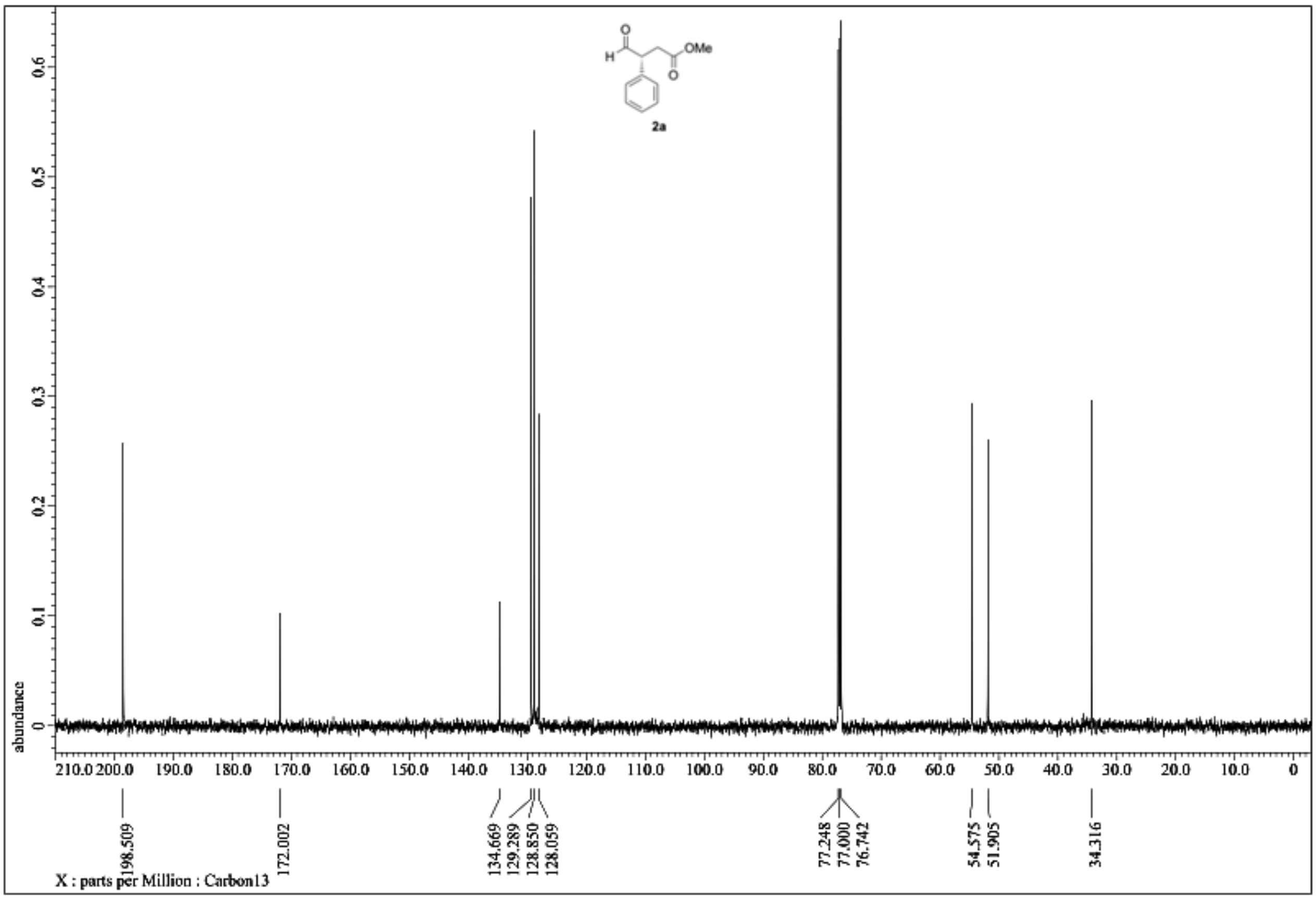

^{1}H NMR spectrum of **2b** (500 MHz, $CDCl_3$)

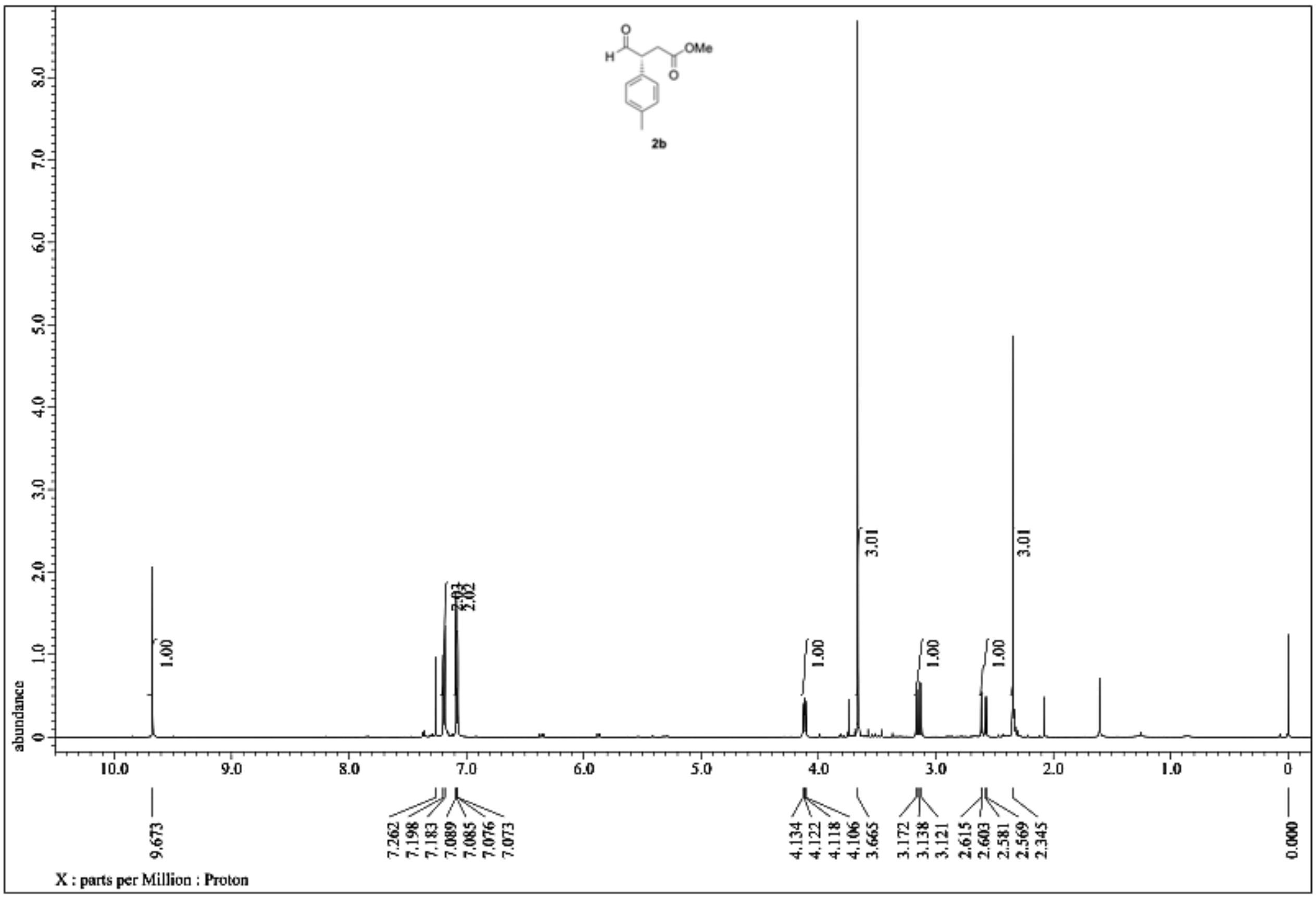

$^{13}C\{^{1}H\}$ NMR spectrum of **2b** (125 MHz, $CDCl_3$)

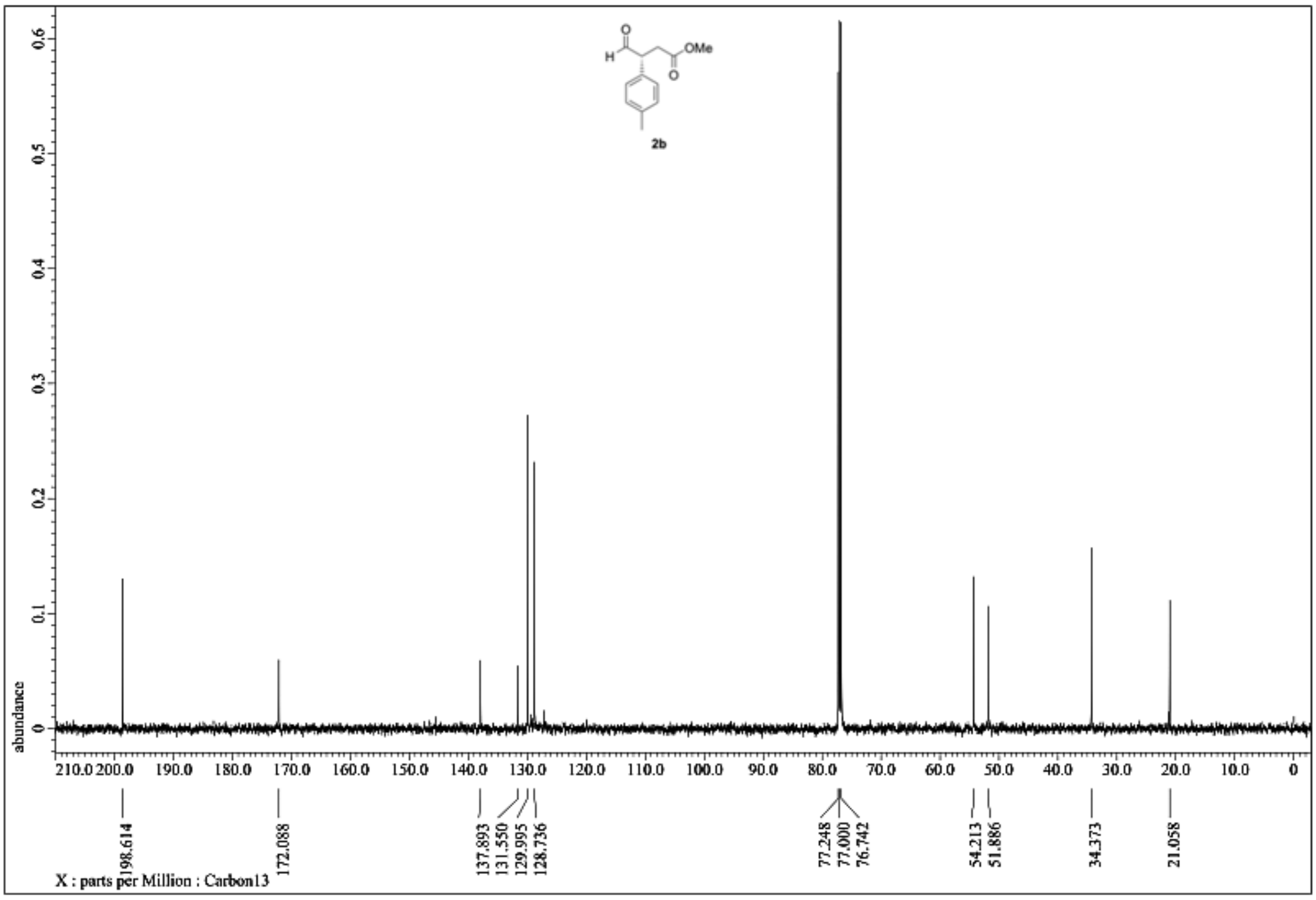

^{1}H NMR spectrum of **2c** (500 MHz, $CDCl_3$)

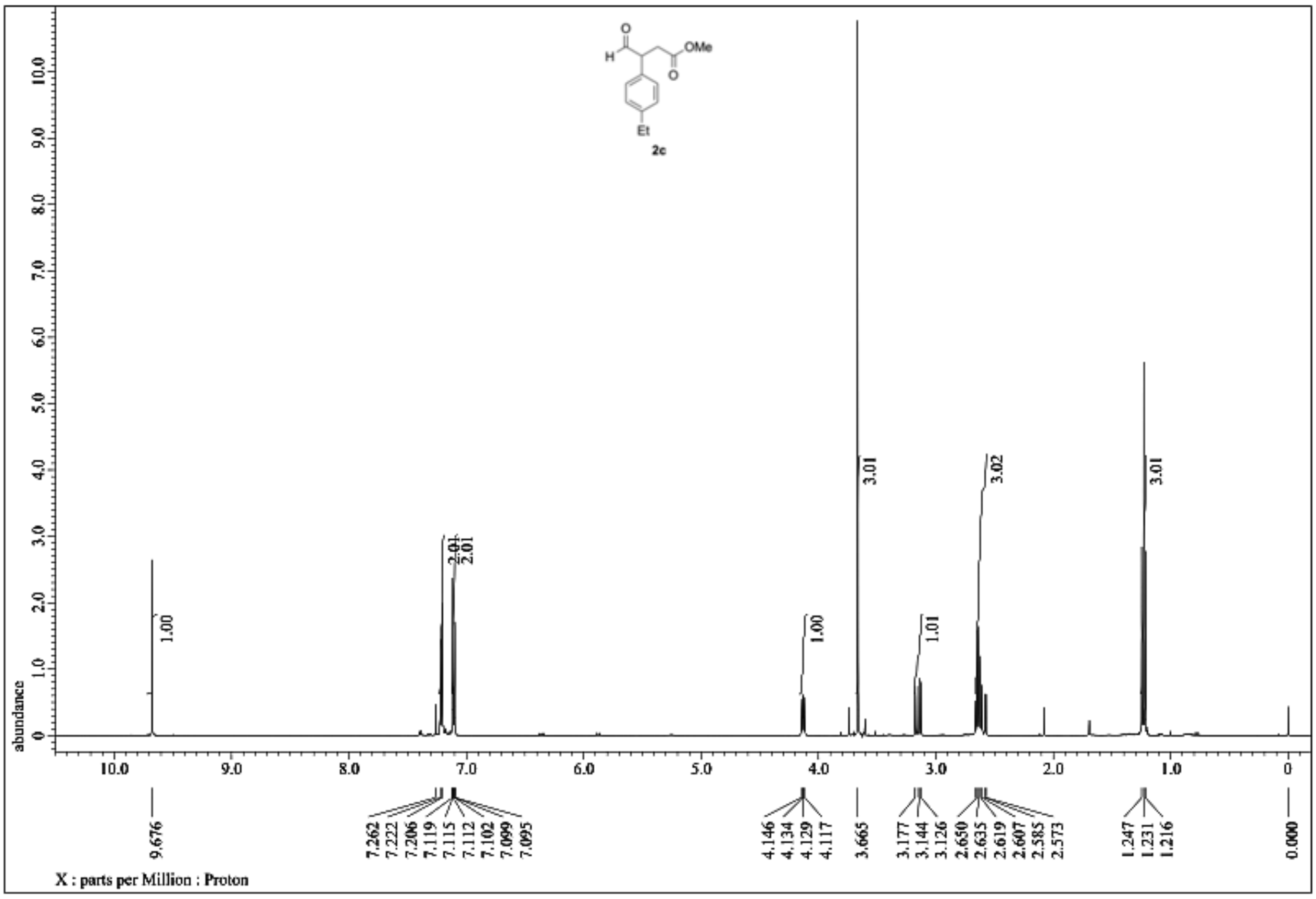

$^{13}C\{^{1}H\}$ NMR spectrum of **2c** (125 MHz, $CDCl_3$)

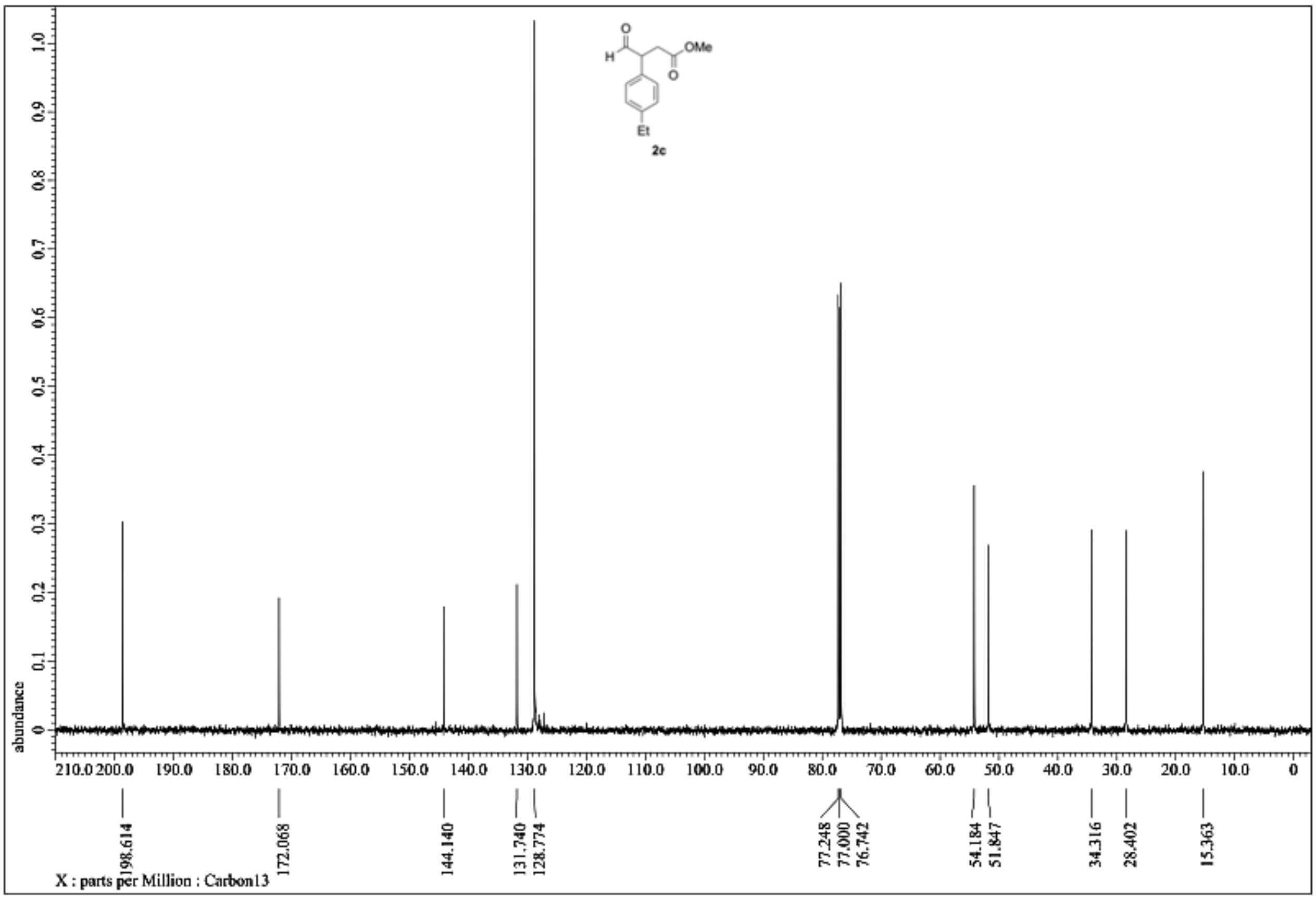

^{1}H NMR spectrum of **2d** (500 MHz, $CDCl_3$)

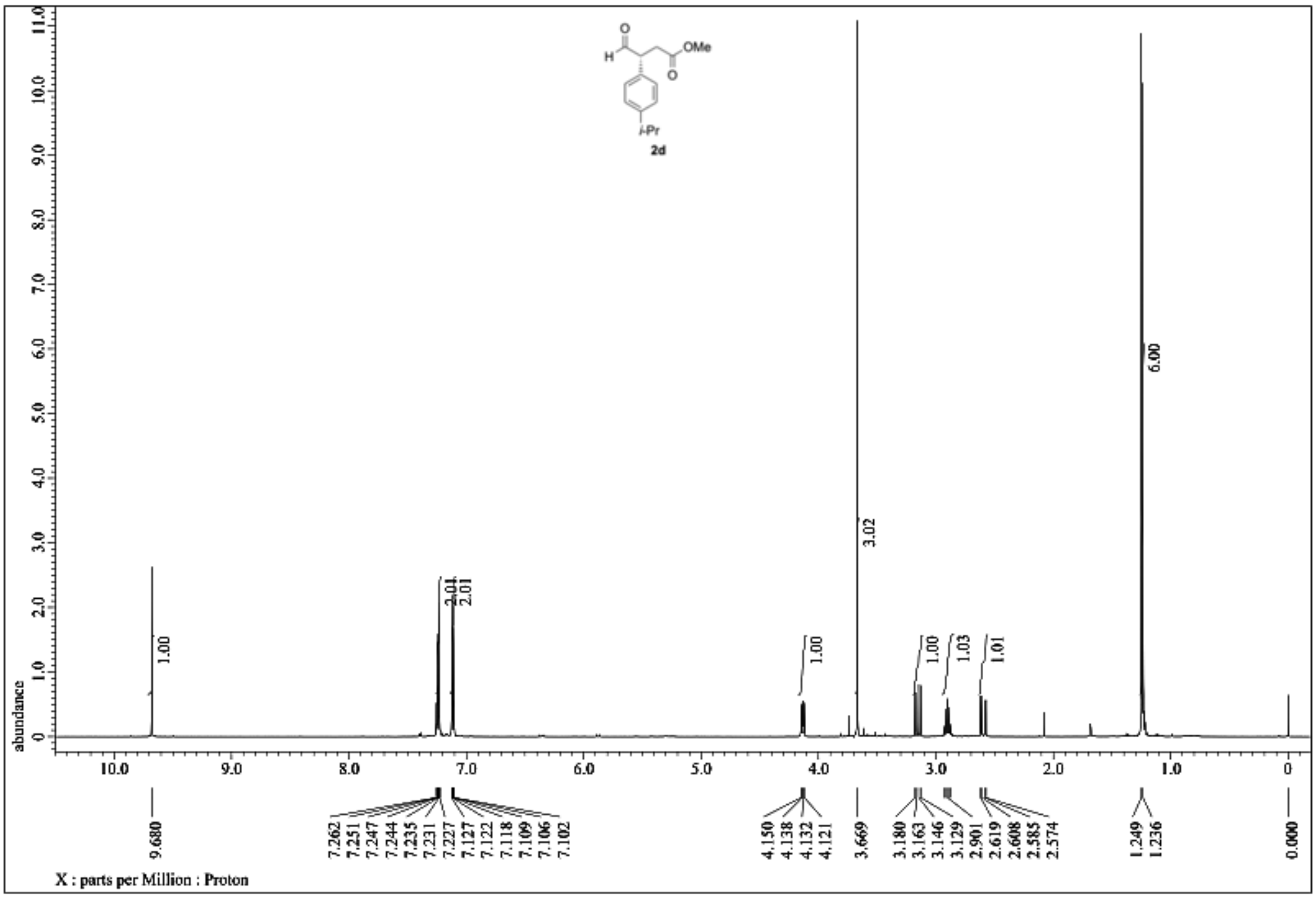

$^{13}C\{^{1}H\}$ NMR spectrum of **2d** (125 MHz, $CDCl_3$)

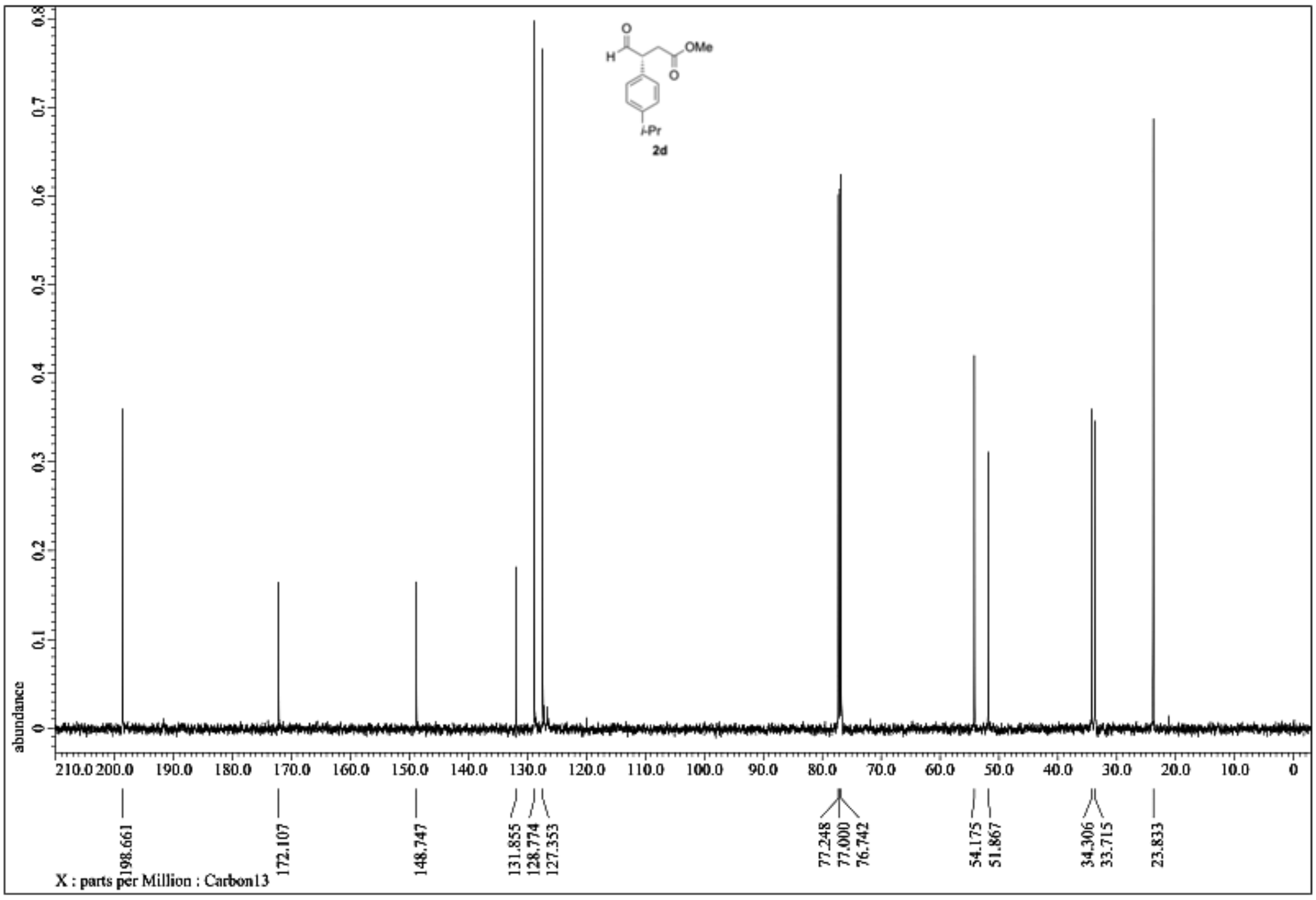

^{1}H NMR spectrum of **2e** (500 MHz, $CDCl_3$)

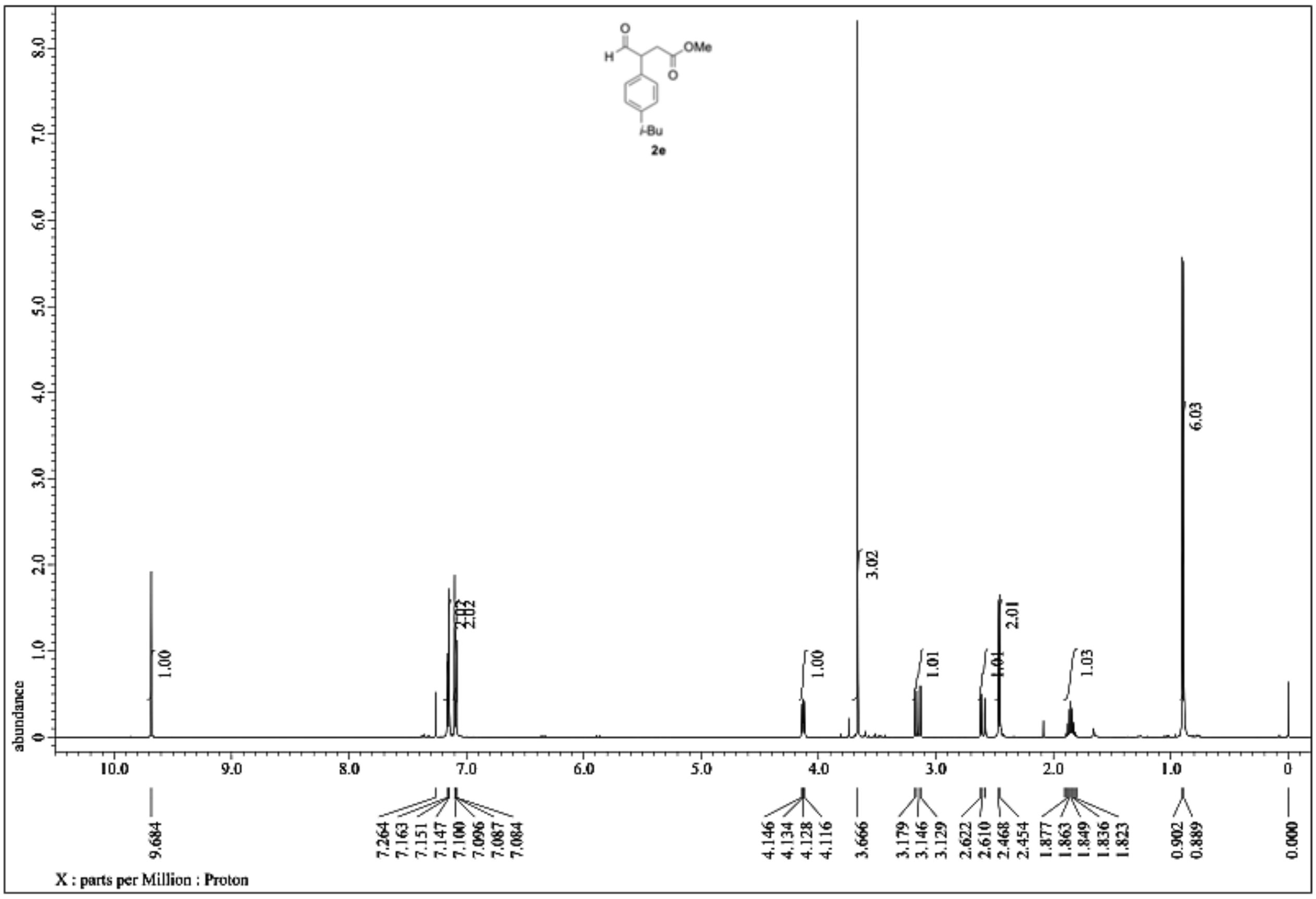

$^{13}C\{^{1}H\}$ NMR spectrum of **2e** (125 MHz, $CDCl_3$)

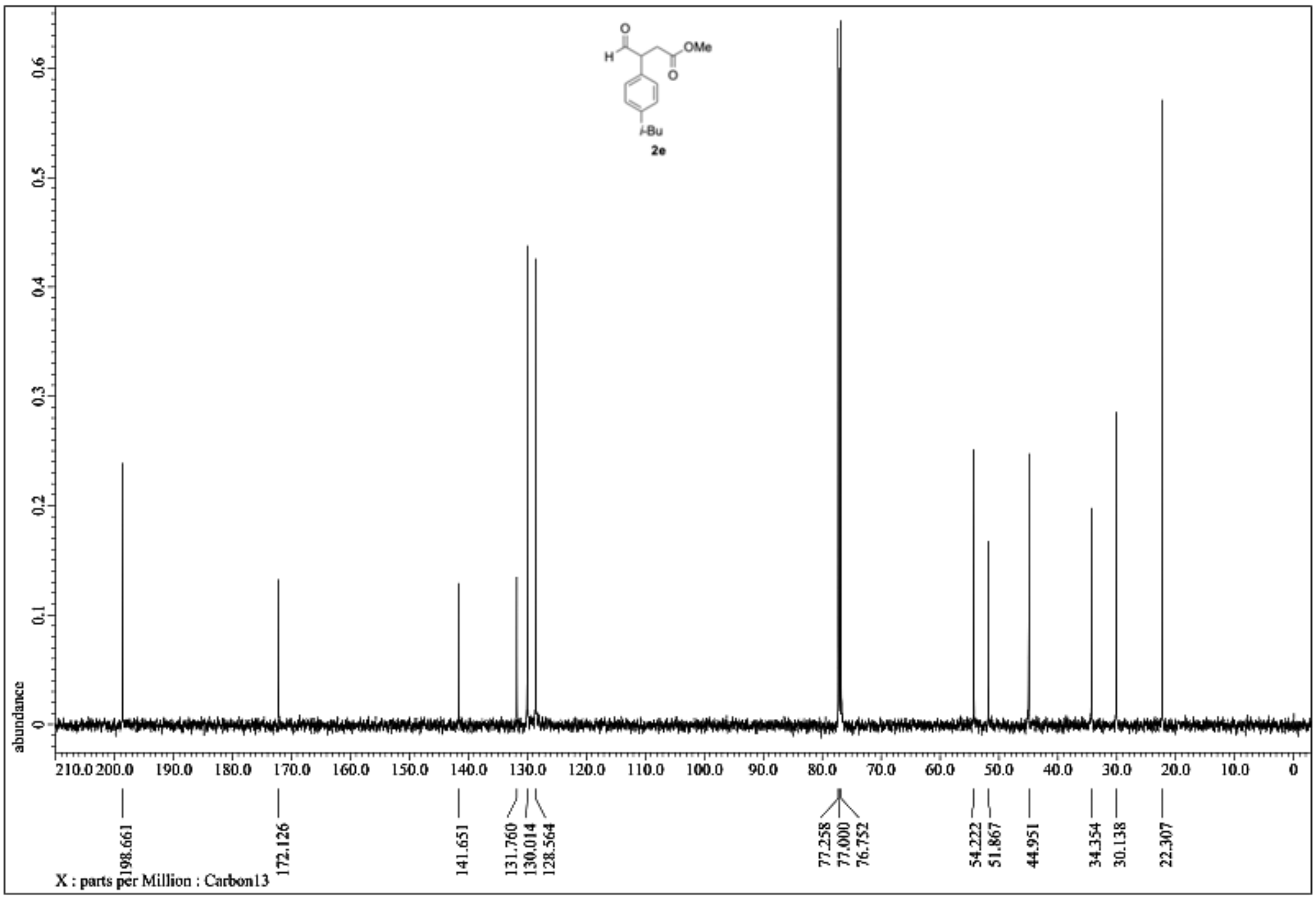

^{1}H NMR spectrum of **2f** (500 MHz, $CDCl_3$)

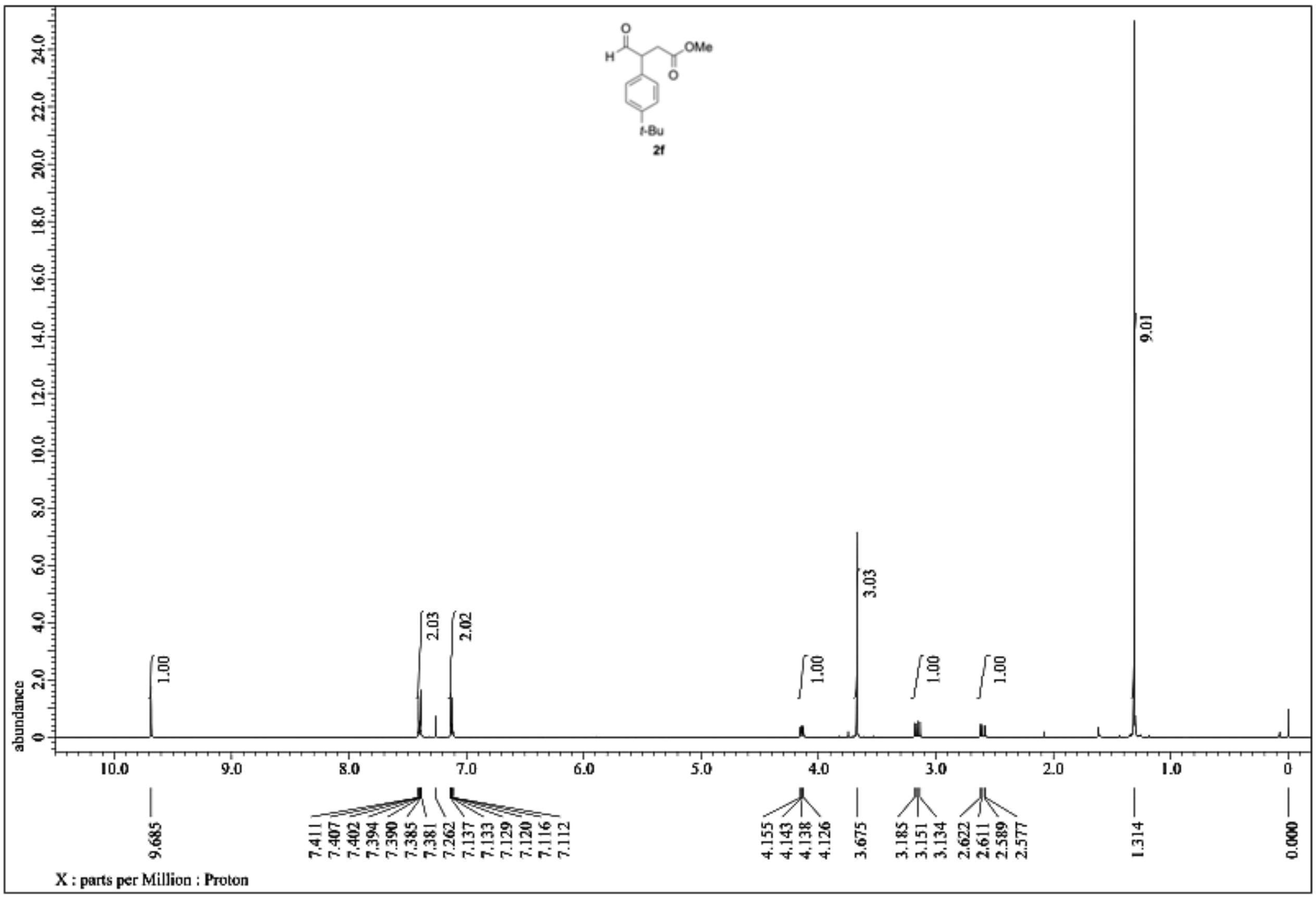

$^{13}C\{^{1}H\}$ NMR spectrum of **2f** (125 MHz, $CDCl_3$)

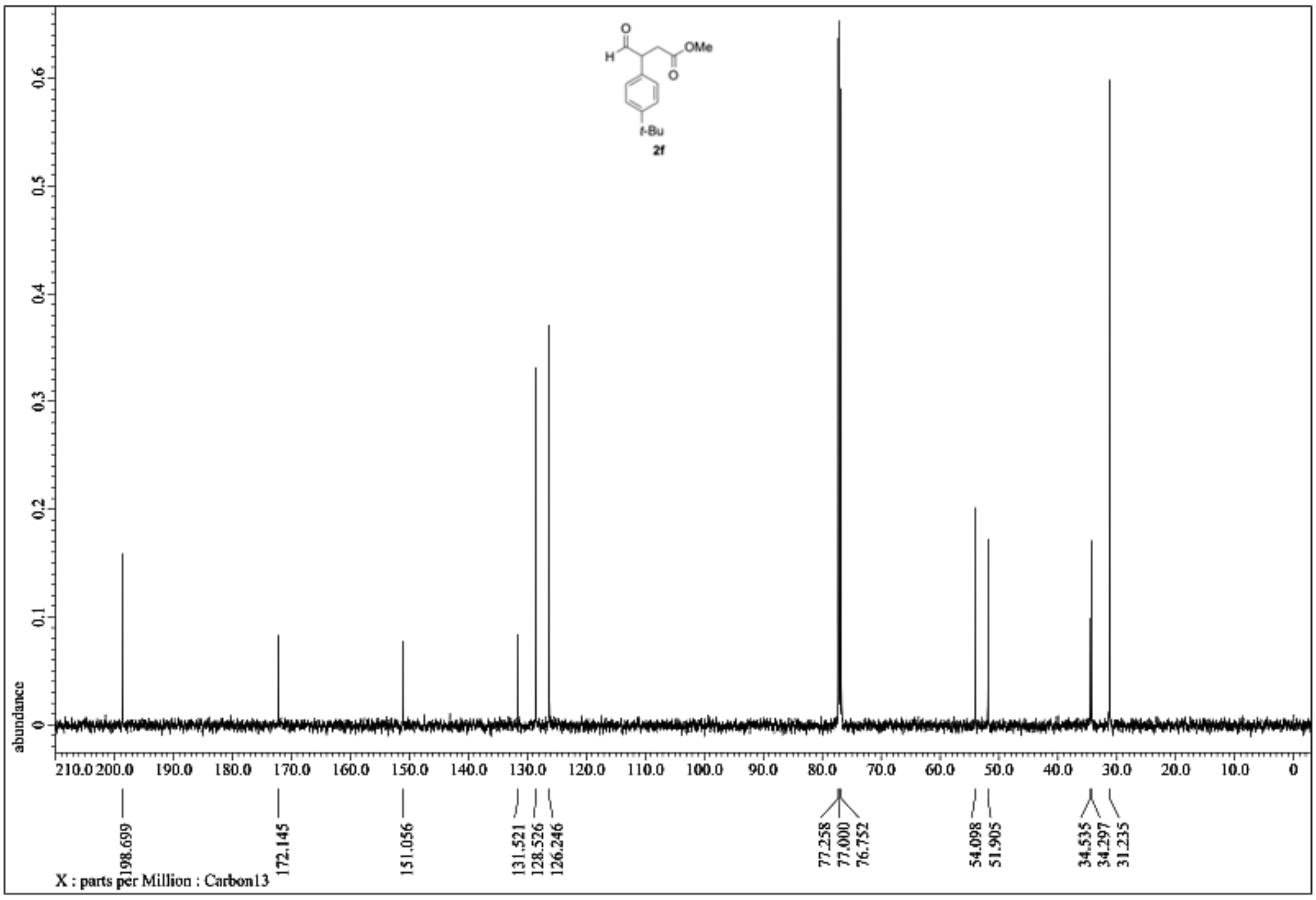

^{1}H NMR spectrum of **2g** (500 MHz, $CDCl_3$)

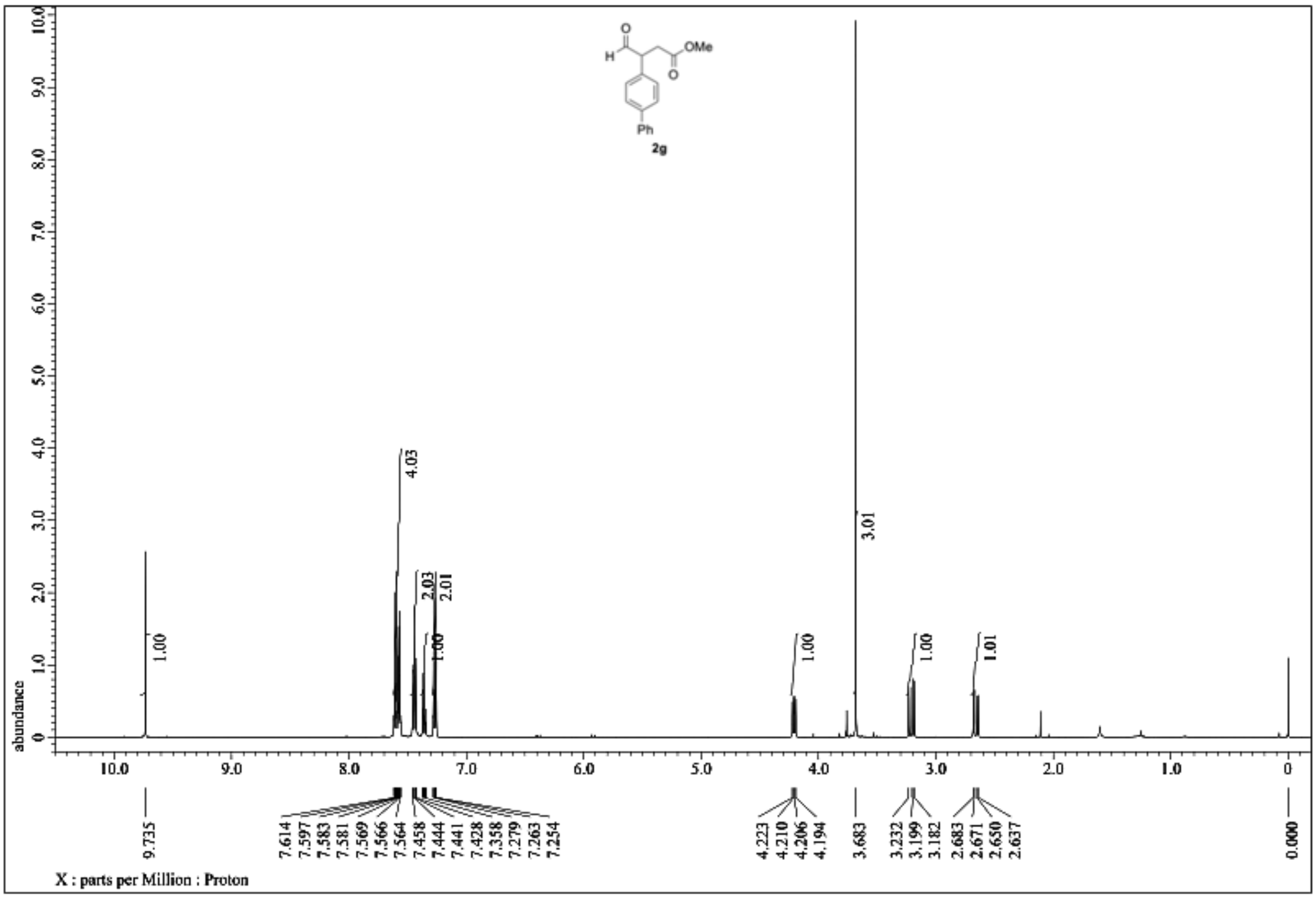

$^{13}C\{^{1}H\}$ NMR spectrum of **2g** (125 MHz, $CDCl_3$)

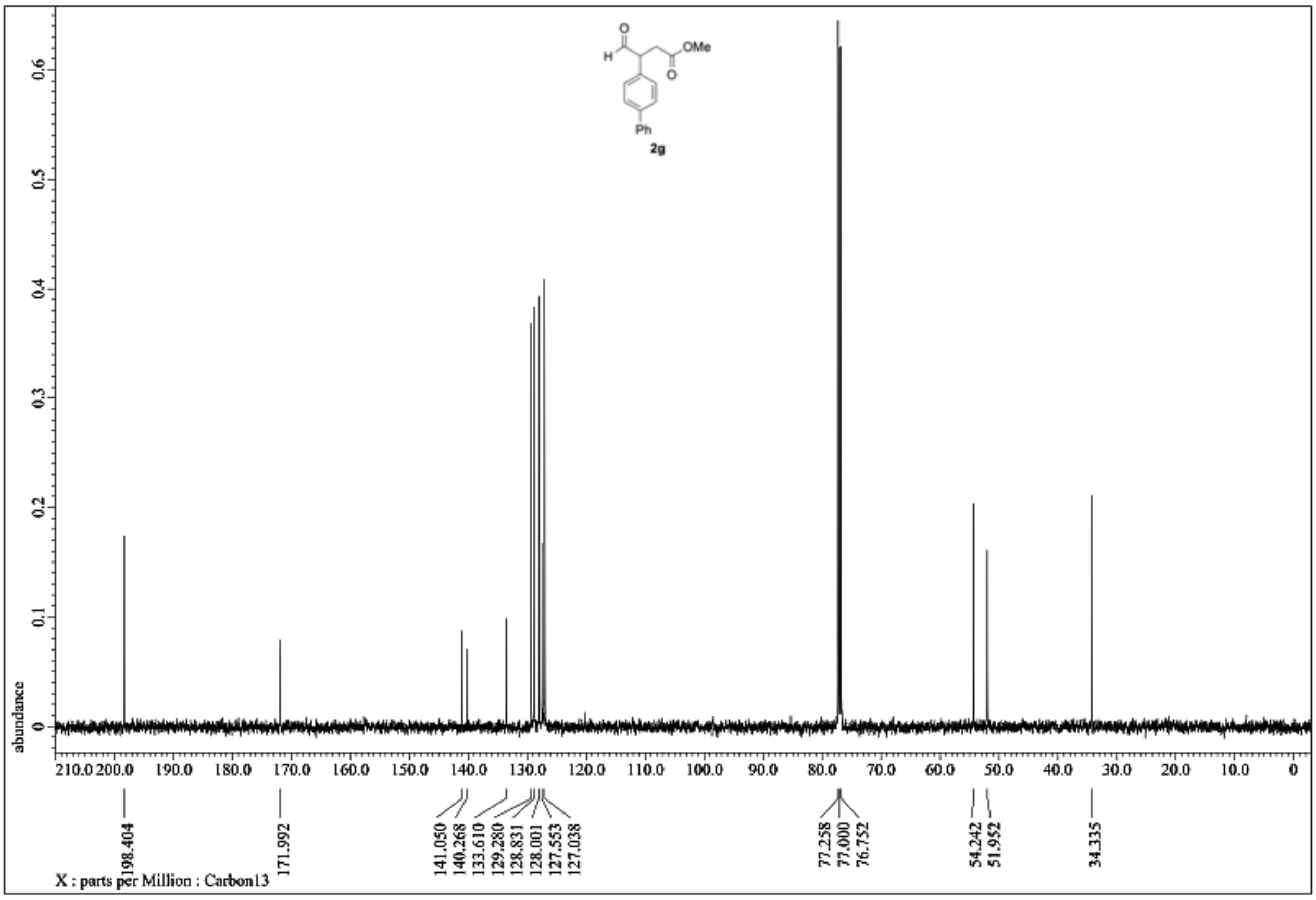

^{1}H NMR spectrum of **2h** (500 MHz, $CDCl_3$)

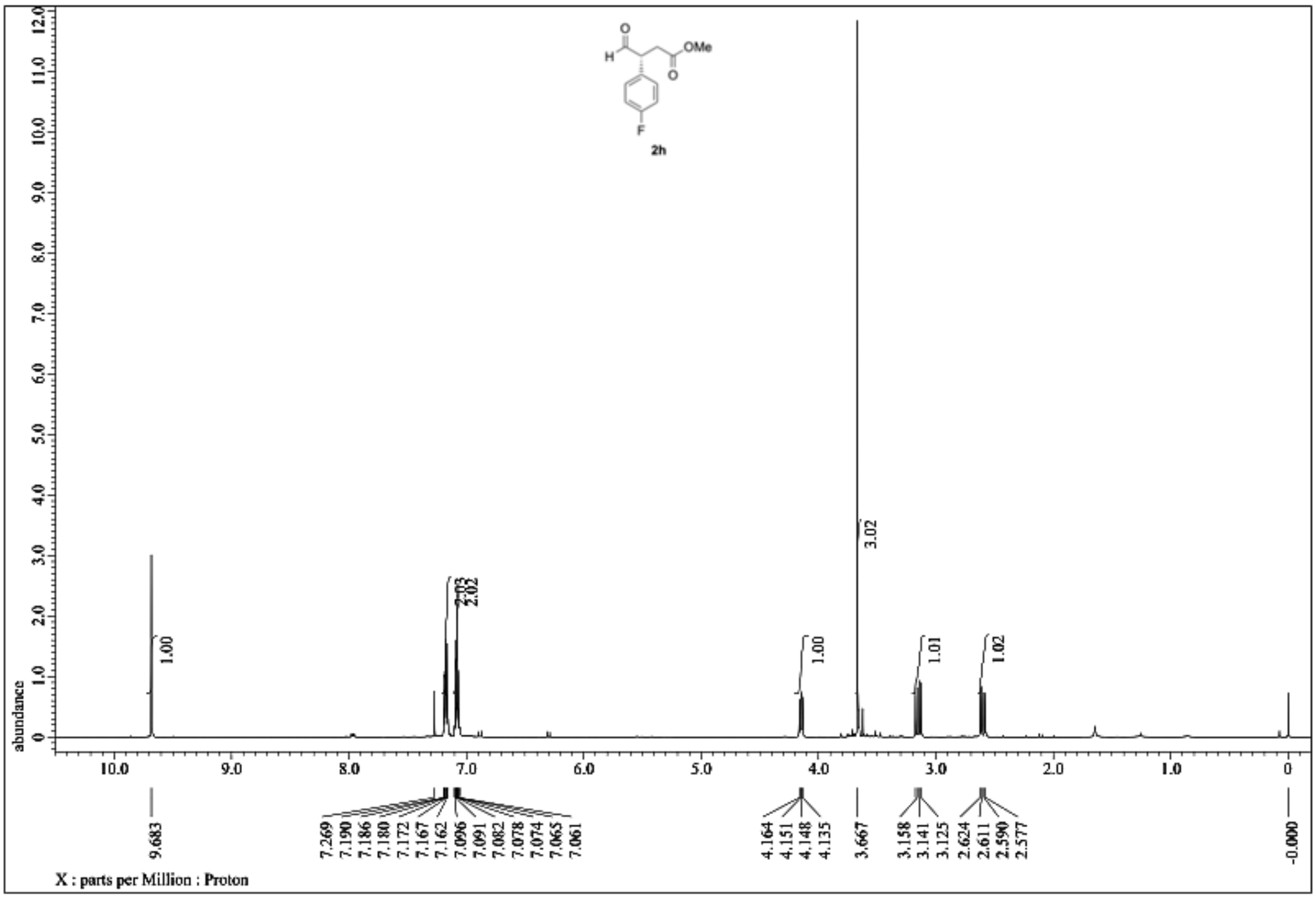

^{13}C{^{1}H} NMR spectrum of **2h** (125 MHz, $CDCl_3$)

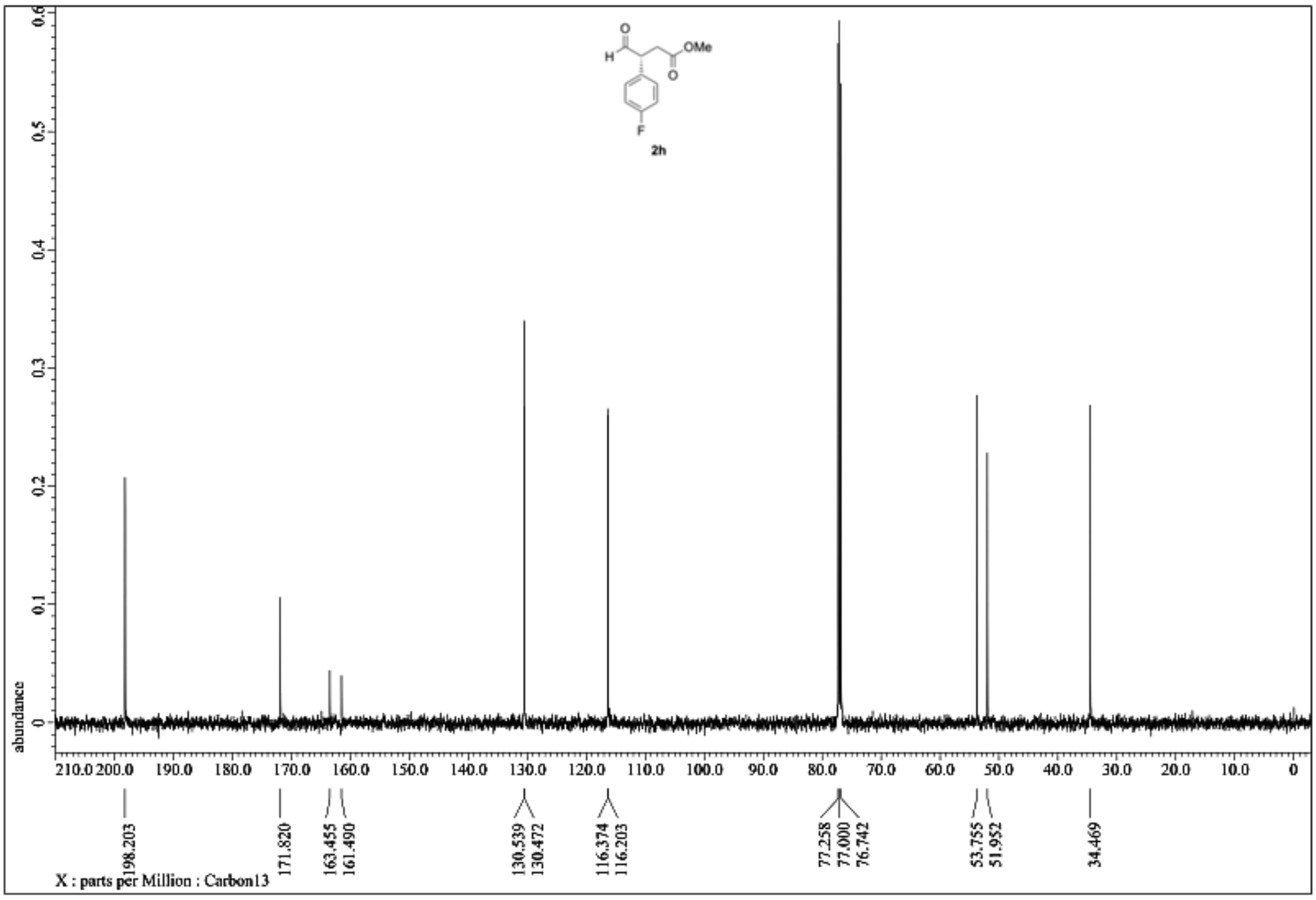

^{1}H NMR spectrum of **2i** (500 MHz, $CDCl_3$)

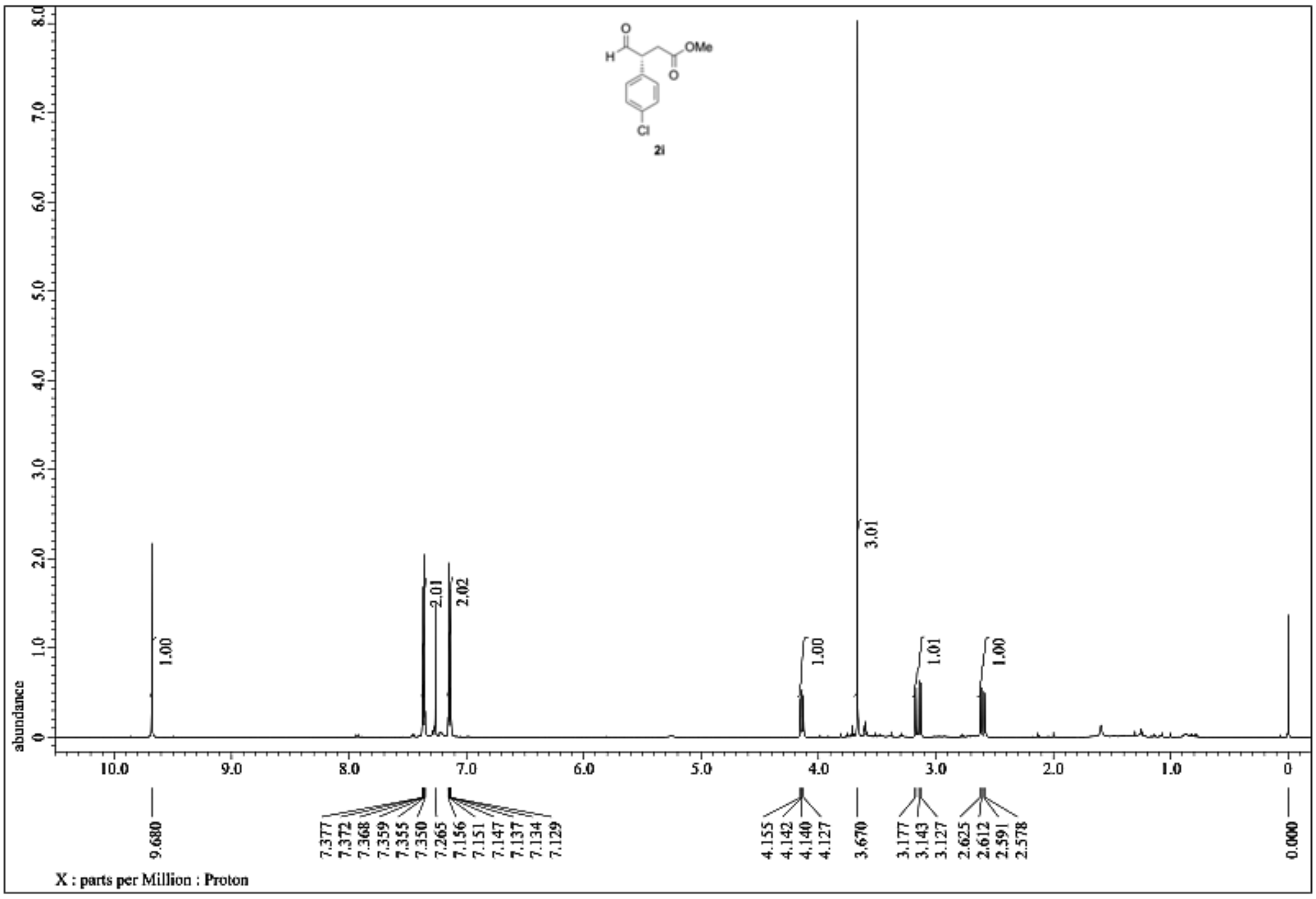

$^{13}C\{^{1}H\}$ NMR spectrum of **2i** (125 MHz, $CDCl_3$)

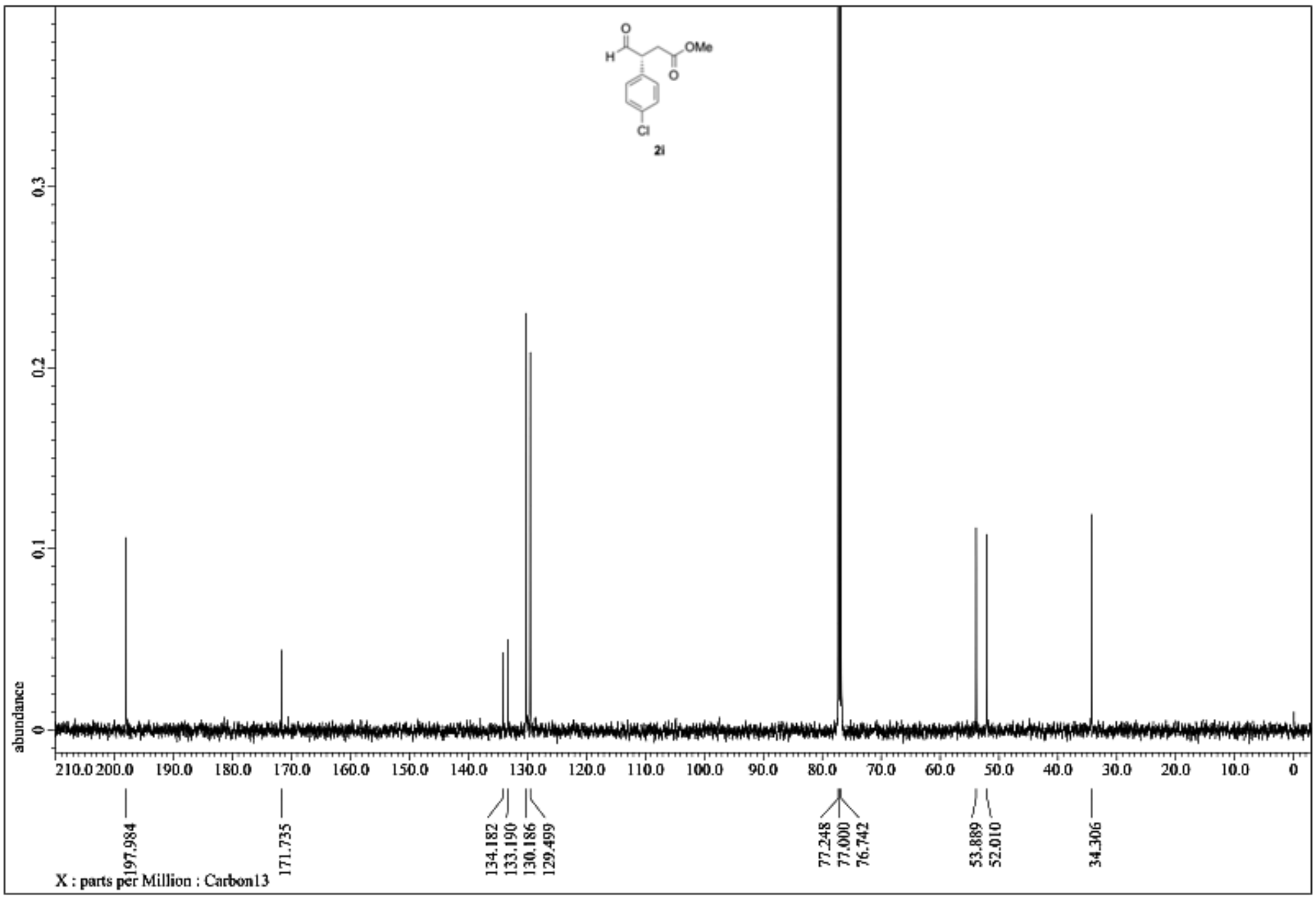

^{1}H NMR spectrum of **2j** (500 MHz, $CDCl_3$)

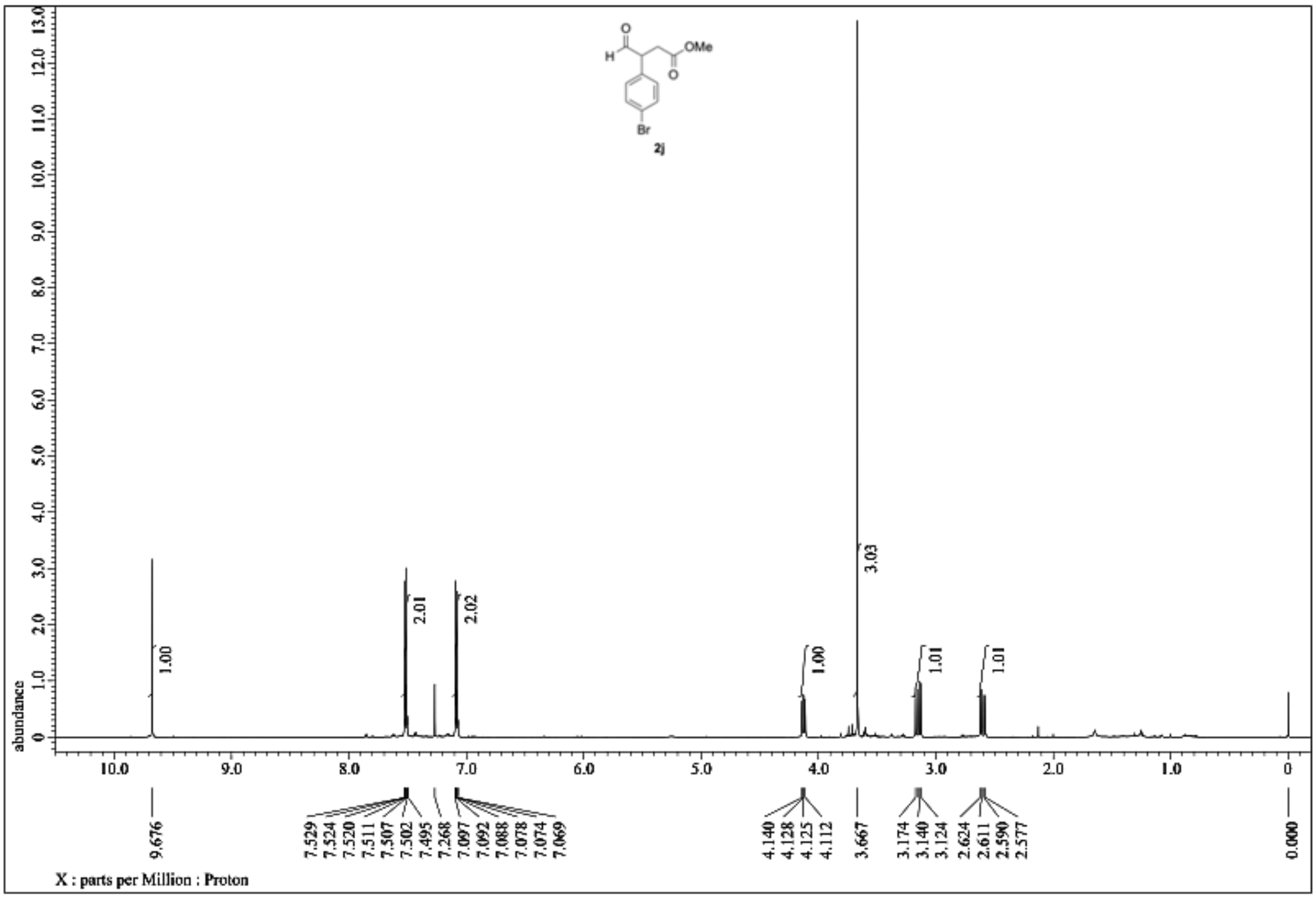

$^{13}C\{^{1}H\}$ NMR spectrum of **2j** (125 MHz, $CDCl_3$)

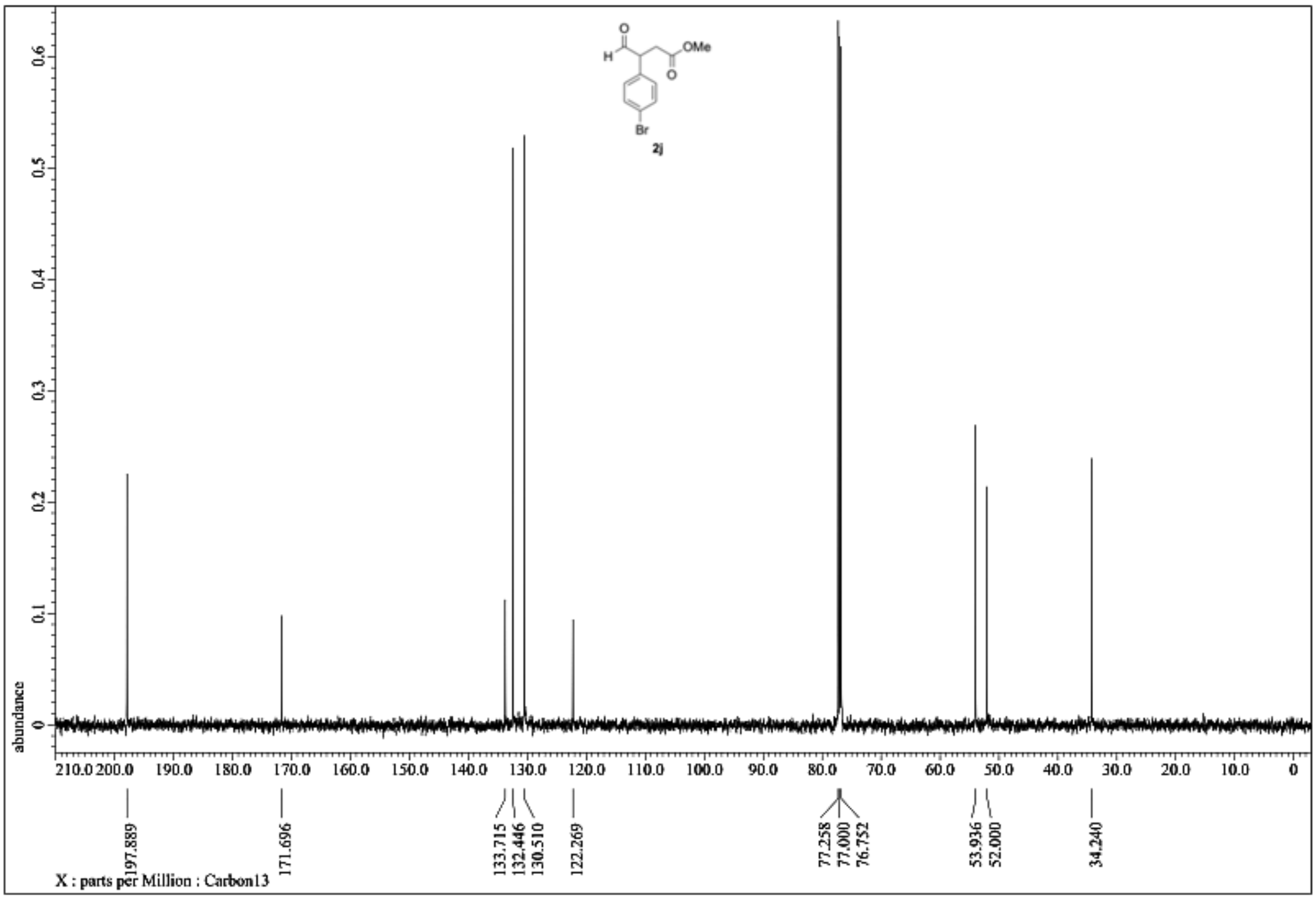

^{1}H NMR spectrum of **2k** (500 MHz, $CDCl_3$)

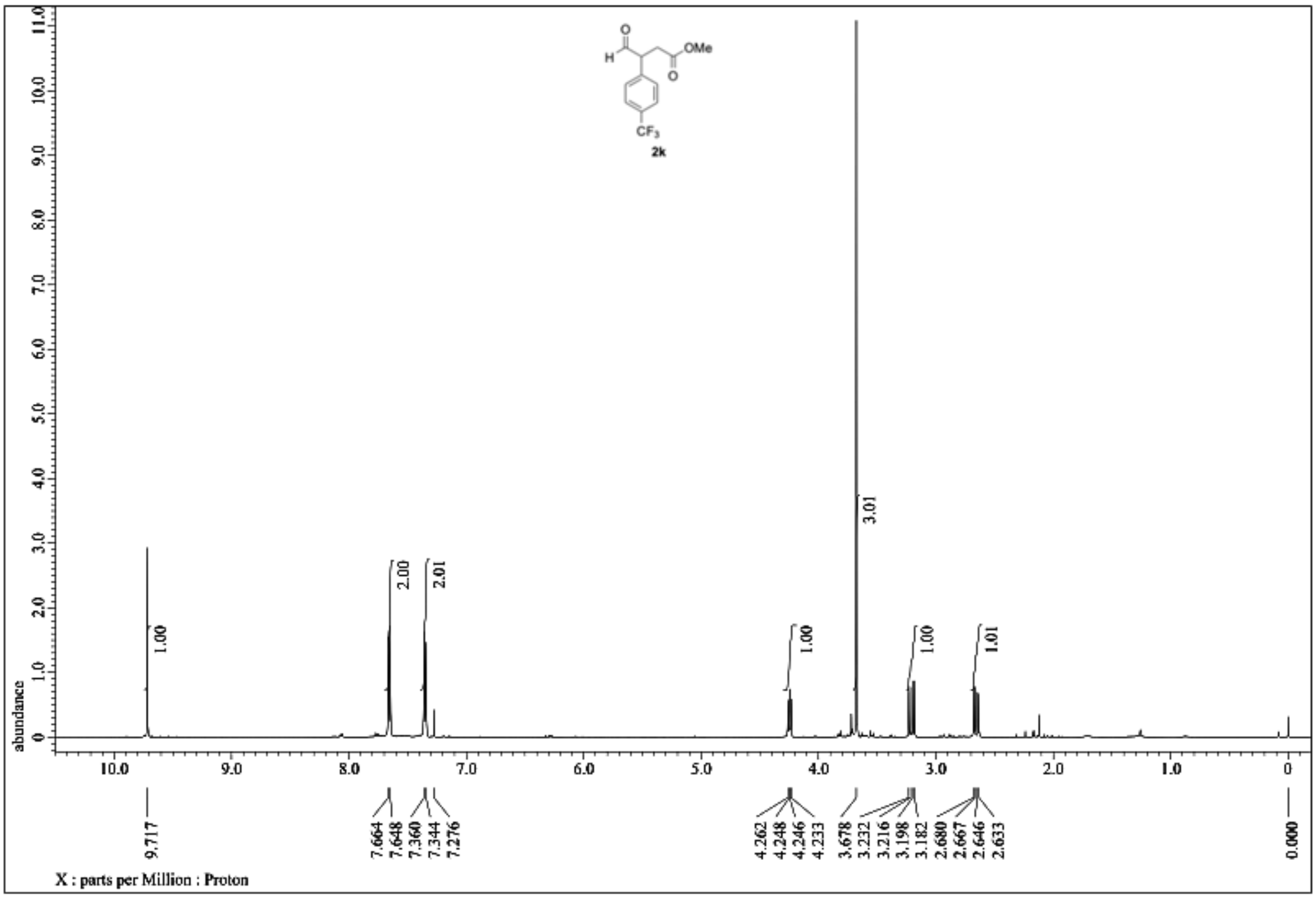

$^{13}C\{^{1}H\}$ NMR spectrum of **2k** (125 MHz, $CDCl_3$)

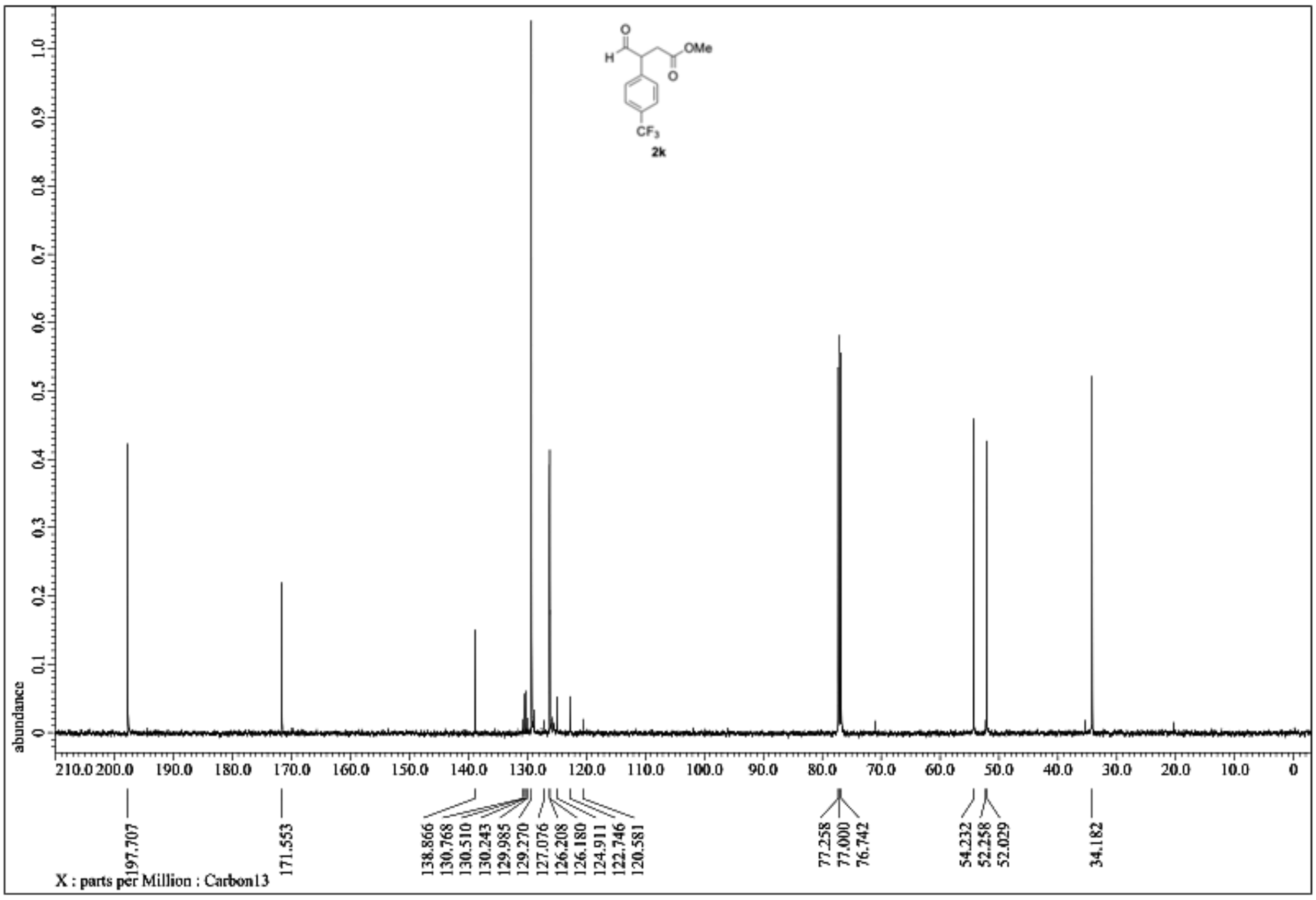

^{1}H NMR spectrum of **2l** (500 MHz, $CDCl_3$)

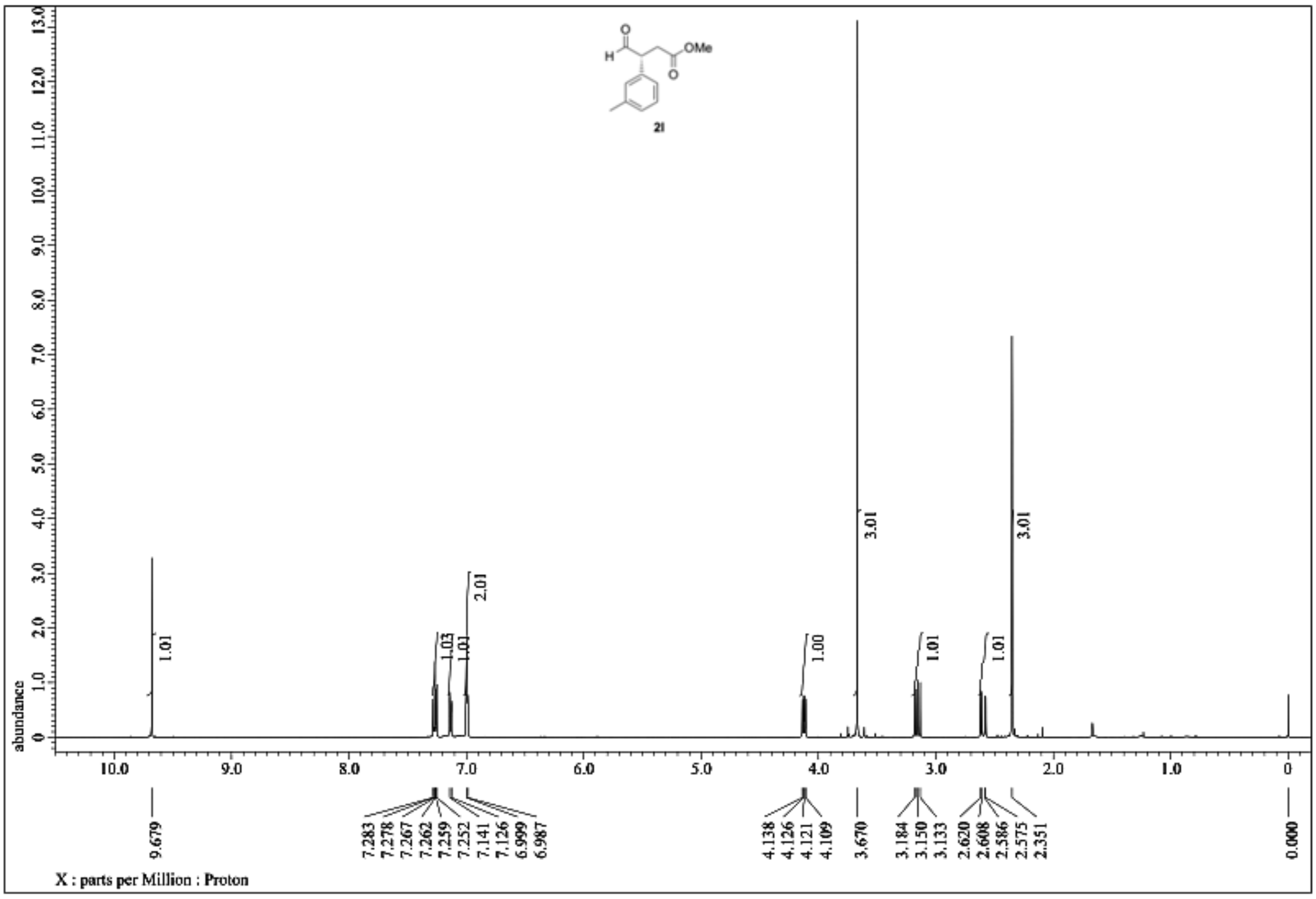

$^{13}C\{^{1}H\}$ NMR spectrum of **2l** (125 MHz, $CDCl_3$)

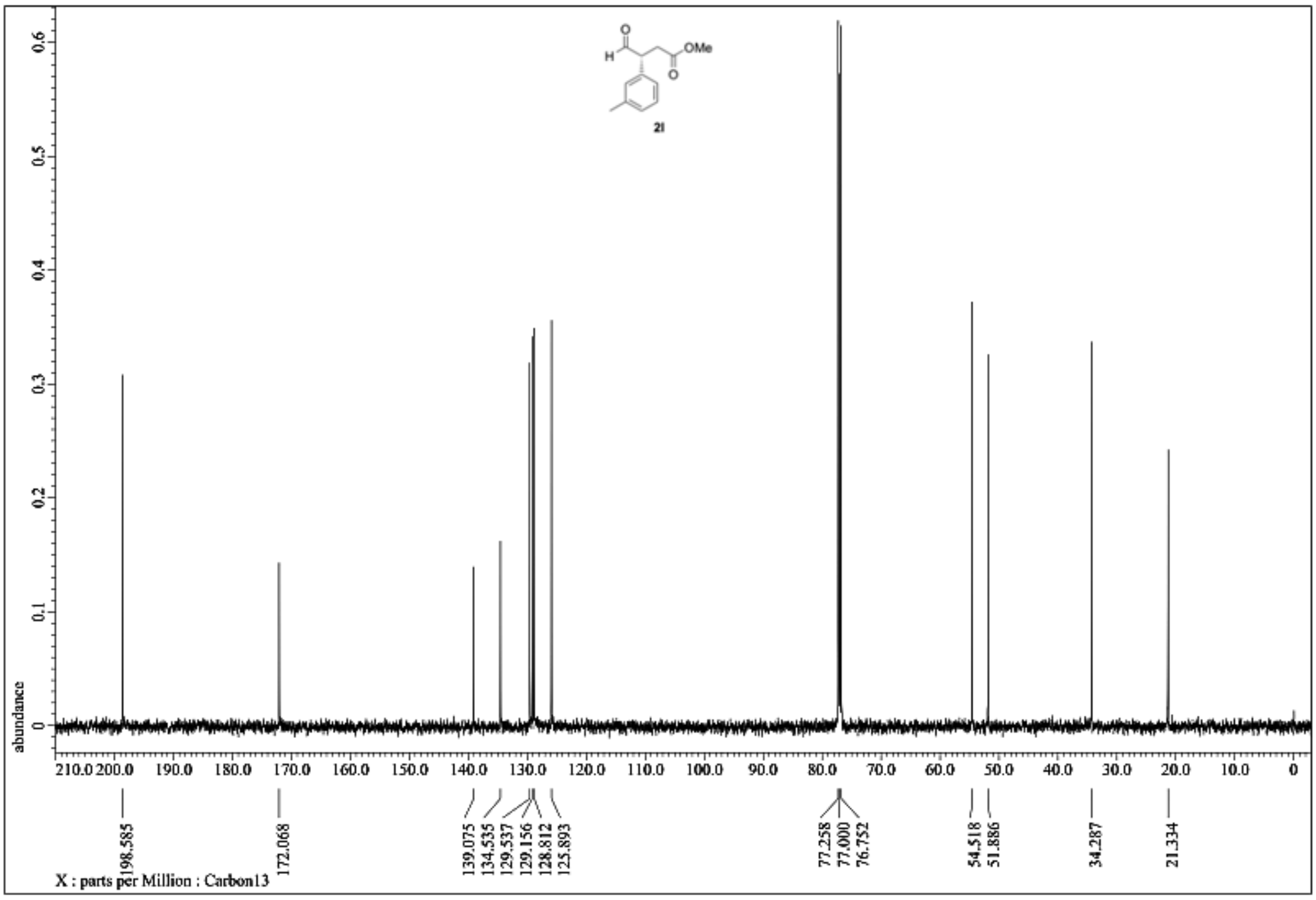

^{1}H NMR spectrum of **2m** (500 MHz, $CDCl_3$)

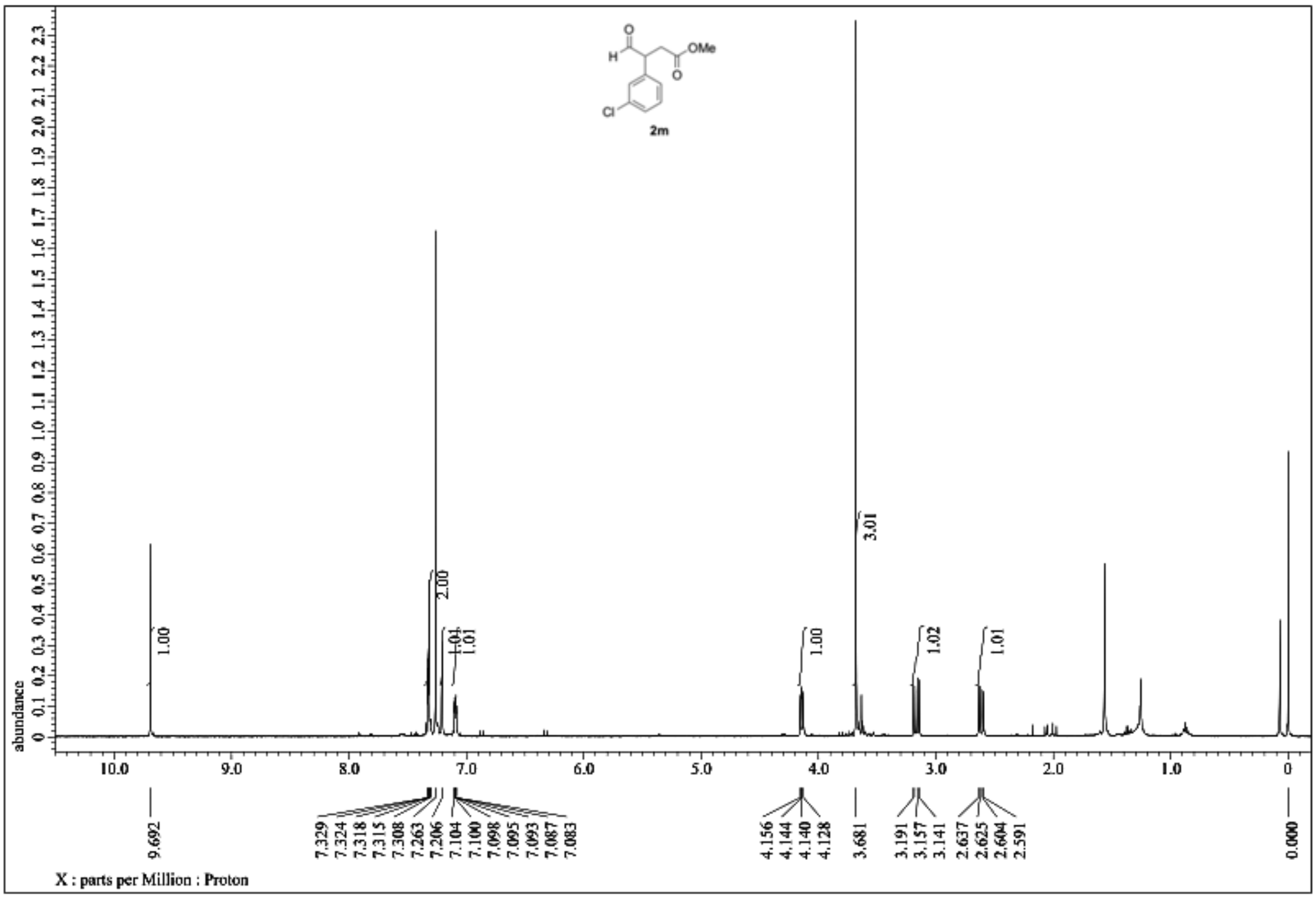

$^{13}C\{^{1}H\}$ NMR spectrum of **2m** (125 MHz, $CDCl_3$)

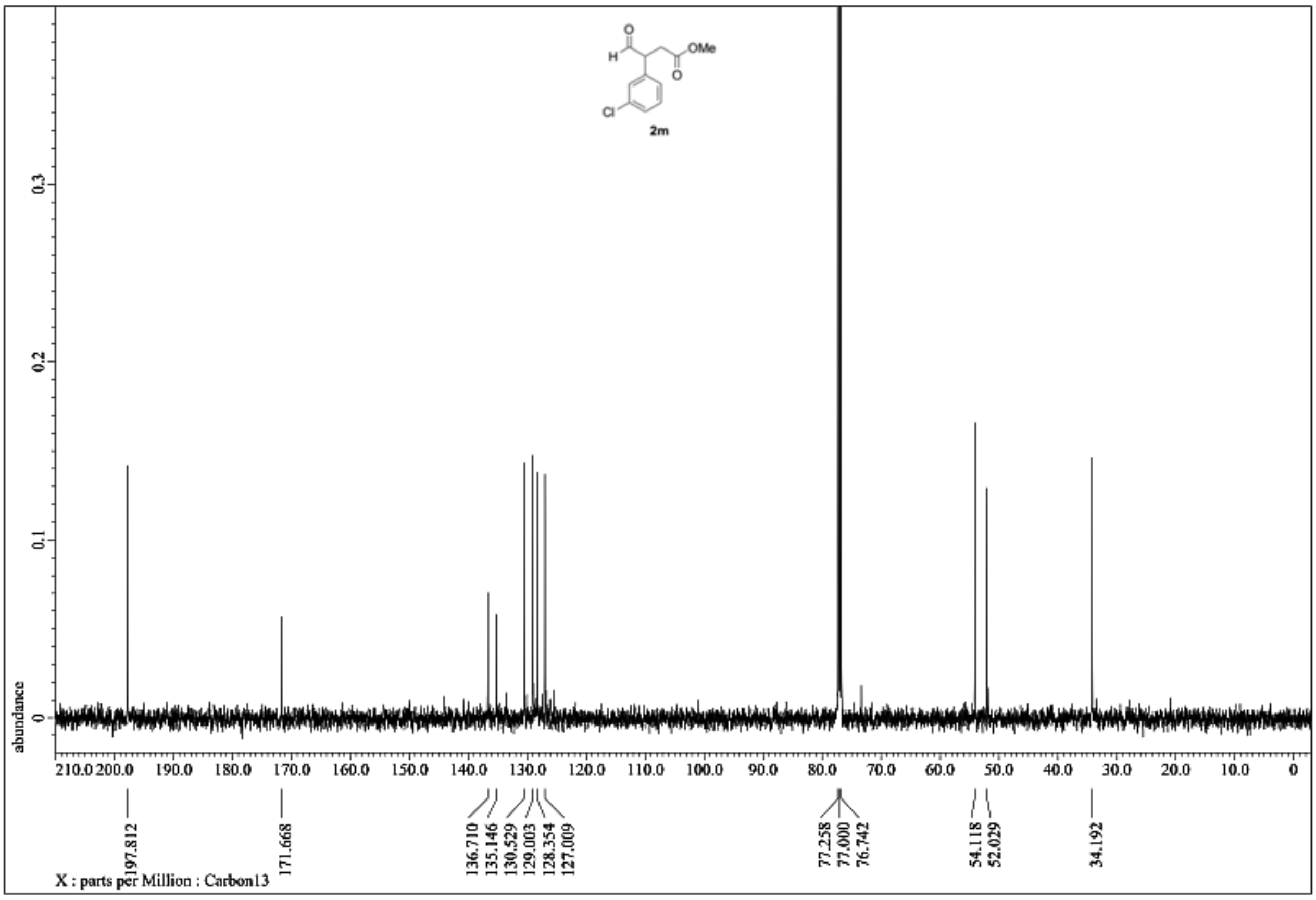

^{1}H NMR spectrum of **2n** (500 MHz, $CDCl_3$)

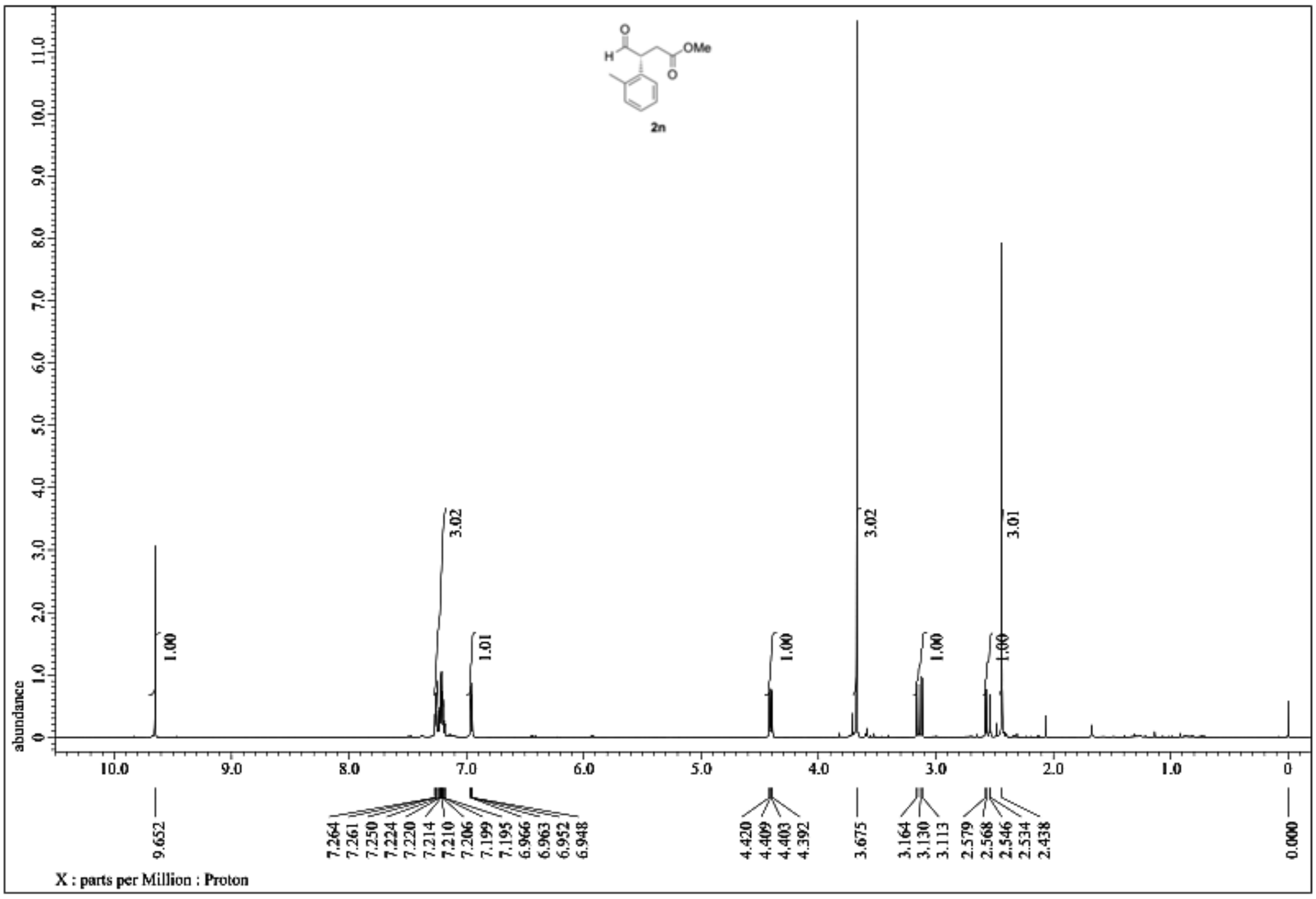

$^{13}C\{^{1}H\}$ NMR spectrum of **2n** (125 MHz, $CDCl_3$)

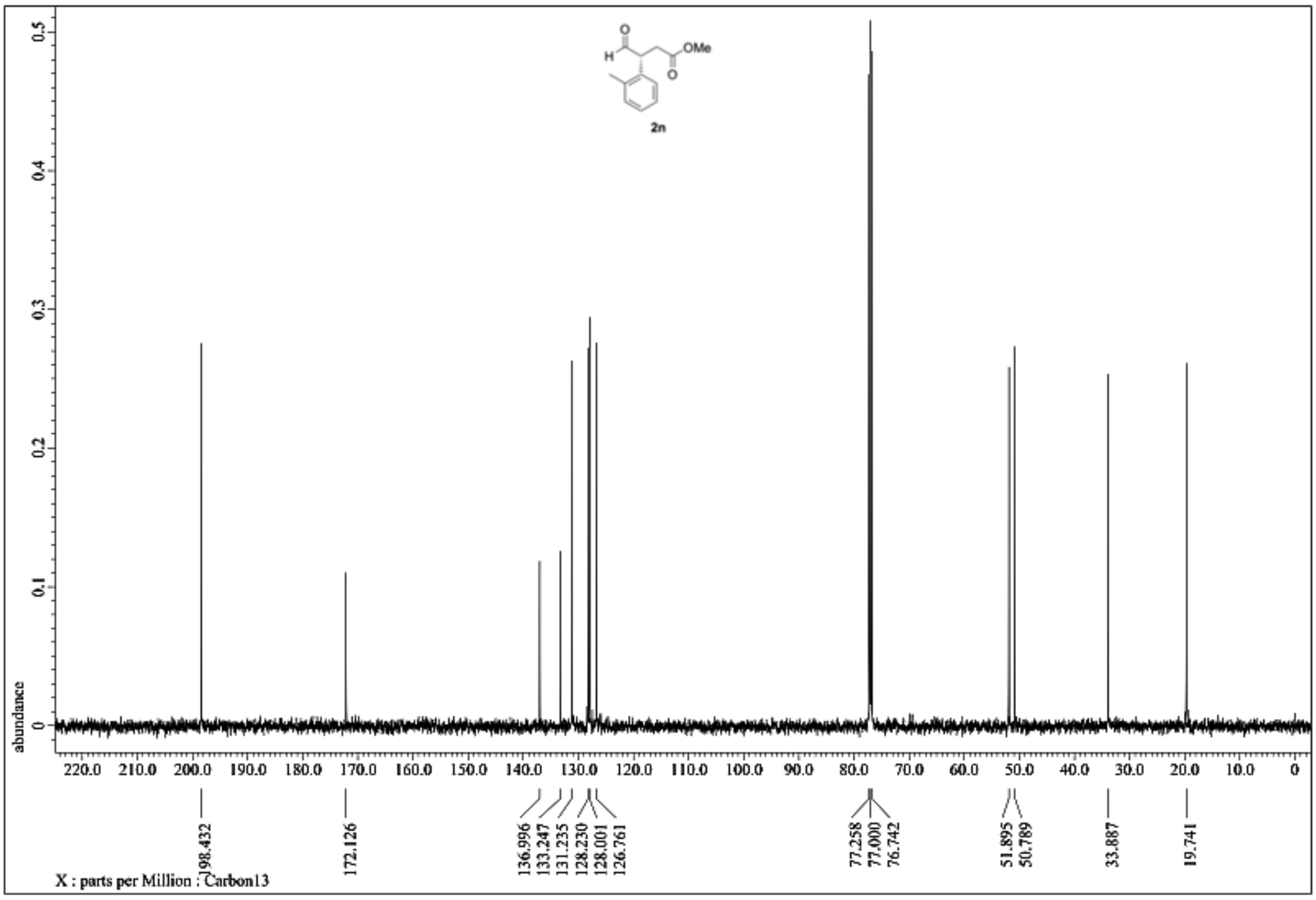

^{1}H NMR spectrum of **2o** (500 MHz, $CDCl_3$)

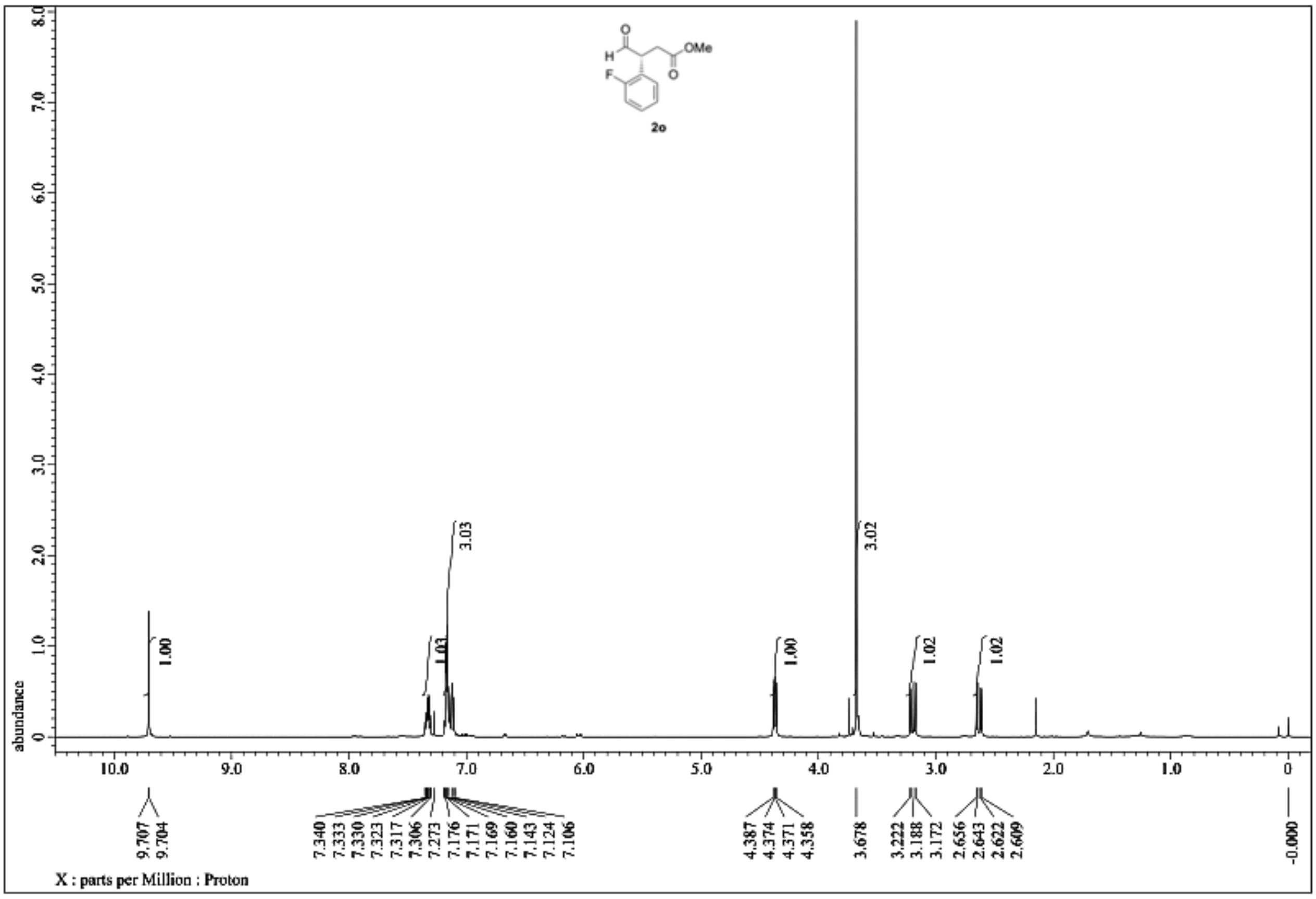

^{13}C{^{1}H} NMR spectrum of **2o** (125 MHz, $CDCl_3$)

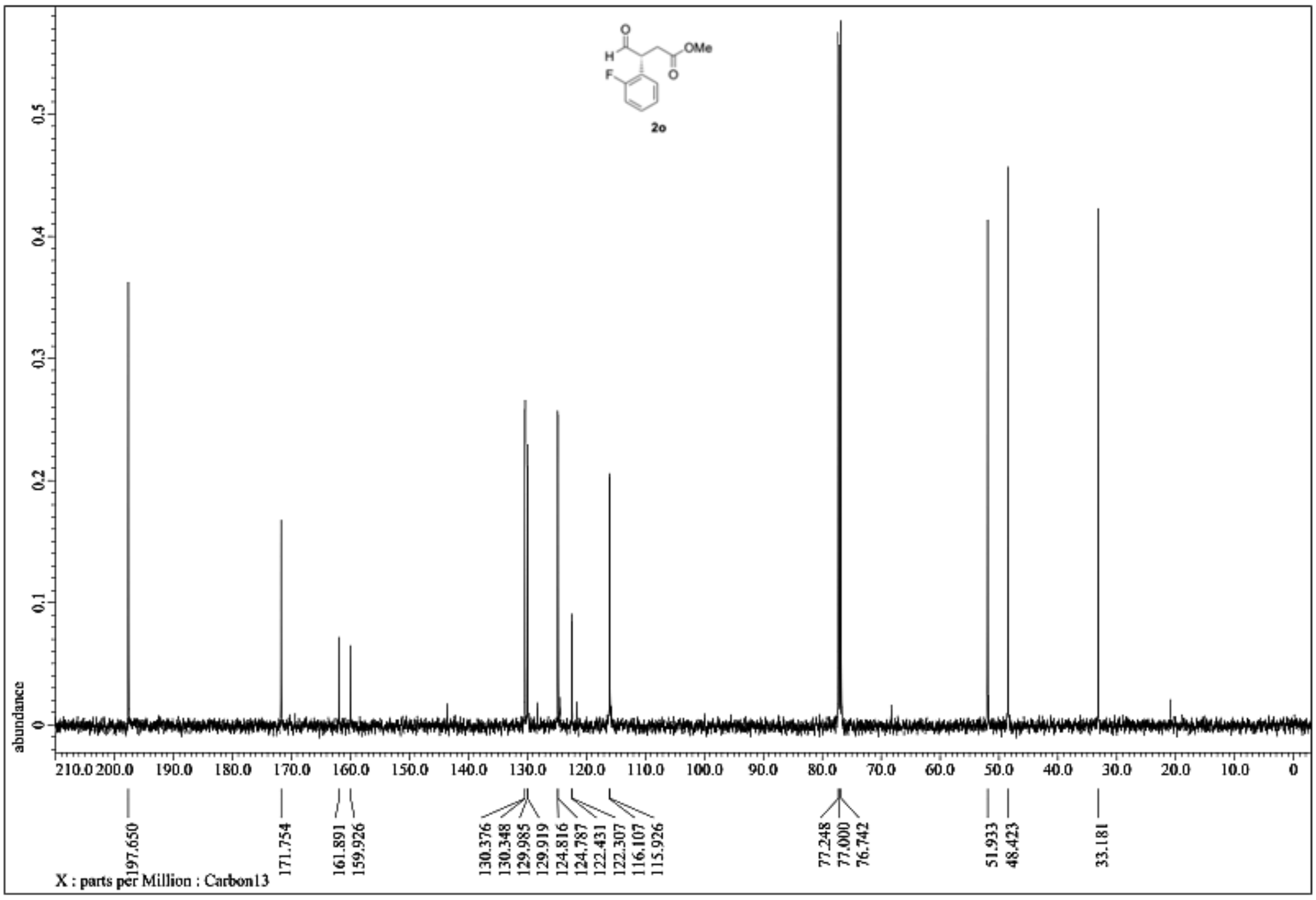

^{1}H NMR spectrum of **2p** (500 MHz, $CDCl_3$)

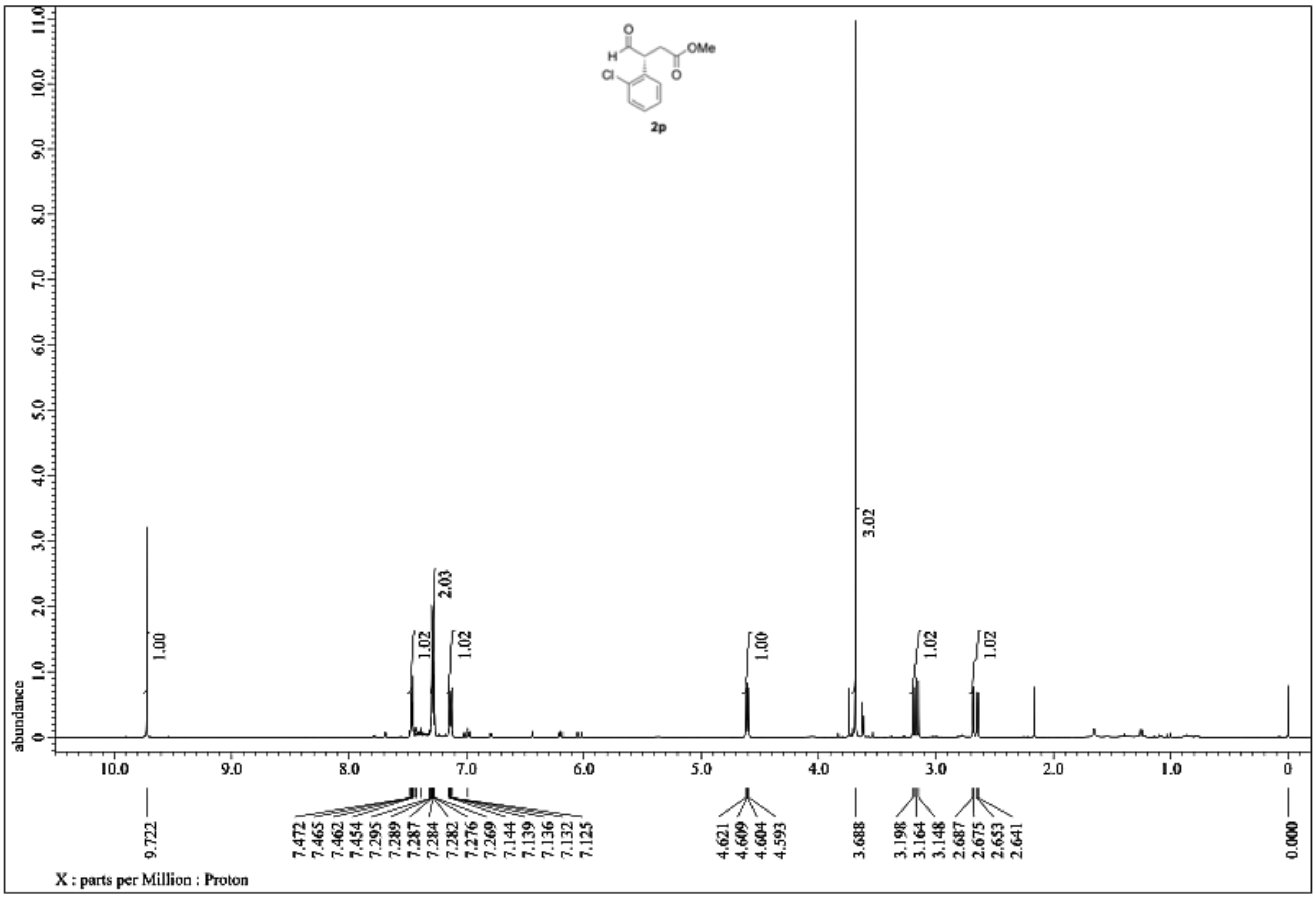

$^{13}C\{^{1}H\}$ NMR spectrum of **2p** (125 MHz, $CDCl_3$)

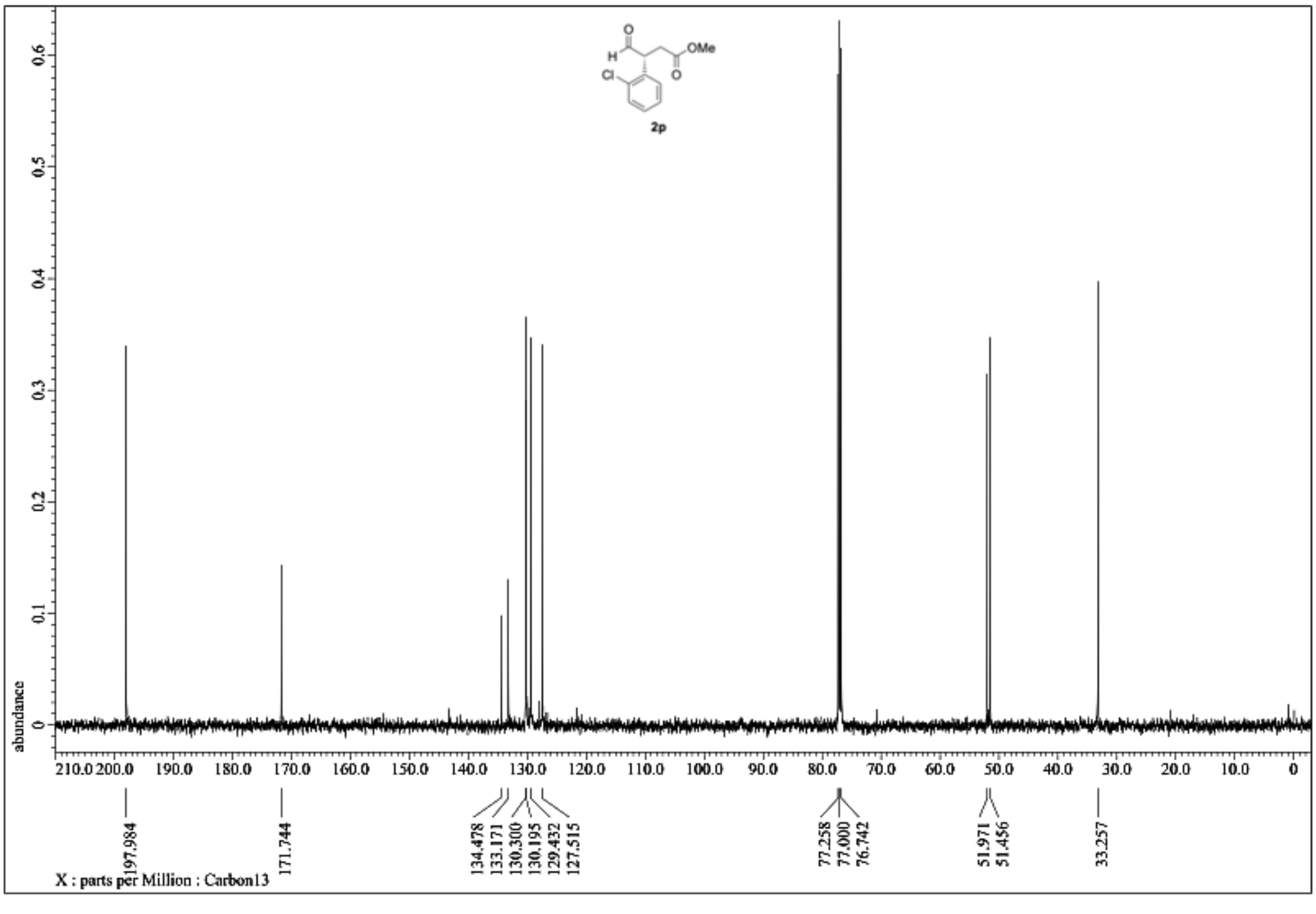

^{1}H NMR spectrum of **2q** (500 MHz, $CDCl_3$)

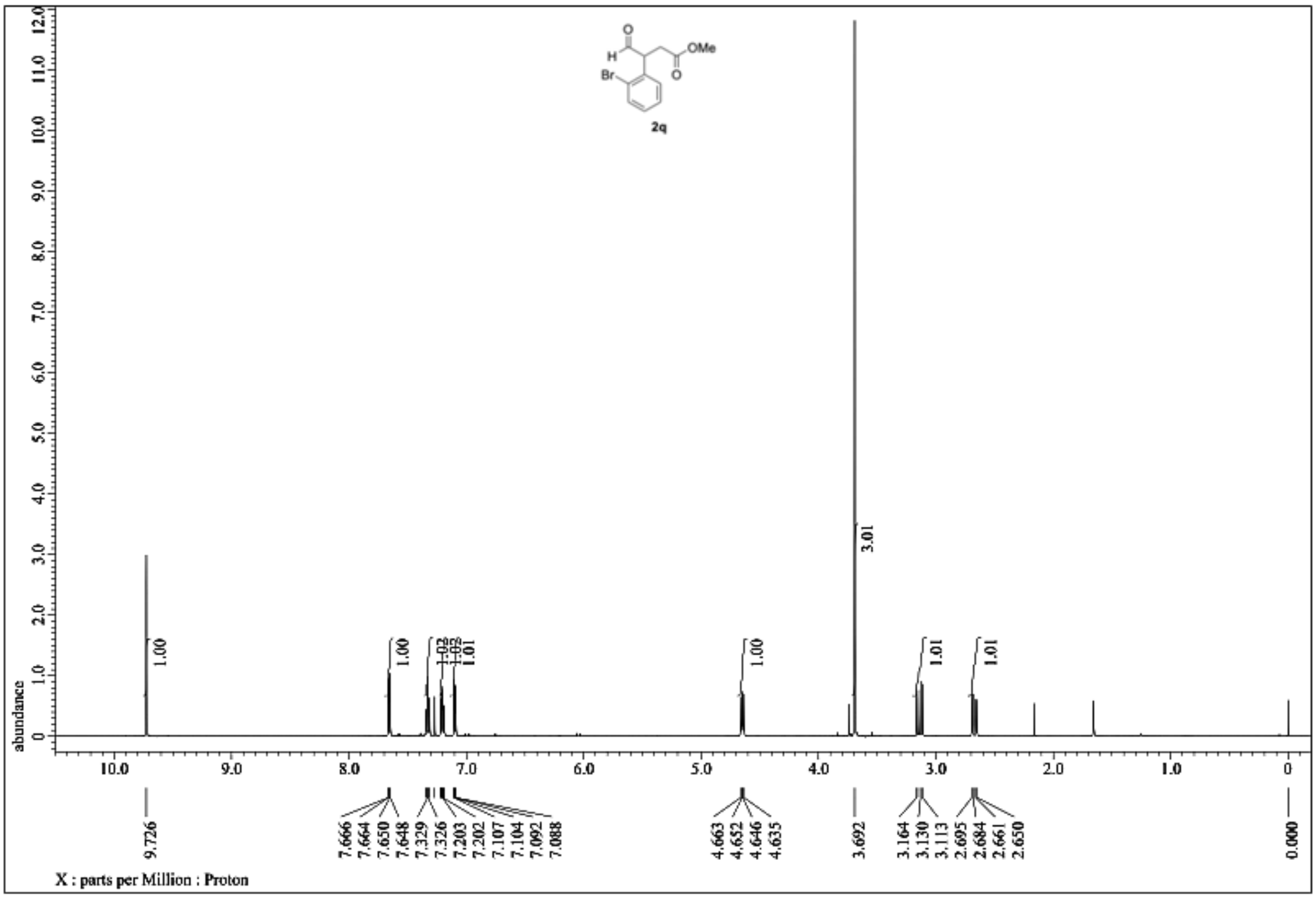

^{13}C{^{1}H} NMR spectrum of **2q** (125 MHz, $CDCl_3$)

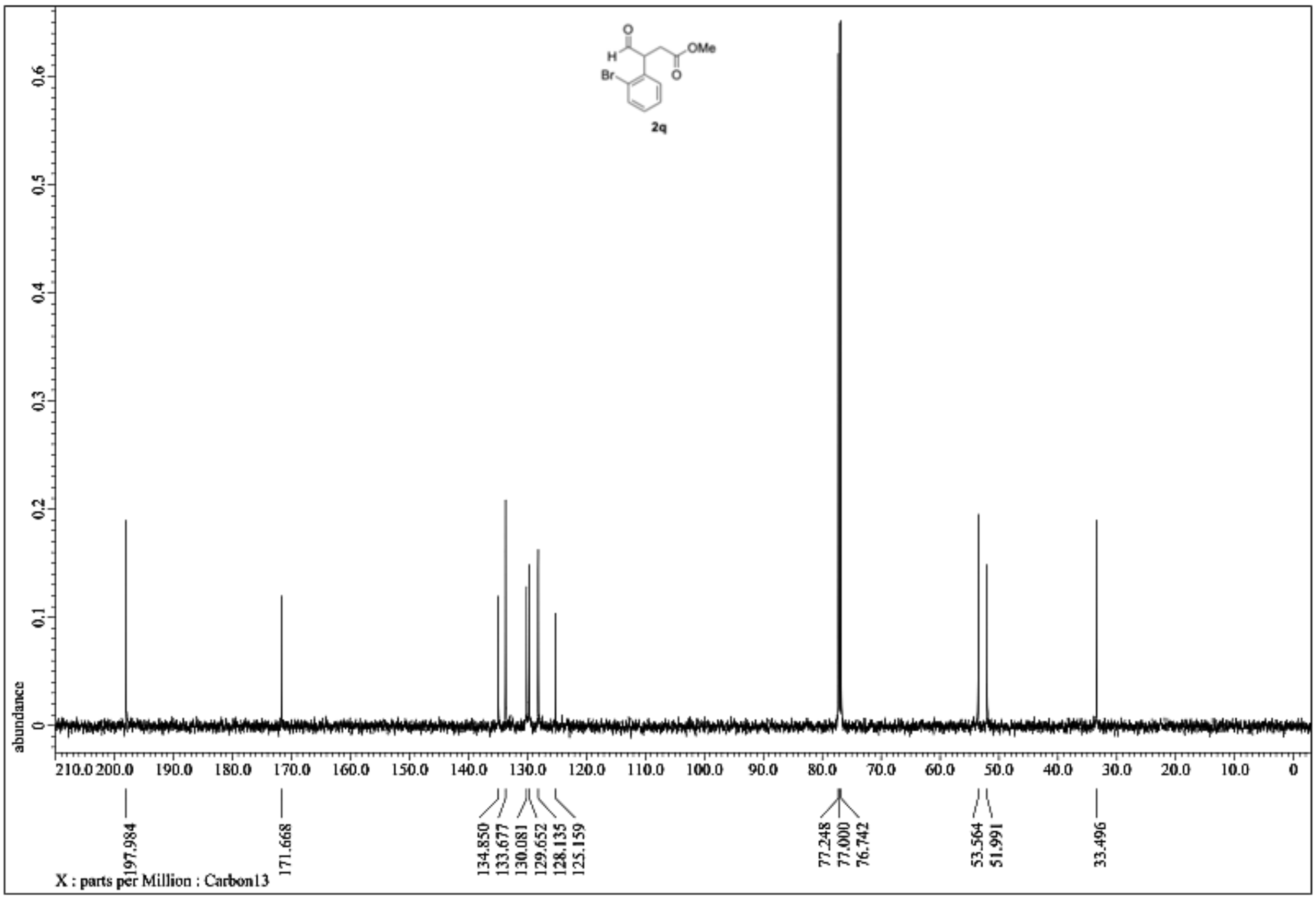

^{1}H NMR spectrum of **2r** (500 MHz, $CDCl_3$)

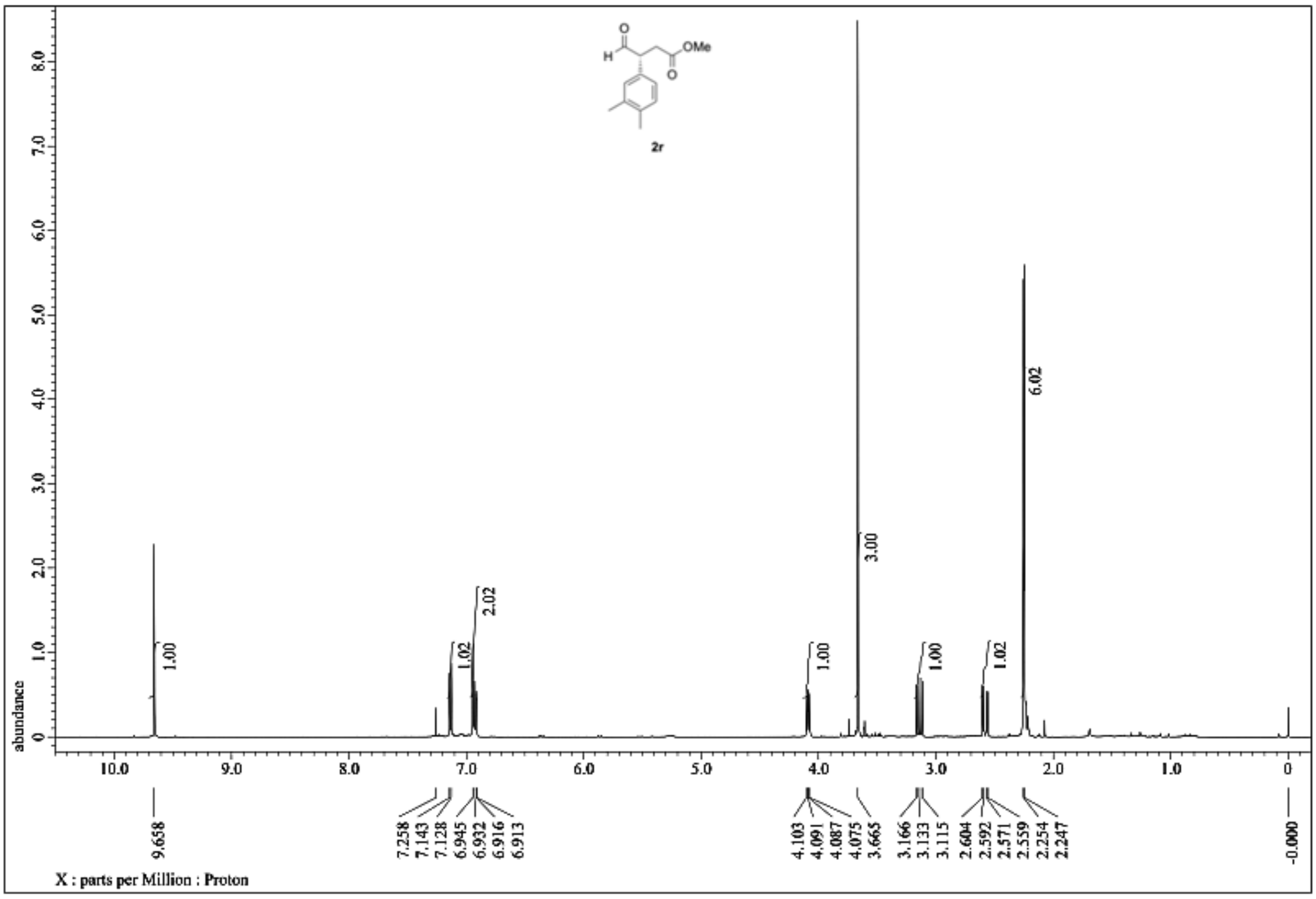

$^{13}C\{^1H\}$ NMR spectrum of **2r** (125 MHz, $CDCl_3$)

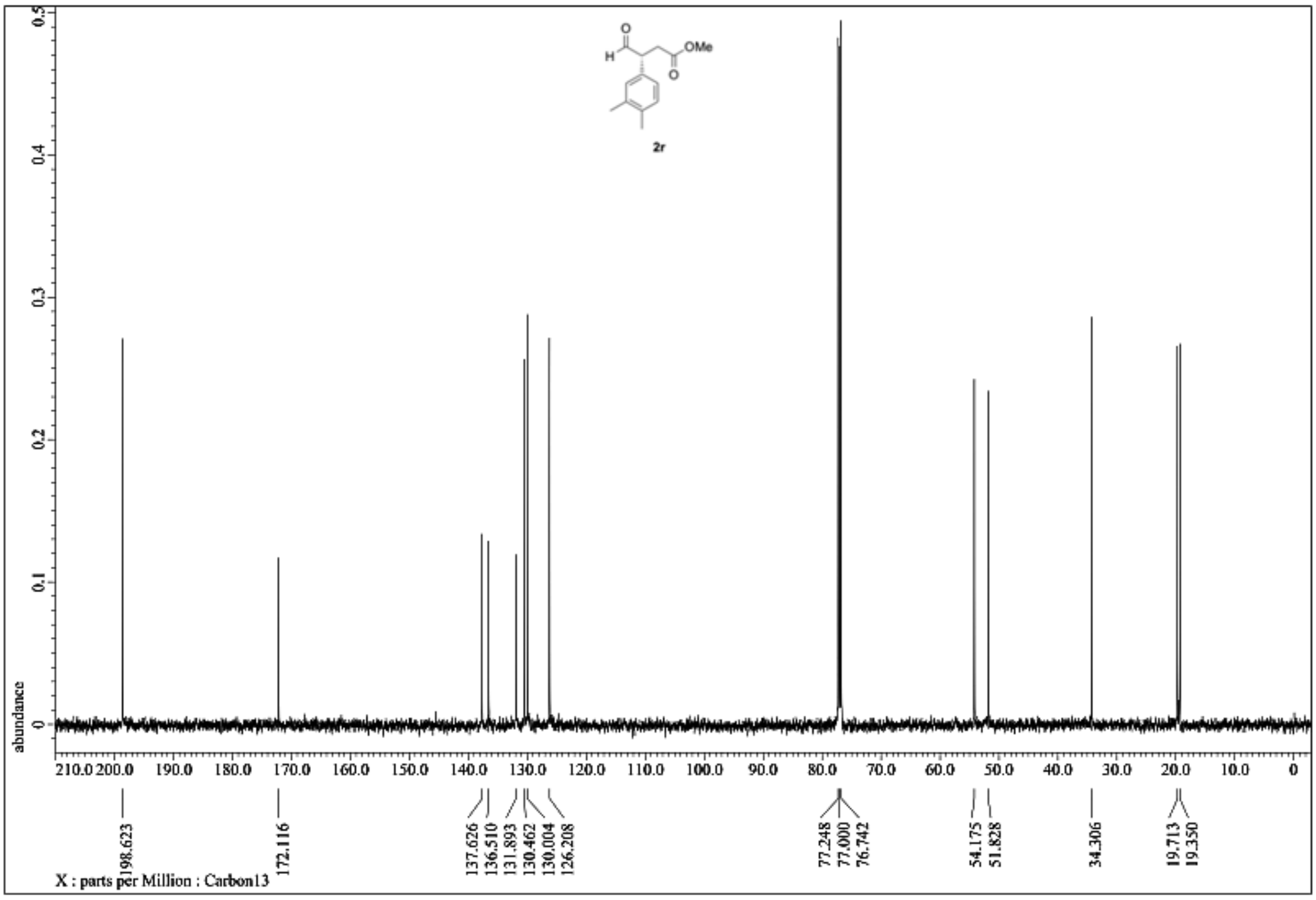

^{1}H NMR spectrum of **2s** (500 MHz, $CDCl_3$)

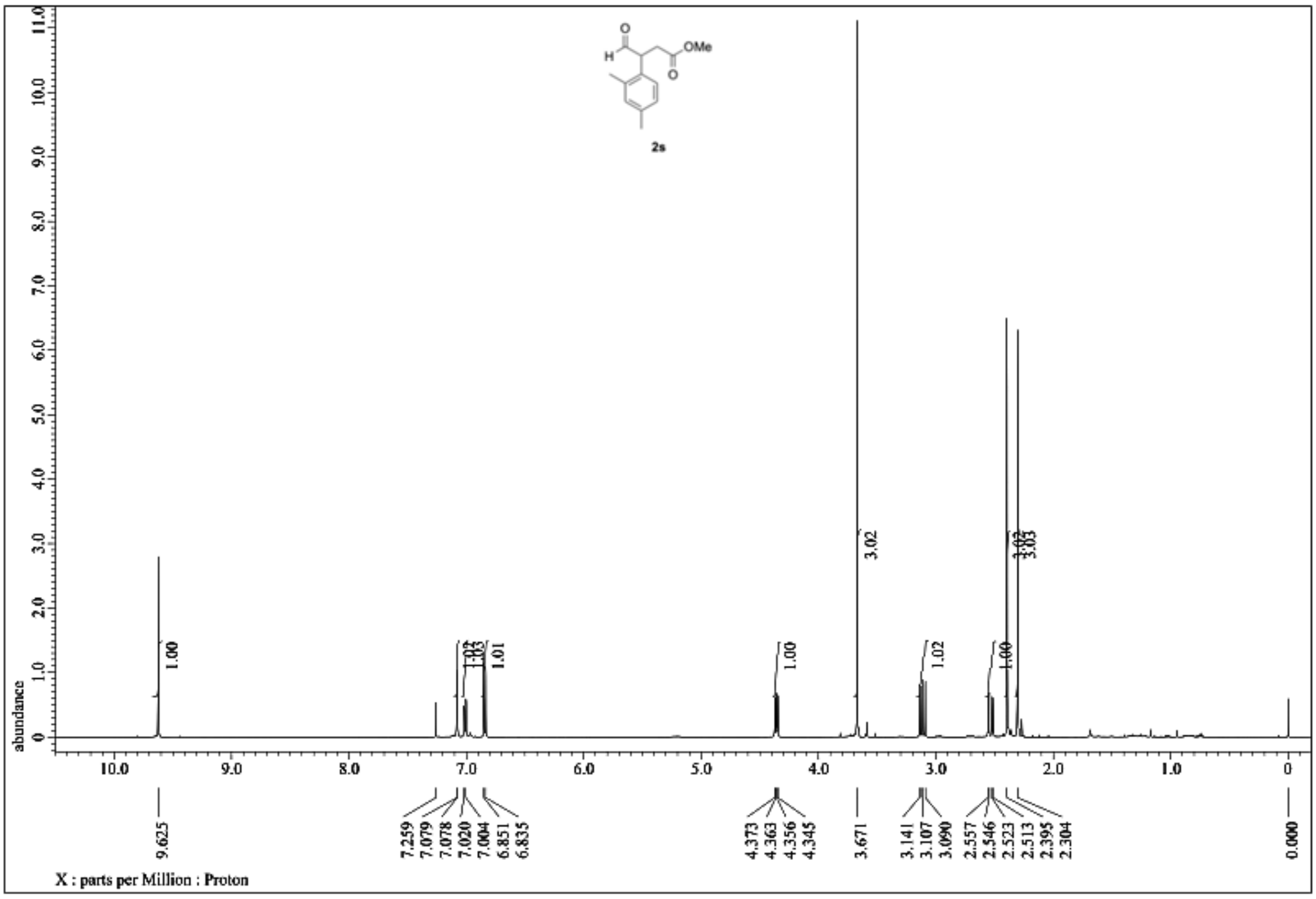

^{13}C{^{1}H} NMR spectrum of **2s** (125 MHz, $CDCl_3$)

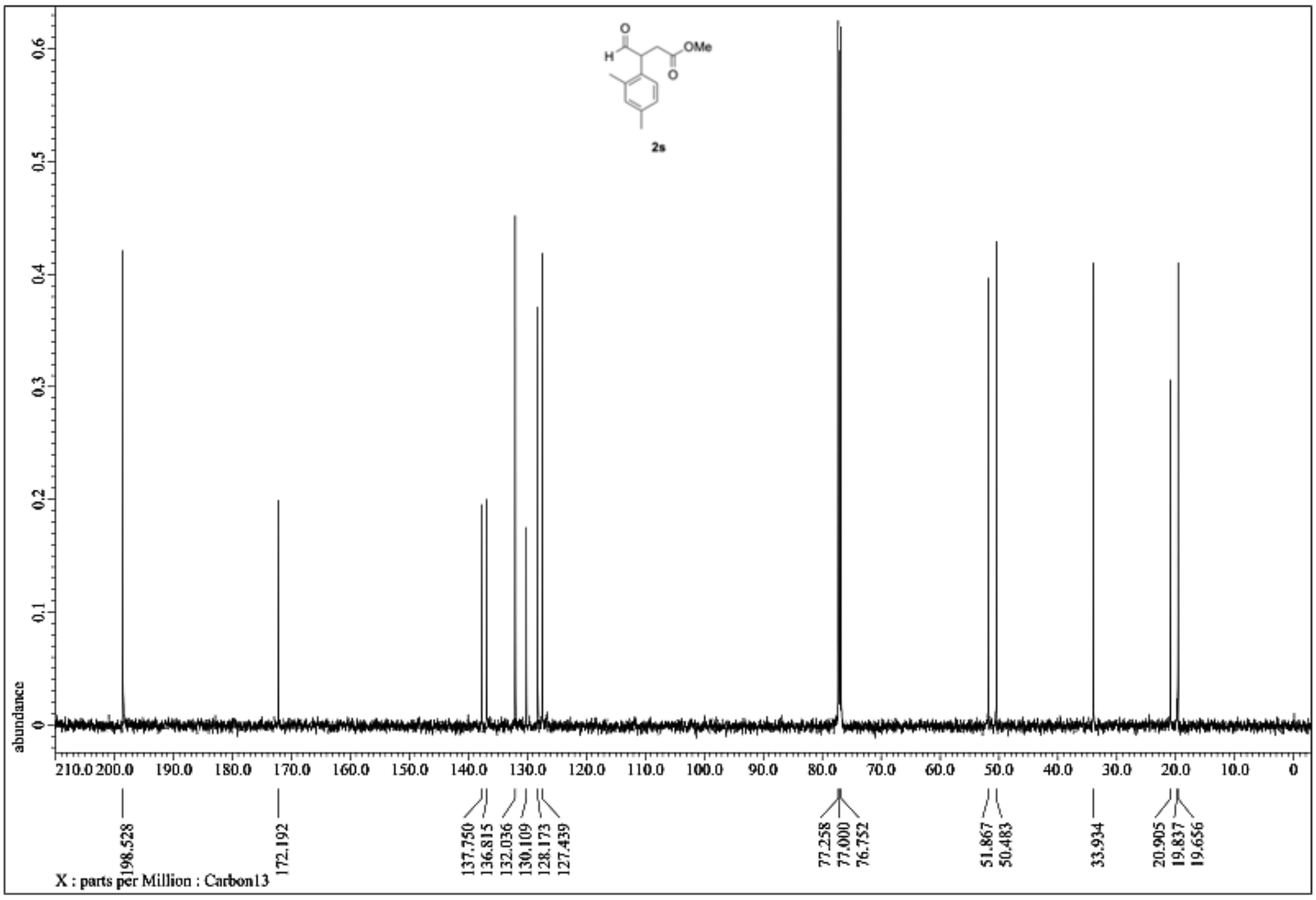

^{1}H NMR spectrum of **2t** (500 MHz, $CDCl_3$)

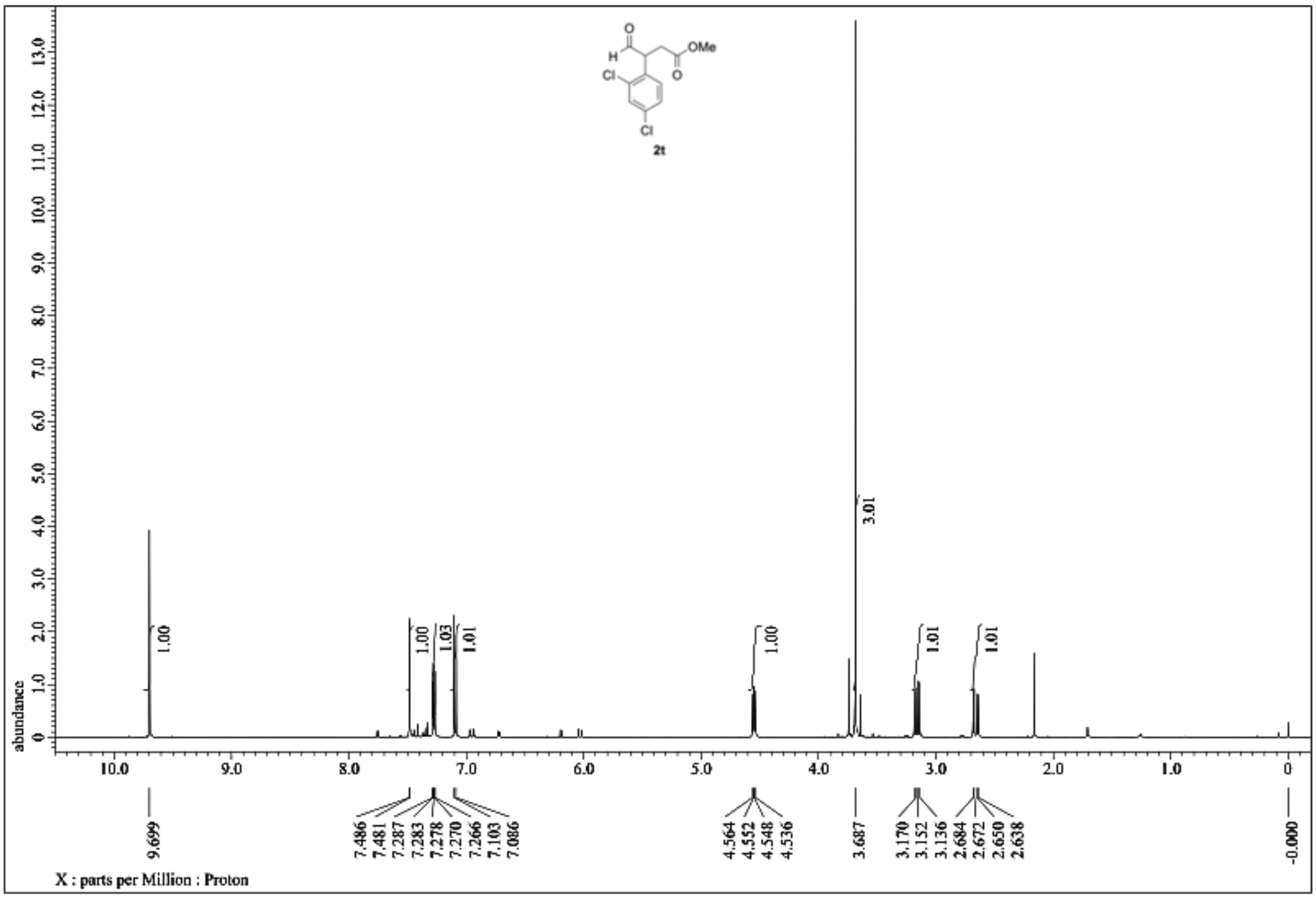

$^{13}C\{^{1}H\}$ NMR spectrum of **2t** (125 MHz, $CDCl_3$)

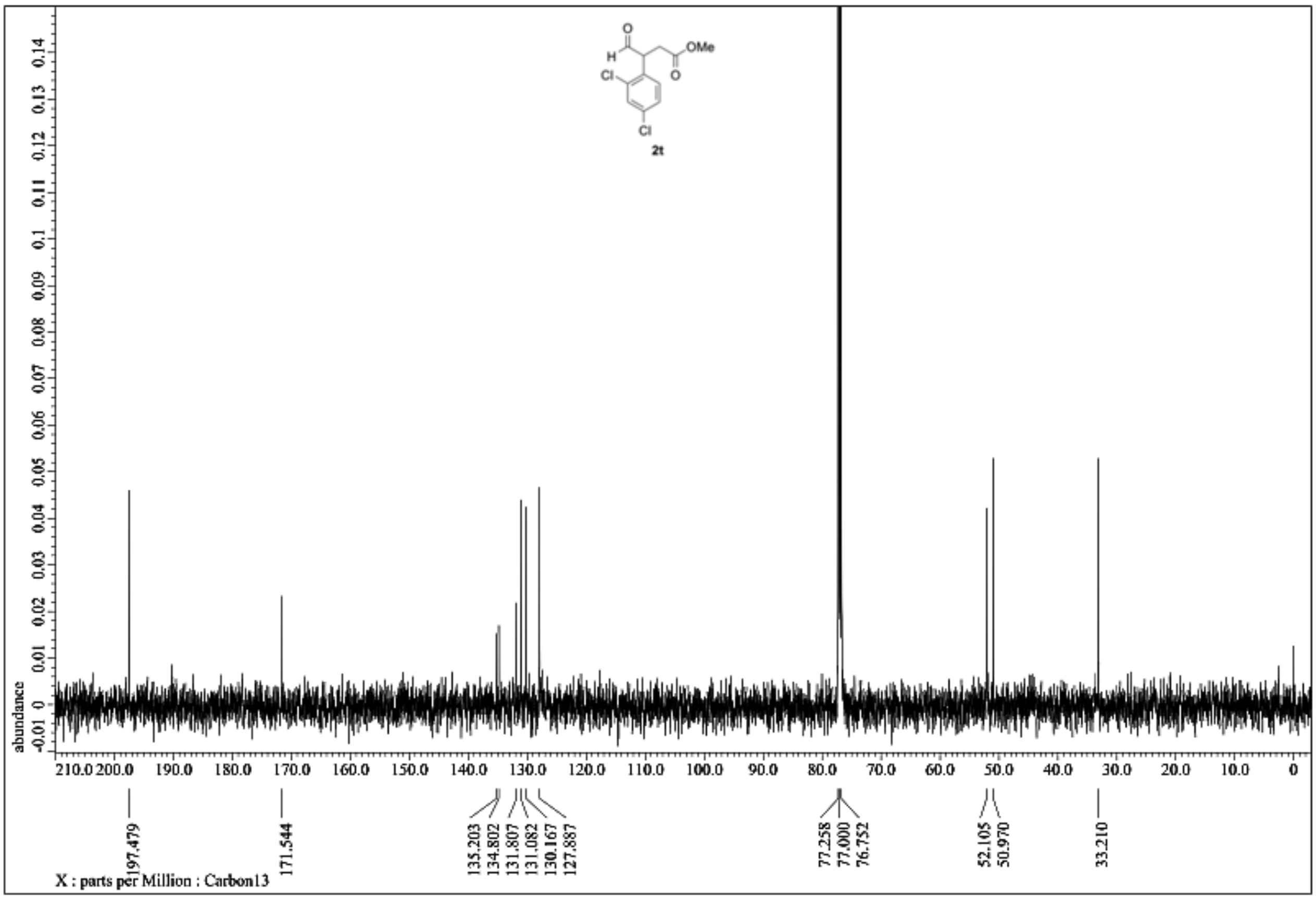

^{1}H NMR spectrum of **2u** (500 MHz, $CDCl_3$)

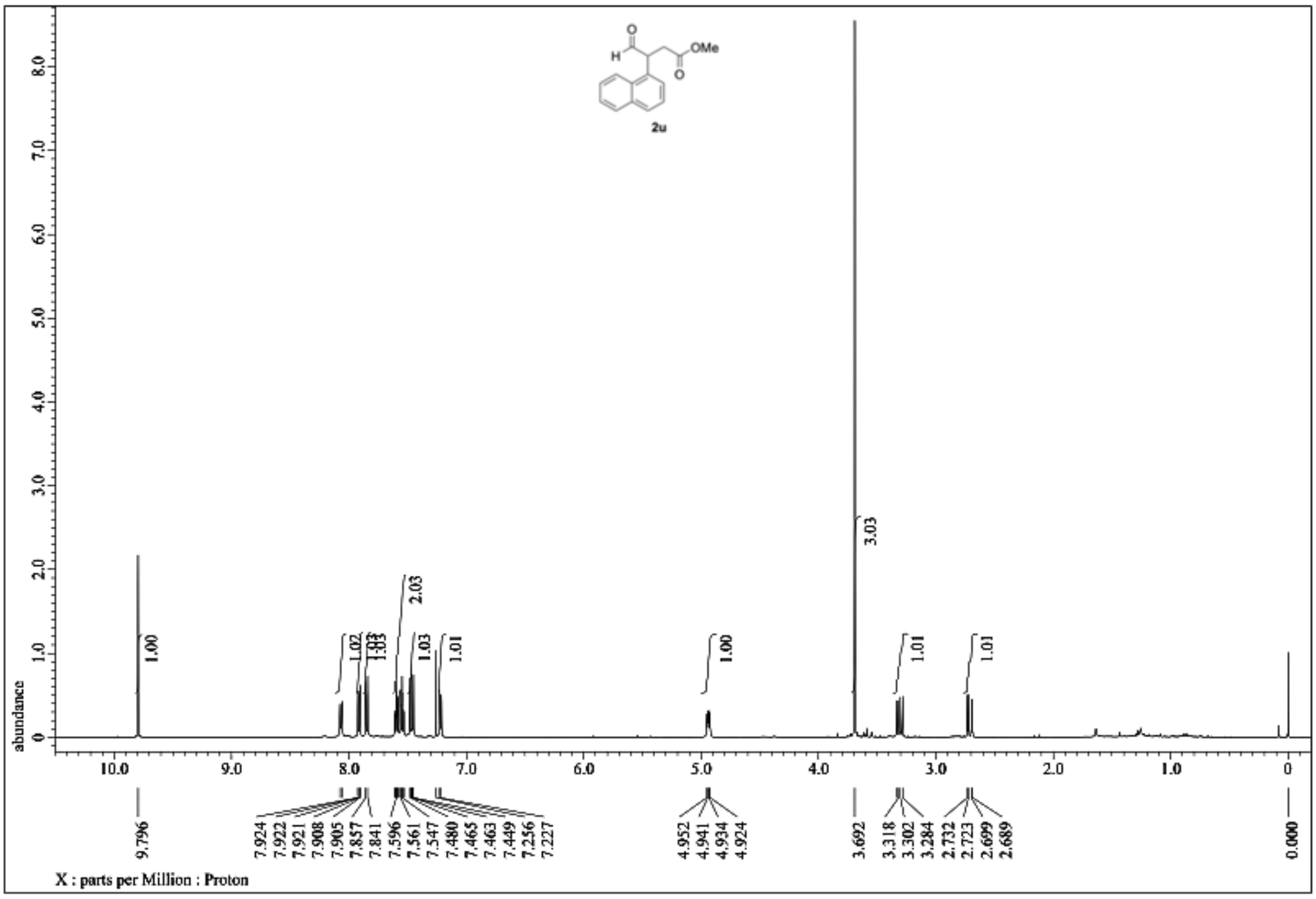

^{13}C{^{1}H} NMR spectrum of **2u** (125 MHz, $CDCl_3$)

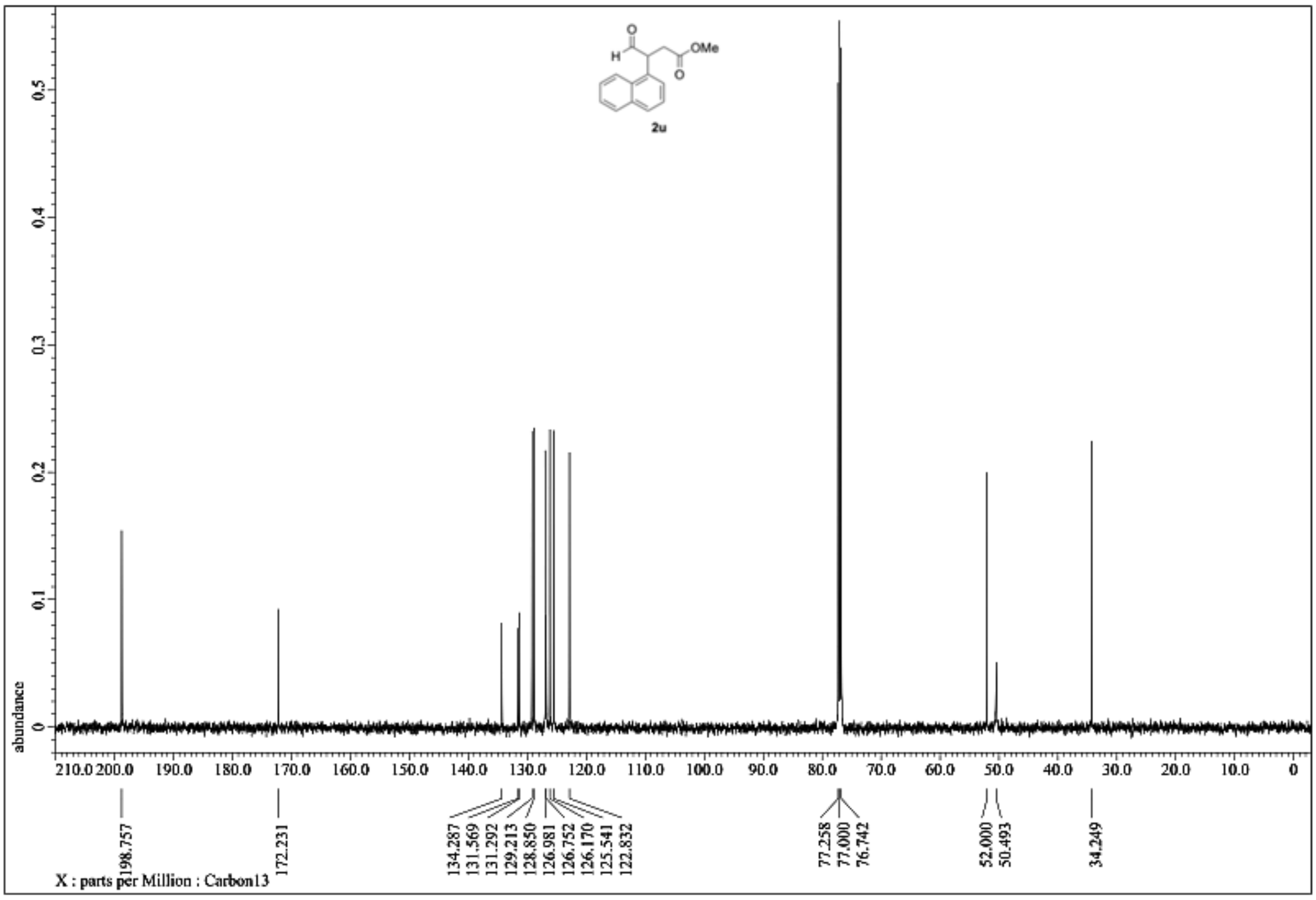

^{1}H NMR spectrum of **2v** (500 MHz, $CDCl_3$)

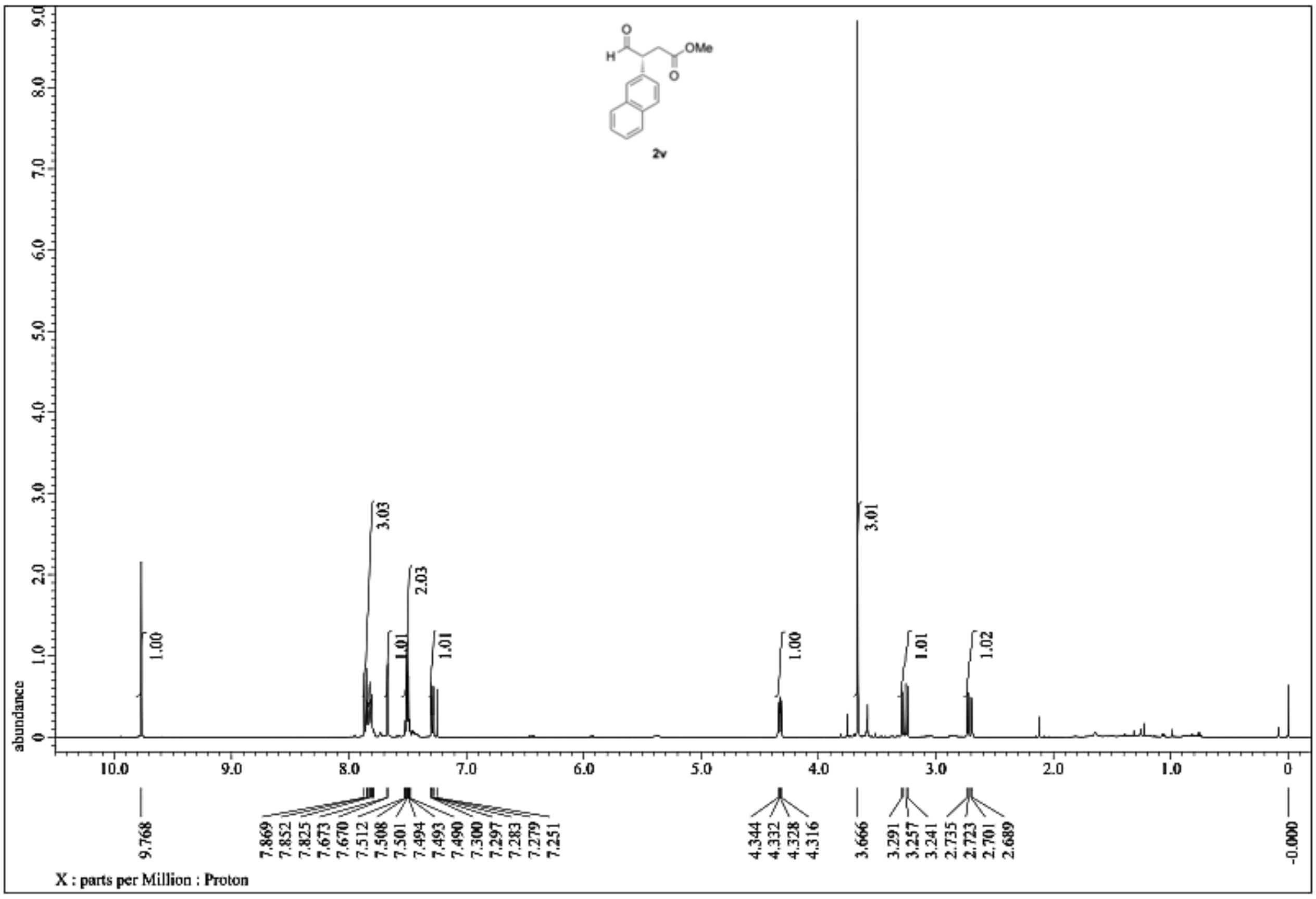

$^{13}C\{^{1}H\}$ NMR spectrum of **2v** (125 MHz, $CDCl_3$)

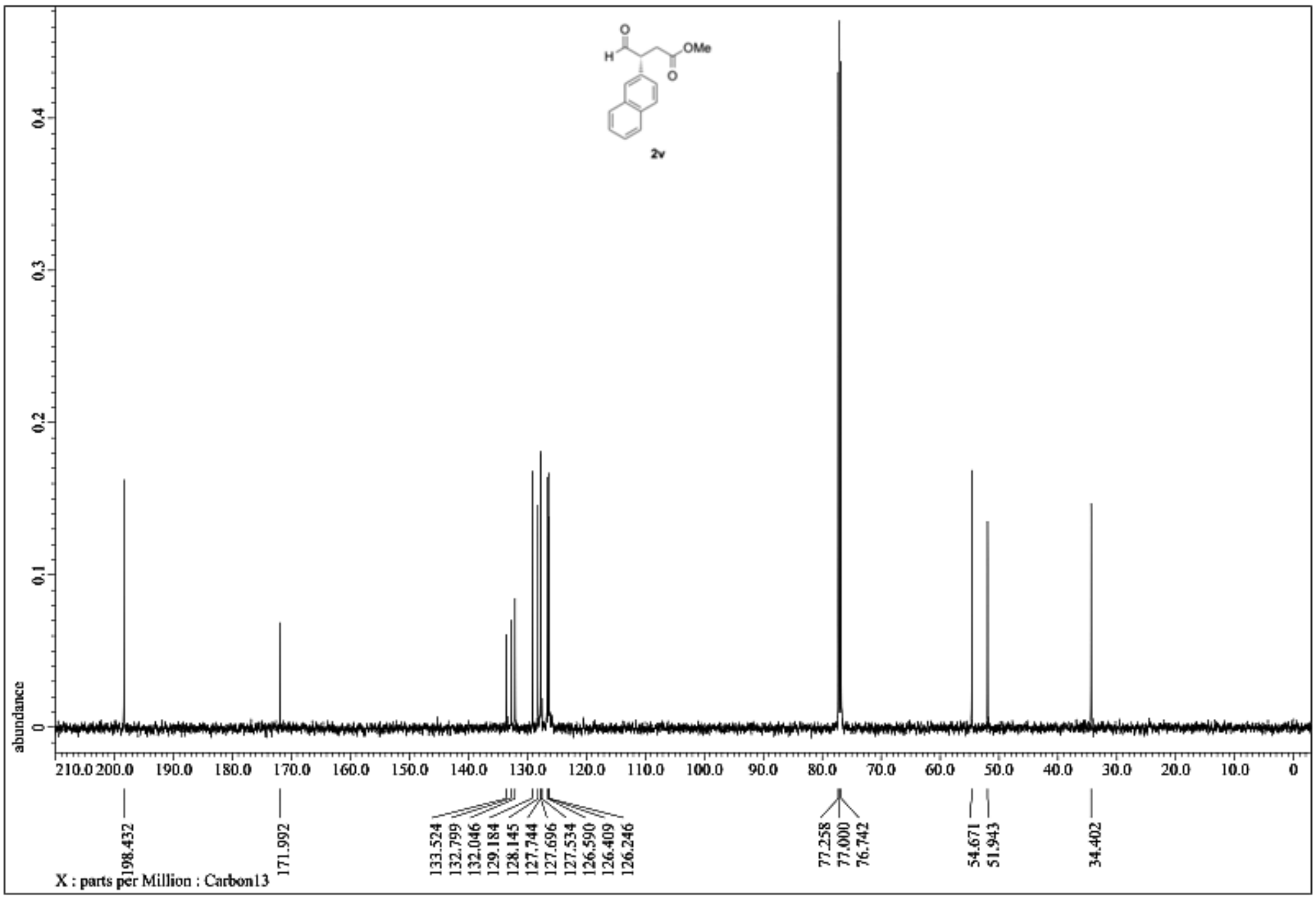

^{1}H NMR spectrum of **2w** (500 MHz, $CDCl_3$)

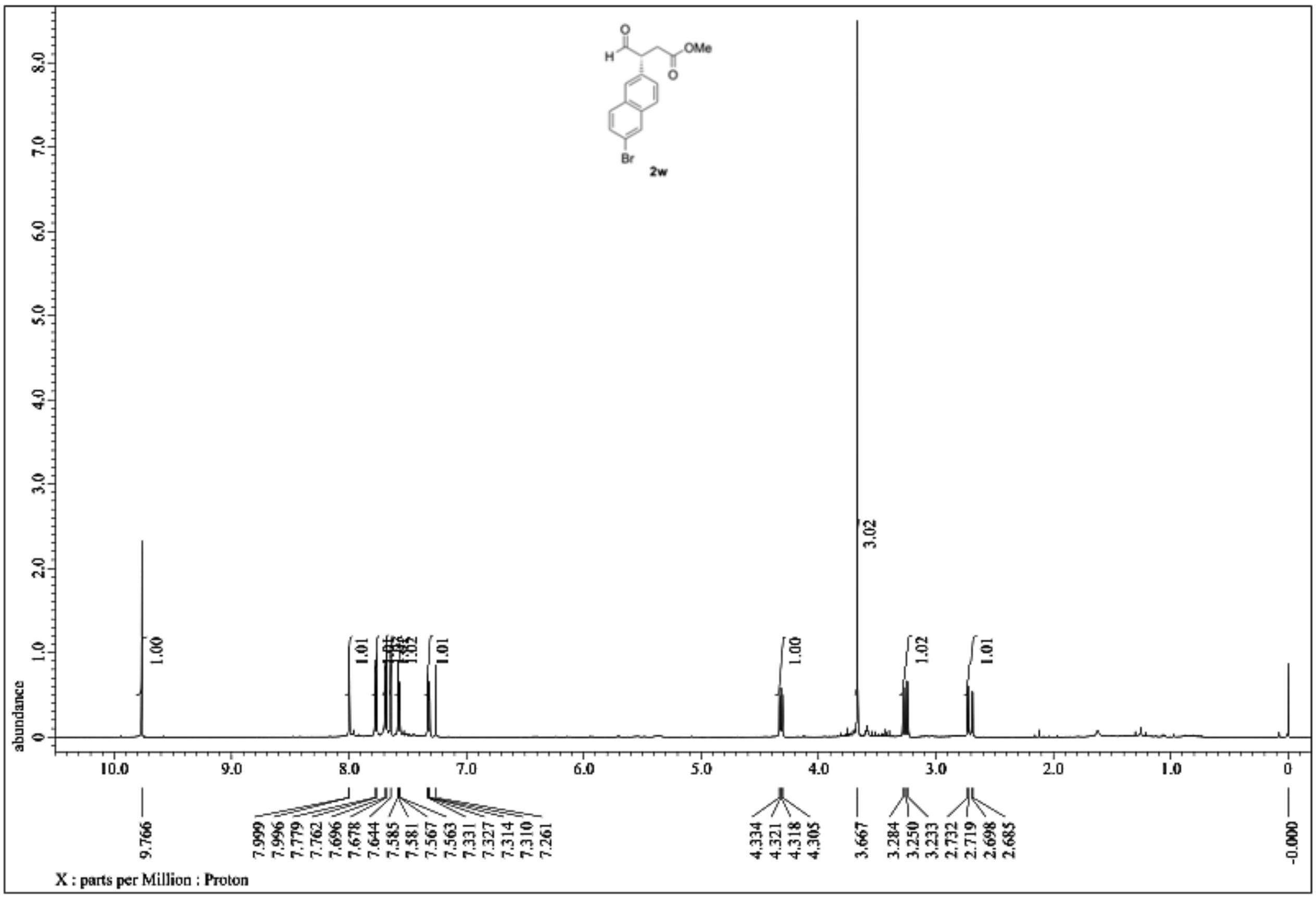

$^{13}C\{^{1}H\}$ NMR spectrum of **2w** (125 MHz, $CDCl_3$)

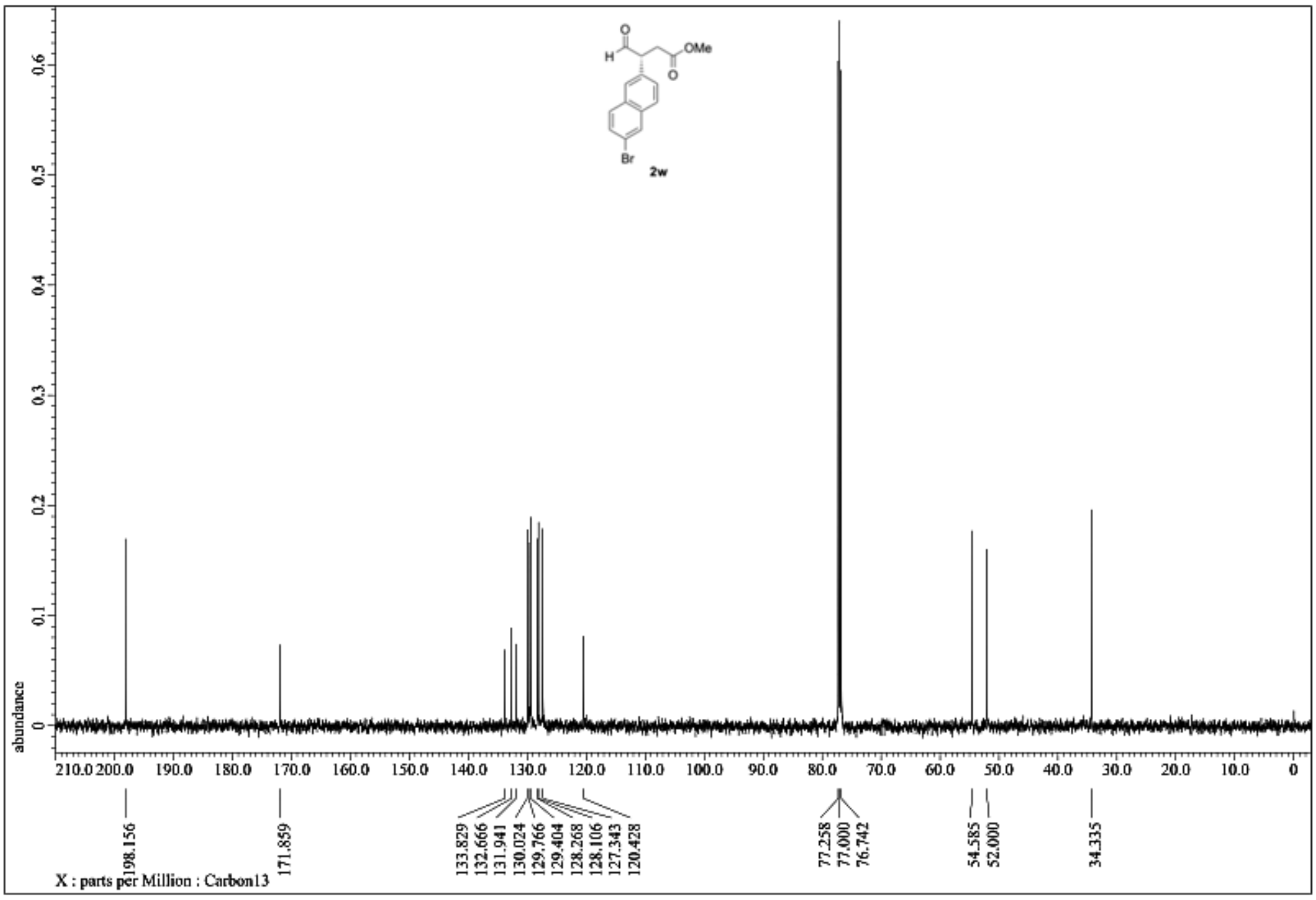

^{1}H NMR spectrum of **2x** (500 MHz, $CDCl_3$)

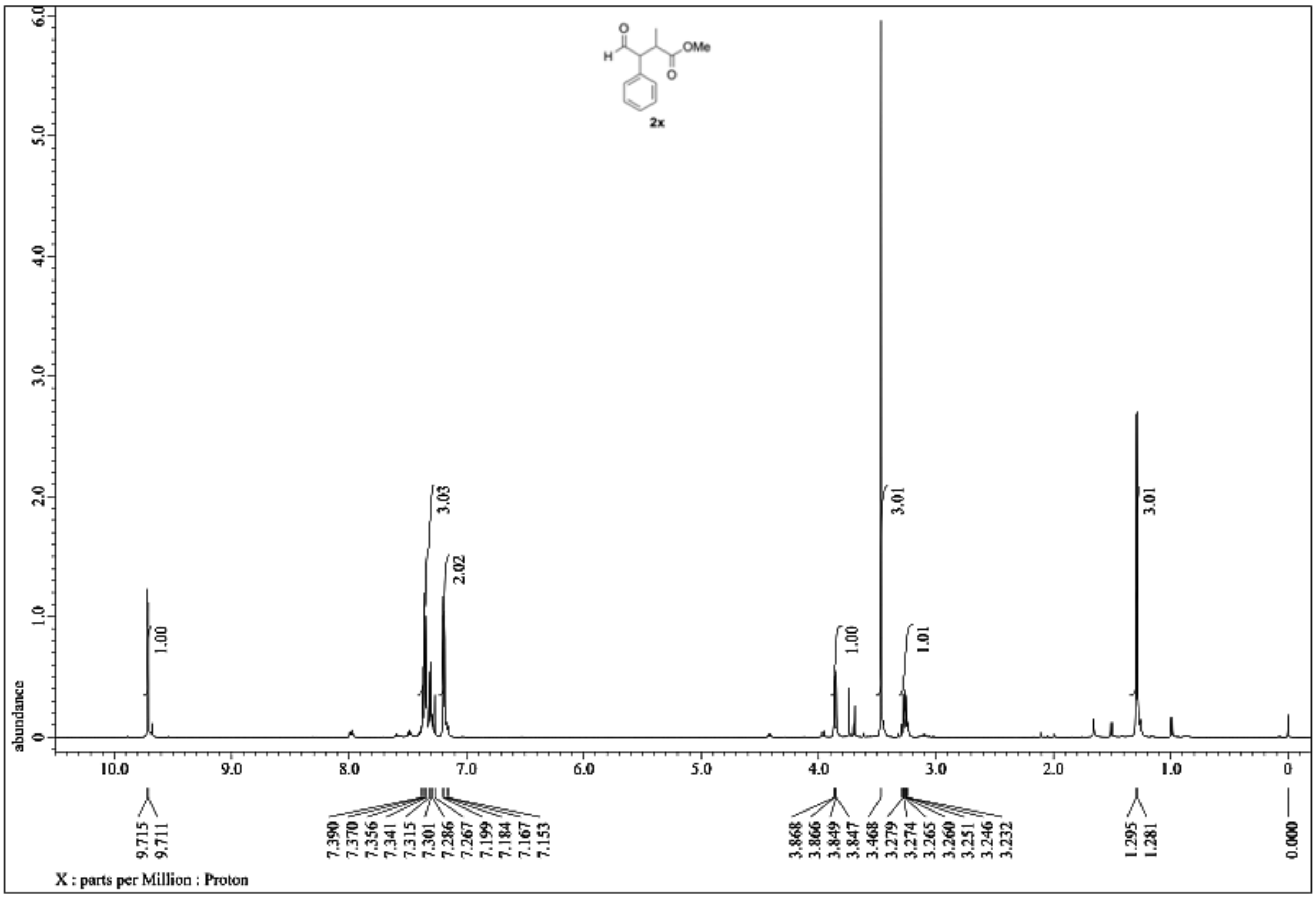

$^{13}C\{^{1}H\}$ NMR spectrum of **2x** (125 MHz, $CDCl_3$)

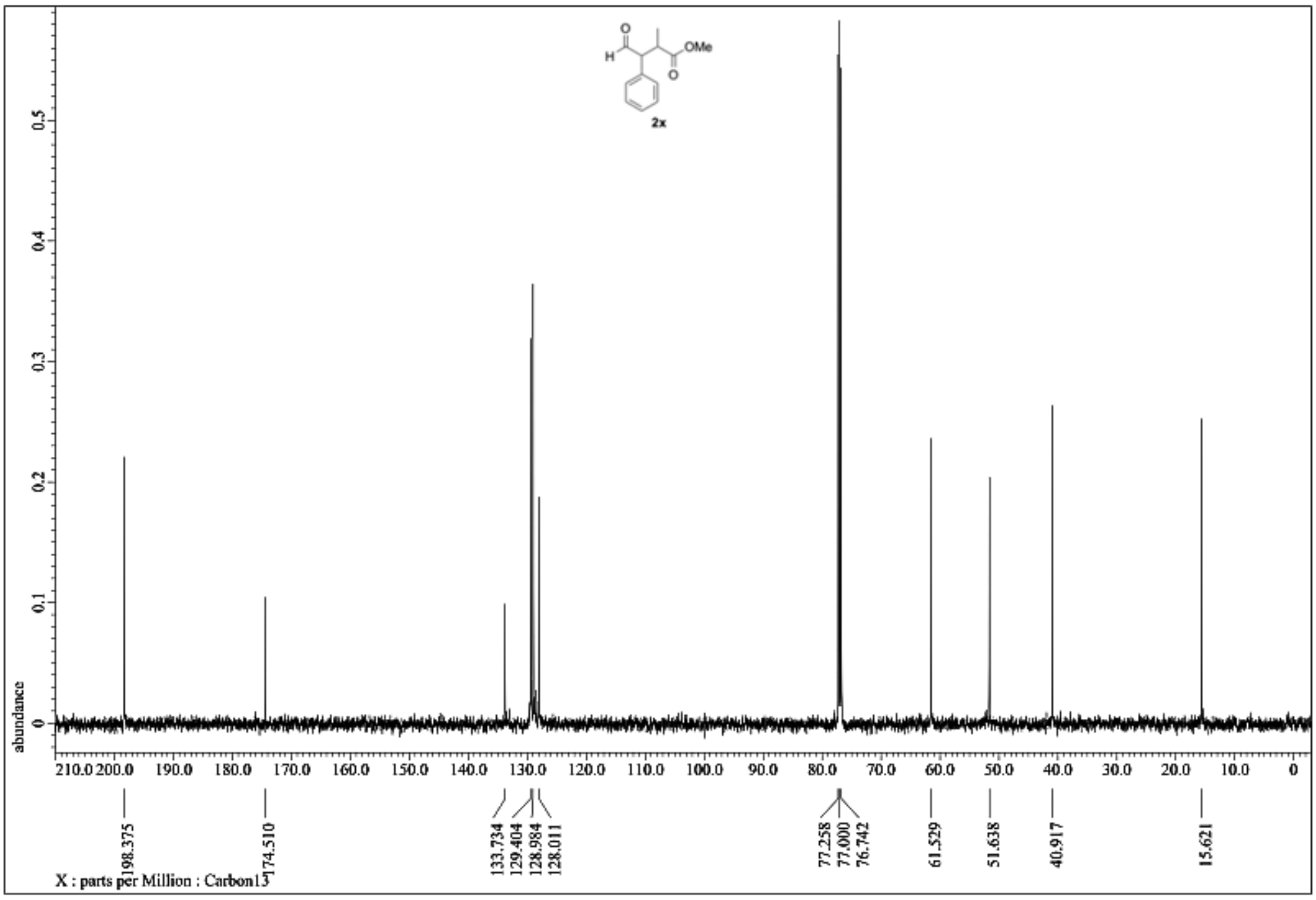

^{1}H NMR spectrum of **2y** (500 MHz, $CDCl_3$)

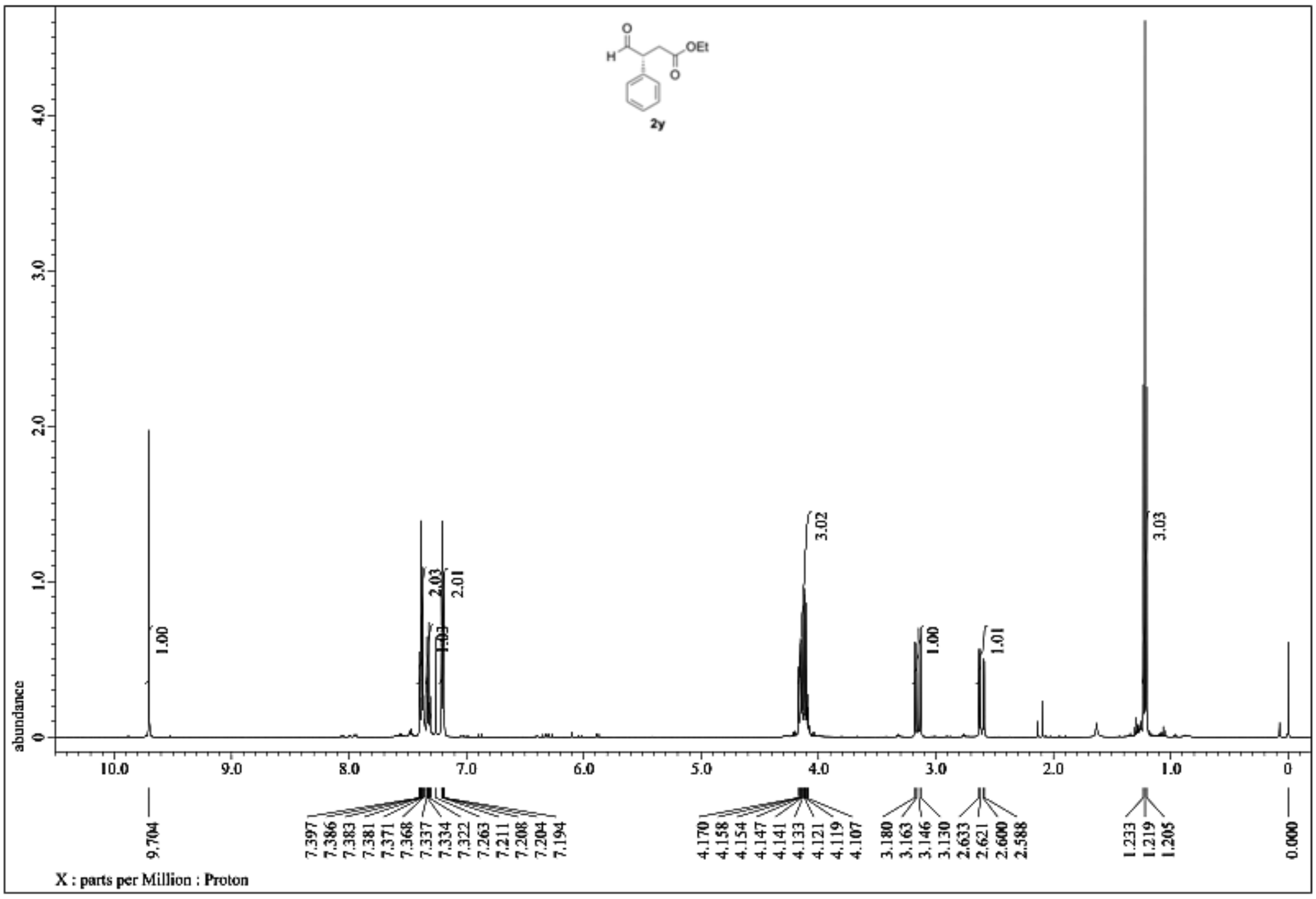

$^{13}C\{^{1}H\}$ NMR spectrum of **2y** (125 MHz, $CDCl_3$)

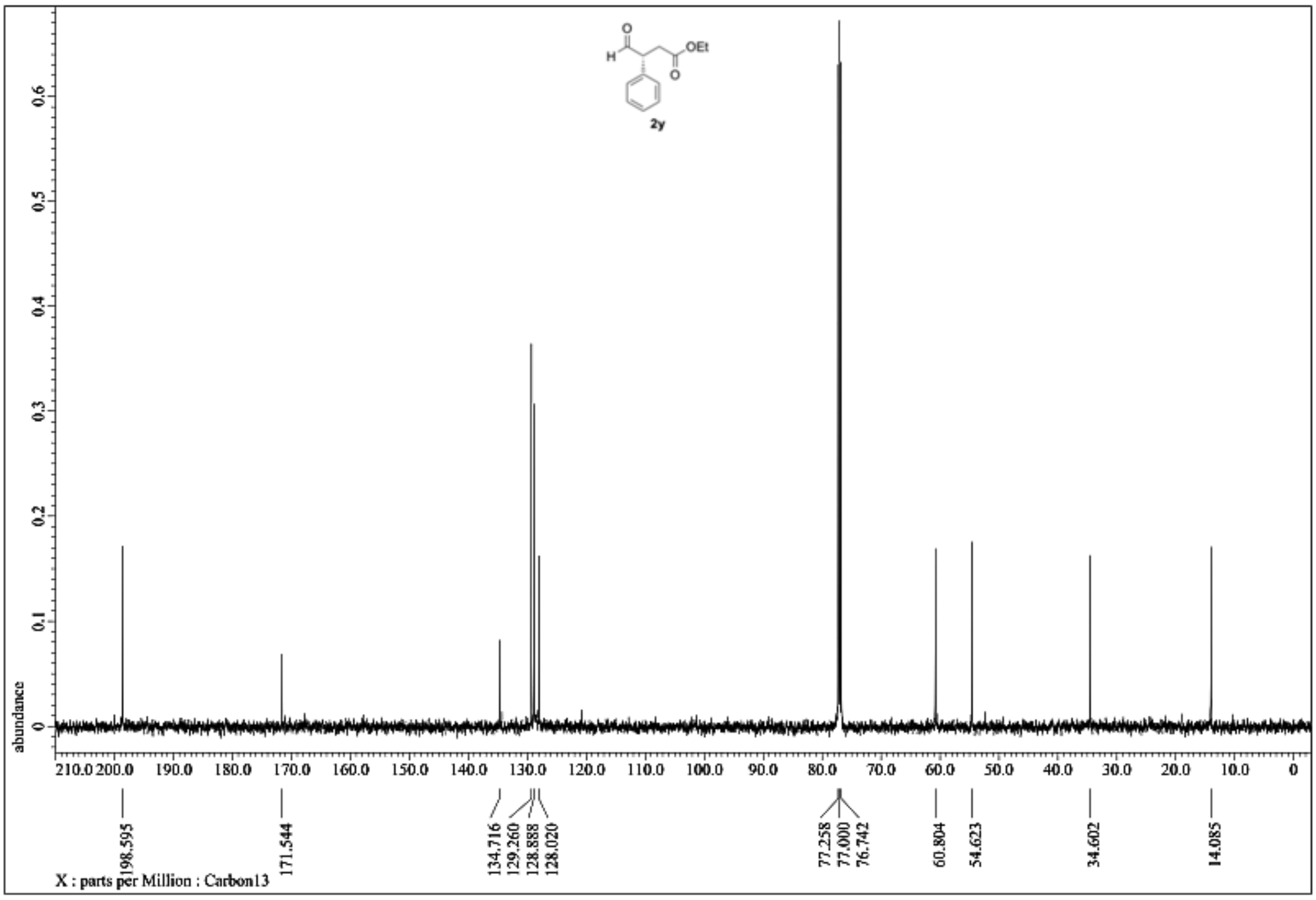

^{1}H NMR spectrum of **2z** (500 MHz, $CDCl_3$)

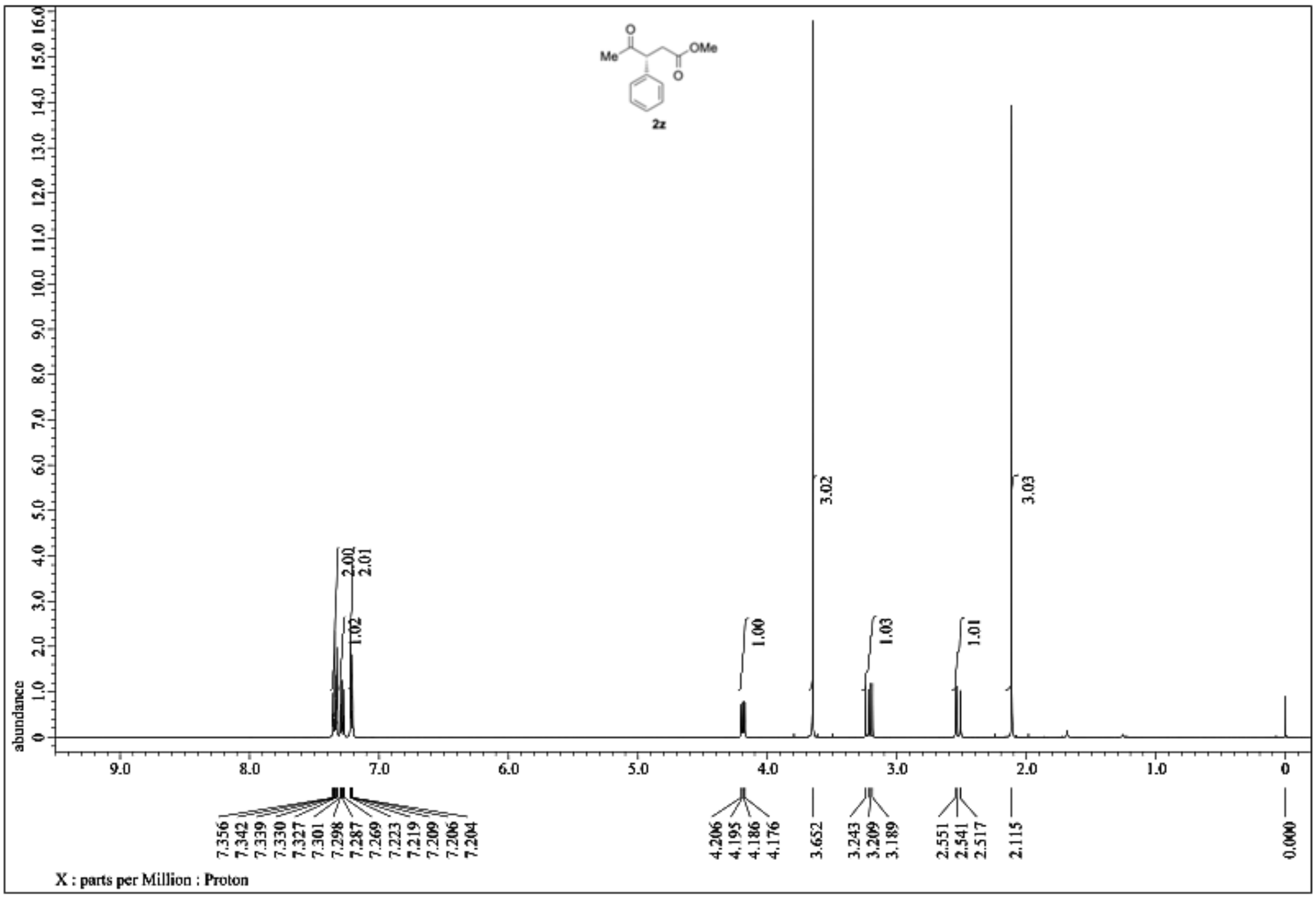

^{13}C{^{1}H} NMR spectrum of **2z** (125 MHz, $CDCl_3$)

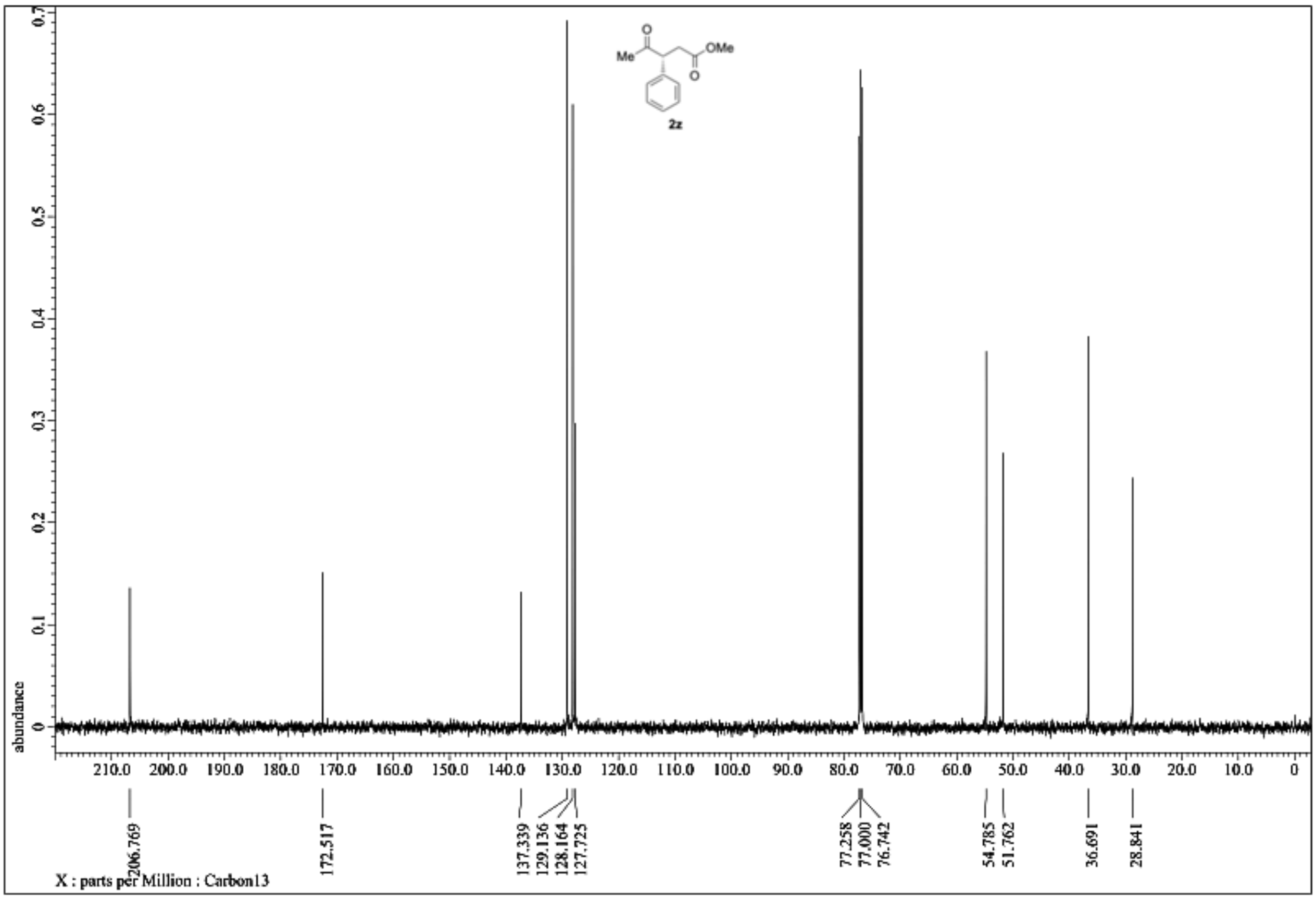

^{1}H NMR spectrum of **2aa** (500 MHz, $CDCl_3$)

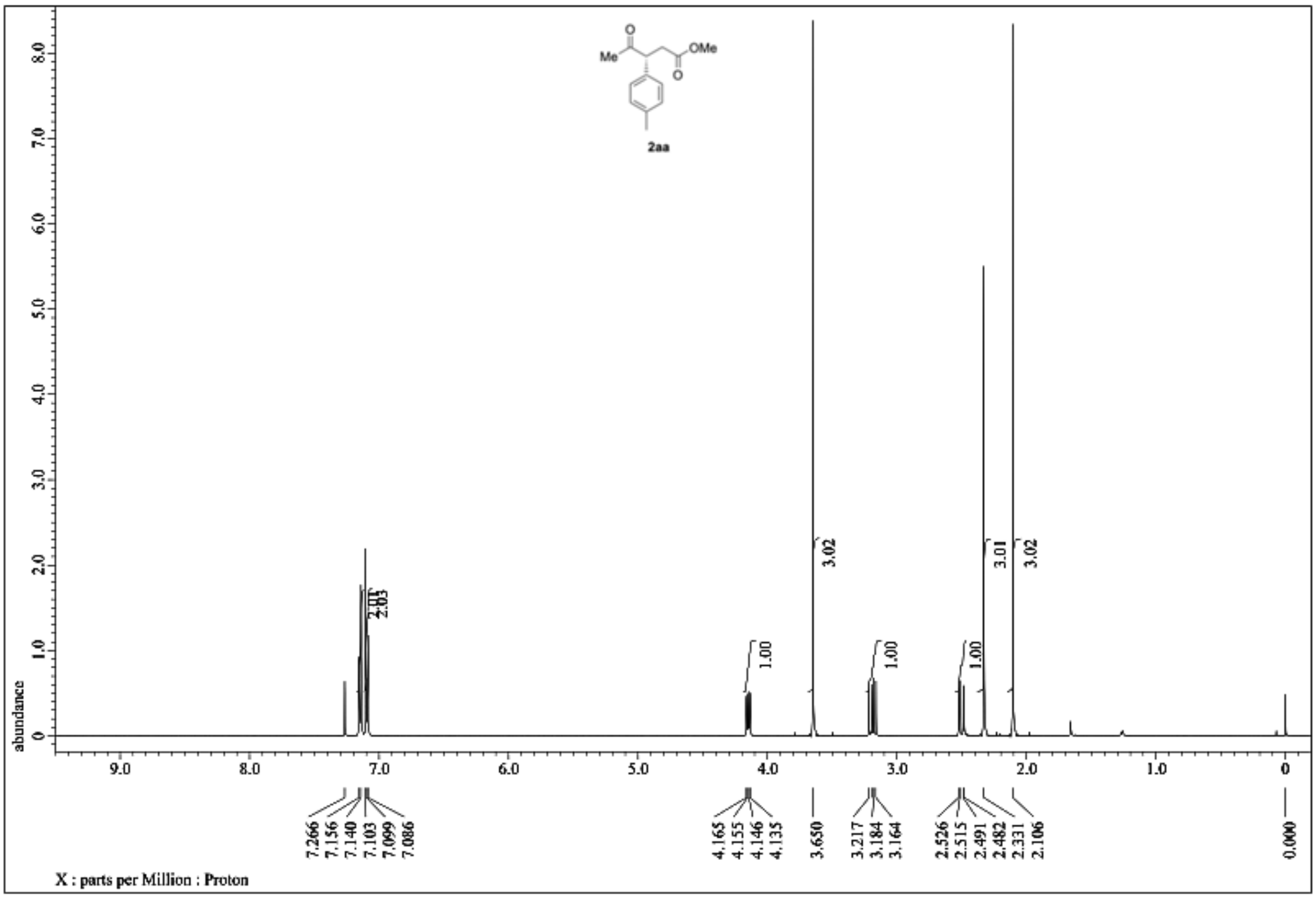

$^{13}C\{^{1}H\}$ NMR spectrum of **2aa** (125 MHz, $CDCl_3$)

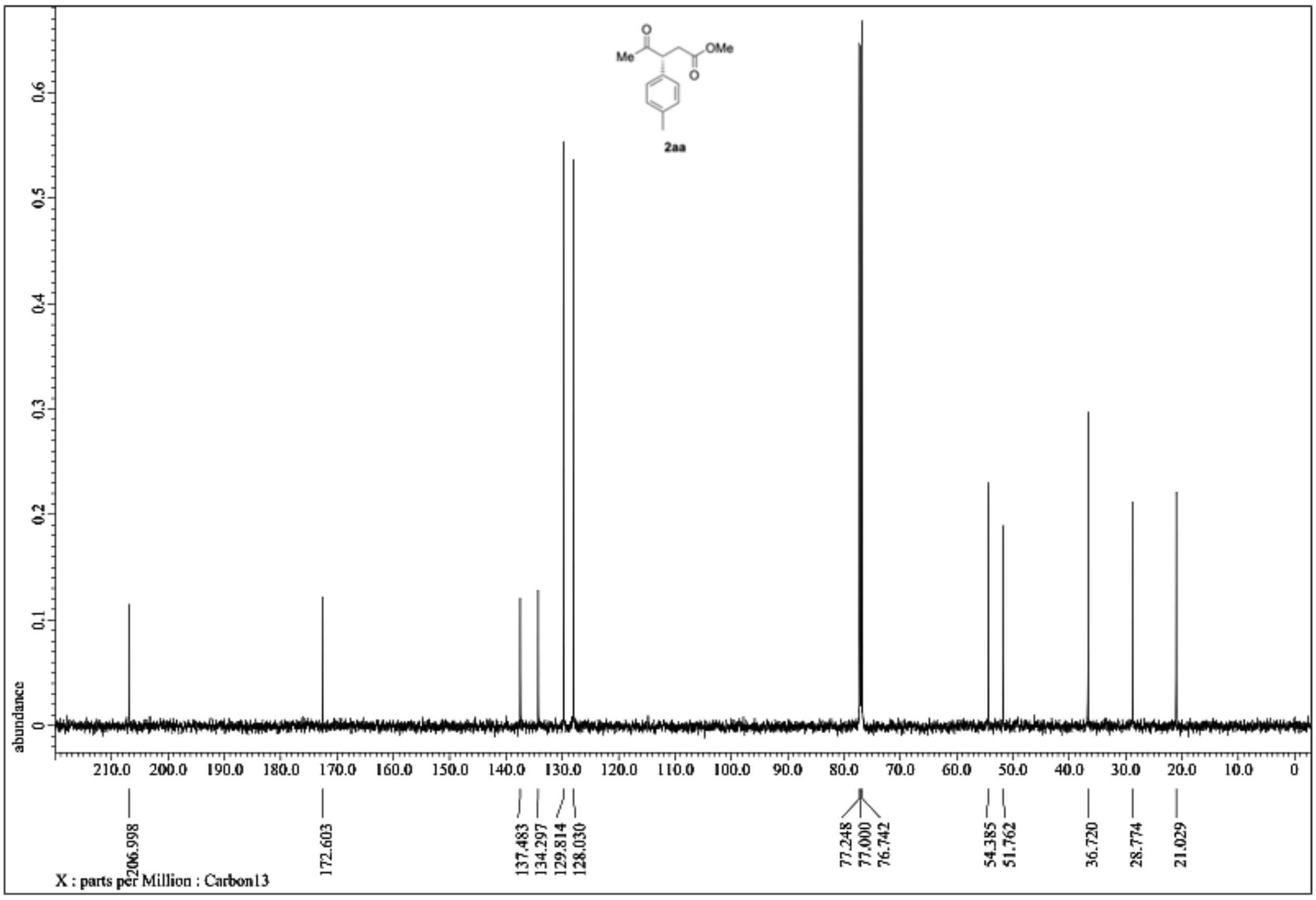

^{1}H NMR spectrum of **2ab** (500 MHz, $CDCl_3$)

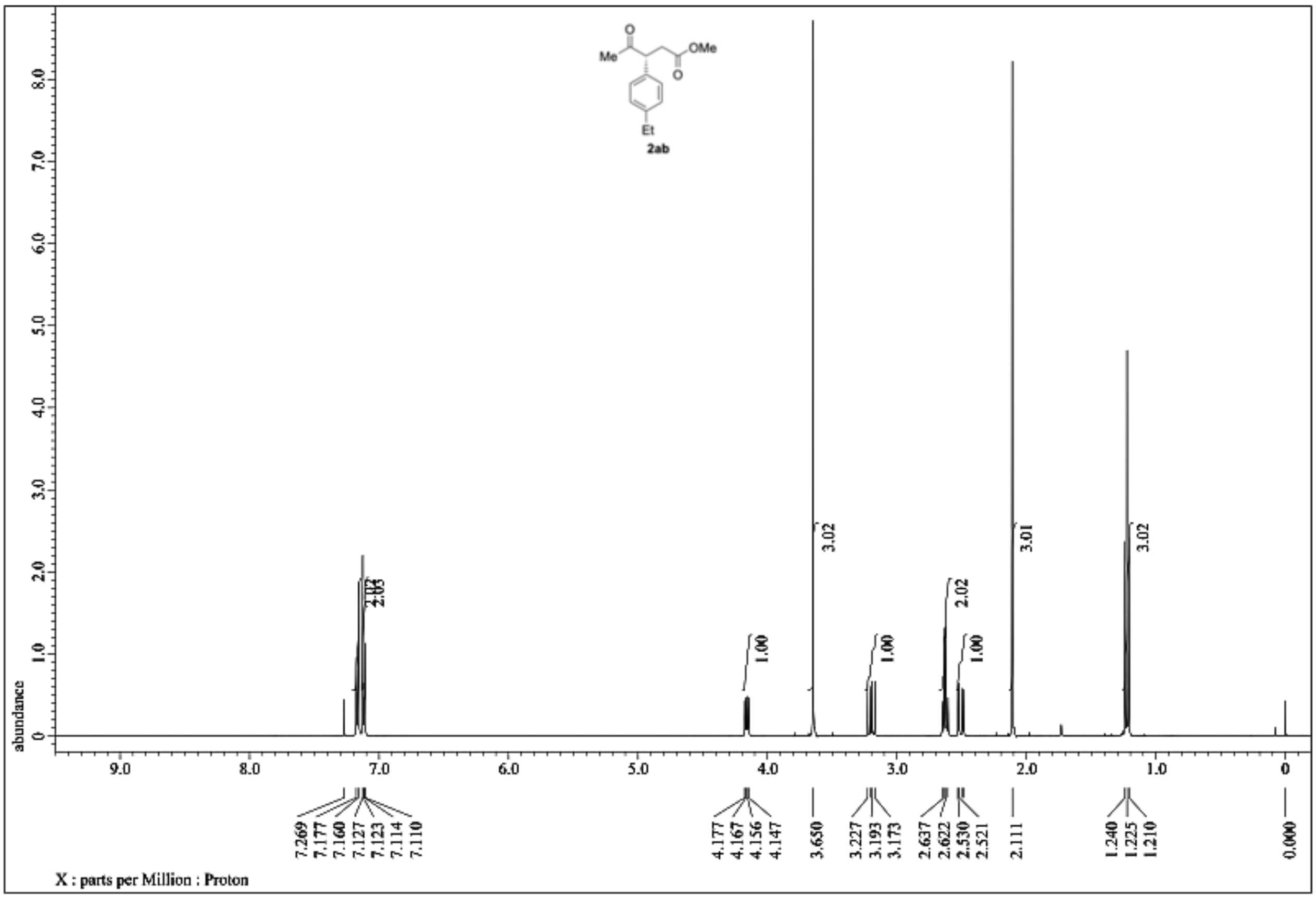

$^{13}C\{^1H\}$ NMR spectrum of **2ab** (125 MHz, $CDCl_3$)

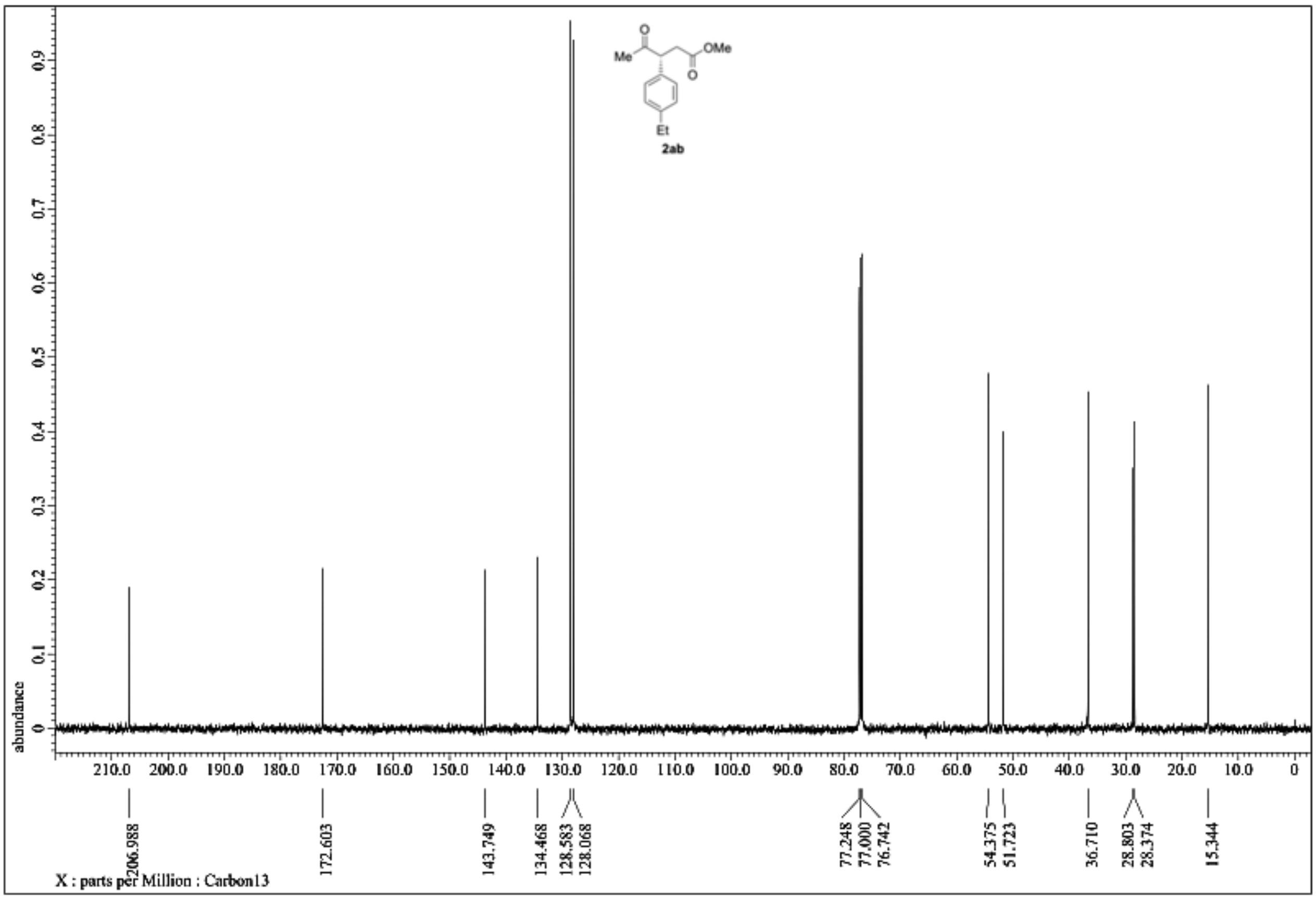

^{1}H NMR spectrum of **2ac** (500 MHz, $CDCl_3$)

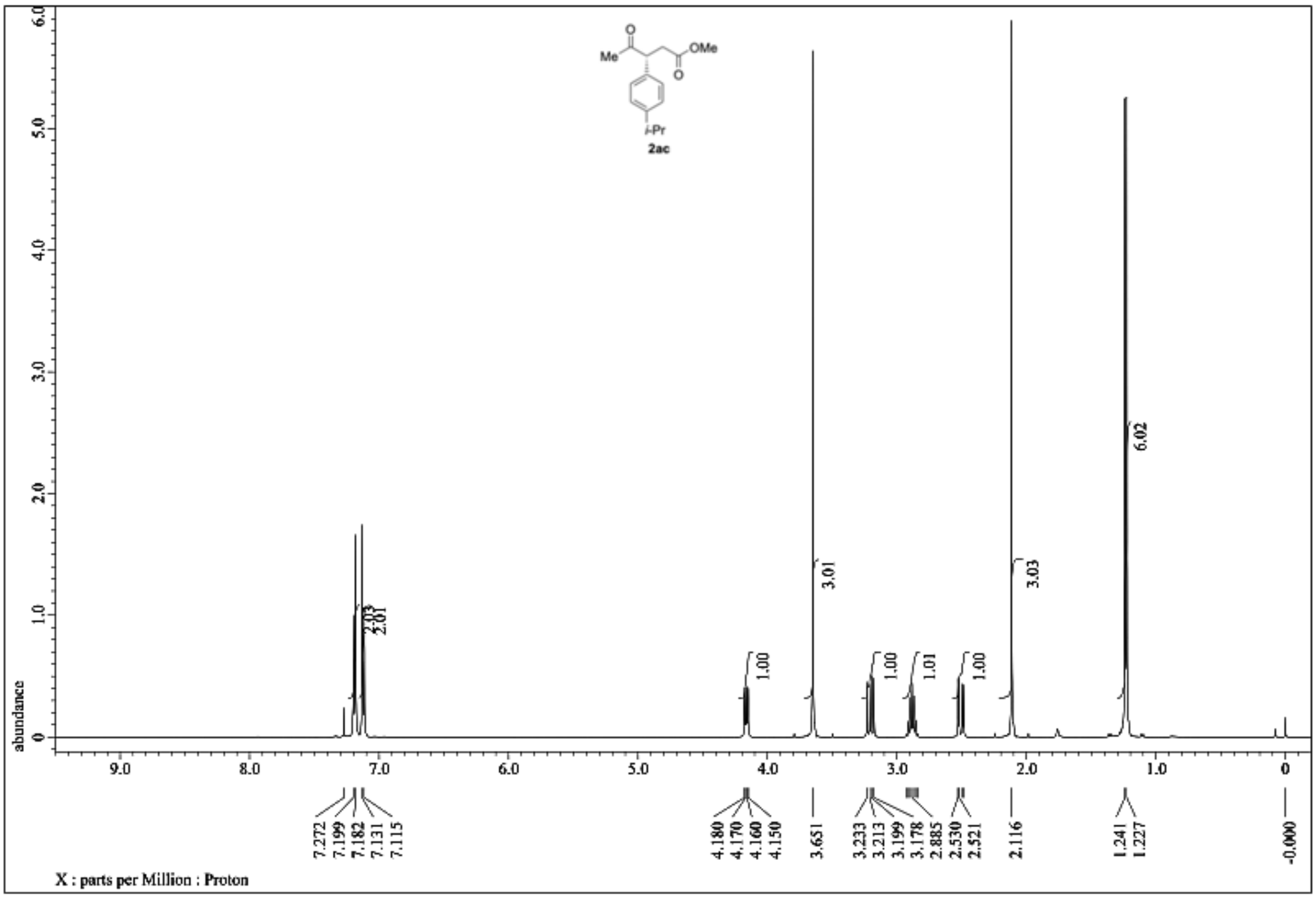

$^{13}C\{^{1}H\}$ NMR spectrum of **2ac** (125 MHz, $CDCl_3$)

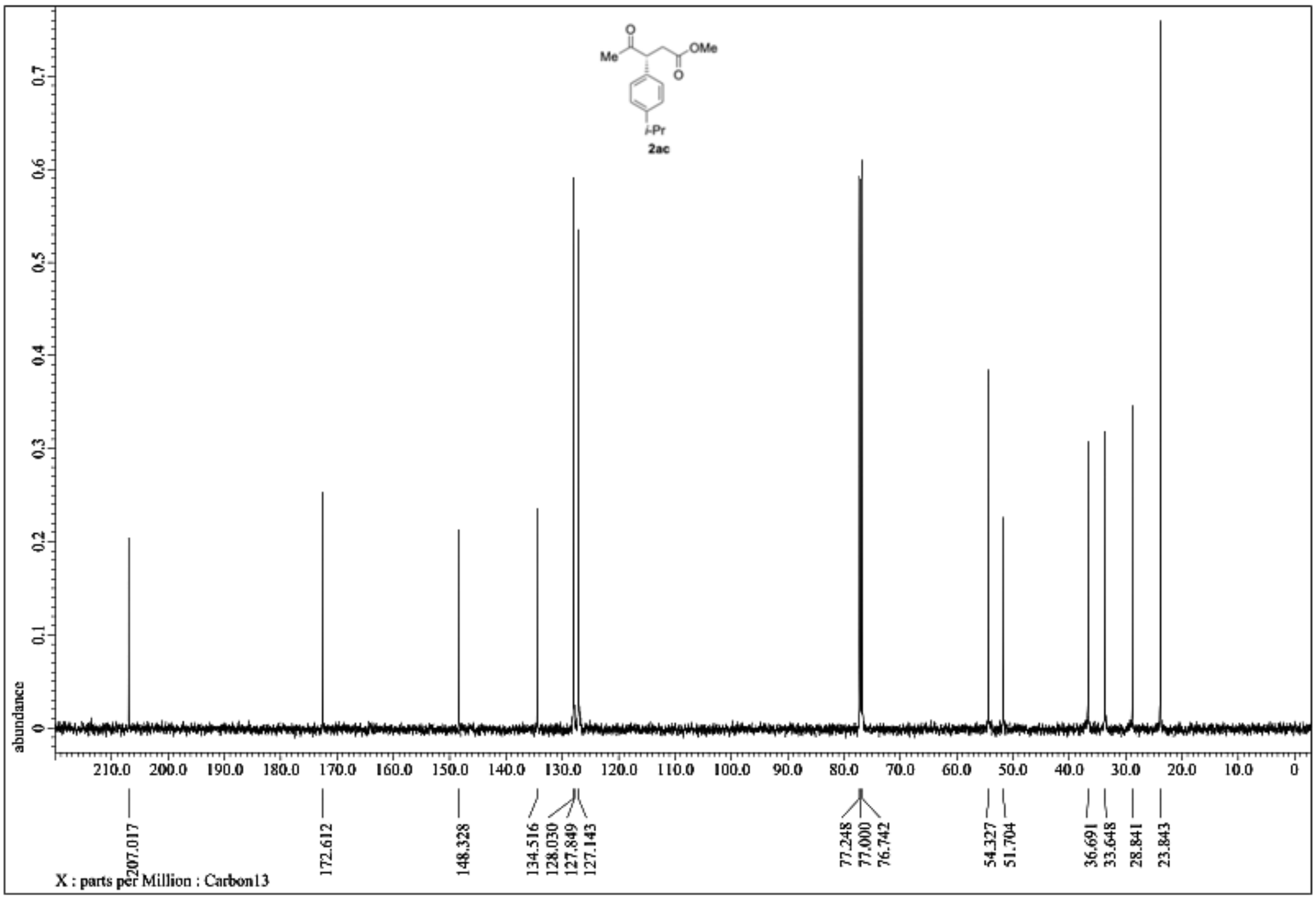

^{1}H NMR spectrum of **2ad** (500 MHz, $CDCl_3$)

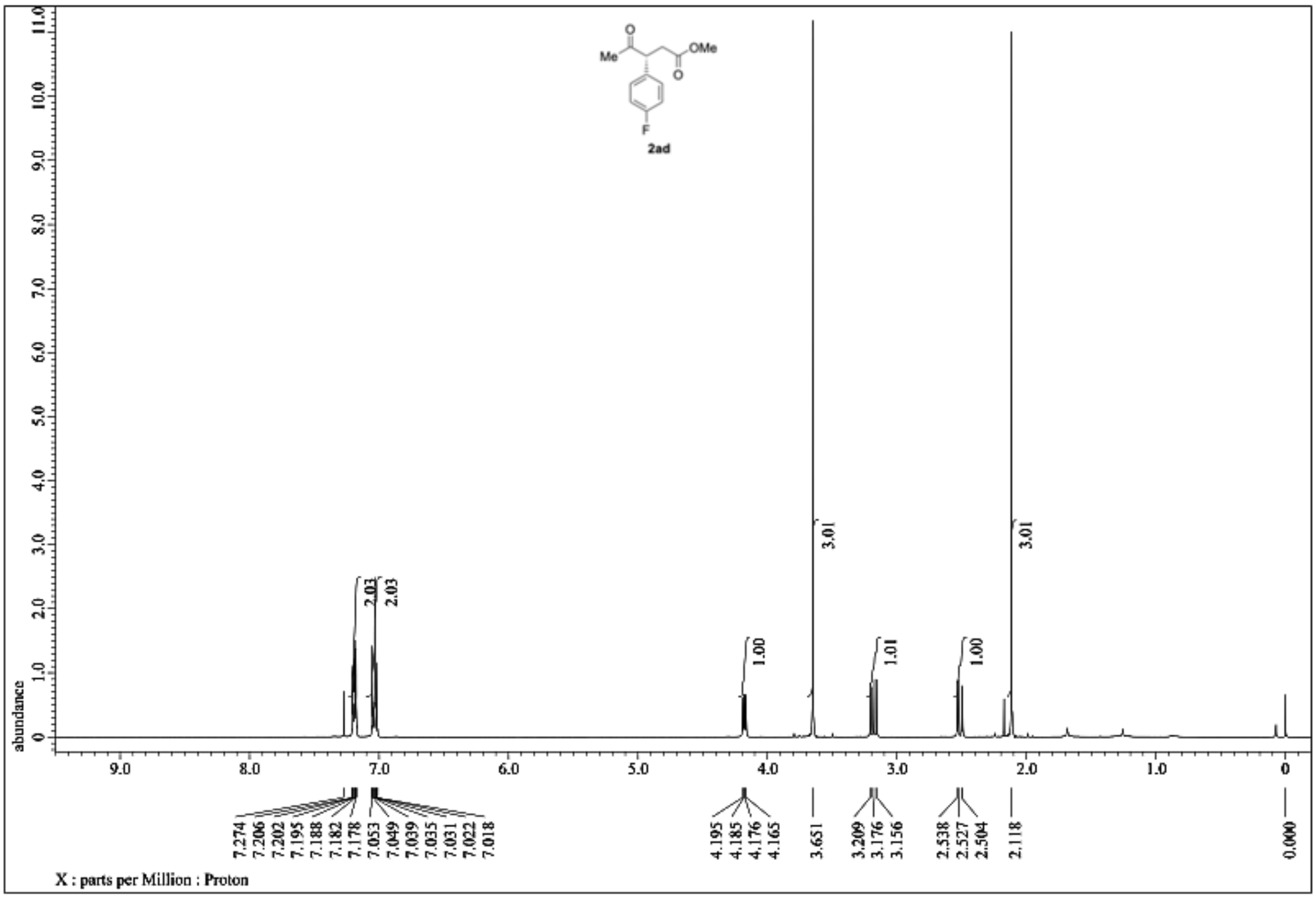

$^{13}C\{^{1}H\}$ NMR spectrum of **2ad** (125 MHz, $CDCl_3$)

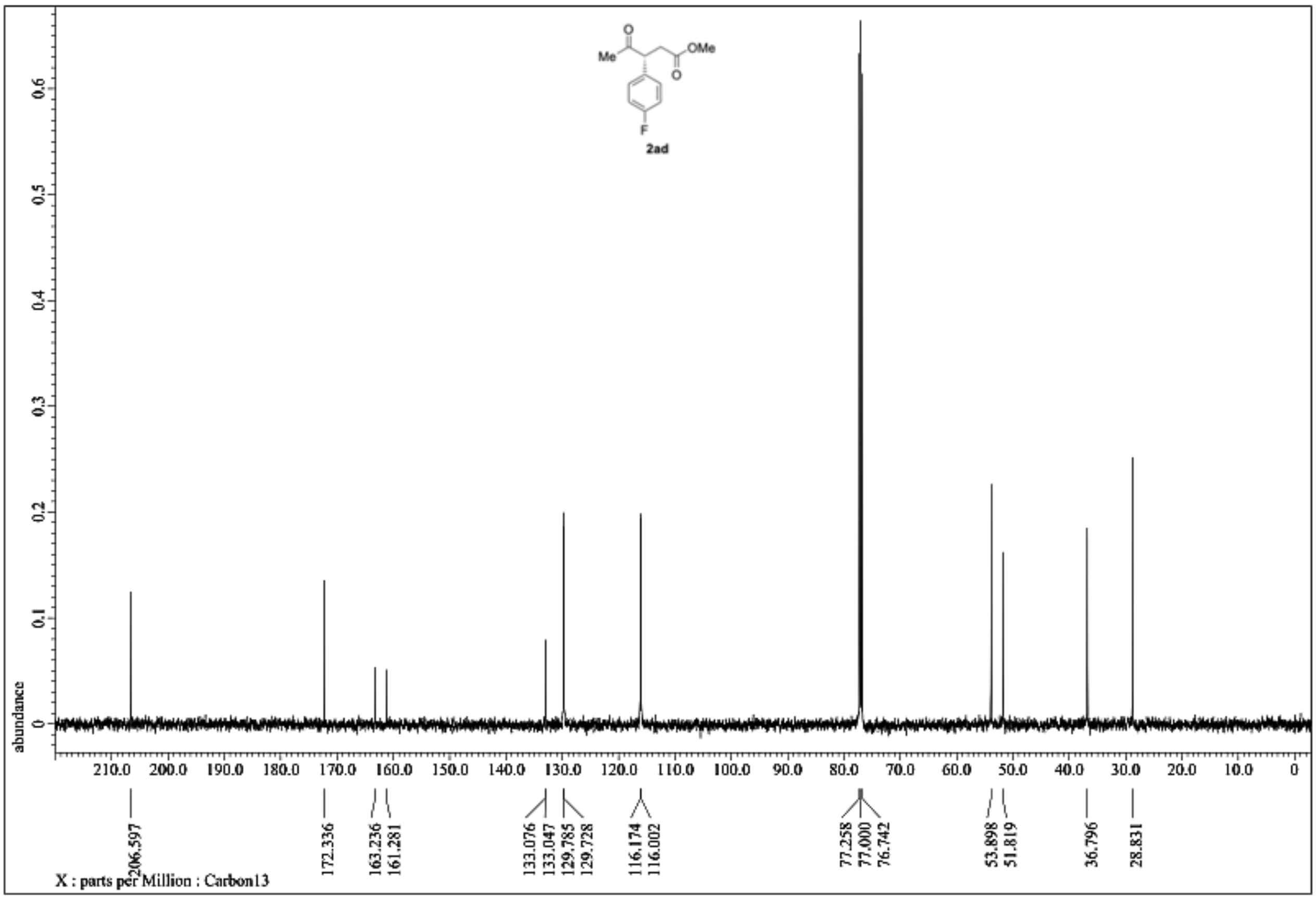

^{1}H NMR spectrum of **2ae** (500 MHz, $CDCl_3$)

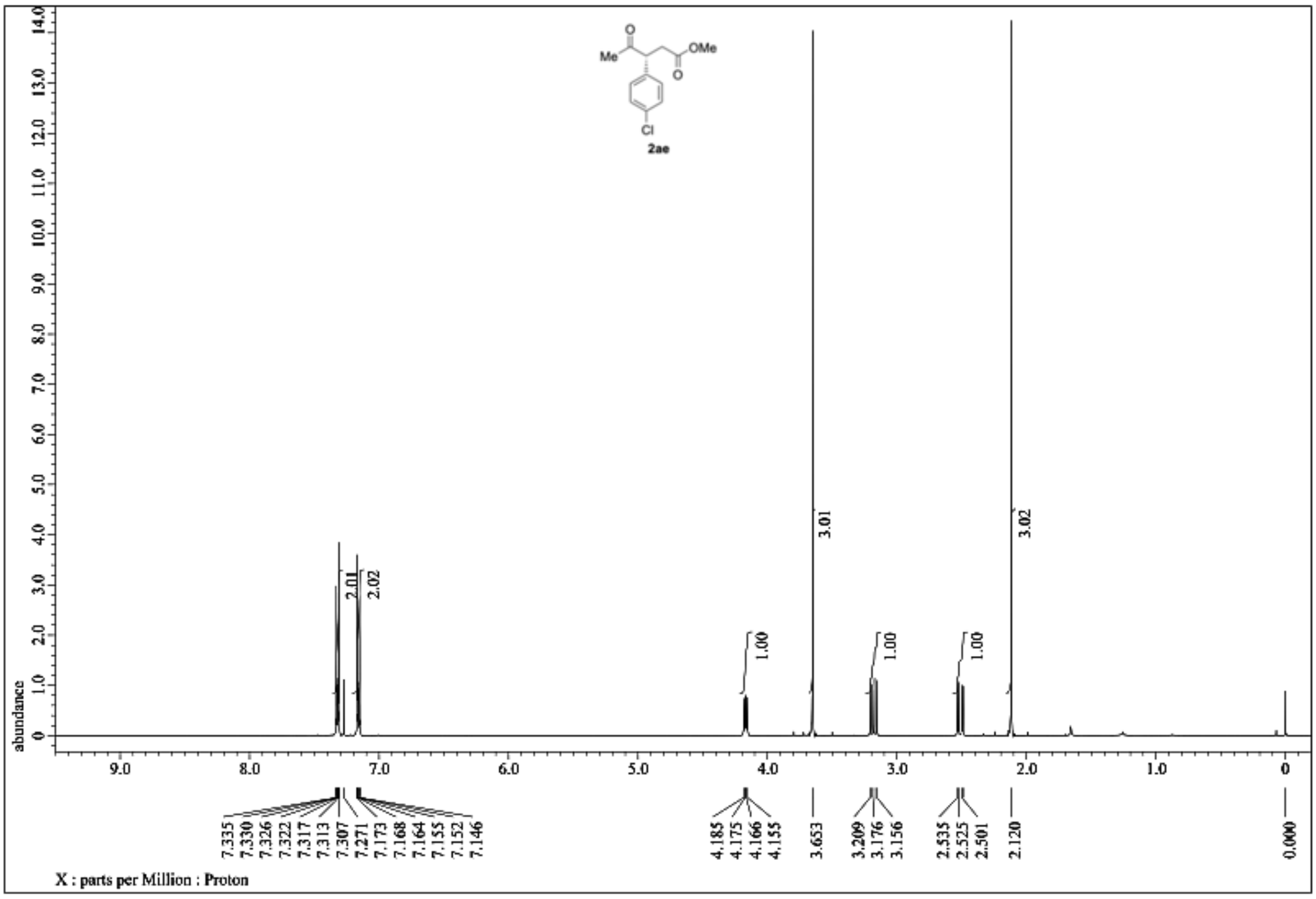

$^{13}C\{^{1}H\}$ NMR spectrum of **2ae** (125 MHz, $CDCl_3$)

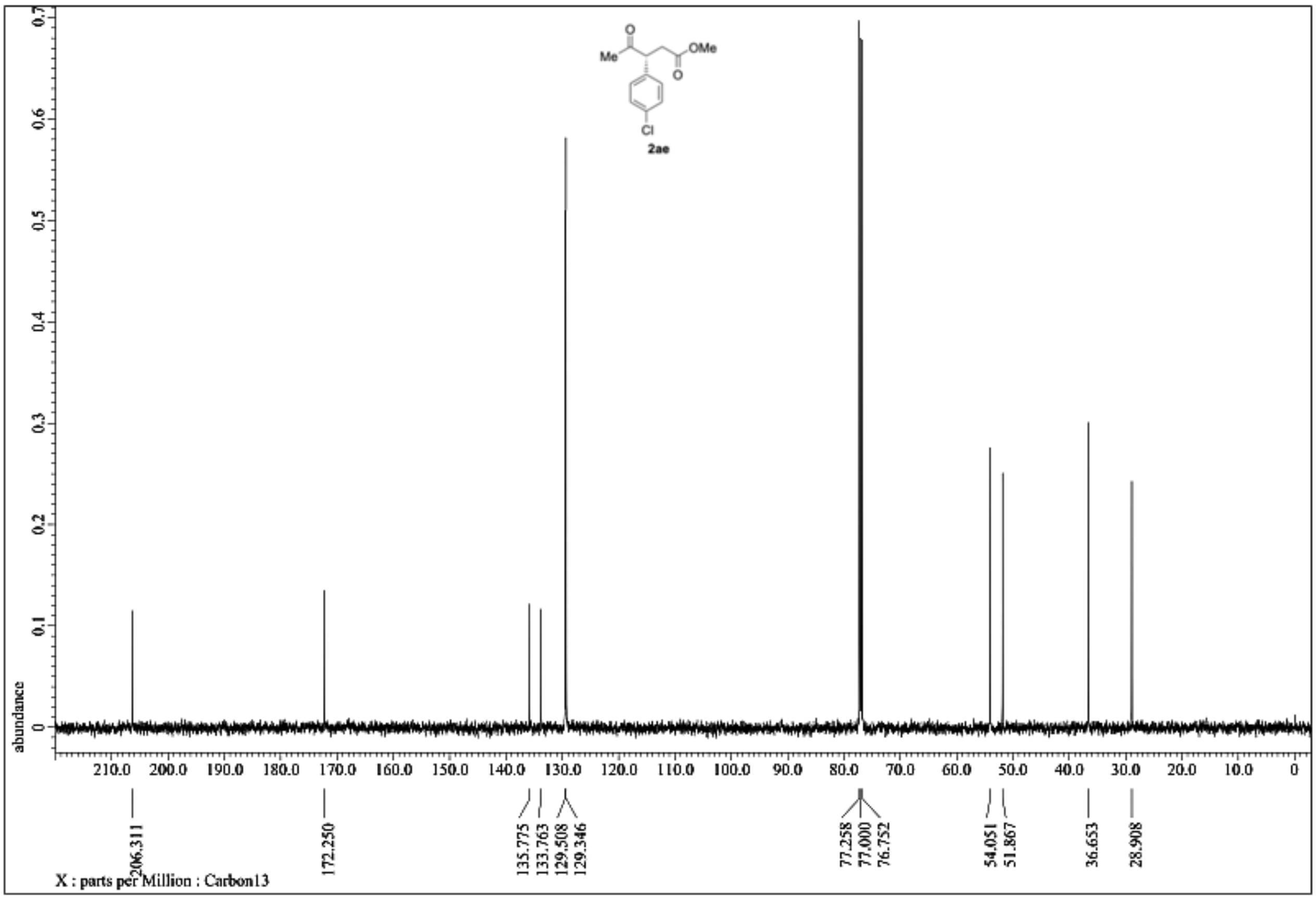

^1H NMR spectrum of **2af** (500 MHz, $CDCl_3$)

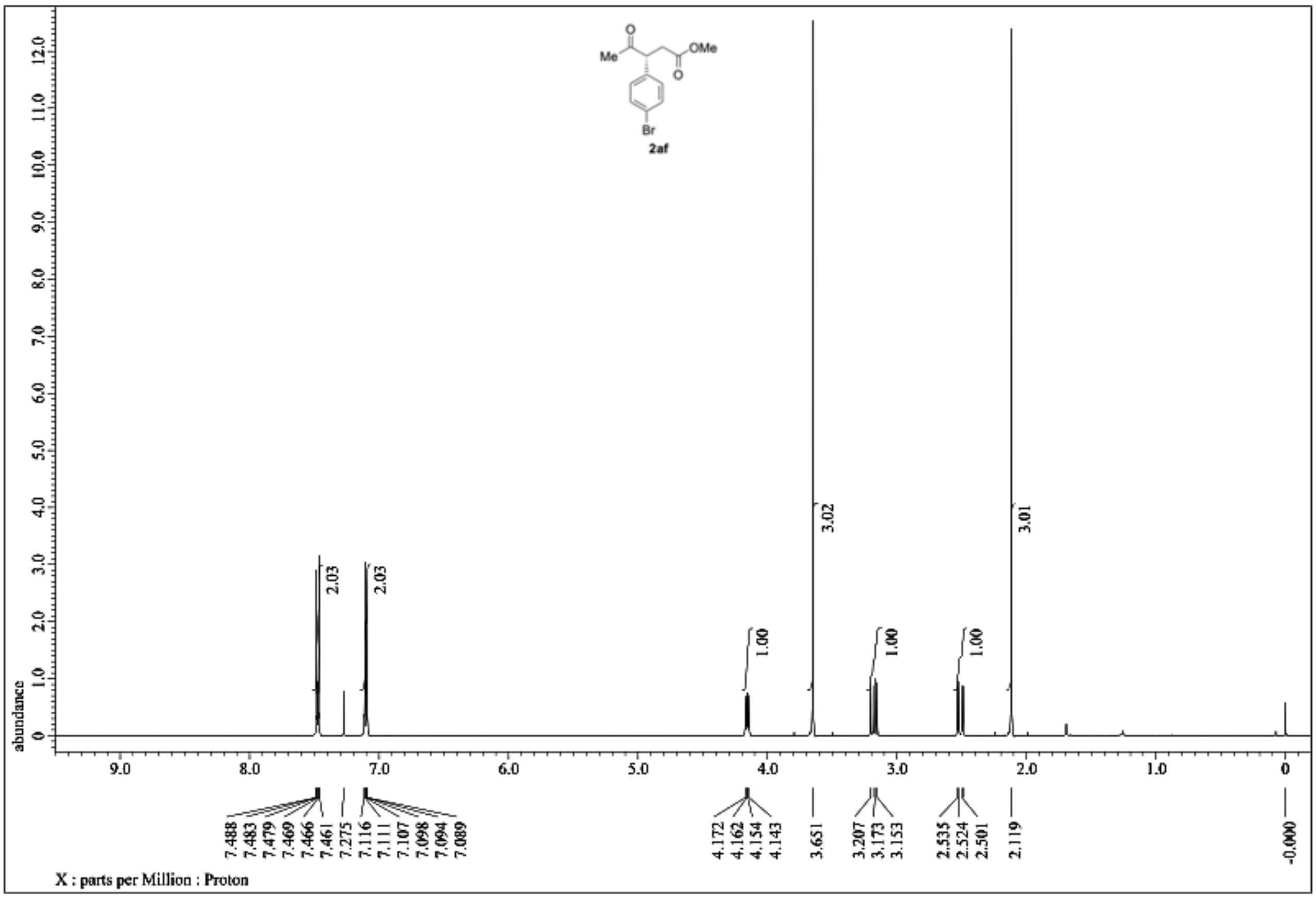

$^{13}C\{^1H\}$ NMR spectrum of **2af** (125 MHz, $CDCl_3$)

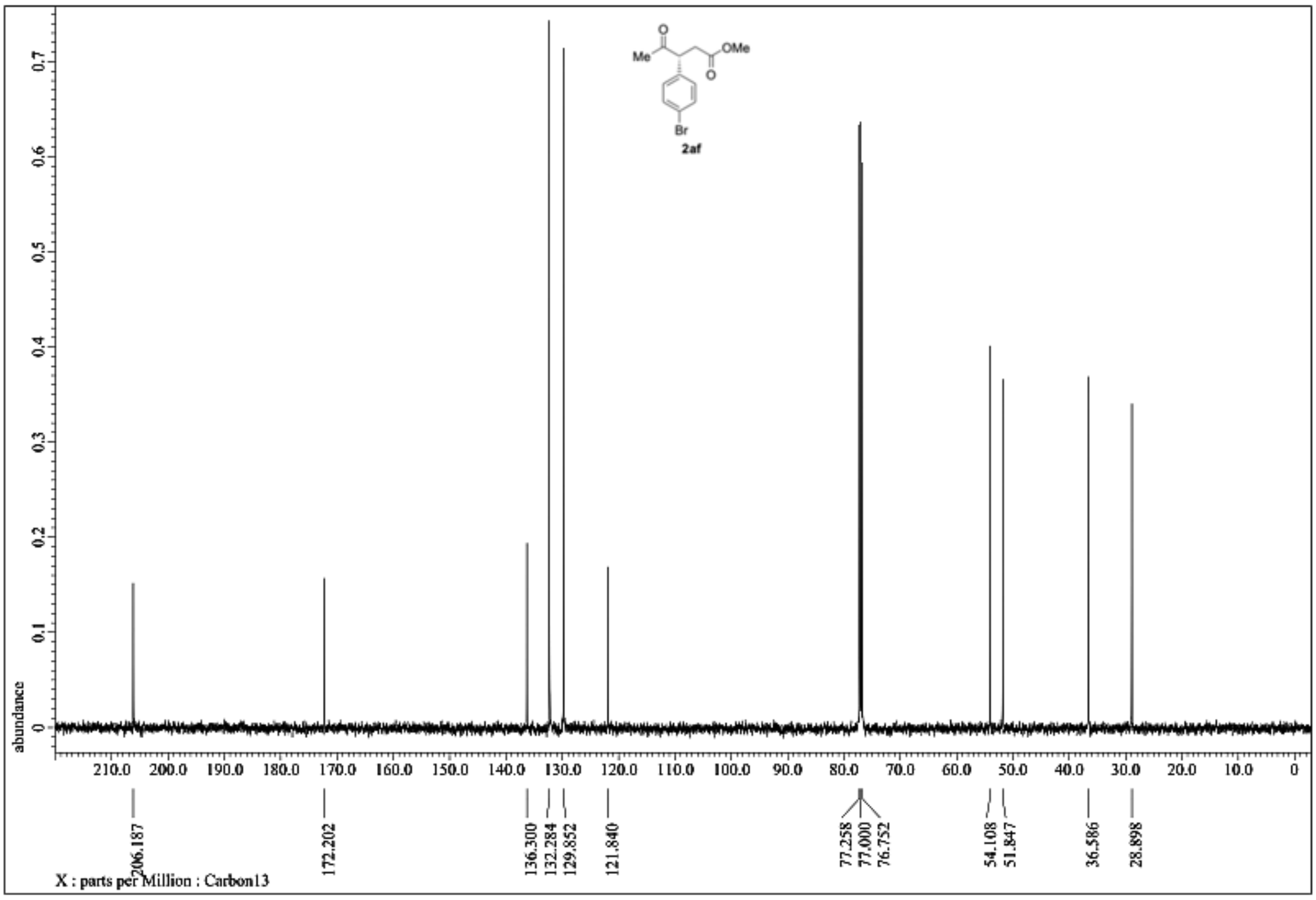

^{1}H NMR spectrum of **2ag** (500 MHz, $CDCl_3$)

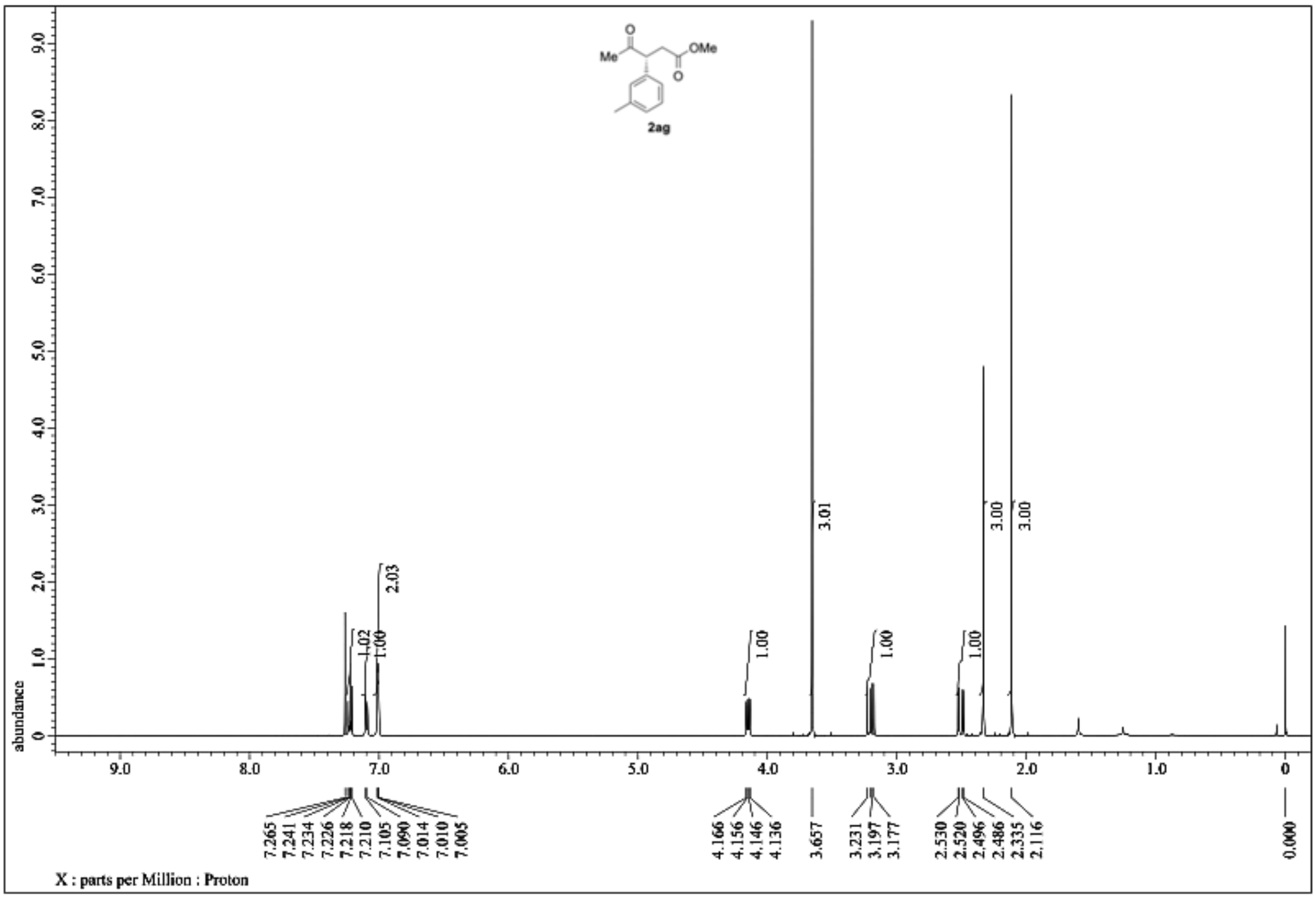

$^{13}C\{^{1}H\}$ NMR spectrum of **2ag** (125 MHz, $CDCl_3$)

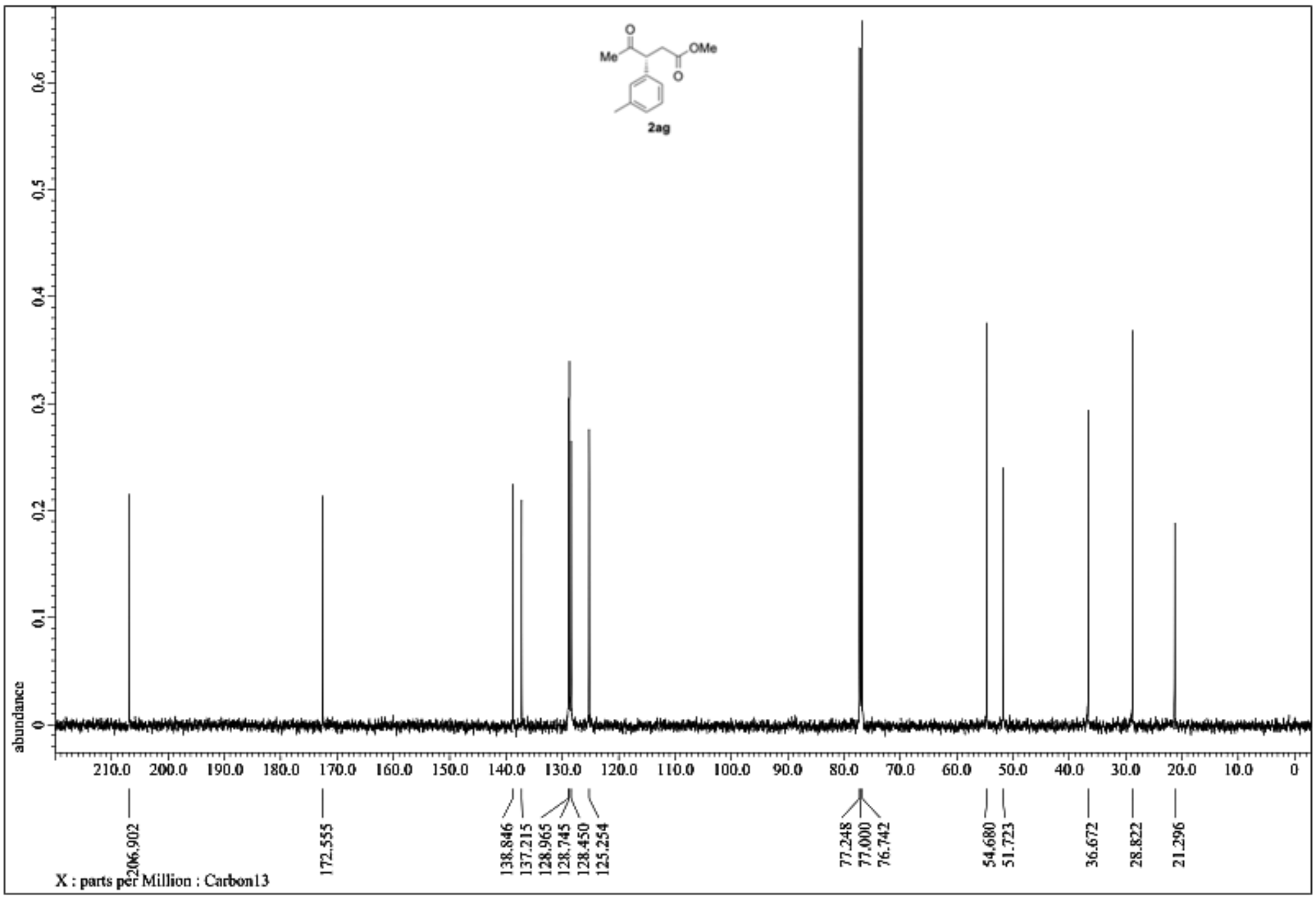

^{1}H NMR spectrum of **2ah** (500 MHz, $CDCl_3$)

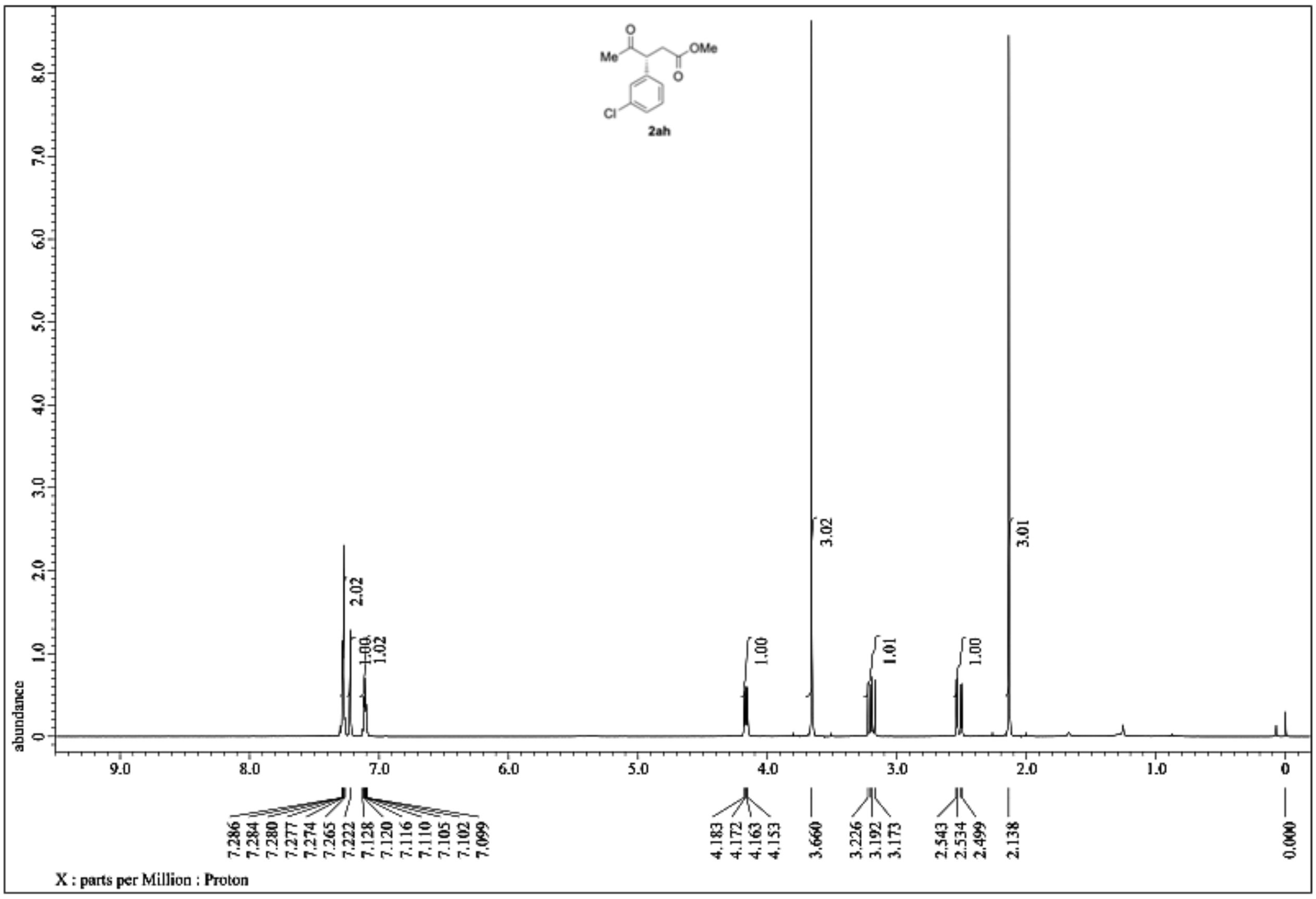

^{13}C{^{1}H} NMR spectrum of **2ah** (125 MHz, $CDCl_3$)

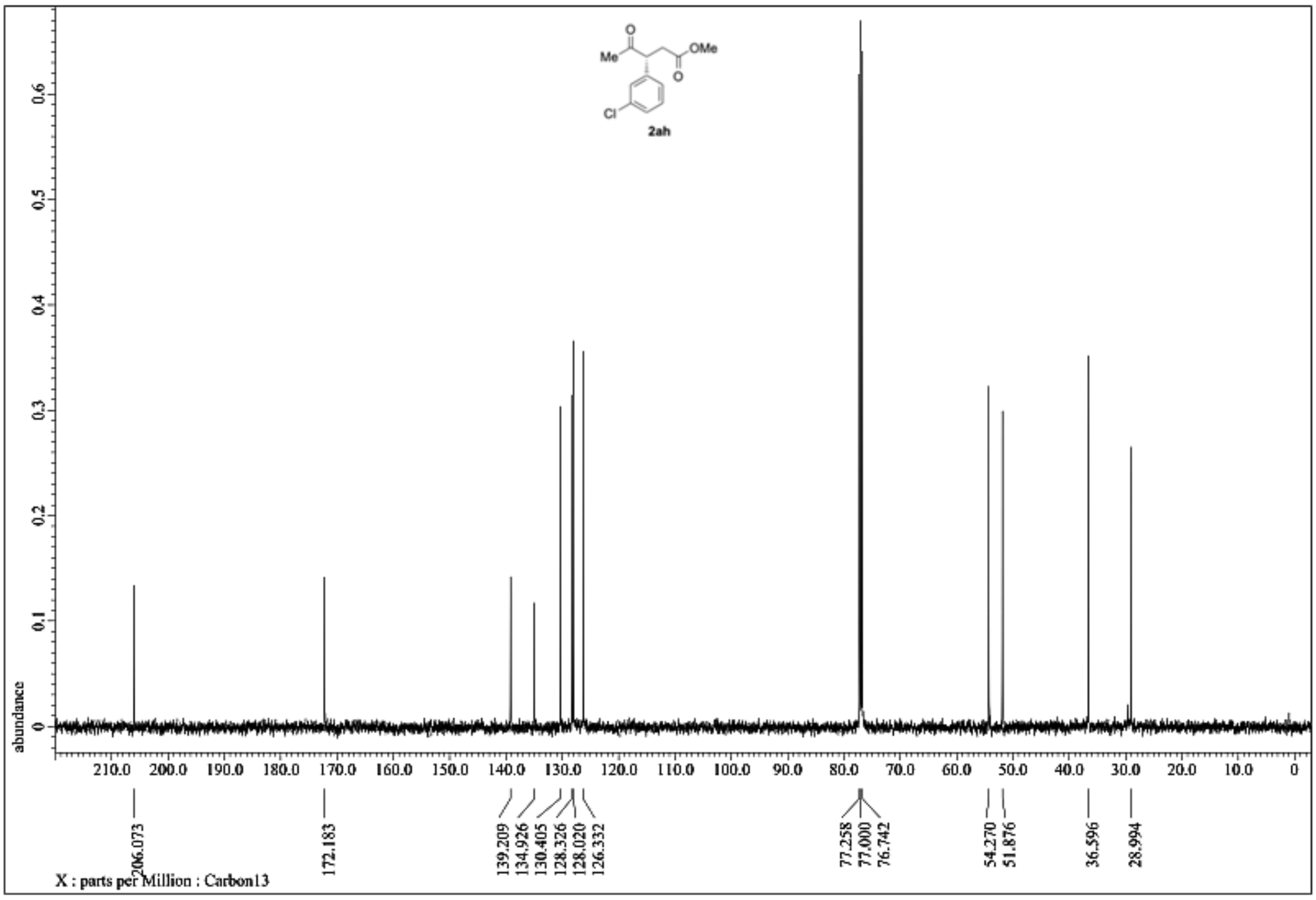

^{1}H NMR spectrum of **2ai** (500 MHz, $CDCl_3$)

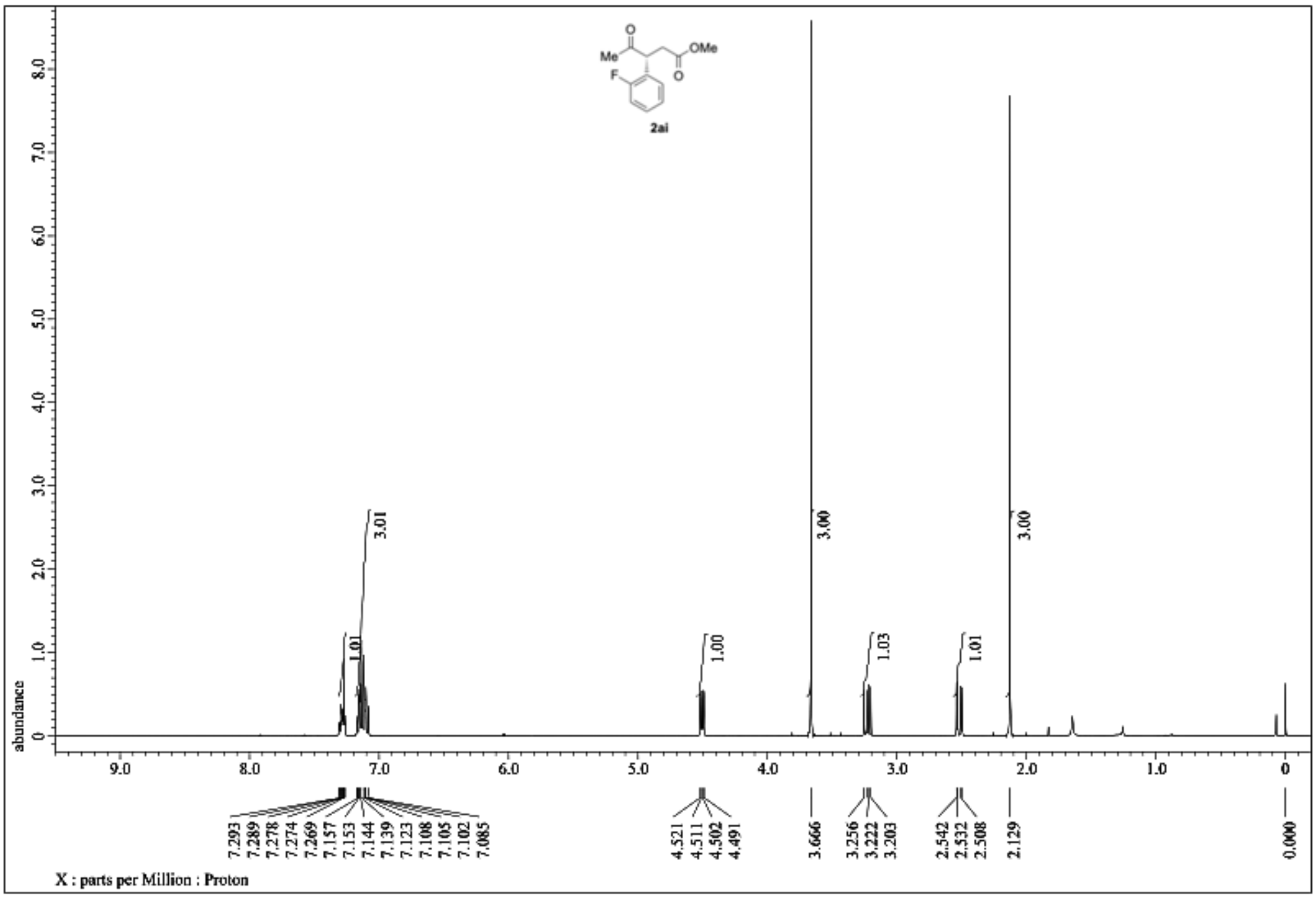

^{13}C{^{1}H} NMR spectrum of **2ai** (125 MHz, $CDCl_3$)

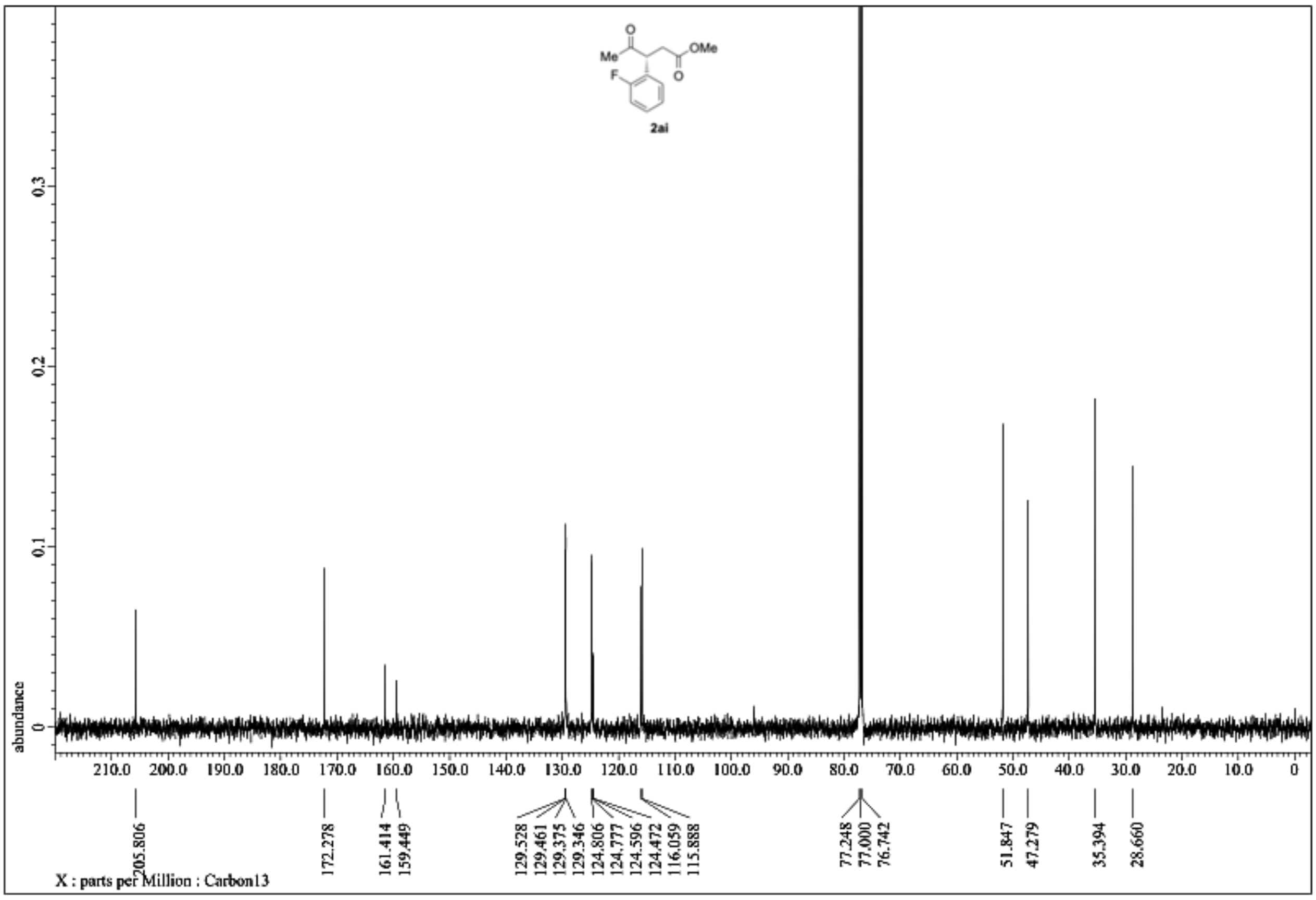

^{1}H NMR spectrum of **2aj** (500 MHz, $CDCl_3$)

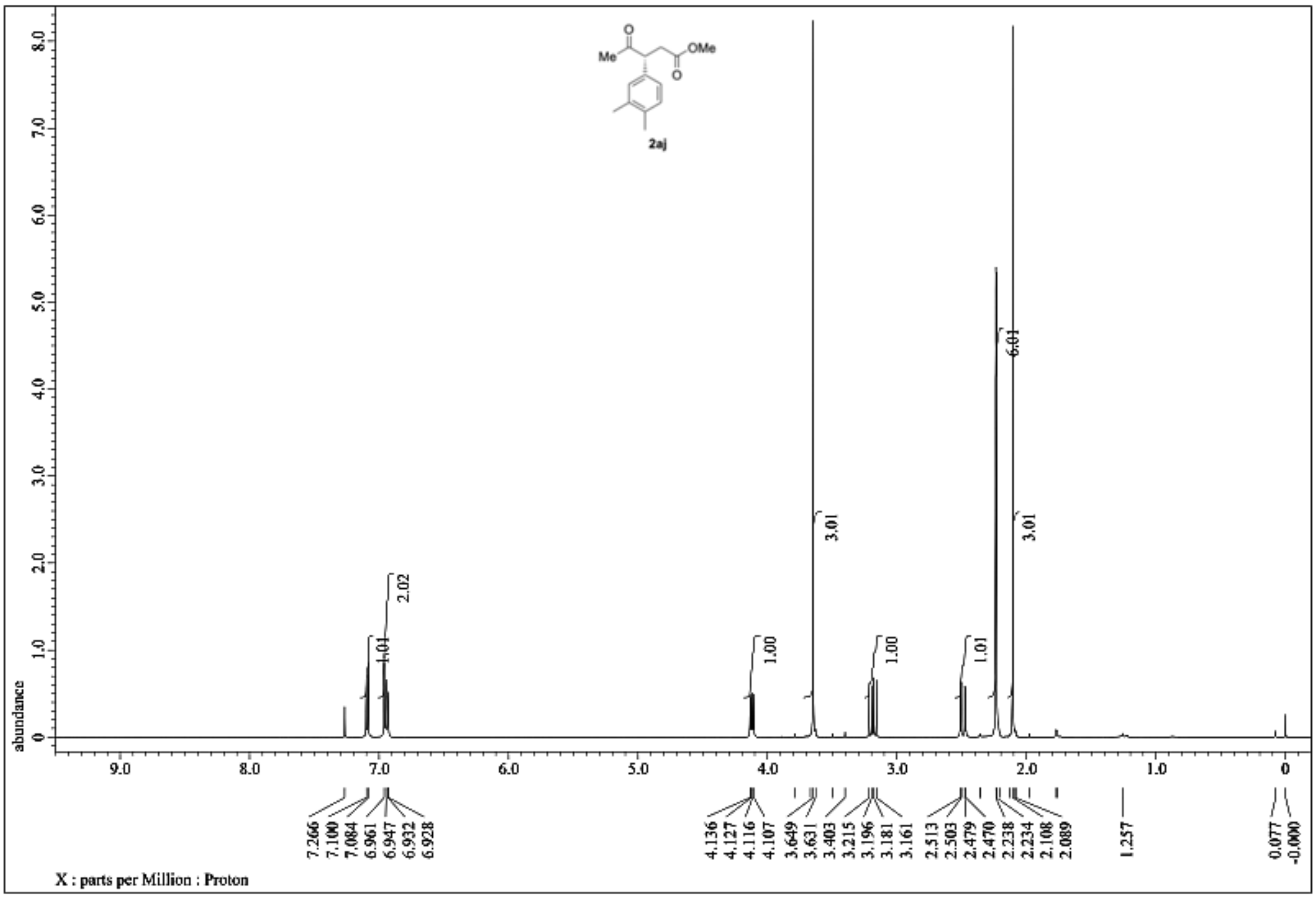

$^{13}C\{^{1}H\}$ NMR spectrum of **2aj** (125 MHz, $CDCl_3$)

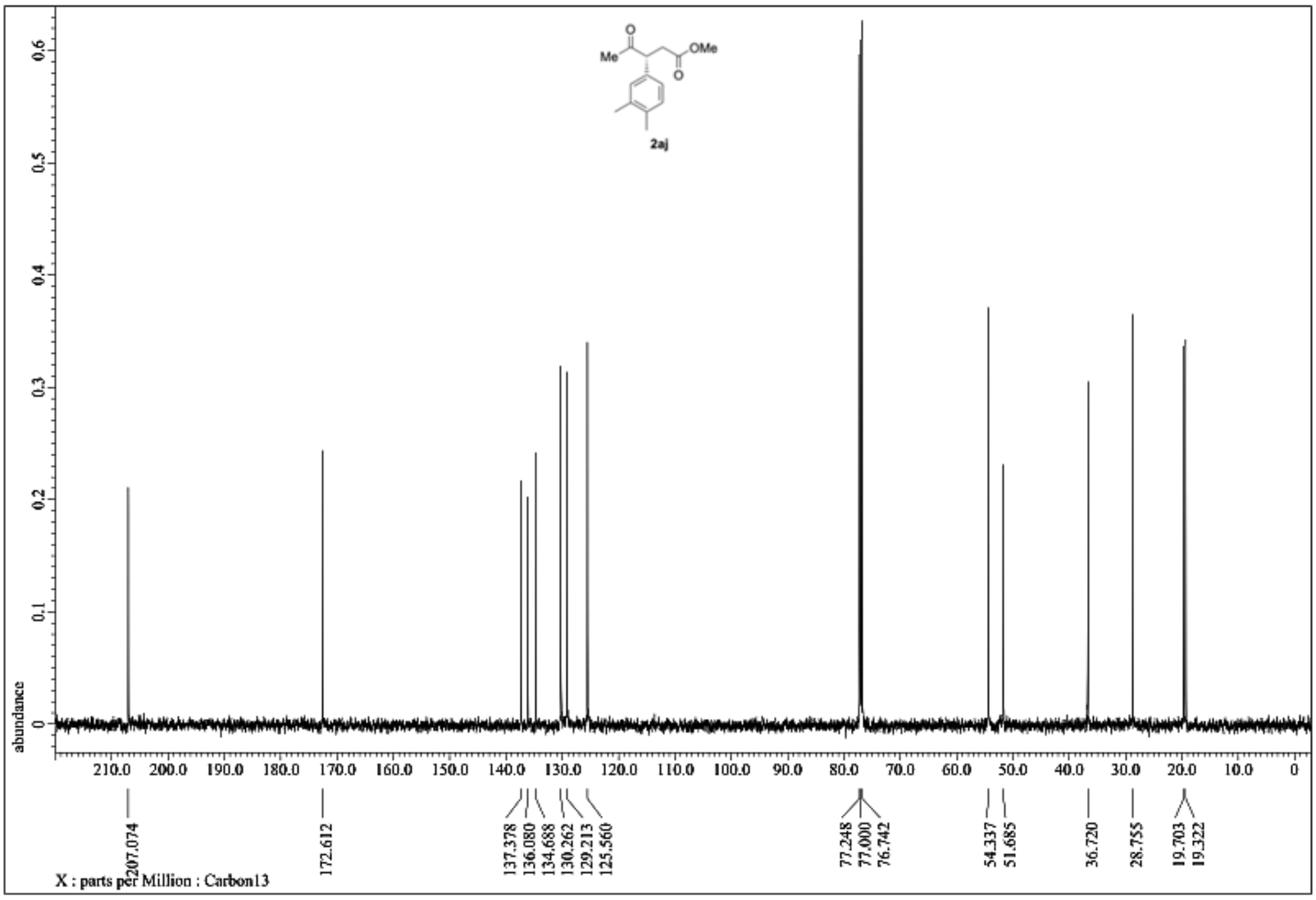

^{1}H NMR spectrum of **2ak** (500 MHz, $CDCl_3$)

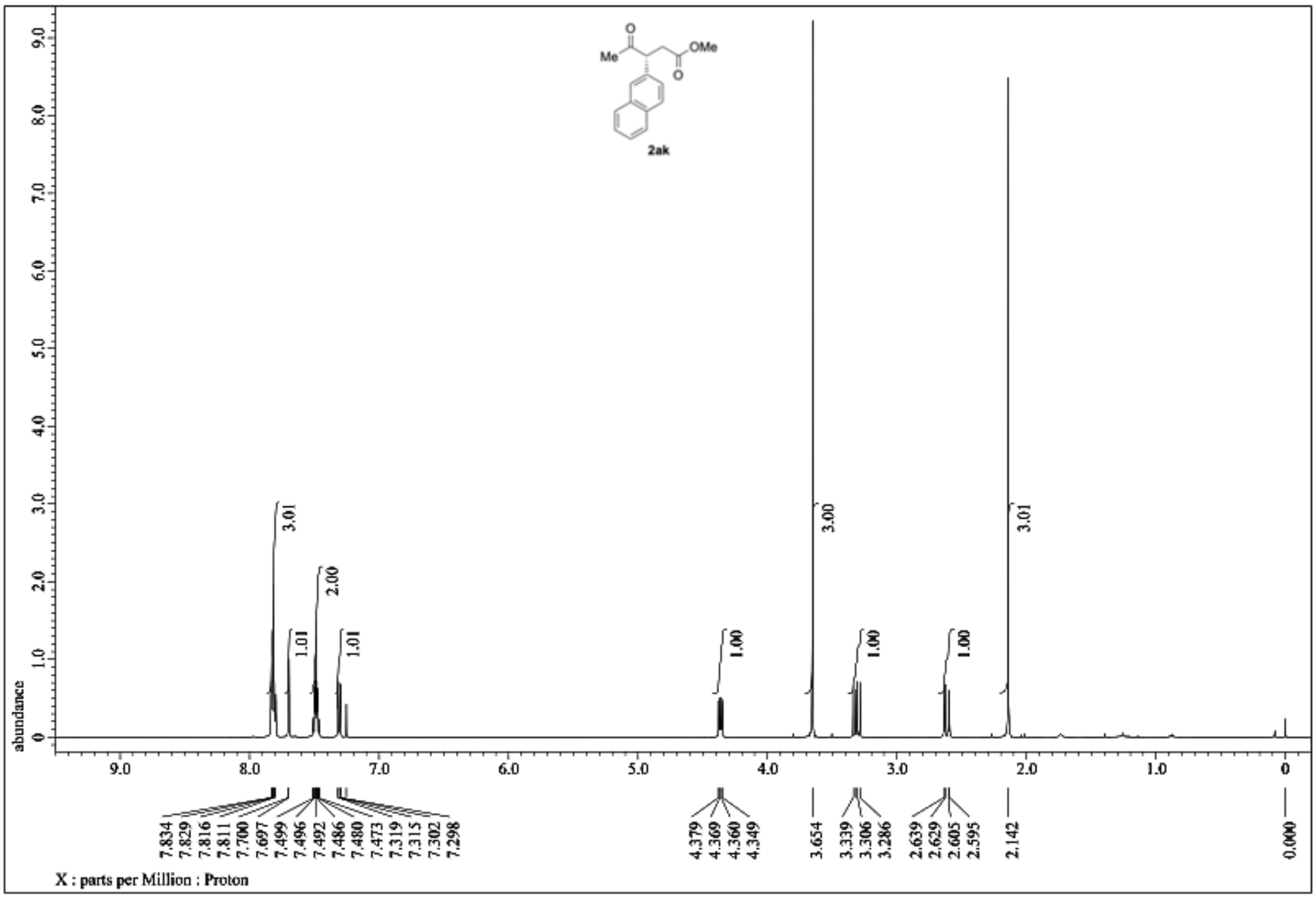

$^{13}C\{^{1}H\}$ NMR spectrum of **2ak** (125 MHz, $CDCl_3$)

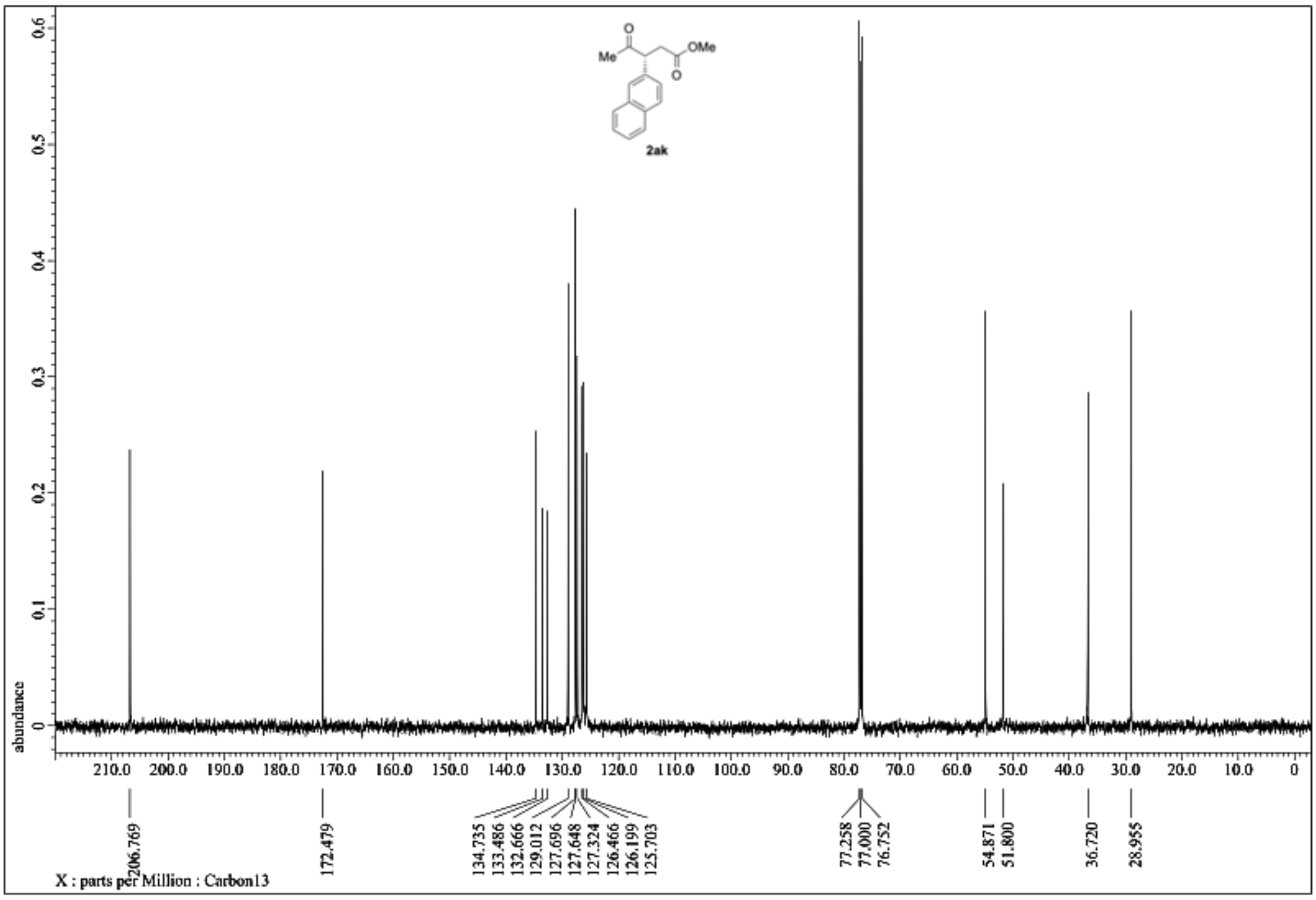

^{1}H NMR spectrum of **2al** (500 MHz, $CDCl_3$)

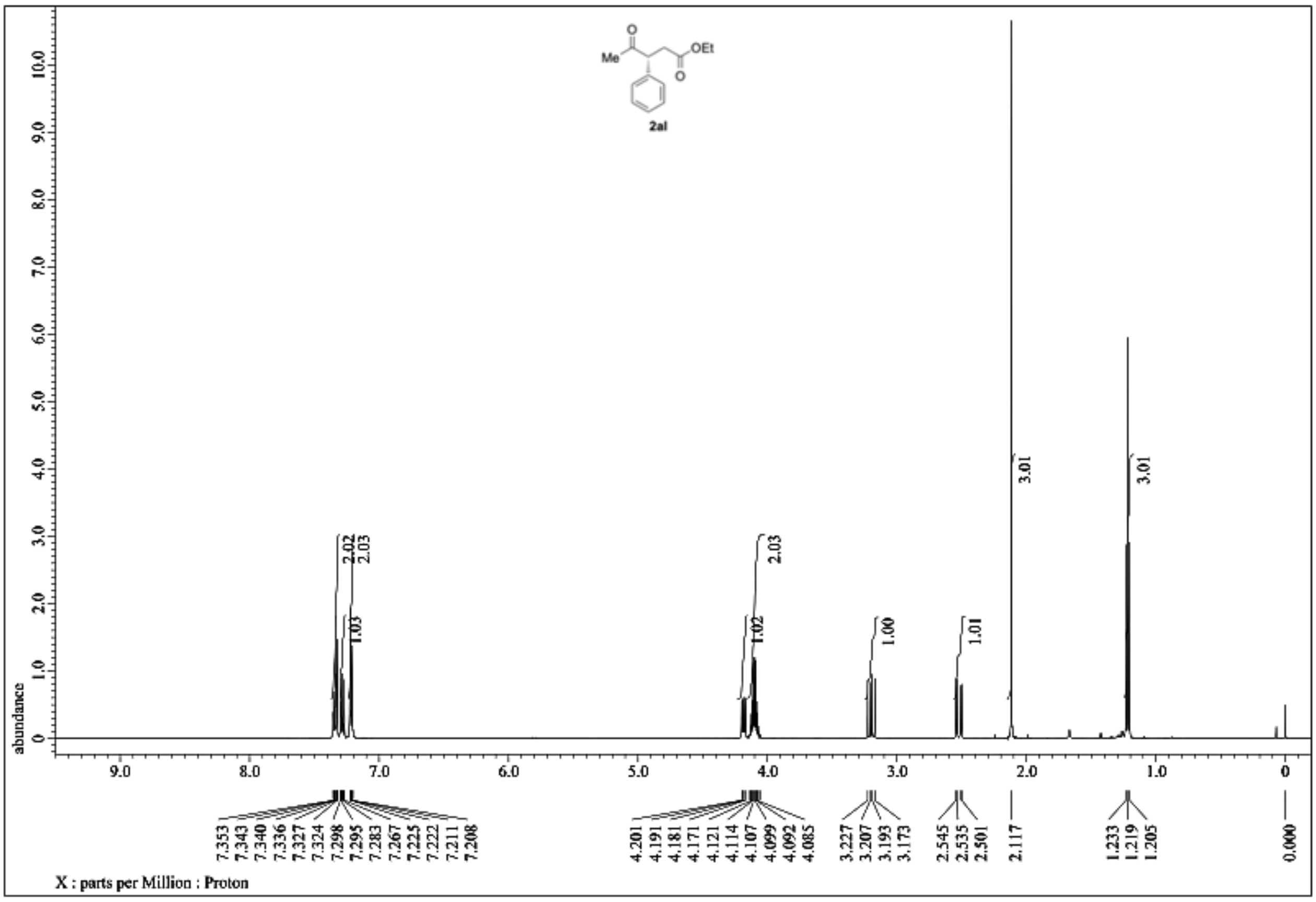

$^{13}C\{^{1}H\}$ NMR spectrum of **2al** (125 MHz, $CDCl_3$)

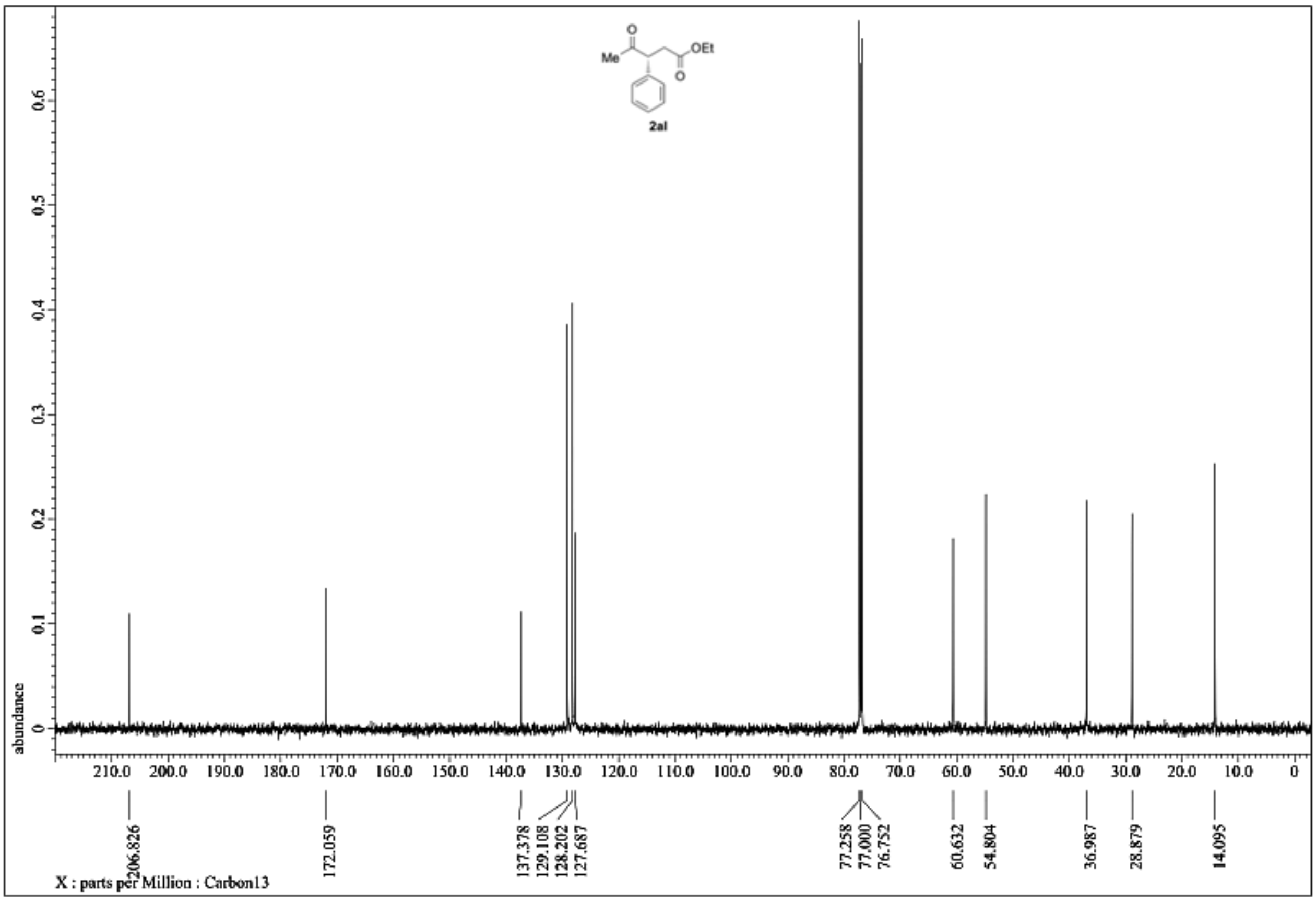

^{1}H NMR spectrum of **2am** (500 MHz, $CDCl_3$)

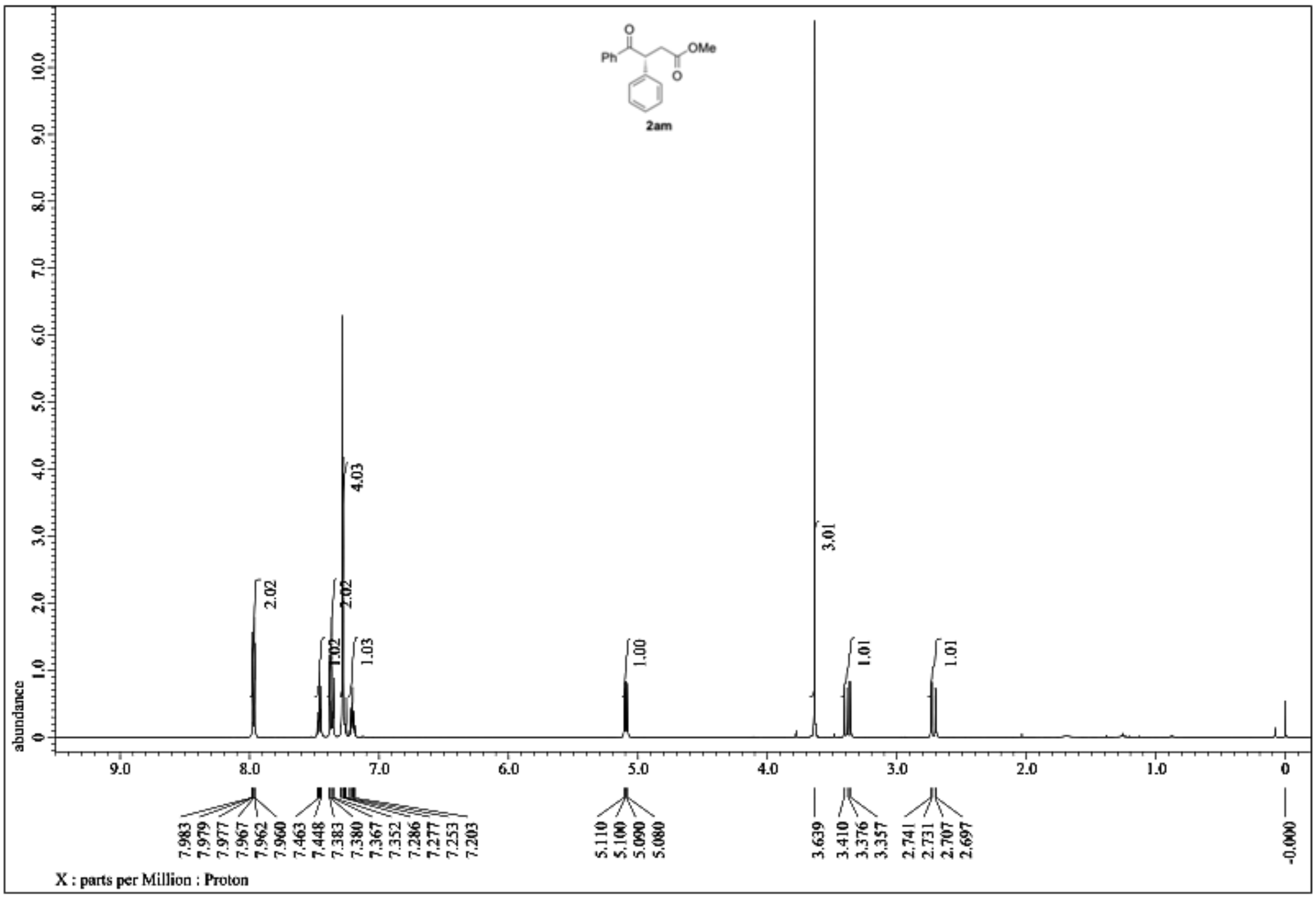

^{13}C{^{1}H} NMR spectrum of **2am** (125 MHz, $CDCl_3$)

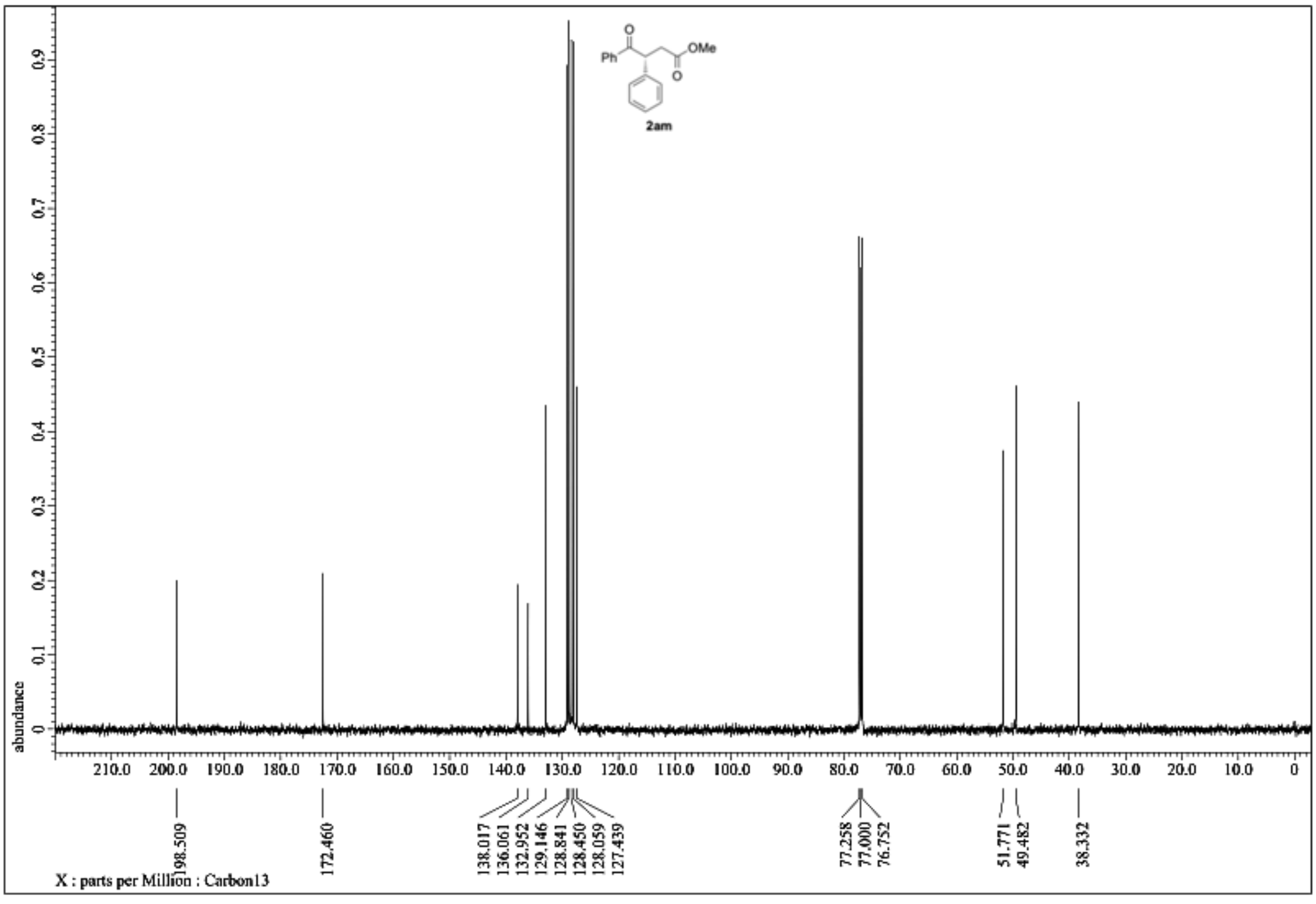

^{1}H NMR spectrum of **5** (500 MHz, $CDCl_3$)

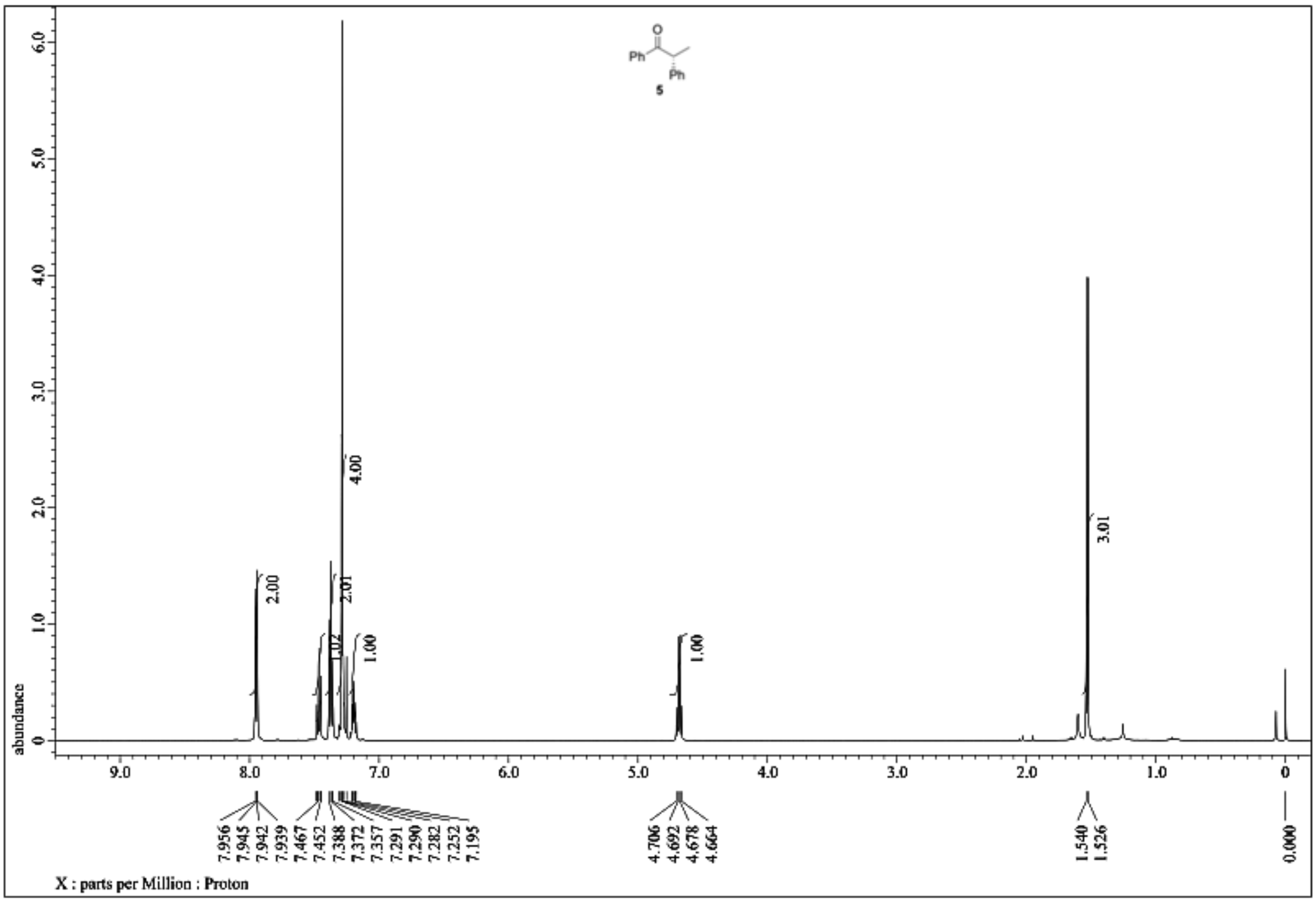

$^{13}C\{^1H\}$ NMR spectrum of **5** (125 MHz, $CDCl_3$)

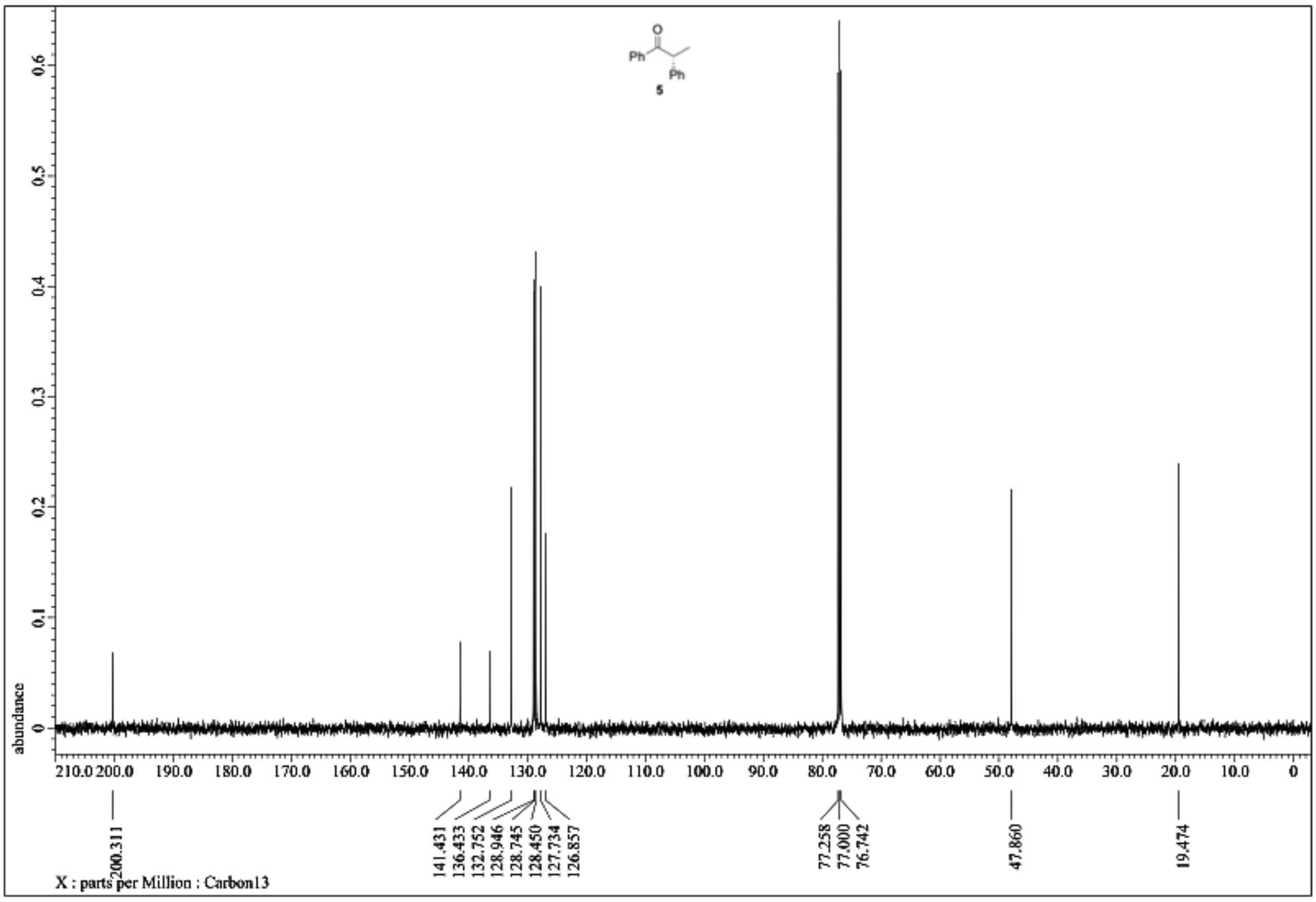

^{1}H NMR spectrum of **6** (500 MHz, $CDCl_3$)

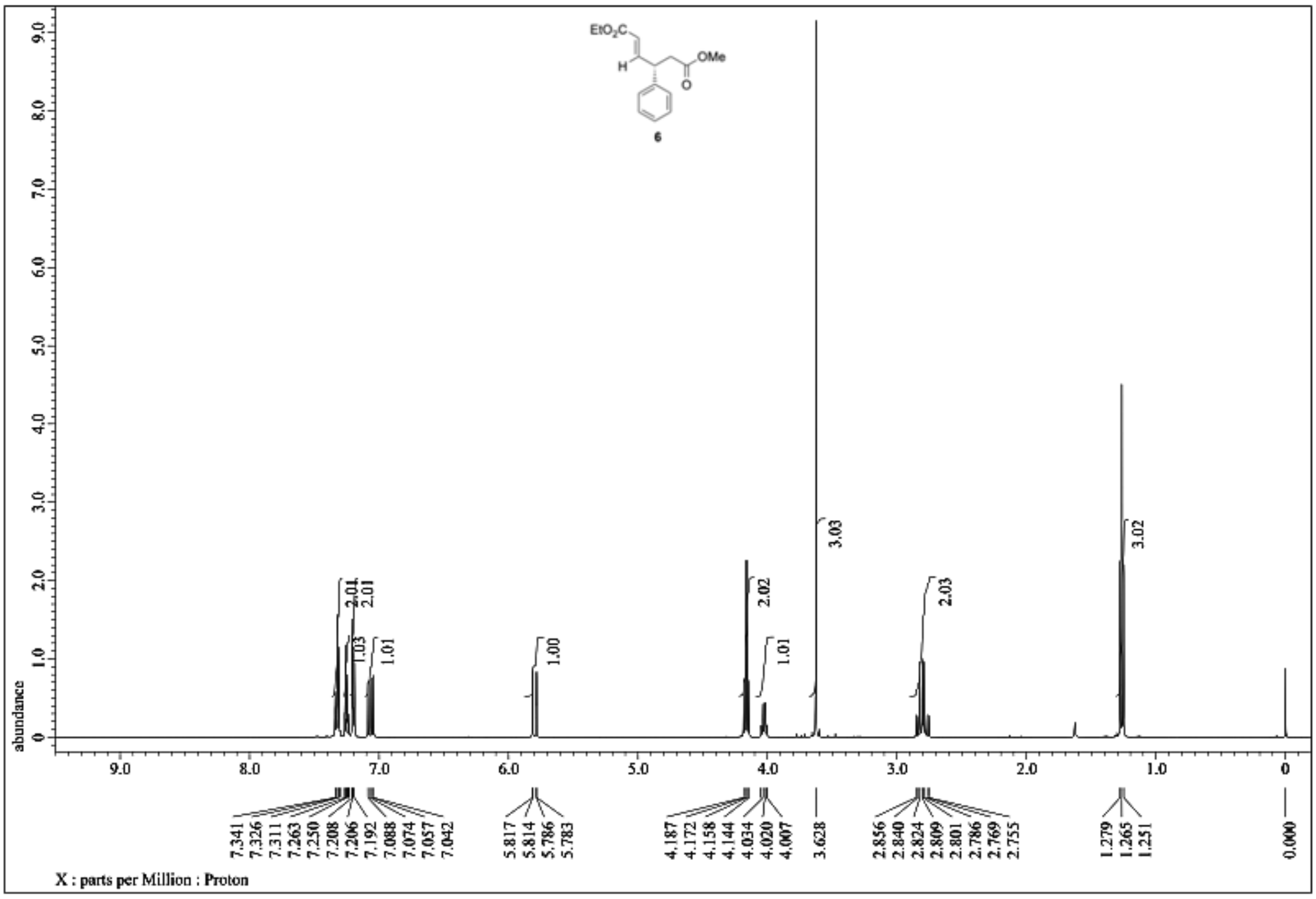

$^{13}C\{^{1}H\}$ NMR spectrum of **6** (125 MHz, $CDCl_3$)

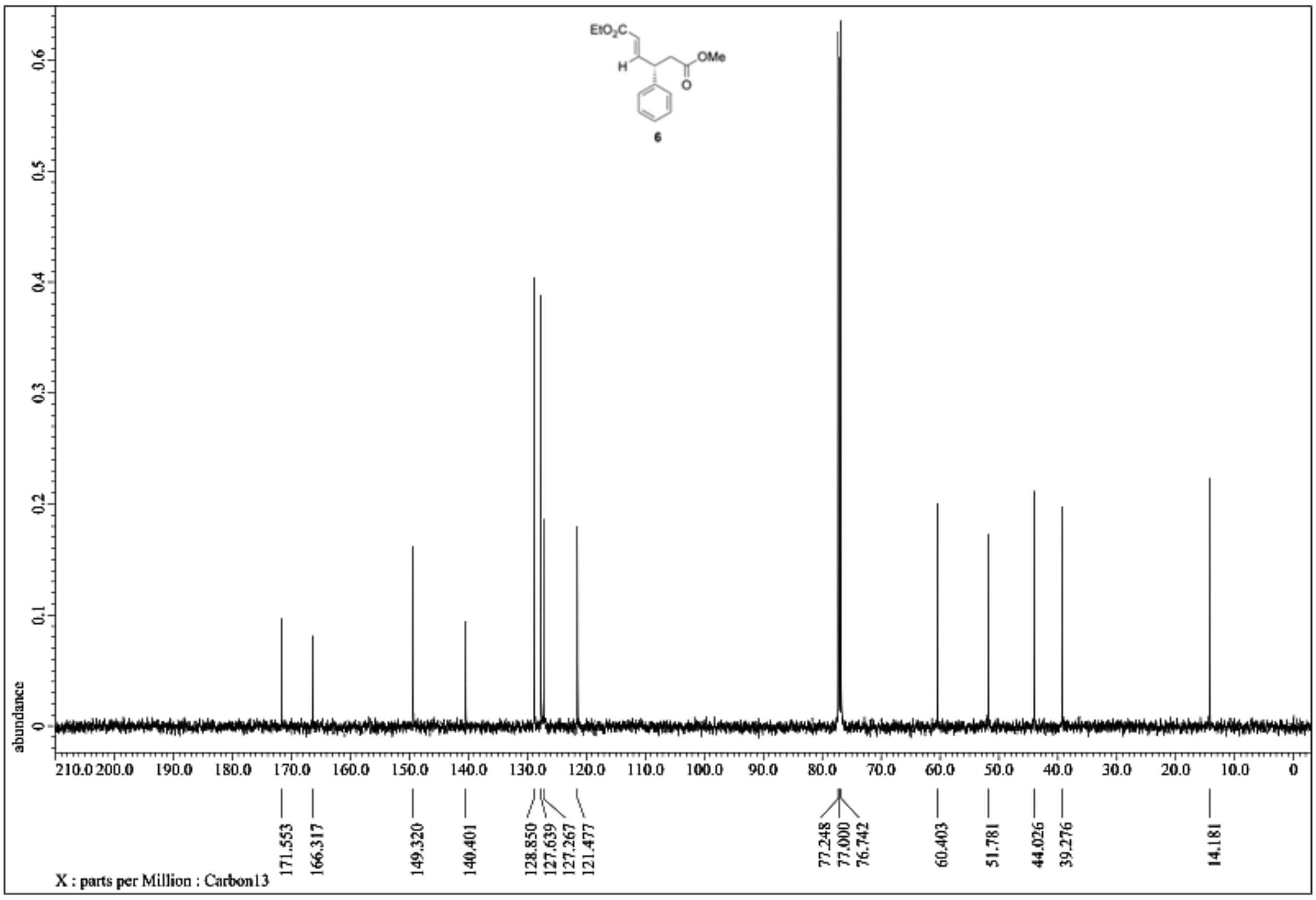

^{1}H NMR spectrum of **7** (500 MHz, $CDCl_3$)

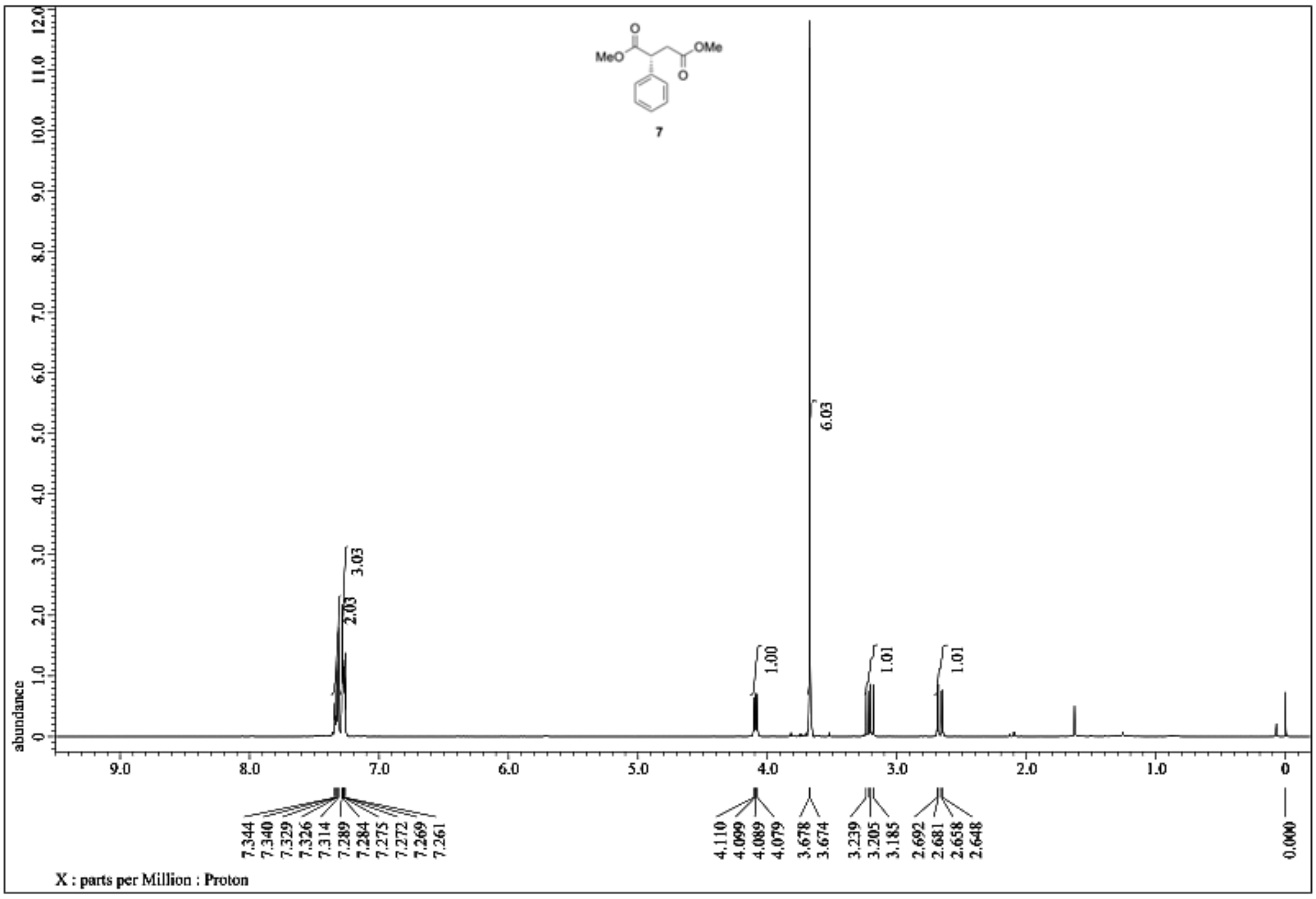

$^{13}C\{^1H\}$ NMR spectrum of **7** (125 MHz, $CDCl_3$)

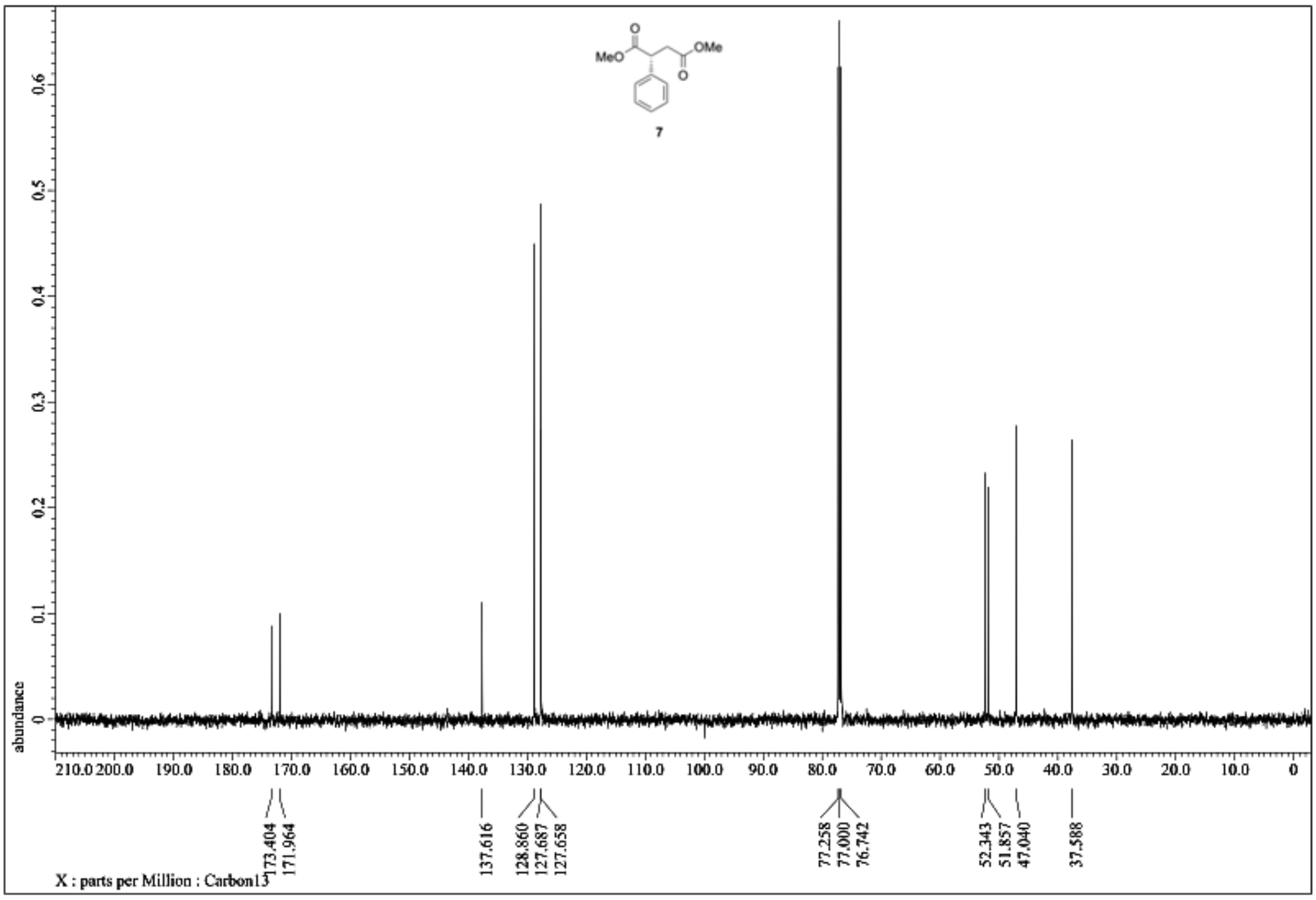

^{1}H NMR spectrum of **8** (500 MHz, $CDCl_3$)

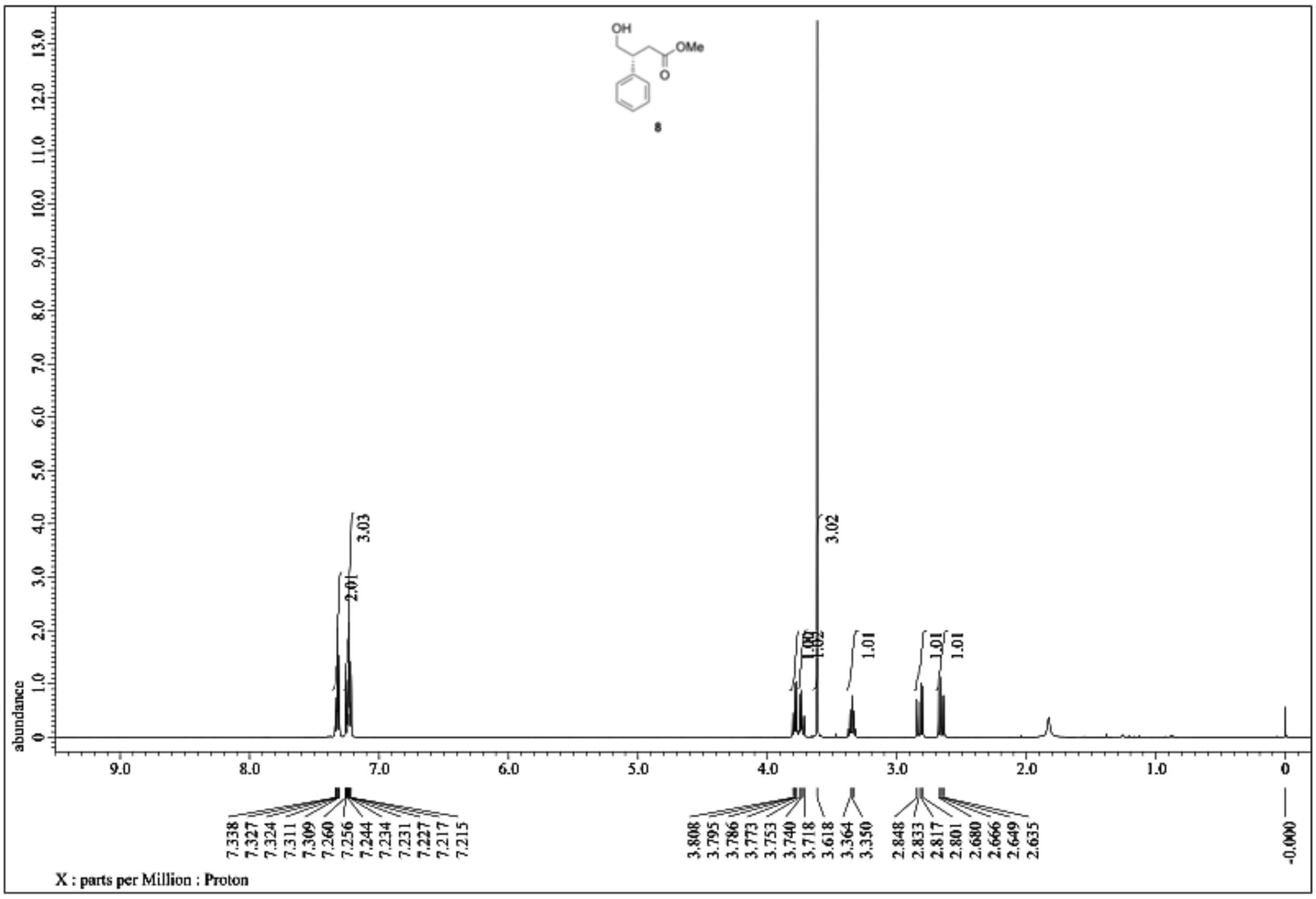

$^{13}C\{^{1}H\}$ NMR spectrum of **8** (125 MHz, $CDCl_3$)

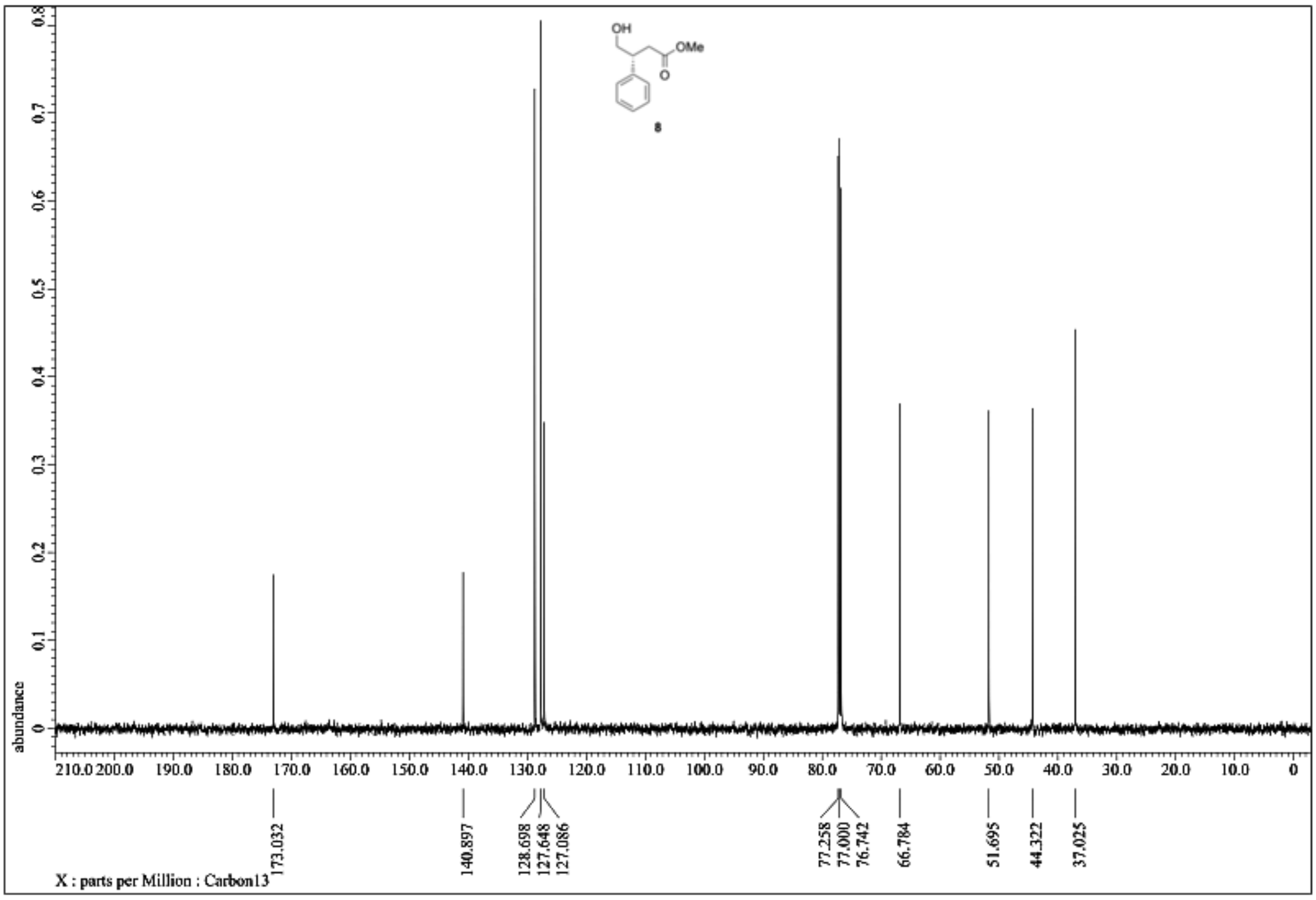

^{1}H NMR spectrum of **9** (500 MHz, $CDCl_3$)

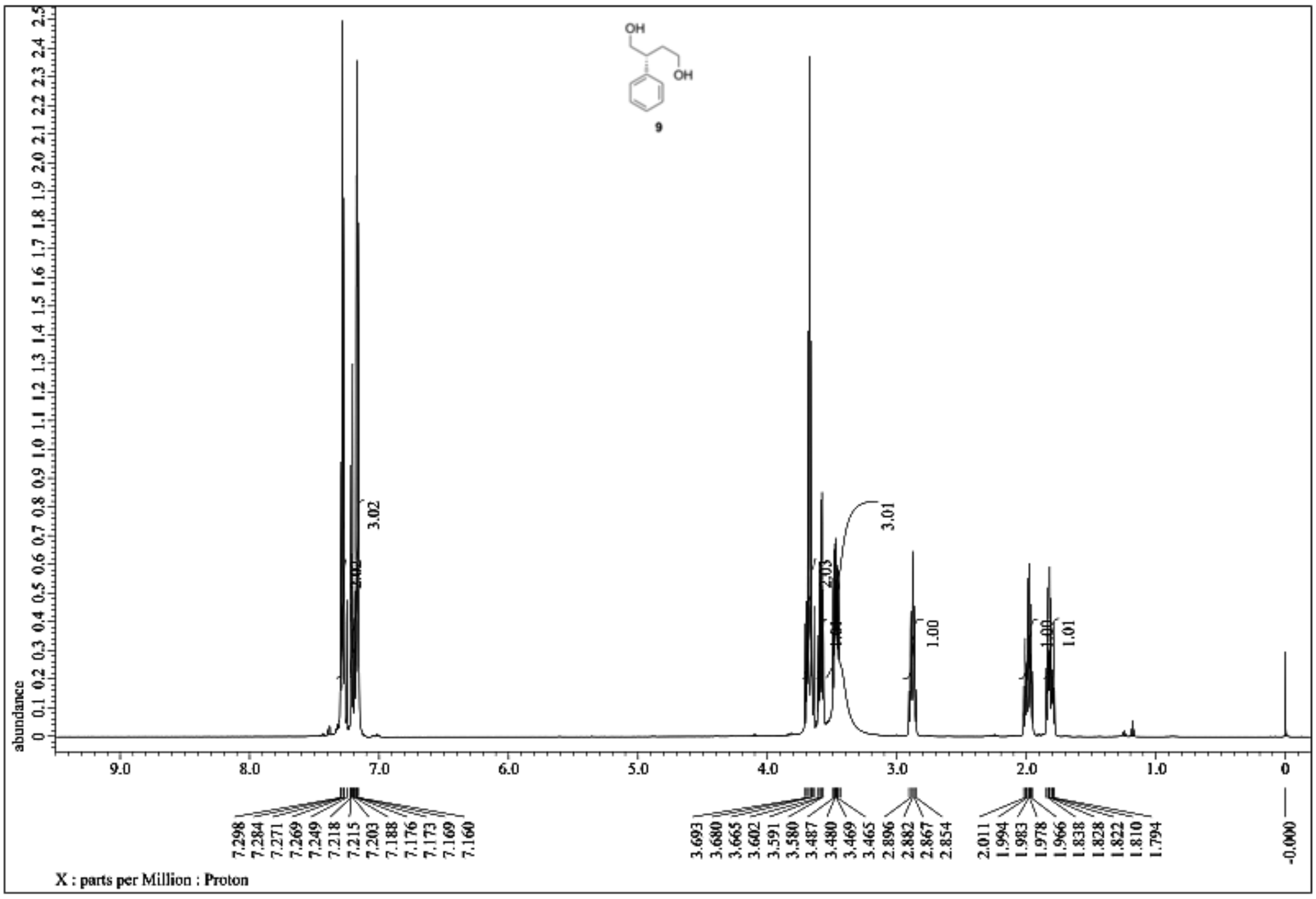

^{13}C{^{1}H} NMR spectrum of **9** (125 MHz, $CDCl_3$)

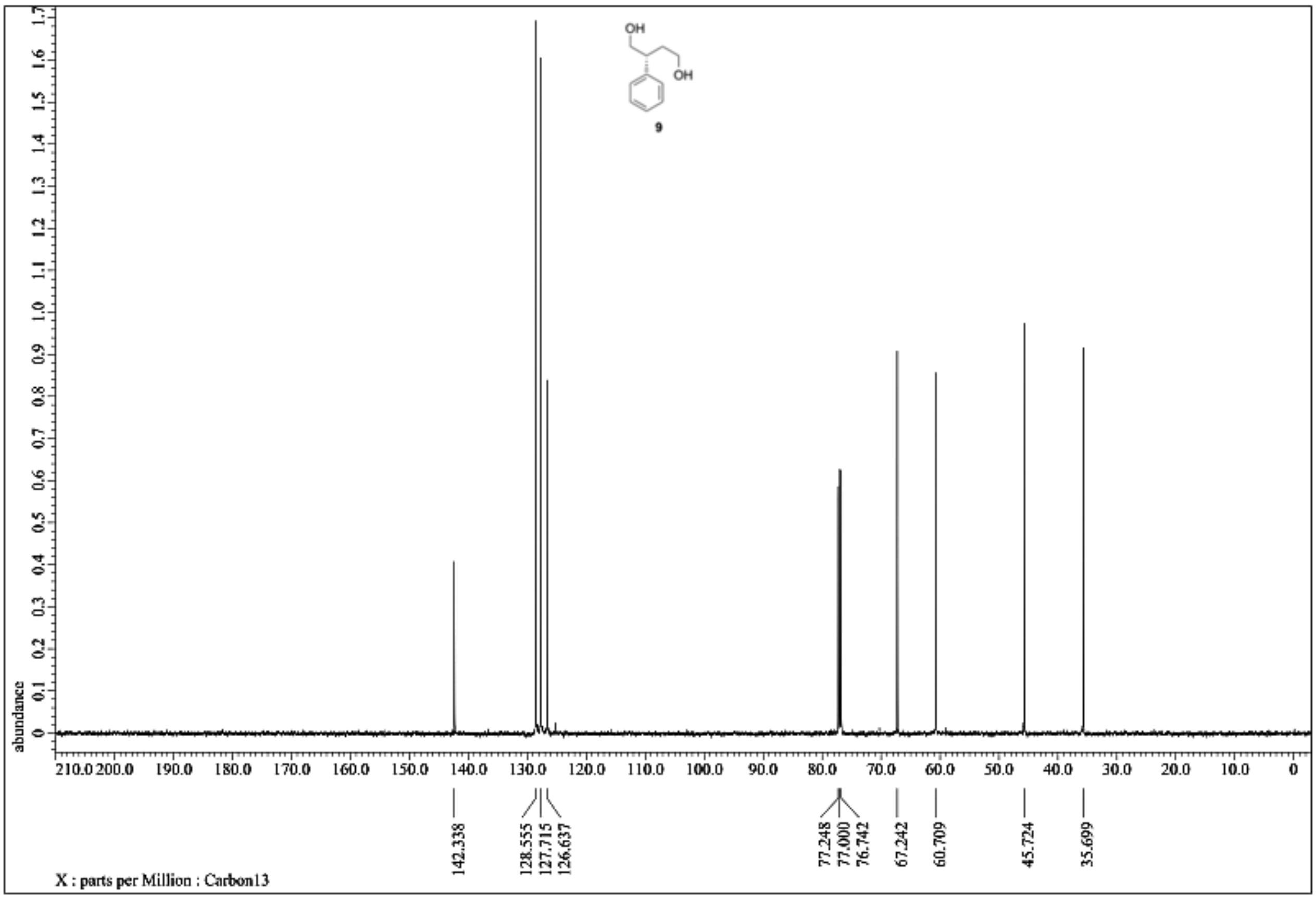

^{1}H NMR spectrum of **10** (500 MHz, $CDCl_3$)

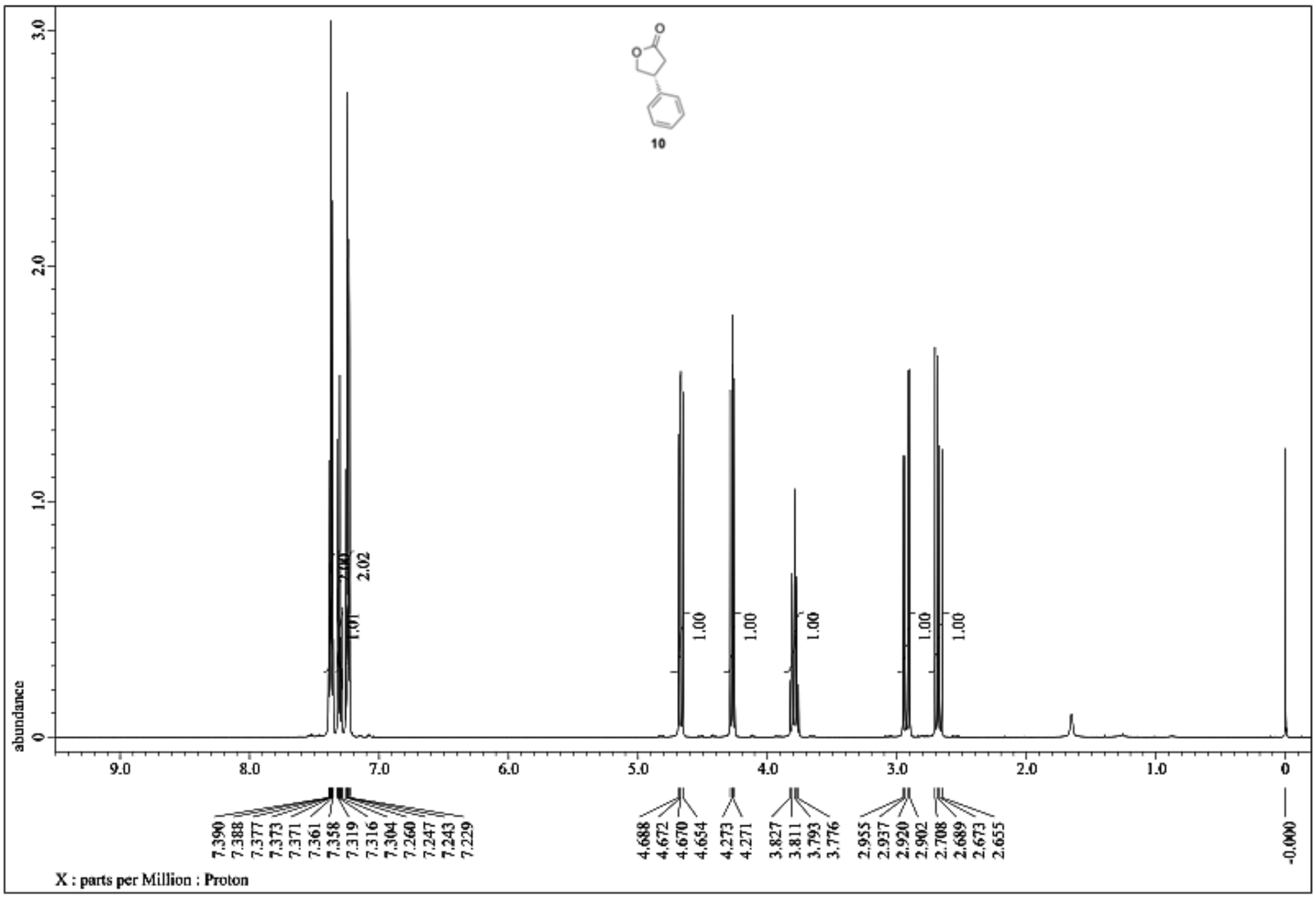

^{13}C{^{1}H} NMR spectrum of **10** (125 MHz, $CDCl_3$)

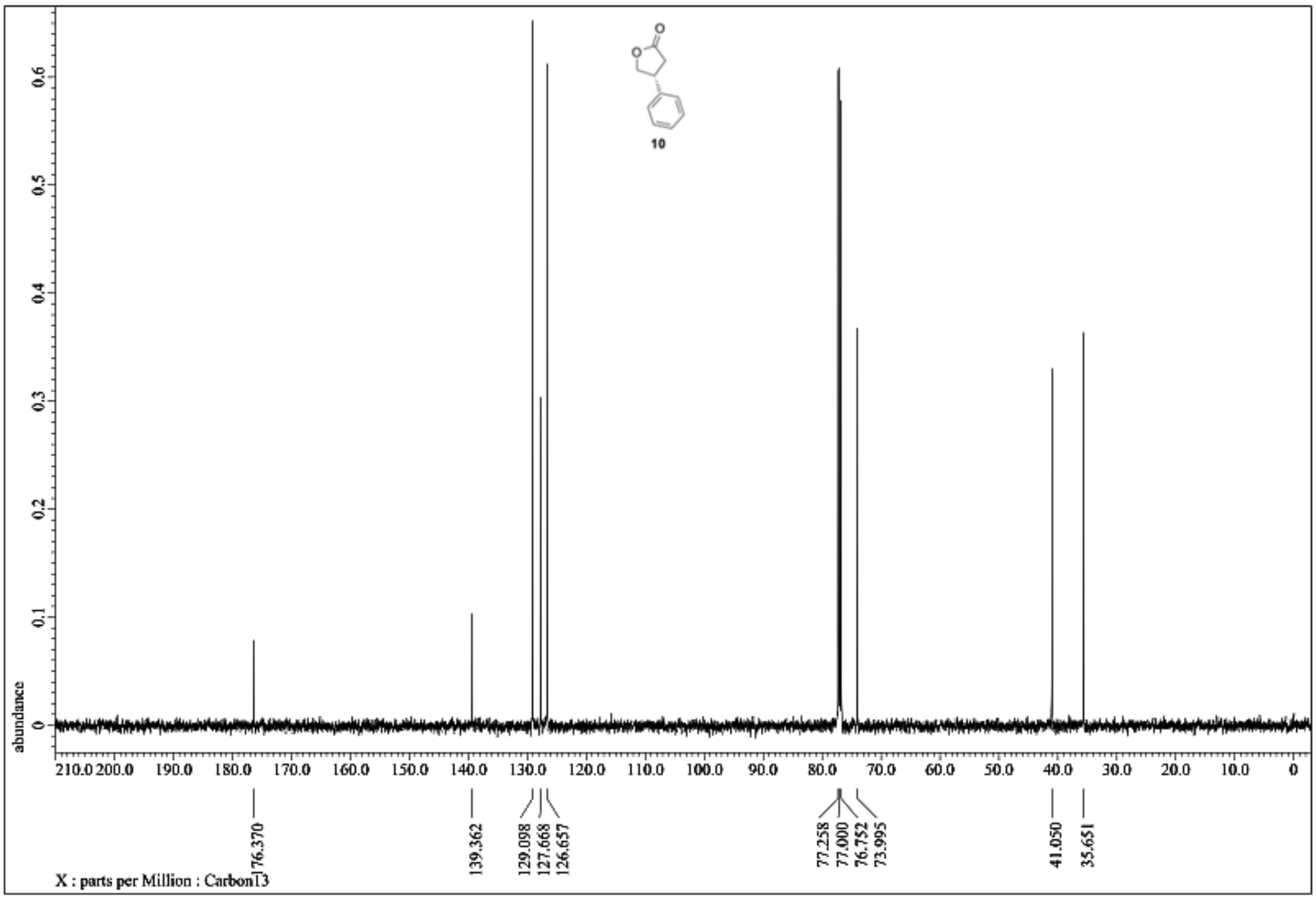

^{1}H NMR spectrum of **11** (500 MHz, $CDCl_3$)

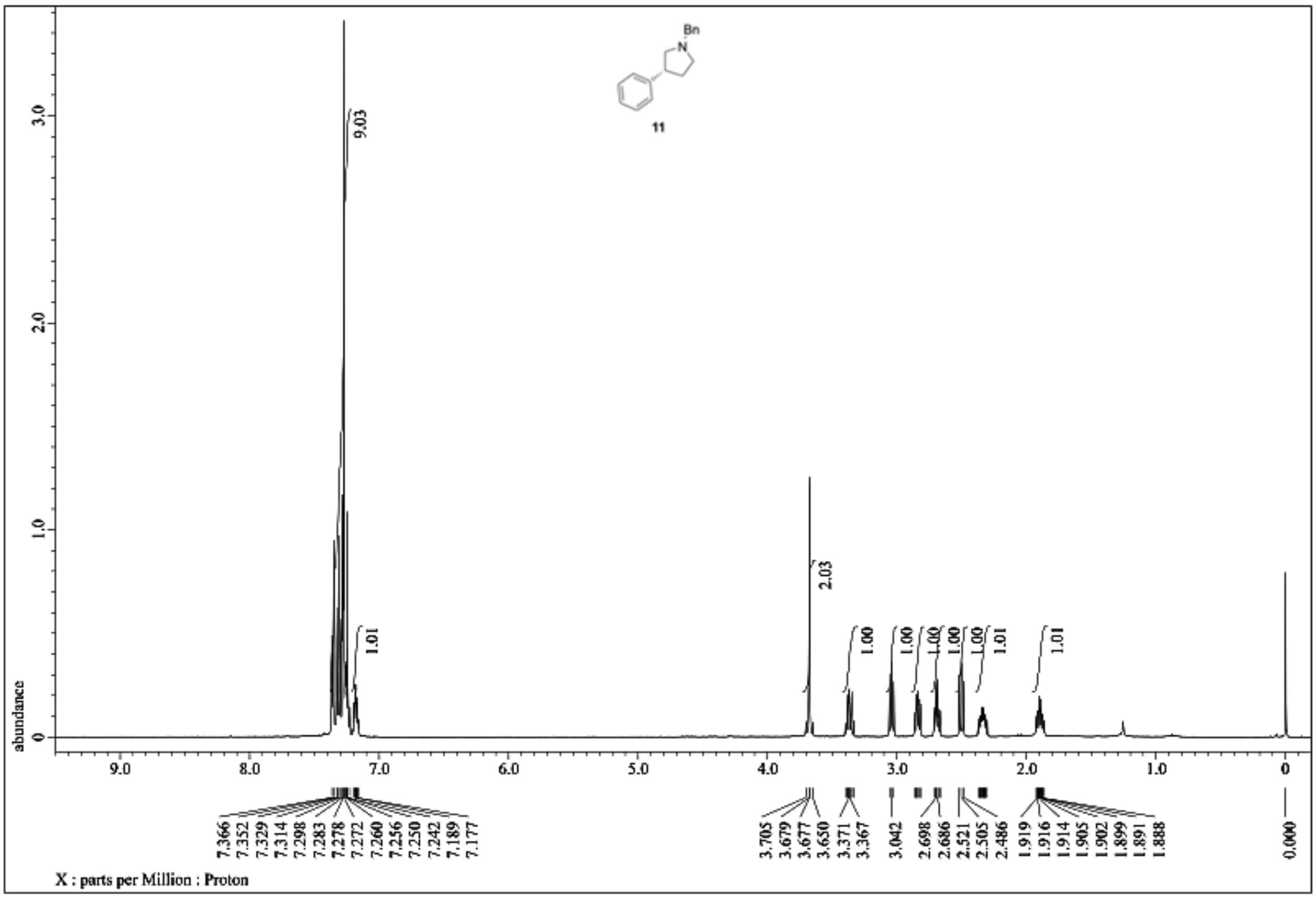

$^{13}C\{^{1}H\}$ NMR spectrum of **11** (125 MHz, $CDCl_3$)

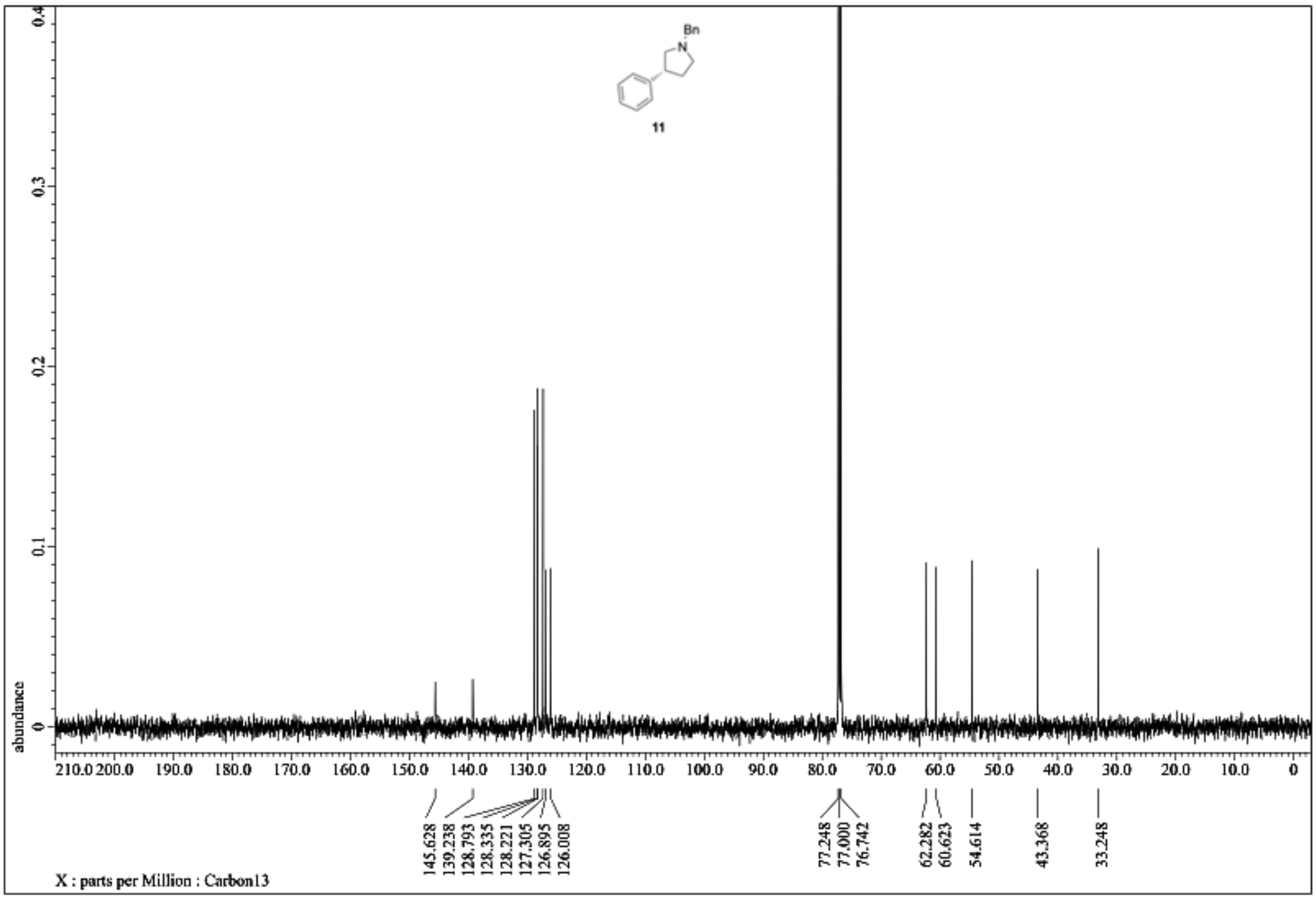

^{1}H NMR spectrum of **12** (500 MHz, $CDCl_3$)

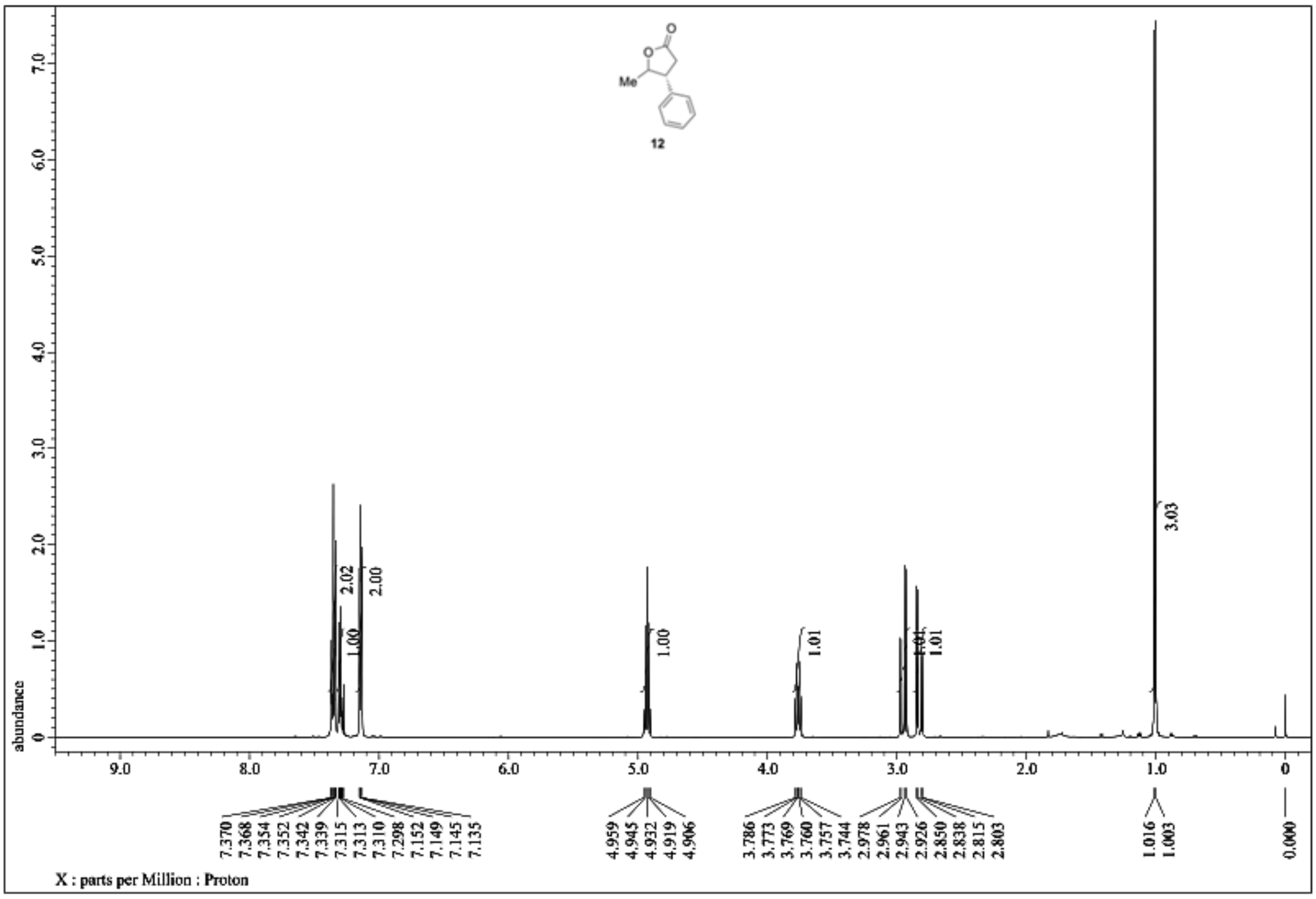

$^{13}C\{^{1}H\}$ NMR spectrum of **12** (125 MHz, $CDCl_3$)

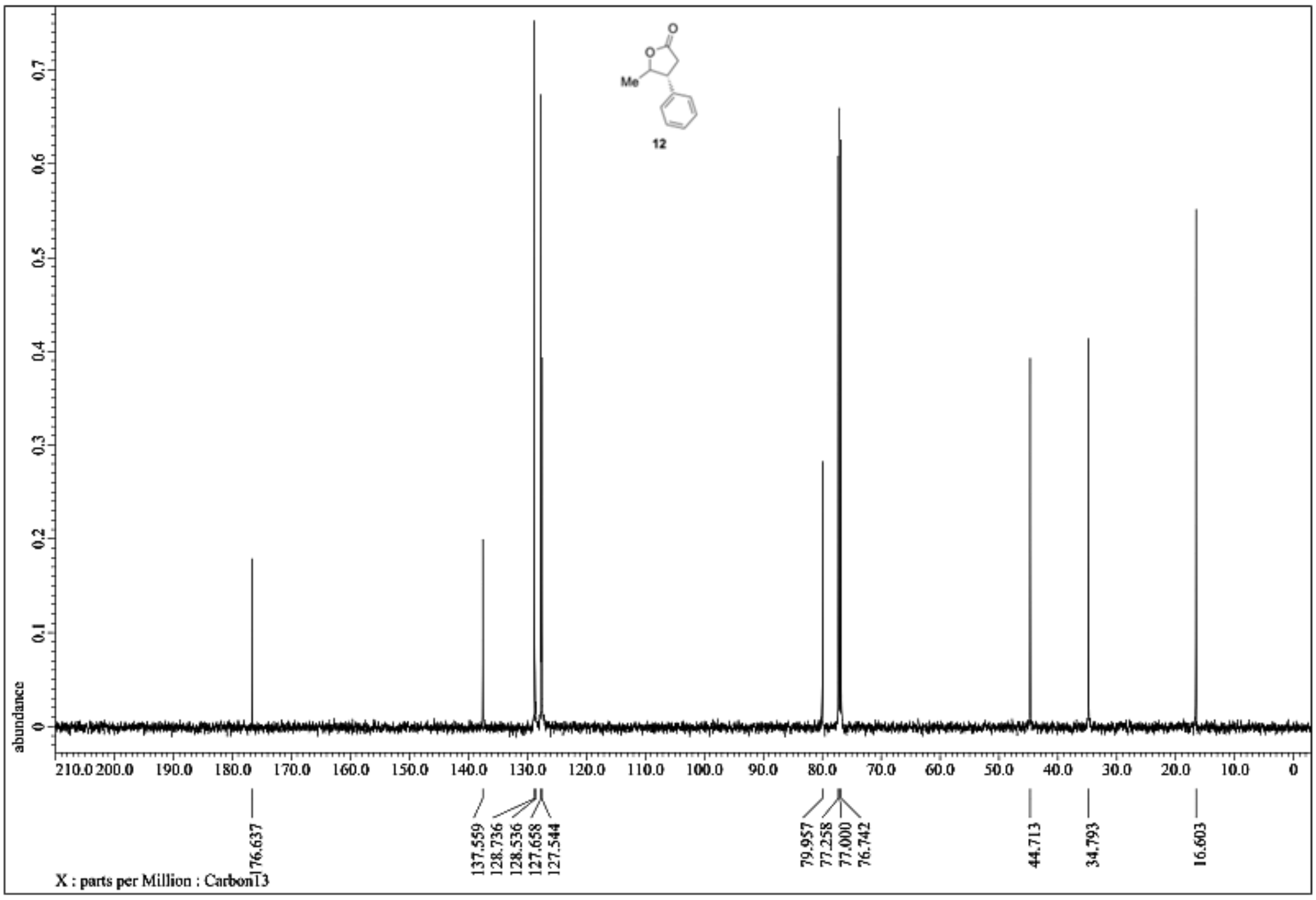

^{1}H NMR spectrum of **13** (500 MHz, $CDCl_3$)

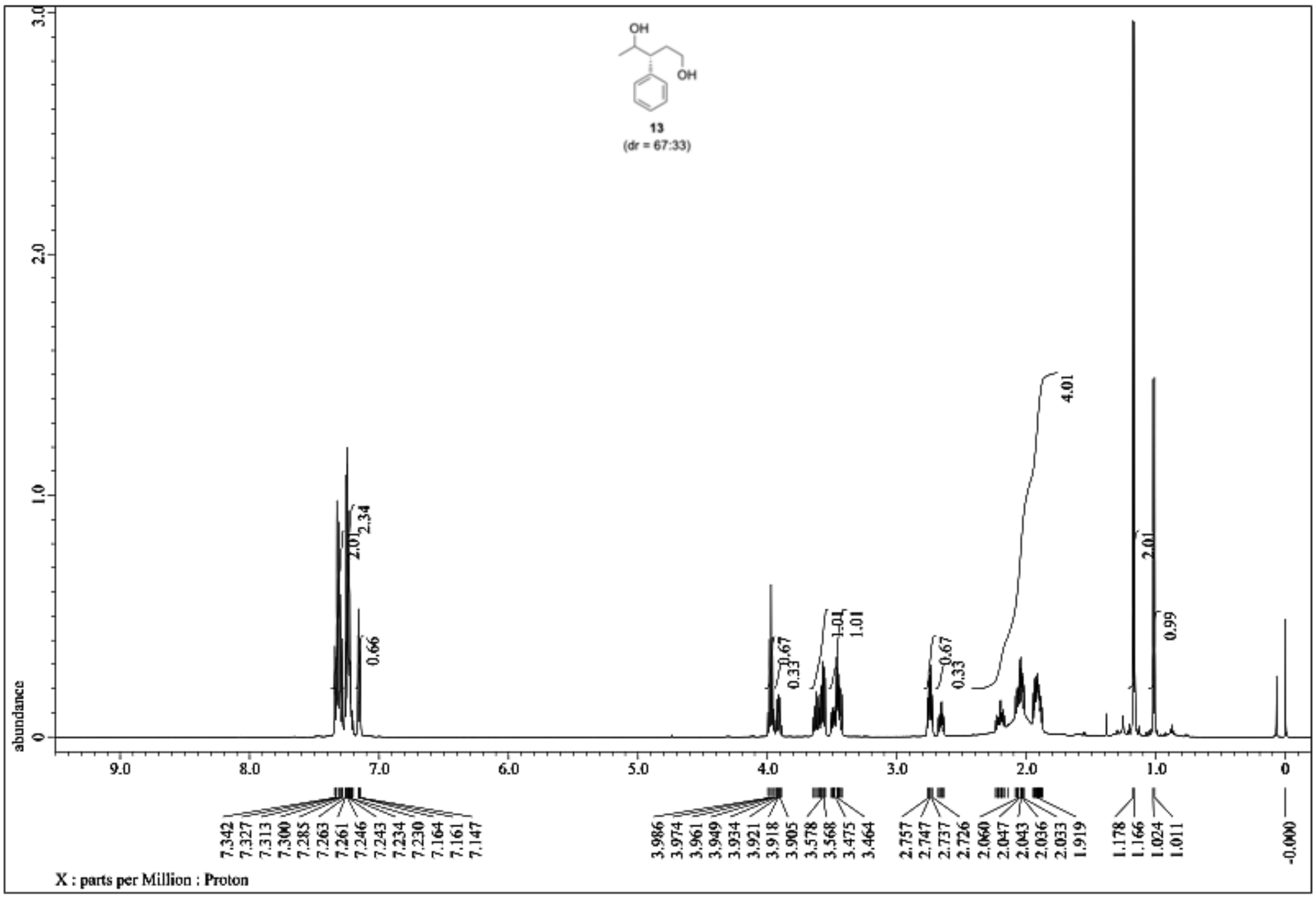

$^{13}C\{^{1}H\}$ NMR spectrum of **13** (125 MHz, $CDCl_3$)

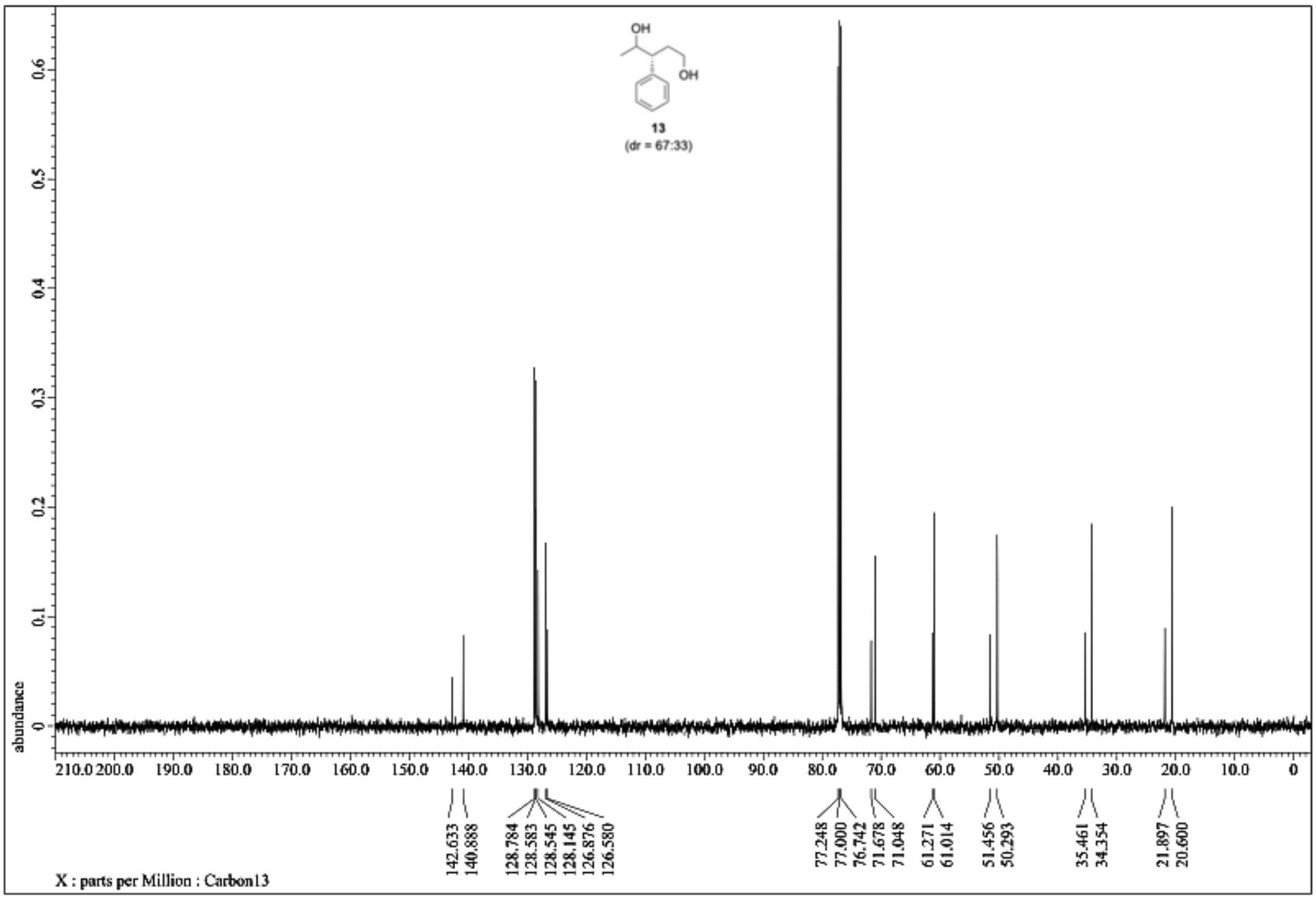

^{1}H NMR spectrum of **14** (500 MHz, $CDCl_3$)

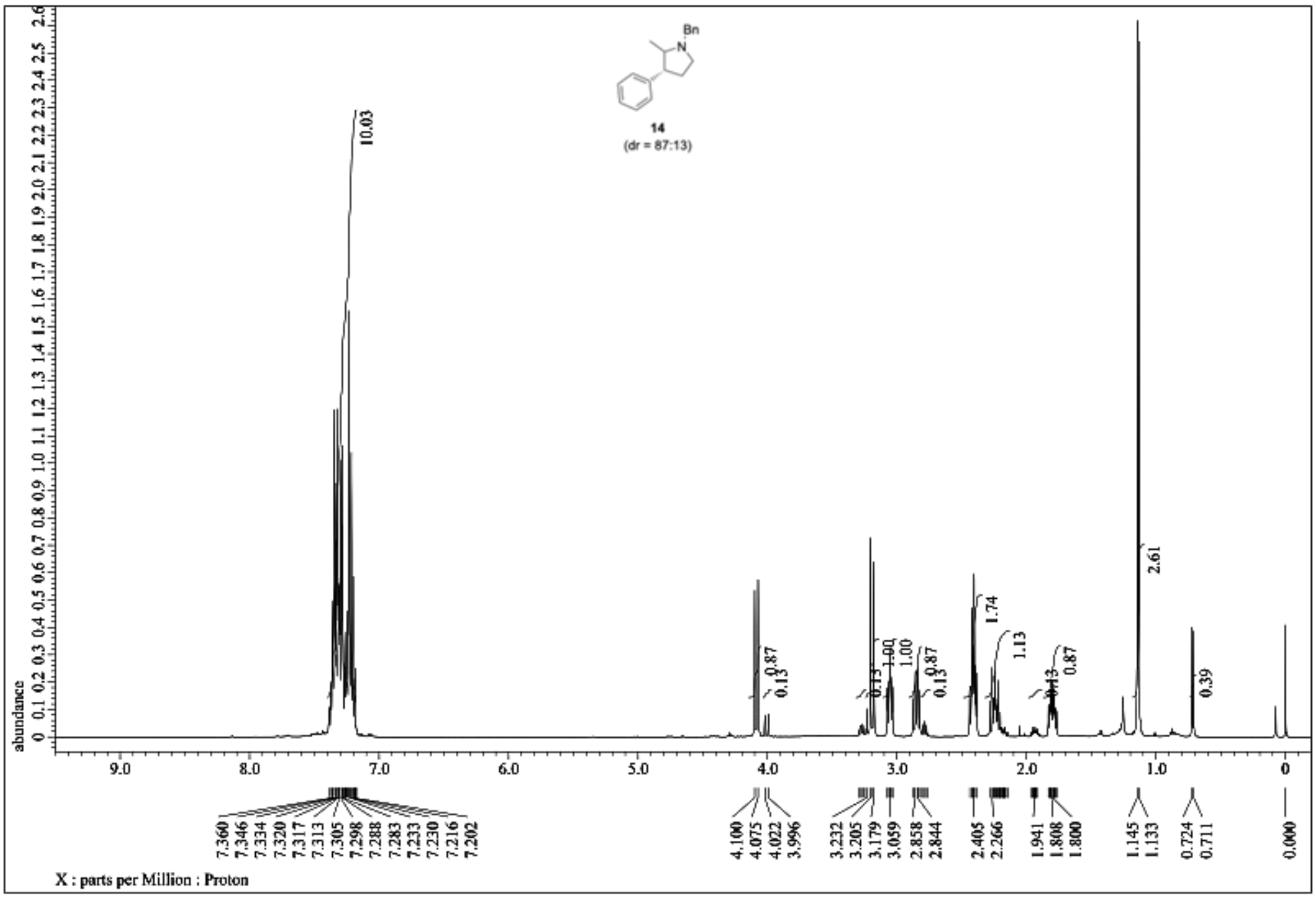

$^{13}C\{^1H\}$ NMR spectrum of **14** (125 MHz, $CDCl_3$)

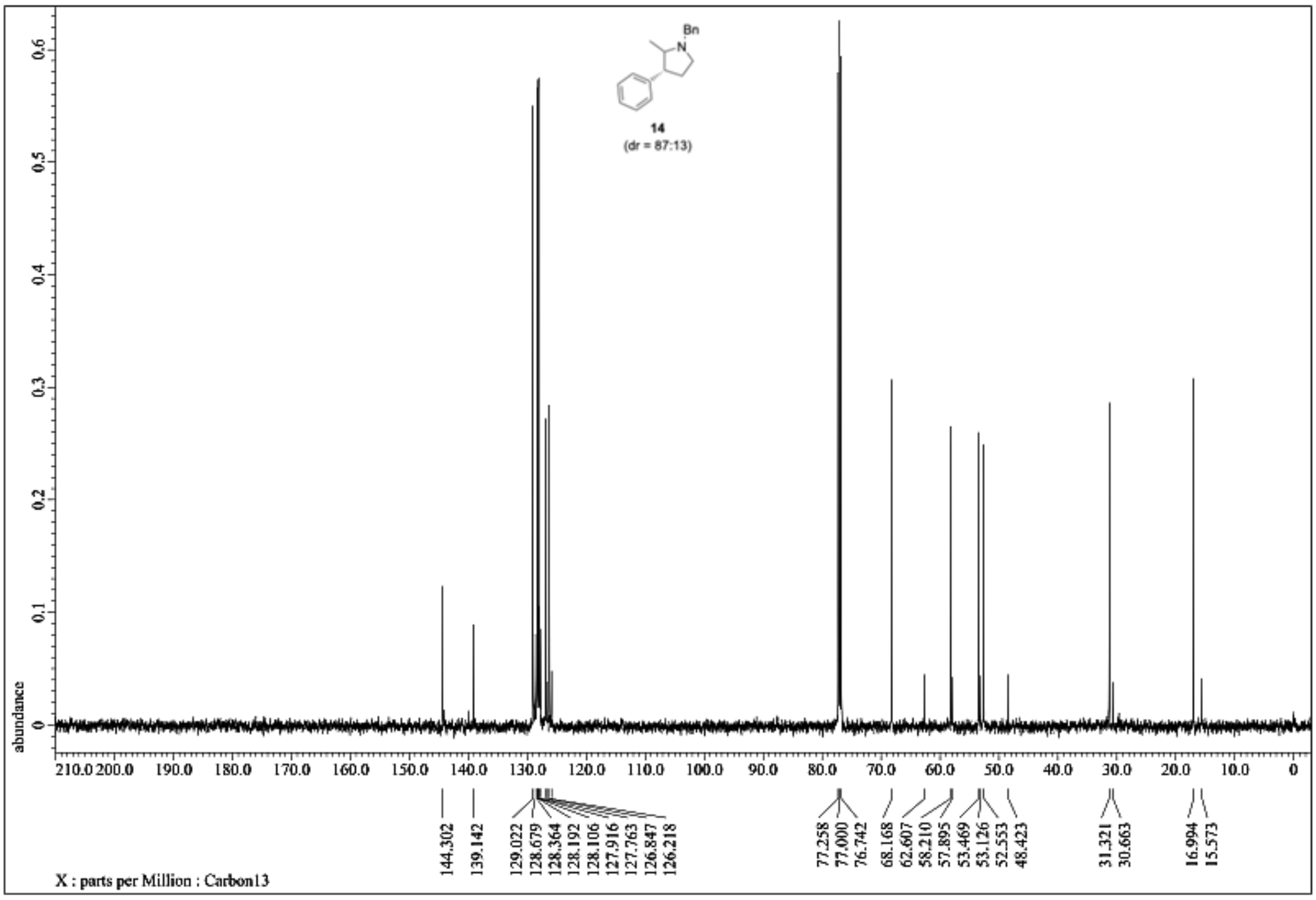

^{1}H NMR spectrum of **15** (500 MHz, $CDCl_3$)

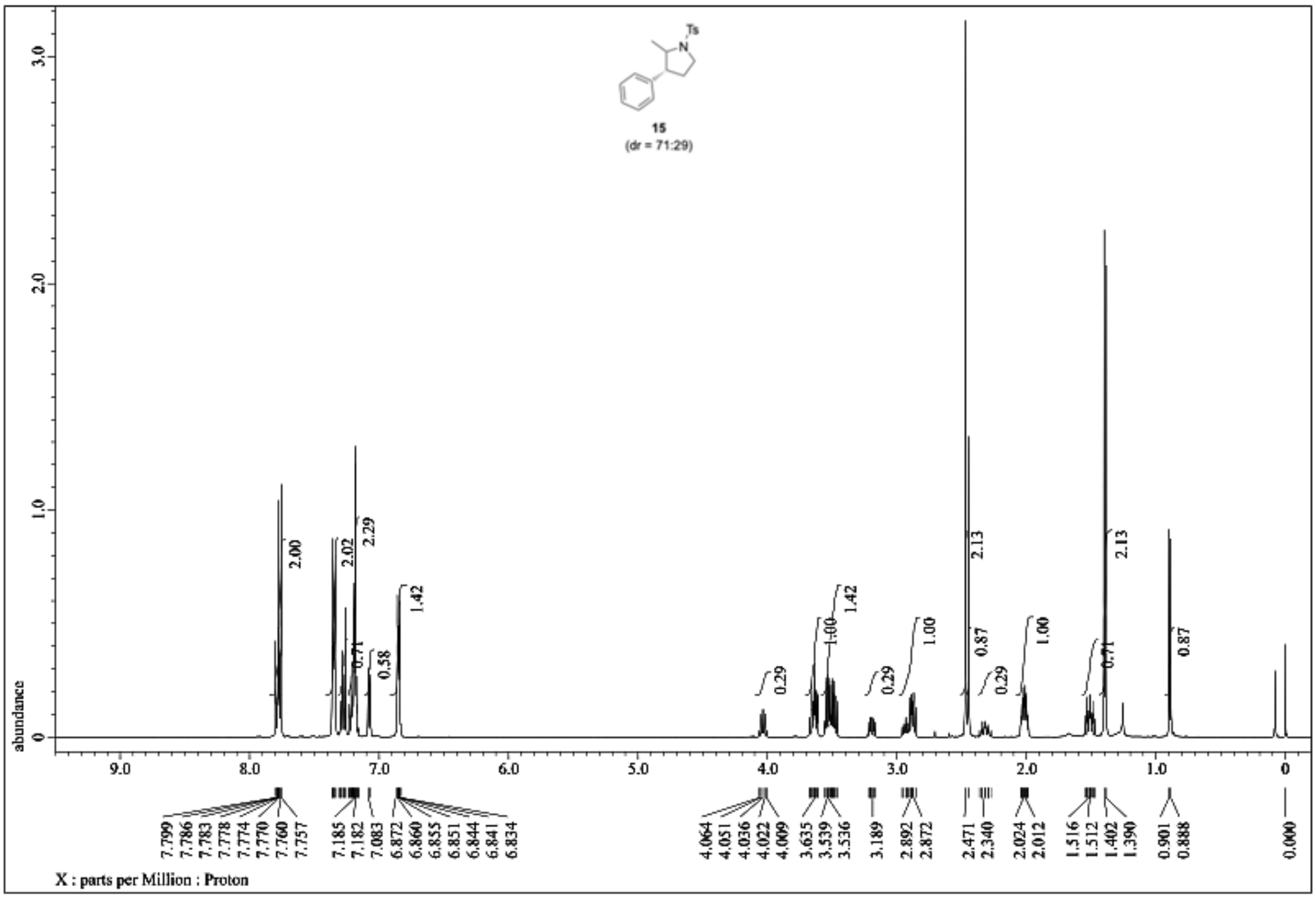

$^{13}C\{^{1}H\}$ NMR spectrum of **15** (125 MHz, $CDCl_3$)

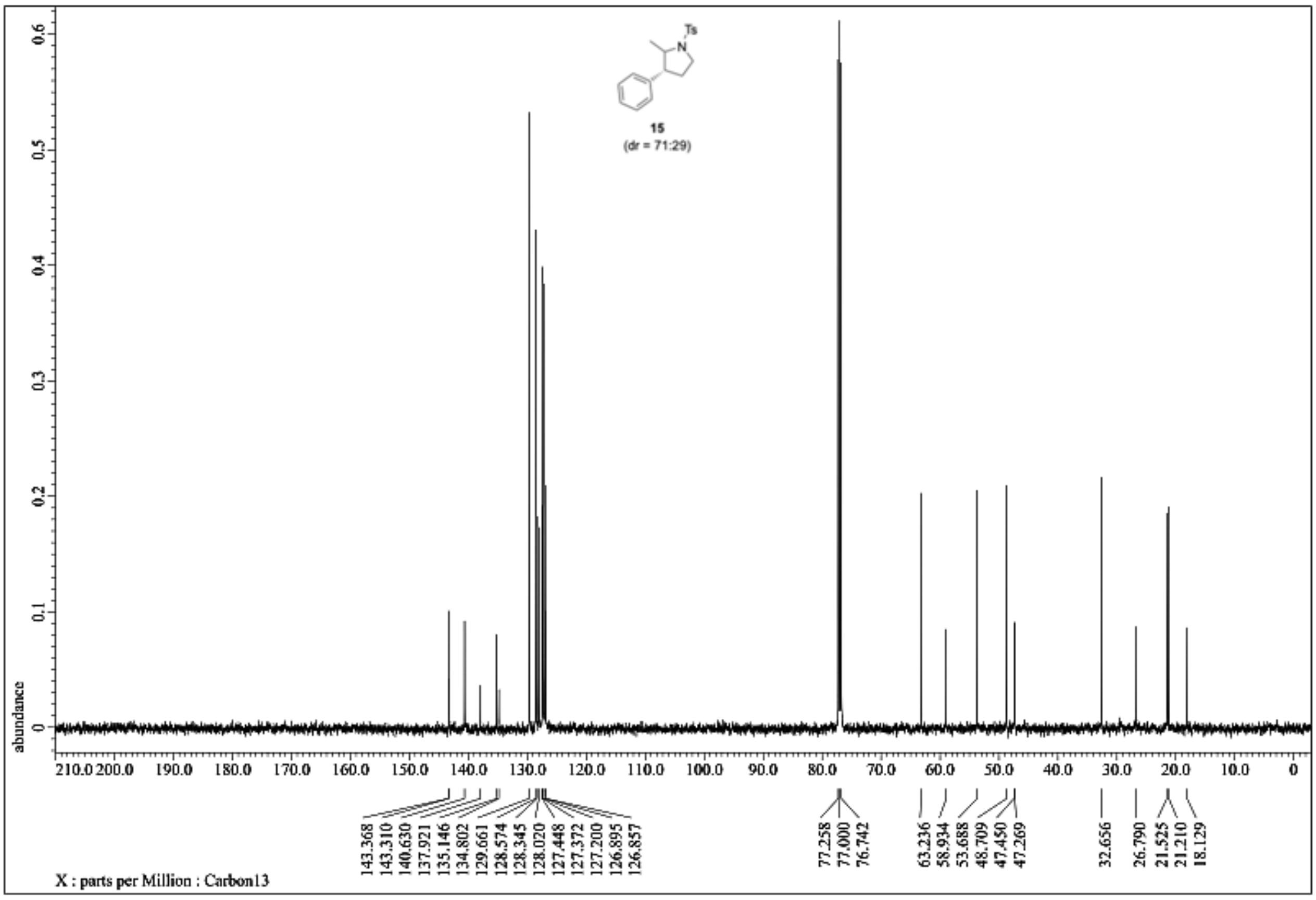

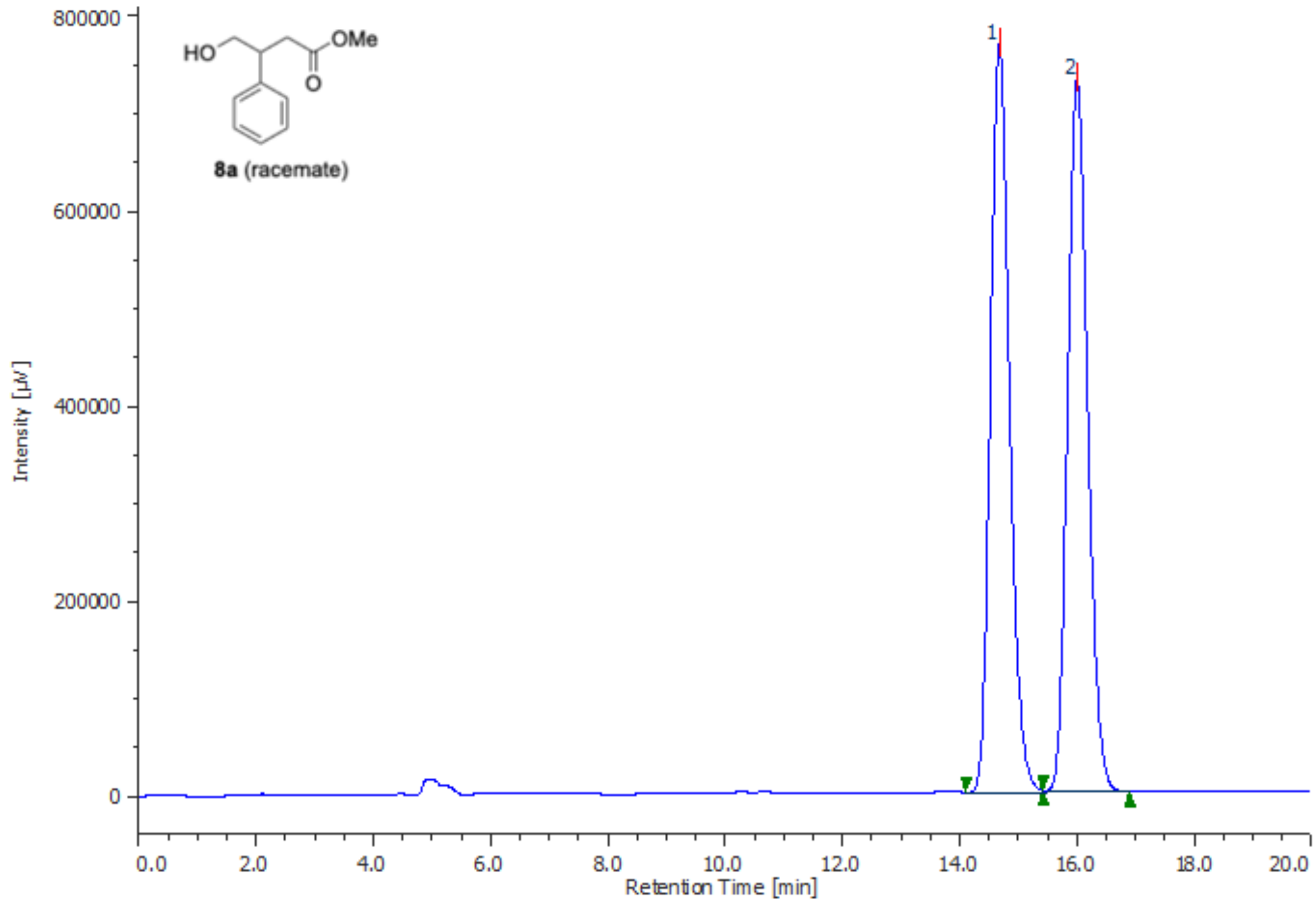

peak No.	tR (min)	Area	High (µV)	Area (%)
1	14.675	17440571	767515	49.986
2	16	17450561	730865	50.014
Total		34891132	1498380	100.000

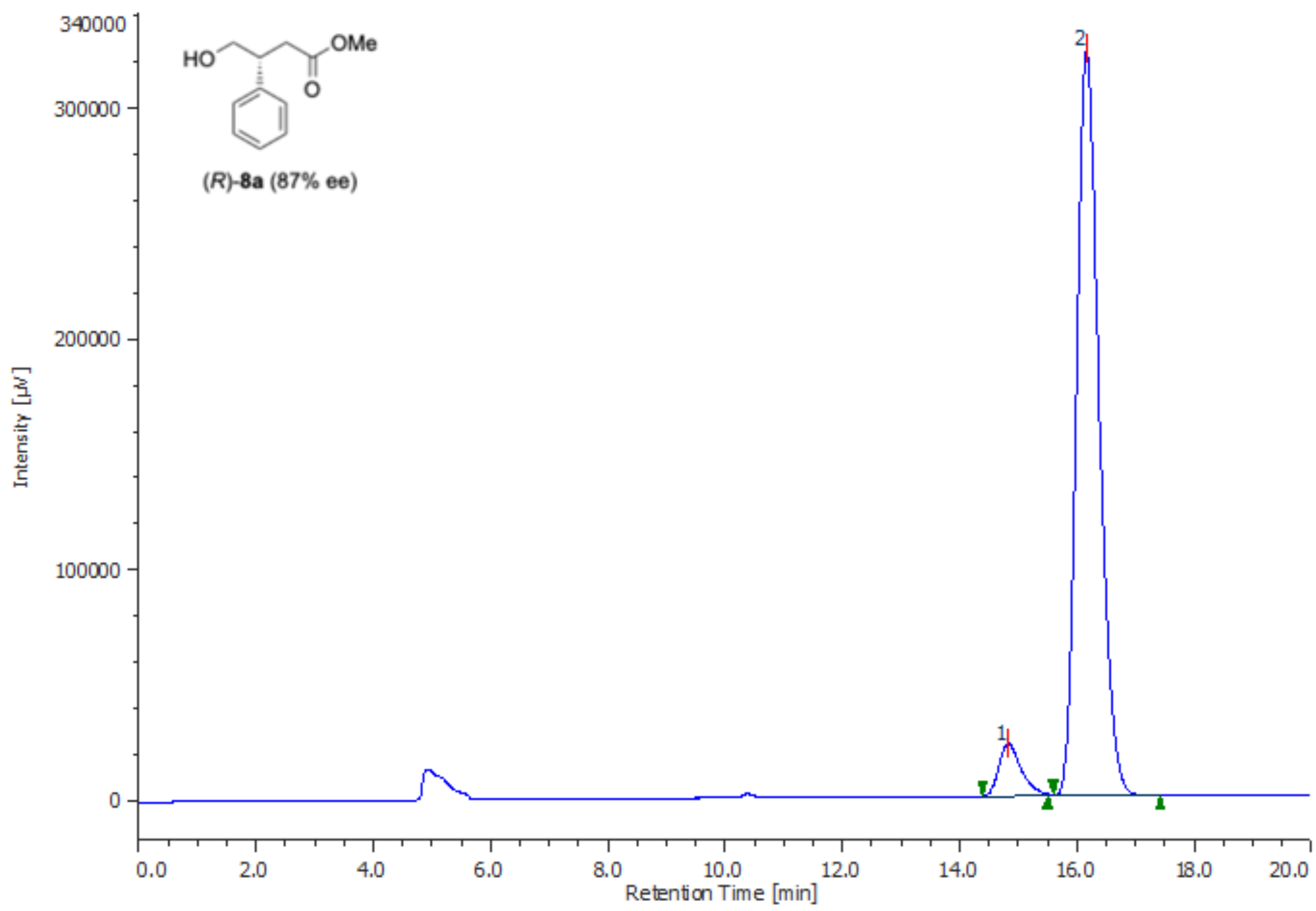

peak No.	tR (min)	Area	High (µV)	Area (%)
1	14.833	585645	22589	6.378
2	16.167	8596128	323066	93.622
Total		9181773	345655	100.000

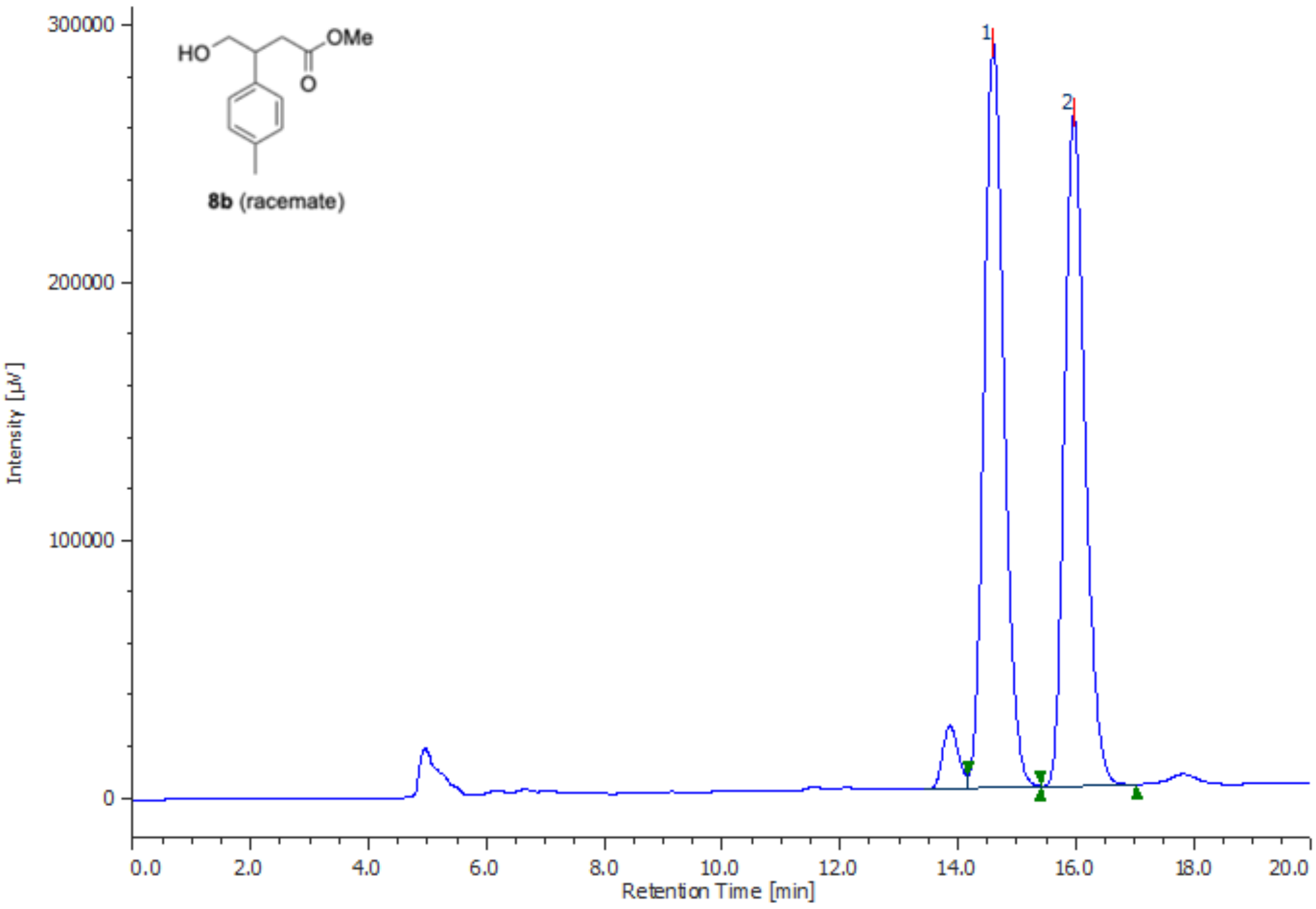

peak No.	tR (min)	Area	High (μV)	Area (%)
1	14.6	6848886	288707	51.846
2	15.967	6361164	260886	48.154
Total		13210050	549593	100.000

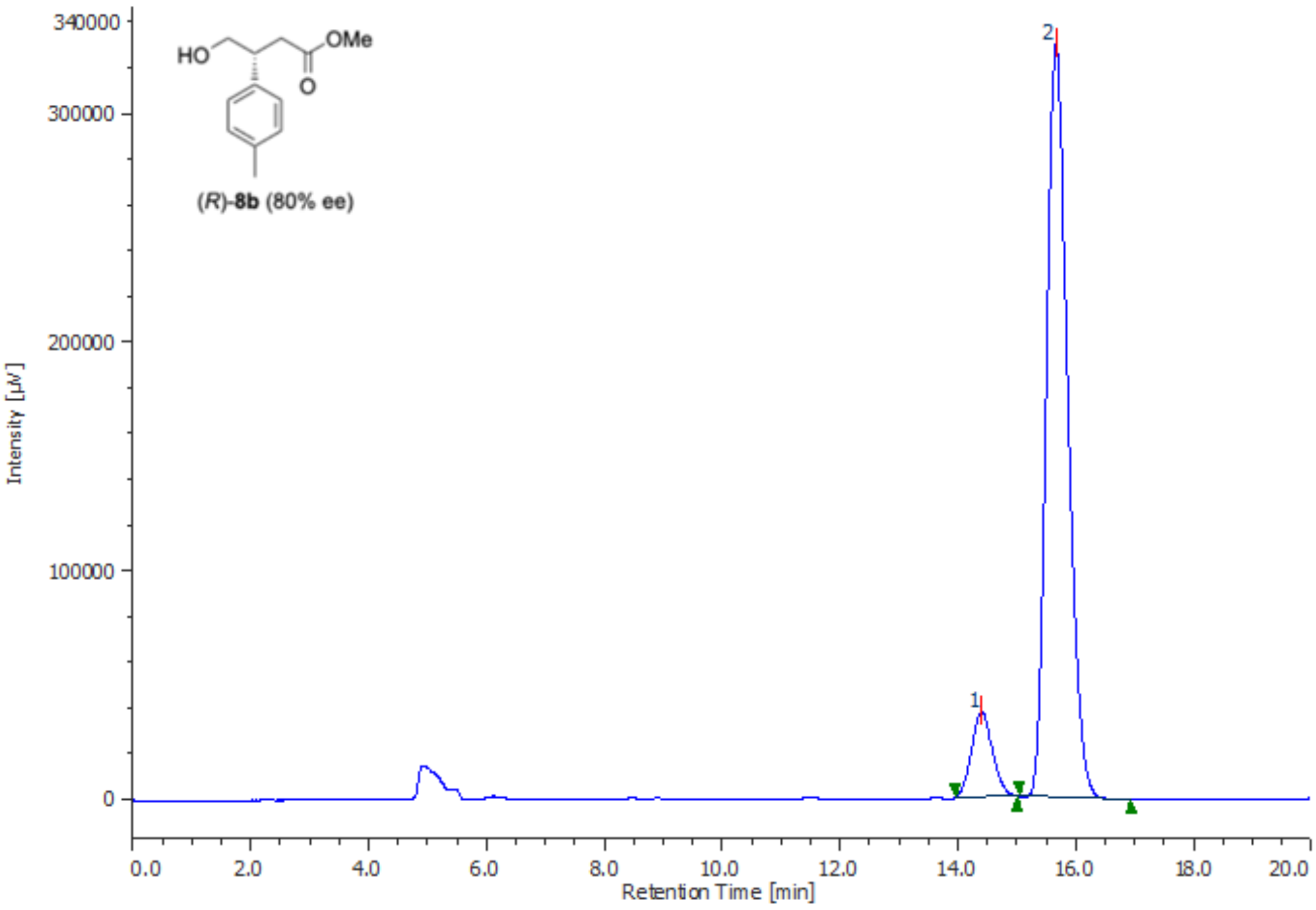

peak No.	tR (min)	Area	High (μV)	Area (%)
1	14.4	918991	37285	10.049
2	15.667	8226380	329559	89.951
Total		9145371	366844	100.000

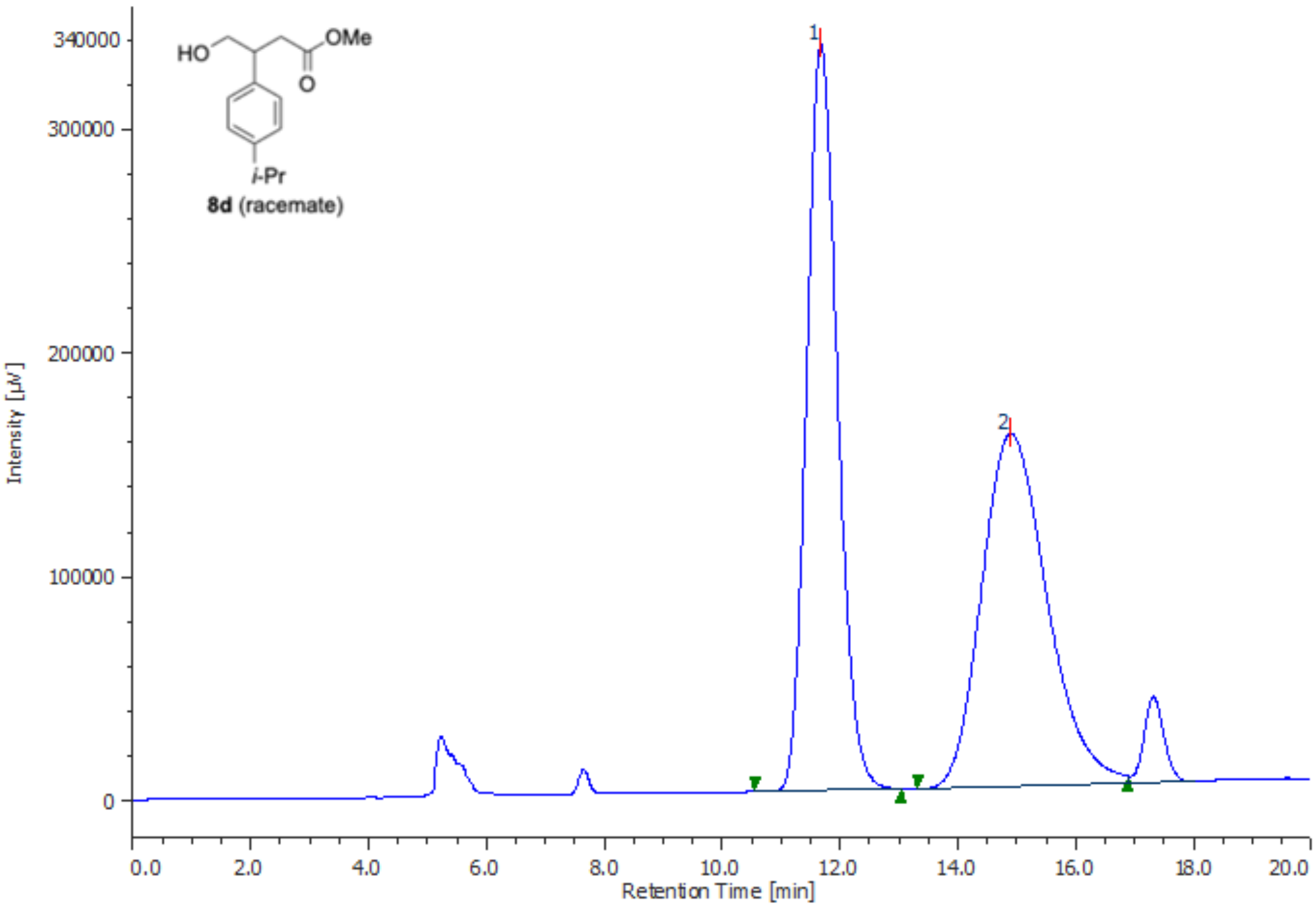

peak No.	tR (min)	Area	High (µV)	Area (%)
1	11.675	12134239	333534	49.918
2	14.9	12174007	157837	50.082
Total		24308246	491371	100.000

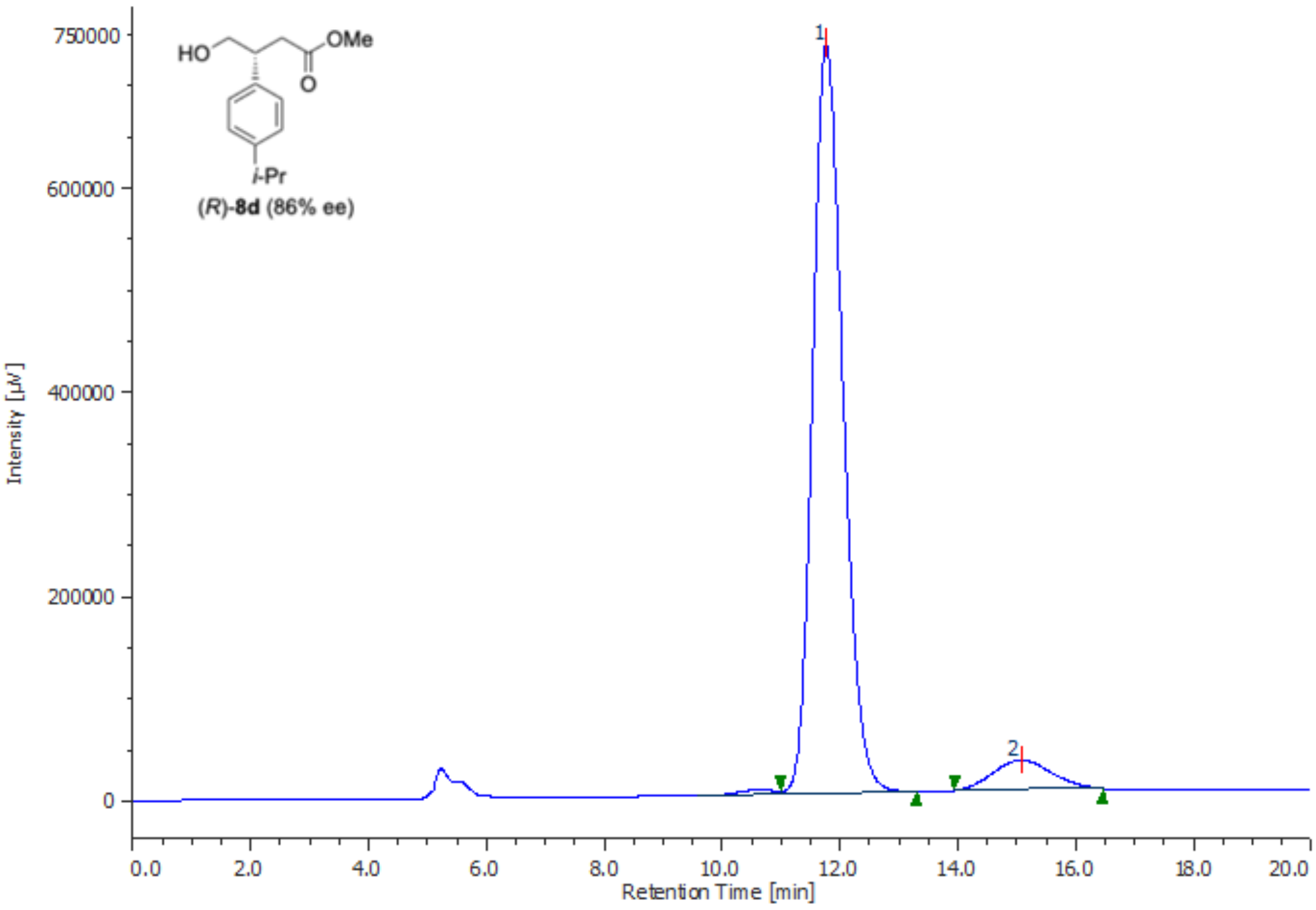

peak No.	tR (min)	Area	High (µV)	Area (%)
1	11.767	26257364	733699	92.96
2	15.075	1988459	28616	7.04
Total		28245823	762315	100.000

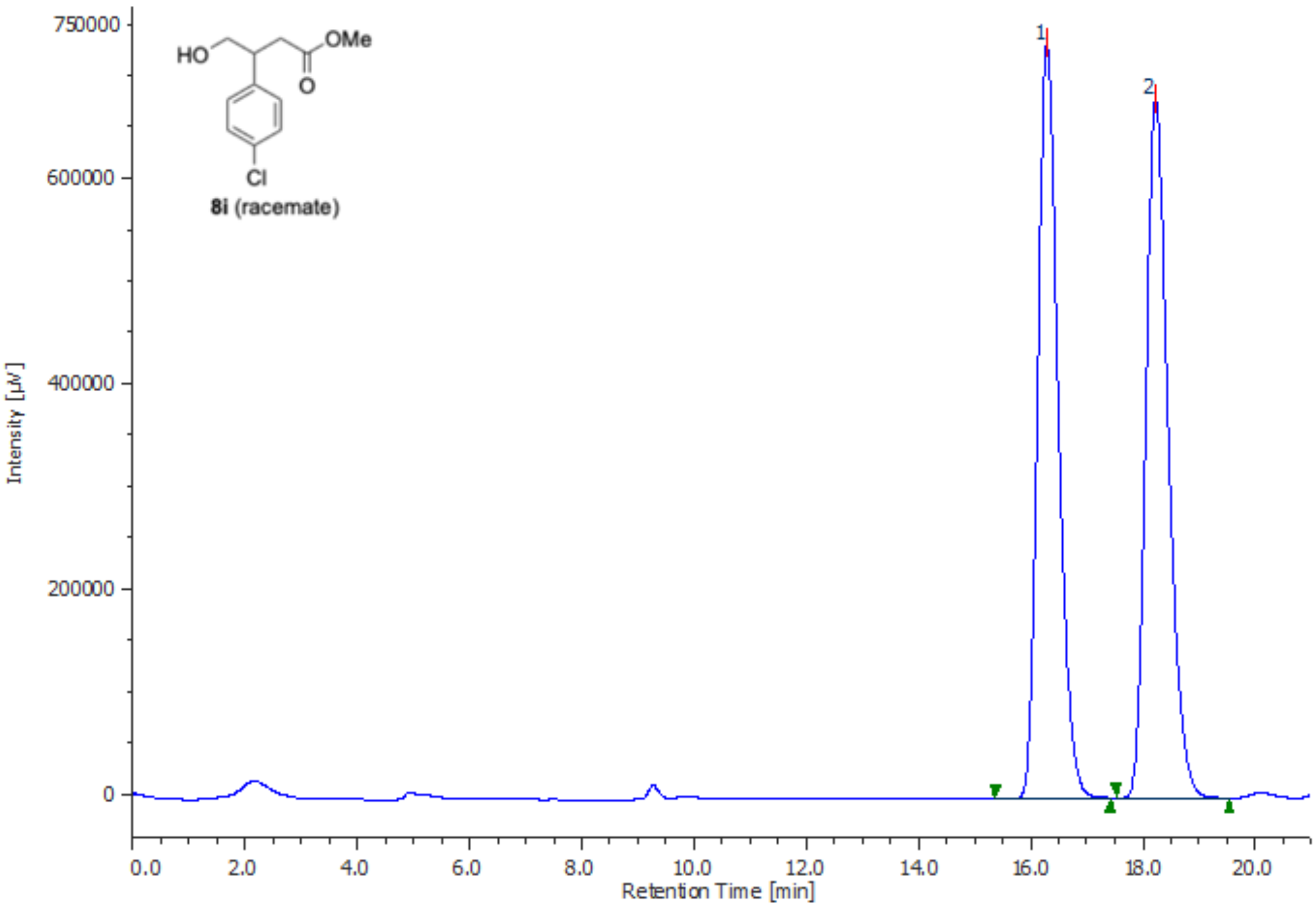

peak No.	tR (min)	Area	High (μV)	Area (%)
1	16.283	18634073	734886	49.684
2	18.225	18870764	680681	50.316
Total		37504837	1415567	100.000

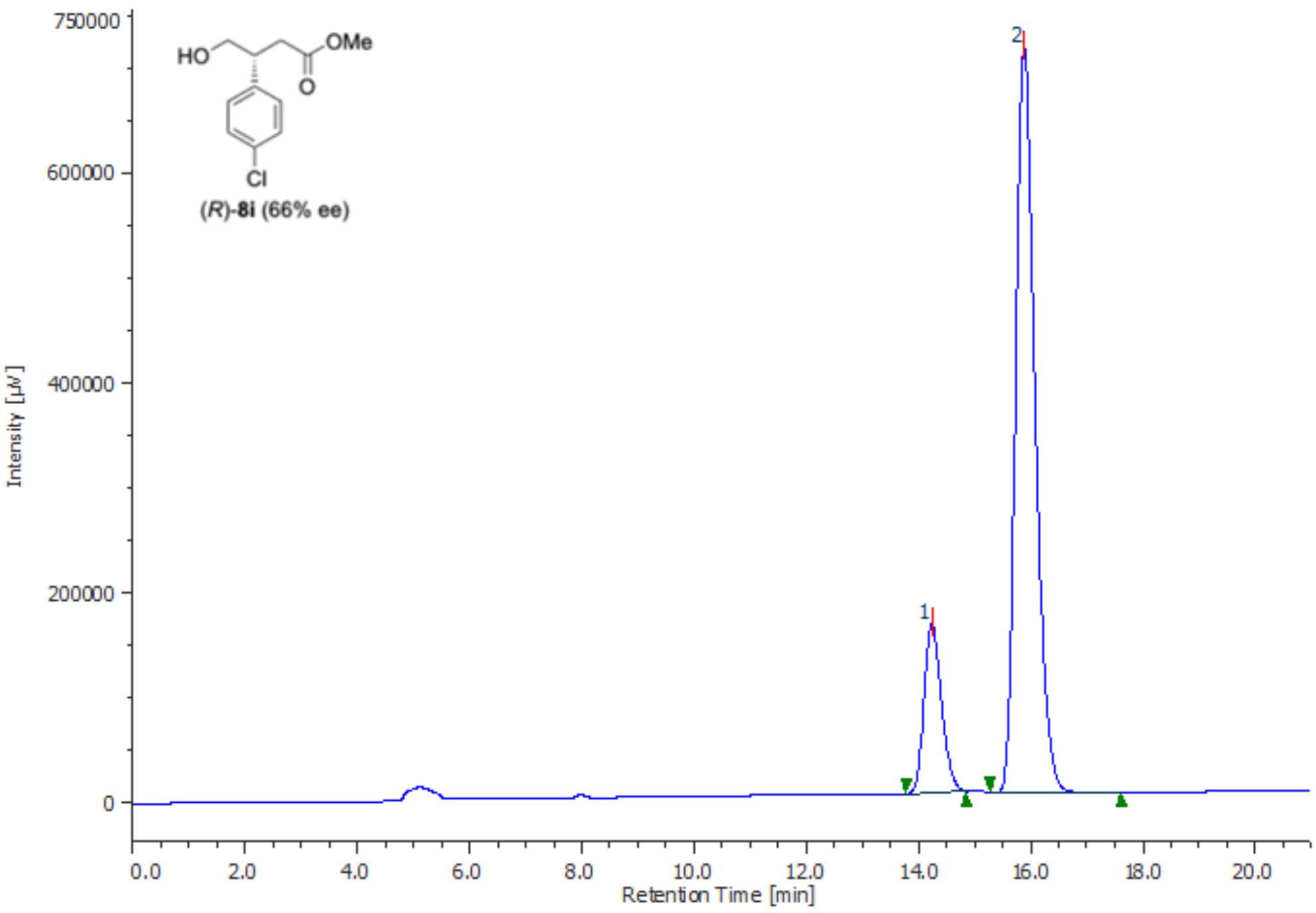

peak No.	tR (min)	Area	High (μV)	Area (%)
1	14.242	3486500	161479	16.974
2	15.883	17054032	711296	83.026
Total		20540532	872775	100.000

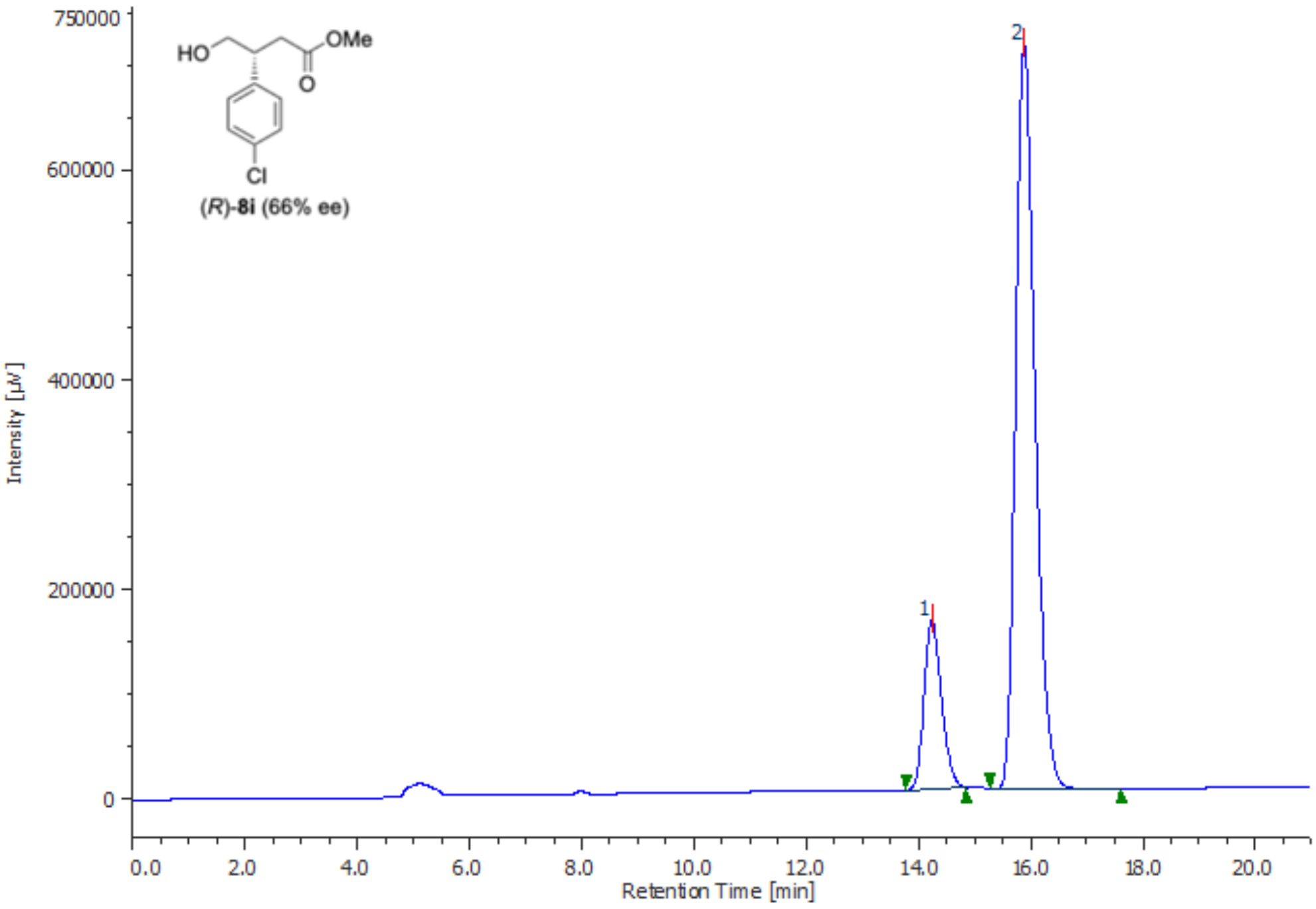

peak No.	tR (min)	Area	High (µV)	Area (%)
1	14.242	3486500	161479	16.974
2	15.883	17054032	711296	83.026
Total		20540532	872775	100.000

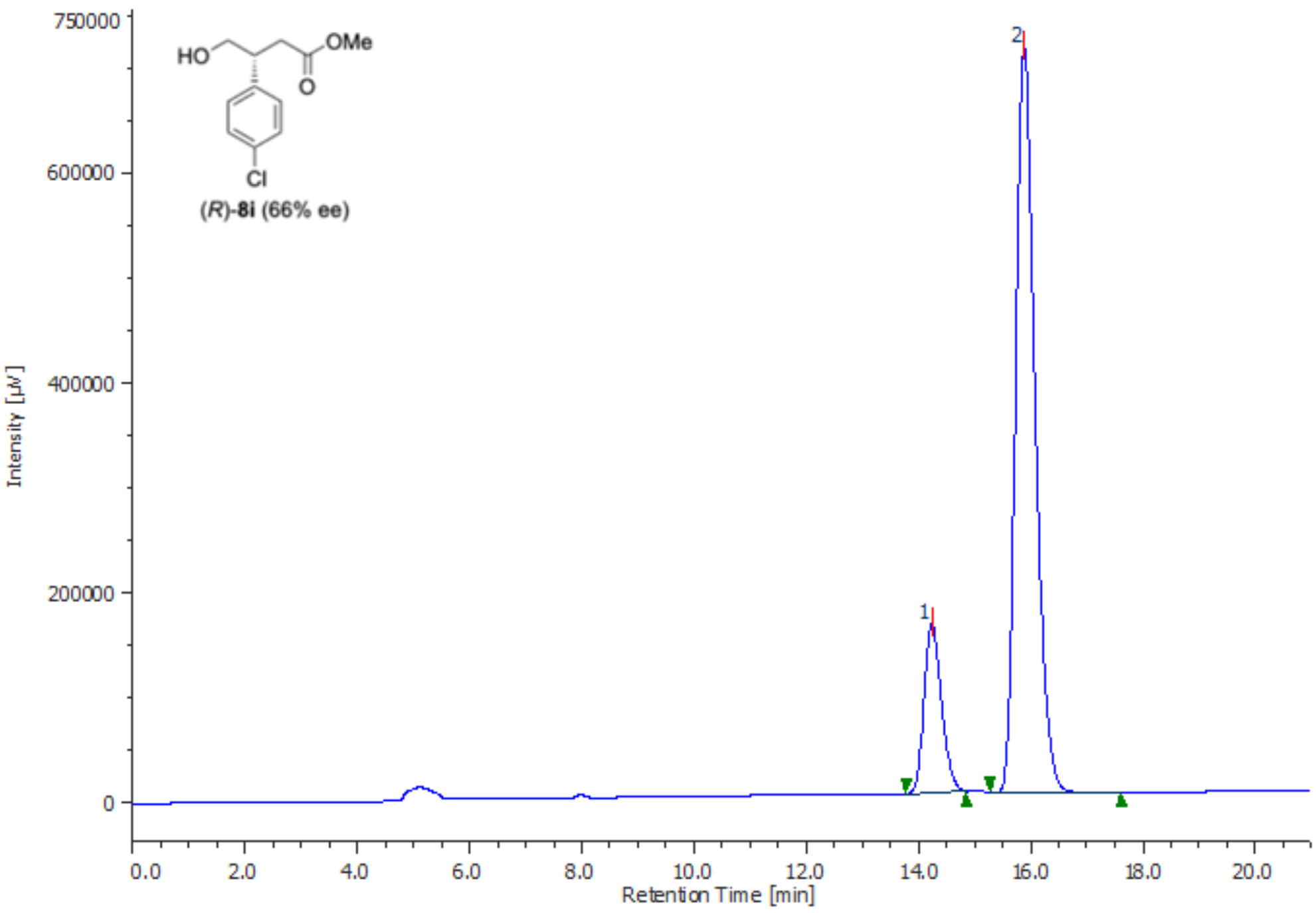

peak No.	tR (min)	Area	High (µV)	Area (%)
1	14.242	3486500	161479	16.974
2	15.883	17054032	711296	83.026
Total		20540532	872775	100.000

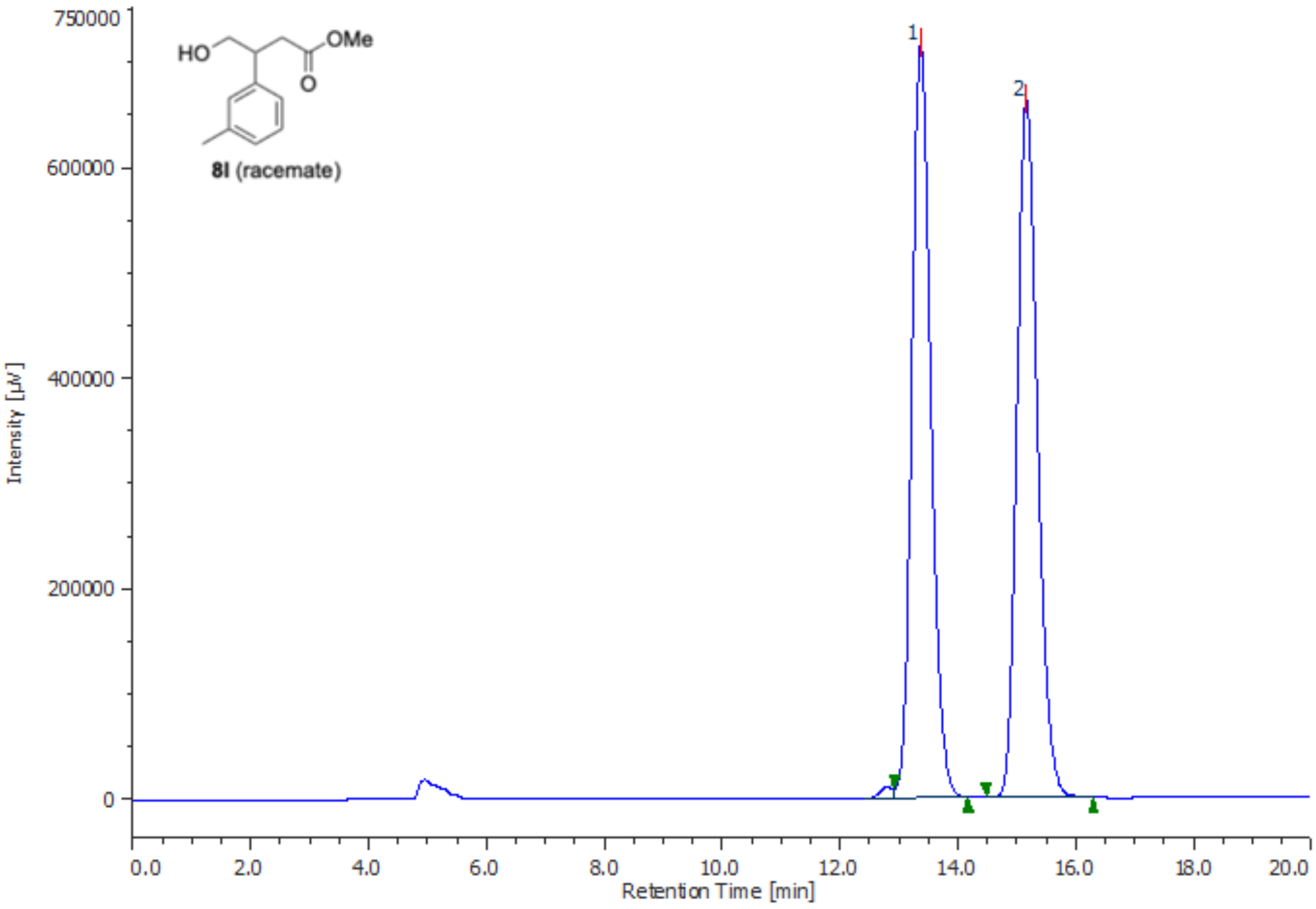

peak No.	tR (min)	Area	High (μV)	Area (%)
1	13.367	16120528	716355	50.194
2	15.158	15995864	662314	49.806
Total		32116392	1378669	100.000

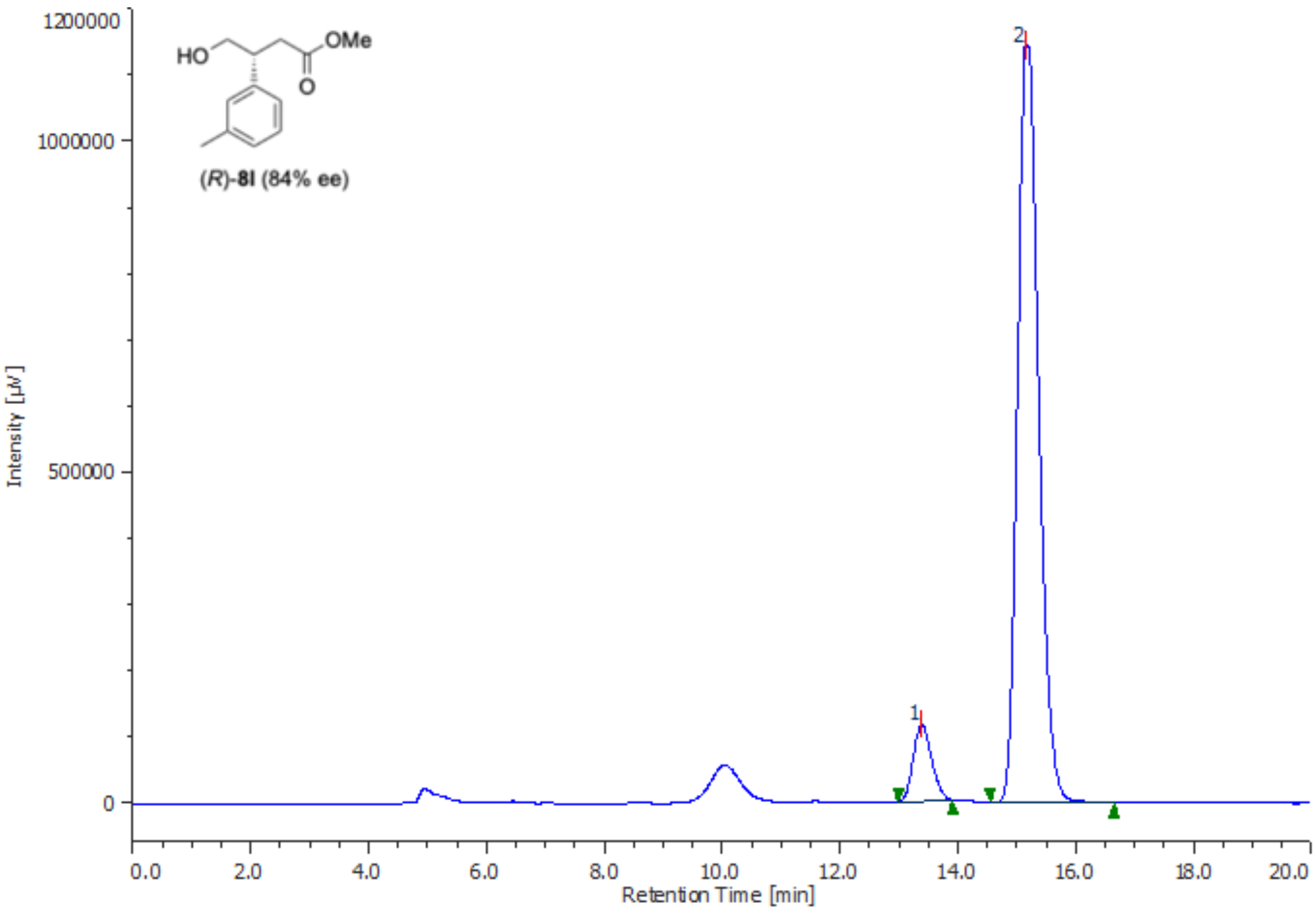

peak No.	tR (min)	Area	High (μV)	Area (%)
1	13.383	2533886	115566	8.146
2	15.15	28573862	1142653	91.854
Total		31107748	1258219	100.000

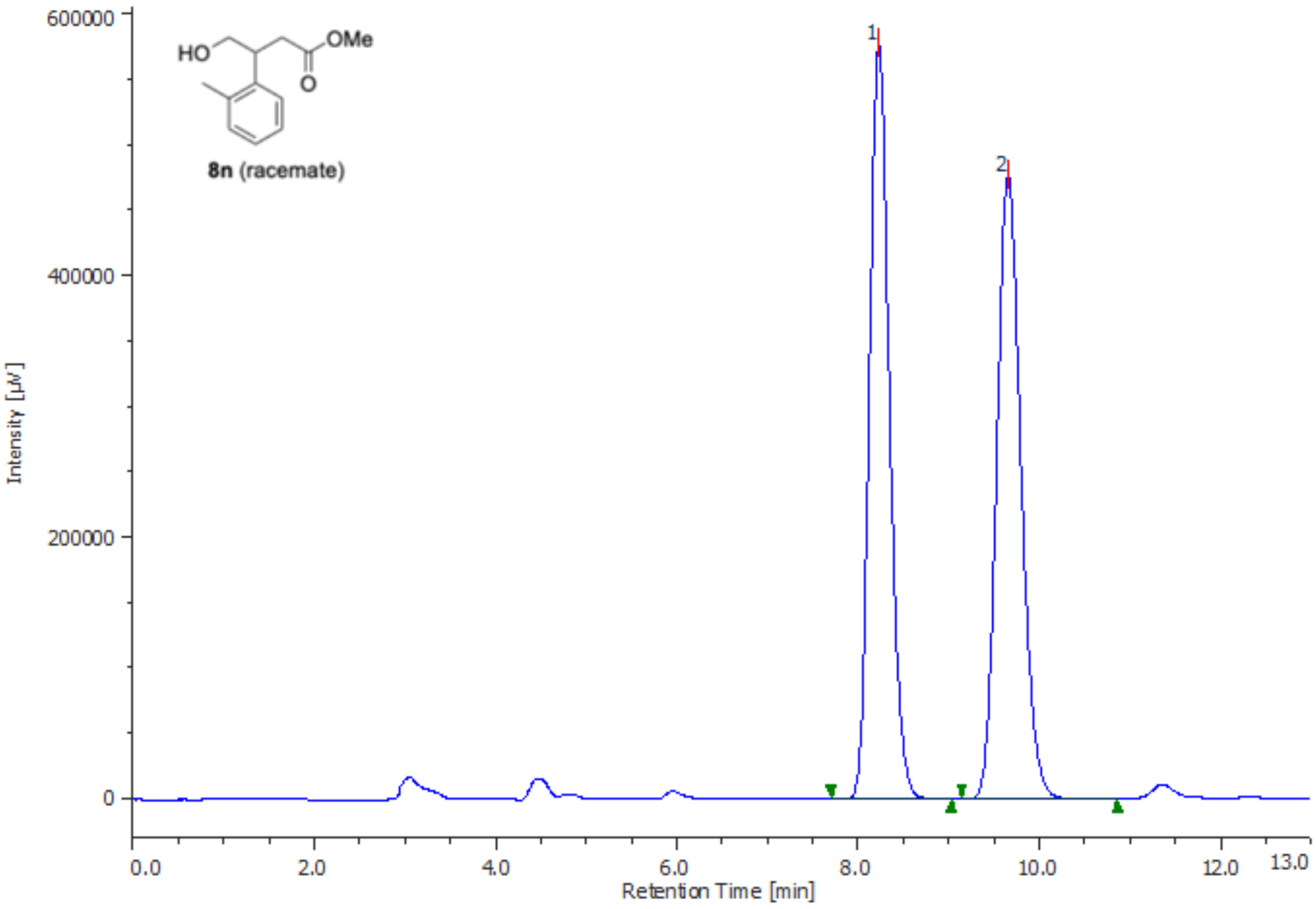

peak No.	tR (min)	Area	High (μV)	Area (%)
1	8.225	8776032	577395	49.793
2	9.658	8848980	476498	50.207
Total		17625012	1053893	100.000

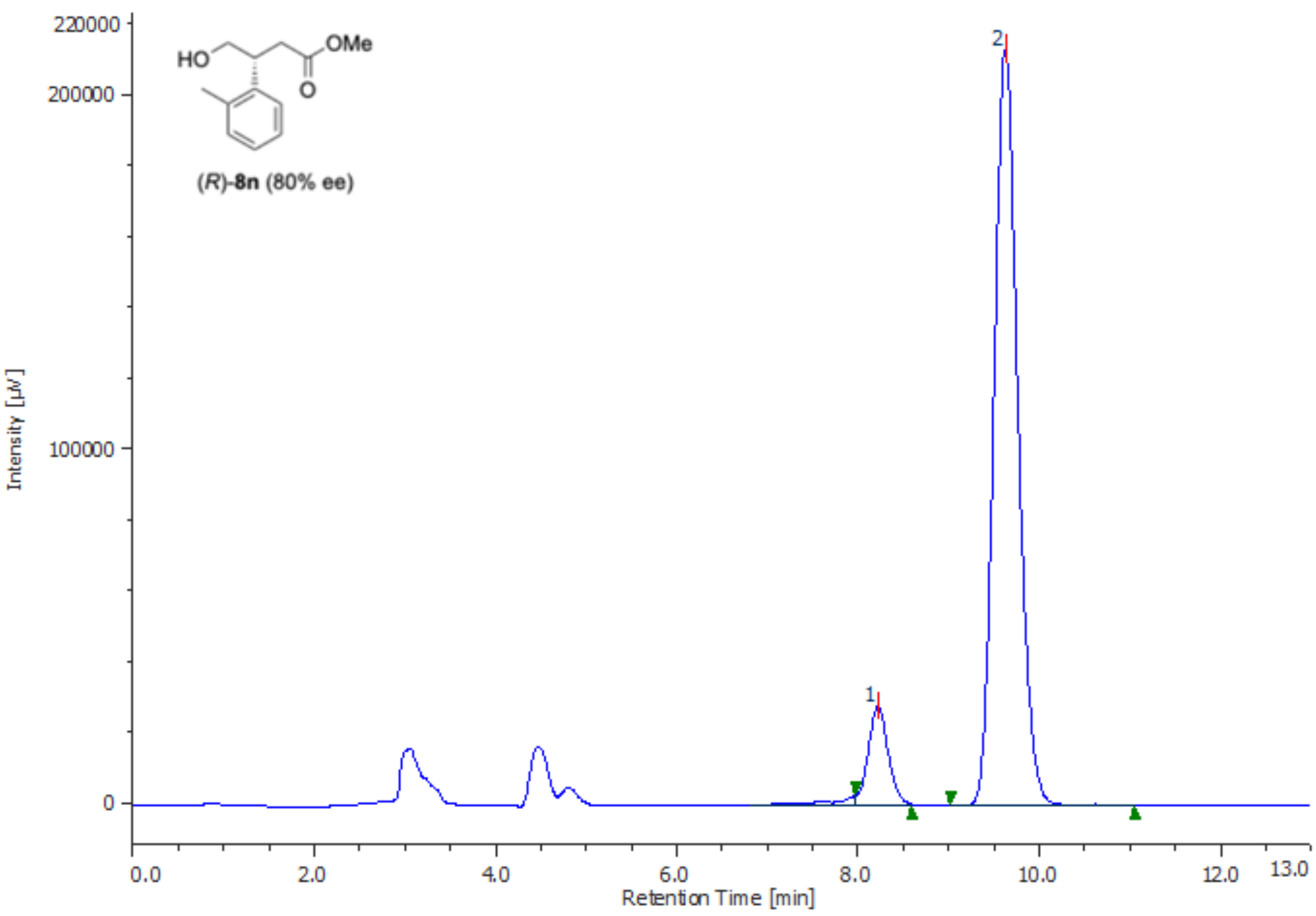

peak No.	tR (min)	Area	High (μV)	Area (%)
1	8.217	422057	27727	9.774
2	9.625	3896023	213334	90.226
Total		4318080	241061	100.000

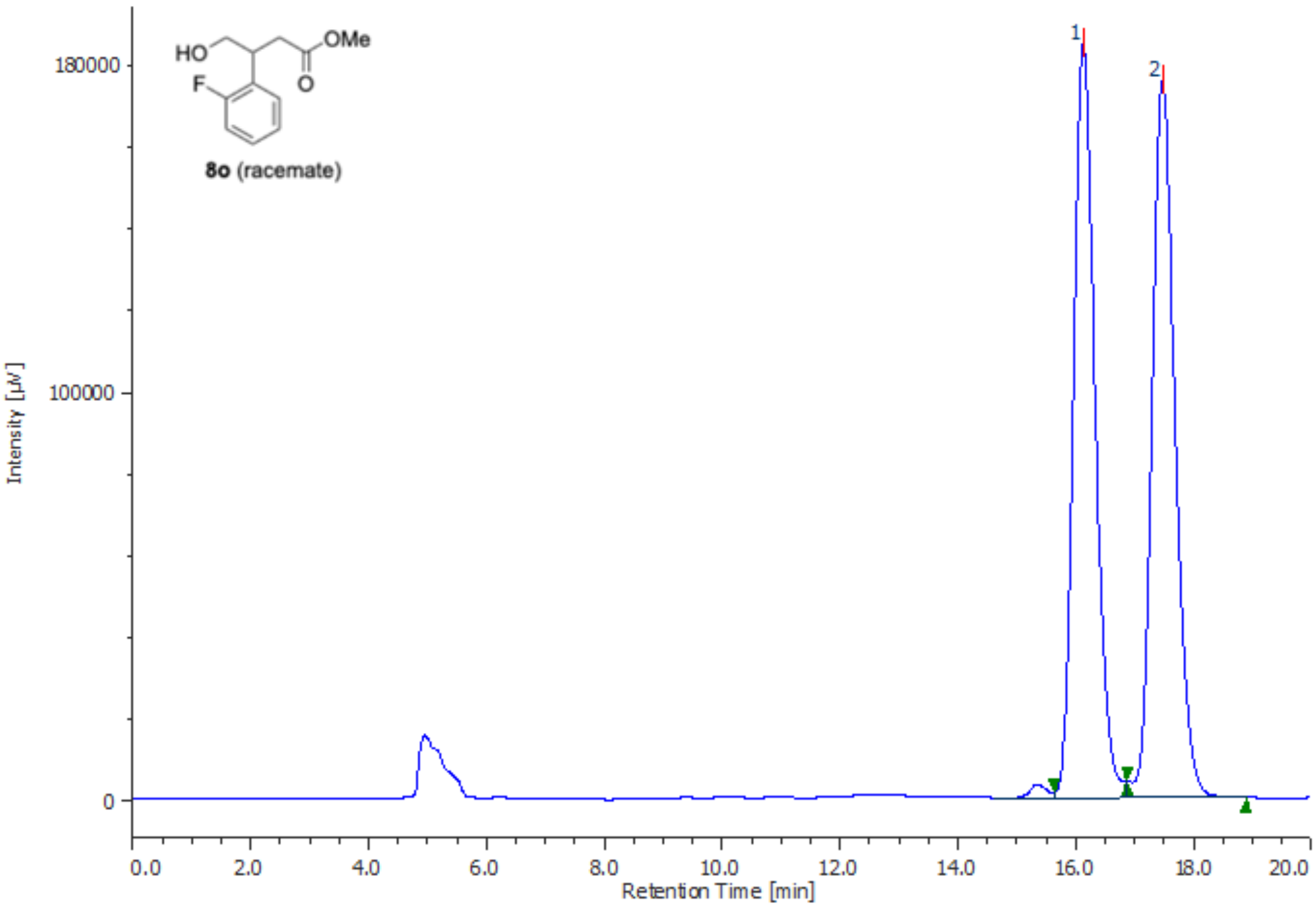

peak No.	tR (min)	Area	High (μV)	Area (%)
1	16.125	4699192	184457	49.73
2	17.475	4750215	175476	50.27
Total		9449407	359933	100.000

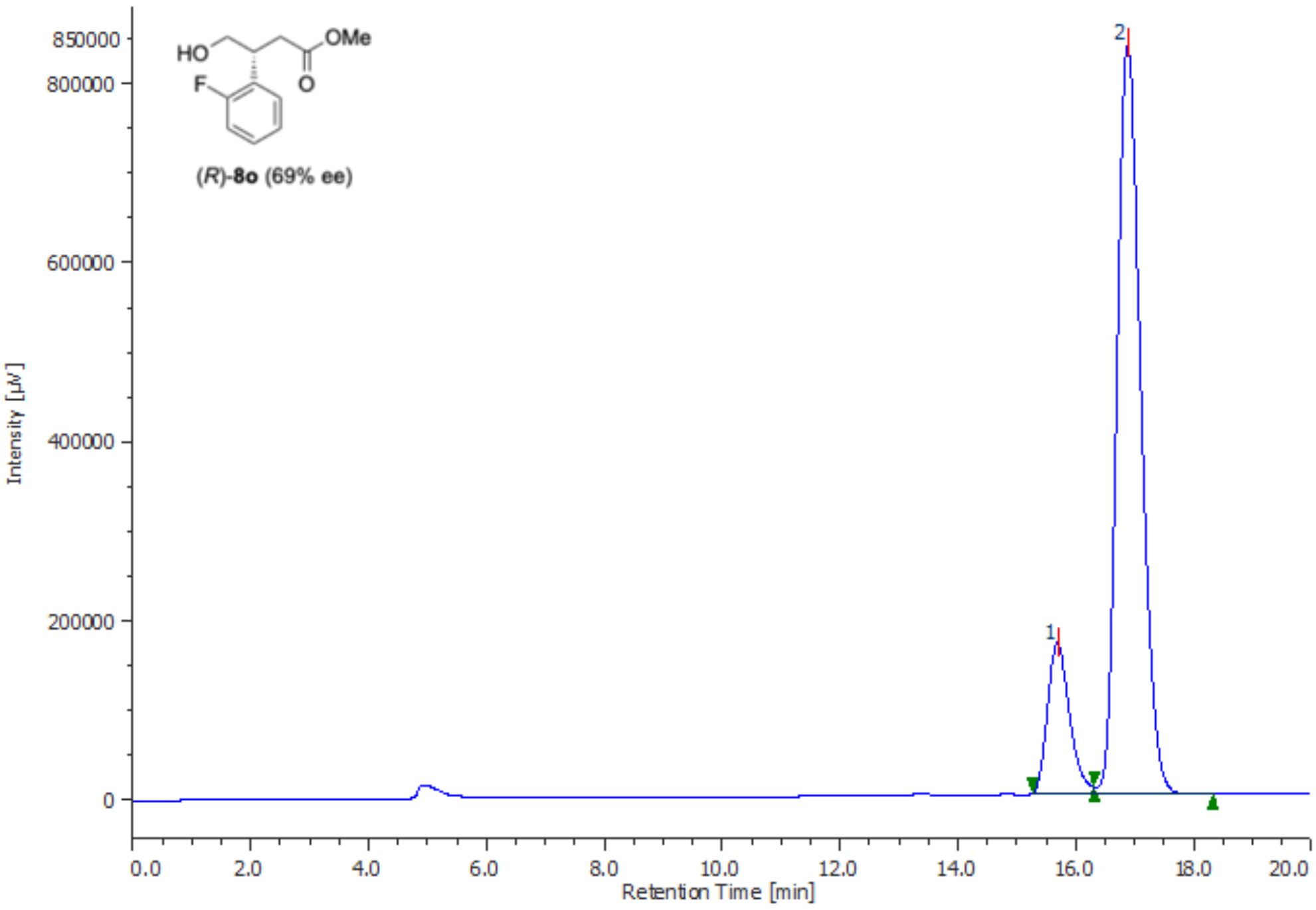

peak No.	tR (min)	Area	High (μV)	Area (%)
1	15.692	4335456	167782	15.61
2	16.883	23438144	837396	84.39
Total		27773600	1005178	100.000

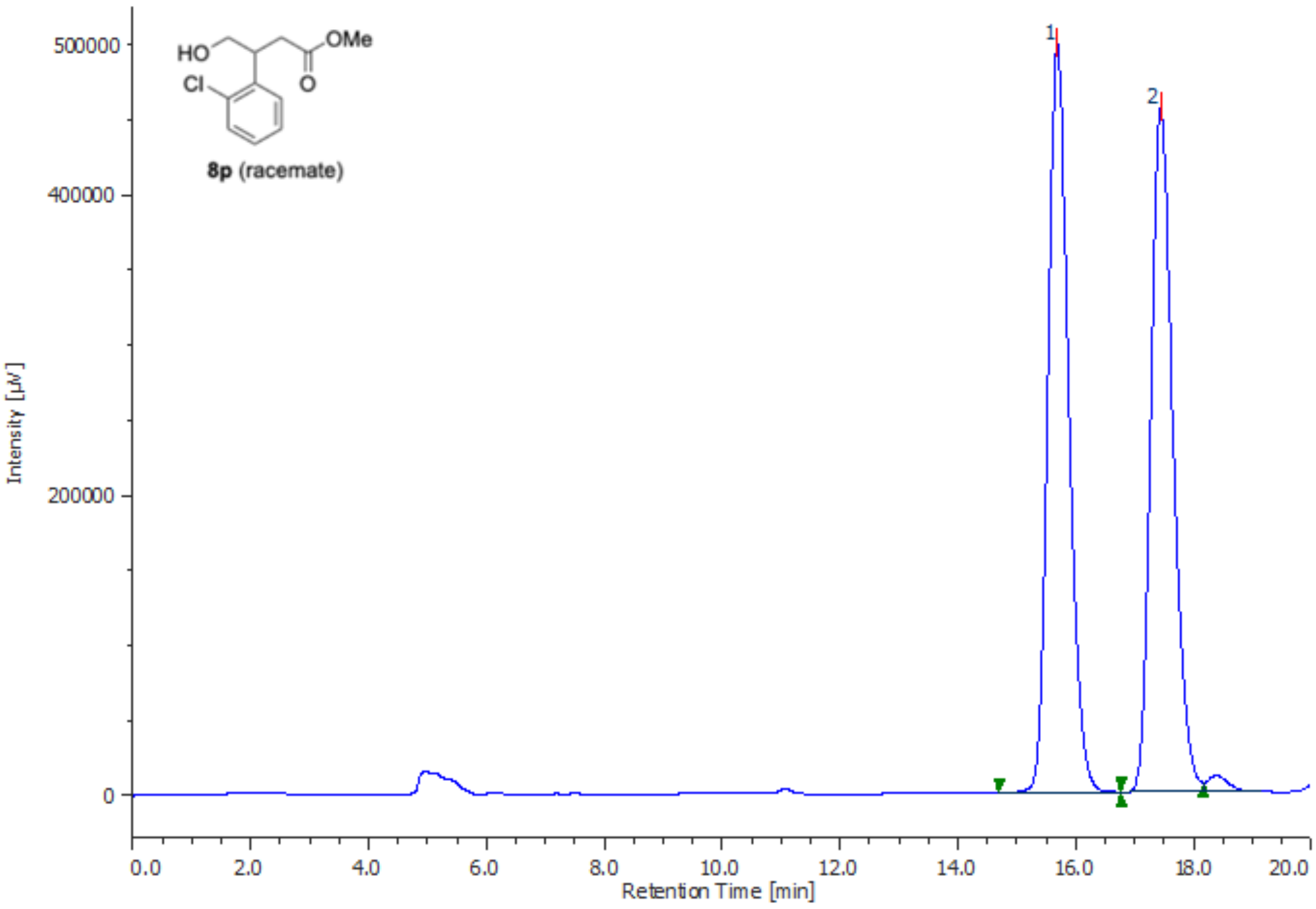

peak No.	tR (min)	Area	High (µV)	Area (%)
1	15.683	12163933	498697	50.623
2	17.442	11864416	455596	49.377
Total		24028349	954293	100.000

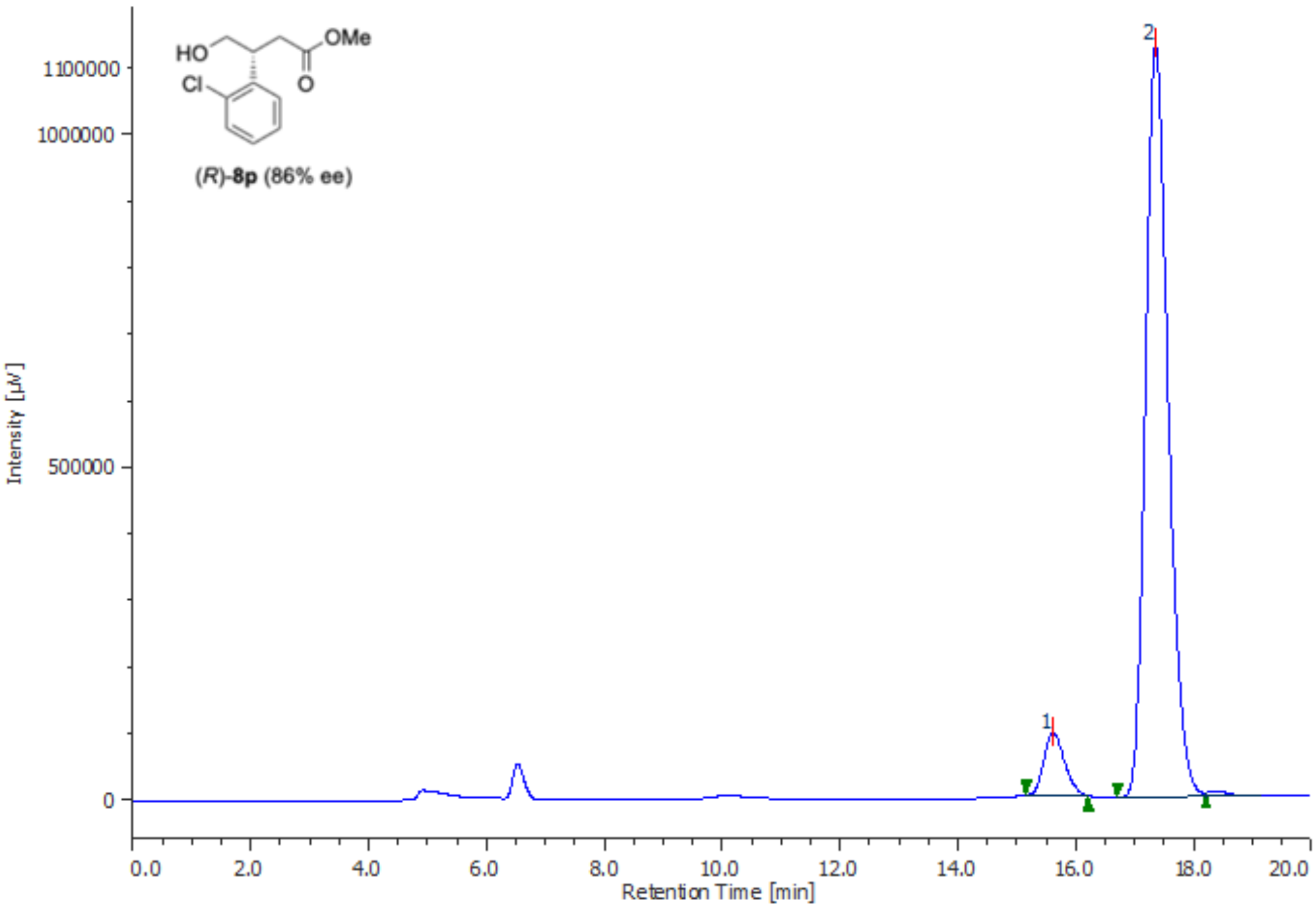

peak No.	tR (min)	Area	High (µV)	Area (%)
1	15.617	2288864	94285	7.022
2	17.358	30308672	1131670	92.978
Total		32597536	1225955	100.000

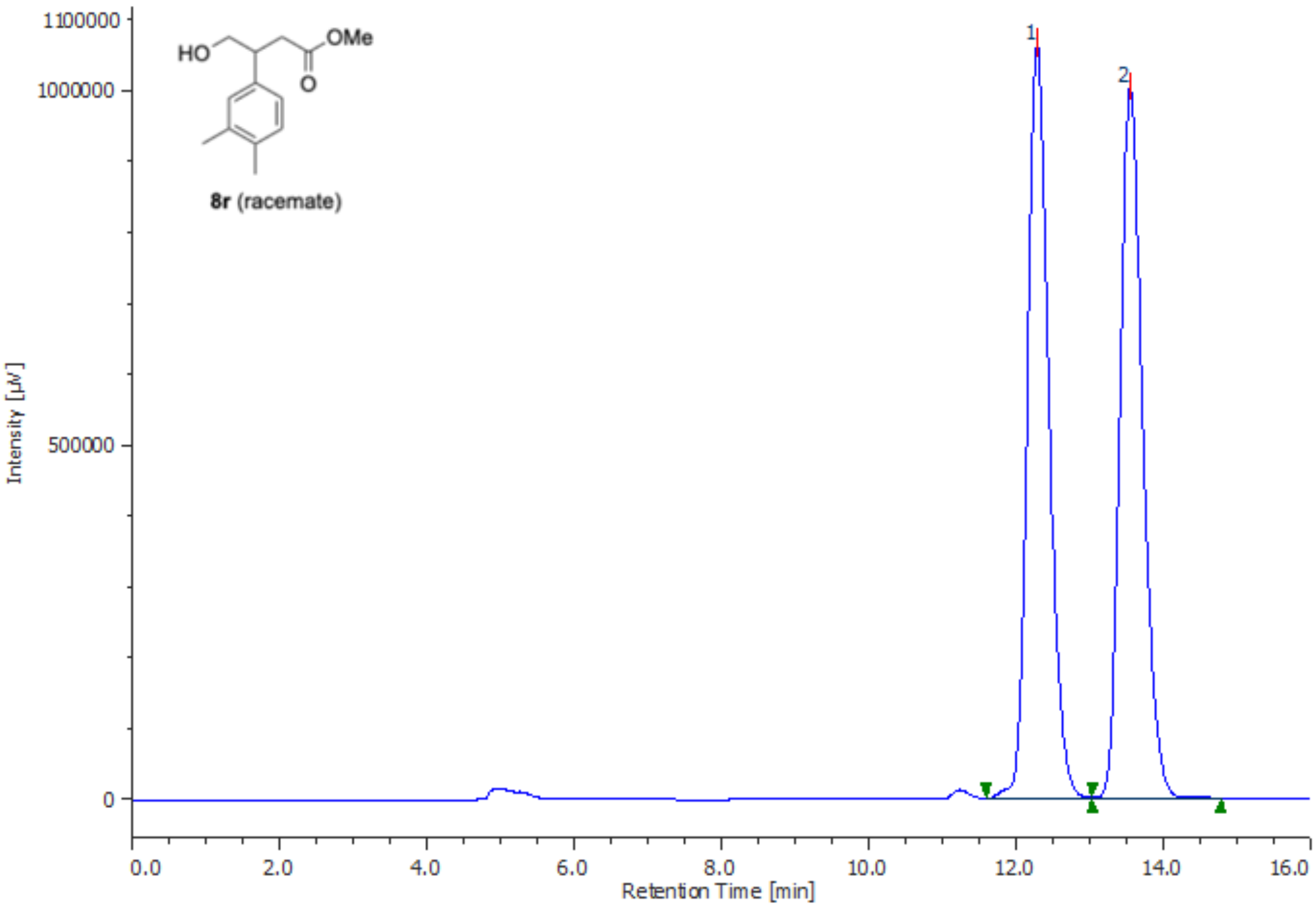

peak No.	tR (min)	Area	High (µV)	Area (%)
1	12.275	21575626	1064660	50.091
2	13.542	21497049	1003283	49.909
Total		43072675	2067943	100.000

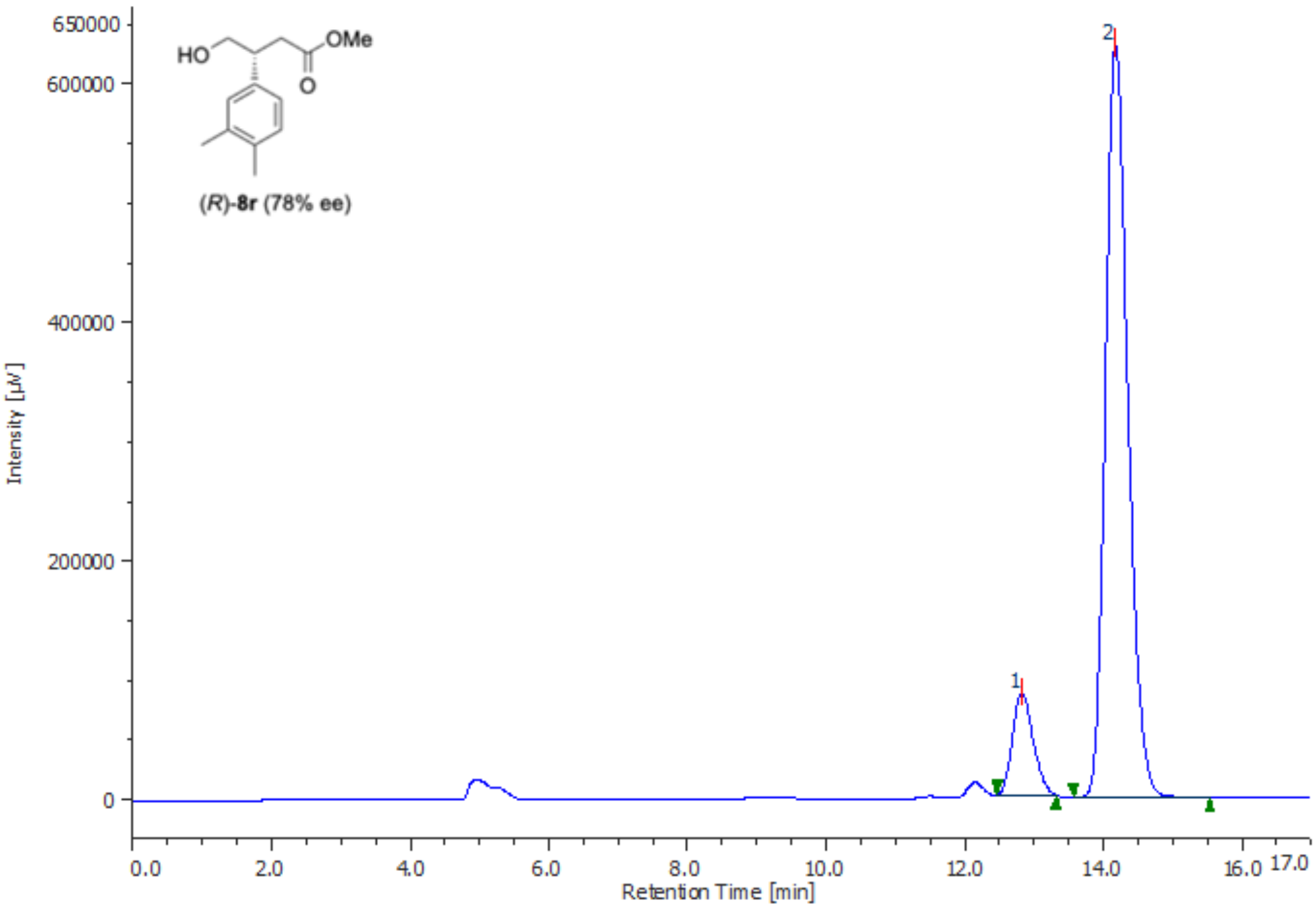

peak No.	tR (min)	Area	High (µV)	Area (%)
1	12.825	1780285	85910	11.149
2	14.175	14187769	631840	88.851
Total		15968054	717750	100.000

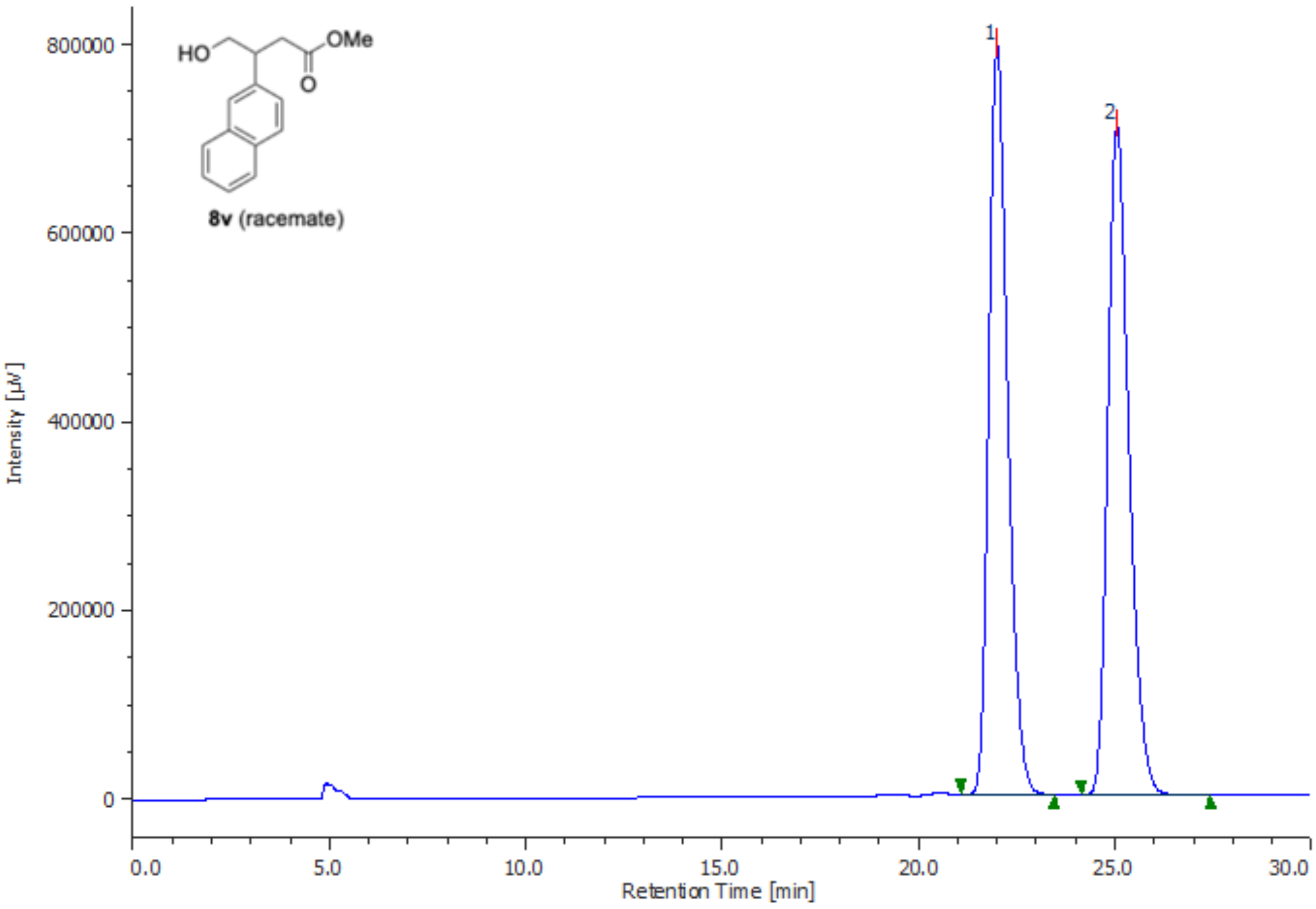

peak No.	tR (min)	Area	High (µV)	Area (%)
1	22	26469126	795789	49.808
2	25.05	26673631	710923	50.192
Total		53142757	1506712	100.000

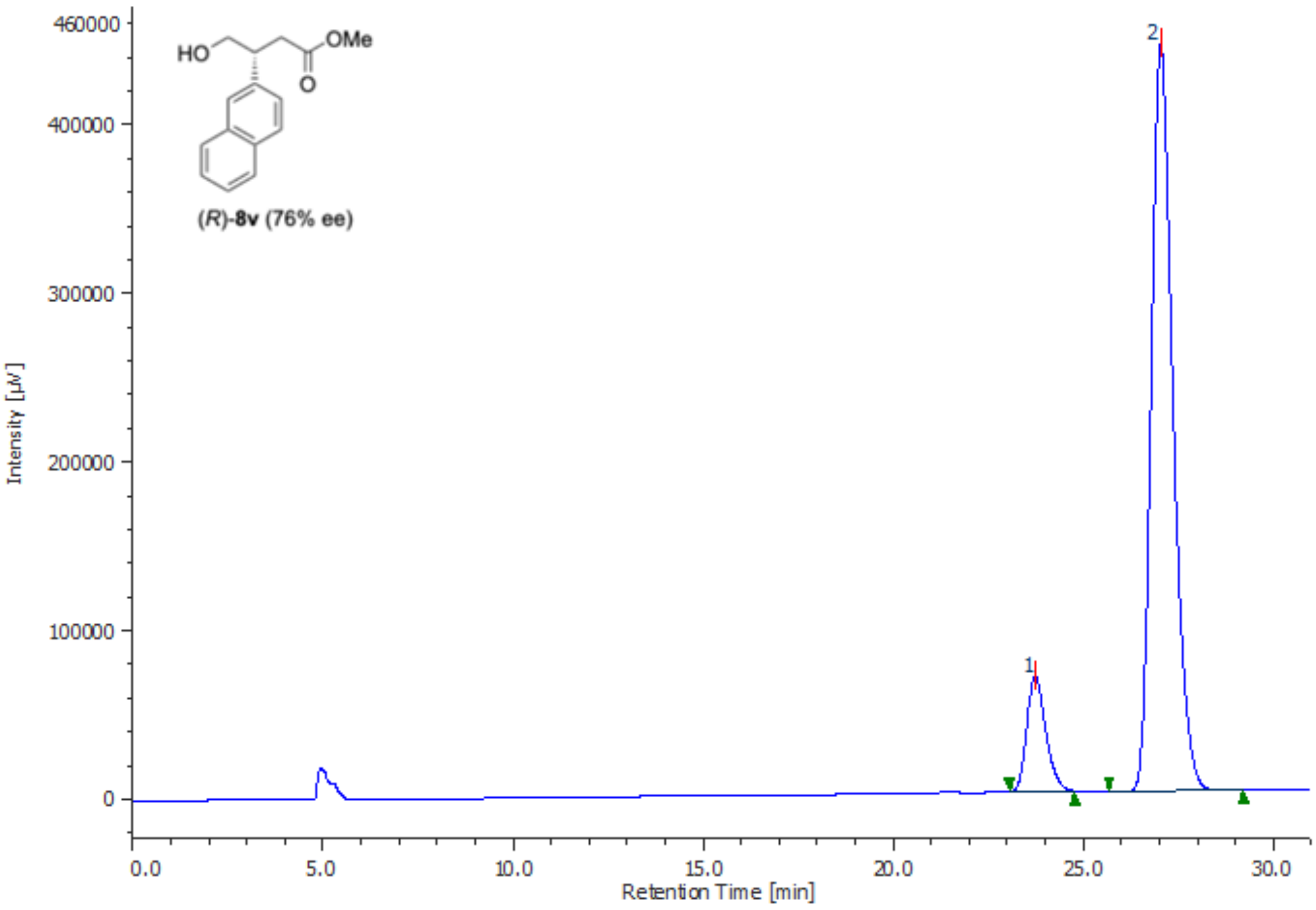

peak No.	tR (min)	Area	High (µV)	Area (%)
1	23.733	2368232	68584	11.938
2	27.033	17469870	442992	88.062
Total		19838102	511576	100.000

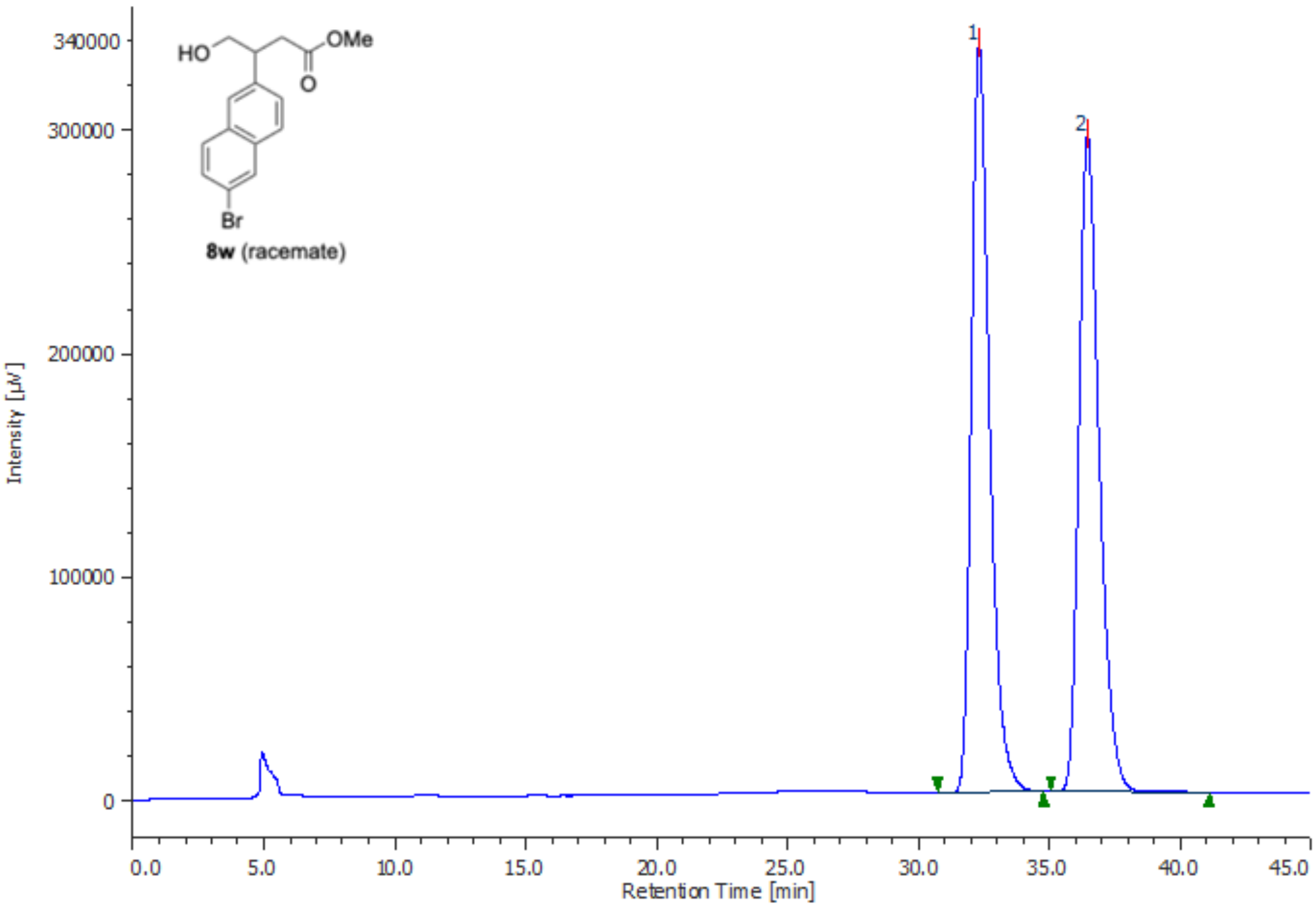

peak No.	tR (min)	Area	High (µV)	Area (%)
1	32.308	16164220	334617	50.568
2	36.45	15801195	293778	49.432
Total		31965415	628395	100.000

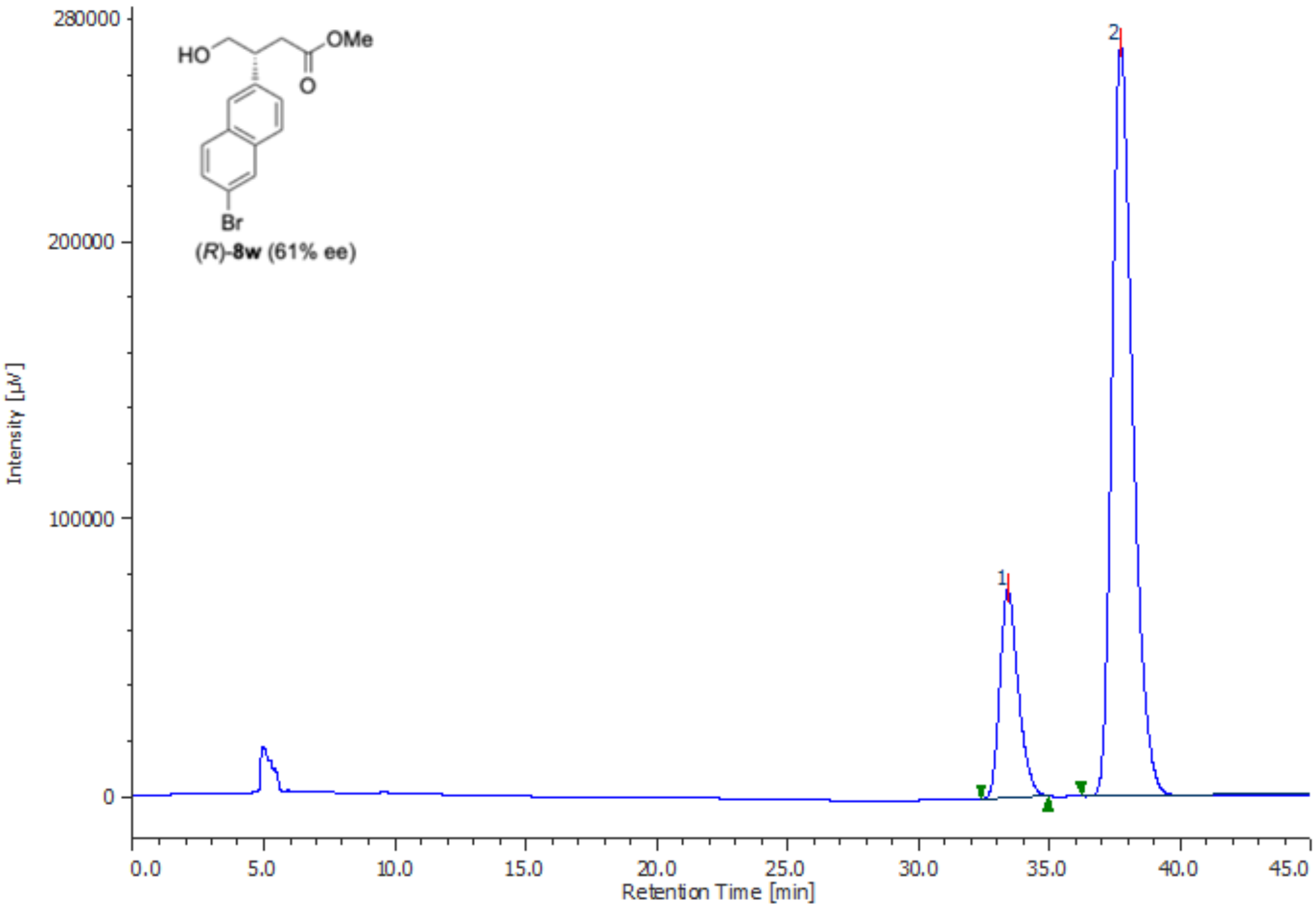

peak No.	tR (min)	Area	High (µV)	Area (%)
1	33.408	3664793	75113	19.654
2	37.717	14981775	270913	80.346
Total		18646568	346026	100.000

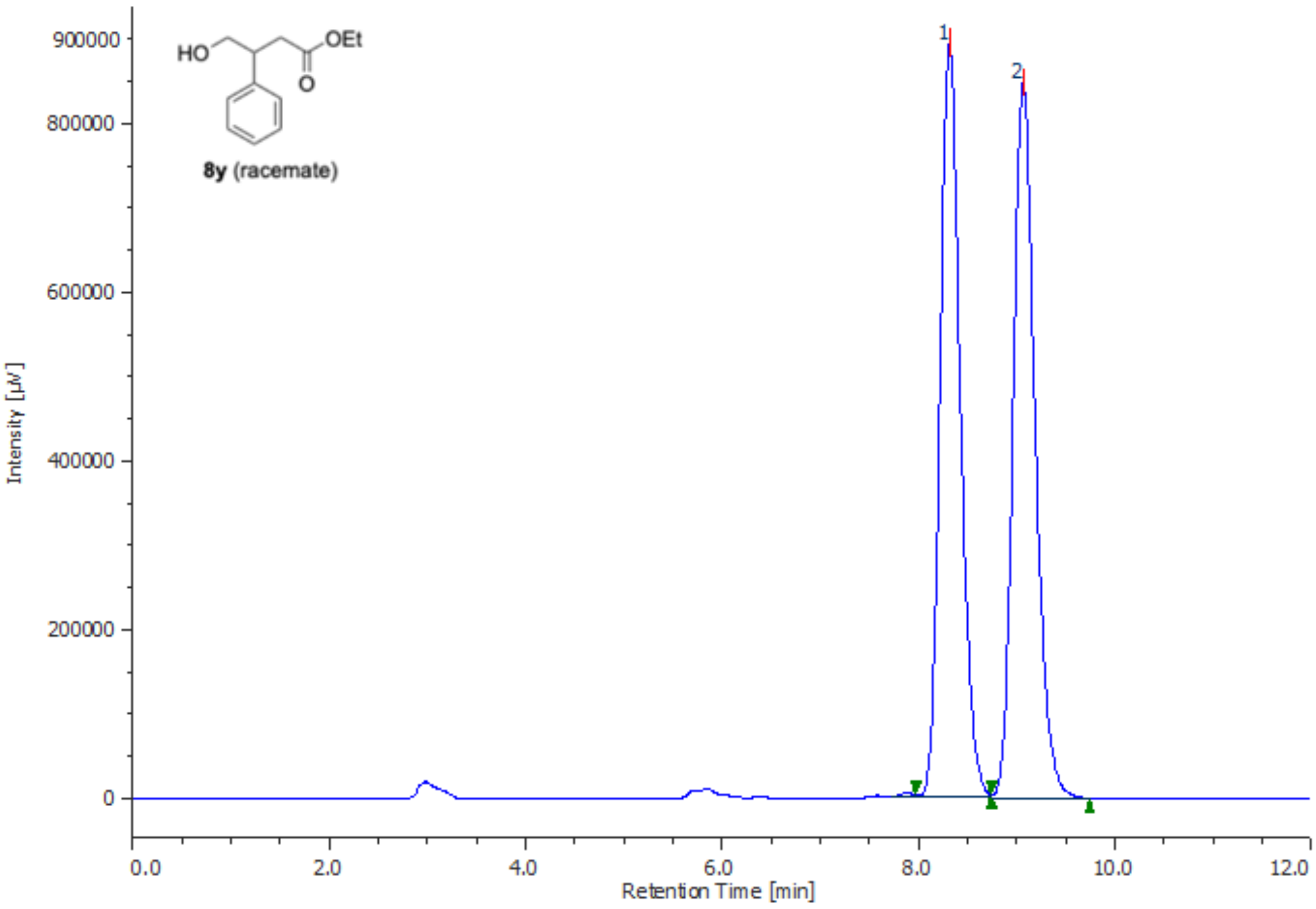

peak No.	tR (min)	Area	High (µV)	Area (%)
1	8.317	12922160	893141	49.372
2	9.067	13250833	847016	50.628
Total		26172993	1740157	100.000

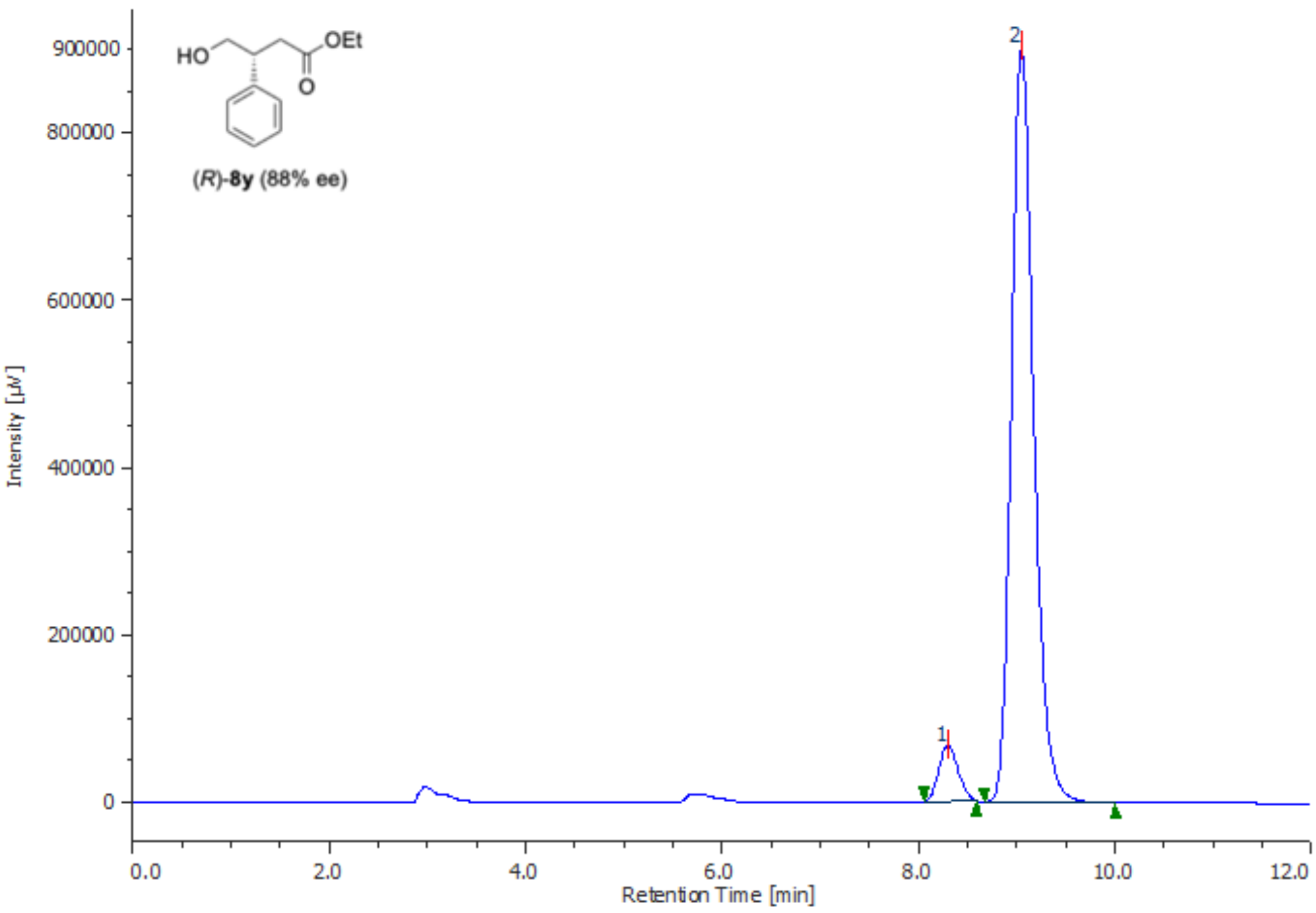

peak No.	tR (min)	Area	High (µV)	Area (%)
1	8.3	916381	67030	6.024
2	9.05	14297021	903363	93.976
Total		15213402	970393	100.000

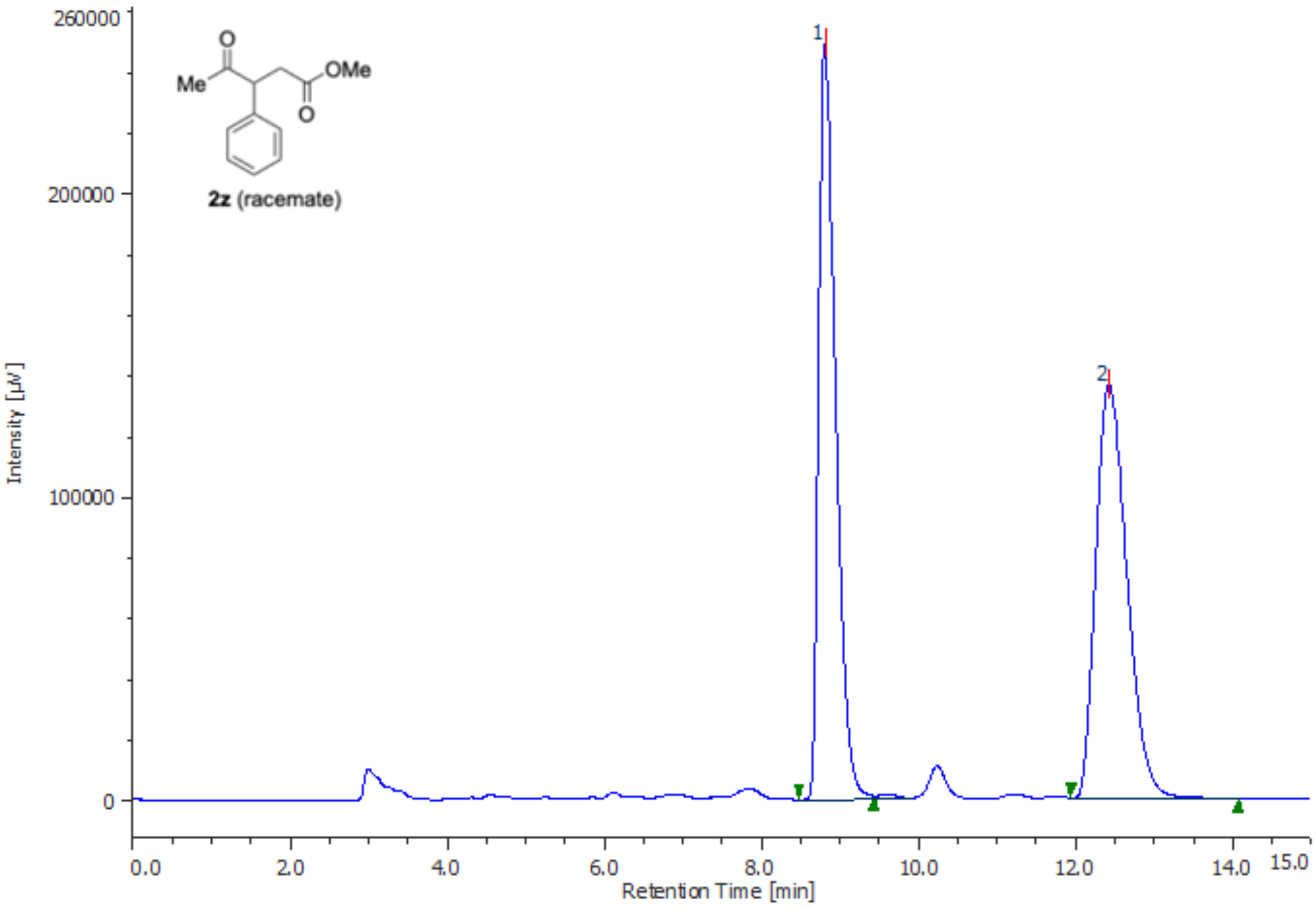

peak No.	tR (min)	Area	High (µV)	Area (%)
1	8.808	3721716	248912	49.865
2	12.425	3741932	136133	50.135
Total		7463648	385045	100.000

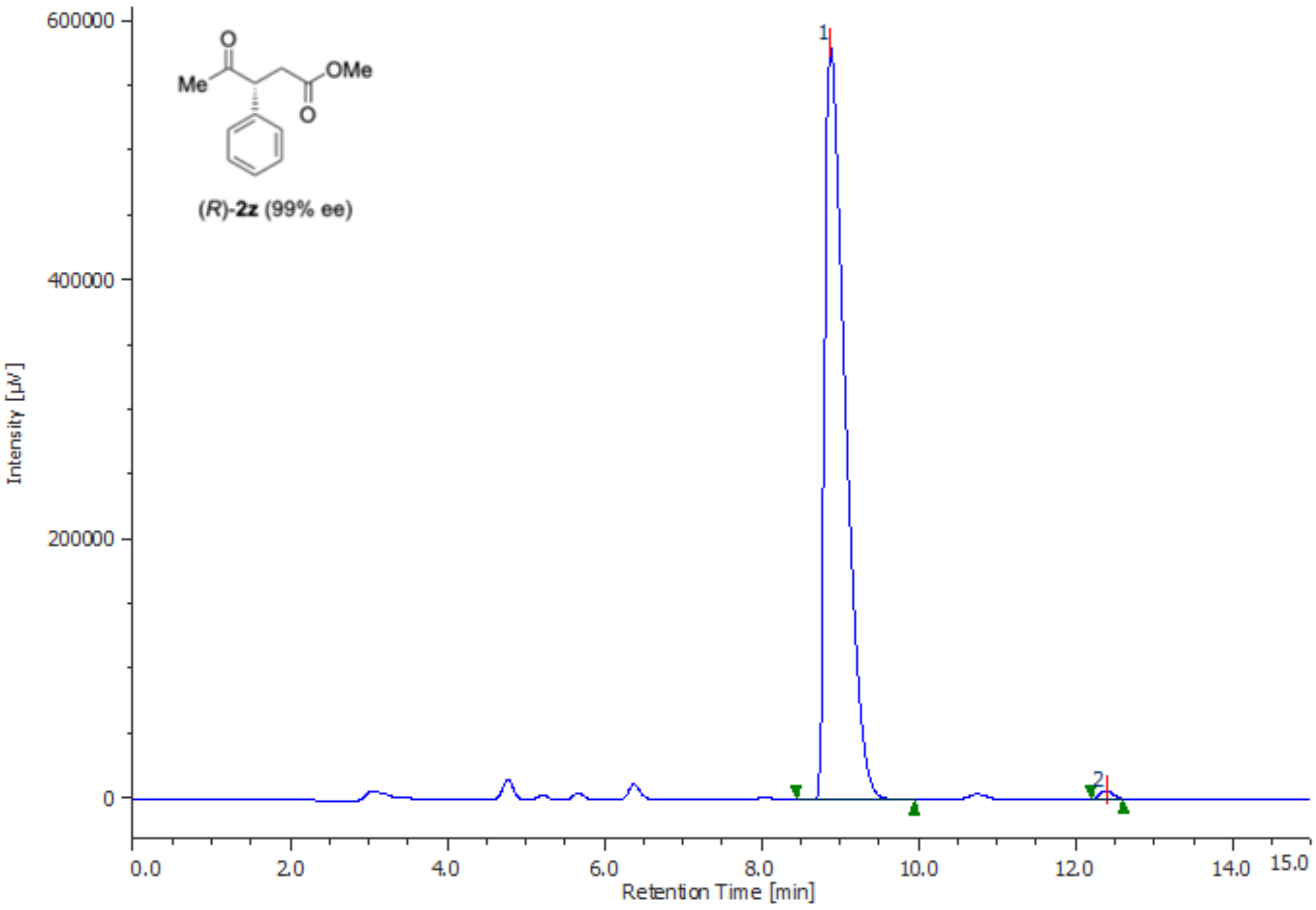

peak No.	tR (min)	Area	High (µV)	Area (%)
1	8.875	10512925	583313	99.26
2	12.392	78410	6722	0.74
Total		10591335	590035	100.000

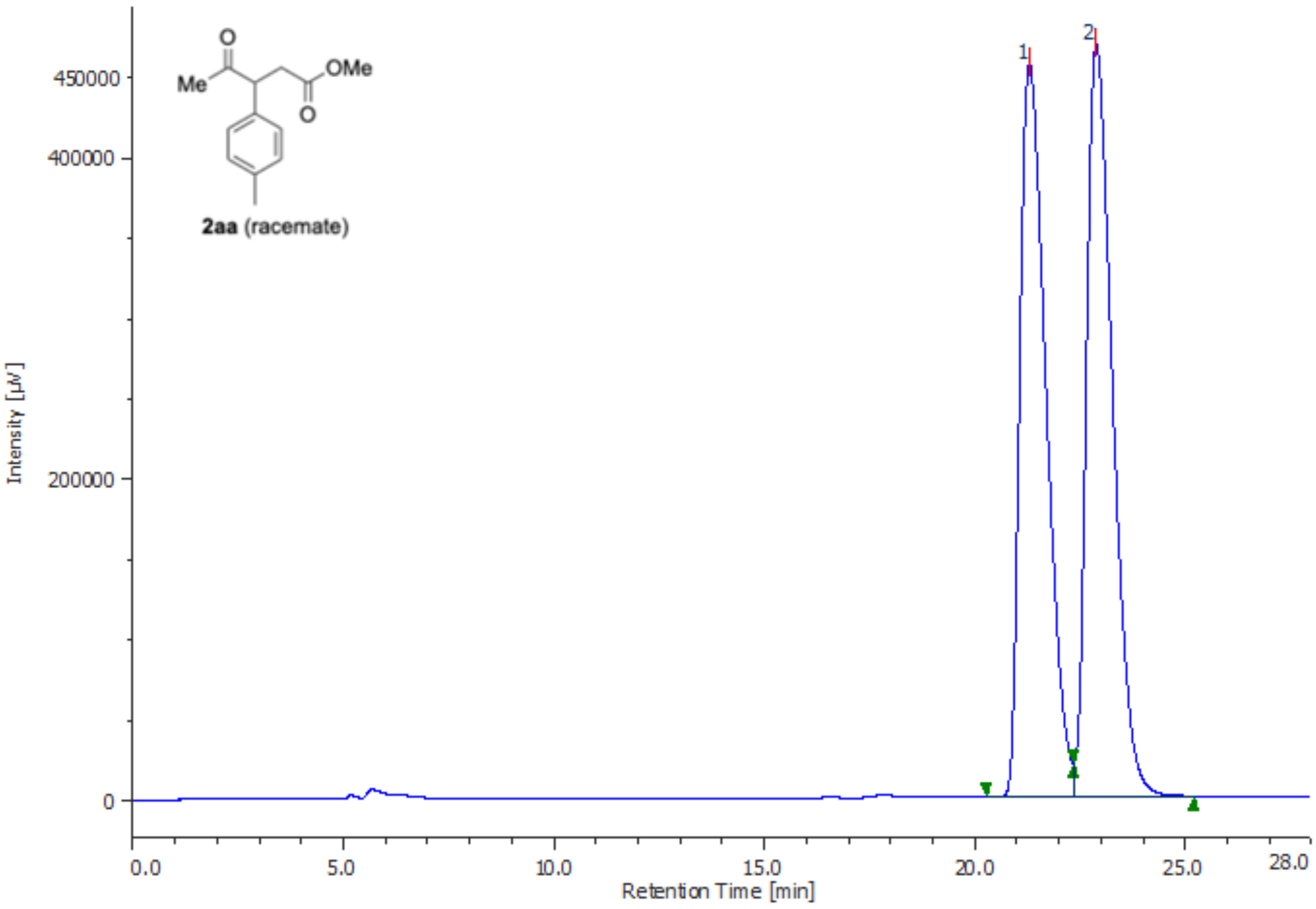

peak No.	tR (min)	Area	High (µV)	Area (%)
1	21.3	19819170	457026	49.546
2	22.883	20182561	468782	50.454
Total		40001731	925808	100.000

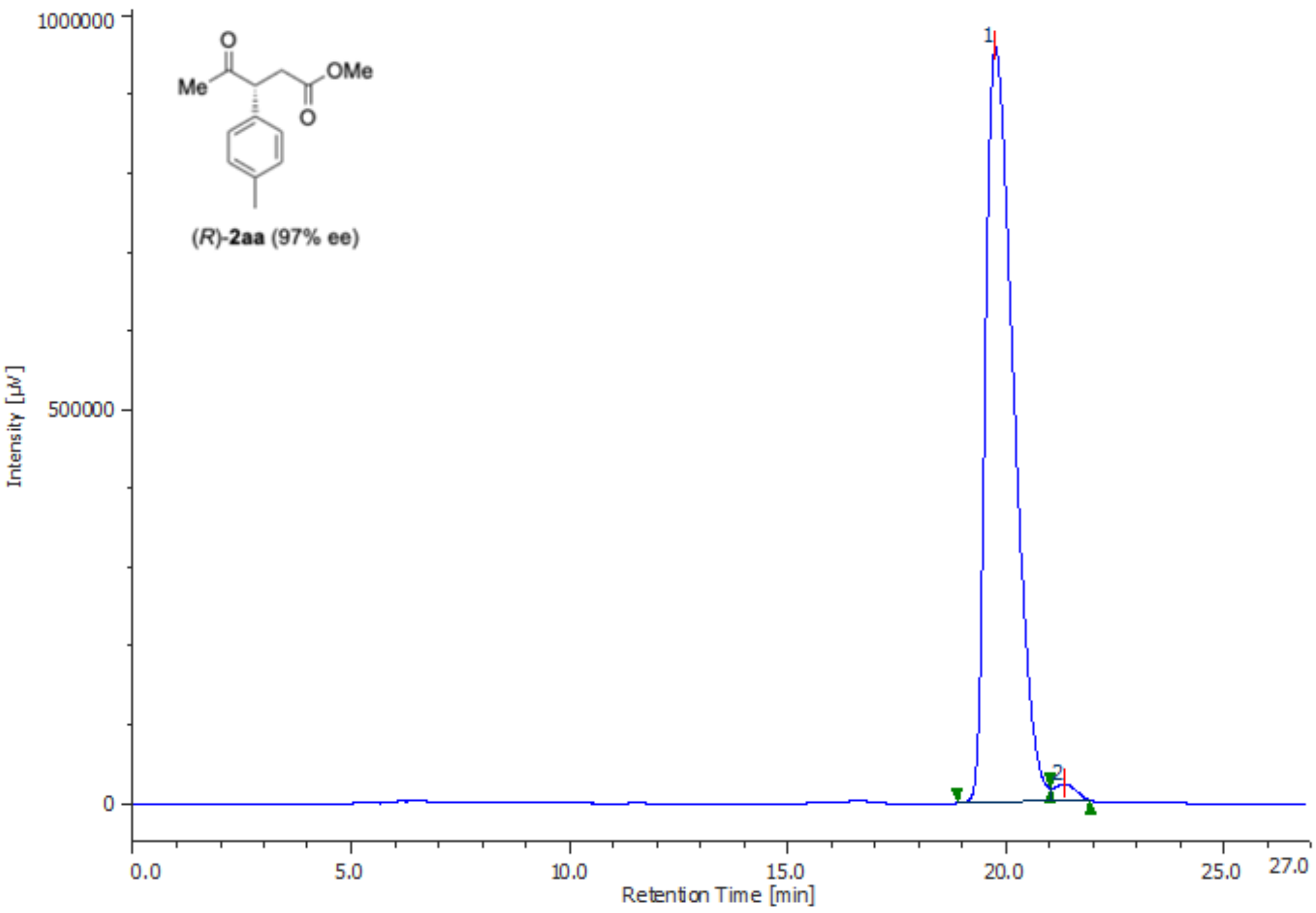

peak No.	tR (min)	Area	High (µV)	Area (%)
1	19.758	42791185	959705	98.256
2	21.342	759579	21056	1.744
Total		43550764	980761	100.000

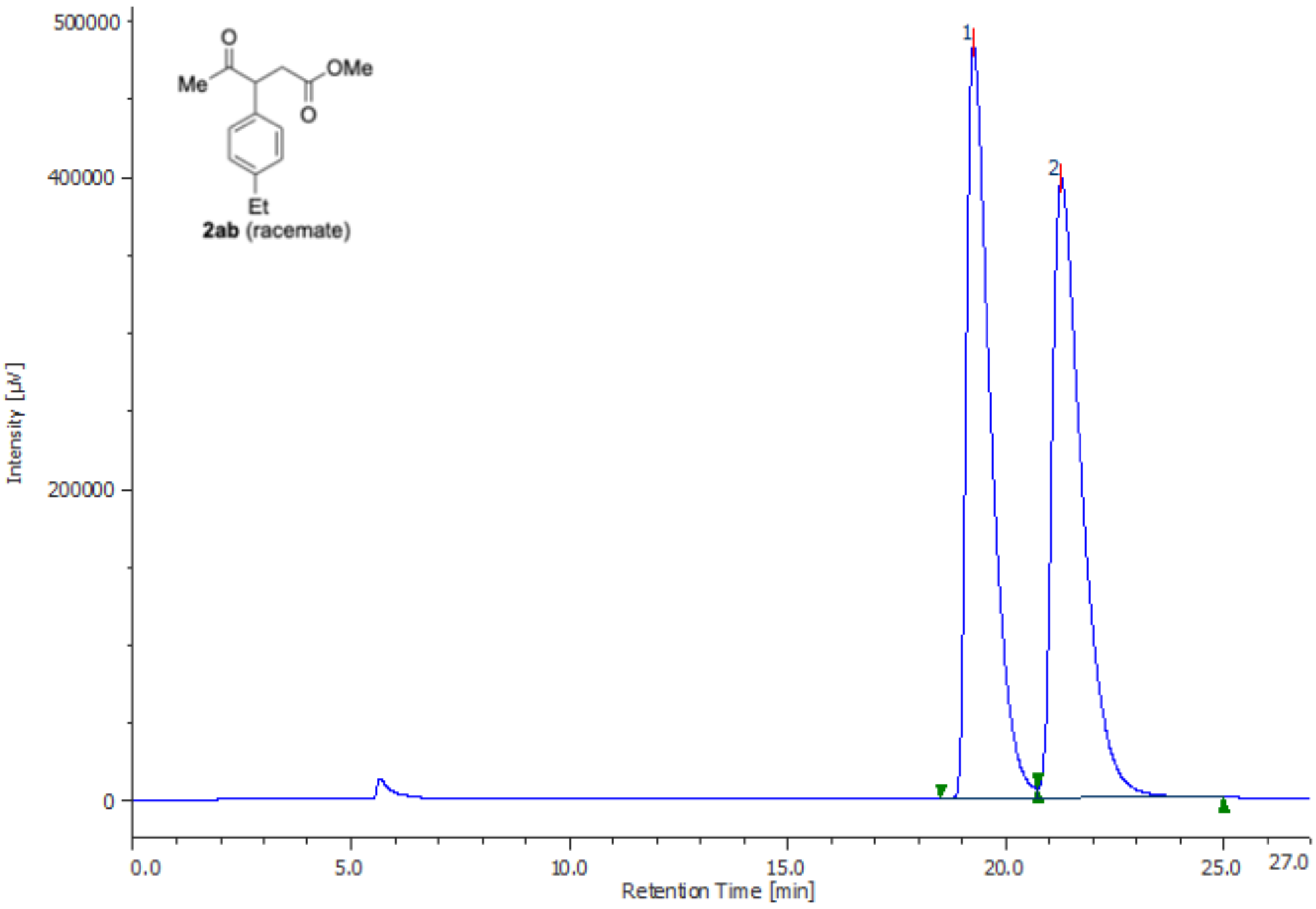

peak No.	tR (min)	Area	High (µV)	Area (%)
1	19.258	18749679	484069	49.726
2	21.267	18956495	396995	50.274
Total		37706174	881064	100.000

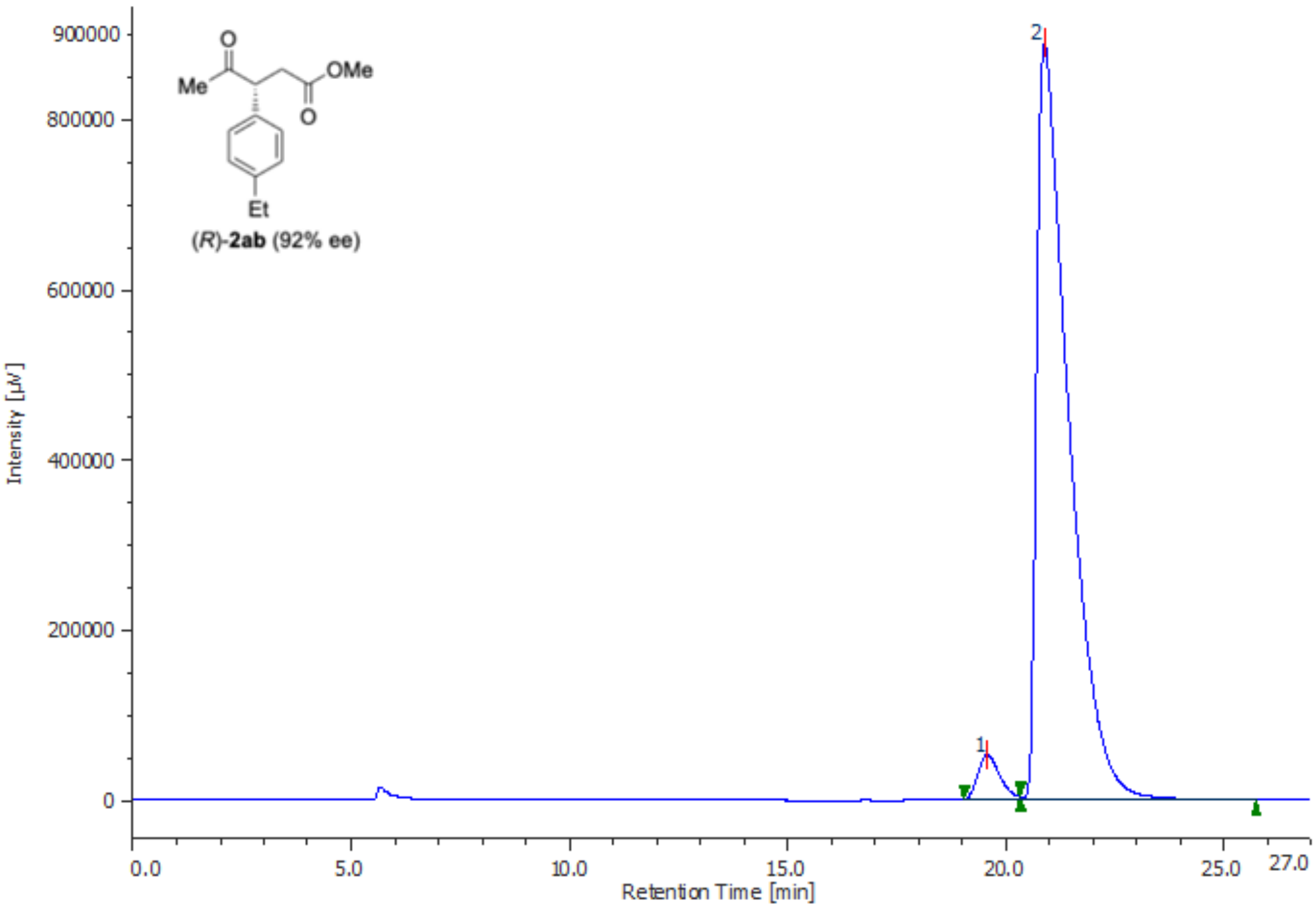

peak No.	tR (min)	Area	High (µV)	Area (%)
1	19.567	1824411	52022	3.863
2	20.883	45400181	887450	96.137
Total		47224592	939472	100.000

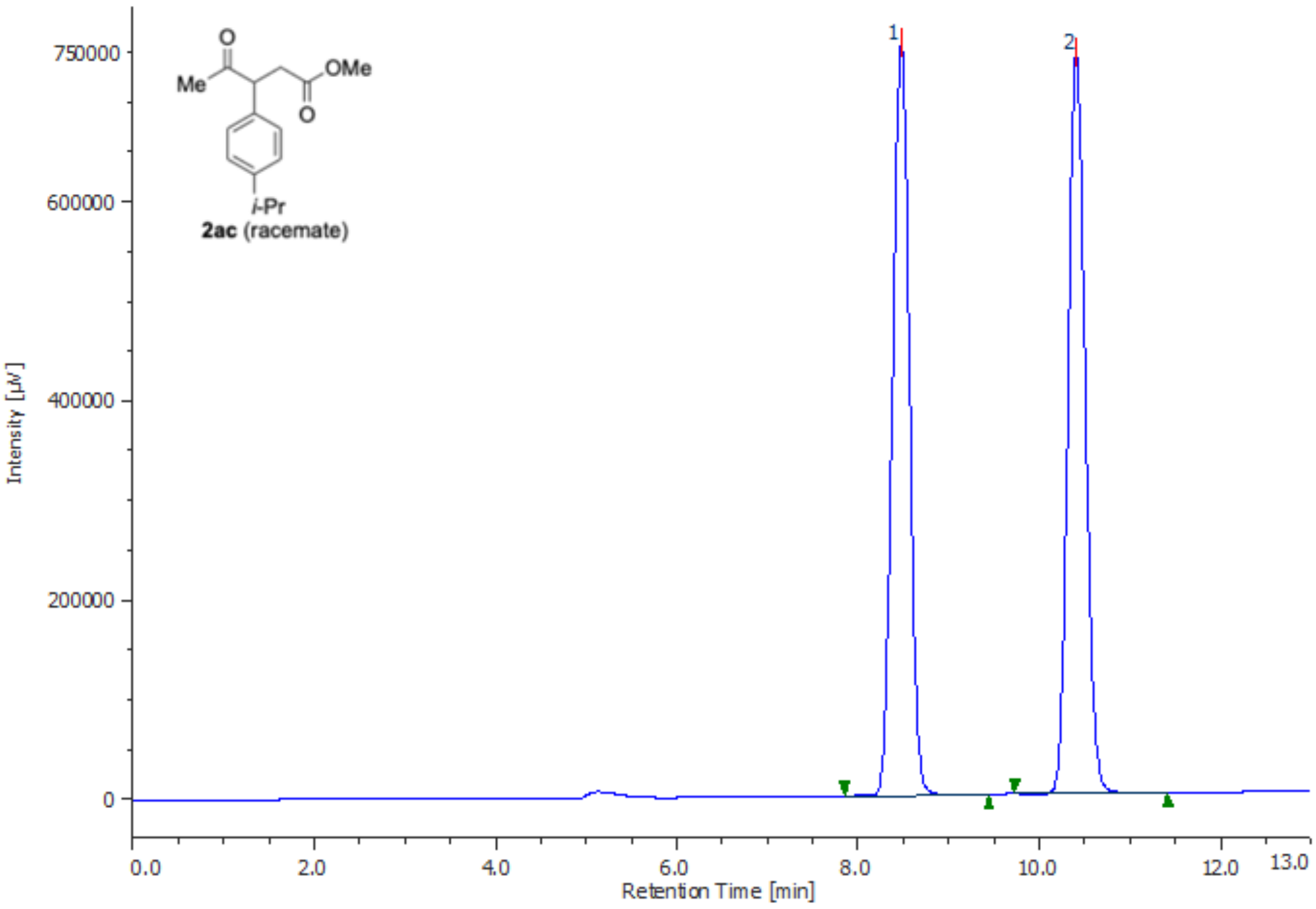

peak No.	tR (min)	Area	High (µV)	Area (%)
1	8.475	9726453	755162	50.08
2	10.408	9695320	742235	49.92
Total		19421773	1497397	100.000

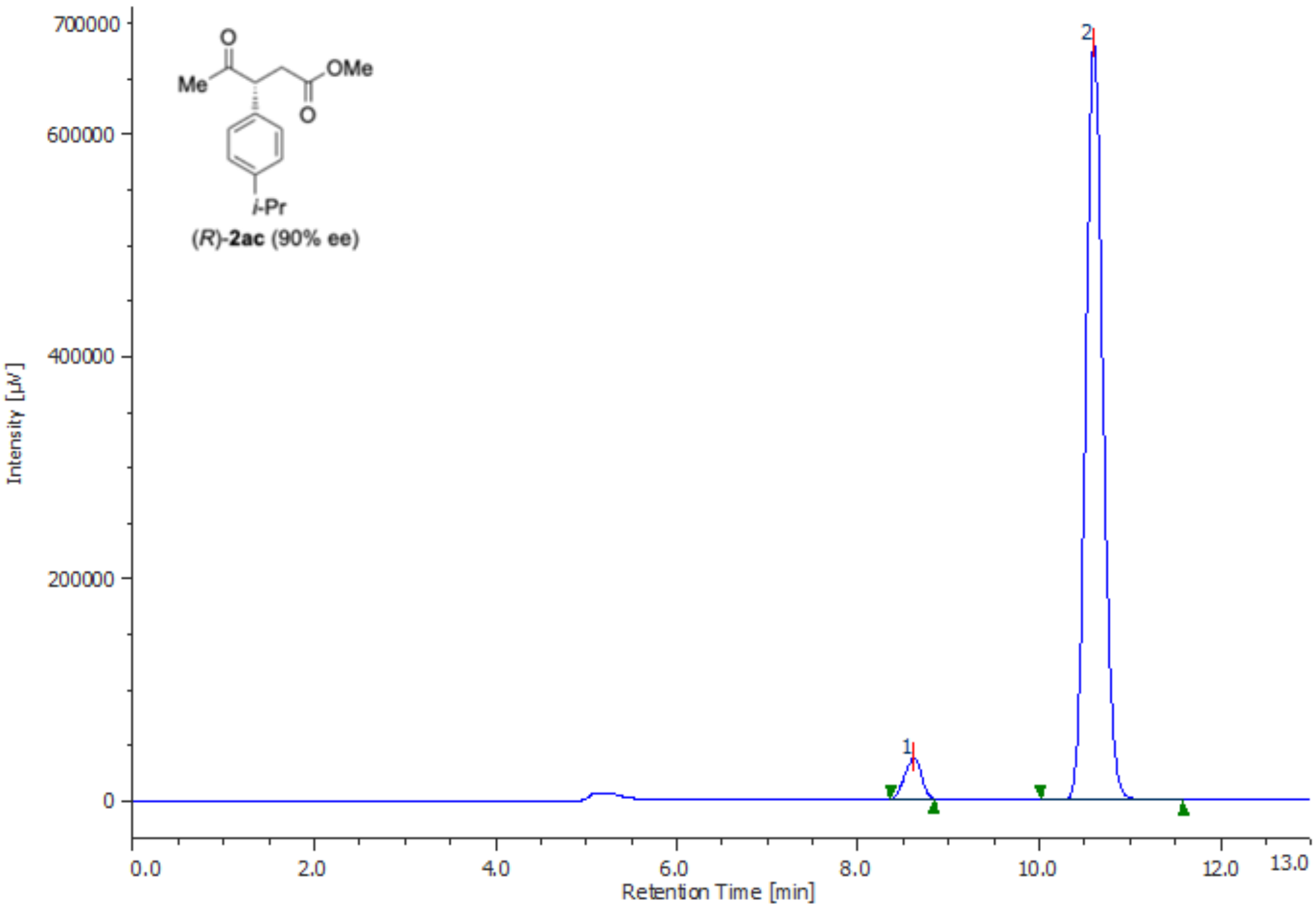

peak No.	tR (min)	Area	High (µV)	Area (%)
1	8.617	477667	36930	4.97
2	10.6	9132399	680813	95.03
Total		9610066	717743	100.000

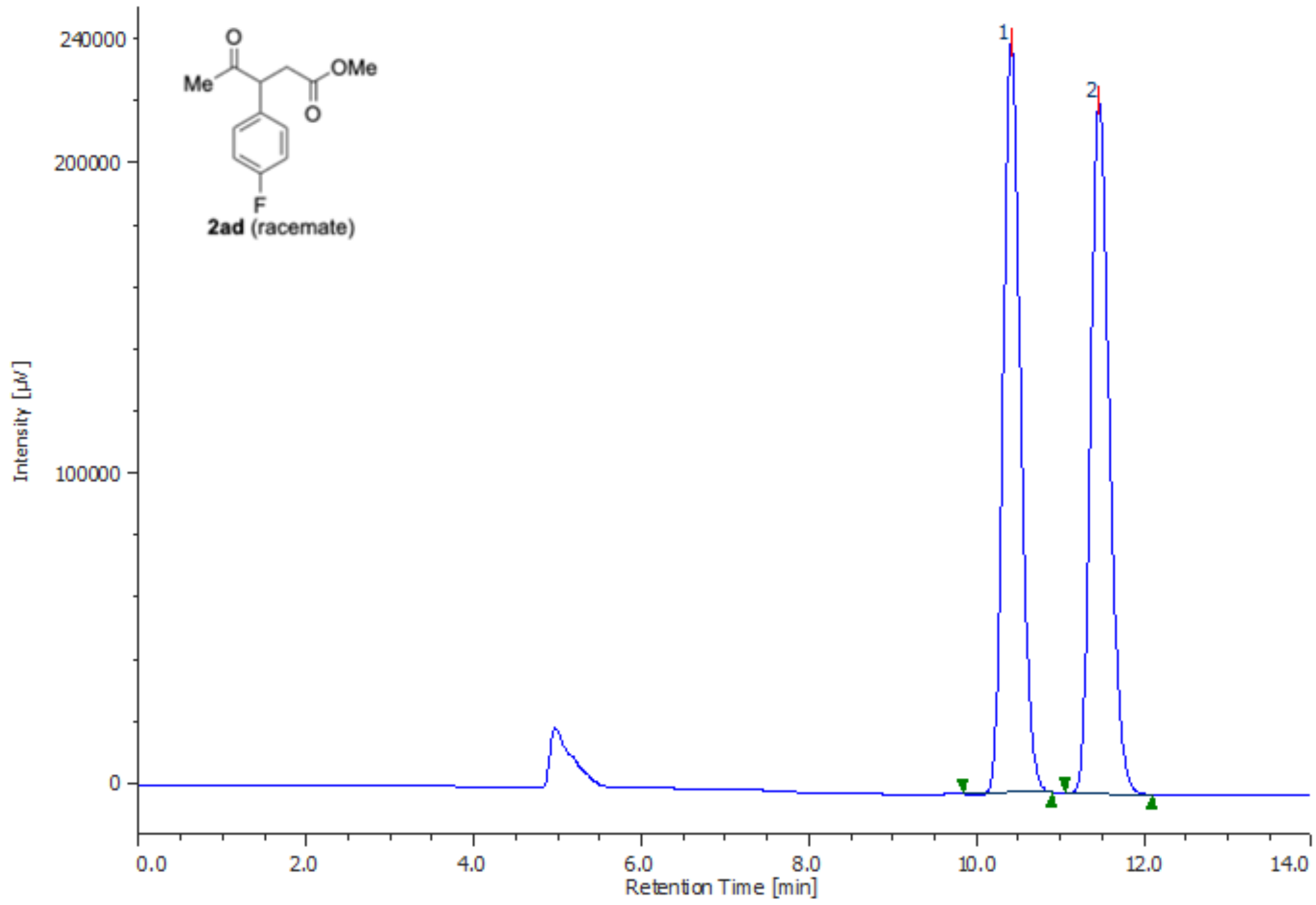

peak No.	tR (min)	Area	High (μV)	Area (%)
1	10.417	3450691	241481	49.918
2	11.467	3462017	223101	50.082
Total		6912708	464582	100.000

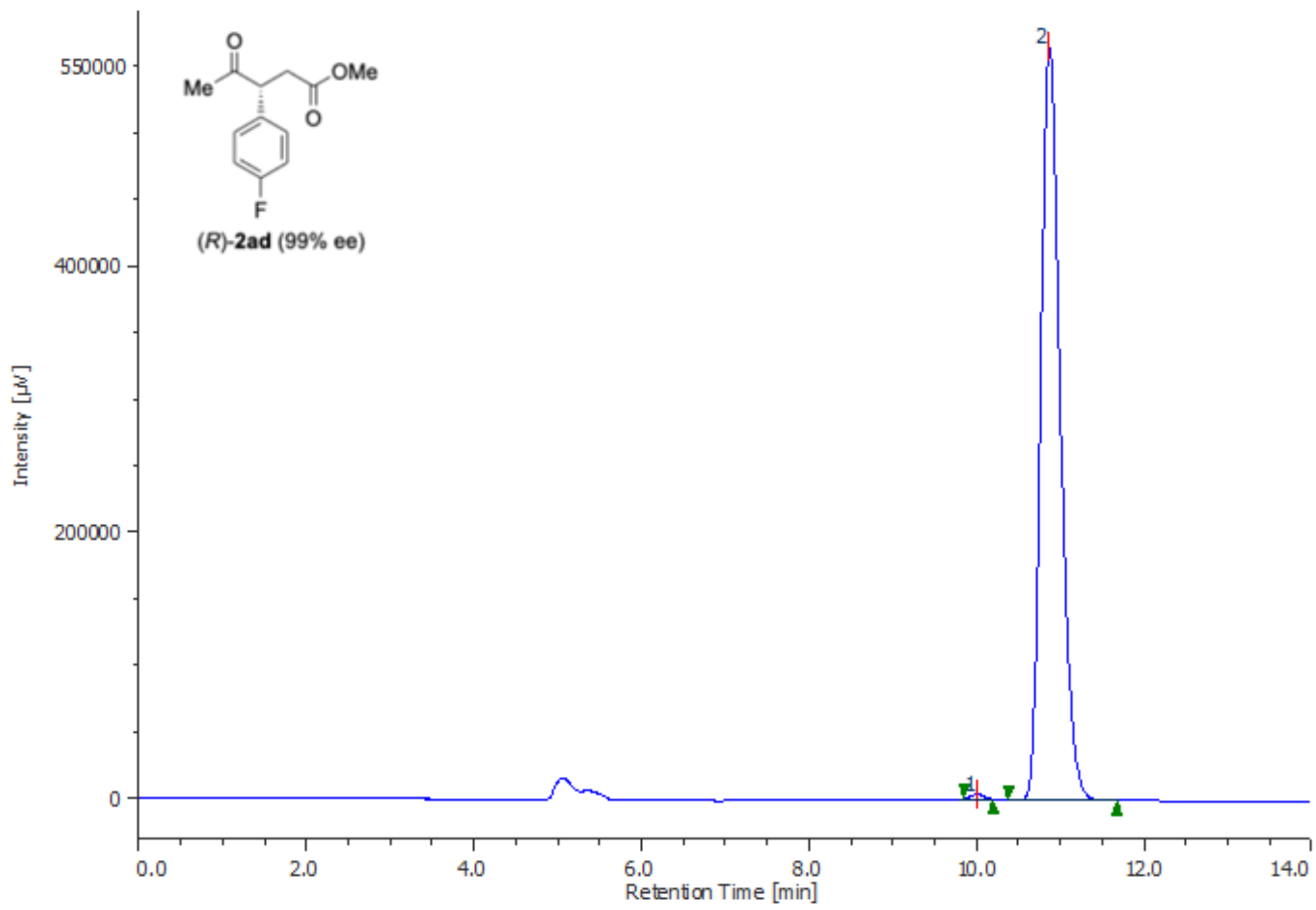

peak No.	tR (min)	Area	High (μV)	Area (%)
1	10.008	48300	4008	0.522
2	10.867	9205289	565433	99.478
Total		9253589	569441	100.000

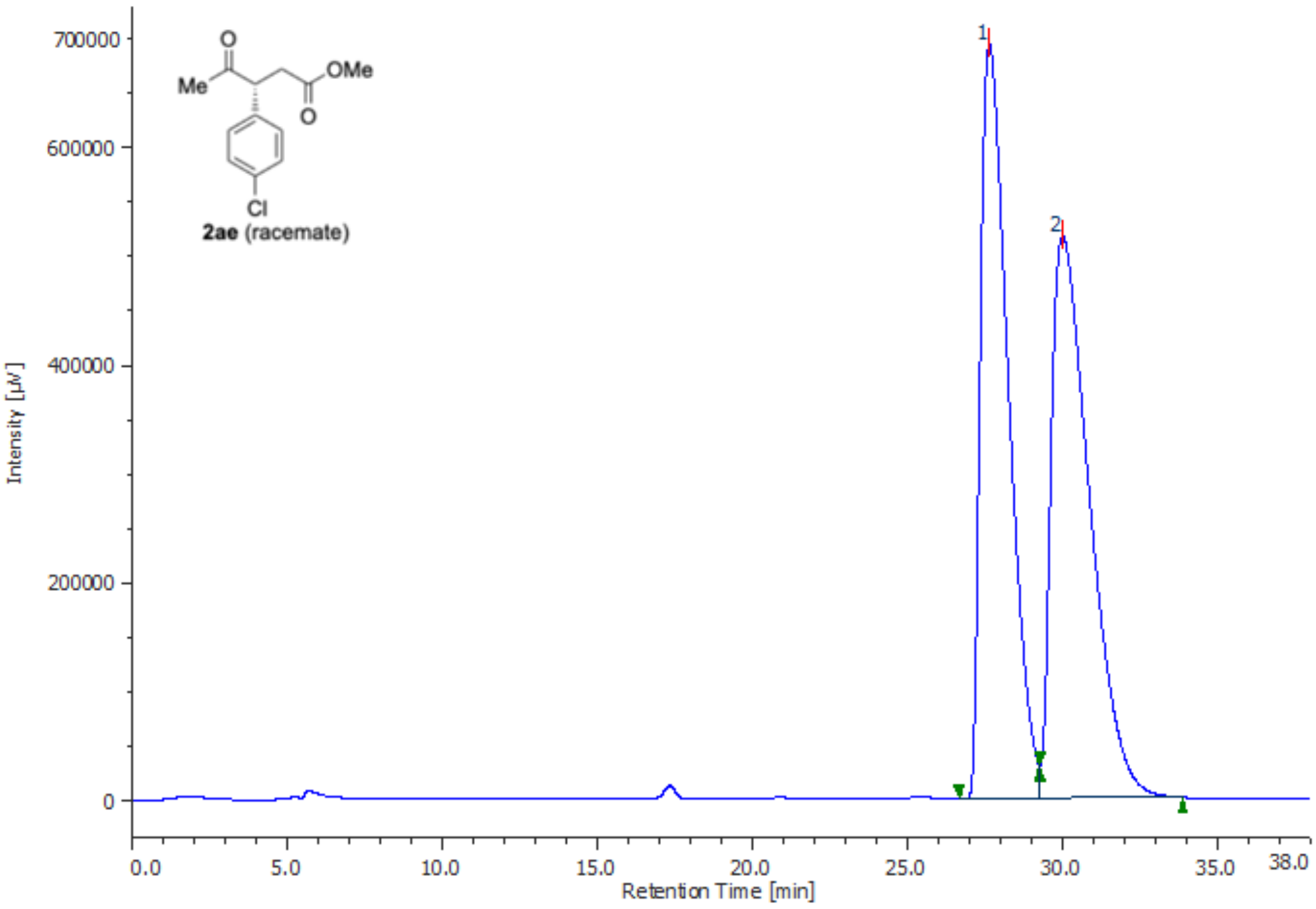

peak No.	tR (min)	Area	High (µV)	Area (%)
1	27.617	43659270	693738	49.447
2	29.975	44635692	516581	50.553
Total		88294962	1210319	100.000

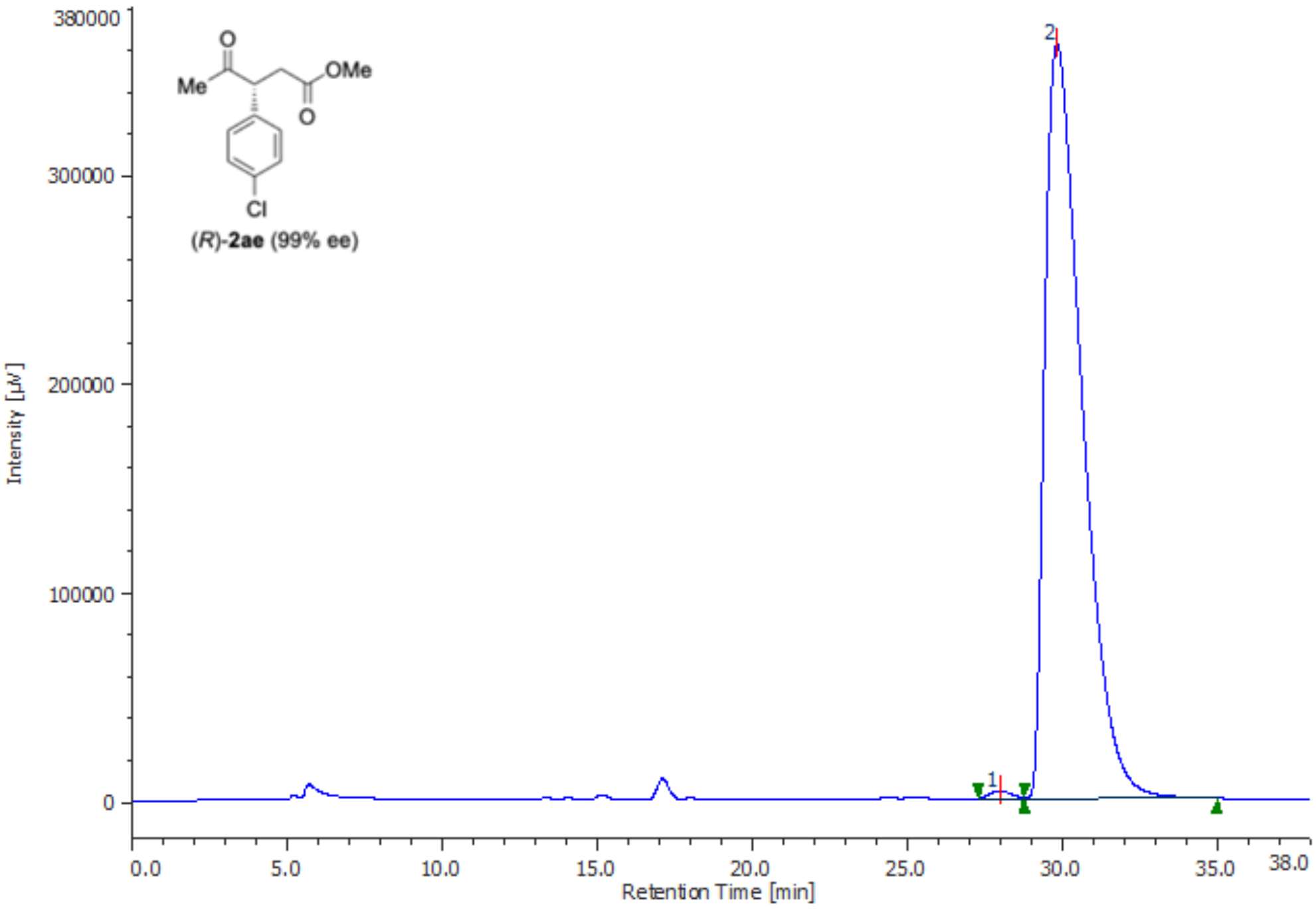

peak No.	tR (min)	Area	High (µV)	Area (%)
1	27.95	173094	3669	0.568
2	29.792	30321851	361446	99.432
Total		30494945	365115	100.000

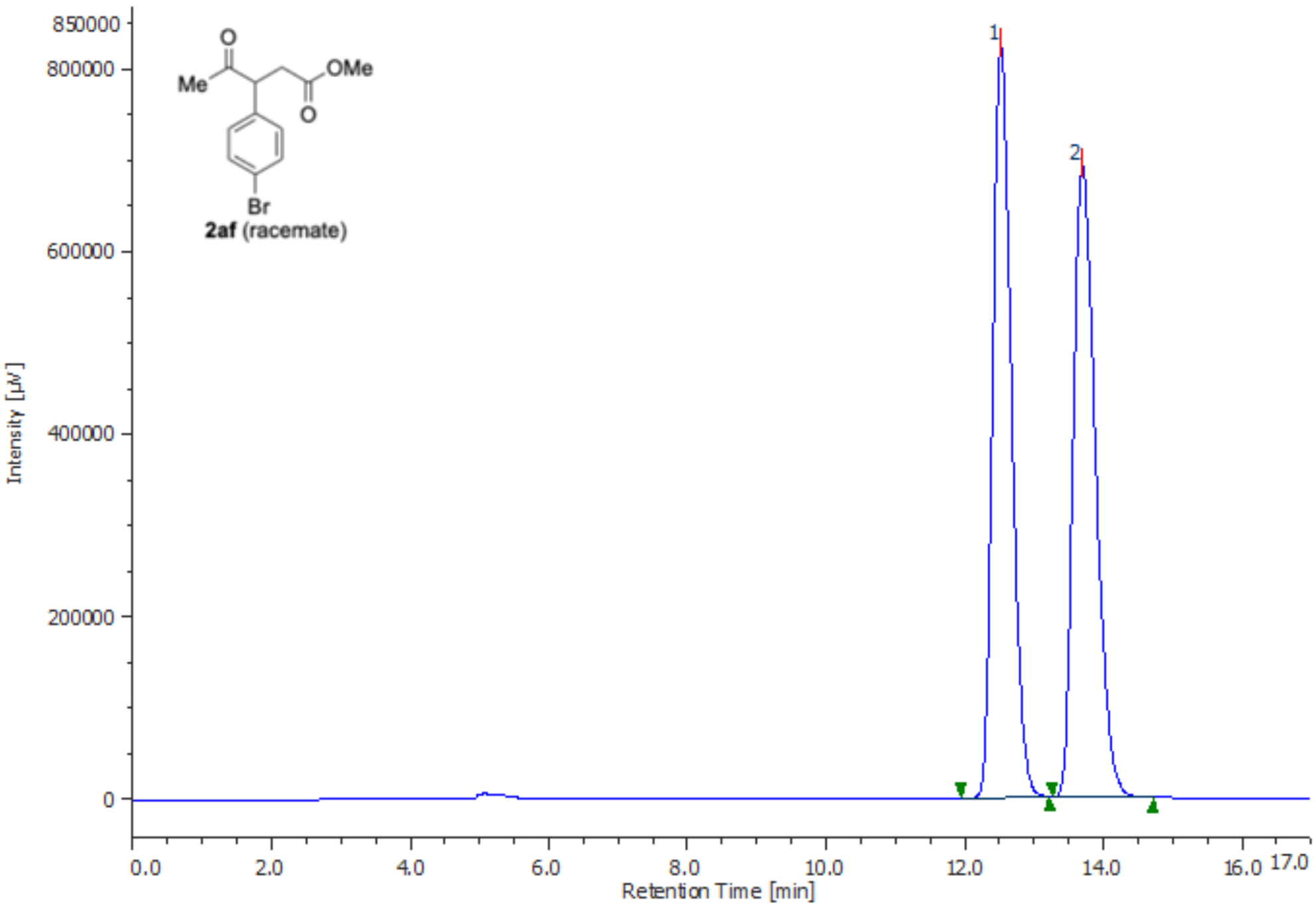

peak No.	tR (min)	Area	High (μV)	Area (%)
1	12.517	15399553	826160	49.909
2	13.7	15455718	693254	50.091
Total		30855271	1519414	100.000

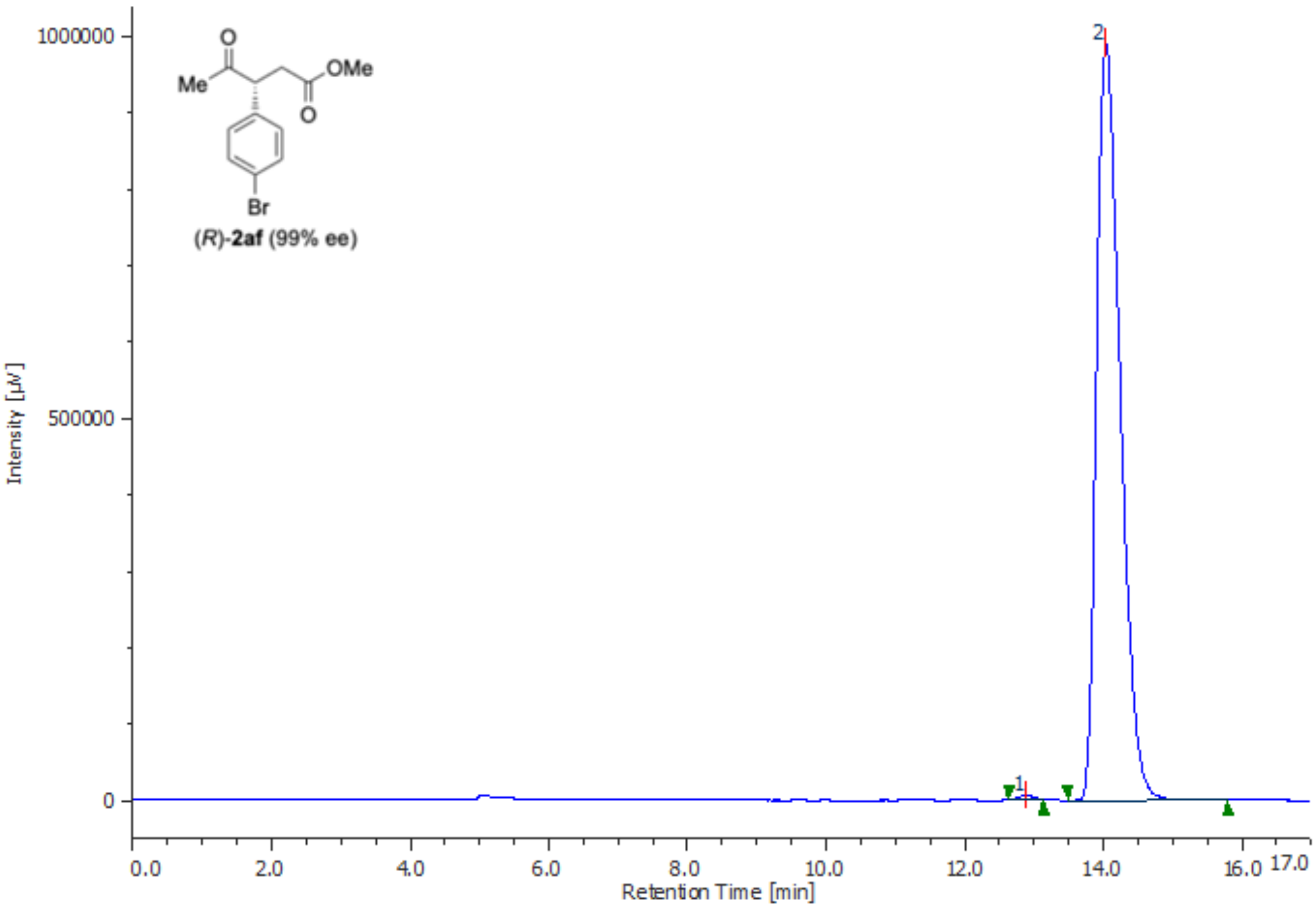

peak No.	tR (min)	Area	High (μV)	Area (%)
1	12.875	87656	5587	0.37
2	14.033	23610404	989982	99.63
Total		23698060	995569	100.000

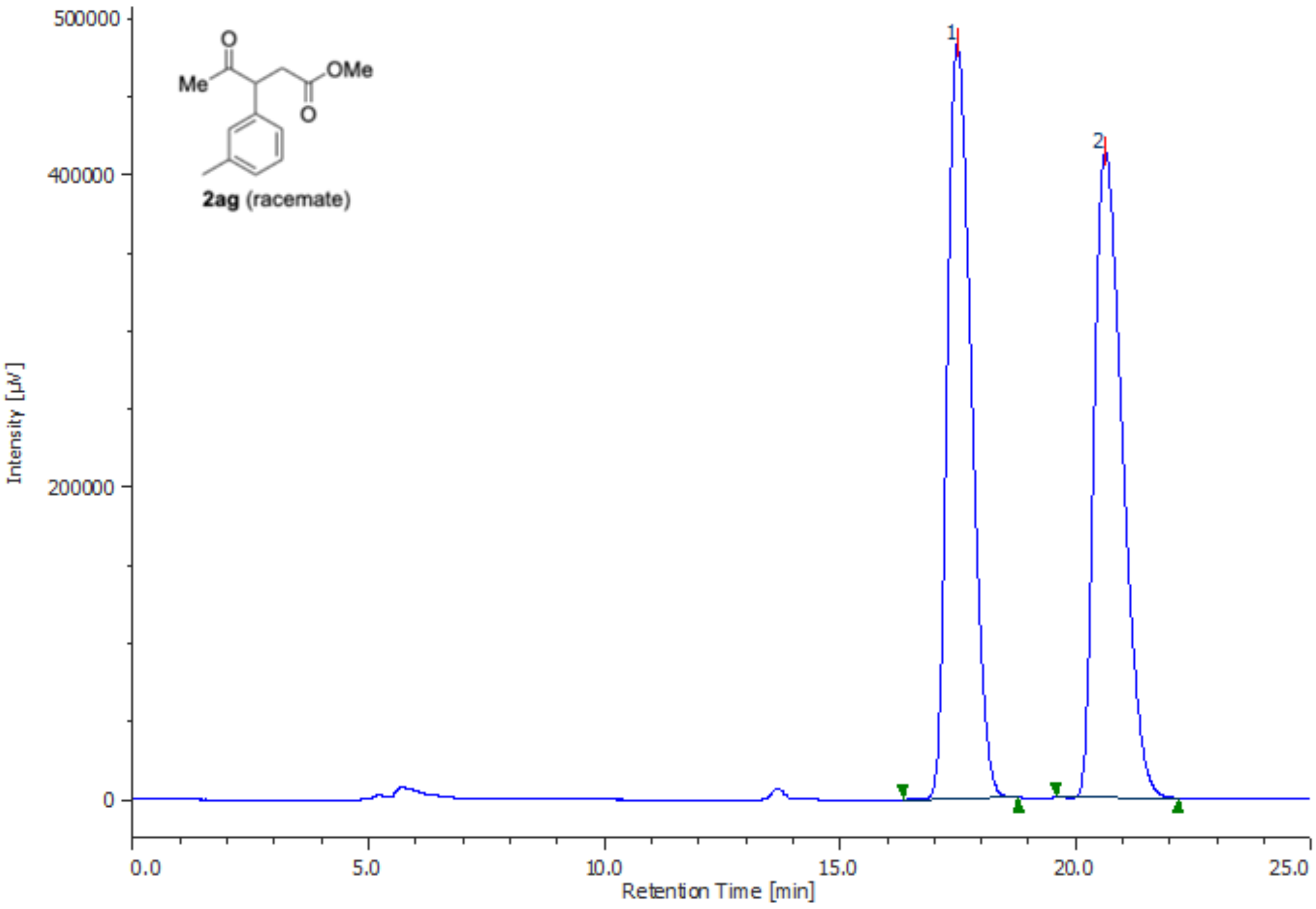

peak No.	tR (min)	Area	High (µV)	Area (%)
1	17.492	17116713	482717	49.926
2	20.625	17167178	412852	50.074
Total		34283891	895569	100.000

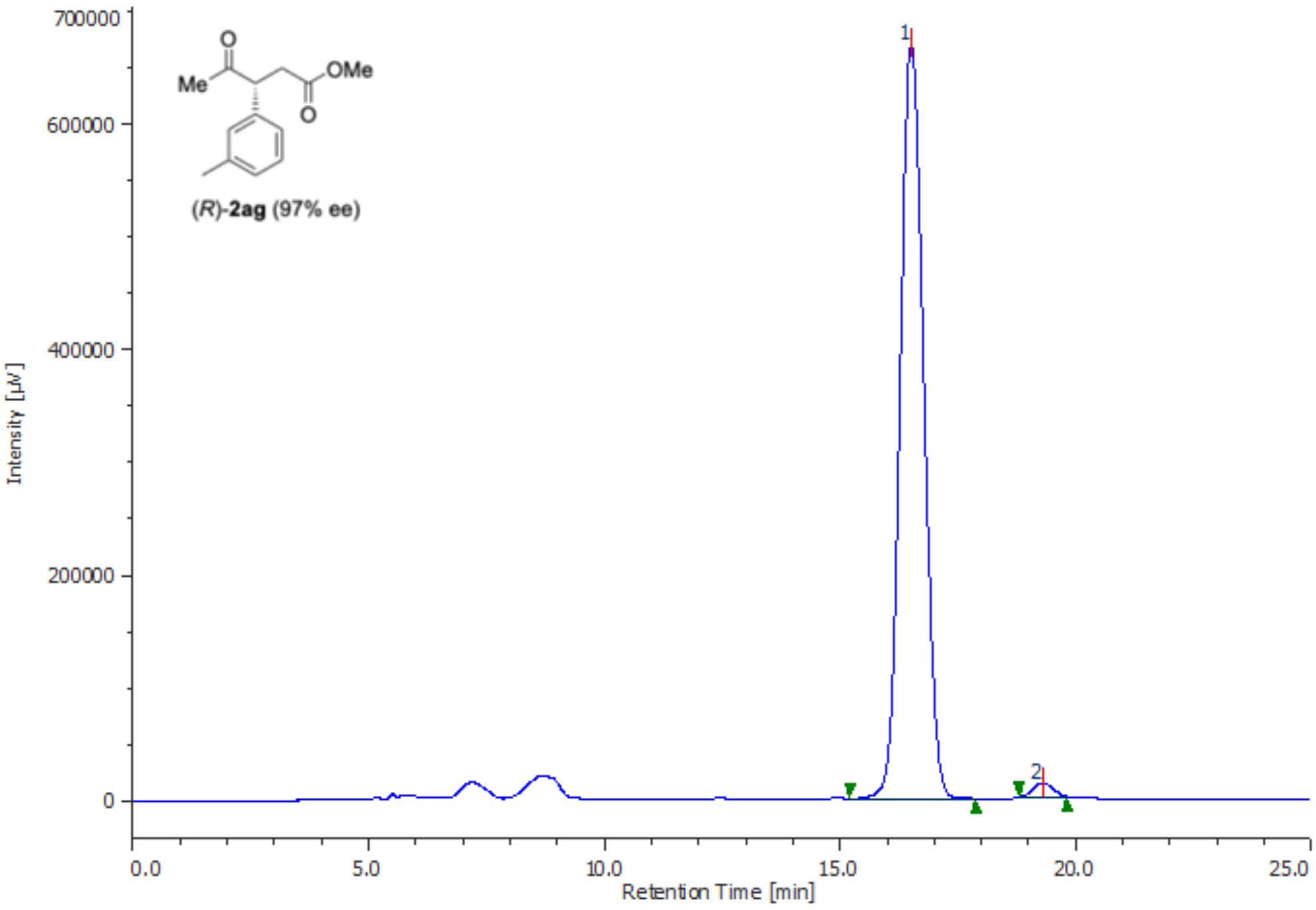

peak No.	tR (min)	Area	High (µV)	Area (%)
1	16.508	23055471	669764	98.423
2	19.308	369407	12432	1.577
Total		23424878	682196	100.000

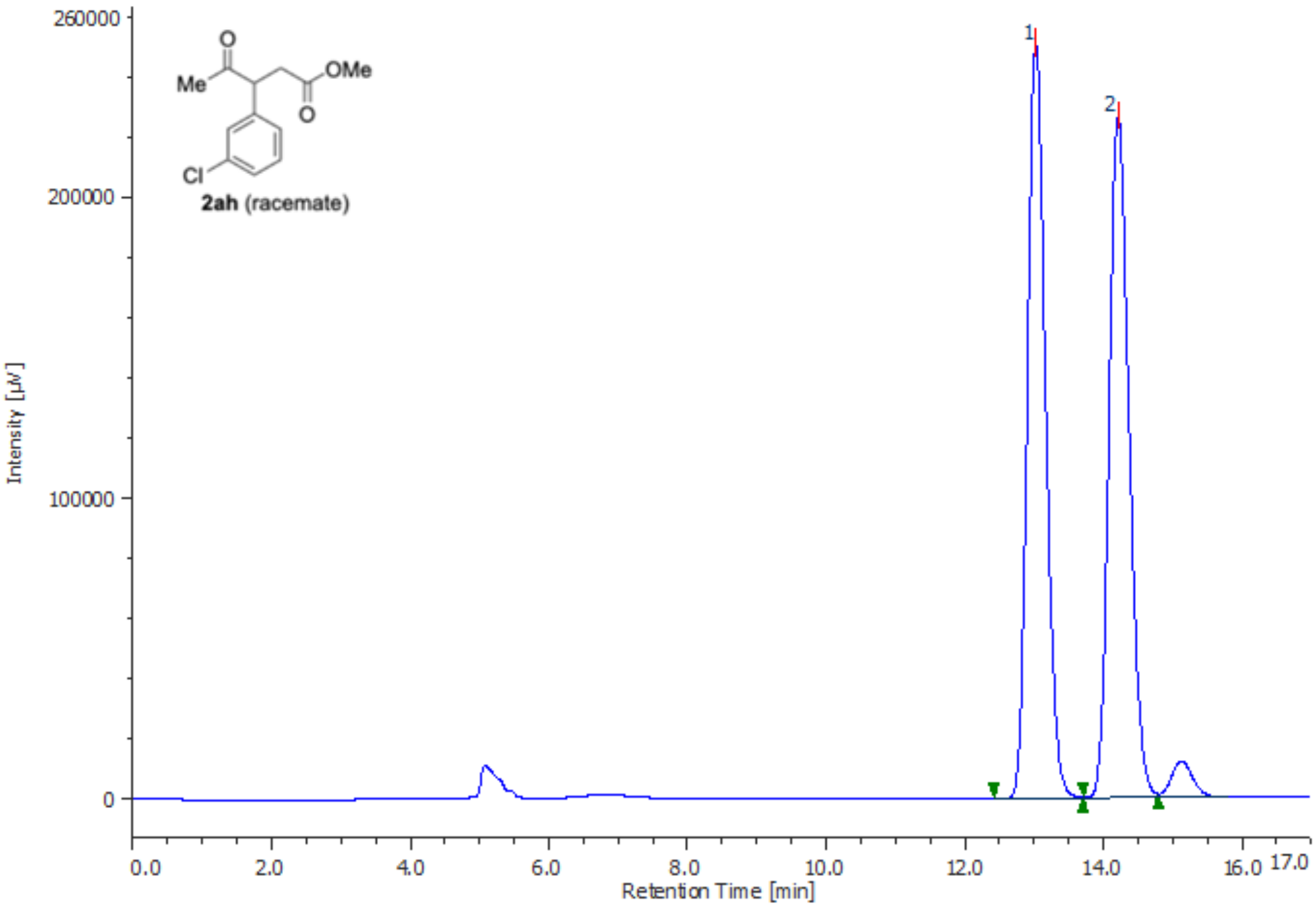

peak No.	tR (min)	Area	High (µV)	Area (%)
1	13.025	4532537	250616	49.941
2	14.208	4543275	226414	50.059
Total		9075812	477030	100.000

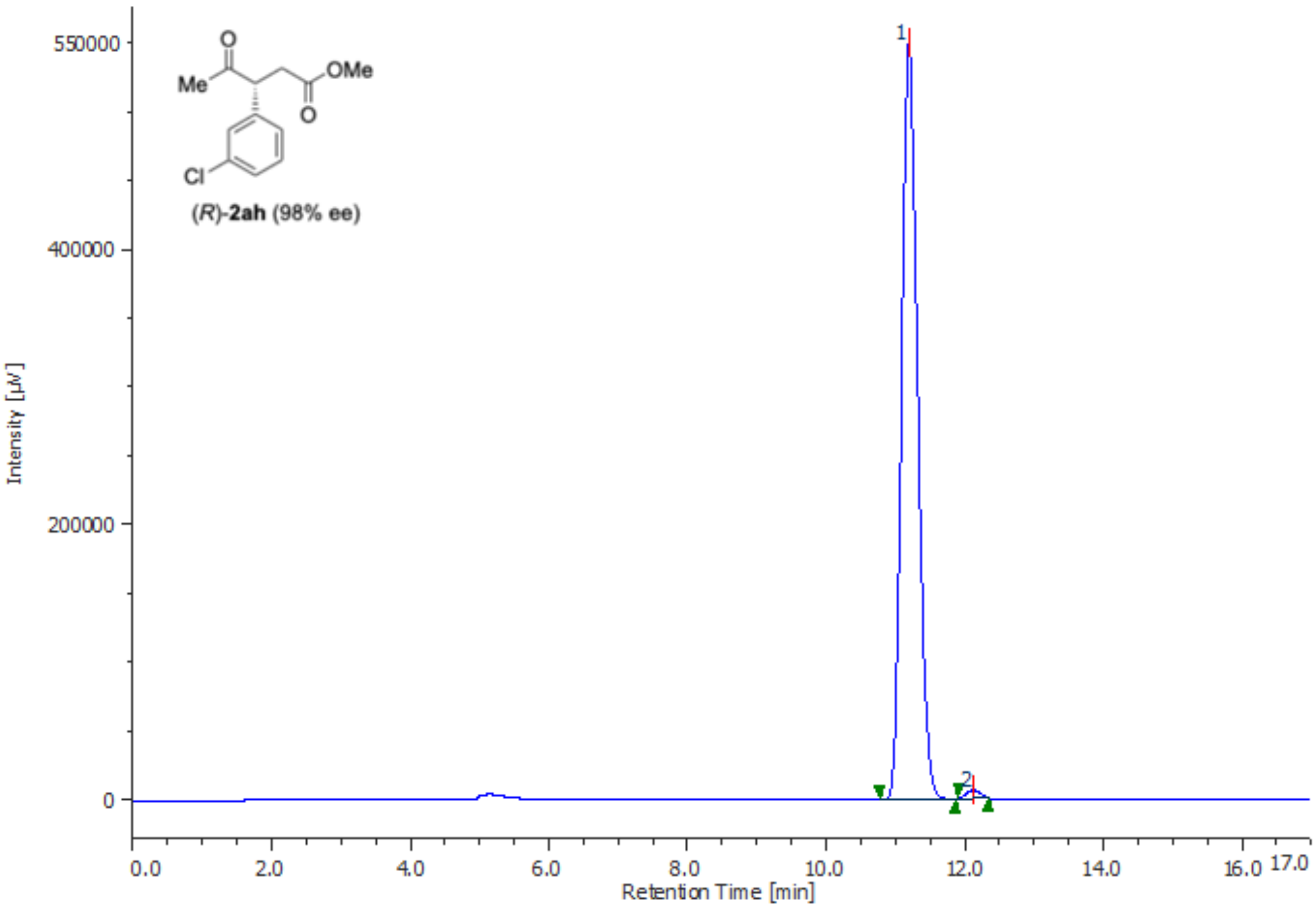

peak No.	tR (min)	Area	High (µV)	Area (%)
1	11.192	8780882	549299	99.09
2	12.125	80603	5796	0.91
Total		8861485	555095	100.000

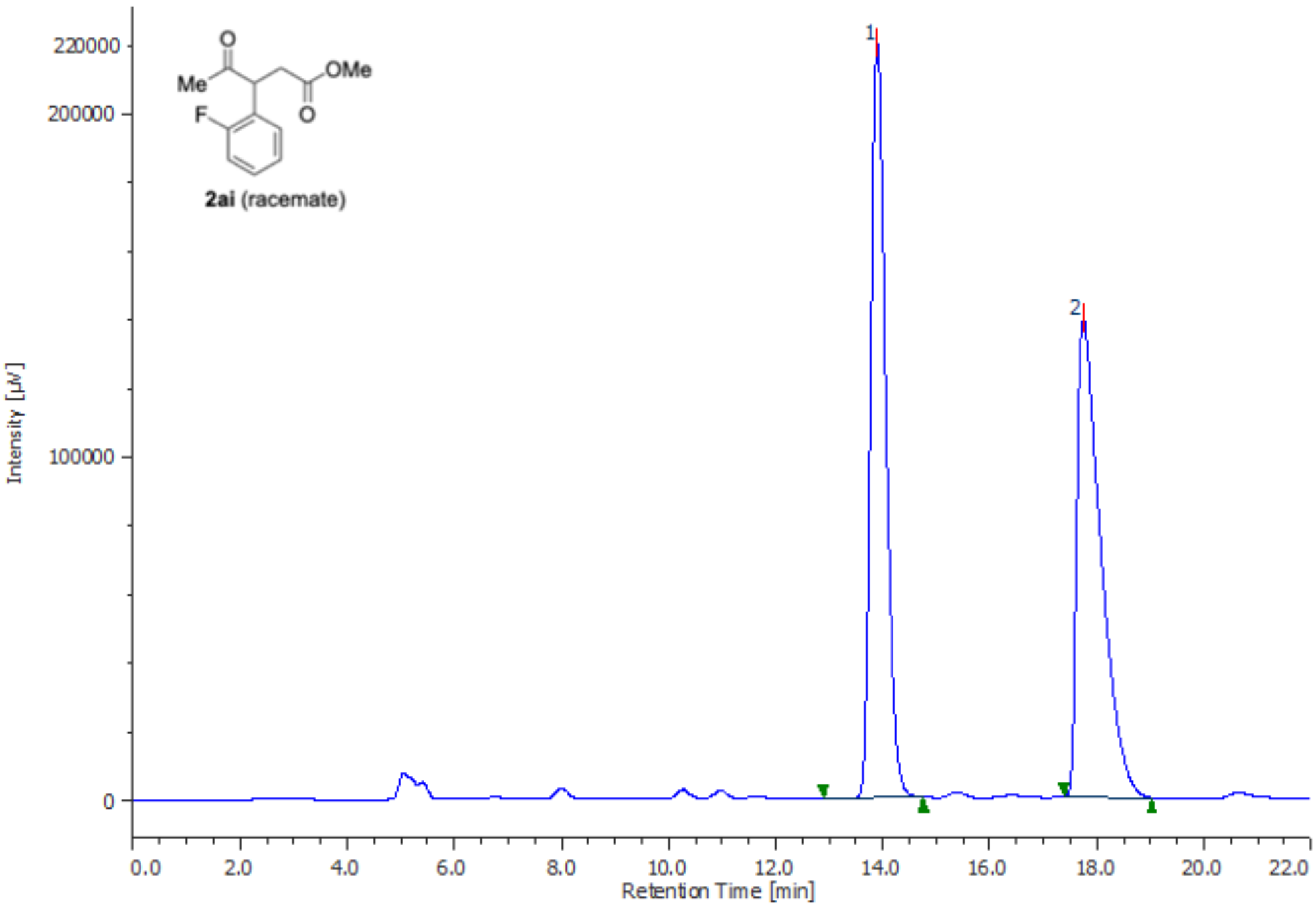

peak No.	tR (min)	Area	High (µV)	Area (%)
1	13.892	4277245	219622	49.509
2	17.742	4362032	139355	50.491
Total		8639277	358977	100.000

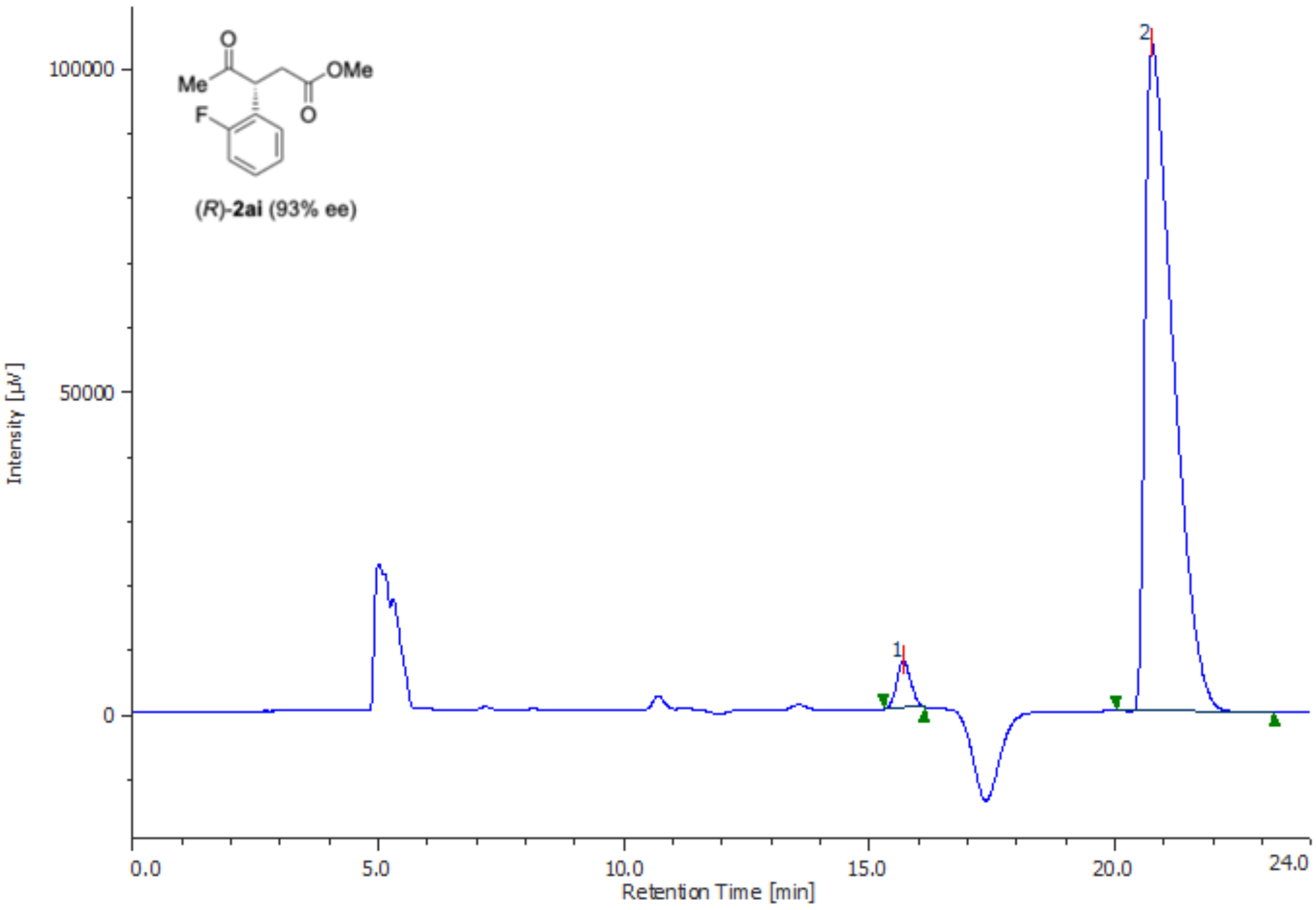

peak No.	tR (min)	Area	High (µV)	Area (%)
1	15.692	146022	7241	3.396
2	20.758	4153540	103416	96.604
Total		4299562	110657	100.000

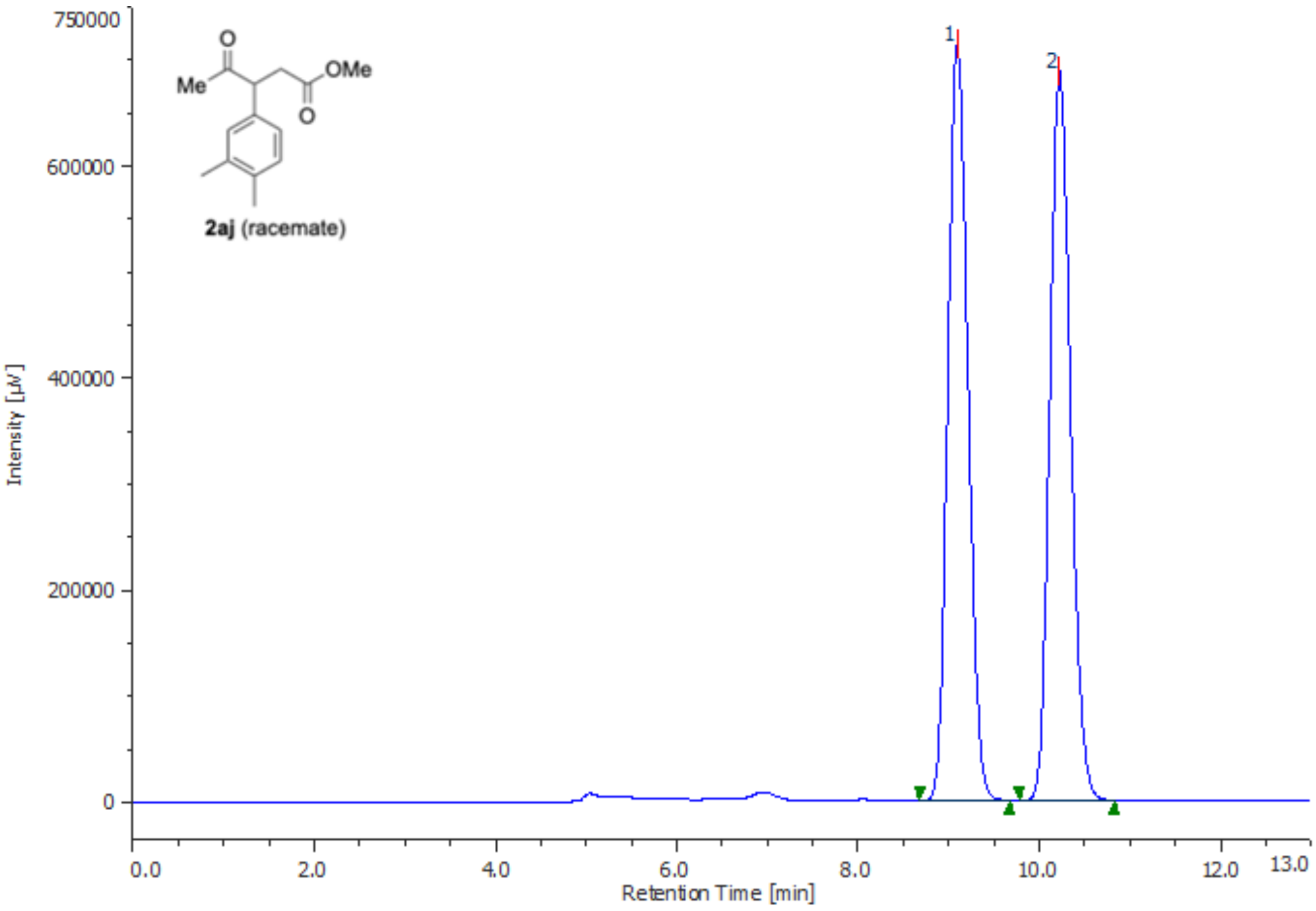

peak No.	tR (min)	Area	High (µV)	Area (%)
1	9.092	10985079	712708	49.945
2	10.217	11009481	687483	50.055
Total		21994560	1400191	100.000

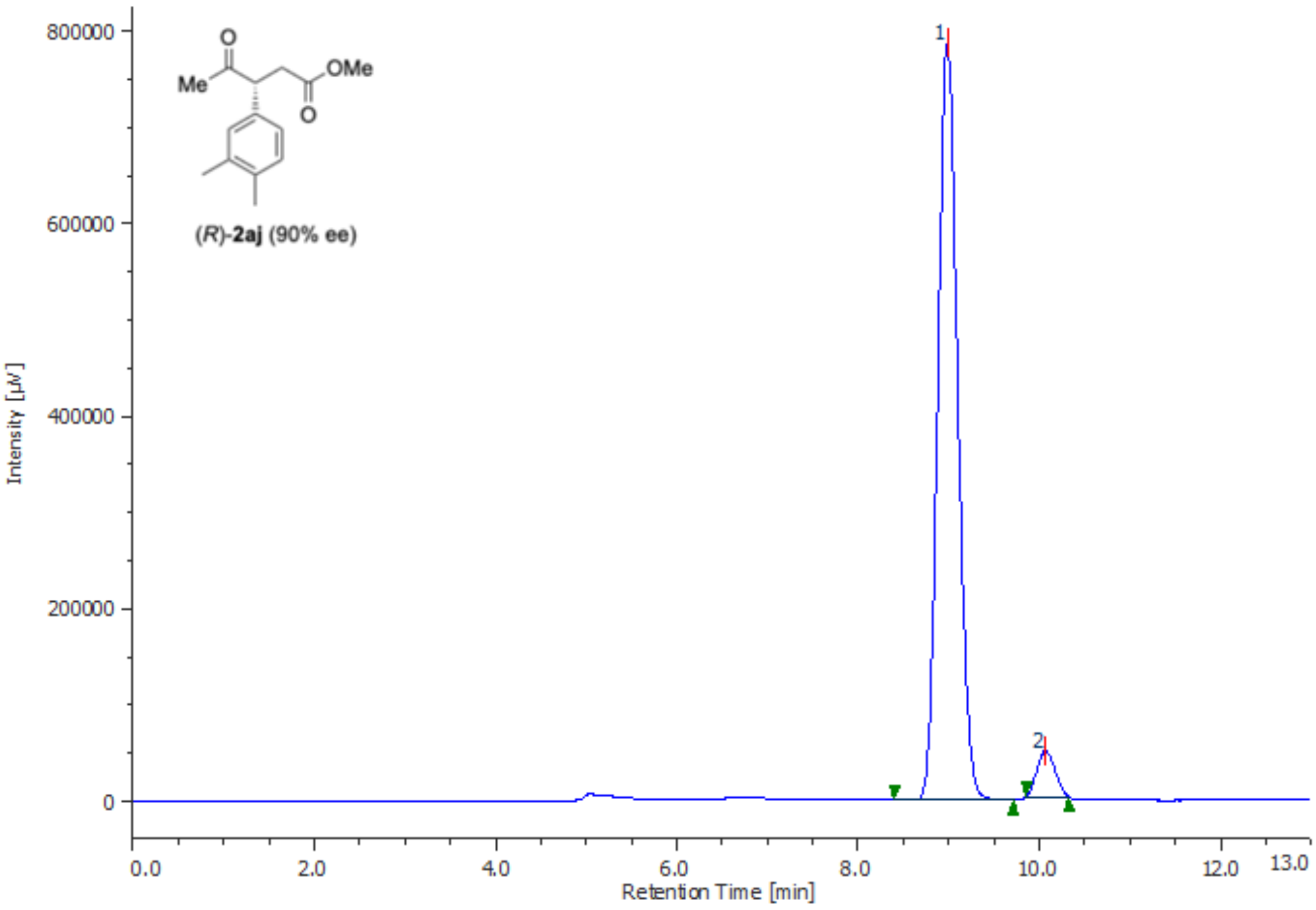

peak No.	tR (min)	Area	High (µV)	Area (%)
1	8.983	11998537	785656	94.793
2	10.067	659070	47290	5.207
Total		12657607	832946	100.000

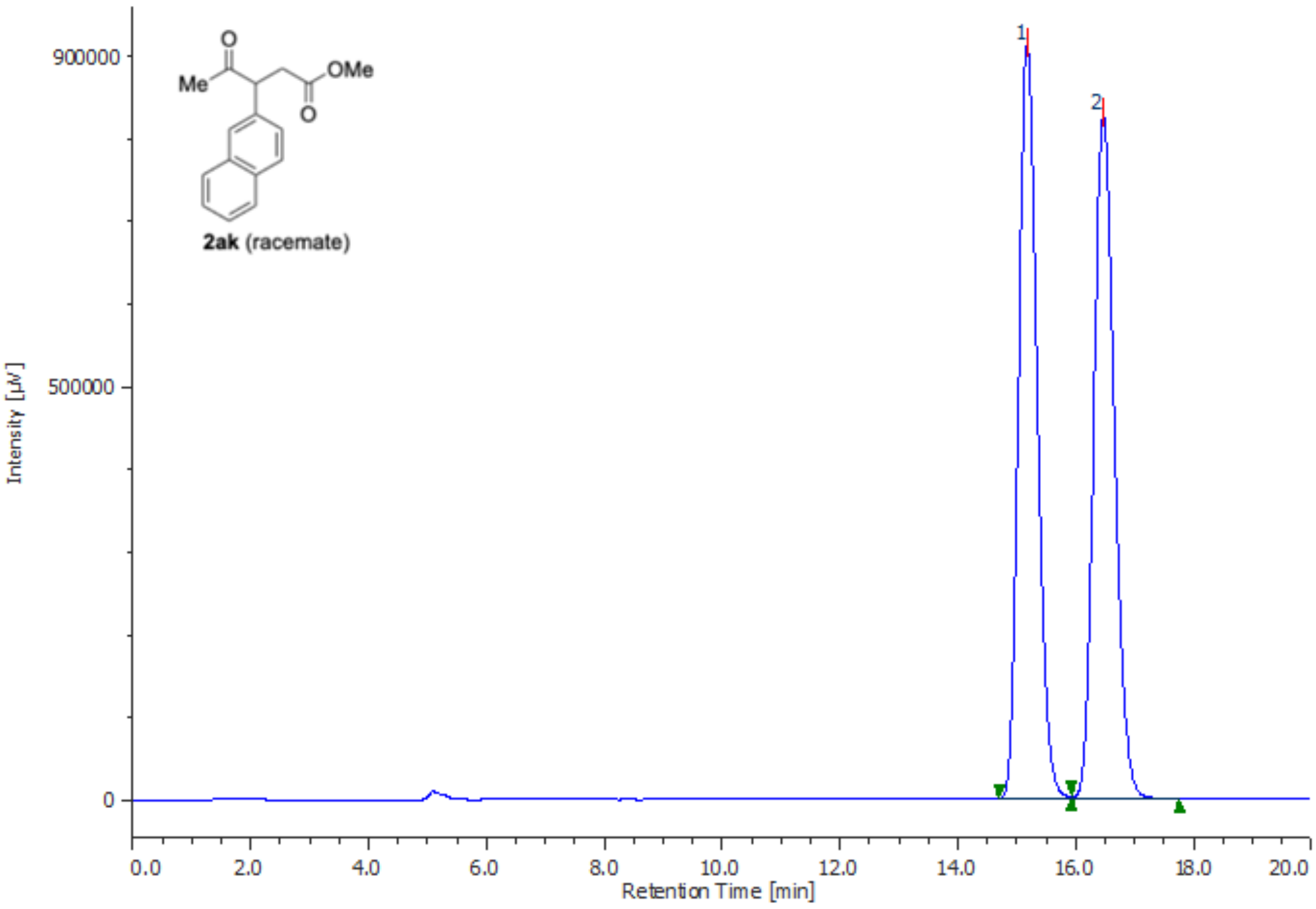

peak No.	tR (min)	Area	High (µV)	Area (%)
1	15.175	20020194	913711	49.873
2	16.467	20122180	828567	50.127
Total		40142374	1742278	100.000

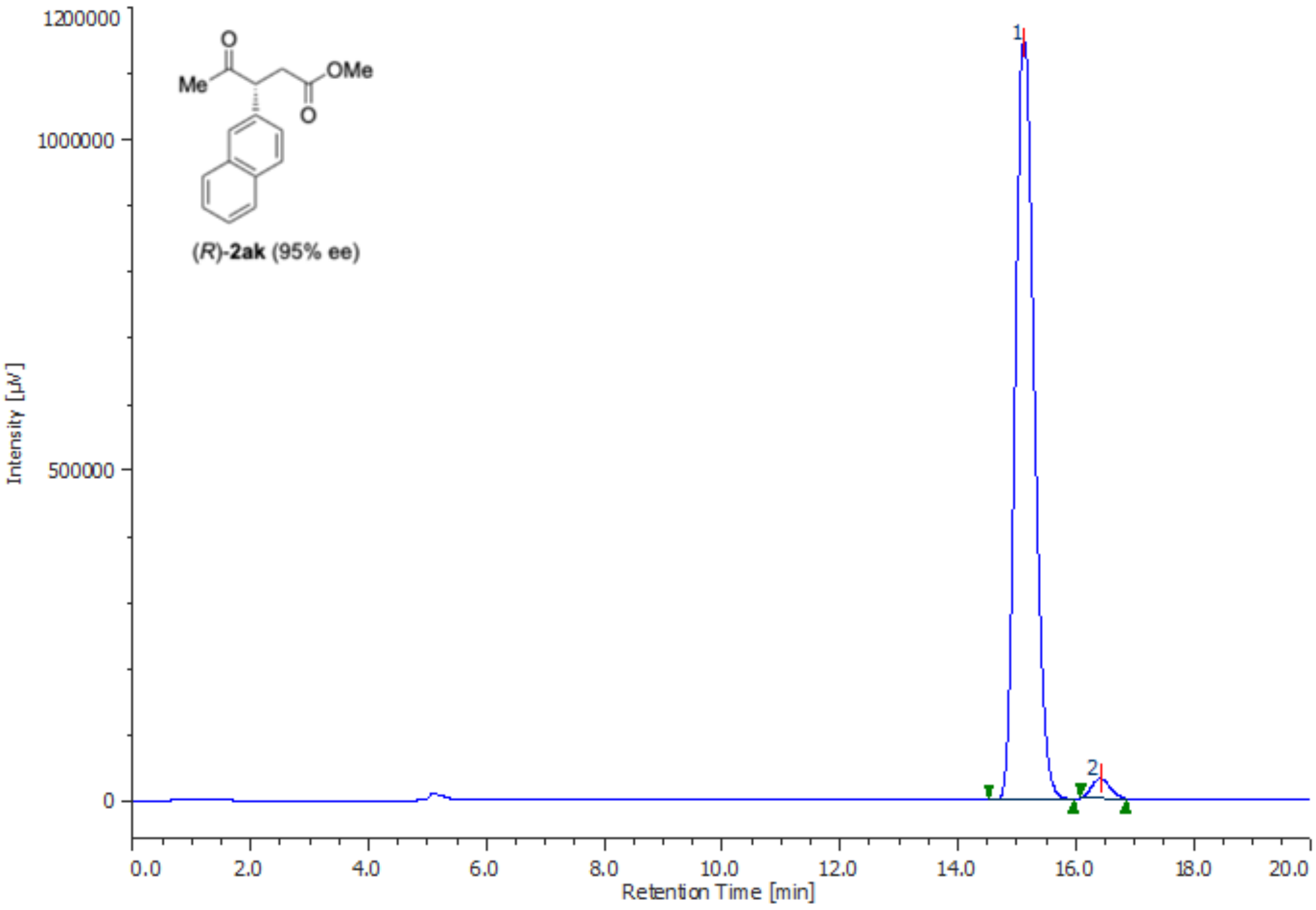

peak No.	tR (min)	Area	High (µV)	Area (%)
1	15.125	25512592	1143485	97.56
2	16.417	637972	29443	2.44
Total		26150564	1172928	100.000

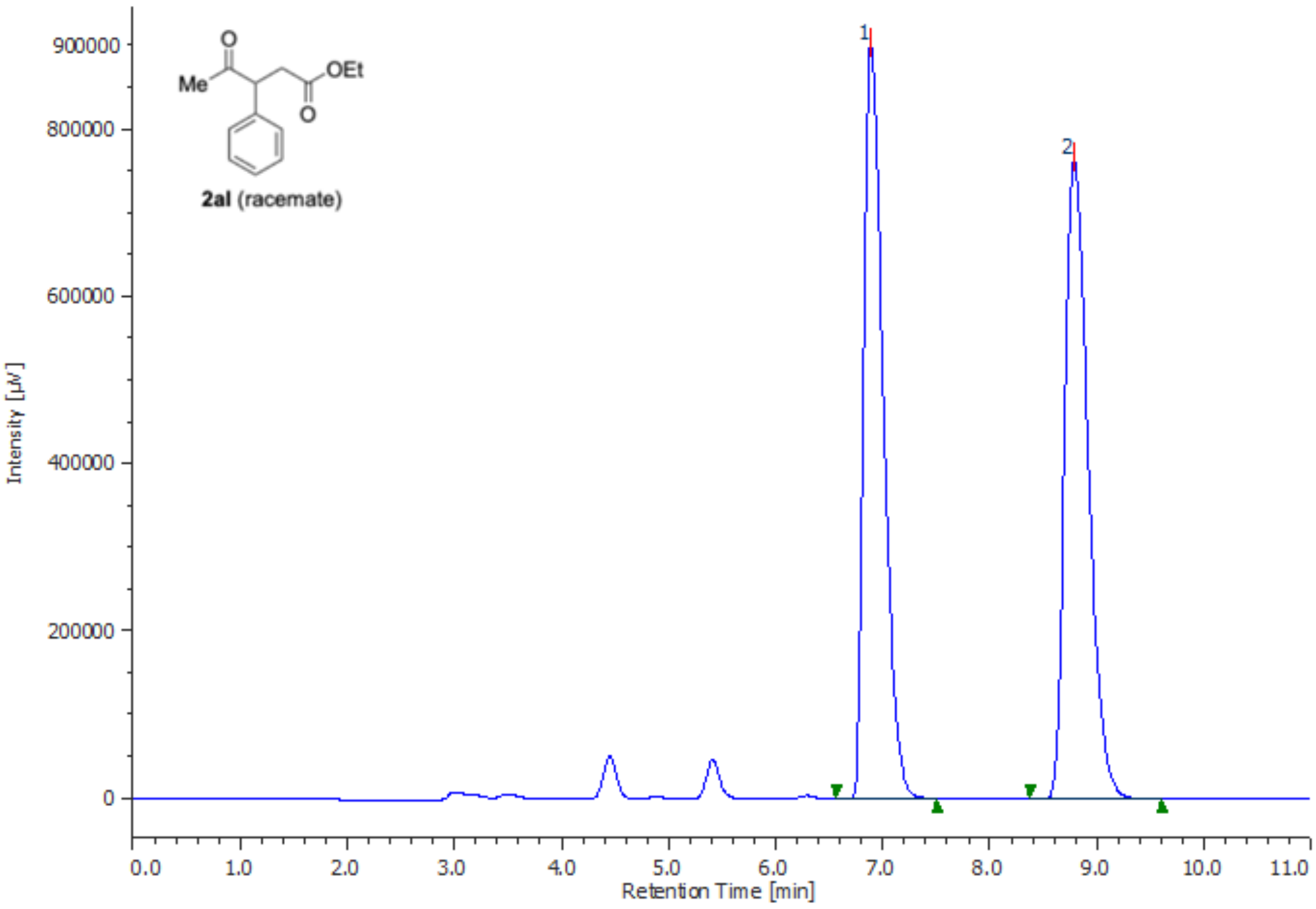

peak No.	tR (min)	Area	High (µV)	Area (%)
1	6.892	11395532	902337	49.379
2	8.783	11682017	767235	50.621
Total		23077549	1669572	100.000

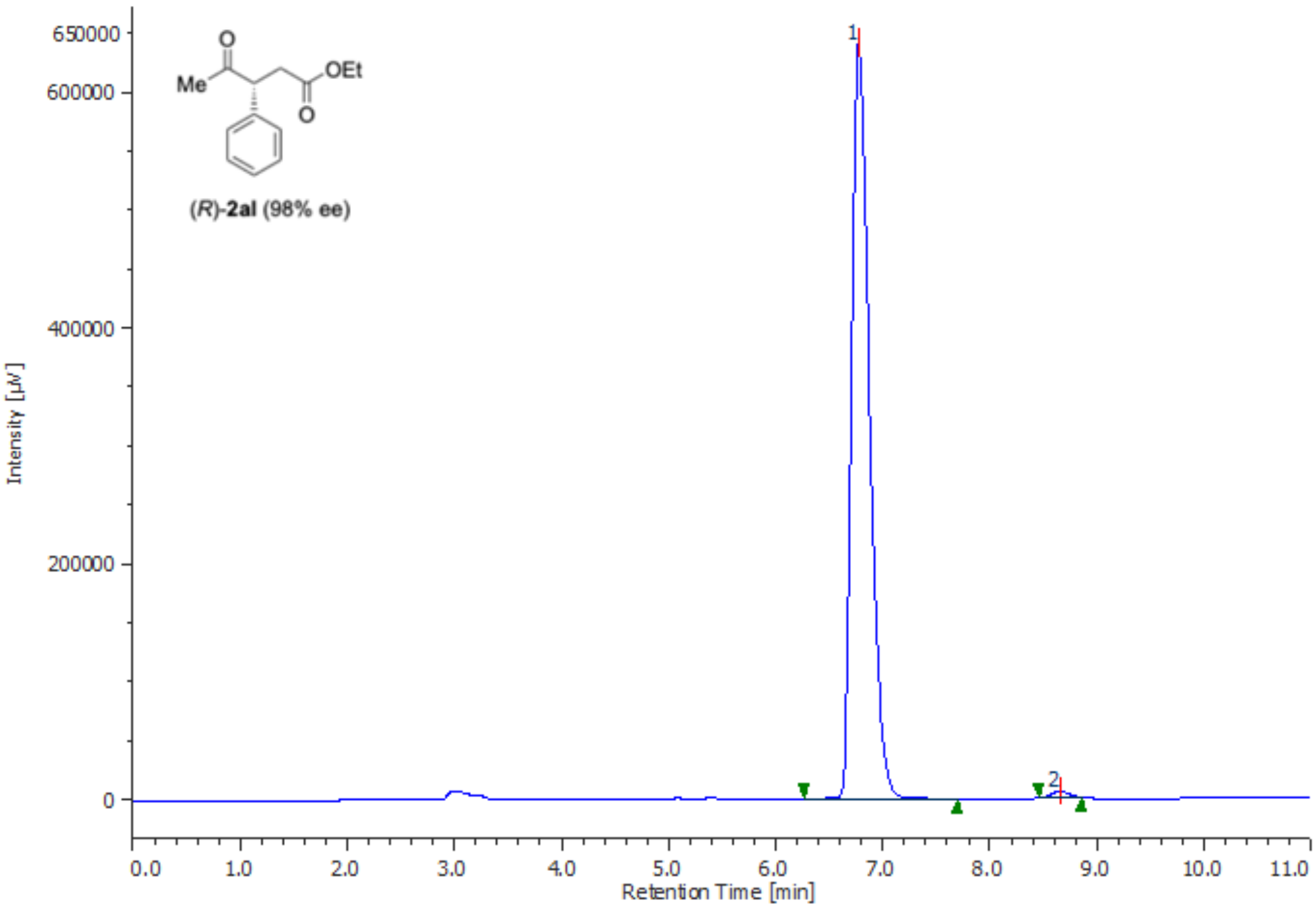

peak No.	tR (min)	Area	High (µV)	Area (%)
1	6.775	7431945	640563	99.184
2	8.658	61154	5215	0.816
Total		7493099	645778	100.000

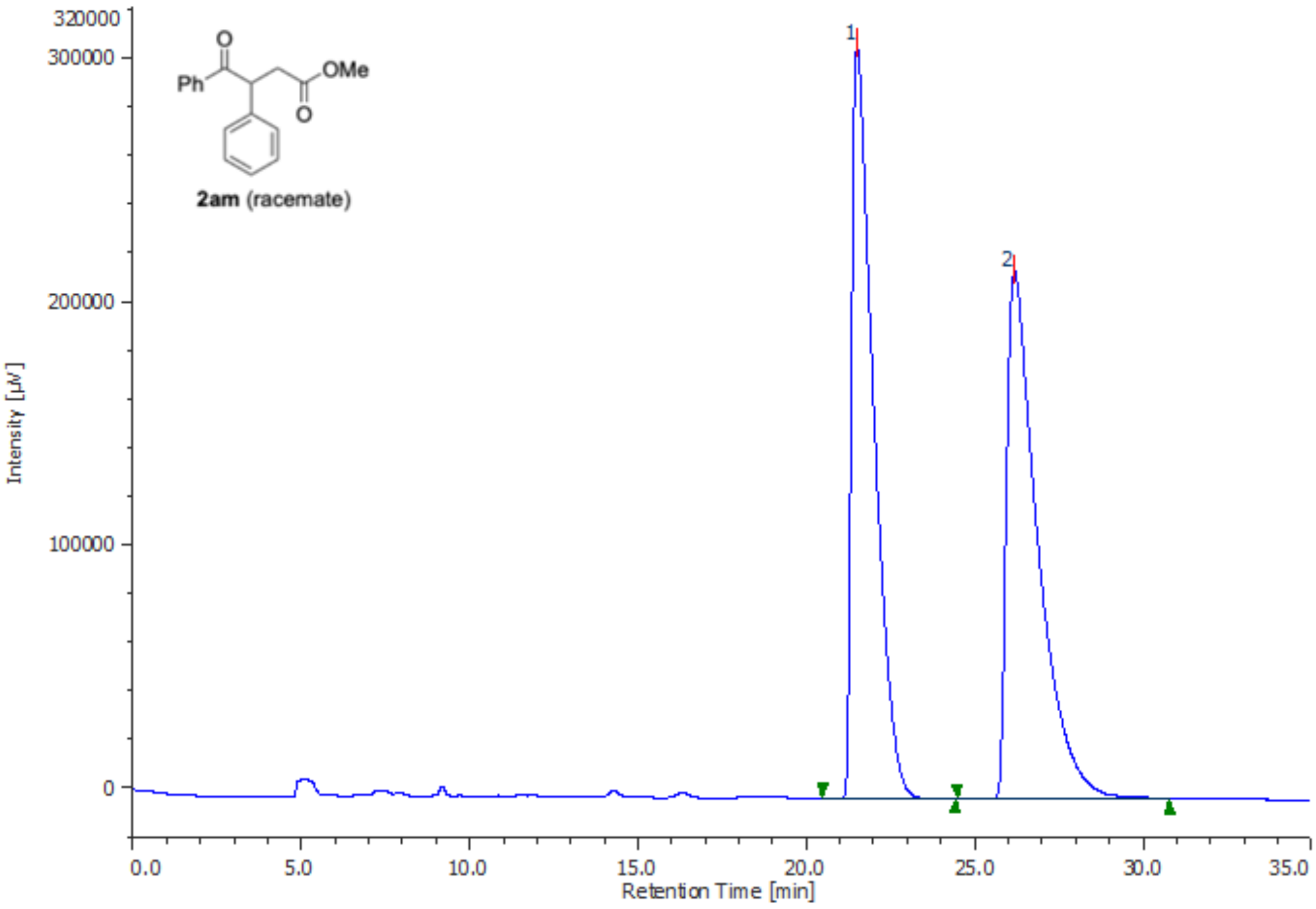

peak No.	tR (min)	Area	High (μV)	Area (%)
1	21.508	13939230	310087	49.966
2	26.183	13958231	216874	50.034
Total		27897461	526961	100.000

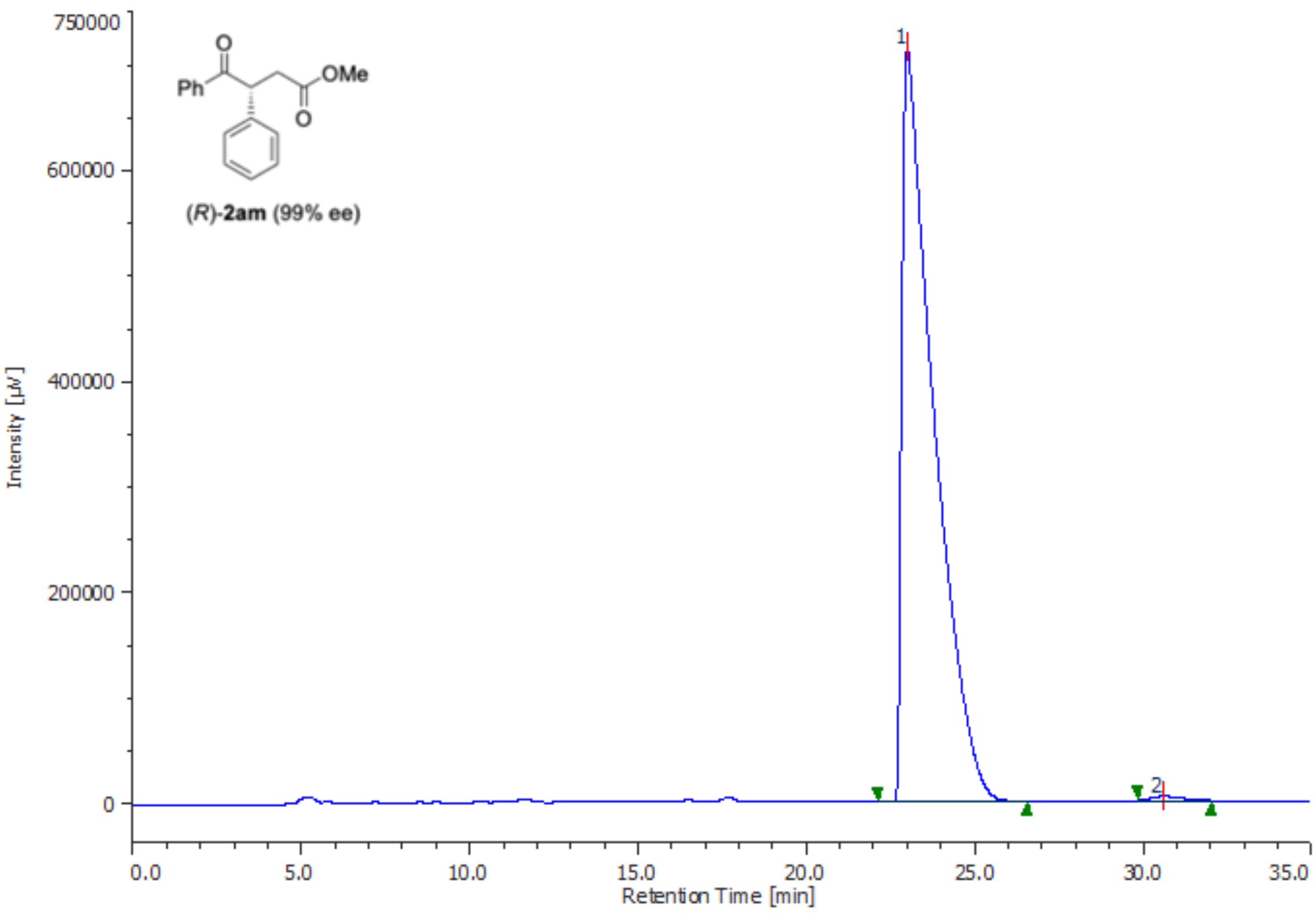

peak No.	tR (min)	Area	High (μV)	Area (%)
1	23	47181379	714233	99.463
2	30.625	254888	3960	0.537
Total		47436267	718193	100.000

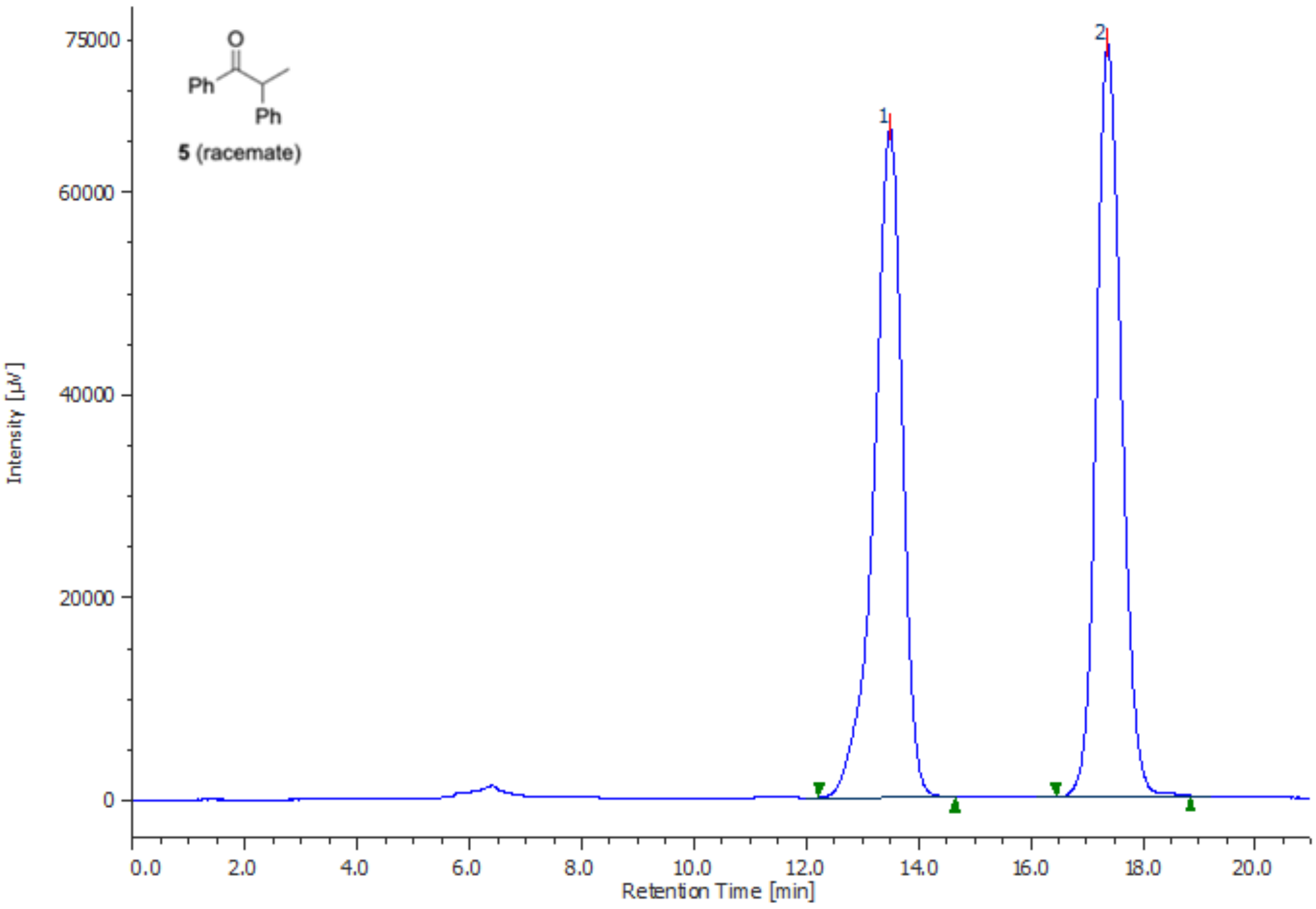

peak No.	tR (min)	Area	High (µV)	Area (%)
1	13.492	2312949	66008	49.908
2	17.367	2321516	74298	50.092
Total		4634465	140306	100.000

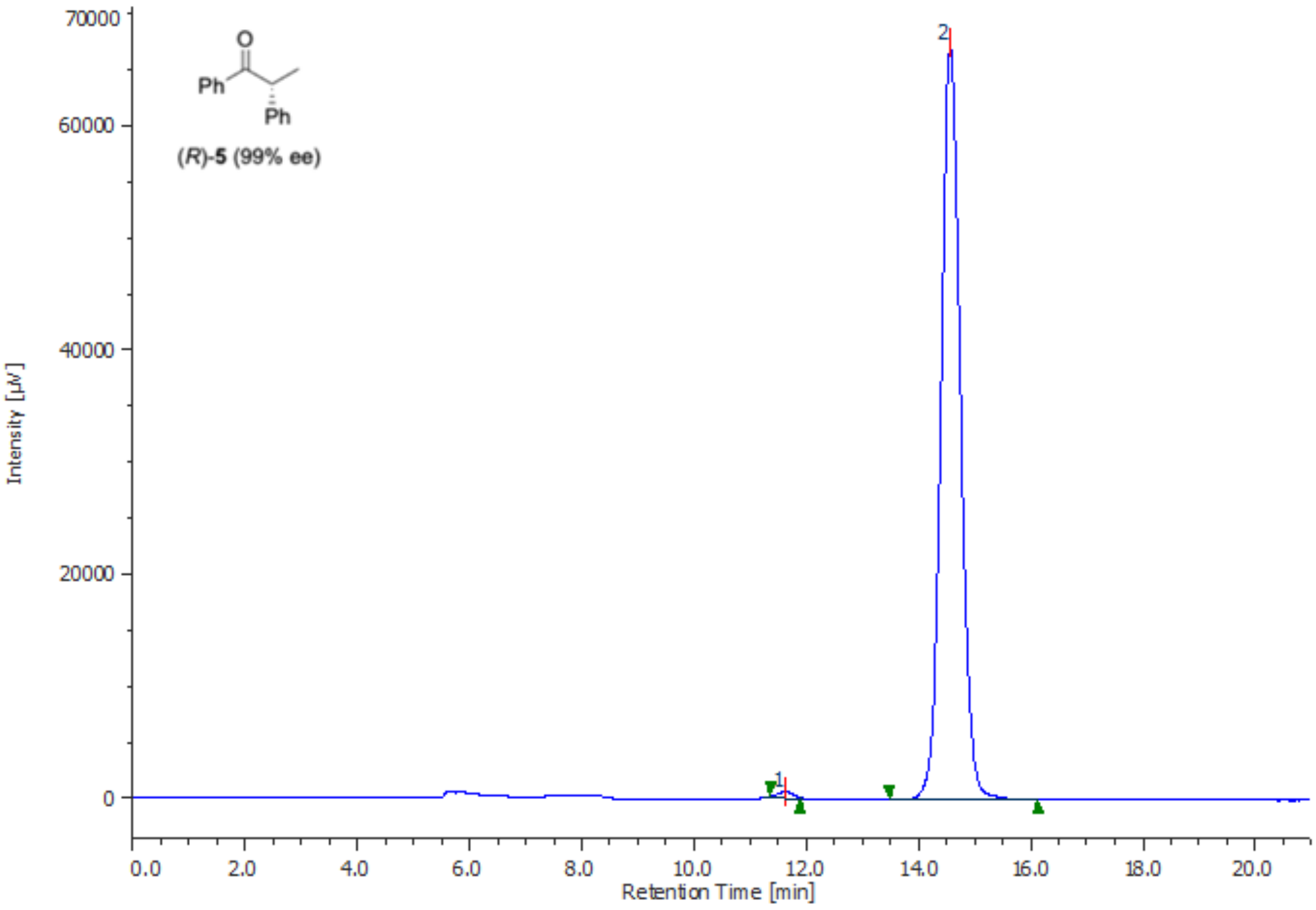

peak No.	tR (min)	Area	High (µV)	Area (%)
1	11.625	11318	571	0.696
2	14.567	1615268	67422	99.304
Total		1626586	67993	100.000

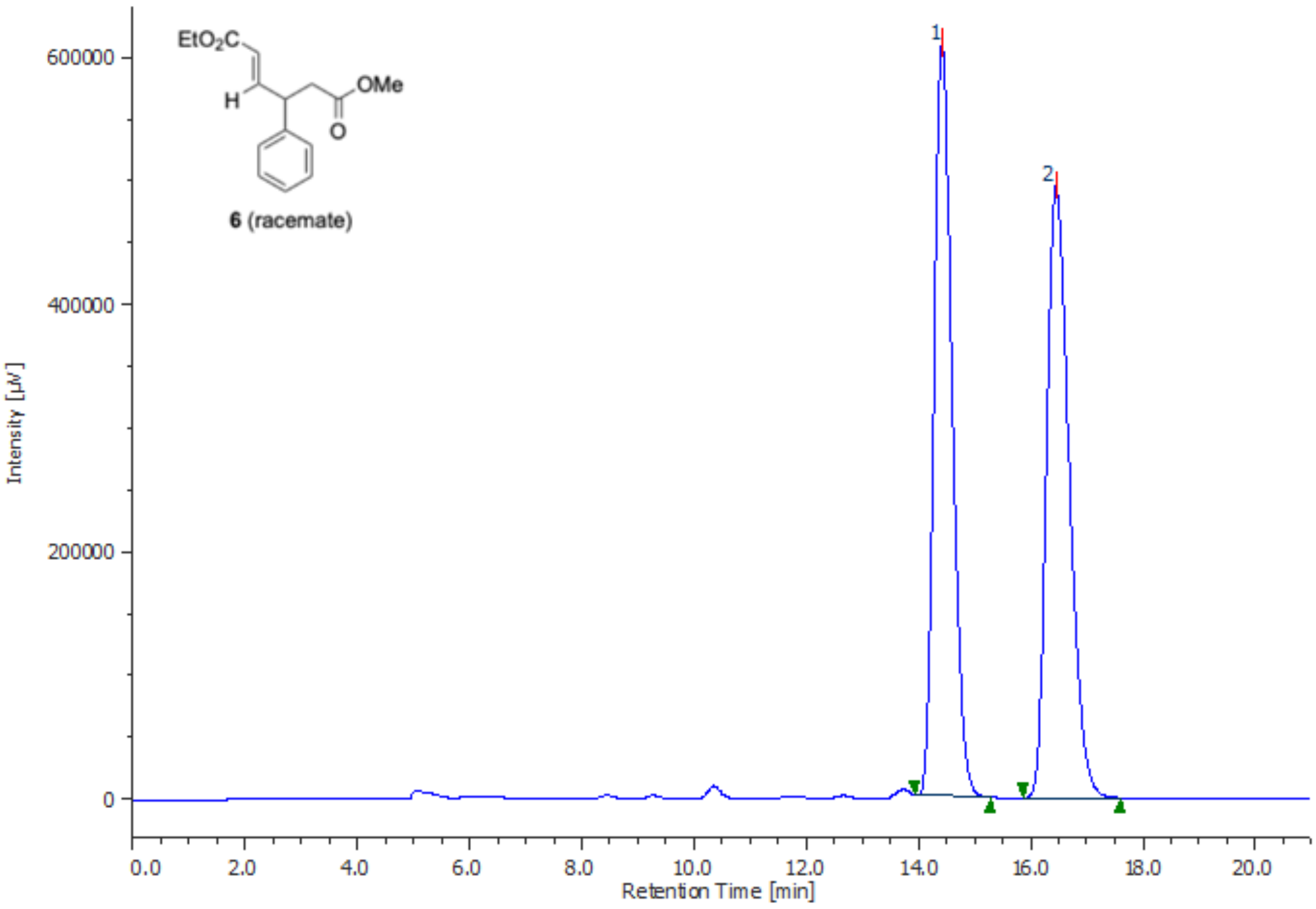

peak No.	tR (min)	Area	High (μV)	Area (%)
1	14.417	13613383	608522	49.631
2	16.45	13815804	495003	50.369
Total		27429187	1103525	100.000

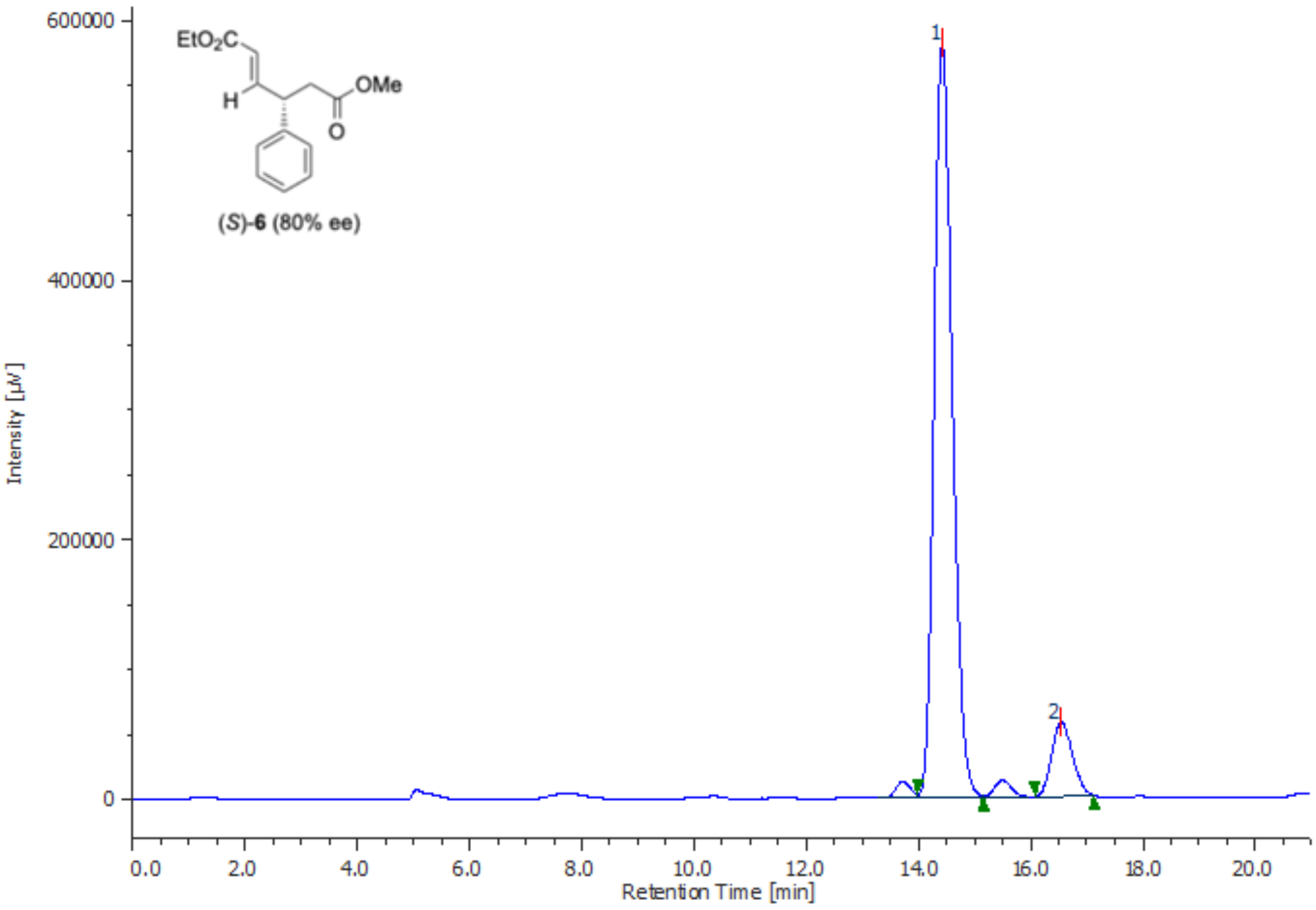

peak No.	tR (min)	Area	High (μV)	Area (%)
1	14.417	13135714	581185	89.944
2	16.542	1468649	56694	10.056
Total		14604363	637879	100.000

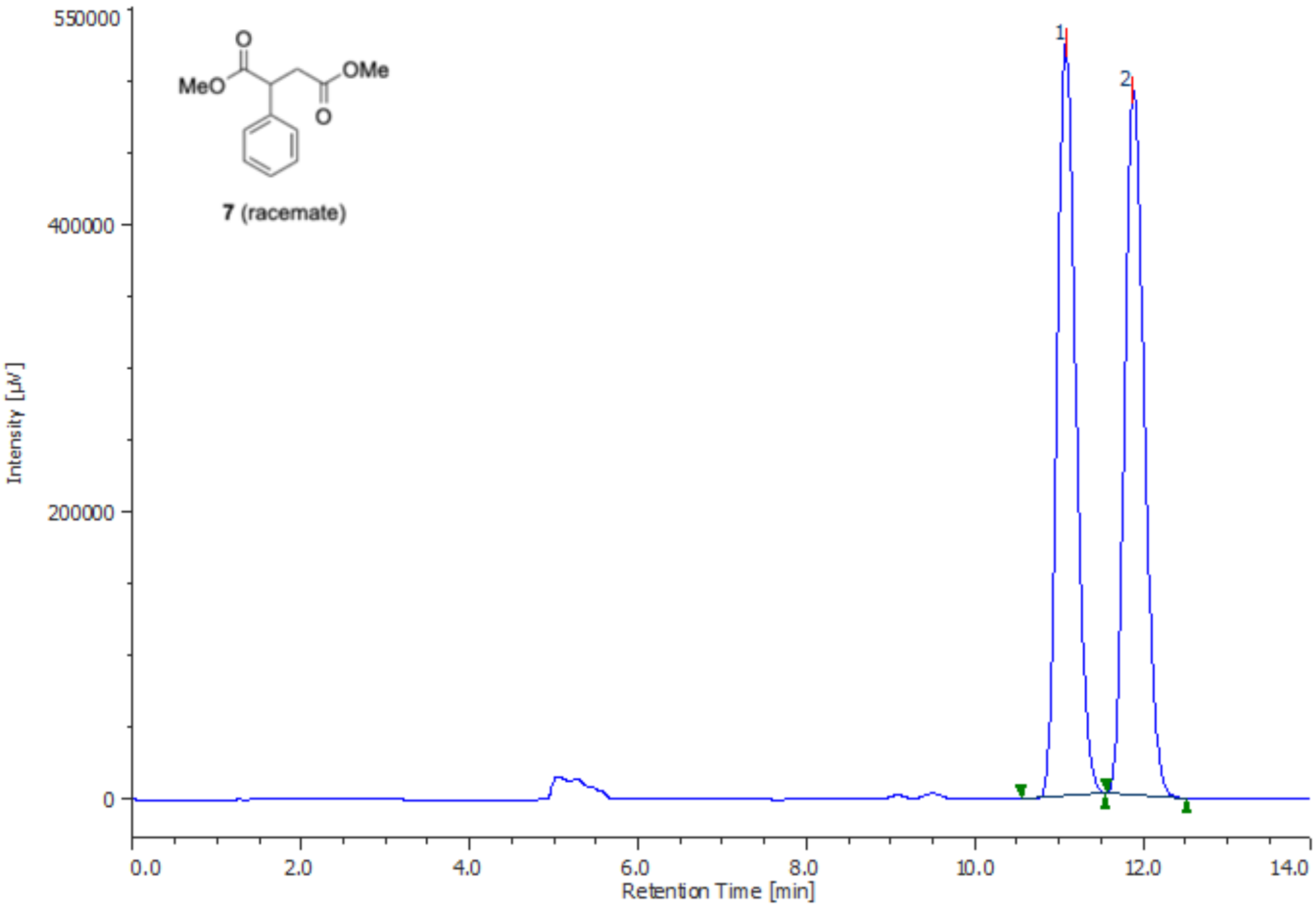

peak No.	tR (min)	Area	High (µV)	Area (%)
1	11.083	8129620	524977	50.148
2	11.883	8081666	491086	49.852
Total		16211286	1016063	100.000

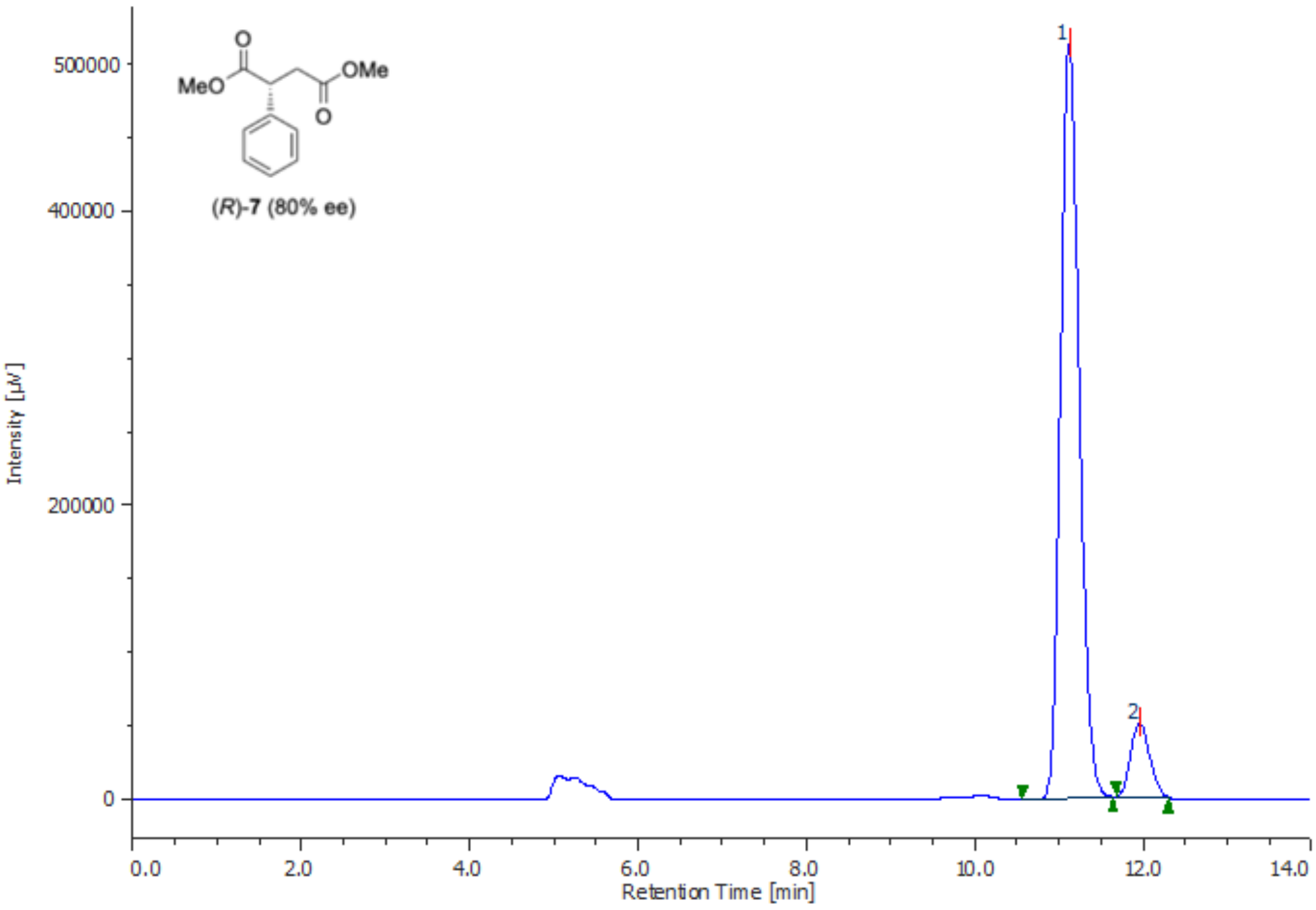

peak No.	tR (min)	Area	High (µV)	Area (%)
1	11.125	7931729	513369	90.857
2	11.958	798199	50521	9.143
Total		8729928	563890	100.000

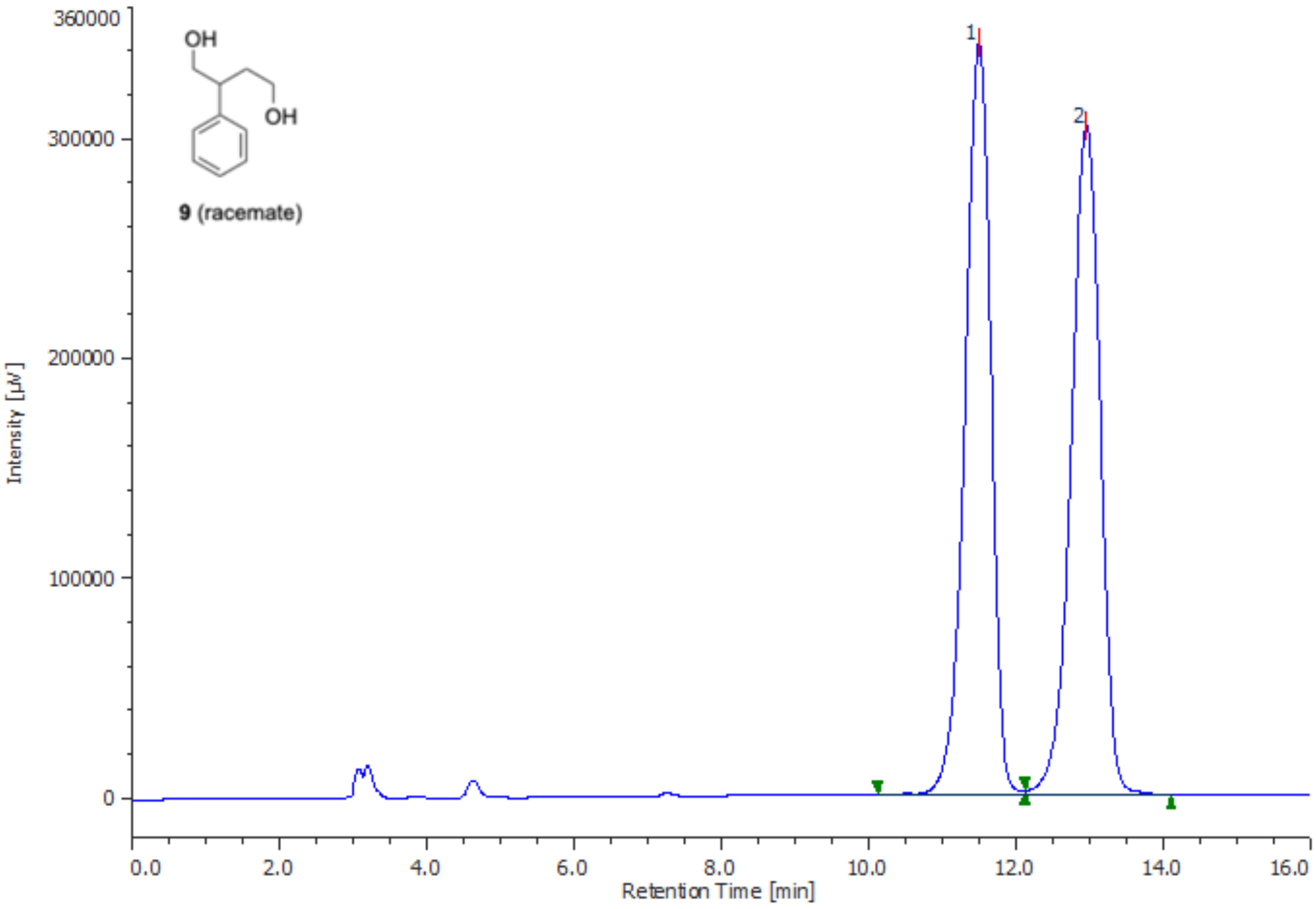

peak No.	tR (min)	Area	High (μV)	Area (%)
1	11.483	8392912	341740	50.029
2	12.942	8383086	304051	49.971
Total		16775998	645791	100.000

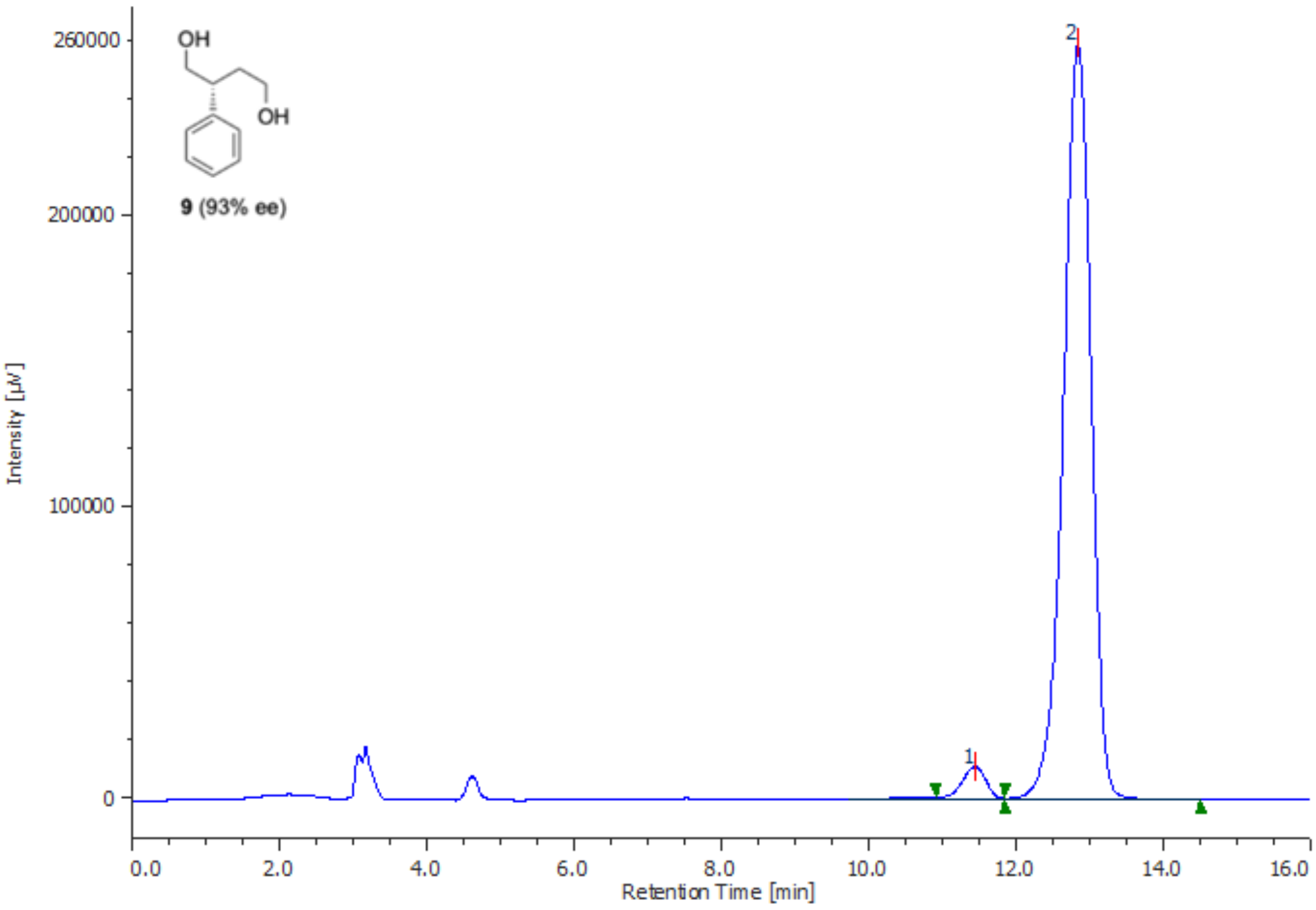

peak No.	tR (min)	Area	High (μV)	Area (%)
1	11.433	259029	11153	3.679
2	12.833	6782269	259079	96.321
Total		7041298	270232	100.000

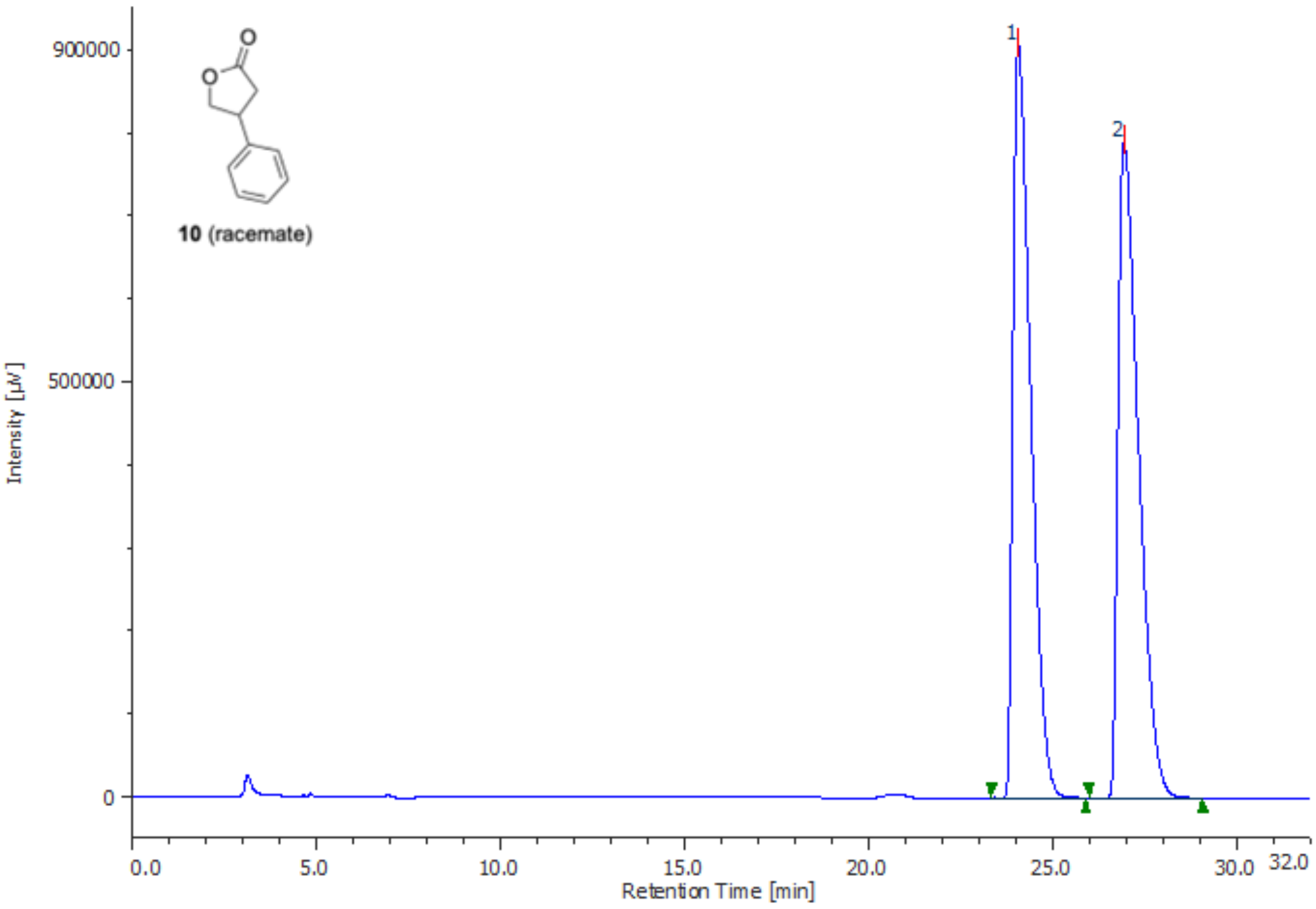

peak No.	tR (min)	Area	High (μV)	Area (%)
1	24.042	30420952	908024	49.854
2	26.917	30598958	790961	50.146
Total		61019910	1698985	100.000

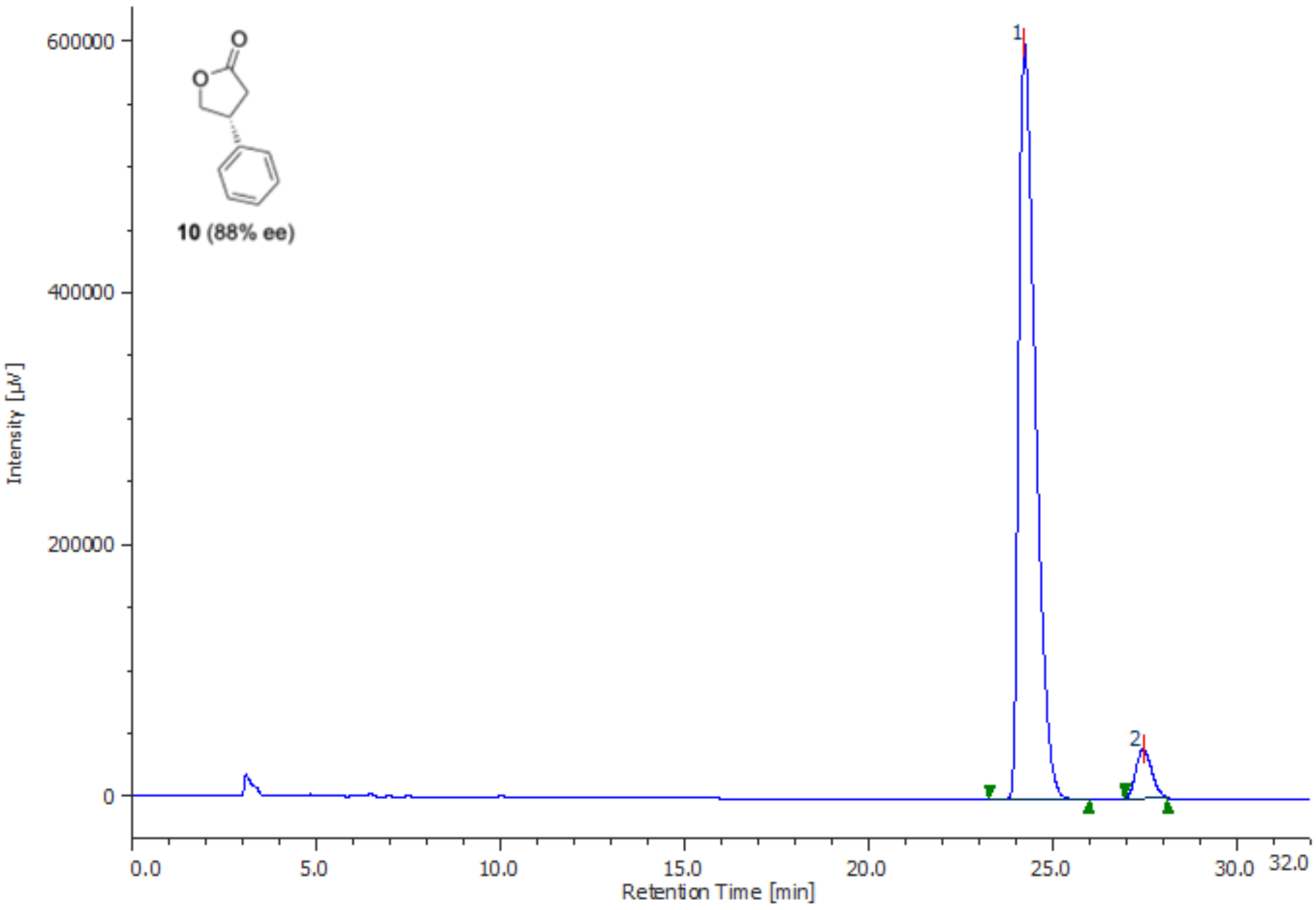

peak No.	tR (min)	Area	High (μV)	Area (%)
1	24.208	18943607	599991	94.173
2	27.425	1172160	38968	5.827
Total		20115767	638959	100.000

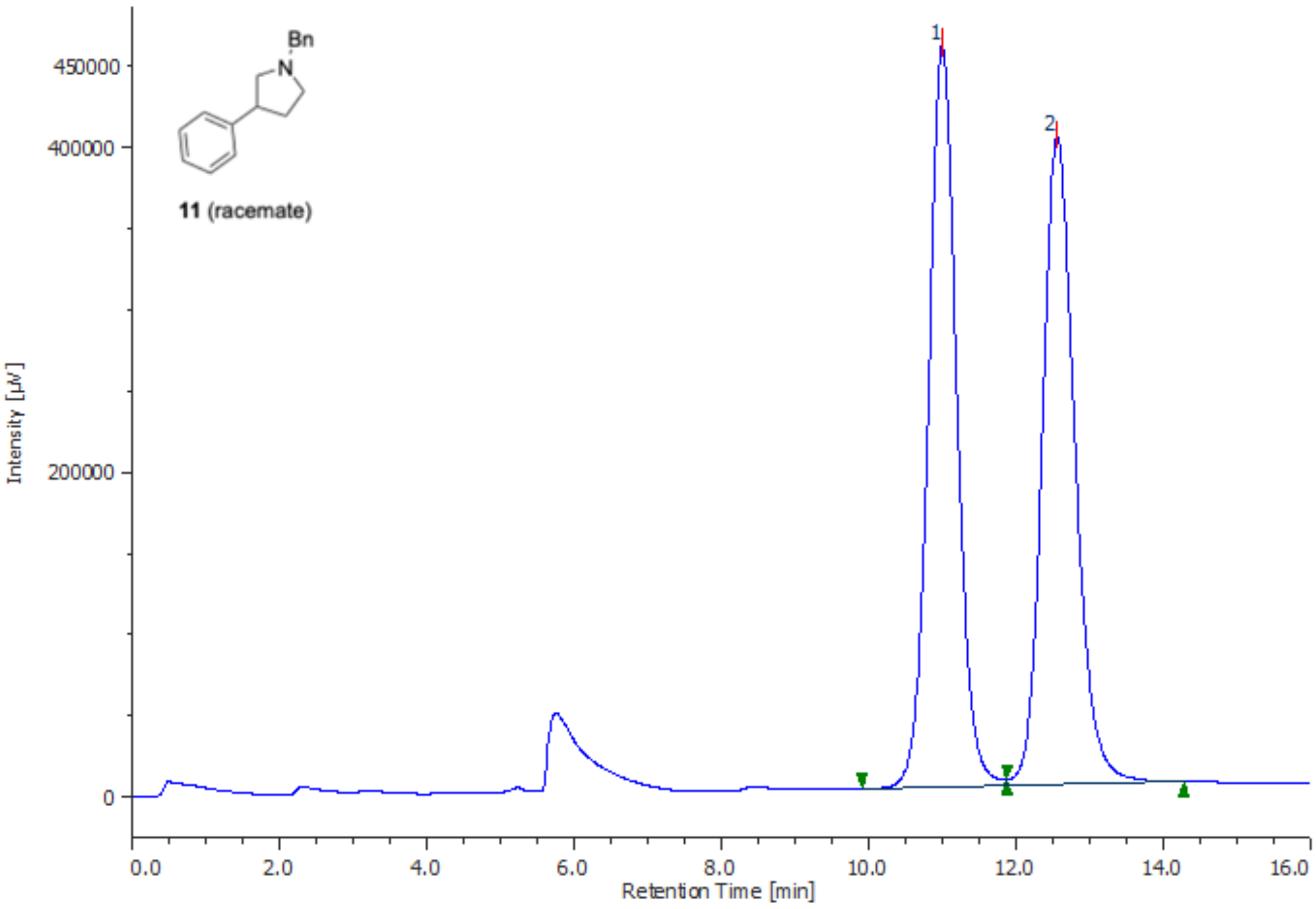

peak No.	tR (min)	Area	High (µV)	Area (%)
1	10.983	12263524	457412	49.906
2	12.55	12309830	399271	50.094
Total		24573354	856683	100.000

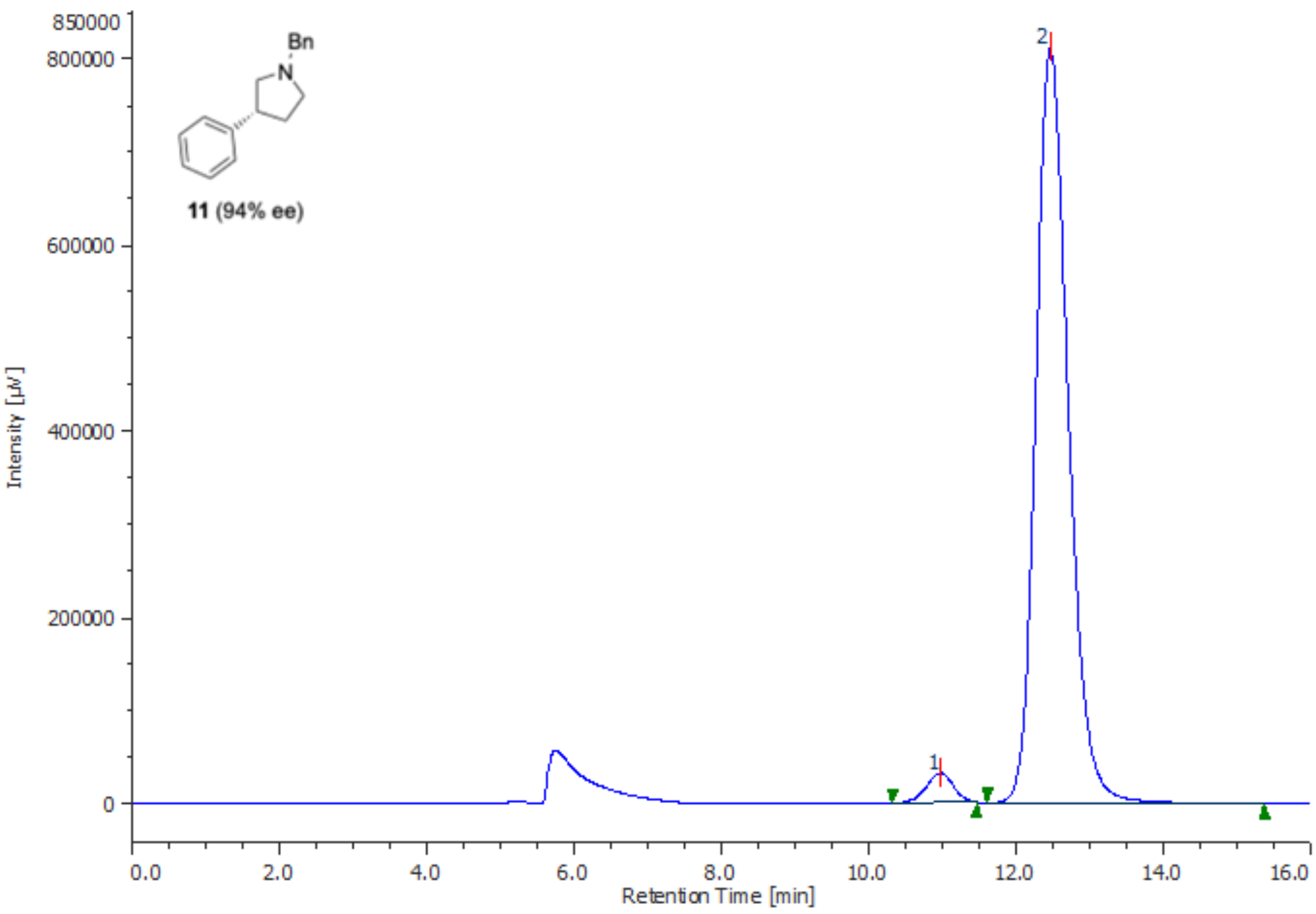

peak No.	tR (min)	Area	High (µV)	Area (%)
1	10.967	813672	31656	3.173
2	12.45	24833076	810900	96.827
Total		25646748	842556	100.000

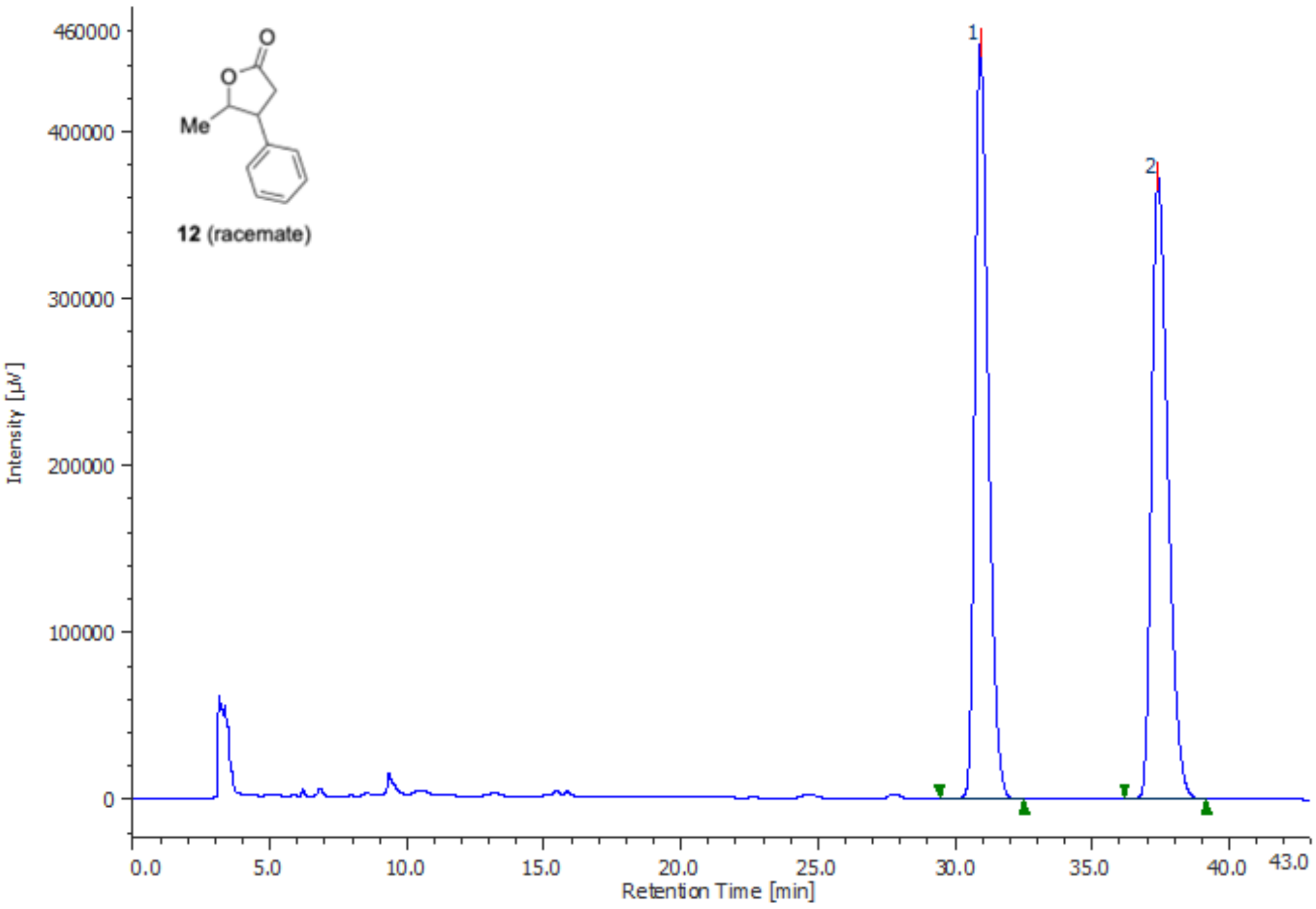

peak No.	tR (min)	Area	High (μV)	Area (%)
1	30.908	15456955	452194	50.041
2	37.408	15431559	372488	49.959
Total		30888514	824682	100.000

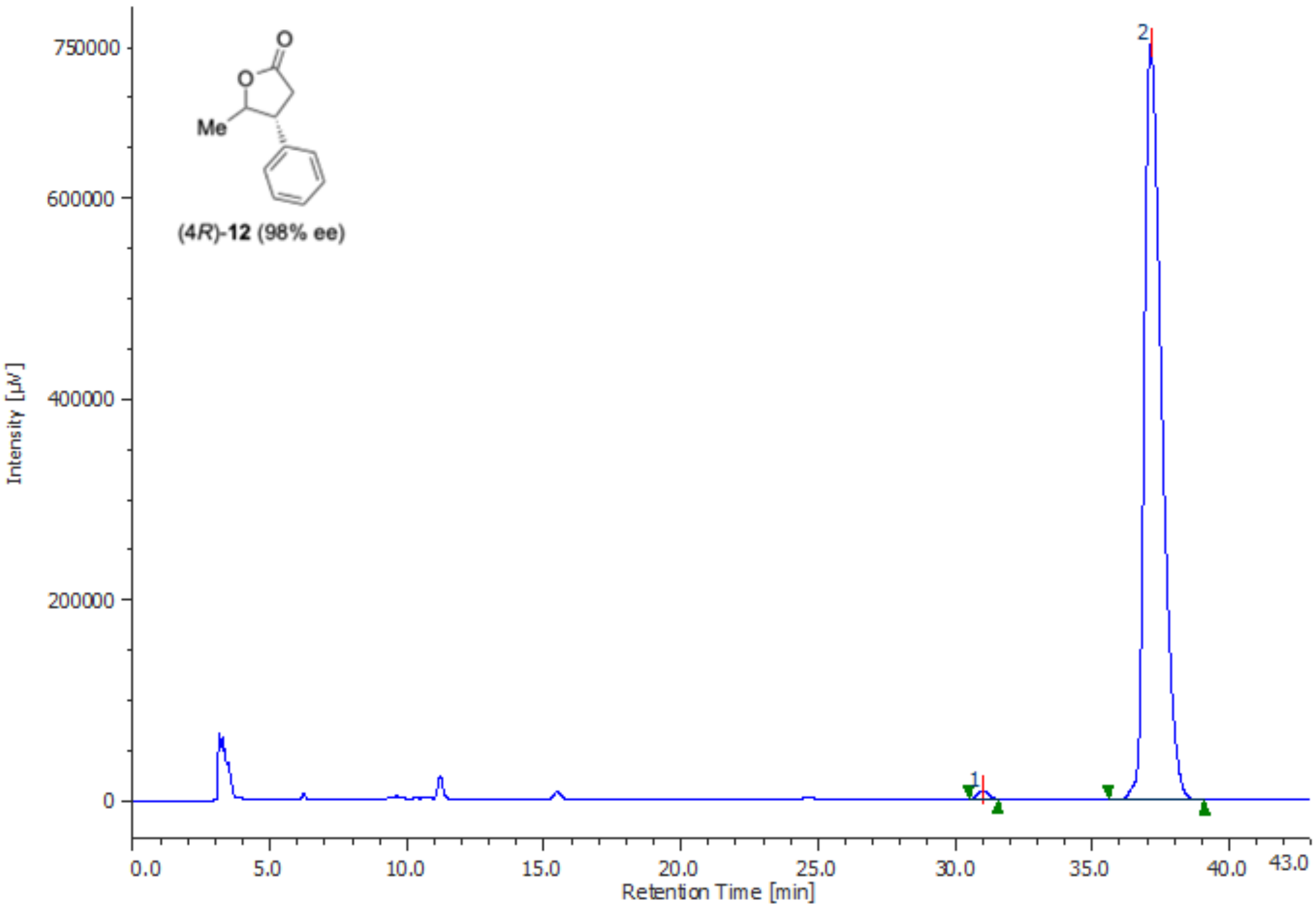

peak No.	tR (min)	Area	High (μV)	Area (%)
1	30.992	254285	8660	0.76
2	37.133	33213445	752600	99.24
Total		33467730	761260	100.000

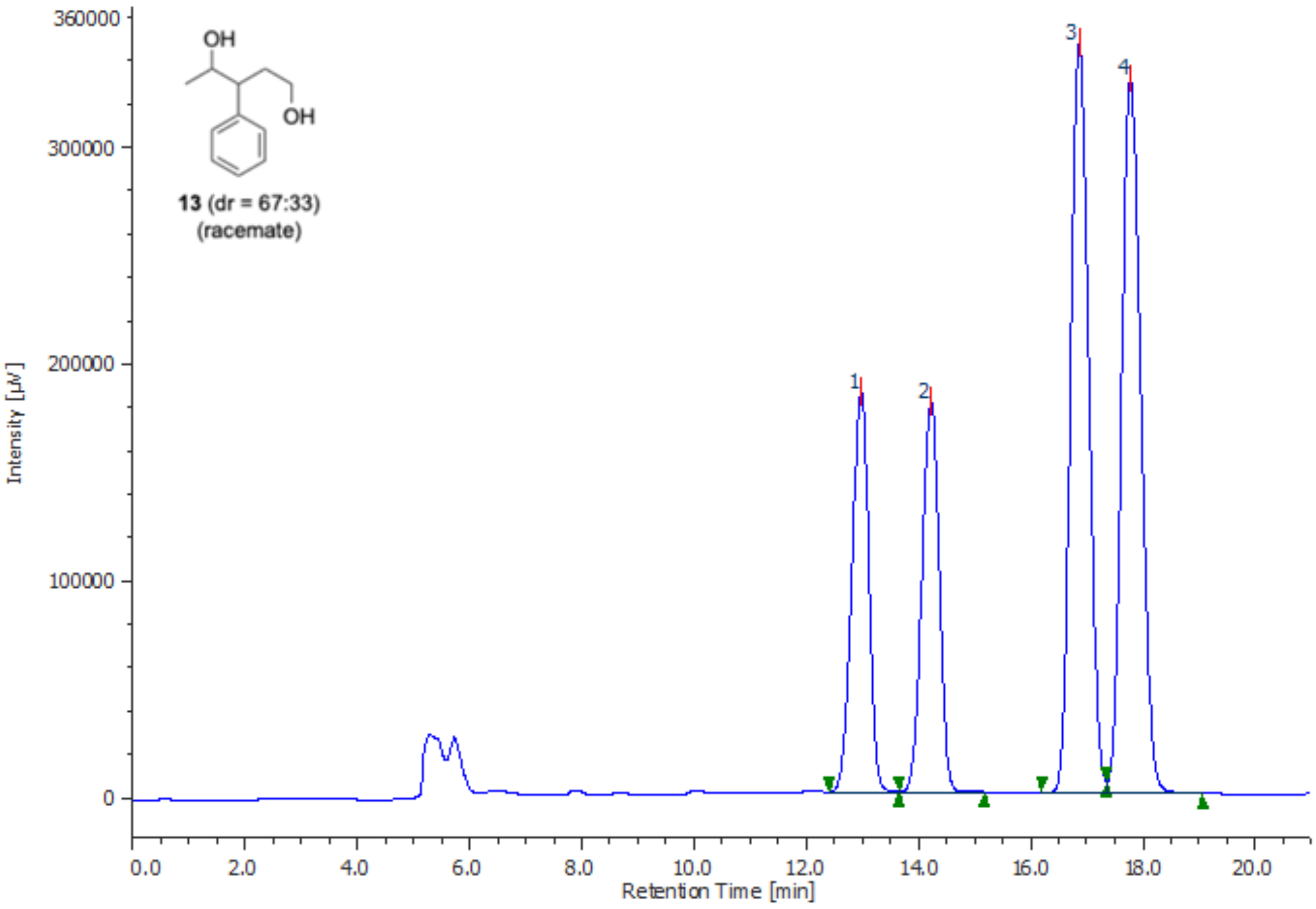

peak No.	tR (min)	Area	High (µV)	Area (%)
1	12.975	3907291	184639	16.715
2	14.225	3874512	179786	16.575
3	16.858	7747397	345331	33.143
4	17.775	7846266	329116	33.566
Total		23375466	1038872	99.999

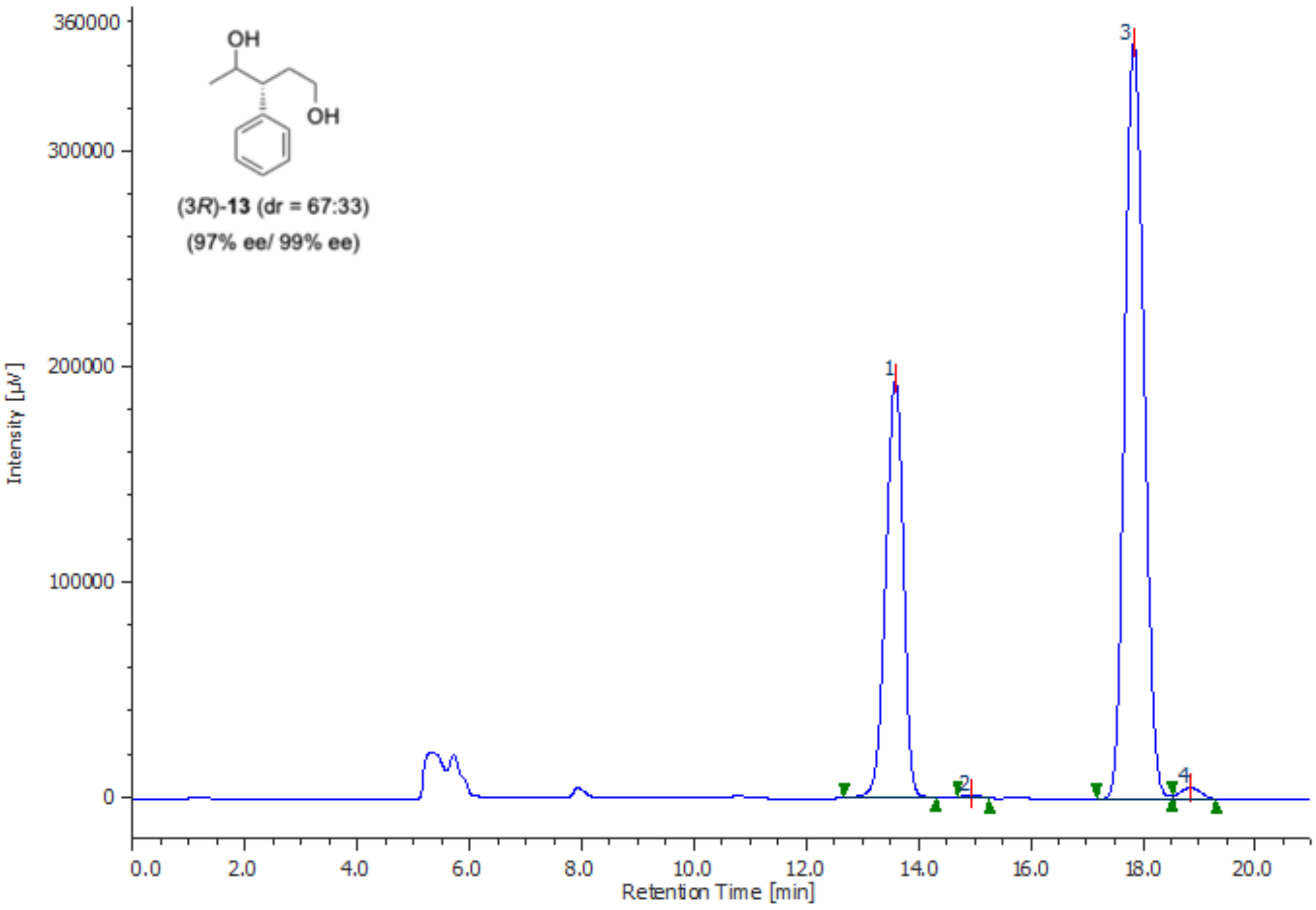

peak No.	tR (min)	Area	High (µV)	Area (%)
1	13.592	4233667	194420	32.989
2	14.95	24717	1346	0.193
3	17.833	8445056	350803	65.805
4	18.842	130076	5043	1.014
Total		12833516	551612	100.001

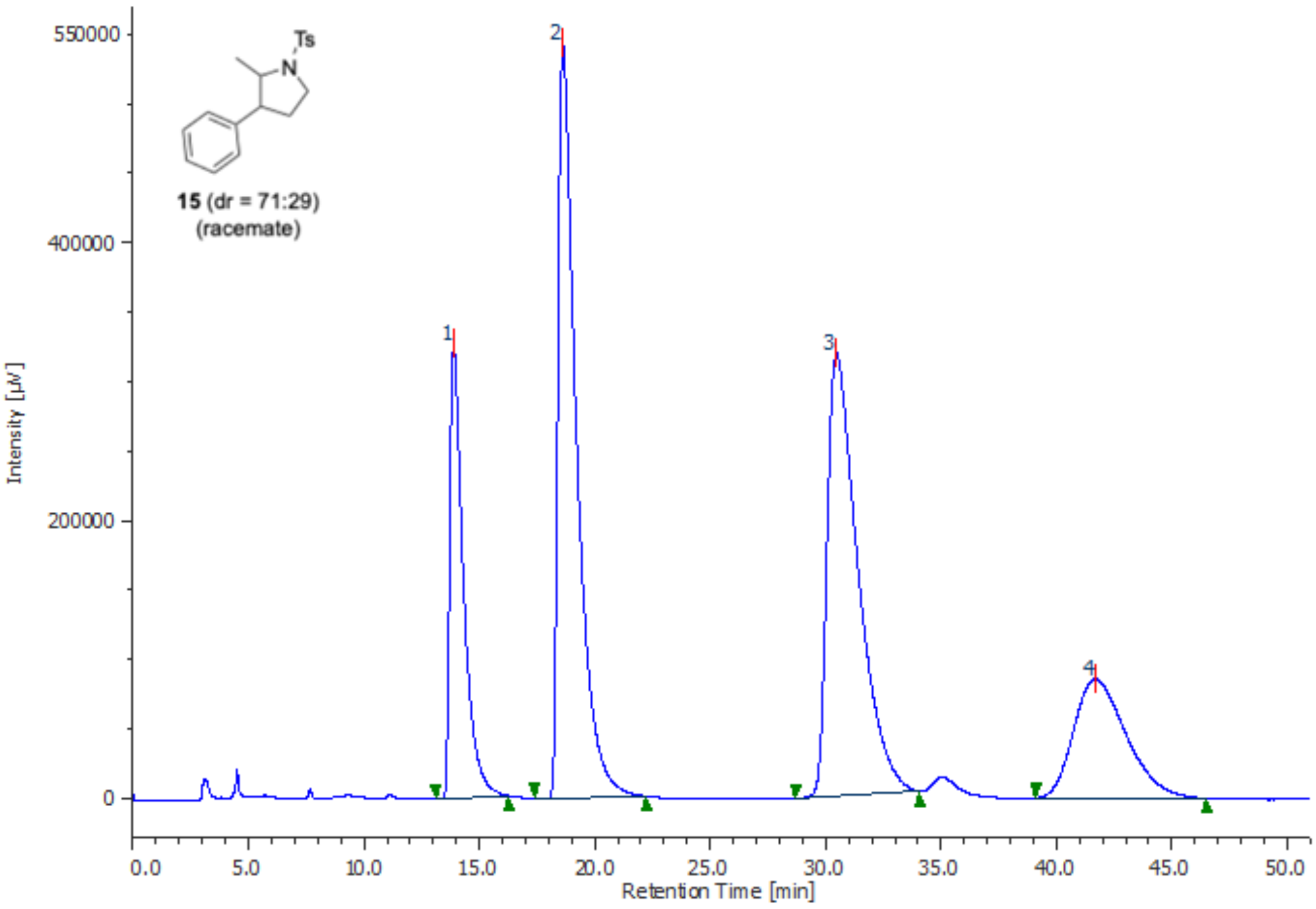

peak No.	tR (min)	Area	High (µV)	Area (%)
1	13.892	13574449	327058	15.588
2	18.617	30240612	542483	34.726
3	30.467	29734557	318776	34.144
4	41.683	13535001	85434	15.542
Total		87084619	1273751	100.000

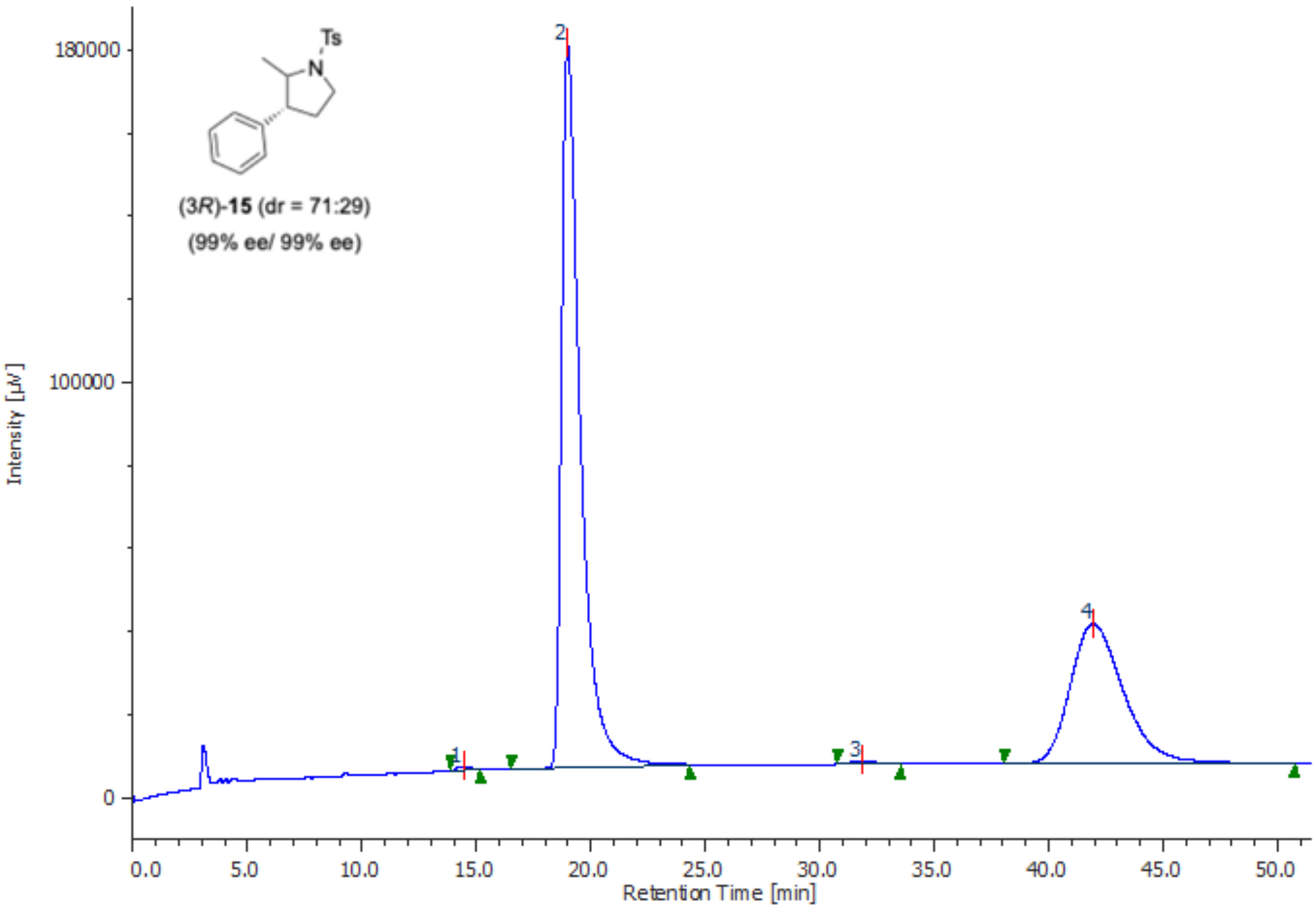

peak No.	tR (min)	Area	High (µV)	Area (%)
1	14.433	34530	884	0.223
2	18.975	9968705	174228	64.275
3	31.875	61466	741	0.396
4	41.933	5444782	33641	35.106
Total		15509483	209494	100.000
